# The design of a readily attachable and cleavable molecular scaffold for *ortho*-selective C–H alkenylation of arene alcohols†
†Electronic supplementary information (ESI) available. See DOI: 10.1039/c5sc03948g


**DOI:** 10.1039/c5sc03948g

**Published:** 2015-12-14

**Authors:** Brian J. Knight, Jacob O. Rothbaum, Eric M. Ferreira

**Affiliations:** a Department of Chemistry , University of Georgia , Athens , GA 30602 , USA . Email: emferr@uga.edu

## Abstract

We describe herein the design of a novel molecular scaffold that can induce facile oxidative olefinations when attached to alcohols. Benzylic, homo-, and bishomobenzylic alcohols are utilized. The scaffold can act as a protecting group for the alcohol in other transformations, and it is recoverable in excellent yield. The overall sequence can also be telescoped without purifications of intermediates, representing a net alcohol-based directed *ortho*-alkenylation.

## Abstract 2

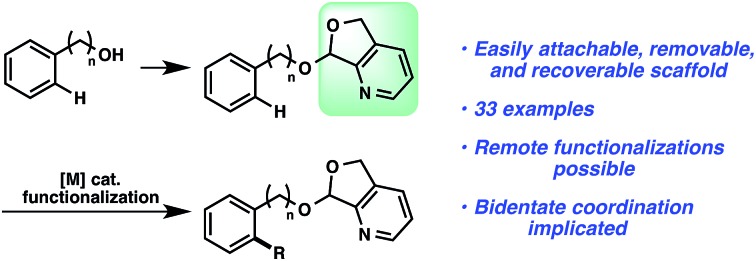
A novel molecular scaffold with distinct features (easily attachable, removable, recoverable) induces Pd-catalyzed oxidative olefinations when attached to arene alcohols.

## Introduction

The ability to forge an array of bond types *via* metal-catalyzed selective C–H functionalization is well appreciated.^1^ A substantial percentage of these transformations rely on the directing capability of the substrate. That is, the appropriate positioning of a Lewis basic functional group will present a metal catalyst center to a specific site, enabling the precise activation of the commonly unreactive C–H bond.^2^ Several commonly encountered functional groups can impart this directing capability. Alcohols can conceivably be one such group, but there are noted restrictions.^3^ Almost all cases of directed catalytic C–H functionalization employ *tertiary* alcohols;^4^ there are, to our knowledge, very few outliers using primary and secondary alcohols, with functionalizations in comparatively diminished yields.4a–c Implicit hypotheses suggest that this general substrate class is prone to oxidation, fragmentation, and other decomposition pathways.

**Fig. 1:**
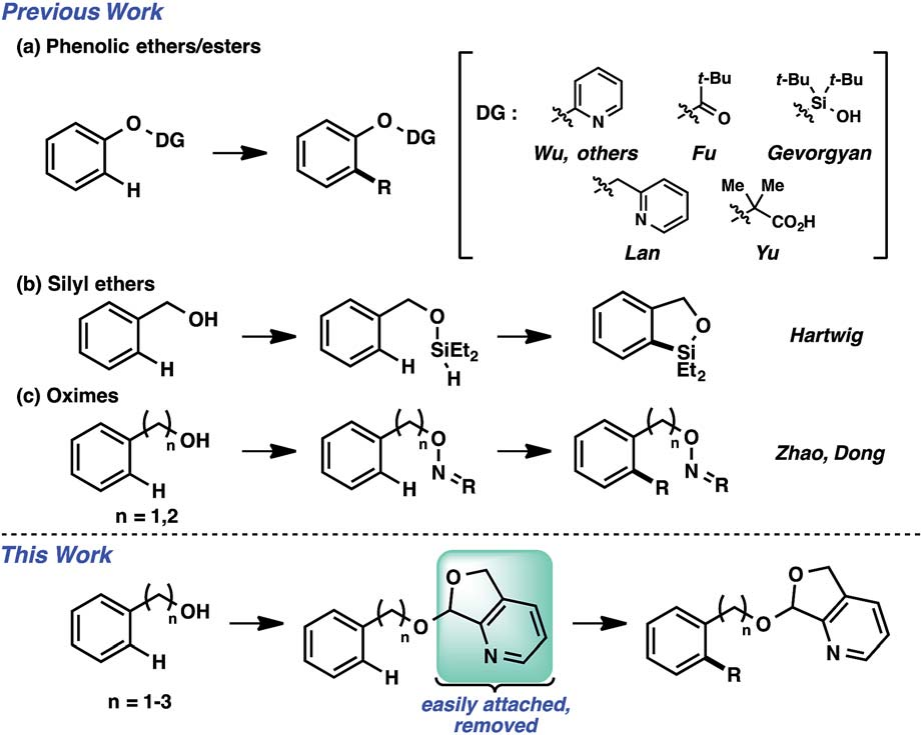
Surrogate approach to hydroxyl-directed C–H functionalization.

An alternative strategy is the utilization of alcohol surrogates, species that can be directly attached to and later removed from the alcohol functional group, which will permit metal-catalyzed functionalizations when connected.^5^ In addition to a few cases where this approach has been employed in sp^3^ C–H functionalization,^6^ this strategy has been applied toward arene sp^2^ C–H reactions. Interestingly, the large majority of examples have been based on phenolic precursors (Fig. 1a), including both Pd-catalyzed^7^–^11^ and non-Pd-catalyzed examples.^12^,^13^ In contrast, few catalytic sp^2^ C–H functionalizations have been based on aliphatic alcohol surrogates. Hartwig's iridium-catalyzed dehydrogenative functionalization with a silane is a landmark example (Fig. 1b).^14^ Tan^15^ and Yu^16^ have both developed attachable and cleavable groups for alcohols that induce *meta*-selective alkenylations. Recently, oximes have been demonstrated by both Zhao^17^ and Dong^18^ to be competent functional groups for Pd-catalyzed *ortho*-selective functionalizations of benzylic and homobenzylic alcohol precursors (Fig. 1c). In these latter oxime cases, they exploit the “*exo*-directing mode” of the functional group for cyclometalation to afford alkenylated, arylated, and acetoxylated products. Although the oxime has proven viable in this capacity, its synthetic manipulation does have its requisite demands (*e.g.*, generally 3 steps for the transformation of alcohol → oxime,^19^ lack of recoverability, reductive oxime removal), and thus surrogates of different design may offer unique advantages. Herein, we disclose the development of a novel molecular scaffold based on a heterocycle-incorporated acetal, delineating the structural features required to impart this desired type of reactivity. Additionally, we illustrate how the scaffold is readily attached and cleaved, highlighting the lability of this specific system and its translation to an overall streamlined process.

## Results and discussion

We anticipated at the onset that certain common functional groups may serve as straightforward solutions for our goal. It was expected that known protecting groups may exploit the potential Lewis basicity of the alcohol oxygen, or more likely, attach a functional group that endows its own directing capacity to induce the metalation. Our analysis of select groups in oxidative olefinations, using ligand-accelerated conditions inspired by the developments of Yu and coworkers^20^ (ethyl acrylate, 10 mol% Pd(OAc)_2_, 20 mol% Ac–Gly–OH, 3 equiv. AgOAc, HFIP, 90 °C), is depicted in Table 1. As can be seen, standard protecting groups were uniformly ineffectual. Ethers, acetals, esters, and carbamates all gave little to no reactivity. The di-*tert*-butyl-silanol group, which had been applied for phenol-based functionalization,^7^ also did not afford appreciable reactivity (entry 8). Pyridyl-incorporated esters and ethers also failed to induce directed olefination (entries 9, 10).

**Table 1:** C–H functionalization – evaluation of select alcohol surrogates

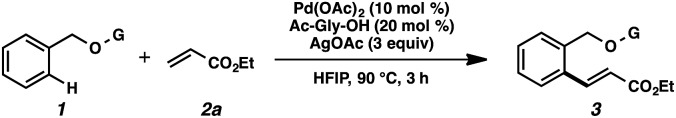					
Entry	G	NMR yield^*a*^ (%)	Entry	G	NMR yield^*a*^ (%)
1	H	0	9	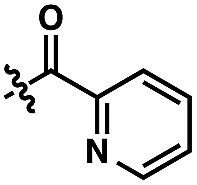	0
2	Me	6			
3	MOM	10	10	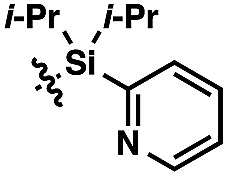	0
4	THP	0			
5	MEM	8			
6	Ac	0	11	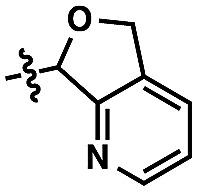	82^*b*^
7	CONH_*n*_–Pr	16			
8	Si(*t*-Bu)_2_OH	7			

^*a*^Based on 1-octene as an internal standard.

^*b*^Isolated yield – total olefinated product (51 : 31 mono/di).

**Scheme 1:**
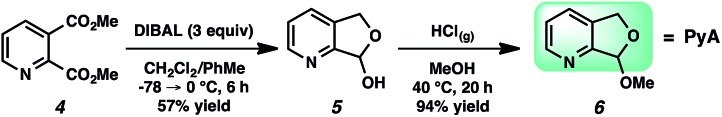
Pyridyl–acetal (PyA) synthesis.

For our goal to ultimately be realized, it was clear that we needed to devise a more unique solution. The design of an original scaffold to address this challenge required particular components: namely (1) a robust directing group, (2) a functional group to allow straightforward attachment and removal, and (3) structural features that would ideally induce proximal direction. Hybridizing these requirements led us to compound **6** (Scheme 1). Pyridines as intramolecular directing groups are well-established, the acetal linkage would enable simple connectivity, and the fused ring system may substantially lower the entropic barriers for the desired cyclometalation. To that end, we synthesized the PyA (**Py**ridyl–**A**cetal) scaffold, available in two steps from commercial dimethyl pyridine-2,3-dicarboxylate.^21^

**Scheme 2:**
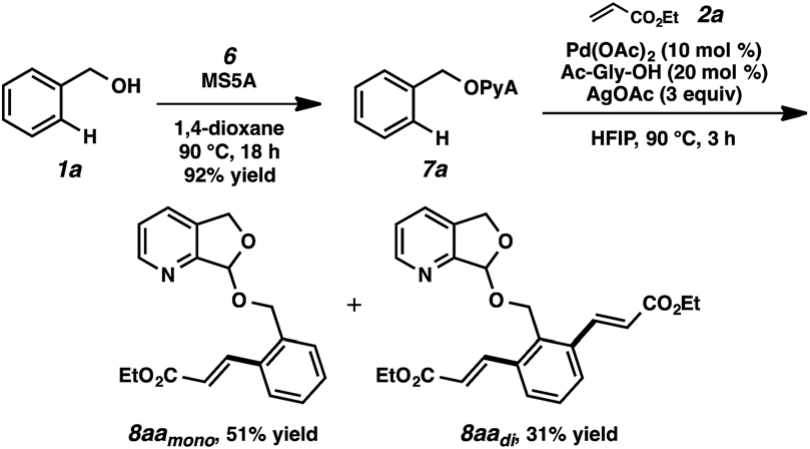
Initial test of PyA-based attachment and C–H olefination.

To our delight, we found that scaffold attachment to benzyl alcohol was straightforward using 5 Å molecular sieves, and subsequent Pd-catalyzed functionalization proceeded with marked improvement over the other cases (Table 1, entry 11 and Scheme 2). We were able to observe notably high levels of alkenylation, isolating monoolefin **8aa_mono_** and diolefin **8aa_di_** in 51% and 31% yield, respectively.^22^

A range of scaffold-attached benzylic alcohol substrates were evaluated in the oxidative olefination using either ethyl acrylate or *N*,*N*-dimethylacrylamide (Table 2). In cases where only monoolefination can occur (*i.e.*, *ortho*-substituted arenes), moderate to very good yields of alkene products were observed. In several of these cases, alcoholysis was also performed immediately following olefination (HCl, EtOH, 23 °C),^23^ and the resulting alcohols were formed in synthetically useful levels for the two-step sequence.^24^ Heterocycles are compatible with this reaction (*e.g.*, compound **8gb**). For cases where mono- and diolefination were possible, approximately 3 : 1 mixtures were generally produced, with monoolefination products being formed with ranging regioselectivities.^25^ Aryl bromides were incompatible with this process, likely due to competitive oxidative addition pathways. A secondary alcohol-based substrate was reactive and remarkably selective for monoalkenylation (**8lb**); a tertiary alcohol-based substrate, in contrast, was unproductive under these conditions.

**Table 2:** Substrate scope (arene)

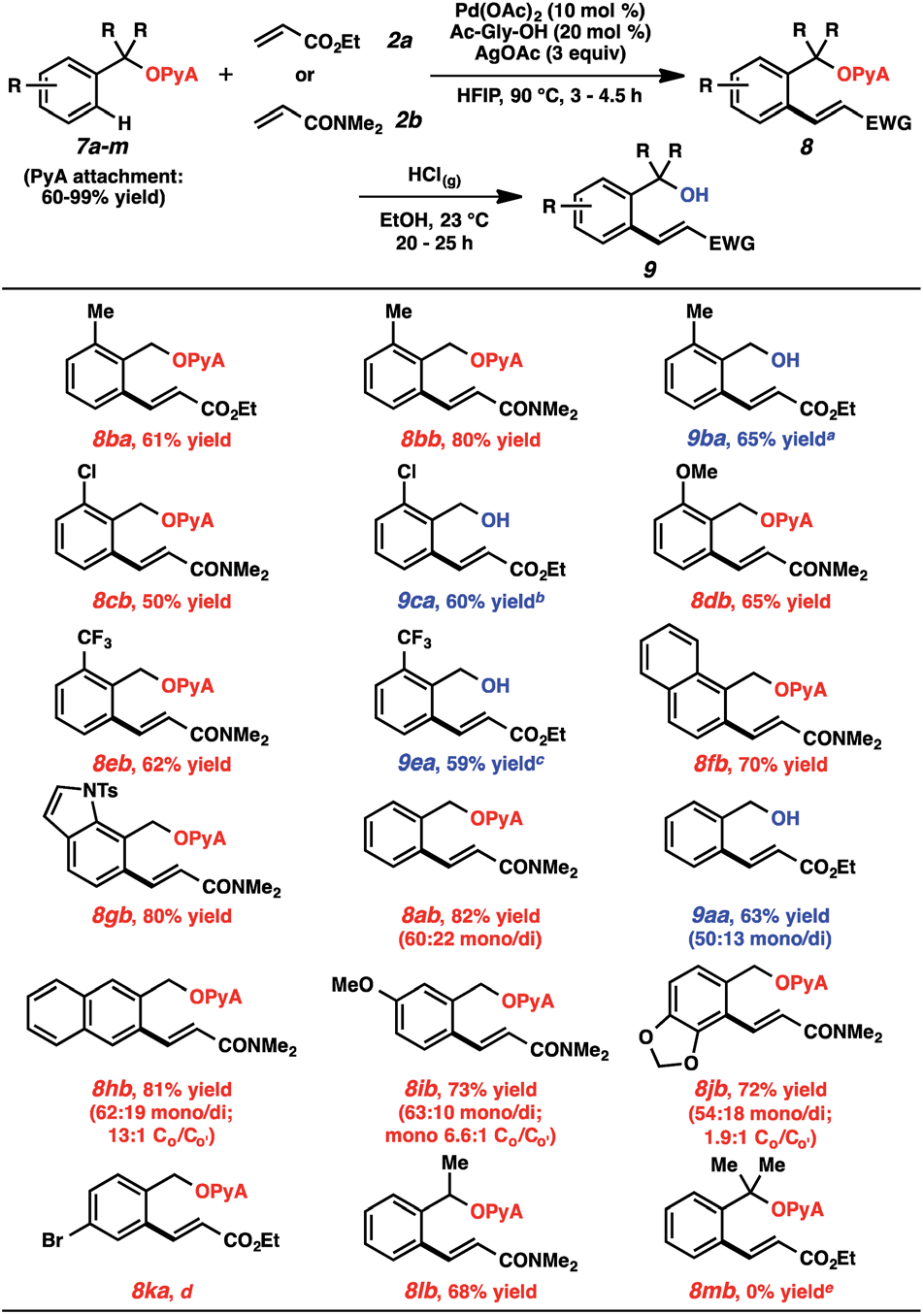

^*a*^17% yield of the nonolefinated alcohol was also recovered.

^*b*^36% yield of the nonolefinated alcohol was also recovered.

^*c*^24% yield of the nonolefinated alcohol was also recovered.

^*d*^A complex mixture was observed.

^*e*^The PyA group was attached on this hindered alcohol in only 29% yield.

Alkene partners were also evaluated (Table 3). In addition to ethyl acrylate and *N*,*N*-dimethylacrylamide, other acrylates and acrylamides were effective reactants (**8bc–g**). Electron-deficient styrenes were competent participants (**8bh**, **8bi**), while more electron-neutral ones afforded the products in diminished yields (**8bj**, **8bk**). Oxazolidinones and phosphine oxides were compatible (**8bl**, **8bm**), but acrylonitrile was only marginally reactive (**8bn**).^26^

**Table 3:** Substrate scope (alkene)

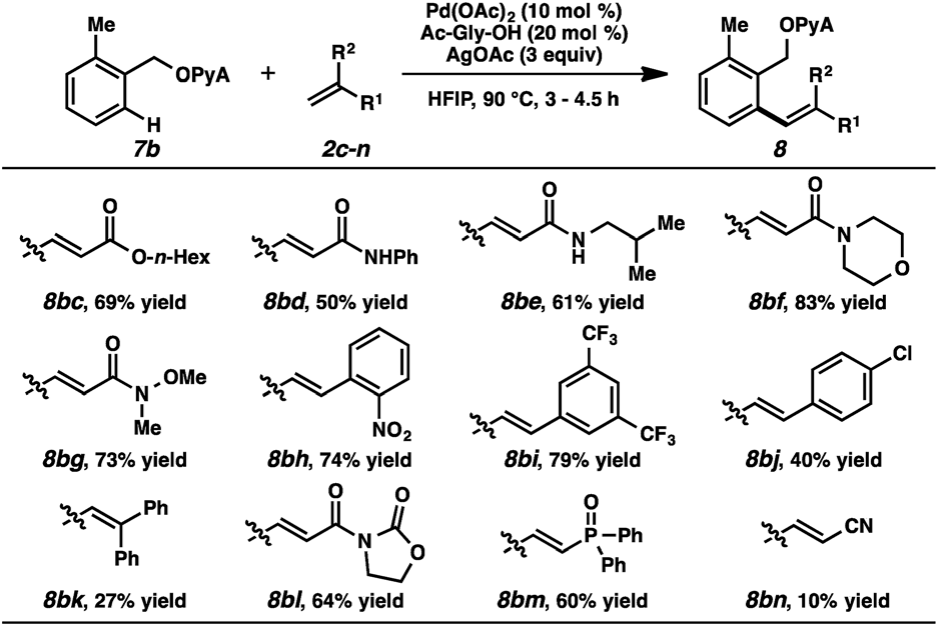

**Scheme 3:**
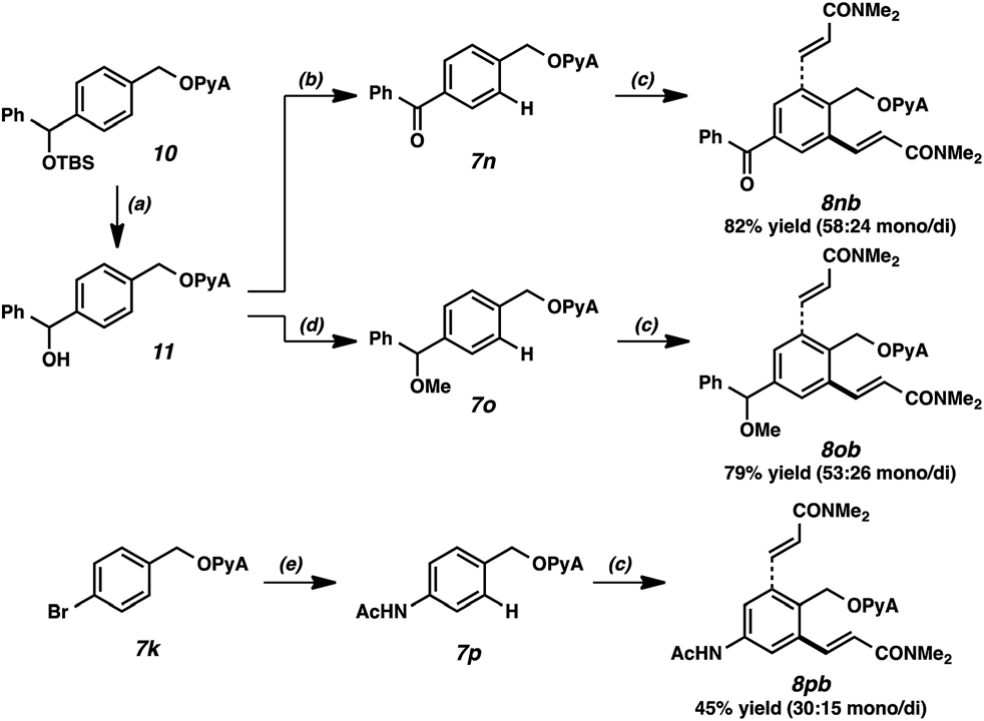
PyA scaffold as a protecting and directing group. (a) TBAF, THF, 23 °C, 3 h, 87% yield. (b) Dess–Martin periodinane, CH_2_Cl_2_, 23 °C, 1.5 h, 97% yield. (c) *N,N*-Dimethylacrylamide, Pd(OAc)_2_ (10 mol%), Ac-Gly-OH (20 mol%), AgOAc (3 equiv.), HFIP, 4.5 h, 90 °C. (d) NaH, MeI, THF, 0 °C, 2 h, 85% yield. (e) AcNH_2_, CuI (30 mol%), *N,N,N′,N′*-tetramethylethylenediamine (90 mol%), K_3_PO_4_, KI, DMF, 110 °C, 15 h, 95% yield.

It merits mention that the PyA scaffold can be employed as a protecting group akin to conventional acetal species. Scheme 3 is illustrative; desilylation, oxidation, etherification, and Cu-catalyzed amidation^27^ were all effective in the presence of this moiety. A key attribute of this scaffold is its ability to be leveraged in protecting group strategies. Alkenylations of the respective products proceeded smoothly to generate diversely functionalized olefinic compounds. Of note, under these alkenylation conditions, the PyA scaffold outcompetes the ketone, ether, and acetamide for reactivity, despite these latter functional groups' directing capacity for Pd-catalyzed functionalization.^28^

**Scheme 4:**
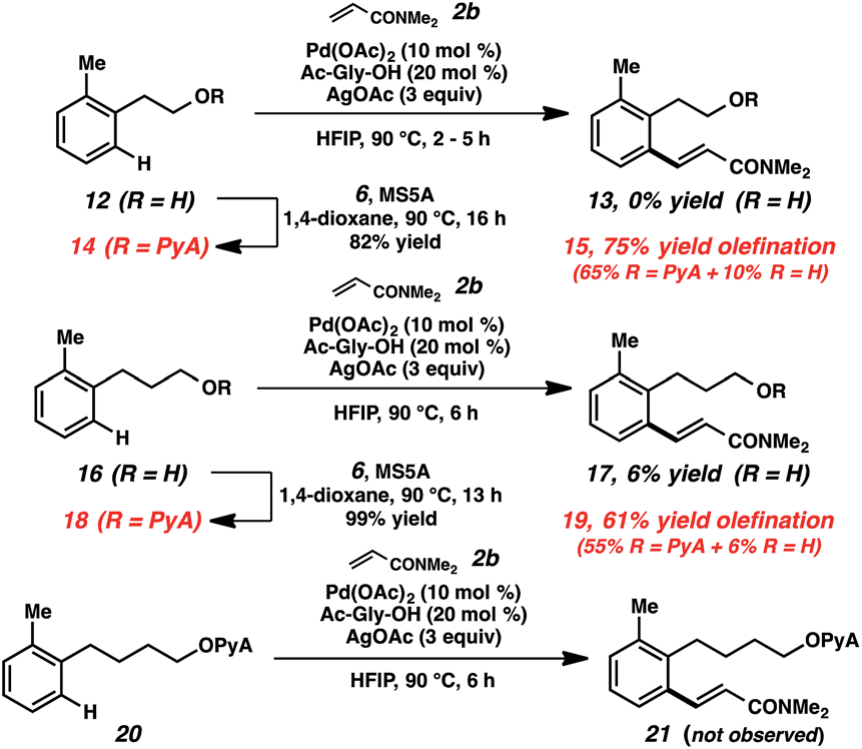
Remote functionalization using PyA scaffold.

Toward increasing the breadth of utility, we sought to expand the transformations to more remote cases (*i.e.*, beyond benzylic alcohols). Examples of these types of remote bond-forming events have been more rare, presumably due to the challenges of forming larger cyclometalated species. The substrates derived from both homobenzylic and bishomobenzylic alcohols can be effectively olefinated using this approach (Scheme 4).^29^ In comparison, when the parent alcohols (**12**, **16**) were subjected to the alkenylation conditions, little to no reactivity was observed. Preliminary attempts to functionalize the further-extended acetal (**20**) were also unsuccessful.

**Scheme 5:**
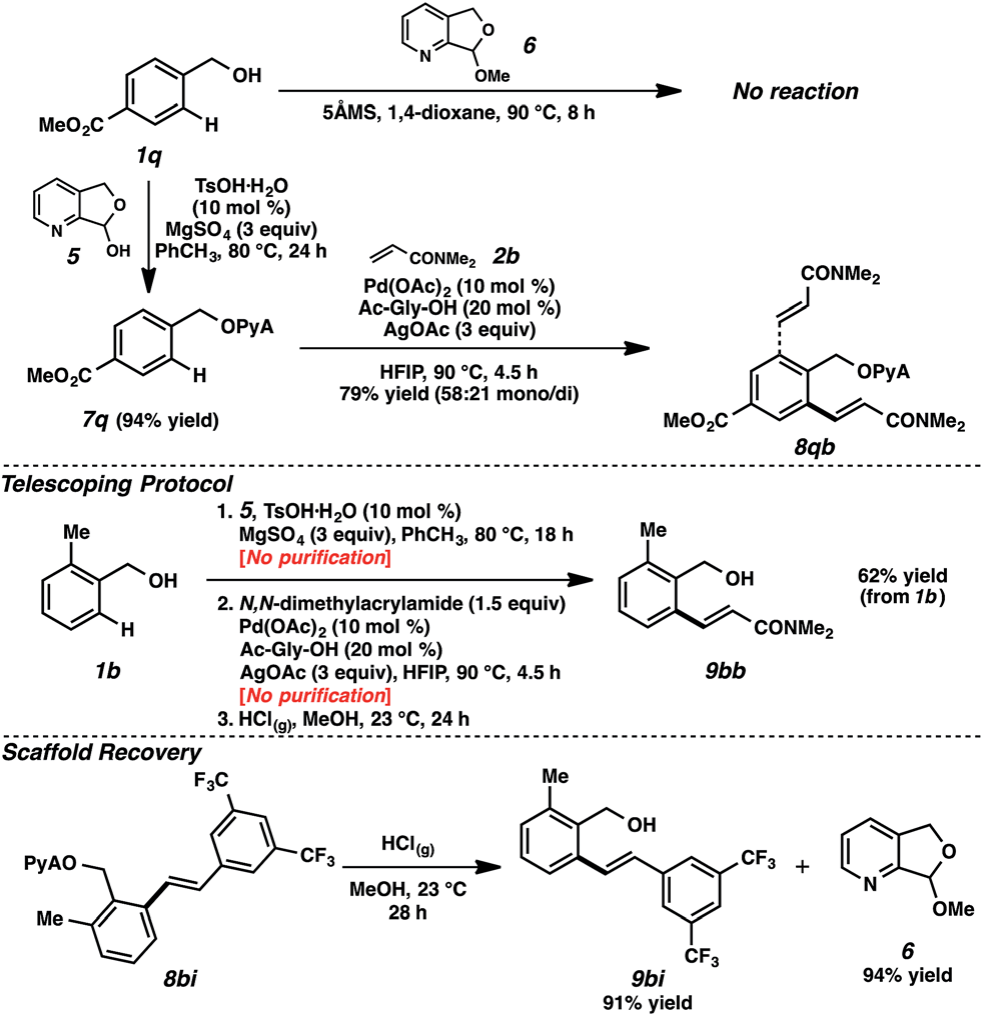
Alternative scaffold attachment method, telescoping, and recovery.

We encountered a limitation of this system when we attempted attaching particularly electron deficient alcohol substrates. For example, the use of alcohol **1q**, bearing an electron-withdrawing ester moiety, was ineffective in attachment using acetal **6** (Scheme 5).^30^ Gratifyingly, however, the application of hemiacetal **5** under dehydrative conditions (10 mol% TsOH·H_2_O, 3 equiv. MgSO_4_) circumvented this problem, and attachment and alkenylation were both possible.^31^ The ease of this hemiacetal approach, where residual hemiacetal **5** was readily removed in the reaction workup, also established a path for the execution of a telescoping sequence.^32^ As illustrated in Scheme 5, benzylic alcohol **1b** could be converted to the olefinic product without any intermediary purifications, representing a net alcohol-based directed *ortho*-functionalization.^33^ The scaffold can also be recovered in the alcoholysis, further highlighting the utility of this scaffold.

**Scheme 6:**
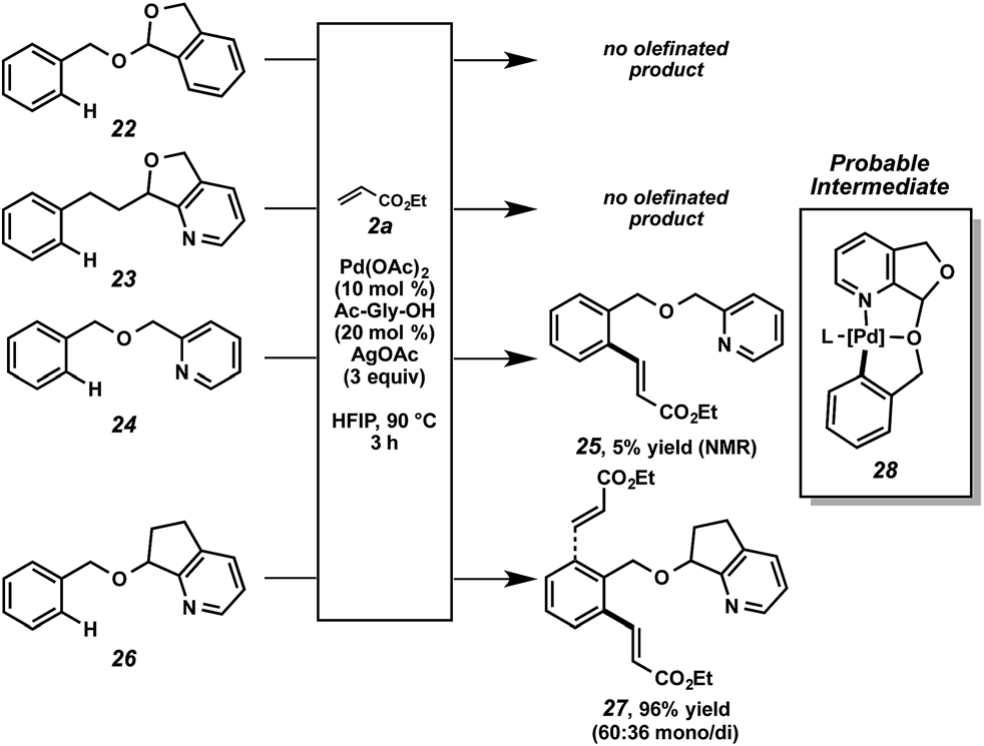
Reaction analysis of PyA scaffold variants.

The nature of the directing group merits further discussion. As mentioned above, we anticipated that this particular scaffold possessed specific features that rendered it uniquely effective for this function. Structural variants were revealing (Scheme 6). No reactivity was observed in the cases of acetal **22** or pyridine **23**.^34^ When pyridyl ether **24** was subjected to the alkenylation conditions, minimal reactivity was observed.^35^ When compound **26** was tested, however, high reactivity was restored. From this set of experiments, a few conclusions can be drawn. First, the bidentate capability of the nitrogen and oxygen atoms is likely important at some stage of the overall reaction.^36^ Second, the fused ring system decreases the conformational degrees of freedom, presumably encouraging a proximal relationship between the metal center and the C–H bond. Lastly, the acetal functional group is not important for catalytic functionalization and only facilitates attachment and cleavage. Based on this data, we believe cyclometalated species **28**, with scaffold bidentate coordination, is a probable intermediate in the overall alkenylation.^37^

## Conclusions

New strategies in catalytic C–H functionalizations that improve utility hold potential for achieving broad synthetic applicability. Our efforts herein outline a convenient and straightforward functionalization of alcohol-based substrates based on a simple molecular scaffolding approach. The scaffold is easily synthesized^38^ and is attachable and removable with high-yielding recovery. It has the capacity to serve as an alcohol protecting group, it can be incorporated into a telescoping process, and it can induce olefinations from notably remote positions on a molecule. We anticipate that these scaffold attributes will provide distinct advantages in catalytic functionalization chemistry.^39^ Further developments and applications in catalysis are underway and will be reported in due course.

## Supplementary Material

Supplementary information
