# Supplementary material for: The design of a readily attachable and cleavable molecular scaffold for ortho-selective C–H alkenylation of arene alcohols
Source: Chem Sci. 2015 Dec 14;7(3):1982–7. doi: 10.1039/c5sc03948g (PMC5966905; doi:10.1039/c5sc03948g)

Electronic Supplementary Material (ESI) for Chemical Science.
This journal is © The Royal Society of Chemistry 2015

Supporting Information

The Design of a Readily Attachable and Cleavable Molecular Scaffold for ortho-Selective C–H Alkenylation of Arene Alcohols

Brian J. Knight, Jacob O. Rothbaum, and Eric M. Ferreira*

Department of Chemistry, University of Georgia, Athens, GA 30602

emferr@uga.edu

Table of Contents

Materials and Methods S3

Scaffold Synthesis S4

Scaffold Attachment S6

Olefination of Benzylic Alcohol Acetals S14

Olefination Optimization Studies S37

Analysis of Benzylic Alcohol Protecting Groups and Directing Groups S43

The Scaffold as a Protecting Group S46

Structural Confirmation of Specifically Functionalized Compounds S52

Analysis of Competency of Potentially Competitive Directing Groups S54

Alcohol Substrate Synthesis S55

Alternative Scaffold Attachment Method, Telescoping, and Scaffold Recovery S57

Additional Analysis of Acetal Cleavage S62

Structural Variants to Probe the Nature of the Directing Group S63

Spectra Compilation S69

Materials and Methods

Reactions were performed under an argon atmosphere unless otherwise noted. Tetrahydrofuran, dichloromethane, diethyl ether, and toluene were purified by passing through activated alumina columns. 1,4-Dioxane was stored over activated 3ÅMS. Anhydrous HCl gas was regularly prepared by adding concentrated HCl to anhydrous $CaCl_2$. All other reagents were used as received unless otherwise noted. Commercially available chemicals were purchased from Alfa Aesar (Ward Hill, Ma), Sigma-Aldrich (St. Louis, MO), Oakwood Products (West Columbia, SC), Strem (Newport, MA), and TCI America (Portland, OR). Qualitative TLC and preparatory plate chromatography was performed on 250 mm thick, 60 Å, glass backed, F254 silica (Silicycle, Quebec City, Canada). Visualization was accomplished with UV light and exposure to $KMnO_4$, CAM, or Dragendorff stain solutions followed by heating. Flash column chromatography were performed using Silicylce silica gel (230-400 mesh). ^{1}H NMR spectra were acquired on either a Varian Mercury 300 (at 300 MHz), a Varian 400 MR (at 400 MHz), Varian Mercury Plus (at 400 MHz), or a Varian Unity Inova (at 500 MHz) and are reported relative to $SiMe_4$ (δ 0.00). ^{13}C NMR spectra were acquired on a Varian 400 MR (at 100 MHz), Varian Mercury Plus (at 100 MHz), or a Varian Unity Inova (at 125 MHz) and are reported relative to $SiMe_4$ (δ 0.0). ^{19}F NMR spectra were acquired on a Varian 400 MR (at 376 MHz) and are reported relative to $PhCF_3$ (δ -63.7). All IR spectra were obtained as a film with a Nicolet iS50 FT-IR or an IRPrestige-21 Spectrophotometer. Low-resolution mass spectrometry data were acquired on an Agilent 1200 series HPLC equipped with an Agilent 6130 quadrupole LC/MS. High-resolution mass spectrometry data was acquired by the Colorado State University Central Instrument Facility on an Agilent 6210 TOF LC/MS or by the University of Georgia Proteomics and Mass Spectrometry Core Facility on a Bruker Esquire 3000 Plus Ion Trap Spectrophotometer.

Scaffold Synthesis

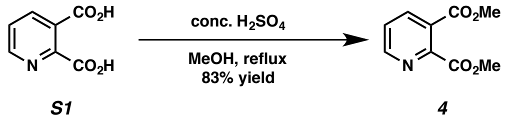

To a suspension of pyridine-2,3-dicarboxylic acid (**S1**, 30.0 g, 180 mmol) in MeOH (120 mL) at 0 °C was slowly added concentrated H_2SO_4 (12.0 mL), and then the suspension was stirred and heated at reflux for 20 h. The reaction solution was cooled to 0 °C and was slowly neutralized with sat. aq. Na_2CO_3 (200 mL). The MeOH was removed by rotary evaporation, and the organic materials were extracted with CH_2Cl_2 (6 x 100 mL). The combined organic layers were dried over $MgSO_4$ and concentrated to afford analytically pure dimethyl ester **4** (29.3 g, 83% yield, R_f = 0.35 in 1:1 hexanes/EtOAc) as a beige solid.

Dimethyl ester 4: ^{1}H NMR (400 MHz, $CDCl_3$) δ 8.76 (dd, *J* = 4.8, 2.0 Hz, 1H), 8.17 (dd, *J* = 8.0, 2.0 Hz, 1H), 7.49 (dd, *J* = 8.0, 4.8 Hz, 1H), 4.00 (s, 3H), 3.94 (s, 3H); ^{13}C NMR (100 MHz, $CDCl_3$) δ 166.7, 165.9, 151.9, 150.8, 137.7, 126.6, 125.1, 53.3, 53.1; IR (film) 1726, 1426, 1284, 1131, 1077 cm^{-1}; LRMS (ESI+) *m/z* calc'd for $(M + H)^+$ $[C_9H_9NO_4 + H]^+$: 196.1, found 196.1.

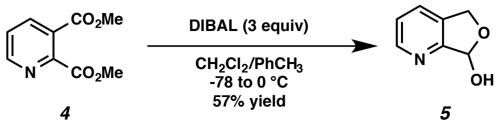

To a stirring suspension of diester **4** (7.29 g, 37.4 mmol) in CH_2Cl_2 (146 mL) cooled to -78 °C was added diisobutylaluminum hydride (85.0 mL, 1.32 M in $PhCH_3$, 112 mmol) dropwise over 10 min. The solution was stirred for 5 h at -78 °C, and was then warmed to 0 °C and stirred for 30 min. To the reaction was carefully added water (4.37 mL) dropwise, then 3.8 M aq. NaOH (4.37 mL) dropwise, and finally water (13.1 mL). The suspension was warmed to ambient temperature and was stirred for 20 min before $MgSO_4$ (~80 g) and celite (~40 g) were added. Stirring was stopped, and the mixture was allowed to sit overnight. The suspension was then filtered through a glass-fritted funnel, and the solids were washed with excess MeOH (100 mL). The filter cake was collected and heated in MeOH (~300 mL, approx. reflux) for ~5 min before refiltering and washing with excess hot MeOH (~150 mL) until the solution passing the filter was no longer UV active by TLC. This washing procedure was repeated 4-5 times to sufficiently remove the product material from the residual solids. (*Note:* Hemiacetal **5** is highly water soluble, and thus measures were taken to avoid aqueous treatments in workup procedures.) The combined filtrates were concentrated by rotary evaporation and purified by flash column chromatography (20:1 EtOAc/MeOH eluent) to afford hemiacetal **5** (2.92 g, 57% yield, R_f = 0.24 in EtOAc) as a beige solid.

Hemiacetal 5 (characterized as a 10:1 mixture of hemiacetal/aldehyde): ^{1}H NMR (400 MHz, $CDCl_3$) δ 10.17 (s, 0.10H), 8.71 (d, *J* = 4.4 Hz, 0.10H), 8.53 (d, *J* = 5.0 Hz, 1H), 7.99 (d, *J* = 7.6 Hz, 0.10H), 7.62 (d, *J* = 7.8 Hz, 1H), 7.50 (dd, *J* = 7.6, 4.4 Hz, 0.10H), 7.29 (dd, *J* = 7.8, 5.0 Hz, 1H), 6.57 (s, 1H), 6.12 (br. s, 1.1H), 5.30 (d, *J* = 13.2 Hz, 1H), 5.05 (d, *J* = 13.2 Hz, 1H), 4.94 (s, 0.20H); ^{13}C NMR (100 MHz, $CDCl_3$) δ 196.5, 158.8, 149.7, 149.1, 148.8, 139.0, 136.9, 133.1, 130.8, 127.6, 123.7, 98.7, 70.1, 61.9; IR (film) 3179, 1425, 1070, 1005, 911 cm^{-1}; HRMS (ESI+) *m/z* calc'd for $(M + H)^+$ $[C_7H_7NO_2 + H]^+$: 138.0550; found 138.0551.

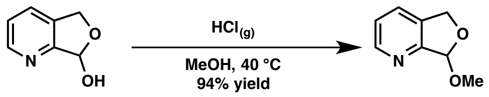

A round-bottomed flask holding a solution of hemiacetal **5** (3.25 g, 23.7 mmol) in MeOH (240 mL) at 23 °C was bubbled anhydrous HCl for 1 min. The flask was capped (yellow plastic cap), and the mixture was heated and stirred at 40 °C for 20 h. Upon completion, the reaction was poured into a mixture of ~1:1 ice/sat. aq. Na_2CO_3 (400 mL), and it was then concentrated by rotary evaporation to remove MeOH. The mixture was extracted with EtOAc (3 x 200 mL), and the combined organic layers were washed with brine (50 mL), dried over $MgSO_4$, and concentrated by rotary evaporation. The crude mixture was purified by flash column chromatography (EtOAc eluent) to afford methyl acetal **6** (3.35 g, 94% yield, R_f = 0.43 in EtOAc) as an amber oil.

Methyl acetal 6: ^{1}H NMR (400 MHz, $CDCl_3$) δ 8.58 (dd, *J* = 4.8, 1.2 Hz, 1H), 7.63 (dd, *J* = 7.6, 1.2 Hz, 1H), 7.27 (dd, *J* = 7.6, 4.8 Hz, 1H), 6.04 (d, *J* = 2.0 Hz, 1H) 5.23 (dd, *J* = 13.2, 2.0 Hz, 1H), 5.08 (d, *J* = 13.2 Hz, 1H), 3.56 (s, 3H); ^{13}C NMR (100 MHz, $CDCl_3$) δ 157.5, 150.0, 133.1, 129.9, 123.7, 105.9, 70.6, 55.9; IR (film) 1374, 1087, 1023, 1007, 962 cm^{-1}; HRMS (ESI+) *m/z* calc'd for $(M + H)^+$ $[C_8H_9NO_2 + H]^+$: 152.0706, found 152.0706.

Scaffold Attachment

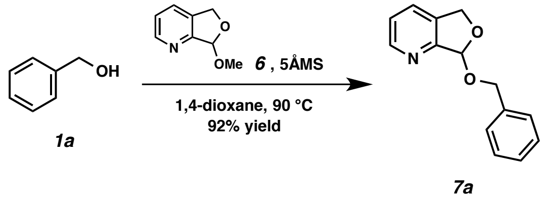

In a flask with a condenser attached, a suspension of benzyl alcohol (**1a**, 340 mg, 3.15 mmol), methyl acetal **6** (502 mg, 3.32 mmol), and 5Å molecular sieves (3.93 g) in 1,4-dioxane (5.00 mL) was heated at 90 °C (without stirring) for 18 h. Upon completion, the reaction was cooled to ambient temperature, and EtOAc (~30 mL) and celite (~3.0 g) were added to the mixture. This suspension was filtered through a pad of celite (65 mm x 10 mm) with 9:1 EtOAc/MeOH (300 mL), the filtrate concentrated by rotary evaporation, and the residue purified by flash column chromatography (1:1 hexanes/EtOAc eluent) to afford acetal **7a** (661 mg, 92% yield, R_f = 0.39 in 1:1 hexanes/EtOAc) as a colorless oil.

Acetal 7a: ^{1}H NMR (400 MHz, $CDCl_3$) δ 8.59 (dd, *J* = 4.8, 1.2 Hz, 1H) 7.63 (dd, *J* = 7.6, 4.8 Hz, 1H), 7.46-7.41 (comp. m, 2H), 7.38-7.31 (comp. m, 2H), 7.30-7.25 (comp. m, 2H), 6.23 (d, *J* = 2.0 Hz, 1H), 5.27 (dd, *J* = 13.2, 2.0 Hz, 1H), 5.09 (d, *J* = 13.2 Hz, 1H), 4.85 (ABq, *J* = 11.6 Hz, Δν = 25.5 Hz, 2H); ^{13}C NMR (100 MHz, $CDCl_3$) δ 157.7, 149.9, 137.7, 133.3, 129.9, 128.5, 128.3, 127.9, 123.6, 104.3, 70.5, 70.3; IR (film) 2870, 1589, 1364, 1073, 1006 cm^{-1}; HRMS (ESI+) *m/z* calc'd for $(M + H)^+$ $[C_{14}H_{13}NO_2 + H]^+$: 228.1019, found 228.1024.

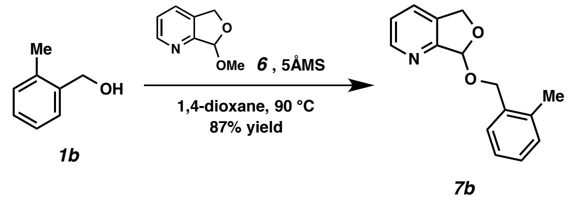

In a flask with a condenser attached, a suspension of 2-methylbenzyl alcohol (**1b**, 778 mg, 6.37 mmol), methyl acetal **6** (1.01 g, 6.69 mmol), and 5Å molecular sieves (7.96 g) in 1,4-dioxane (10.1 mL) was heated at 90 °C (without stirring) for 18 h. Upon completion, the reaction was cooled to ambient temperature, and EtOAc (~60 mL) and celite (~6.0 g) were added to the mixture. This suspension was filtered through a pad of celite (65 mm x 20 mm) with 9:1 EtOAc/MeOH (400 mL), the filtrate concentrated by rotary evaporation, and the residue purified by flash column chromatography (7:3 hexanes/EtOAc eluent) to afford acetal **7b** (1.34 g, 87% yield, R_f = 0.36 in 3:2 hexanes/EtOAc) as a colorless oil.

Acetal 7b: ^{1}H NMR (400 MHz, $CDCl_3$) δ 8.58 (d, *J* = 4.8 Hz, 1H), 7.62 (d, *J* = 7.6 Hz, 1H), 7.43-7.39 (comp. m, 1H), 7.26 (dd, *J* = 7.6, 4.8 Hz, 1H), 7.26-7.14 (comp. m, 3H), 6.22 (d, *J* = 2.0 Hz, 1H), 5.27 (dd, *J* = 13.2, 2.0 Hz, 1H), 5.09 (d, *J* = 13.2 Hz, 1H), 4.92 (d, *J* = 11.2 Hz, 1H), 4.79 (d, *J* = 11.2 Hz, 1H), 2.41 (s, 3H); ^{13}C NMR (100 MHz, $CDCl_3$) δ 157.8, 149.9, 137.1, 135.6, 133.2, 130.4, 129.8, 129.3, 128.1, 126.0, 123.6, 104.3, 70.5, 68.8, 19.1; IR (film) 1588, 1363, 1075, 1000, 745 cm^{-1}; HRMS (ESI+) *m/z* calc'd for $(M + H)^+$ $[C_{15}H_{15}NO_2 + H]^+$: 242.1183, found 242.1176.

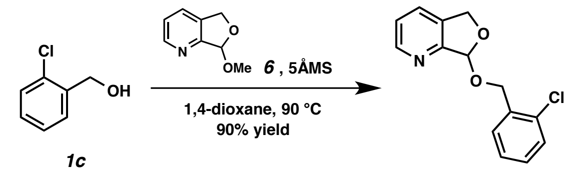

In a 2-dram vial with a PTFE-lined cap, a suspension of 2-chlorobenzyl alcohol (**1c**, 71.3 mg, 0.500 mmol), methyl acetal **6** (79.4 mg, 0.525 mmol), and 5Å molecular sieves (625 mg) in 1,4-dioxane (0.794 mL) was heated at 90 °C (without stirring) for 15 h. Upon completion, the reaction was cooled to ambient temperature, and EtOAc (~5.0 mL) and celite (~500 mg) were added to the mixture. This suspension was filtered through a pad of celite (65 mm x 10 mm) with 9:1 EtOAc/MeOH (200 mL), the filtrate concentrated by rotary evaporation, and the residue purified by flash column chromatography (7:3 hexanes/EtOAc eluent) to afford acetal **7c** (118 mg, 90% yield, R_f = 0.34 in 7:3 hexanes/EtOAc) as a beige solid.

Acetal 7c: ^{1}H NMR (400 MHz, $CDCl_3$) δ 8.61 (d, *J* = 4.4 Hz, 1H), 7.66 (d, *J* = 7.6 Hz, 1H), 7.58 (d, *J* = 7.2 Hz, 1H), 7.34 (d, *J* = 7.6 Hz, 1H), 7.29 (dd, *J* = 7.6, 4.4 Hz, 1H), 7.27-7.18 (comp. m, 2H), 6.27 (s, 1H), 5.29 (d, *J* =13.2 Hz, 1H), 5.11 (d, *J* = 13.2 Hz, 1H), 4.95 (ABq, *J* =12.8 Hz, Δν = 33.3 Hz, 2H); ^{13}C NMR (100 MHz, $CDCl_3$) δ 157.5, 150.0, 135.7, 133.3, 133.0, 130.0, 129.5, 129.3, 128.8, 126.9, 123.7, 104.7, 70.6, 67.3; IR (film) 1088, 1007, 914, 791, 752 cm^{-1}; HRMS (ESI+) *m/z* calc'd for $(M + H)^+$ $[C_{14}H_{12}ClNO_2 + H]^+$: 262.0629, found 262.0631.

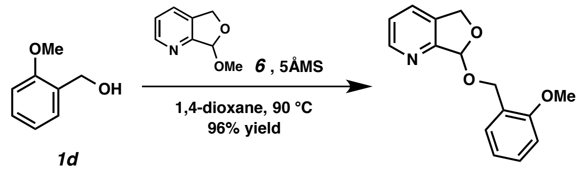

In a 2-dram vial with a PTFE-lined cap, a suspension of 2-methoxybenzyl alcohol (**1d**, 69.4 mg, 0.500 mmol), methyl acetal **6** (79.4 mg, 0.525 mmol), and 5Å molecular sieves (625 mg) in 1,4-dioxane (0.794 mL) was heated at 90 °C (without stirring) for 8 h. Upon completion, the reaction was cooled to ambient temperature, and EtOAc (~5.0 mL) and celite (~500 mg) were added to the mixture. This suspension was filtered through a pad of celite (65 mm x 10 mm) with 9:1 EtOAc/MeOH (150 mL), the filtrate concentrated by rotary evaporation, and the residue purified by flash column chromatography (7:3 hexanes/EtOAc eluent) to afford acetal **7d** (123 mg, 96% yield, R_f = 0.16 in 7:3 hexanes/EtOAc) as a white solid.

Acetal 7d: ^{1}H NMR (400 MHz, $CDCl_3$) δ 8.59 (d, *J* = 4.8 Hz, 1H), 7.63 (d, *J* = 7.6 Hz, 1H), 7.49 (d, *J* = 7.6 Hz, 1H), 7.30-7.23 (comp. m, 2H), 6.95 (t, *J* = 7.6 Hz, 1H), 6.86 (d, *J* = 8.0 Hz, 1H), 6.27 (s, 1H), 5.28 (d, *J* = 13.2 Hz, 1H), 5.09 (d, *J* = 13.2 Hz, 1H), 4.91 (ABq, *J* = 12.4 Hz, Δν = 18.7 Hz, 2H), 3.84 (s, 3H); ^{13}C NMR (100 MHz, $CDCl_3$) δ 157.8, 157.2, 149.8, 133.3, 129.8, 129.3, 128.8, 126.2, 123.5, 120.5, 110.3, 104.4, 70.4, 65.1, 55.5; IR (film) 1495, 1236, 1076, 999, 752 cm^{-1}; HRMS (ESI+) *m/z* calc'd for $(M + H)^+$ $[C_{15}H_{15}NO_3 + H]^+$: 258.1125, found 258.1129.

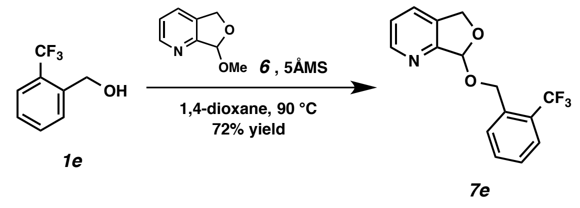

In a 2-dram vial with a PTFE-lined cap, a suspension of 2-(trifluoromethyl)benzyl alcohol (**1e**, 88.6 mg, 0.500 mmol), methyl acetal **6** (79.4 mg, 0.525 mmol), and 5Å molecular sieves (625 mg) in 1,4-dioxane (0.794 mL) was heated at 90 °C (without stirring) for 15 h. Upon completion, the reaction was cooled to ambient temperature, and EtOAc (~5.0 mL) and celite (~500 mg) were added to the mixture. This suspension was filtered through a pad of celite (65 mm x 10 mm) with 9:1 EtOAc/MeOH (150 mL), the filtrate concentrated by rotary evaporation, and the residue purified by flash column chromatography (7:3 hexanes/EtOAc eluent) to afford acetal **7e** (106 mg, 72% yield, R_f = 0.40 in 3:2 hexanes/EtOAc) as a colorless oil.

Acetal 7e: ^{1}H NMR (400 MHz, $CDCl_3$) δ 8.61 (d, J = 4.8 Hz, 1H), 7.81 (d, J = 7.6 Hz, 1H), 7.65 (d, J = 7.6 Hz, 1H), 7.62 (d, J = 7.6 Hz, 1H), 7.54 (t, J = 7.6 Hz, 1H), 7.36 (t, J = 7.6 Hz, 1H), 7.29 (dd, J = 7.6, 4.8 Hz, 1H), 6.26 (d, J = 2.0 Hz, 1H), 5.27 (dd, J = 13.6, 2.0 Hz, 1H), 5.10 (d, J = 13.6 Hz, 1H), 5.04 (ABq, J = 13.2 Hz, Δν = 36.1 Hz, 2H); ^{13}C NMR (100 MHz, $CDCl_3$) δ 157.4, 150.0, 136.7, 133.3, 132.2, 130.0, 129.4, 127.5 (q, $^2J_{C\text{-}F}$ = 30.5 Hz), 127.4, 125.7 (q, $^3J_{C\text{-}F}$ = 5.7 Hz), 124.5 (q, $^1J_{C\text{-}F}$ = 272.0 Hz), 123.8, 104.7, 70.6, 66.1 (q, $^4J_{C\text{-}F}$ = 2.9 Hz); IR (film) 1314, 1119, 1007, 910, 731 cm^{-1}; HRMS (ESI+) m/z calc'd for $(M + H)^+$ $[C_{15}H_{12}F_3NO_2 + H]^+$: 296.0893, found 296.0892.

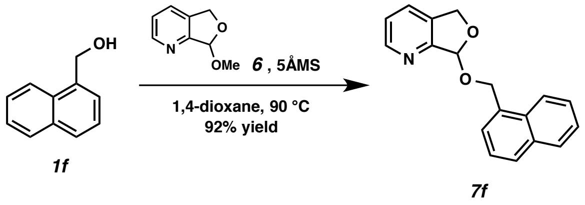

In a 2-dram vial with a PTFE-lined cap, a suspension of 1-naphthalenemethanol (**1f**, 47.5 mg, 0.300 mmol), methyl acetal **6** (47.6 mg, 0.315 mmol), and 5Å molecular sieves (375 mg) in 1,4-dioxane (0.476 mL) was heated at 90 °C (without stirring) for 48 h. Upon completion, the reaction was cooled to ambient temperature, and EtOAc (~5.0 mL) and celite (~500 mg) were added to the mixture. This suspension was filtered through a pad of celite (65 mm x 10 mm) with 9:1 EtOAc/MeOH (150 mL), the filtrate concentrated by rotary evaporation, and the residue purified by flash column chromatography (3:2 hexanes/EtOAc eluent) to afford acetal **7f** (277 mg, 92% yield, R_f = 0.35 in 3:2 hexanes/EtOAc) as a white solid.

Acetal 7f: ^{1}H NMR (400 MHz, $CDCl_3$) δ 8.58 (d, J = 4.4 Hz, 1H), 8.22 (d, J = 8.0 Hz, 1H), 7.85 (d, J = 8.0 Hz, 1H), 7.80 (d, J = 8.0 Hz, 1H), 7.63 (d, J = 8.0 Hz, 1H), 7.61 (d, J = 8.0 Hz, 1H), 7.52 (t, J = 8.0 Hz, 1H), 7.48 (t, J = 8.0 Hz, 1H), 7.44 (t, J = 8.0 Hz, 1H), 7.26 (dd, J = 8.0, 4.4 Hz, 1H), 6.35 (s, 1H), 5.41 (d, J = 11.4 Hz, 1H), 5.32 (d, J = 13.0 Hz, 1H), 5.21 (d, J = 11.4 Hz, 1H), 5.14 (d, J = 13.0 Hz, 1H); ^{13}C NMR (100 MHz, $CDCl_3$) δ 157.6, 149.9, 133.9, 133.3, 133.2, 131.9, 129.9, 128.9, 128.6, 127.1, 126.4, 125.8, 125.4, 124.1, 123.7, 104.5, 70.7, 68.6; IR (film) 1088, 1003, 912, 793, 779 cm^{-1}; HRMS (ESI+) m/z calc'd for $(M + H)^+$ $[C_{18}H_{15}NO_2 + H]^+$: 278.1176, found 278.1177.

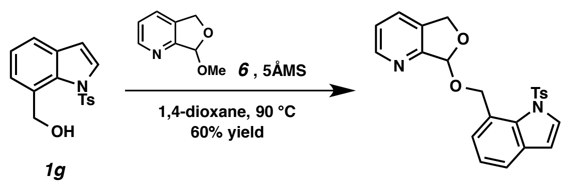

1g *7g*

In a 2-dram vial with a PTFE-lined cap, a suspension of alcohol **1g** (121 mg, 400 mmol), methyl acetal **6** (63.5 mg, 0.420 mmol), and 5Å molecular sieves (500 mg) in 1,4-dioxane (0.635 mL) was heated at 90 °C (without stirring) for 18 h. Upon completion, the reaction was cooled to ambient temperature, and EtOAc (~5.0 mL) and celite (~500 mg) were added to the mixture. This suspension was filtered through a pad of celite (65 mm x 10 mm) with 9:1 EtOAc/MeOH (150 mL), the filtrate concentrated by rotary evaporation, and the residue purified by flash column chromatography (3:2 hexanes/EtOAc eluent) to afford acetal **7g** (101 mg, 60% yield, R_f = 0.27 in 3:2 hexanes/EtOAc) as a white foam.

Acetal 7g: ^{1}H NMR (400 MHz, $CDCl_3$) δ 8.60 (d, *J* = 4.6 Hz, 1H), 7.66 (d, *J* = 3.6 Hz, 1H), 7.65 (d, *J* = 7.6 Hz, 1H), 7.61 (d, *J* = 7.6 Hz, 1H), 7.53 (d, *J* = 8.4 Hz, 2H), 7.40 (d, *J* = 7.6 Hz, 1H), 7.28 (dd, *J* = 7.6, 4.6 Hz, 1H), 7.23 (t, *J* = 7.6 Hz, 1H), 7.13 (d, *J* = 8.4 Hz, 2H), 6.67 (d, *J* = 3.6 Hz, 1H), 6.27 (s, 1H), 5.27 (s, 2H), 5.26 (d, *J* = 13.2 Hz, 1H), 5.08 (d, *J* = 13.2 Hz, 1H), 2.31 (s, 3H); ^{13}C NMR (100 MHz, $CDCl_3$) δ 157.7, 149.8, 144.7, 135.8, 133.4, 133.3, 133.2, 130.2, 129.9, 129.8, 126.7, 126.1, 125.3, 124.3, 123.6, 120.7, 110.4, 104.5, 70.6, 67.2, 21.7; IR (film) 1363, 1173, 1089, 1005, 910 cm^{-1}; HRMS (ESI+) *m/z* calc'd for $(M + H)^+$ $[C_{23}H_{20}N_2O_4S + H]^+$: 421.1217, found 421.1211.

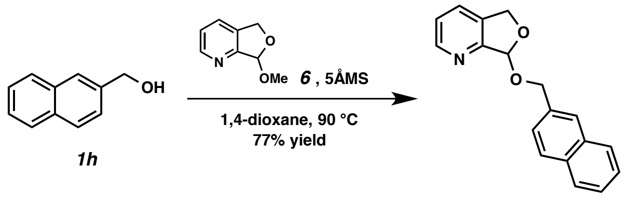

1h *7h*

In a 2-dram vial with a PTFE-lined cap, a suspension of 2-naphthalenemethanol (**1h**, 47.5 mg, 0.300 mmol), methyl acetal **6** (47.6 mg, 0.315 mmol), and 5Å molecular sieves (375 mg) in 1,4-dioxane (0.476 mL) was heated at 90 °C (without stirring) for 11 h. Upon completion, the reaction was cooled to ambient temperature, and EtOAc (~5.0 mL) and celite (~500 mg) were added to the mixture. This suspension was filtered through a pad of celite (65 mm x 10 mm) with 9:1 EtOAc/MeOH (150 mL), the filtrate concentrated by rotary evaporation, and the residue purified by flash column chromatography (7:3 hexanes/EtOAc eluent) to afford acetal **7h** (64.2 mg, 77% yield, R_f = 0.21 in 7:3 hexanes/EtOAc) as a white solid.

Acetal 7h: ^{1}H NMR (400 MHz, $CDCl_3$) δ 8.60 (d, *J* = 4.6 Hz, 1H), 7.88 (s, 1H), 7.86-7.79 (comp. m, 3H), 7.65 (d, *J* = 7.6 Hz, 1H), 7.57 (d, *J* = 8.4 Hz, 1H), 7.49-7.44 (comp. m, 2H), 7.28 (dd, *J* = 7.6, 4.6 Hz, 1H), 6.29 (s, 1H), 5.29 (d, *J* = 13.2 Hz, 1H), 5.10 (d, *J* = 13.2 Hz, 1H), 5.01 (ABq, *J* = 11.8 Hz, Δν = 24.7 Hz, 2H); ^{13}C NMR (100 MHz, $CDCl_3$) δ 157.6, 149.9, 135.2, 133.4, 133.3, 133.1, 129.9, 128.3, 128.1, 127.8, 127.1, 126.2, 126.1, 126.0, 123.7, 104.2, 70.6, 70.4; IR (film) 1367, 1078, 1005, 910, 729 cm^{-1}; HRMS (ESI+) *m/z* calc'd for $(M + H)^+$ $[C_{18}H_{15}NO_2 + H]^+$: 278.1176, found 278.1176.

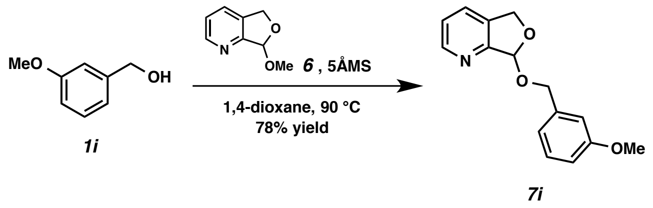

In a 2-dram vial with a PTFE-lined cap, a suspension of 3-methoxybenzyl alcohol (**1i**, 55.3 mg, 0.400 mmol), methyl acetal **6** (63.5 mg, 0.420 mmol), and 5Å molecular sieves (500 mg) in 1,4-dioxane (0.635 mL) was heated at 90 °C (without stirring) for 8.5 h. Upon completion, the reaction was cooled to ambient temperature, and EtOAc (~5.0 mL) and celite (~500 mg) were added to the mixture. This suspension was filtered through a pad of celite (65 mm x 10 mm) with 9:1 EtOAc/MeOH (150 mL), the filtrate concentrated by rotary evaporation, and the residue purified by flash column chromatography (3:2 hexanes/EtOAc eluent) to afford acetal **7i** (80.5 mg, 78% yield, R_f = 0.14 in 3:2 hexanes/EtOAc) as a colorless oil.

Acetal 7i: ^{1}H NMR (400 MHz, $CDCl_3$) δ 8.57 (d, *J* = 4.0 Hz, 1H), 7.62 (d, *J* = 7.6 Hz, 1H), 7.26 (dd, *J* = 7.6, 4.0 Hz, 1H), 7.24 (t, *J* = 8.0 Hz, 1H), 7.00 (d, *J* = 8.0 Hz, 1H), 6.99 (s, 1H), 6.81 (d, *J* = 8.0 Hz, 1H), 6.22 (s, 1H), 5.25 (d, *J* = 13.2 Hz, 1H), 5.08 (d, *J* = 13.2 Hz, 1H), 4.81 (ABq, *J* = 11.6 Hz, Δν = 24.6 Hz, 2H), 3.79 (s, 3H); ^{13}C NMR (100 MHz, $CDCl_3$) δ 159.8, 157.6, 149.8, 139.2, 133.2, 129.9, 129.5, 123.6, 120.5, 113.7, 113.4, 104.2, 70.5, 70.2, 55.3; IR (film) 1491, 1445, 1250, 1001, 727 cm^{-1}; HRMS (ESI+) *m/z* calc'd for $(M + H)^+$ $[C_{15}H_{15}NO_3 + H]^+$: 258.1125, found 258.1126.

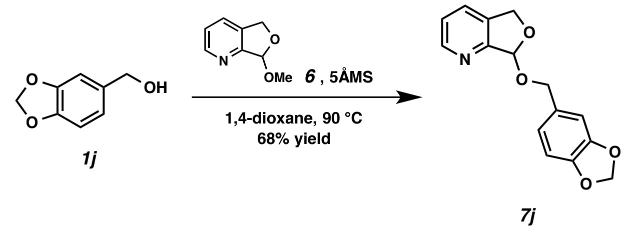

In a 2-dram vial with a PTFE-lined cap, a suspension of alcohol **1j** (55.3 mg, 0.400 mmol), methyl acetal **6** (63.5 mg, 0.420 mmol), and 5Å molecular sieves (500 mg) in 1,4-dioxane (0.635 mL) was heated at 90 °C (without stirring) for 12 h. Upon completion, the reaction was cooled to ambient temperature, and EtOAc (~5.0 mL) and celite (~500 mg) were added to the mixture. This suspension was filtered through a pad of celite (65 mm x 10 mm) with 9:1 EtOAc/MeOH (150 mL), the filtrate concentrated by rotary evaporation, and the residue purified by flash column chromatography (1:1 hexanes/EtOAc eluent) to afford acetal **7j** (73.9 mg, 68% yield, R_f = 0.32 in 1:1 hexanes/EtOAc) as a white solid.

Acetal 7j: ^{1}H NMR (400 MHz, $CDCl_3$) δ 8.56 (d, *J* = 4.8 Hz, 1H), 7.60 (d, *J* = 7.6 Hz, 1H), 7.24 (dd, *J* = 7.6, 4.8 Hz, 1H), 6.93 (s, 1H), 6.87 (d, *J* = 8.0 Hz, 1H), 6.75 (d, *J* = 8.0 Hz, 1H), 6.18 (d, *J* = 2.0 Hz, 1H), 5.91 (s, 2H), 5.24 (dd, *J* = 13.2, 2.0 Hz, 1H), 5.06 (d, *J* = 13.2 Hz, 1H), 4.72 (ABq, *J* = 11.2 Hz, Δν = 27.3 Hz, 2H); ^{13}C NMR (100 MHz, $CDCl_3$) δ 157.6, 149.8, 147.8, 147.3, 133.1, 131.4, 129.8, 123.5, 121.9, 109.1, 108.1, 103.9, 101.0, 70.4, 70.2; IR (film) 1491, 1445, 1250, 1001, 727 cm^{-1}; HRMS (ESI+) *m/z* calc'd for $(M + H)^+$ $[C_{15}H_{13}NO_4 + H]^+$: 272.0917, found 272.0918.

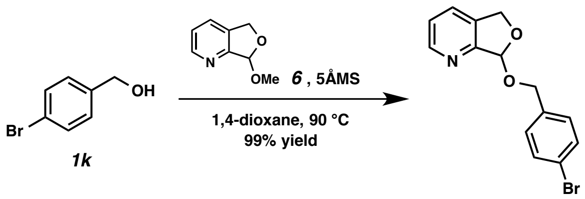

1k *7k*

In a 2-dram vial with a PTFE-lined cap, a suspension of 4-bromobenzyl alcohol (**1k**, 56.1 mg, 0.300 mmol), methyl acetal **6** (47.6 mg, 0.315 mmol), and 5Å molecular sieves (375 mg) in 1,4-dioxane (0.476 mL) was heated at 90 °C (without stirring) for 4 h. Upon completion, the reaction was cooled to ambient temperature, and EtOAc (~5.0 mL) and celite (~500 mg) were added to the mixture. This suspension was filtered through a pad of celite (65 mm x 10 mm) with 9:1 EtOAc/MeOH (150 mL), the filtrate concentrated by rotary evaporation, and the residue purified by flash column chromatography (3:2 EtOAc/hexanes eluent) to afford acetal **7k** (90.1 mg, 99% yield, R_f = 0.36 in 3:2 EtOAc/hexanes) as a white solid.

Acetal 7k: ^{1}H NMR (400 MHz, $CDCl_3$) δ 8.59 (d, *J* = 4.8 Hz, 1H), 7.63 (d, *J* = 7.6 Hz, 1H), 7.45 (d, *J* = 8.4 Hz, 2H), 7.29 (d, *J* = 8.4 Hz, 2H), 7.28 (dd, *J* = 7.6, 4.8 Hz, 1H), 6.20 (d, *J* = 1.6 Hz, 1H), 5.25 (dd, *J* = 13.2, 1.6 Hz, 1H), 5.09 (d, *J* = 13.2 Hz, 1H), 4.78 (ABq, *J* = 12.0 Hz, Δν = 23.7 Hz, 2H); ^{13}C NMR (100 MHz, $CDCl_3$) δ 157.4, 149.9, 136.8, 133.2, 131.6, 130.0, 129.8, 123.7, 121.7, 104.3, 70.6, 69.5; IR (film) 2922, 1489, 1070, 1007, 795 cm^{-1}; HRMS (ESI+) *m/z* calc'd for $(M + H)^+$ $[C_{14}H_{12}BrNO_2 + H]^+$: 306.0124, found 306.0130.

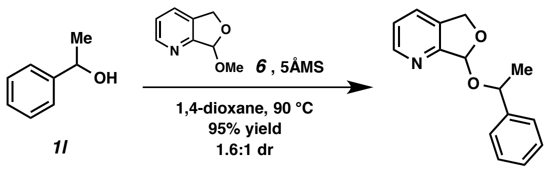

1l *7l*

In a 2-dram vial with a PTFE-lined cap, a suspension of α-methylbenzyl alcohol (**1l**, 36.7 mg, 0.300 mmol), methyl acetal **6** (47.6 mg, 0.315 mmol), and 5Å molecular sieves (375 mg) in 1,4-dioxane (0.476 mL) was heated at 90 °C (without stirring) for 18 h. Upon completion, the reaction was cooled to ambient temperature, and EtOAc (~5.0 mL) and celite (~500 mg) were added to the mixture. This suspension was filtered through a pad of celite (65 mm x 10 mm) with 9:1 EtOAc/MeOH (150 mL), the filtrate concentrated by rotary evaporation, and the residue purified by flash column chromatography (3:2 hexanes/EtOAc eluent) to afford acetal **7l** (major diastereomer, 42.4 mg, 59% yield, R_f = 0.33 in 3:2 hexanes/EtOAc) as a colorless oil and acetal **7l** (minor diastereomer, 26.3 mg, 36% yield, R_f = 0.21 in 3:2 hexanes/EtOAc) as a colorless oil that slowly transformed to a white solid.

Acetal 7l (major diastereomer): ^{1}H NMR (400 MHz, $CDCl_3$) δ 8.59 (d, *J* = 4.6 Hz, 1H), 7.51 (d, *J* = 7.6 Hz, 1H), 7.51 (d, *J* = 7.6 Hz, 2H), 7.41 (t, *J* = 7.6 Hz, 2H), 7.30 (t, *J* = 7.6 Hz, 1H), 7.25 (dd, *J* = 7.6, 4.6 Hz, 1H), 5.96 (d, *J* = 1.6 Hz, 1H), 5.25 (dd, *J* = 13.2, 1.6 Hz, 1H), 5.06 (q, *J* = 6.4 Hz, 1H), 5.03 (d, *J* = 13.2 Hz, 1H), 1.52 (d, *J* = 6.4 Hz, 3H); ^{13}C NMR (100 MHz, $CDCl_3$) δ 158.0, 149.9, 142.8, 133.3, 129.8, 128.7, 127.8, 126.8, 123.5, 102.0, 75.3, 70.2, 24.5; IR (film) 1082, 1001, 914, 764, 702 cm^{-1}; HRMS (ESI+) *m/z* calc'd for $(M + H)^+$ $[C_{15}H_{15}NO_2 + H]^+$: 242.1176, found 242.1177.

Acetal 7l (minor diastereomer): ^{1}H NMR (400 MHz, $CDCl_3$) δ 8.59 (d, *J* = 4.8 Hz, 1H), 7.61 (d, *J* = 7.6 Hz, 1H), 7.40 (d, *J* = 7.3 Hz, 2H), 7.34 (t, *J* = 7.3 Hz, 2H), 7.29-7.23 (comp. m, 2H), 6.27 (d, *J* = 1.6 Hz, 1H), 5.11 (dd, *J* = 13.2, 1.6 Hz, 1H), 5.06 (q, *J* = 6.4 Hz, 1H), 4.95 (d, *J* = 13.2 Hz, 1H), 1.60

(d, J = 6.4 Hz, 3H); ^{13}C NMR (100 MHz, $CDCl_3$) δ 158.2, 149.8, 144.3, 133.2, 129.9, 128.4, 127.4, 126.3, 123.5, 103.9, 103.8, 70.2, 23.3; IR (film) 1072, 1003, 912, 791, 700 cm^{-1}; HRMS (ESI+) *m/z* calc'd for $(M + H)^+$ $[C_{15}H_{15}NO_2 + H]^+$: 242.1176, found 242.1176

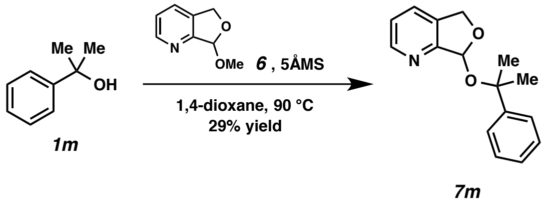

In a 2-dram vial with a PTFE-lined cap, a suspension of 2-phenyl-2-propanol (**1m**, 54.5 mg, 0.400 mmol), methyl acetal **6** (63.5 mg, 0.420 mmol), and 5Å molecular sieves (500 mg) in 1,4-dioxane (0.635 mL) was heated at 90 °C (without stirring) for 6.5 h. Upon completion, the reaction was cooled to ambient temperature and EtOAc (~5.0 mL) and celite (~500 mg) were added to the mixture. This suspension was filtered through a pad of celite (65 mm x 10 mm) with 9:1 EtOAc/MeOH (150 mL), concentrated by rotary evaporation, and purified by flash column chromatography (4:1 hexanes/EtOAc eluent) to afford acetal **7m** (29.6 mg, 29% yield, R_f = 0.22 in 4:1 hexanes/EtOAc) as a white solid.

Acetal 7m: ^{1}H NMR (400 MHz, $CDCl_3$) δ 8.61 (d, J = 4.8 Hz, 1H), 7.69 (d, J = 7.6 Hz, 2H), 7.59 (d, J = 7.2 Hz, 1H), 7.41 (t, J = 7.6 Hz, 2H), 7.28 (t, J = 7.6 Hz, 1H), 7.24 (dd, J = 7.2, 4.8 Hz, 1H), 6.12 (s, 1H), 5.22 (d, J = 12.8 Hz, 1H), 4.94 (d, J = 12.8 Hz, 1H), 1.82 (s, 3H), 1.65 (s, 3H); ^{13}C NMR (100 MHz, $CDCl_3$) δ 158.7, 149.9, 146.4, 133.1, 129.7, 128.4, 127.2, 126.4, 123.2, 101.0, 79.2, 69.9, 31.6, 28.4; IR (film) 1368, 1065, 999, 911, 762 cm^{-1}; HRMS (ESI+) *m/z* calc'd for $(M + H)^+$ $[C_{16}H_{17}NO_2 + H]^+$: 256.1332, found 256.1334.

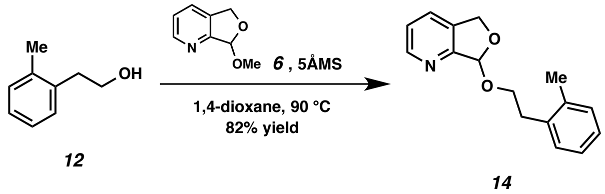

In a 2-dram vial with a PTFE-lined cap, a suspension of alcohol **12** (68.1 mg, 0.500 mmol), methyl acetal **6** (79.4 mg, 0.525 mmol), and 5Å molecular sieves (625 mg) in 1,4-dioxane (0.794 mL) was heated at 90 °C (without stirring) for 16 h. Upon completion, the reaction was cooled to ambient temperature, and EtOAc (~5.0 mL) and celite (~500 mg) were added to the mixture. This suspension was filtered through a pad of celite (65 mm x 10 mm) with 9:1 EtOAc/MeOH (150 mL), the filtrate concentrated by rotary evaporation, and the residue purified by flash column chromatography (13:7 hexanes/EtOAc eluent) to afford acetal **14** (105 mg, 82% yield, R_f = 0.28 in 13:7 hexanes/EtOAc) as a beige oil.

Acetal 14: ^{1}H NMR (400 MHz, $CDCl_3$) δ 8.60 (d, J = 4.8 Hz, 1H), 7.63 (d, J = 7.2 Hz, 1H), 7.28 (dd, J = 7.2, 4.8 Hz, 1H), 7.22-7.08 (comp. m, 4H), 6.19 (s, 1H), 5.22 (d, J = 12.8 Hz, 1H), 5.07 (d, J = 12.8 Hz, 1H), 4.03-3.84 (comp. m, 2H), 3.10-2.93 (comp. m, 2H), 2.33 (s, 3H); ^{13}C NMR (100 MHz, $CDCl_3$) δ 157.4, 149.9, 136.53, 136.47, 133.1, 130.2, 129.9, 129.4, 126.5, 126.1, 123.6, 105.0, 70.4, 68.4, 33.8, 19.5; IR (film) 1098, 1003, 914, 793, 746 cm^{-1}; HRMS (ESI+) *m/z* calc'd for $(M + H)^+$ $[C_{16}H_{17}NO_2 + H]^+$: 256.1332, found 256.1334.

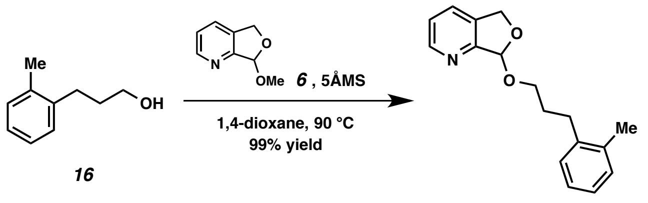

16 **18**

In a 2-dram vial with a PTFE-lined cap, a suspension of alcohol **16** (15.0 mg, 0.100 mmol), methyl acetal **6** (15.9 mg, 0.105 mmol), and 5Å molecular sieves (125 mg) in 1,4-dioxane (0.159 mL) was heated at 90 °C (without stirring) for 13 h. Upon completion, the reaction was cooled to ambient temperature, and EtOAc (~5.0 mL) and celite (~500 mg) were added to the mixture. This suspension was filtered through a pad of celite (65 mm x 10 mm) with 9:1 EtOAc/MeOH (150 mL), the filtrate concentrated by rotary evaporation, and the residue purified by flash column chromatography (4:1 hexanes/acetone eluent) to afford acetal **18** (26.6 mg, 99% yield, R_f = 0.14 in 4:1 hexanes/acetone) as a beige oil.

Acetal 18: ^{1}H NMR (400 MHz, $CDCl_3$) δ 8.60 (d, *J* = 4.8 Hz, 1H), 7.64 (d, *J* = 7.2 Hz, 1H), 7.28 (dd, *J* = 7.2, 4.8 Hz, 1H), 7.19-7.06 (comp. m, 4H), 6.15 (s, 1H), 5.25 (d, *J* = 12.8 Hz, 1H) 5.08 (d, *J* = 12.8 Hz, 1H), 3.93-3.83 (comp. m, 1H), 3.81-3.73 (comp. m, 1H), 2.70 (t, *J* = 7.6 Hz, 2H), 2.30 (s, 3H), 1.96 (app. quint, *J* = 7.6 Hz, 2H); ^{13}C NMR (100 MHz, $CDCl_3$) δ 157.6, 149.9, 140.1, 136.1, 133.1, 130.2, 129.9, 129.0, 126.00, 125.97, 123.6, 105.2, 70.4, 68.4, 30.2, 29.7, 19.4; IR (film) 1370, 1087, 1008, 793, 746 cm^{-1}; HRMS (ESI+) *m/z* calc'd for $(M + H)^+$ $[C_{17}H_{19}NO_2 + H]^+$: 270.1489, found 270.1490.

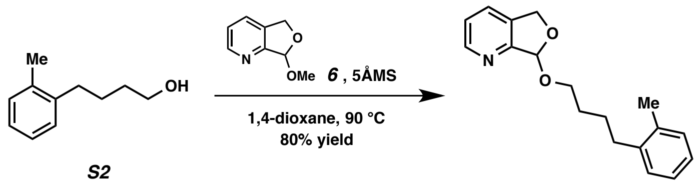

S2 **20**

In a 2-dram vial with a PTFE-lined cap, a suspension of alcohol **S2**[1] (82.1 mg, 0.500 mmol), methyl acetal **6** (79.4 mg, 0.525 mmol), and 5Å molecular sieves (625 mg) in 1,4-dioxane (0.794 mL) was heated at 90 °C and stirred for 13 h. Upon completion, the reaction was cooled to ambient temperature, and EtOAc (~5.0 mL) and celite (~500 mg) were added to the mixture. This suspension was filtered through a pad of celite (65 mm x 10 mm) with 9:1 EtOAc/MeOH (150 mL), the filtrate concentrated by rotary evaporation, and the residue purified by flash column chromatography (7:3 hexanes/EtOAc eluent) to afford acetal **20** (113 mg, 80% yield, R_f = 0.33 in 7:3 hexanes/EtOAc) as a colorless oil.

Acetal 20: ^{1}H NMR (500 MHz, $CDCl_3$) δ 8.58 (d, *J* = 5.0 Hz, 1H), 7.63 (d, *J* = 7.8 Hz, 1H), 7.27 (dd, *J* = 7.8, 5.0 Hz, 1H), 7.13-7.05 (comp. m, 4H), 6.12 (d, *J* = 2.0 Hz, 1H), 5.22 (dd, *J* = 13.3, 2.0 Hz, 1H), 5.06 (d, *J* = 13.2 Hz, 1H), 3.87 (dt, *J* = 9.5, 6.5 Hz, 1H), 3.76 (dt, *J* = 9.5, 6.5 Hz, 1H), 2.62 (dt, *J* = 8.3, 1.5 Hz, 2H), 2.29 (s, 3H), 1.80-1.72 (comp. m, 2H), 1.70-1.62 (comp. m, 2H); ^{13}C NMR (125 MHz, $CDCl_3$) δ 157.7, 150.0, 140.8, 136.1, 133.2, 130.3, 130.0, 129.1, 126.02, 125.99, 123.7, 105.1, 70.5, 69.0, 33.2, 29.9, 26.9, 19.5; IR (film) 1092, 1004, 909, 786, 744 cm^{-1}; HRMS (ESI+) *m/z* calc'd for $(M + H)^+$ $[C_{18}H_{21}NO_2 + H]^+$: 284.1648, found 284.1648.

Olefination of Benzylic Alcohol Acetals

General Procedure

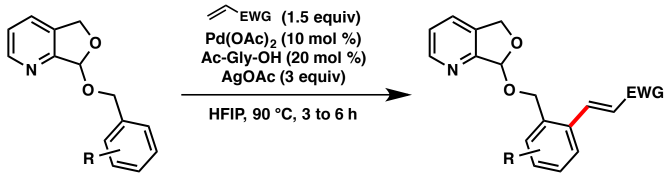

A suspension of benzyl acetal, olefin (1.5 equiv), $Pd(OAc)_2$ (10 mol %), *N*-acetylglycine (20 mol %), and AgOAc (3 equiv) in hexafluoroisopropanol (50.0 mM) in a 2-dram vial with a PTFE-lined cap was heated at 90 °C and stirred for 3 to 6 h. Upon completion, the reaction was cooled to ambient temperature, and then ethylenediamine (~150 wt. % relative to acetal) and celite (~300 wt. % relative to acetal) were added, and the resulting mixture was stirred overnight. The mixture was filtered through a plug of SiO_2, the filtrate was concentrated by rotary evaporation, and the resulting residue was dissolved in EtOAc and then filtered through a second plug of SiO_2. The filtrate was concentrated in vacuo, and the residue was purified by flash column chromatography to afford the olefinated product.

Scope of Benzylic Alcohol Acetal Functionalization

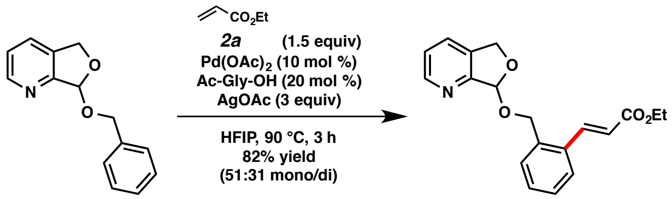

7a *8aa*

A suspension of acetal **7a** (22.7 mg, 0.100 mmol), ethyl acrylate (**2a**, 16.0 µL, 0.150 mmol), $Pd(OAc)_2$ (2.2 mg, 10.0 µmol), *N*-acetylglycine (2.3 mg, 20.0 µmol), and AgOAc (50.1 mg, 0.300 mmol) in hexafluoroisopropanol (2.00 mL) in a 2-dram vial with a PTFE-lined cap was heated at 90 °C and stirred for 3 h. Upon completion, the reaction was cooled to ambient temperature, ethylenediamine (40 µL) and celite (70 mg) were added, and the resulting mixture was stirred overnight. The mixture was filtered through a plug of SiO_2 (5 mm x 20 mm, EtOAc eluent, ~20 mL), the filtrate was concentrated by rotary evaporation, and the resulting residue was dissolved in EtOAc and then filtered through a second plug of SiO_2 (5 mm x 20 mm, EtOAc eluent, ~20 mL). The filtrate was concentrated by rotary evaporation, and the crude residue was purified by flash column chromatography (3:2 hexanes/EtOAc eluent) to afford olefin **8aa** (29.7 mg, 82% yield, 1:0.61 mono/di, R_f = 0.21 in 3:2 hexanes/EtOAc) as a colorless oil.

Olefin 8aa: ^{1}H NMR (400 MHz, $CDCl_3$) δ 8.56 (d, *J* = 4.8 Hz, 1H), 8.55 (d, *J* = 4.8 Hz, 0.61H), 8.19 (d, *J* = 15.8 Hz, 1.22H), 8.10 (d, *J* = 15.8 Hz, 1H), 7.64 (app. d, *J* = 7.6 Hz, 1.61H), 7.57 (app. t, *J* = 8.6 Hz, 2H), 7.48 (d, *J* = 7.2 Hz, 1H), 7.37-7.22 (comp. m, 4.44H), 6.38 (d, *J* = 15.8 Hz, 1H), 6.36 (d, *J* = 15.8 Hz, 1.22H), 6.29 (s, 0.61H), 6.26 (s, 1H), 5.53 (d, *J* = 13.6 Hz, 0.61H), 5.42 (d, *J* = 13.2 Hz, 1H), 5.20-5.05 (comp. m, 3.22H), 4.91 (d, *J* = 10.8 Hz, 0.61H), 4.78 (d, *J* = 11.2 Hz, 1H), 4.28 (q, *J* = 7.2 Hz, 2.44H), 4.26 (q, *J* = 7.2 Hz, 2H), 1.35 (t, *J* = 7.2 Hz, 3.66H), 1.33 (t, *J* = 7.2 Hz, 3H); ^{13}C NMR

(100 MHz, $CDCl_3$) δ 166.9, 166.6, 157.3, 157.1, 149.73, 149.71, 142.0, 141.7, 136.3, 136.2, 134.3, 133.8, 133.32, 133.26, 130.2, 129.9, 129.81, 129.78, 128.9, 128.42, 128.36, 126.6, 123.5, 121.9, 120.2, 104.3, 104.2, 70.7, 70.5, 67.9, 62.6, 60.6, 60.4, 14.3; IR (film) 1710, 1637, 1312, 1165, 1003 cm^{-1}; HRMS (ESI+) *m/z* calc'd for $(M_{mono} + H)^+$ $[C_{19}H_{19}NO_4 + H]^+$: 326.1387, found 326.1390; HRMS (ESI+) *m/z* calc'd for $(M_{di} + H)^+$ $[C_{24}H_{25}NO_6 + H]^+$: 424.1755, found 424.1756.

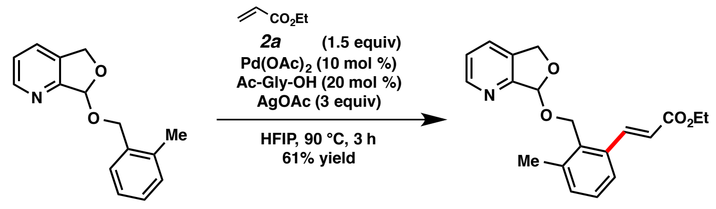

7b **8ba**

A suspension of acetal **7b** (121 mg, 0.500 mmol), ethyl acrylate (**2a**, 79.8 µL, 0.750 mmol), $Pd(OAc)_2$ (11.2 mg, 50.0 µmol), *N*-acetylglycine (11.7 mg, 0.100 mmol), and AgOAc (250 mg, 1.50 mmol) in hexafluoroisopropanol (10.0 mL) in a 2-dram vial with a PTFE-lined cap was heated at 90 °C and stirred for 3 h. Upon completion, the reaction was cooled to ambient temperature, ethylenediamine (40 µL) and celite (70 mg) were added, and the resulting mixture was stirred overnight. The mixture was filtered through a plug of SiO_2 (5 mm x 20 mm, EtOAc eluent, ~20 mL), the filtrate was concentrated by rotary evaporation, and the resulting residue was dissolved in EtOAc and then filtered through a second plug of SiO_2 (5 mm x 20 mm, EtOAc eluent, ~20 mL). The filtrate was concentrated by rotary evaporation and the crude residue was purified by flash column chromatography (3:2 hexanes/EtOAc eluent) to afford olefin **8ba** (104 mg, 61% yield, R_f = 0.27 in 3:2 hexanes/EtOAc) as a beige oil.

Olefin 8ba: ^{1}H NMR (400 MHz, $CDCl_3$) δ 8.56 (dd, *J* = 4.8, 1.6 Hz, 1H), 8.21 (d, *J* = 16.0 Hz, 1H), 7.63 (dd, *J* = 8.0, 1.6 Hz, 1H), 7.39 (dd, *J* = 5.6, 4.2 Hz, 1H), 7.25 (dd, *J* = 7.6, 4.8 Hz, 1H), 7.20 (d, *J* = 5.6 Hz, 1H), 7.20 (d, *J* = 4.2 Hz, 1H), 6.35 (d, *J* = 16.0 Hz, 1H), 6.24 (d, *J* = 2.0 Hz, 1H), 5.42 (dd, *J* = 13.6, 2.0 Hz, 1H), 5.15 (d, *J* = 13.6 Hz, 1H), 5.04 (d, *J* = 10.4 Hz, 1H), 4.86 (d, *J* = 10.4 Hz, 1H), 4.28 (q, *J* = 7.2 Hz, 2H), 2.49 (s, 3H), 1.35 (t, *J* = 7.2 Hz, 3H); ^{13}C NMR (100 MHz, $CDCl_3$) δ 167.1, 157.6, 149.9, 143.1, 139.1, 135.6, 134.2, 133.4, 132.2, 129.9, 128.7, 124.9, 123.6, 120.9, 104.4, 70.7, 63.6, 60.5, 19.8, 14.5; IR (film) 1707, 1312, 1178, 1165, 1001 cm^{-1}; HRMS (ESI+) *m/z* calc'd for $(M + H)^+$ $[C_{20}H_{21}NO_4 + H]^+$: 340.1543, found 340.1539.

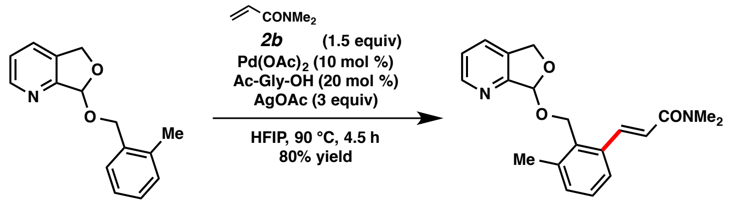

7b **8bb**

A suspension of acetal **7b** (24.1 mg, 0.100 mmol), *N,N*-dimethylacrylamide (**2b**, 15.5 µL, 0.150 mmol), $Pd(OAc)_2$ (2.2 mg, 10.0 µmol), *N*-acetylglycine (2.3 mg, 20.0 µmol), and AgOAc (50.1 mg, 0.300 mmol) in hexafluoroisopropanol (2.00 mL) in a 2-dram vial with a PTFE-lined cap was heated at 90 °C and stirred for 4.5 h. Upon completion, the reaction was cooled to ambient temperature, ethylenediamine (40 µL) and celite (70 mg) were added, and the resulting mixture was stirred overnight. The mixture was filtered through a plug of SiO_2 (5 mm x 20 mm, EtOAc → 9:1 EtOAc/MeOH eluent, ~20 mL), the filtrate was concentrated by rotary evaporation, and the resulting

residue was dissolved in EtOAc and then filtered through a second plug of SiO_2 (5 mm x 20 mm, EtOAc → 9:1 EtOAc/MeOH eluent, ~20 mL). The filtrate was concentrated by rotary evaporation, and the crude residue was purified by flash column chromatography (9:1 EtOAc/MeOH eluent) to afford olefin **8bb** (27.1 mg, 80% yield, R_f = 0.26 in 9:1 EtOAc/MeOH) as a white solid.

Olefin 8bb: ^{1}H NMR (400 MHz, $CDCl_3$) δ 8.53 (d, *J* = 4.8 Hz, 1H), 8.09 (d, *J* = 15.4 Hz, 1H), 7.62 (d, *J* = 7.6 Hz, 1H), 7.34 (app. d, *J* = 6.8 Hz, 1H), 7.23 (dd, *J* = 7.6, 4.8 Hz, 1H), 7.18 (d, *J* = 6.8 Hz, 1H), 7.17 (app. s, 1H), 6.80 (d, *J* = 15.4 Hz, 1H), 6.23 (s, 1H), 5.44 (d, *J* = 13.2 Hz, 1H), 5.15 (d, *J* = 13.2 Hz, 1H), 5.03 (d, *J* = 10.2 Hz, 1H), 4.83 (d, *J* = 10.2 Hz, 1H), 3.16 (s, 3H), 3.08 (s, 3H), 2.49 (s, 3H); ^{13}C NMR (100 MHz, $CDCl_3$) δ 166.9, 157.6, 149.7, 140.7, 139.2, 137.0, 133.8, 133.5, 131.4, 129.9, 128.5, 125.2, 123.6, 121.0, 104.3, 70.7, 63.8, 37.7, 36.1, 19.8; IR (film) 1651, 1610, 1387, 1069, 1003 cm^{-1}; HRMS (ESI+) *m/z* calc'd for $(M + H)^+$ $[C_{20}H_{22}N_2O_3 + H]^+$: 339.1703, found 339.1706.

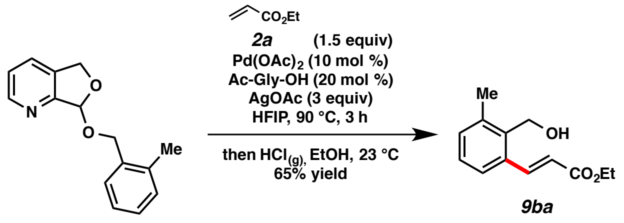

7b *9ba*

A suspension of acetal **7b** (24.1 mg, 0.100 mmol), ethyl acrylate (**2a**, 16.0 µL, 0.150 mmol), $Pd(OAc)_2$ (2.2 mg, 10.0 µmol), *N*-acetylglycine (2.3 mg, 20.0 µmol), and AgOAc (50.1 mg, 0.300 mmol) in hexafluoroisopropanol (2.00 mL) in a 2-dram vial with a PTFE-lined cap was heated at 90 °C and stirred for 3 h. Upon completion, the reaction was cooled to ambient temperature, ethylenediamine (40 µL) and celite (70 mg) were added, and the resulting mixture was stirred overnight. The mixture was filtered through a plug of SiO_2 (5 mm x 20 mm, EtOAc eluent, ~20 mL), the filtrate was concentrated by rotary evaporation, and the resulting residue was dissolved in EtOAc and then filtered through a second plug of SiO_2 (5 mm x 20 mm, EtOAc eluent, ~20 mL). The solution was concentrated by rotary evaporation, EtOH (1.00 mL) was added to the residue, and anhydrous HCl was bubbled through the solution for 1 min. The solution was stirred at 23 °C for 25 h. Sat. aq. Na_2CO_3 (5 mL) was added to the reaction mixture, and it was extracted with EtOAc (3 x 10 mL). The combined organic layers were washed with brine (5 mL) and dried with $MgSO_4$. The solution was concentrated by rotary evaporation, and the crude residue was purified by flash column chromatography (7:3 hexanes/EtOAc eluent) to afford alcohol **9ba** (14.2 mg, 65% yield, R_f = 0.33 in 7:3 hexanes/EtOAc) as a colorless oil. Additionally, 2-methylbenzyl alcohol (**1b**, 2.1 mg, 17% yield) was isolated.

Alcohol 9ba: ^{1}H NMR (400 MHz, $CDCl_3$) δ 8.14 (d, *J* = 15.6 Hz, 1H), 7.43 (t, *J* = 4.8 Hz, 1H), 7.23 (app. d, *J* = 4.8 Hz, 2H), 6.37 (d, *J* = 15.6 Hz, 1H), 4.83 (s, 2H), 4.27 (q, *J* = 7.2 Hz, 2H), 2.47 (s, 3H), 1.66 (br. s, 1H), 1.34 (t, *J* = 7.2 Hz, 3H); ^{13}C NMR (100 MHz, $CDCl_3$) δ 167.0, 142.6, 138.2, 137.2, 134.8, 132.4, 128.6, 125.2, 121.2, 60.7, 58.7, 19.6, 14.5; IR (film) 3425, 2925, 1709, 1632, 1312, 1180, 1163, 1005 cm^{-1}; HRMS (ESI+) *m/z* calc'd for $(M + H)^+$ $[C_{13}H_{16}O_3 + H]^+$: 221.1172, found 221.1175.

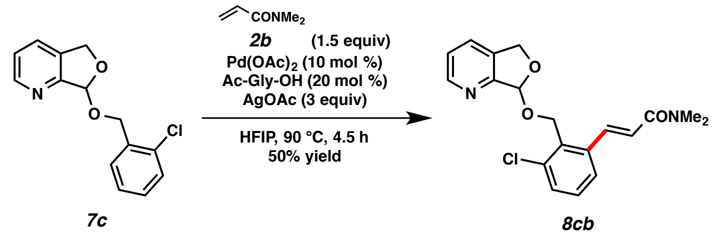

A suspension of acetal **7c** (25.7 mg, 0.100 mmol), *N,N*-dimethylacrylamide (**2b**, 15.5 µL, 0.150 mmol), $Pd(OAc)_2$ (2.2 mg, 10.0 µmol), *N*-acetylglycine (2.3 mg, 20.0 µmol), and AgOAc (50.1 mg, 0.300 mmol) in hexafluoroisopropanol (2.00 mL) in a 2-dram vial with a PTFE-lined cap was heated at 90 °C and stirred for 4.5 h. Upon completion, the reaction was cooled to ambient temperature, ethylenediamine (40 µL) and celite (70 mg) were added, and the resulting mixture was stirred overnight. The mixture was filtered through a plug of SiO_2 (5 mm x 20 mm, EtOAc → 9:1 EtOAc/MeOH eluent, ~20 mL), the filtrate was concentrated by rotary evaporation, and the resulting residue was dissolved in EtOAc and then filtered through a second plug of SiO_2 (5 mm x 20 mm, EtOAc → 9:1 EtOAc/MeOH eluent, ~20 mL). The filtrate was concentrated by rotary evaporation, and the crude residue was purified by flash column chromatography (EtOAc eluent) to afford olefin **8cb** (27.5 mg, 50% yield, R_f = 0.32 in EtOAc) as a white solid.
Olefin 8cb: ^1H NMR (500 MHz, $CDCl_3$) δ 8.52 (d, *J* = 4.5 Hz, 1H), 8.01 (d, *J* = 15.1 Hz, 1H), 7.62 (d, *J* = 7.5 Hz, 1H), 7.39 (d, *J* = 7.5 Hz, 1H), 7.36 (d, *J* = 8.0 Hz, 1H), 7.25-7.19 (comp. m, 2H), 6.81 (d, *J* = 15.1 Hz, 1H), 6.27 (d, *J* = 1.5 Hz, 1H), 5.45 (dd, *J* = 13.5, 1.5 Hz, 1H), 5.13 (d, *J* = 13.5 Hz, 1H), 5.11 (d, *J* = 10.5 Hz, 1H), 5.02 (d, *J* = 10.5 Hz, 1H), 3.13 (s, 3H), 3.06 (s, 3H); ^{13}C NMR (125 MHz, $CDCl_3$) δ 166.5, 157.6, 149.8, 139.6, 139.1, 133.6, 133.4, 130.30, 129.99, 129.6, 125.9, 123.7, 122.2, 104.5, 70.8, 64.0, 37.7, 36.1; IR (film) 1651, 1609, 1065, 998, 788 cm^{-1}; HRMS (ESI+) *m/z* calc'd for $(M + H)^+$ $[C_{19}H_{19}ClN_2O_3 + H]^+$: 359.1157, found 359.1158.

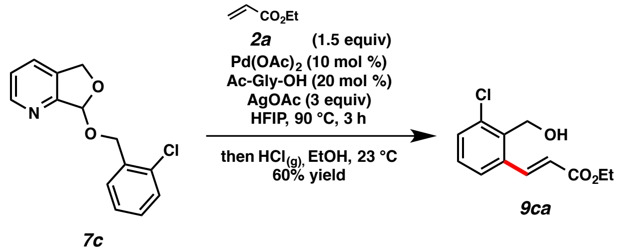

A suspension of acetal **7c** (26.2 mg, 0.100 mmol), ethyl acrylate (**2a**, 16.0 µL, 0.150 mmol), $Pd(OAc)_2$ (2.2 mg, 10.0 µmol), *N*-acetylglycine (2.3 mg, 20.0 µmol), and AgOAc (50.1 mg, 0.300 mmol) in hexafluoroisopropanol (2.00 mL) in a 2-dram vial with a PTFE-lined cap was heated at 90 °C and stirred for 3 h. Upon completion, the reaction was cooled to ambient temperature, ethylenediamine (40 µL) and celite (70 mg) were added, and the resulting mixture was stirred overnight. The mixture was filtered through a plug of SiO_2 (5 mm x 20 mm, EtOAc eluent, ~20 mL), the filtrate was concentrated by rotary evaporation, and the resulting residue was dissolved in EtOAc and then filtered through a second plug of SiO_2 (5 mm x 20 mm, EtOAc eluent, ~20 mL). The solution was concentrated by rotary evaporation, EtOH (1.00 mL) was added to the residue, and anhydrous HCl was bubbled through the solution for 1 min. The solution was stirred at 23 °C for 25 h. Sat. aq. Na_2CO_3 (5 mL) was added to the reaction mixture, and it was extracted with EtOAc (3 x 10 mL). The combined organic layers were washed with brine (5 mL) and dried with $MgSO_4$. The solution was concentrated by rotary evaporation,

and the crude residue was purified by flash column chromatography (4:1 hexanes/EtOAc eluent) to afford alcohol **10ca** (18.0 mg, 60% yield, R_f = 0.31 in 4:1 hexanes/EtOAc) as a colorless oil. Additionally, 2-chlorobenzyl alcohol (**1c**, 6.3 mg, 36% yield) was isolated.

Alcohol 10ca: ^{1}H NMR (500 MHz, $CDCl_3$) δ 8.09 (d, *J* = 15.6 Hz, 1H), 7.48 (d, *J* = 7.6 Hz, 1H), 7.43 (d, *J* = 8.0 Hz, 1H), 7.28 (d, *J* = 8.0 Hz, 1H), 6.40 (d, *J* = 16.0 Hz, 1H), 4.94 (s, 2H), 4.28 (q, *J* = 6.8 Hz, 2H), 1.96 (br. s, 1H), 1.34 (t, *J* = 6.8 Hz, 3H); ^{13}C NMR (125 MHz, $CDCl_3$) δ 166.7, 141.5, 137.0, 136.5, 135.8, 131.1, 129.7, 126.2, 122.6, 61.0, 59.1, 14.5; IR (film) 3460, 1701, 1313, 1176, 1024 cm^{-1}; HRMS (ESI+) *m/z* calc'd for $(M + Na)^+$ $[C_{12}H_{13}ClO_3 + Na]^+$: 263.0445, found 263.0447.

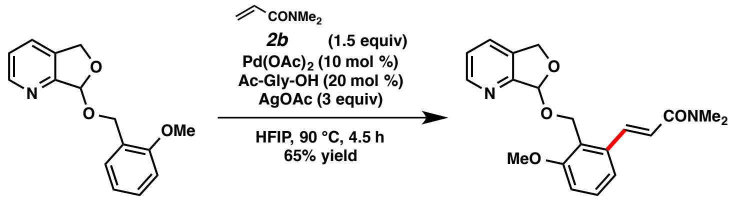

7d *8db*

A suspension of acetal **7d** (24.2 mg, 0.100 mmol), *N,N*-dimethylacrylamide (**2b**, 15.5 µL, 0.150 mmol), $Pd(OAc)_2$ (2.2 mg, 10.0 µmol), *N*-acetylglycine (2.3 mg, 20.0 µmol), and AgOAc (50.1 mg, 0.300 mmol) in hexafluoroisopropanol (2.00 mL) in a 2-dram vial with a PTFE-lined cap was heated at 90 °C and stirred for 4.5 h. Upon completion, the reaction was cooled to ambient temperature, ethylenediamine (40 µL) and celite (70 mg) were added, and the resulting mixture was stirred overnight. The mixture was filtered through a plug of SiO_2 (5 mm x 20 mm, EtOAc → 9:1 EtOAc/MeOH eluent, ~20 mL), the filtrate was concentrated by rotary evaporation, and the resulting residue was dissolved in EtOAc and then filtered through a second plug of SiO_2 (5 mm x 20 mm, EtOAc → 9:1 EtOAc/MeOH eluent, ~20 mL). The filtrate was concentrated by rotary evaporation, and the crude residue was purified by flash column chromatography (EtOAc eluent) to afford olefin **8db** (23.0 mg, 65% yield, R_f = 0.29 in EtOAc) as a white solid.

Olefin 8db: ^{1}H NMR (400 MHz, $CDCl_3$) δ 8.50 (d, *J* = 5.0 Hz, 1H), 8.04 (d, *J* = 15.4 Hz, 1H), 7.61 (d, *J* = 7.6 Hz, 1H), 7.24 (dd, *J* = 8.4, 7.6 Hz, 1H), 7.21 (dd, *J* = 7.6, 5.0 Hz, 1H), 7.23 (d, *J* = 7.6 Hz, 1H), 6.86 (d, *J* = 8.4 Hz, 1H), 6.82 (d, *J* = 15.4 Hz, 1H), 6.26 (s, 1H), 5.48 (d, *J* = 13.4 Hz, 1H), 5.13 (d, *J* = 13.4 Hz, 1H), 5.01 (ABq, *J* = 10.0 Hz, Δν = 18.6 Hz, 2H), 3.84 (s, 3H), 3.12 (s, 3H), 3.05 (s, 3H); ^{13}C NMR (100 MHz, $CDCl_3$) δ 166.9, 158.6, 157.8, 149.6, 140.1, 138.1, 133.7, 129.9, 129.4, 124.5, 123.4, 120.9, 119.2, 111.5, 104.4, 70.6, 60.5, 56.0, 37.6, 36.1; IR (film) 1646, 1392, 1252, 995, 788 cm^{-1}; HRMS (ESI+) *m/z* calc'd for $(M + H)^+$ $[C_{20}H_{22}N_2O_4 + H]^+$: 355.1652, found 355.1652.

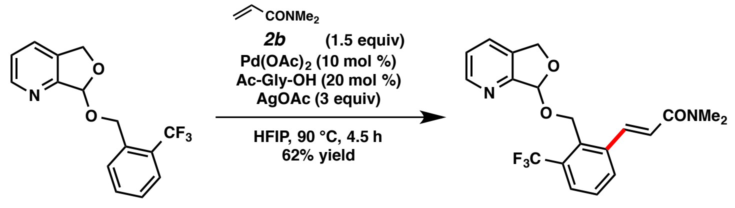

7e *8eb*

A suspension of acetal **7e** (19.7 mg, 66.7 µmol), *N,N*-dimethylacrylamide (**2b**, 10.3 µL, 0.100 mmol), $Pd(OAc)_2$ (1.5 mg, 6.67 µmol), *N*-acetylglycine (1.6 mg, 13.3 µmol), and AgOAc (33.6 mg, 0.200 mmol) in hexafluoroisopropanol (1.33 mL) in a 2-dram vial with a PTFE-lined cap was heated 90 °C and stirred at for 4.5 h. Upon completion, the reaction was cooled to ambient temperature,

ethylenediamine (40 µL) and celite (70 mg) were added, and the resulting mixture was stirred overnight. The mixture was filtered through a plug of SiO_2 (5 mm x 20 mm, EtOAc → 9:1 EtOAc/MeOH eluent, ~20 mL), the filtrate was concentrated by rotary evaporation, and the resulting residue was dissolved in EtOAc and then filtered through a second plug of SiO_2 (5 mm x 20 mm, EtOAc → 9:1 EtOAc/MeOH eluent, ~20 mL). The filtrate was concentrated by rotary evaporation, and the crude residue was purified by flash column chromatography (EtOAc eluent) to afford olefin **8eb** (16.3 mg, 62% yield, R_f = 0.29 in EtOAc) as a white solid.

Olefin 8eb: ^{1}H NMR (400 MHz, $CDCl_3$) δ 8.52 (d, J = 5.0 Hz, 1H), 8.09 (d, J = 15.4 Hz, 1H), 7.68 (d, J = 8.0 Hz, 1H), 7.64 (d, J = 7.4 Hz, 1H), 7.62 (d, J = 8.0 Hz, 1H), 7.39 (t, J = 8.0 Hz, 1H), 7.23 (dd, J = 7.4, 5.0 Hz, 1H), 6.81 (d, J = 15.4 Hz, 1H), 6.25 (s, 1H), 5.52 (d, J = 13.6 Hz, 1H), 5.15 (d, J = 13.6 Hz, 1H), 5.14 (d, J = 10.8 Hz, 1H), 4.90 (d, J = 10.8 Hz, 1H), 3.14 (s, 3H), 3.08 (s, 3H); ^{13}C NMR (100 MHz, $CDCl_3$) δ 166.4, 157.4, 149.8, 139.3 (q, $^5J_{C-F}$ = 2.1 Hz), 133.9, 133.6, 130.7 (q, $^2J_{C-F}$ = 29.8 Hz), 129.9, 128.6, 126.9 (q, $^1J_{C-F}$ = 272.7 Hz), 126.5 (q, $^3J_{C-F}$ = 5.8 Hz), 123.6, 122.3, 104.5, 70.8, 63.0 (q, $^4J_{C-F}$ = 2.1 Hz), 37.7, 36.1; IR (film) 1653, 1109, 1062, 1000, 805 cm^{-1}; HRMS (ESI+) *m/z* calc'd for $(M + H)^+$ $[C_{20}H_{19}F_3N_2O_3 + H]^+$: 393.1421, found 393.1421.

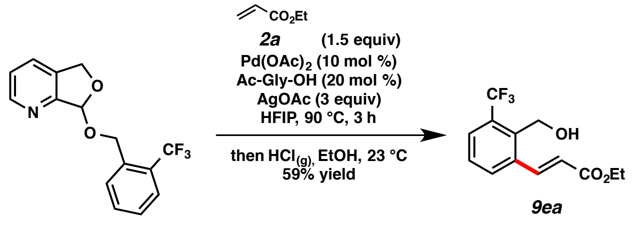

7d 9ea

A suspension of acetal **7e** (29.5 mg, 0.100 mmol), ethyl acrylate (**2a**, 16.0 µL, 0.150 mmol), $Pd(OAc)_2$ (2.2 mg, 10.0 µmol), *N*-acetylglycine (2.3 mg, 20.0 µmol), and AgOAc (50.1 mg, 0.300 mmol) in hexafluoroisopropanol (2.00 mL) in a 2-dram vial with a PTFE-lined cap was heated at 90 °C and stirred for 3 h. Upon completion, the reaction was cooled to ambient temperature, ethylenediamine (40 µL) and celite (70 mg) were added, and the resulting mixture was stirred overnight. The mixture was filtered through a plug of SiO_2 (5 mm x 20 mm, EtOAc eluent, ~20 mL), the filtrate was concentrated by rotary evaporation, and the resulting residue was dissolved in EtOAc and then filtered through a second plug of SiO_2 (5 mm x 20 mm, EtOAc eluent, ~20 mL). The solution was concentrated by rotary evaporation, EtOH (1.00 mL) was added to the residue, and anhydrous HCl was bubbled through the solution for 1 min. The solution was stirred at 23 °C for 25 h. Sat aq. Na_2CO_3 (5 mL) was added, and the mixture was extracted with EtOAc (3 x 10 mL). The combined organic layers were washed with brine (5 mL) and dried with $MgSO_4$. The solution was concentrated by rotary evaporation, and the crude residue was purified by flash column chromatography (1:1 hexanes/EtOAc eluent) to afford alcohol **9ea** (11.0 mg, 40% yield, R_f = 0.33 in 1:1 hexanes/EtOAc) as a white solid. An additional portion of the olefinated alcohol (5.2 mg, 19% yield) was isolated as the isobenzofuran. Additionally, 2-(trifluoromethyl)benzyl alcohol (**1e**, 4.3 mg, 24% recovered alcohol) was isolated.

Alcohol 9ea: ^{1}H NMR (400 MHz, $CDCl_3$) δ 8.19 (d, J = 16.0 Hz, 1H), 7.78 (d, J = 8.0 Hz, 1H), 7.70 (d, J = 8.0 Hz, 1H), 7.46 (t, J = 8.0 Hz, 1H), 6.44 (d, J = 16.0 Hz, 1H), 4.90 (d, J = 3.2 Hz, 2H), 4.29 (q, J = 7.2 Hz, 2H), 1.86 (br. s, 1H), 1.35 (t, J = 7.2 Hz, 3H); ^{13}C NMR (125 MHz, $CDCl_3$) δ 166.5, 141.1, 137.5, 137.1, 131.1, 129.9 (q, $^2J_{C-F}$ = 30.0 Hz), 128.7, 127.2 (q, $^3J_{C-F}$ = 5.8 Hz), 124.3 (q, $^1J_{C-F}$ = 272.9 Hz), 122.8, 61.0, 58.1 (d, $^4J_{C-F}$ = 2.5 Hz), 14.5**;** IR (film) 1701, 1318, 1166, 1113, 736 cm^{-1}; HRMS (ESI+) *m/z* calc'd for $(M + Na)^+$ $[C_{13}H_{13}F_3O_3 + Na]^+$: 297.0709, found 297.0710.

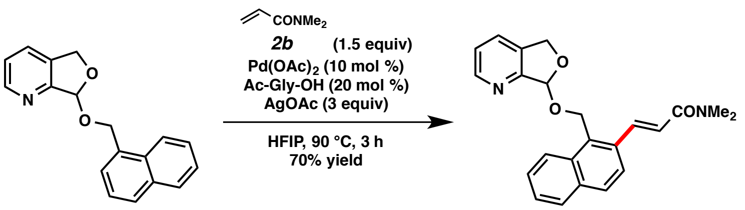

7f **8fb**

A suspension of acetal **7f** (24.1 mg, 0.100 mmol), *N,N*-dimethylacrylamide (**2b**, 15.5 µL, 0.150 mmol), $Pd(OAc)_2$ (2.2 mg, 10.0 µmol), *N*-acetylglycine (2.3 mg, 20.0 µmol), and AgOAc (50.1 mg, 0.300 mmol) in hexafluoroisopropanol (2.00 mL) in a 2-dram vial with a PTFE-lined cap was heated at 90 °C and stirred for 3 h. Upon completion, the reaction was cooled to ambient temperature, ethylenediamine (40 µL) and celite (70 mg) were added, and the resulting mixture was stirred overnight. The mixture was filtered through a plug of SiO_2 (5 mm x 20 mm, EtOAc → 9:1 EtOAc/MeOH eluent, ~20 mL), the filtrate was concentrated by rotary evaporation, and the resulting residue was dissolved in EtOAc and then filtered through a second plug of SiO_2 (5 mm x 20 mm, EtOAc → 9:1 EtOAc/MeOH eluent, ~20 mL). The filtrate was concentrated by rotary evaporation, and the crude residue was purified by flash column chromatography (EtOAc → 9:1 EtOAc/MeOH eluent) to afford olefin **8fb** (26.2 mg, 70% yield, R_f = 0.51 in 9:1 EtOAc/MeOH) as a white solid.

Olefin 8fb: ^{1}H NMR (400 MHz, $CDCl_3$) δ 8.53 (d, *J* = 4.6 Hz, 1H), 8.37 (d, *J* = 8.4 Hz, 1H), 8.29 (d, *J* = 15.2 Hz, 1H), 7.78 (app. d, *J* = 9.2 Hz, 2H), 7.64-7.55 (comp. m, 3H), 7.48 (t, *J* = 7.6 Hz, 1H), 7.23 (dd, *J* = 7.6, 4.6 Hz, 1H), 6.96 (d, *J* = 15.2 Hz, 1H), 6.38 (s, 1H), 5.46 (d, *J* = 13.6 Hz, 1H), 5.43 (d, *J* = 10.8 Hz, 1H), 5.31 (d, *J* = 10.8 Hz, 1H), 5.20 (d, *J* = 13.6 Hz, 1H), 3.22 (s, 3H), 3.11 (s, 3H); ^{13}C NMR (100 MHz, $CDCl_3$) δ 166.8, 157.5, 149.8, 140.3, 133.99, 133.96, 133.5, 133.2, 131.6, 130.0, 129.3, 128.4, 127.2, 126.5, 125.2, 124.4, 123.6, 121.8, 104.3, 70.9, 62.5, 37.7, 36.2; IR (film) 1642, 1594, 1505, 1263, 1005 cm^{-1}; HRMS (ESI+) *m/z* calc'd for $(M + H)^+$ $[C_{23}H_{22}N_2O_3 + H]^+$: 375.1703, found 375.1708.

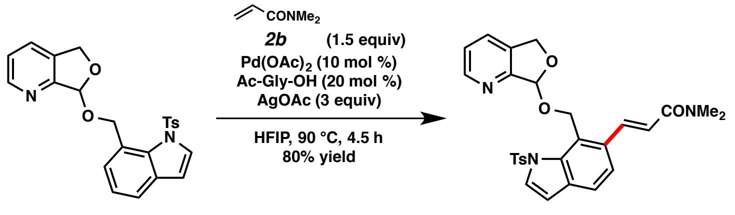

7g **8gb**

A suspension of acetal **7g** (42.0 mg, 0.100 mmol), *N,N*-dimethylacrylamide (**2b**, 15.5 µL, 0.150 mmol), $Pd(OAc)_2$ (2.2 mg, 10.0 µmol), *N*-acetylglycine (2.3 mg, 20.0 µmol), and AgOAc (50.1 mg, 0.300 mmol) in hexafluoroisopropanol (2.00 mL) in a 2-dram vial with a PTFE-lined cap was heated at 90 °C and stirred for 6 h. Upon completion, the reaction was cooled to ambient temperature, ethylenediamine (40 µL) and celite (70 mg) were added, and the resulting mixture was stirred overnight. The mixture was filtered through a plug of SiO_2 (5 mm x 20 mm, EtOAc → 9:1 EtOAc/MeOH eluent, ~20 mL), the filtrate was concentrated by rotary evaporation, and the resulting residue was dissolved in EtOAc and then filtered through a second plug of SiO_2 (5 mm x 20 mm, EtOAc → 9:1 EtOAc/MeOH eluent, ~20 mL). The filtrate was concentrated by rotary evaporation, and the crude residue was purified by flash

column chromatography (3:2 hexanes/EtOAc → EtOAc eluent) to afford olefin **8gb** (41.4 mg, 80% yield, R_f = 0.26 in EtOAc) as a white powder.

Olefin 8gb: ^{1}H NMR (400 MHz, $CDCl_3$) δ 8.53 (d, *J* = 4.8 Hz, 1H), 8.22 (d, *J* = 15.2 Hz, 1H), 7.77 (d, *J* = 8.2 Hz, 2H), 7.70 (d, *J* = 3.6 Hz, 1H), 7.65 (d, *J* = 7.4 Hz, 1H), 7.45 (d, *J* = 8.4 Hz, 1H), 7.33 (d, *J* = 8.4 Hz, 1H), 7.23 (dd, *J* = 7.4, 4.8 Hz, 1H), 7.07 (d, *J* = 8.2 Hz, 2H), 6.75 (d, *J* = 15.6 Hz, 1H), 6.61 (d, *J* = 3.6 Hz, 1H), 6.32 (s, 1H), 5.73 (d, *J* = 13.6 Hz, 1H), 5.62 (d, *J* = 10.6 Hz, 1H), 5.30 (d, *J* = 10.6 Hz, 1H), 5.19 (d, *J* =13.6 Hz, 1H), 3.12 (s, 3H), 3.06 (s, 3H), 2.29 (s, 3H); ^{13}C NMR (100 MHz, $CDCl_3$) δ 166.8, 158.1, 149.4, 144.7, 140.9, 136.7, 134.84, 134.81, 134.5, 134.0, 132.7, 129.9, 129.6, 127.5, 125.2, 123.3, 123.2, 121.4, 119.7, 111.3, 104.3, 70.8, 64.0, 37.7, 36.2, 21.7; IR (film) 1643, 1599, 1366, 1172, 1004 cm^{-1}; HRMS (ESI+) *m/z* calc'd for $(M + H)^+$ $[C_{28}H_{27}N_3O_5S+ H]^+$: 518.1744, found 518.1734.

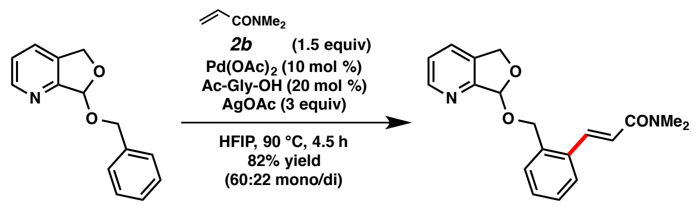

7a *8ab*

A suspension of acetal **7a** (22.7 mg, 0.100 mmol), *N,N*-dimethylacrylamide (**2b**, 15.5 µL, 0.150 mmol), $Pd(OAc)_2$ (2.2 mg, 10.0 µmol), *N*-acetylglycine (2.3 mg, 20.0 µmol), and AgOAc (50.1 mg, 0.300 mmol) in hexafluoroisopropanol (2.00 mL) in a 2-dram vial with a PTFE-lined cap was heated at 90 °C and stirred for 4.5 h. Upon completion, the reaction was cooled to ambient temperature, ethylenediamine (40 µL) and celite (70 mg) were added, and the resulting mixture was stirred overnight. The mixture was filtered through a plug of SiO_2 (5 mm x 20 mm, EtOAc → 1:1 EtOAc/MeOH eluent, ~20 mL), the filtrate was concentrated by rotary evaporation, and the resulting residue was dissolved in EtOAc and then filtered through a second plug of SiO_2 (5 mm x 20 mm, EtOAc → 1:1 EtOAc/MeOH eluent, ~20 mL). The filtrate was concentrated by rotary evaporation, and the crude residue was purified by flash column chromatography (19:1 EtOAc/hexanes eluent) to afford monoolefin **8ab$_{mono}$** (19.6 mg, 60% yield, R_f = 0.31 in 19:1 EtOAc/hexanes) as a white solid. The column was washed with MeOH, the filtrate concentrated by rotary evaporation, and the residue was purified by preparatory plate chromatography (9:1 EtOAc/MeOH eluent) to afford diolefin **8ab$_{di}$** (9.3 mg, 22% yield, R_f = 0.22 in EtOAc/MeOH) as a white solid.

Monoolefin 8ab$_{mono}$: ^{1}H NMR (400 MHz, $CDCl_3$) δ 8.55 (d, *J* = 4.8 Hz, 1H), 8.00 (d, *J* = 15.2 Hz, 1H), 7.64 (d, *J* = 8.0 Hz, 1H), 7.54 (d, *J* = 7.2 Hz, 1H), 7.50 (d, *J* = 7.2 Hz, 1H), 7.35-7.23 (comp. m, 3H), 6.83 (d, *J* = 15.2 Hz, 1H), 6.25 (s, 1H), 5.45 (d, *J* = 13.2 Hz, 1H), 5.14 (d, *J* = 13.2 Hz, 1H), 5.10 (d, *J* = 11.2 Hz, 1H), 4.80 (d, *J* = 11.2 Hz, 1H), 3.16 (s, 3H), 3.07 (s, 3H); ^{13}C NMR (100 MHz, $CDCl_3$) δ 168.8, 157.6, 149.8, 139.5, 136.2, 135.1, 133.6, 130.2, 130.0, 129.4, 128.3, 126.8, 123.6, 120.2, 104.3, 70.7, 67.8, 37.7, 36.1; IR (film) 1768, 1652, 1605, 1389, 1005 cm^{-1}; HRMS (ESI+) *m/z* calc'd for $(M + H)^+$ $[C_{19}H_{20}N_2O_3 + H]^+$: 325.1547, found 325.1554.

Diolefin 8ab$_{di}$: ^{1}H NMR (400 MHz, $CDCl_3$) δ 8.49 (d, *J* = 4.8 Hz, 1H), 8.08 (d, *J* = 15.2 Hz, 2H), 7.62 (d, *J* = 7.6 Hz, 1H), 7.48 (d, *J* = 7.6 Hz, 2H), 7.30 (t, *J* = 7.6 Hz, 1H), 7.22 (dd, *J* = 7.6, 4.8 Hz, 1H), 6.83 (d, *J* = 15.6 Hz, 2H), 6.27 (s, 1H), 5.57 (d, *J* = 13.6 Hz, 1H), 5.18 (d, *J* = 13.6 Hz, 1H), 5.08 (d, *J* = 10.4 Hz, 1H), 4.86 (d, *J* = 10.4 Hz, 1H), 3.18 (s, 6H), 3.08 (s, 6H); ^{13}C NMR (100 MHz, $CDCl_3$) δ 166.6, 157.4, 149.6, 140.1, 137.8, 133.71, 133.69, 130.0, 128.8, 128.1, 123.6, 121.9, 104.1, 70.9, 63.3,

37.7, 36.1; IR (film) 1645, 1600, 1391, 995, 727 cm^{-1}; HRMS (ESI+) *m/z* calc'd for $(M + H)^+$ $[C_{24}H_{27}N_3O_4 + H]^+$: 422.2074, found 422.2077.

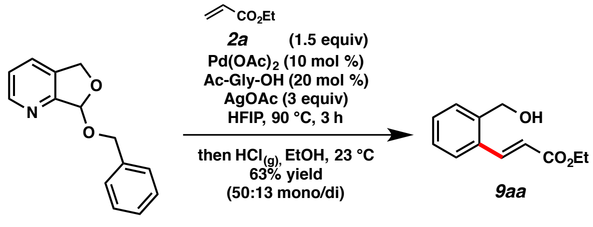

7a *9aa*

A suspension of acetal **7a** (22.7 mg, 0.100 mmol), ethyl acrylate (**2a**, 16.0 µL, 0.150 mmol), $Pd(OAc)_2$ (2.2 mg, 10.0 µmol), *N*-acetylglycine (2.3 mg, 20.0 µmol), and AgOAc (50.1 mg, 0.300 mmol) in hexafluoroisopropanol (2.00 mL) in a 2-dram vial with a PTFE-lined cap was heated to 90 °C and stirred for 3 h. Upon completion, the reaction was cooled to ambient temperature, ethylenediamine (40 µL) and celite (70 mg) were added, and the resulting mixture was stirred overnight. The mixture was filtered through a plug of SiO_2 (5 mm x 20 mm, EtOAc eluent, ~20 mL), the filtrate was concentrated by rotary evaporation, and the resulting residue was dissolved in EtOAc and then filtered through a second plug of SiO_2 (5 mm x 20 mm, EtOAc eluent, ~20 mL). The solution was concentrated by rotary evaporation, EtOH (1.00 mL) was added to the residue, and anhydrous HCl was bubbled through the solution for 1 min. The solution was stirred at 23 °C for 20 h. Sat. aq. Na_2CO_3 (5 mL) was added to the reaction mixture, and it was extracted with EtOAc (3 x 10 mL), washed with brine (5 mL), and dried with $MgSO_4$. The solution was concentrated by rotary evaporation, and the crude residue was purified by flash column chromatography (7:3 hexanes/EtOAc eluent) to afford alcohol **9aa** (10.7 mg, 12:1 mono/di, 54% yield, R_f = 0.33 in 7:3 hexanes/EtOAc) as a colorless oil. An additional portion of the diolefinated alcohol (2.5 mg, 9% yield) was isolated as the isobenzofuran.

Alcohol 9aa$_{mono}$: ^{1}H NMR (400 MHz, $CDCl_3$) δ 8.03 (d, *J* = 16.0 Hz, 1H), 7.61 (d, *J* = 7.4 Hz, 1H), 7.45 (d, *J* = 7.4 Hz, 1H), 7.39 (t, *J* = 7.4 Hz, 1H), 7.34 (t, *J* = 7.4 Hz, 1H), 6.41 (d, *J* = 16.0 Hz, 1H), 4.84 (s, 1H), 4.27 (q, *J* = 7.2 Hz, 2H), 1.81 (br. s, 1H), 1.34 (t, *J* = 7.2 Hz, 3H); ^{13}C NMR (125 MHz, $CDCl_3$) δ 167.1, 141.5, 139.6, 133.3, 130.3, 128.9, 128.5, 127.1, 120.6, 63.2, 60.9, 14.5; IR (film) 3455, 1701, 1318, 1182, 914 cm^{-1}; HRMS (ESI+) *m/z* calc'd for $(M_{mono} + Na)^+$ $[C_{12}H_{14}O_3 + Na]^+$: 229.0835, found 229.0836.

Diolefin 9aa: Representative peaks in ^{1}H NMR (400 MHz, $CDCl_3$) δ 8.14 (d, *J* = 16.0 Hz, 2H), 6.40 (d, *J* = 16.0 Hz, 2H); HRMS (ESI+) *m/z* calc'd for $(M_{di} + Na)^+$ $[C_{17}H_{20}O_5 + Na]^+$: 327.1203, found 327.1203.

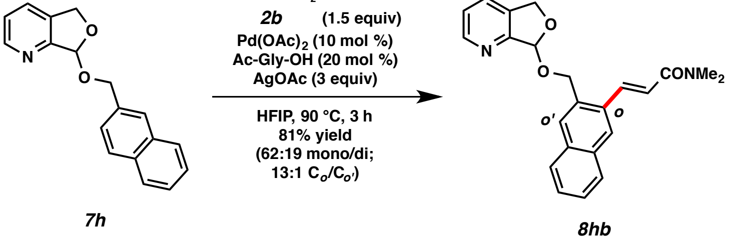

7h *8hb*

A suspension of acetal **7h** (27.7 mg, 0.100 mmol), *N,N*-dimethylacrylamide (**2b**, 15.5 µL, 0.150 mmol), $Pd(OAc)_2$ (2.2 mg, 10.0 µmol), *N*-acetylglycine (2.3 mg, 20.0 µmol), and AgOAc (50.1 mg, 0.300

mmol) in hexafluoroisopropanol (2.00 mL) in a 2-dram vial with a PTFE-lined cap was heated at 90 °C and stirred for 3 h. Upon completion, the reaction was cooled to ambient temperature, ethylenediamine (40 µL) and celite (70 mg) were added, and the resulting mixture was stirred overnight. The mixture was filtered through a plug of SiO_2 (5 mm x 20 mm, EtOAc → 1:1 EtOAc/MeOH eluent, ~20 mL), the filtrate was concentrated by rotary evaporation, and the resulting residue was dissolved in EtOAc and then filtered through a second plug of SiO_2 (5 mm x 20 mm, EtOAc → 1:1 EtOAc/MeOH eluent, ~20 mL). The filtrate was concentrated by rotary evaporation, and the crude residue was purified by flash column chromatography (9:1 EtOAc/MeOH eluent) to afford monoolefin **8hb$_{mono}$** (23.3 mg, 13:1 *o*/*o'*, 62% yield, R_f = 0.37 in 9:1 EtOAc/MeOH) as a beige solid. The column was washed with MeOH, the filtrate concentrated by rotary evaporation, and the residue was purified by preparatory plate chromatography (9:1 EtOAc/MeOH eluent) to afford diolefin **8hb$_{di}$** (8.8 mg, 19% yield, R_f = 0.56 in 9:1 EtOAc/MeOH) as a white solid.

Monoolefin 8hb$_{mono}$: ^{1}H NMR (400 MHz, $CDCl_3$) δ 8.56 (d, *J* = 4.8 Hz, 1H), 8.11 (d, *J* = 15.4 Hz, 1H), 8.01 (s, 1H), 7.94 (s, 1H), 7.84-7.78 (comp. m, 2H), 7.65 (d, *J* = 7.6 Hz, 1H), 7.49-7.43 (comp. m, 2H), 7.26 (dd, *J* = 7.6, 4.8 Hz, 1H), 6.96 (d, *J* = 15.4 Hz, 1H), 6.32 (d, *J* = 1.2 Hz, 1H), 5.49 (dd, *J* = 13.2, 1.2 Hz, 1H), 5.24 (d, *J* = 11.4 Hz, 1H), 5.16 (d, *J* = 13.2 Hz, 1H), 4.95 (d, *J* = 11.4 Hz, 1H), 3.20 (s, 3H), 3.10 (s, 3H); ^{13}C NMR (100 MHz, $CDCl_3$) δ 166.8, 157.5, 149.8, 140.0, 133.63, 133.57, 133.4, 133.0, 130.0, 129.1, 128.0, 127.9, 126.8, 126.6, 126.4, 123.7, 120.5, 104.3, 70.8, 68.4, 37.7, 36.1; IR (film) 1652, 1584, 1389, 994, 910 cm^{-1}; HRMS (ESI+) *m/z* calc'd for $(M + H)^+$ $[C_{23}H_{22}N_2O_3 + H]^+$: 375.1703, found 375.1702.

Diolefin 8hb$_{di}$: ^{1}H NMR (400 MHz, $CDCl_3$) δ 8.47 (d, *J* = 4.8 Hz, 1H), 8.29 (d, *J* = 15.2 Hz, 1H), 8.28 (d, *J* = 15.6 Hz, 1H), 8.04-7.99 (comp. m, 1H), 7.98 (s, 1H), 7.83-7.79 (comp. m, 1H), 7.63 (d, *J* = 7.6 Hz, 1H), 7.51-7.47 (comp. m, 2H), 7.22 (dd, *J* = 7.6, 4.8 Hz, 1H), 7.02 (d, *J* = 15.6 Hz, 1H), 6.95 (d, *J* = 15.2 Hz, 1H), 6.28 (s, 1H), 5.60 (d, *J* = 13.6 Hz, 1H), 5.20 (d, *J* = 13.6 Hz, 1H), 5.13 (d, *J* = 9.8 Hz, 1H), 4.88 (d, *J* = 9.8 Hz, 1H), 3.29 (s, 3H), 3.22 (s, 3H), 3.16 (s, 3H), 3.12 (s, 3H); ^{13}C NMR (125 MHz, $CDCl_3$) δ 166.6, 166.1, 157.4, 149.5, 140.8, 138.6, 136.4, 135.0, 133.5, 132.8, 132.1, 130.3, 129.8, 128.4, 127.0, 126.9, 126.6, 126.5, 125.6, 123.5, 121.1, 104.1, 70.9, 65.0, 37.71, 37.67, 36.1; IR (film) 1649, 1607, 1008, 914, 730 cm^{-1}; HRMS (ESI+) *m/z* calc'd for $(M + H)^+$ $[C_{28}H_{29}N_3O_4 + H]^+$: 472.2231, found 472.2232.

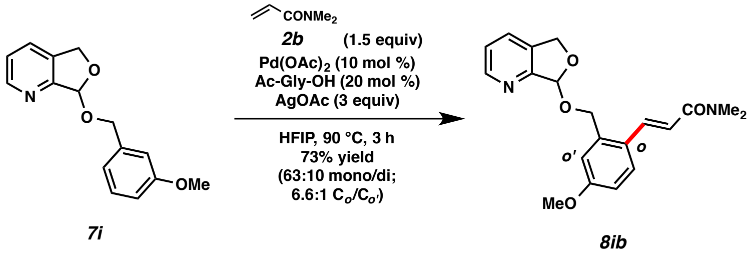

A suspension of acetal **7i** (25.5 mg, 92.7 µmol), *N,N*-dimethylacrylamide (**2b**, 14.3 µL, 0.139 mmol), $Pd(OAc)_2$ (2.1 mg, 9.27 µmol), *N*-acetylglycine (2.2 mg, 19.0 µmol), and AgOAc (46.4 mg, 0.278 mmol) in hexafluoroisopropanol (1.85 mL) in a 2-dram vial with a PTFE-lined cap was heated at 90 °C and stirred for 3 h. Upon completion, the reaction was cooled to ambient temperature, ethylenediamine (40 µL) and celite (70 mg) were added, and the resulting mixture was stirred overnight. The mixture was filtered through a plug of SiO_2 (5 mm x 20 mm, EtOAc → 1:1 EtOAc/MeOH eluent, ~20 mL), the filtrate was concentrated by rotary evaporation, and the resulting residue was dissolved in EtOAc and then filtered through a second plug of SiO_2 (5 mm x 20 mm, EtOAc → 1:1 EtOAc/MeOH eluent, ~20

mL). The filtrate was concentrated by rotary evaporation, and the crude residue was purified by flash column chromatography (EtOAc eluent) to afford monoolefin **8ib$_{mono}$** (20.6 mg, 6.6:1 *o*/*o'*, 63% yield, R_f = 0.33 in EtOAc) as a white film. The column was washed with MeOH, the filtrate concentrated by rotary evaporation, and the residue was purified by preparatory plate chromatography (9:1 EtOAc/MeOH eluent) to afford diolefin **8ib$_{di}$** (5.1 mg, 10% yield, R_f = 0.19 in 9:1 EtOAc/MeOH) as a white solid.

Monoolefin 8ib$_{mono}$: ^{1}H NMR (400 MHz, CDCl$_3$) δ 8.54 (d, *J* = 4.8 Hz, 1H), 7.92 (d, *J* = 15.4 Hz, 1H), 7.63 (d, *J* = 7.6 Hz, 1H), 7.51 (d, *J* = 8.4 Hz, 1H), 7.25 (dd, *J* = 7.6, 4.8 Hz, 1H), 7.06 (d, *J* = 2.4 Hz, 1H), 6.81 (dd, *J* = 8.4, 2.4 Hz, 1H), 6.72 (d, *J* = 15.4 Hz, 1H), 6.25 (s, 1H), 5.43 (d, *J* = 13.2 Hz, 1H), 5.13 (d, *J* = 13.2 Hz, 1H), 5.07 (d, *J* = 11.4 Hz, 1H), 4.79 (d, *J* = 11.4 Hz, 1H), 3.82 (s, 3H), 3.14 (s, 3H), 3.06 (s, 3H); ^{13}C NMR (100 MHz, CDCl$_3$) δ 167.1, 160.6, 157.4, 149.7, 138.9, 138.2, 133.5, 130.0, 128.1, 127.1, 123.7, 117.6, 114.7, 114.3, 104.3, 70.7, 67.7, 55.5, 37.6, 36.1; IR (film) 1647, 1594, 1505, 1394, 1262 cm^{-1}; HRMS (ESI+) *m/z* calc'd for $(M + H)^+$ $[C_{20}H_{22}N_2O_4 + H]^+$: 355.1652, found 355.1651.

Diolefin 8ib$_{di}$: ^{1}H NMR (400 MHz, CDCl$_3$) δ 8.47 (d, *J* = 4.8 Hz, 1H), 8.12 (d, *J* = 15.6 Hz, 1H), 7.89 (d, *J* = 15.6 Hz, 1H), 7.62 (d, *J* = 7.6 Hz, 1H), 7.51 (d, *J* = 8.8 Hz, 1H), 7.22 (dd, *J* = 7.6, 4.8 Hz, 1H), 7.08 (d, *J* = 15.6 Hz, 1H), 6.85 (d, *J* = 8.8 Hz. 1H), 6.75 (d, *J* = 15.6 Hz, 1H), 6.25 (s, 1H), 5.58 (d, *J* = 13.6 Hz, 1H), 5.20 (d, *J* = 13.6 Hz, 1H), 5.02 (d, *J* = 9.6 Hz, 1H), 4.72 (d, *J* = 9.6 Hz, 1H), 3.83 (s, 3H), 3.25 (s, 3H), 3.18 (s, 3H), 3.10 (s, 3H), 3.08 (s, 3H); ^{13}C NMR (100 MHz, CDCl$_3$) δ 169.8, 167.0, 158.9, 157.5, 149.6, 140.1, 136.0, 134.6, 133.7, 130.1, 129.9, 128.1, 127.8, 124.7, 123.6, 119.2, 110.8, 104.1, 71.0, 64.1, 55.9, 37.8, 37.7, 36.1; IR (film) 1649, 1601, 1397, 1266, 1008 cm^{-1}; HRMS (ESI+) *m/z* calc'd for $(M + H)^+$ $[C_{25}H_{29}N_3O_5 + H]^+$: 452.2180, found 452.2179.

Note on characterization: Monoolefin **8ib$_{mono}$** is characterized as being functionalized at *o* instead of *o'* by the presence of a doublet with the small J coupling at 7.06 ppm. The J coupling observed is presumably due to W-coupling. Functionalization at o' would result in a product with three aromatic signals with larger J coupling values (two doublets and a doublet of doublets). These signals are not observed in the major olefinic product.

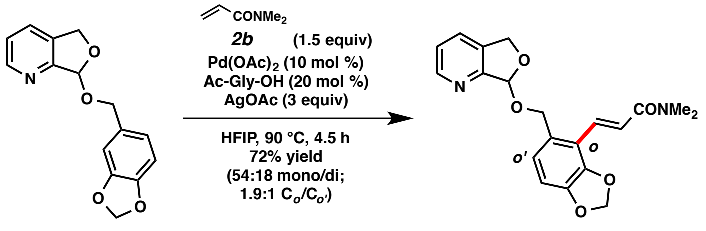

7j **8jb**

A suspension of acetal **7j** (27.1 mg, 0.100 mmol), *N,N*-dimethylacrylamide (**2b**, 15.5 µL, 0.150 mmol), Pd(OAc)$_2$ (2.2 mg, 10.0 µmol), *N*-acetylglycine (2.3 mg, 20.0 µmol), and AgOAc (50.1 mg, 0.300 mmol) in hexafluoroisopropanol (2.00 mL) in a 2-dram vial with a PTFE-lined cap was heated at 90 °C and stirred for 3 h. Upon completion, the reaction was cooled to ambient temperature, ethylenediamine (40 µL) and celite (70 mg) were added, and the resulting mixture was stirred overnight. The mixture was filtered through a plug of SiO$_2$ (5 mm x 20 mm, EtOAc → 1:1 EtOAc/MeOH eluent, ~20 mL), the filtrate was concentrated by rotary evaporation, and the resulting residue was dissolved in EtOAc and then filtered through a second plug of SiO$_2$ (5 mm x 20 mm, EtOAc → 1:1 EtOAc/MeOH eluent, ~20 mL). The filtrate was concentrated by rotary evaporation, and the crude residue was purified by flash column chromatography (9:1 EtOAc/MeOH eluent) to afford monoolefin **8jb$_{mono}$** (20.0 mg, 1:0.53 *o*/*o'*,

54% yield, R_f = 0.33 in 9:1 EtOAc/MeOH) as a white solid. The column was washed with MeOH, the filtrate concentrated by rotary evaporation, and the residue was purified by preparatory plate chromatography (9:1 EtOAc/MeOH eluent) to afford diolefin **8jb$_{di}$** (8.2 mg, 18% yield, R_f = 0.12 in 9:1 EtOAc/MeOH) as a white solid.

Monoolefin 8jb$_{mono}$: ^{1}H NMR (400 MHz, $CDCl_3$) δ 8.55 (d, *J* = 4.8 Hz, 0.53H), 8.52 (d, *J* = 4.8 Hz, 1H), 7.93 (d, *J* = 15.2 Hz, 0.53H), 7.84 (d, *J* = 15.6 Hz, 1H), 7.63 (app. d, *J* = 7.6 Hz, 1.53H), 7.28-7.21 (comp. m, 2.53H), 7.03 (s, 0.53H), 6.98 (s, 0.53H), 6.95 (d, *J* = 8.0 Hz, 1H), 6.73 (d, *J* = 8.0 Hz, 1H), 6.69 (d, *J* = 15.2 Hz, 0.53H), 6.23 (app. s, 1.53H), 6.04 (s, 1H), 6.01 (s, 1H), 5.96 (s, 0.53H), 5.95 (s, 0.53H), 5.52 (d, *J* = 13.6 Hz, 1H), 5.42 (d, *J* = 13.2 Hz, 0.53H), 5.18-4.98 (comp. m, 3.06H), 4.76-4.67 (comp. m, 1.53H), 3.14 (s, 0.53H), 3.13 (s, 1H), 3.06 (app. s, 1.53H); ^{13}C NMR (100 MHz, $CDCl_3$) δ 167.3, 166.9, 157.6, 149.8, 149.7, 148.8, 147.85, 147.82, 146.7, 138.9, 133.9, 133.7, 133.6, 131.5, 130.1, 130.0, 128.8, 124.3, 123.64, 123.57, 122.9, 118.2, 118.1, 110.4, 108.4, 106.1, 104.2, 104.1, 101.5, 101.4, 70.8, 70.7, 68.1, 67.4, 37.6, 36.1, 36.0; IR (film) 1642, 1584, 1394, 1452, 999 cm^{-1}; HRMS (ESI+) *m/z* calc'd for $(M + H)^+$ $[C_{20}H_{20}N_2O_5 + H]^+$: 369.1445, found 369.1446.

Diolefin 8jb$_{di}$: ^{1}H NMR (400 MHz, $CDCl_3$) δ 8.50 (d, *J* = 4.8 Hz, 1H), 8.01 (d, *J* = 15.2 Hz, 1H), 7.90 (d, *J* = 15.2 Hz, 1H), 7.62 (d, *J* = 7.6 Hz, 1H), 7.23 (d, *J* = 15.2 Hz, 1H), 7.21 (dd, *J* = 7.6, 4.8 Hz, 1H), 6.95 (s, 1H), 6.74 (d, *J* = 15.2 Hz, 1H), 6.26 (s, 1H), 6.06 (s, 1H), 6.02 (s, 1H), 5.62 (d, *J* = 10.0 Hz, 1H), 5.19 (d, *J* = 10.0 Hz, 1H), 5.06 (d, *J* = 10.6 Hz, 1H), 4.84 (d, *J* = 10.6 Hz, 1H), 3.19 (s, 3H), 3.16 (s, 3H), 3.08 (app. s, 6H); ^{13}C NMR (125 MHz, $CDCl_3$) δ 167.1, 166.7, 157.4, 149.6, 147.9, 147.3, 139.9, 133.81, 133.76, 131.6, 130.0, 128.8, 124.0, 123.5, 120.4, 123.5, 120.4, 119.5, 107.1, 104.0, 101.7, 70.9, 62.7, 37.71, 37.66, 36.12, 36.06; IR (film) 1649, 1607, 1391, 1003, 730 cm^{-1}; HRMS (ESI+) *m/z* calc'd for $(M + H)^+$ $[C_{25}H_{27}N_3O_6 + H]^+$: 466.1973, found 466.1972.

Note on characterization: Monoolefin **8jb$_{mono}$** is characterized as being predominantly functionalized at *o* instead of *o'* by characteristic peaks in the 1H NMR. The major product has two doublets at 6.95 and 6.73 ppm with a J coupling of 8.0 Hz, consistent with an ortho relationship between two aromatic protons. The minor product has two singlets at 7.03 and 6.98 ppm, consistent with a para relationship between two aromatic protons.

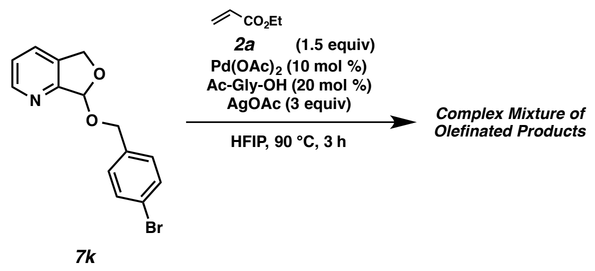

A suspension of acetal **7k** (30.6 mg, 0.100 mmol), ethyl acrylate (**2a**, 16.0 µL, 0.150 mmol), $Pd(OAc)_2$ (2.2 mg, 10.0 µmol), *N*-acetylglycine (2.3 mg, 20.0 µmol), and AgOAc (50.1 mg, 0.300 mmol) in hexafluoroisopropanol (2.00 mL) in a 2-dram vial with a PTFE-lined cap was heated at 90 °C and stirred for 3 h. Upon completion, the reaction was cooled to ambient temperature, ethylenediamine (40 µL) and celite (70 mg) were added, and the resulting mixture was stirred overnight. The mixture was filtered through a plug of SiO_2 (5 mm x 20 mm, MeOH eluent, ~20 mL), the filtrate was concentrated by rotary evaporation, and the resulting residue was dissolved in EtOAc and then filtered through a second plug of SiO_2 (5 mm x 20 mm, MeOH eluent, ~20 mL). The filtrate was concentrated by rotary evaporation, and a complex mixture of olefinated products was observed by NMR in the crude residue.

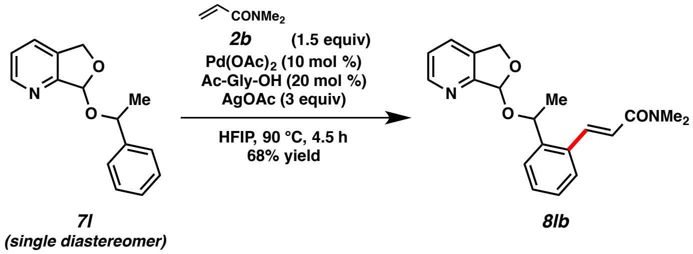

A suspension of acetal **7l** (24.1 mg, 0.100 mmol), *N,N*-dimethylacrylamide (**2b**, 15.5 µL, 0.150 mmol), $Pd(OAc)_2$ (2.2 mg, 10.0 µmol), *N*-acetylglycine (2.3 mg, 20.0 µmol), and AgOAc (50.1 mg, 0.300 mmol) in hexafluoroisopropanol (2.00 mL) in a 2-dram vial with a PTFE-lined cap was heated at 90 °C and stirred for 6 h. Upon completion, the reaction was cooled to ambient temperature, ethylenediamine (40 µL) and celite (70 mg) were added, and the resulting mixture was stirred overnight. The mixture was filtered through a plug of SiO_2 (5 mm x 20 mm, EtOAc → 9:1 EtOAc/MeOH eluent, ~20 mL), the filtrate was concentrated by rotary evaporation, and the resulting residue was dissolved in EtOAc and then filtered through a second plug of SiO_2 (5 mm x 20 mm, EtOAc → 9:1 EtOAc/MeOH eluent, ~20 mL). The filtrate was concentrated by rotary evaporation, and the crude residue was purified by flash column chromatography (EtOAc eluent) to afford olefin **8lb** (23.0 mg, 68% yield, R_f = 0.33 in EtOAc) as a white film.

Olefin 8lb: ^{1}H NMR (400 MHz, $CDCl_3$) δ 8.58 (d, *J* = 4.8 Hz, 1H), 8.06 (d, *J* = 15.2 Hz, 1H), 7.82 (d, *J* = 8.0 Hz, 1H), 7.62 (d, *J* = 7.6 Hz, 1H), 7.51 (d, *J* = 7.6 Hz, 1H), 7.47 (t, *J* = 7.6 Hz, 1H), 7.29 (t, *J* = 7.6 Hz, 1H), 7.26 (dd, *J* = 8.0, 4.8 Hz, 1H), 6.79 (d, *J* = 15.2 Hz, 1H), 5.93 (s, 1H), 5.51 (q, *J* = 6.4 Hz, 1H), 5.24 (d, *J* = 13.2 Hz, 1H), 5.02 (d, *J* = 13.2 Hz, 1H), 3.18 (s, 3H), 3.08 (s, 3H), 1.45 (d, *J* = 6.4 Hz, 3H); ^{13}C NMR (100 MHz, $CDCl_3$) δ 166.6, 158.0, 149.8, 142.2, 139.5, 134.0, 133.4, 130.1, 129.9, 127.6, 126.7, 126.6, 123.5, 120.4, 102.1, 70.9, 70.2, 37.6, 36.0; IR (film) 1642, 1589, 1389, 973, 910 cm^{-1}; HRMS (ESI+) *m/z* calc'd for $(M + H)^+$ $[C_{20}H_{22}N_2O_3 + H]^+$: 339.1703, found 339.1704.

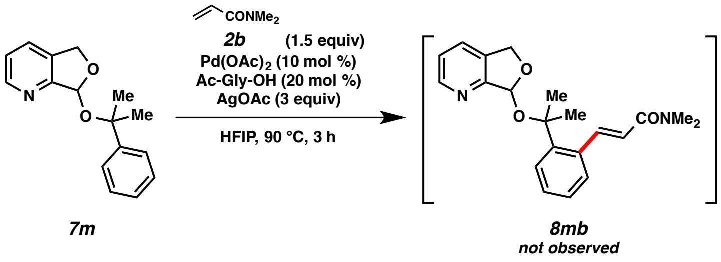

A suspension of acetal **7m** (25.5 mg, 0.100 mmol), *N,N*-dimethylacrylamide (**2b**, 15.5 µL, 0.150 mmol), $Pd(OAc)_2$ (2.2 mg, 10.0 µmol), *N*-acetylglycine (2.3 mg, 20.0 µmol), and AgOAc (50.1 mg, 0.300 mmol) in hexafluoroisopropanol (2.00 mL) in a 2-dram vial with a PTFE-lined cap was heated at 90 °C and stirred for 3 h. Upon completion, the reaction was cooled to ambient temperature, ethylenediamine (40 µL) and celite (70 mg) were added, and the resulting mixture was stirred overnight. The mixture was filtered through a plug of SiO_2 (5 mm x 20 mm, MeOH eluent, ~20 mL), the filtrate was concentrated by rotary evaporation, and the resulting residue was dissolved in EtOAc and then filtered through a second plug of SiO_2 (5 mm x 20 mm, MeOH eluent, ~20 mL). The filtrate was concentrated by rotary evaporation, and olefin **8mb** was not observed by NMR in the crude residue.

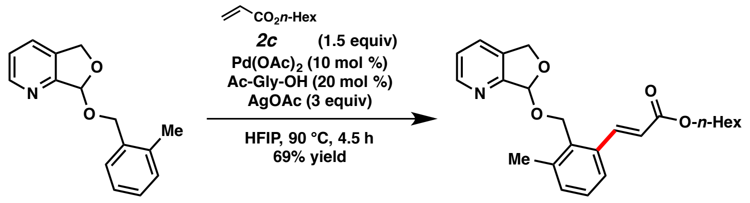

7b **8bc**

A suspension of acetal **7b** (24.1 mg, 0.100 mmol), *n*-hexyl acrylate (**2c**, 26.4 μL, 0.150 mmol), $Pd(OAc)_2$ (2.2 mg, 10.0 μmol), *N*-acetylglycine (2.3 mg, 20.0 μmol), and AgOAc (50.1 mg, 0.300 mmol) in hexafluoroisopropanol (2.00 mL) in a 2-dram vial with a PTFE-lined cap was heated at 90 °C and stirred for 4.5 h. Upon completion, the reaction was cooled to ambient temperature, ethylenediamine (40 μL) and celite (70 mg) were added, and the resulting mixture was stirred overnight. The mixture was filtered through a plug of SiO_2 (5 mm x 20 mm, EtOAc eluent, ~20 mL), the filtrate was concentrated by rotary evaporation, and the resulting residue was dissolved in EtOAc and then filtered through a second plug of SiO_2 (5 mm x 20 mm, EtOAc eluent, ~20 mL). The filtrate was concentrated by rotary evaporation, and the crude residue was purified by flash column chromatography (13:7 hexanes/EtOAc) to afford olefin **8bc** (27.2 mg, 69% yield, R_f = 0.37 in 13:7 hexanes/EtOAc) as a colorless oil.

Olefin 8bc: ^{1}H NMR (400 MHz, $CDCl_3$) δ 8.55 (d, *J* = 4.8 Hz, 1H), 8.20 (d, *J* = 15.6 Hz, 1H), 7.63 (d, *J* = 7.6 Hz, 1H), 7.40 (t, *J* = 4.4 Hz, 1H), 7.25 (dd, *J* = 7.6, 4.8 Hz, 1H), 7.20 (d, *J* = 4.4 Hz, 2H), 6.36 (d, *J* = 15.6 Hz, 1H), 6.24 (s, 1H), 5.43 (d, *J* = 12.8 Hz, 1H), 5.15 (d, *J* = 12.8 Hz, 1H), 5.05 (d, *J* = 10.8 Hz, 1H), 4.85 (d, *J* = 12.8 Hz, 1H), 4.21 (t, *J* = 6.8 Hz, 2H), 2.49 (s, 3H), 1.71 (quint, *J* = 6.8 Hz, 2H), 1.46-1.36 (comp. m, 2H), 1.36-1.27 (comp. m, 4H), 0.90 (t, *J* = 6.8 Hz, 3H); ^{13}C NMR (100 MHz, $CDCl_3$) δ 167.9, 157.5, 149.8, 143.1, 139.1, 135.6, 134.1, 133.4, 132.2, 129.9, 128.6, 124.9, 123.6, 120.8, 104.3, 70.7, 64.8, 63.6, 31.6, 28.8, 25.8, 22.7, 19.9, 14.2; IR (film) 1711, 1313, 1166, 1003, 793 cm^{-1}; HRMS (ESI+) *m/z* calc'd for $(M + H)^+$ $[C_{24}H_{29}NO_4 + H]^+$: 396.2169, found 396.2174.

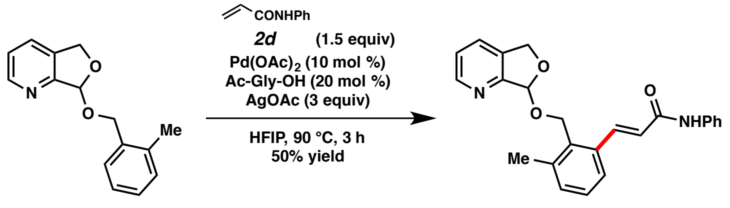

7b **8bd**

A suspension of acetal **7b** (24.1 mg, 0.100 mmol), *N*-phenylacrylamide (**2d**, 22.1 mg, 0.150 mmol), $Pd(OAc)_2$ (2.2 mg, 10.0 μmol), *N*-acetylglycine (2.3 mg, 20.0 μmol), and AgOAc (50.1 mg, 0.150 mmol) in hexafluoroisopropanol (2.00 mL) in a 2-dram vial with a PTFE-lined cap was heated at 90 °C and stirred for 3 h. Upon completion, the reaction was cooled to ambient temperature, ethylenediamine (40 μL) and celite (70 mg) were added, and the resulting mixture was stirred overnight. The mixture was filtered through a plug of SiO_2 (5 mm x 20 mm, EtOAc → 9:1 EtOAc/MeOH eluent, ~20 mL), the filtrate was concentrated by rotary evaporation, and the resulting residue was dissolved in EtOAc and then filtered through a second plug of SiO_2 (5 mm x 20 mm, EtOAc → 9:1 EtOAc/MeOH eluent, ~20 mL). The filtrate was concentrated by rotary evaporation, and the crude residue was purified by flash column chromatography (1:1 hexanes/EtOAc eluent) to afford olefin **8bd** (19.5 mg, 50% yield, R_f = 0.27 in 1:1 hexanes/EtOAc) as a light green oil.

Olefin 8bd: ^{1}H NMR (400 MHz, $CDCl_3$) δ 9.87 (br. s, 1H), 8.60 (d, *J* = 5.2 Hz, 1H), 7.89 (d, *J* = 15.8 Hz, 1H), 7.76 (d, *J* = 7.6 Hz, 2H), 7.73 (d, *J* = 8.0 Hz, 1H), 7.36 (dd, *J* = 8.0, 5.2 Hz, 1H), 7.35 (t, *J* = 7.6 Hz, 2H), 7.28 (d, *J* = 8.0 Hz, 1H), 7.23 (t, *J* = 7.6 Hz, 1H), 7.16 (d, *J* = 6.8 Hz, 1H), 7.14 (d, *J* = 15.8 Hz, 1H), 7.11 (dd, *J* = 8.0, 6.8 Hz, 1H), 6.37 (s, 1H), 5.38 (d, *J* = 13.4 Hz, 1H), 5.20 (d, *J* = 13.4 Hz, 1H), 5.12 (d, *J* = 9.2 Hz, 1H), 4.70 (d, *J* = 9.2 Hz, 1H), 2.51 (t, 3H); ^{13}C NMR (100 MHz, $CDCl_3$) δ 169.8, 164.9, 157.4, 149.3, 139.7, 139.4, 138.3, 133.9, 132.5, 130.9, 130.8, 129.1, 128.7, 127.7, 124.4, 124.0, 119.9, 104.9, 70.9, 66.7, 19.7; IR (film) 3263, 1596, 1533, 1444, 1003 cm^{-1}; HRMS (ESI+) *m/z* calc'd for $(M + H)^+$ $[C_{24}H_{22}N_2O_3 + H]^+$: 387.1703, found 387.1703.

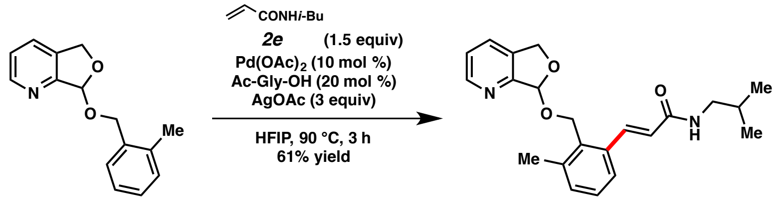

7b 8be

A suspension of acetal **7b** (24.1 mg, 0.100 mmol), *N*-isobutylacrylamide[2] (**2e**, 19.1 mg, 0.150 mmol), $Pd(OAc)_2$ (2.2 mg, 10.0 µmol), *N*-acetylglycine (2.3 mg, 20.0 µmol), and AgOAc (50.1 mg, 0.150 mmol) in hexafluoroisopropanol (2.00 mL) in a 2-dram vial with a PTFE-lined cap was heated at 90 °C and stirred for 3 h. Upon completion, the reaction was cooled to ambient temperature, ethylenediamine (40 µL) and celite (70 mg) were added, and the resulting mixture was stirred overnight. The mixture was filtered through a plug of SiO_2 (5 mm x 20 mm, EtOAc → 9:1 EtOAc/MeOH eluent, ~20 mL), the filtrate was concentrated by rotary evaporation, and the resulting residue was dissolved in EtOAc and then filtered through a second plug of SiO_2 (5 mm x 20 mm, EtOAc → 9:1 EtOAc/MeOH eluent, ~20 mL). The filtrate was concentrated by rotary evaporation, and the crude residue was purified by flash column chromatography (9:1 EtOAc/hexanes eluent) to afford olefin **8be** (22.3 mg, 61% yield, R_f = 0.29 in 9:1 EtOAc/hexanes) as a colorless oil.

Olefin 8be: ^{1}H NMR (400 MHz, $CDCl_3$) δ 8.51 (d, *J* = 4.8 Hz, 1H), 7.82 (d, *J* = 16.0 Hz, 1H), 7.67 (d, *J* = 7.6 Hz, 1H), 7.53 (br. s, 1H), 7.30 (dd, *J* = 7.6, 4.8 Hz, 1H), 7.27 (d, *J* = 7.4 Hz, 1H), 7.20 (t, *J* = 7.4 Hz, 1H), 7.14 (d, *J* = 7.4 Hz, 1H), 6.83 (d, *J* = 16.0 Hz, 1H), 6.25 (s, 1H), 5.37 (d, *J* = 13.6 Hz, 1H), 5.16 (d, *J* = 13.6 Hz, 1H), 5.09 (d, *J* = 9.6 Hz, 1H), 4.69 (d, *J* = 9.6 Hz, 1H), 3.26 (t, *J* = 6.8 Hz, 2H), 2.48 (s, 3H), 1.96-1.84 (m, 1H), 0.98 (d, *J* = 6.8 Hz, 6H); ^{13}C NMR (100 MHz, $CDCl_3$) δ 166.7, 157.4, 149.4, 139.2, 138.2, 138.1, 133.6, 132.7, 130.7, 130.4, 128.6, 126.9, 126.8, 124.0, 104.9, 70.8, 66.2, 47.3, 28.8, 20.49, 20.46, 19.7; IR (film) 3300, 1003, 1659, 1622, 1549 cm^{-1}; HRMS (ESI+) *m/z* calc'd for $(M + Na)^+$ $[C_{22}H_{26}N_2O_3 + Na]^+$: 389.1836, found 389.1838.

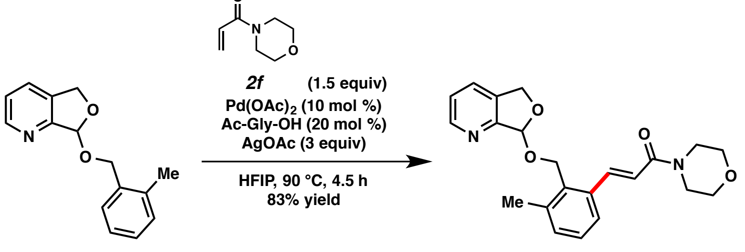

7b 8bf

A suspension of acetal **7b** (24.1 mg, 0.100 mmol), acrylamide **2f**[2] (21.2 mg, 0.15 mmol), $Pd(OAc)_2$ (2.2 mg, 10.0 µmol), *N*-acetylglycine (2.3 mg, 20.0 µmol), and AgOAc (50.1 mg, 0.150 mmol) in hexafluoroisopropanol (2.00 mL) in a 2-dram vial with a PTFE-lined cap was heated at 90 °C and stirred for 4.5 h. Upon completion, the reaction was cooled to ambient temperature, ethylenediamine (40 µL) and celite (70 mg) were added, and the resulting mixture was stirred overnight. The mixture was filtered through a plug of SiO_2 (5 mm x 20 mm, EtOAc → 9:1 EtOAc/MeOH eluent, ~20 mL), the filtrate was concentrated by rotary evaporation, and the resulting residue was dissolved in EtOAc and then filtered through a second plug of SiO_2 (5 mm x 20 mm, EtOAc → 9:1 EtOAc/MeOH eluent, ~20 mL). The filtrate was concentrated by rotary evaporation, and the crude residue was purified by flash column chromatography (19:1 EtOAc/hexanes eluent) to afford olefin **8bf** (31.4 mg, 83% yield, R_f = 0.29 in 19:1 EtOAc/hexanes) as a white solid.

Olefin 8bf: ^{1}H NMR (400 MHz, $CDCl_3$) δ 8.52 (d, *J* = 4.8 Hz, 1H), 8.07 (d, *J* = 15.4 Hz, 1H), 7.62 (d, *J* = 7.6 Hz, 1H), 7.32 (dd, *J* = 7.6 Hz, 1H), 7.24 (dd, *J* = 7.6, 4.8 Hz, 1H), 7.19 (t, *J* = 7.6 Hz, 1H), 7.18 (d, *J* = 2.4 Hz, 1H), 6.80 (d, *J* = 15.4 Hz, 1H), 6.22 (s, 1H), 5.39 (d, *J* = 13.2 Hz, 1H), 5.14 (d, *J* = 13.2 Hz, 1H), 5.01 (d, *J* = 10.2 Hz, 1H), 4.82 (d, *J* = 10.2 Hz, 1H), 3.80-3.62 (br. m, 8H), 2.49 (s, 3H); ^{13}C NMR (100 MHz, $CDCl_3$) δ 165.8, 157.5, 149.7, 141.6, 139.3, 136.9, 133.6, 133.4, 131.5, 129.9, 128.6, 125.5, 123.6, 120.3, 104.2, 70.7, 67.1, 63.9, 46.5, 42.6, 19.7; IR (film) 1643, 1606, 1428, 1229, 1003 cm^{-1}; HRMS (ESI+) *m/z* calc'd for $(M + H)^+$ $[C_{22}H_{24}N_2O_4 + H]^+$: 381.1809, found 381.1808.

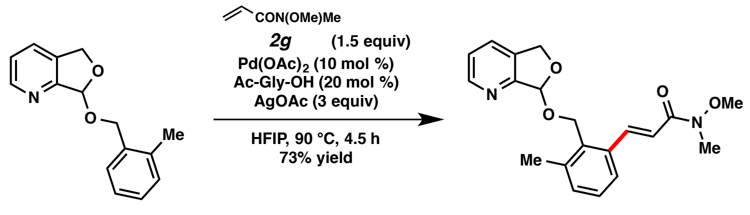

7b *8bg*

A suspension of acetal **7b** (24.1 mg, 0.100 mmol), acrylamide **2g**[3] (17.3 mg, 0.150 mmol), $Pd(OAc)_2$ (2.2 mg, 10.0 µmol), *N*-acetylglycine (2.3 mg, 20.0 µmol), and AgOAc (50.1 mg, 0.150 mmol) in hexafluoroisopropanol (2.00 mL) in a 2-dram vial with a PTFE-lined cap was heated at 90 °C and stirred for 4.5 h. Upon completion, the reaction was cooled to ambient temperature, ethylenediamine (40 µL) and celite (70 mg) were added, and the resulting mixture was stirred overnight. The mixture was filtered through a plug of SiO_2 (5 mm x 20 mm, EtOAc → 9:1 EtOAc/MeOH eluent, ~20 mL), the filtrate was concentrated by rotary evaporation, and the resulting residue was dissolved in EtOAc and then filtered through a second plug of SiO_2 (5 mm x 20 mm, EtOAc → 9:1 EtOAc/MeOH eluent, ~20 mL). The filtrate was concentrated by rotary evaporation, and the crude residue was purified by flash column chromatography (9:1 EtOAc/hexanes eluent) to afford olefin **8bg** (25.9 mg, 73% yield, R_f = 0.21 in 9:1 EtOAc/hexanes) as a white foam.

Olefin 8bg: ^{1}H NMR (400 MHz, $CDCl_3$) δ 8.53 (d, *J* = 4.8 Hz, 1H), 8.22 (d, *J* = 15.6 Hz, 1H), 7.62 (d, *J* = 7.6 Hz, 1H), 7.43 (dd, *J* = 6.4, 2.6 Hz, 1H), 7.23 (dd, *J* = 7.6, 4.8 Hz, 1H), 7.20 (d, *J* = 6.4 Hz, 1H), 7.18 (d, *J* = 2.6 Hz, 1H), 6.93 (d, *J* = 15.6 Hz, 1H), 6.24 (s, 1H), 5.48 (d, *J* = 13.4 Hz, 1H), 5.15 (d, *J* = 13.4 Hz, 1H), 5.07 (d, *J* = 10.4 Hz, 1H), 4.85 (d, *J* = 10.4 Hz, 1H), 3.73 (s, 3H), 3.32 (s, 3H), 2.49 (s, 3H); ^{13}C NMR (100 MHz, $CDCl_3$) δ 167.0, 157.6, 149.7, 141.9, 139.1, 136.5, 134.2, 133.6, 131.8, 129.9, 128.5, 124.9, 123.6, 104.3, 70.7, 63.6, 62.0, 32.7, 19.8; IR (film) 3438, 1651, 1613, 1382, 1003 cm^{-1}; HRMS (ESI+) *m/z* calc'd for $(M + H)^+$ $[C_{22}H_{22}N_2O_4 + H]^+$: 355.1652, found 355.1655.

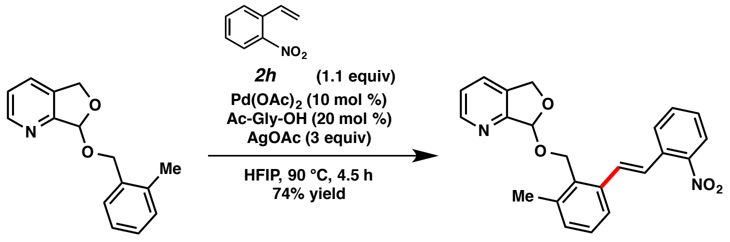

7b **8bh**

A suspension of acetal **7b** (24.1 mg, 0.100 mmol), 2-nitrostyrene[4] (**2h**, 16.4 mg, 0.110 mmol), $Pd(OAc)_2$ (2.2 mg, 10.0 µmol), *N*-acetylglycine (2.3 mg, 20.0 µmol), and AgOAc (50.1 mg, 0.300 mmol) in hexafluoroisopropanol (2.00 mL) in a 2-dram vial with a PTFE-lined cap was heated at 90 °C and stirred for 4.5 h. Upon completion, the reaction was cooled to ambient temperature, ethylenediamine (40 µL) and celite (70 mg) were added, and the resulting mixture was stirred overnight. The mixture was filtered through a plug of SiO_2 (5 mm x 20 mm, EtOAc eluent, ~20 mL), the filtrate was concentrated by rotary evaporation, and the resulting residue was dissolved in EtOAc and then filtered through a second plug of SiO_2 (5 mm x 20 mm, EtOAc eluent, ~20 mL). The filtrate was concentrated by rotary evaporation, and the crude residue was purified by flash column chromatography (3:2 EtOAc/hexanes eluent) to afford stilbene **8bh** (28.9 mg, 74% yield, R_f = 0.27 in 3:2 EtOAc/hexanes) as a yellow solid.

Stillbene 8bh: ^{1}H NMR (400 MHz, $CDCl_3$) δ 8.54 (d, *J* = 4.8 Hz, 1H), 8.97 (app. d, *J* = 8.0 Hz, 2H), 7.69 (d, *J* = 16.2 Hz, 1H), 7.65 (t, *J* = 8.0 Hz, 1H), 7.61 (d, *J* = 8.0 Hz, 1H), 7.50 (d, *J* = 8.0 Hz, 1H), 7.46 (d, *J* = 16.2 Hz, 1H), 7.42 (t, *J* = 8.0 Hz, 1H), 7.27-7.21 (comp. m, 2H), 7.16 (d, *J* = 7.2 Hz, 1H), 7.27 (d, *J* = 1.2 Hz, 1H), 5.25 (dd, *J* = 13.2, 1.2 Hz, 1H), 5.10 (d, *J* = 13.2 Hz, 1H), 4.96 (ABq, *J* = 10.6 Hz, Δν = 24.4 Hz, 2H), 2.50 (s, 3H); ^{13}C NMR (100 MHz, $CDCl_3$) δ 157.5, 149.9, 148.1, 138.7, 137.8, 133.6, 133.18, 133.16, 132.9, 132.4, 130.8, 129.9, 129.0, 128.8, 128.0, 126.0, 124.8, 124.7, 123.7, 104.3, 70.7, 63.8, 19.8; IR (film) 1521, 1342, 994, 910, 725 cm^{-1}; HRMS (ESI+) *m/z* calc'd for $(M + H)^+$ $[C_{23}H_{20}N_2O_4 + H]^+$: 389.1496, found 389.1495.

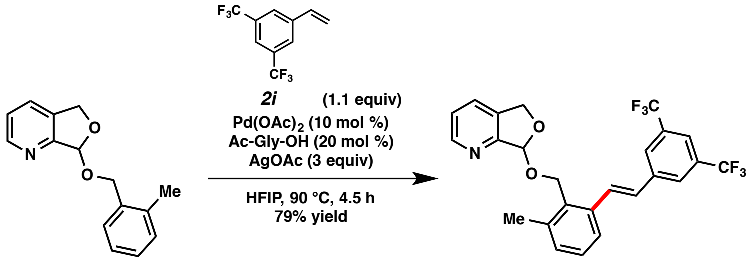

7b **8bi**

A suspension of acetal **7b** (24.1 mg, 0.100 mmol), styrene **2i** (26.4 mg, 0.110 mmol), $Pd(OAc)_2$ (2.2 mg, 10.0 µmol), *N*-acetylglycine (2.3 mg, 20.0 µmol), and AgOAc (50.1 mg, 0.300 mmol) in hexafluoroisopropanol (2.00 mL) in a 2-dram vial with a PTFE-lined cap was heated at 90 °C and stirred for 4.5 h. Upon completion, the reaction was cooled to ambient temperature, ethylenediamine (40 µL) and celite (70 mg) were added, and the resulting mixture was stirred overnight. The mixture was filtered through a plug of SiO_2 (5 mm x 20 mm, EtOAc eluent, ~20 mL), the filtrate was concentrated by rotary evaporation, and the resulting residue was dissolved in EtOAc and then filtered through a second plug of SiO_2 (5 mm x 20 mm, EtOAc eluent, ~20 mL). The filtrate was concentrated by rotary evaporation, and the crude residue was purified by flash column chromatography (7:3

hexanes/EtOAc eluent) to afford stilbene **8bi** (38.0 mg, 79% yield, R_f = 0.29 in 7:3 hexanes/EtOAc) as a white solid.

Stillbene 8bi: ^{1}H NMR (400 MHz, $CDCl_3$) δ 8.57 (d, J = 4.8 Hz, 1H), 8.07 (s, 2H), 7.91 (d, J = 16.2 Hz, 1H), 7.76 (s, 1H), 7.63 (d, J = 7.6 Hz, 1H), 7.46 (d, J = 7.6 Hz, 1H), 7.27 (dd, J = 7.6, 4.8 Hz, 1H), 7.24 (t, J = 7.6 Hz, 1H), 7.17 (d, J = 7.6 Hz, 1H), 7.07 (d, J = 16.2 Hz, 1H), 6.40 (s, 1H), 5.22 (ABq, J = 12.8 Hz, Δν = 49.9 Hz, 2H), 4.96 (ABq, J = 10.4 Hz, Δν = 36.1 Hz, 2H), 2.52 (s, 3H); ^{13}C NMR (100 MHz, $CDCl_3$) δ 157.3, 150.1, 140.2, 138.9, 137.6, 133.1, 133.0, 132.0 (q, $^2J_{C\text{-}F}$ = 33.1 Hz), 131.2, 130.9, 129.9, 128.8, 128.2, 126.7 (q, $^3J_{C\text{-}F}$ = 3.3 Hz), 125.0, 124.4, 123.8, 123.6 (q, $^1J_{C\text{-}F}$ = 271.1 Hz) 122.3, 120.8 (septet, $^3J_{C\text{-}F}$ = 3.6 Hz), 104.5, 70.7, 64.0, 19.9; IR (film) 1381, 1281, 1182, 1134, 736 cm^{-1}; HRMS (ESI+) m/z calc'd for $(M + H)^+$ $[C_{25}H_{19}F_6NO_2 + H]^+$: 480.1393, found 480.1392.

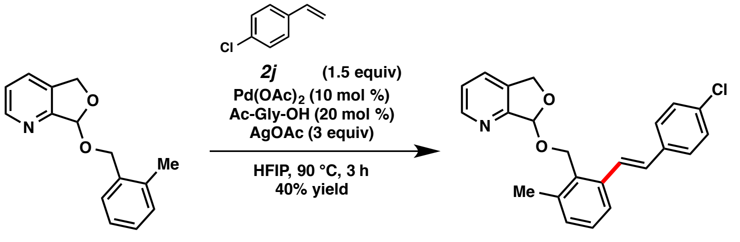

7b 8bj

A suspension of acetal **7b** (24.1 mg, 0.100 mmol), 4-chlorostyrene (**2j**, 20.8 mg, 0.150 mmol), $Pd(OAc)_2$ (2.2 mg, 10.0 µmol), *N*-acetylglycine (2.3 mg, 20.0 µmol), and AgOAc (50.1 mg, 0.150 mmol) in hexafluoroisopropanol (2.00 mL) in a 2-dram vial with a PTFE-lined cap was heated at 90 °C and stirred for 3 h. Upon completion, the reaction was cooled to ambient temperature, ethylenediamine (40 µL) and celite (70 mg) were added, and the resulting mixture was stirred overnight. The mixture was filtered through a plug of SiO_2 (5 mm x 20 mm, EtOAc eluent, ~20 mL), the filtrate was concentrated by rotary evaporation, and the resulting residue was dissolved in EtOAc and then filtered through a second plug of SiO_2 (5 mm x 20 mm, EtOAc eluent, ~20 mL). The filtrate was concentrated by rotary evaporation, and the crude residue was purified by preparatory thin-layer chromatography (7:3 hexanes/EtOAc eluent) to afford olefin **8bj** (15.0 mg, 40% yield, R_f = 0.27 in 7:3 hexanes/EtOAc) as a colorless oil.

Olefin 8bj: ^{1}H NMR (400 MHz, $CDCl_3$) δ 8.57 (d, J = 4.4 Hz, 1H), 7.67 (d, J = 16.0 Hz, 1H), 7.62 (d, J = 8.0 Hz, 1H), 7.54 (d, J = 8.4 Hz, 2H), 7.44 (d, J = 7.6 Hz, 1H), 7.35 (d, J = 8.4 Hz, 2H), 7.26 (dd, J = 8.0, 4.4 Hz, 1H), 7.20 (t, J = 7.6 Hz, 1H), 7.12 (d, J = 7.6 Hz, 1H), 6.93 (d, J = 16.0 Hz, 1H), 6.29 (d, J = 1.6 Hz, 1H), 5.26 (dd, J = 13.2, 1.6 Hz, 1H), 5.13 (d, J = 13.2 Hz, 1H), 4.95 (ABq, J = 10.2 Hz, Δν = 35.4 Hz, 2H), 2.49 (s, 3H); ^{13}C NMR (100 MHz, $CDCl_3$) δ 157.5, 150.0, 138.8, 138.3, 136.5, 133.20, 133.17, 132.7, 130.2, 130.0, 129.9, 128.9, 128.7, 128.1, 127.8, 124.2, 123.7, 104.4, 70.7, 64.0, 19.9; IR (film) 2921, 1589, 1489, 1073, 1005 cm^{-1}; HRMS (ESI+) m/z calc'd for $(M + H)^+$ $[C_{23}H_{20}ClNO_2 + H]^+$: 378.1255, found 378.1261.

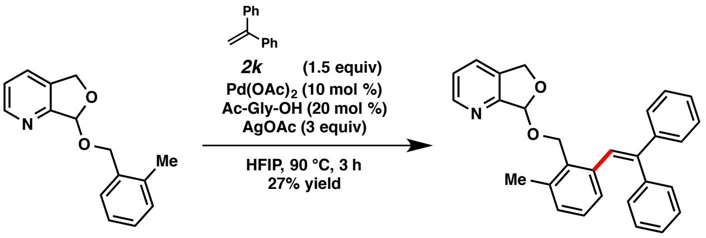

7b 8bk

A suspension of acetal **7b** (24.1 mg, 0.100 mmol), α,α-diphenylethene (**2k**, 26.5 mg, 0.150 mmol), $Pd(OAc)_2$ (2.2 mg, 10.0 µmol), *N*-acetylglycine (2.3 mg, 20.0 µmol), and AgOAc (50.1 mg, 0.300 mmol) in hexafluoroisopropanol (2.00 mL) in a 2-dram vial with a PTFE-lined cap was heated at 90 °C and stirred for 3 h. Upon completion, the reaction was cooled to ambient temperature, ethylenediamine (40 µL) and celite (70 mg) were added, and the resulting mixture was stirred overnight. The mixture was filtered through a plug of SiO_2 (5 mm x 20 mm, EtOAc eluent, ~20 mL), the filtrate was concentrated by rotary evaporation, and the resulting residue was dissolved in EtOAc and then filtered through a second plug of SiO_2 (5 mm x 20 mm, EtOAc eluent, ~20 mL). The filtrate was concentrated by rotary evaporation, and the crude residue was purified by flash column chromatography (7:3 EtOAc/hexanes) to afford a mixture of olefin **8bk** and acetal **7b** (24.3 mg, 27% yield **8bk**, 54% rsm **7b**, R_f = 0.28 in 7:3 EtOAc/hexanes). An analytically pure sample of olefin **8bk** was obtained by preparatory plate chromatography (7:3 EtOAc/hexanes) to afford a white oil.

Olefin 8bk: ^{1}H NMR (400 MHz, $CDCl_3$) δ 8.47 (d, *J* = 4.8 Hz, 1H), 7.57 (d, *J* = 8.0 Hz, 1H), 7.38-7.28 (comp. m, 6H), 7.23-7.15 (comp. m, 4H), 7.09-7.04 (comp. m, 2H), 6.95 (d, *J* = 7.6 Hz, 1H), 6.83 (t, *J* = 7.6 Hz, 1H), 6.66 (d, *J* = 7.6 Hz, 1H), 6.21 (s, 1H), 5.06 (ABq, *J* = 13.8 Hz, Δν = 47.1 Hz, 2H), 4.92 (ABq, *J* = 10.6 Hz, Δν = 34.8 Hz, 2H), 2.46 (s, 3H); ^{13}C NMR (100 MHz, $CDCl_3$) δ 157.8, 149.8, 143.7, 143.6, 140.3, 138.9, 138.3, 133.9, 133.2, 131.0, 129.7, 129.2, 128.3, 128.2, 128.1, 127.7, 127.6, 127.5, 127.1, 123.5, 104.2, 70.6, 64.7, 19.8; IR (film) 1073, 1005, 910, 731, 704 cm^{-1}; HRMS (ESI+) *m/z* calc'd for $(M + H)^+$ $[C_{29}H_{25}NO_2 + H]^+$: 420.1958, found 420.1964.

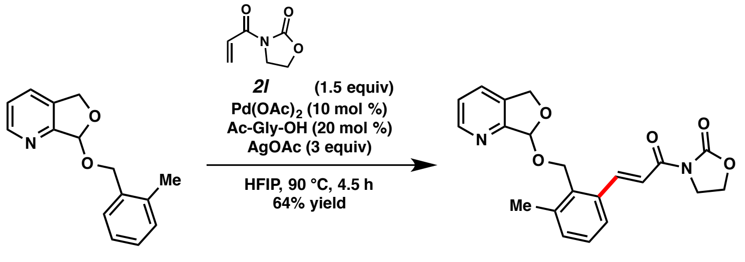

7b *8bl*

A suspension of acetal **7b** (24.1 mg, 0.100 mmol), *N*-acryloyloxazolidinone **2l** (21.1 mg, 0.150 mmol), $Pd(OAc)_2$ (2.2 mg, 10.0 µmol), *N*-acetylglycine (2.3 mg, 20.0 µmol), and AgOAc (50.1 mg, 0.300 mmol) in hexafluoroisopropanol (2.00 mL) in a 2-dram vial with a PTFE-lined cap was heated at 90 °C and stirred for 3 h. Upon completion, the reaction was cooled to ambient temperature, ethylenediamine (40 µL) and celite (70 mg) were added, and the resulting mixture was stirred overnight. The mixture was filtered through a plug of SiO_2 (5 mm x 20 mm, EtOAc → 9:1 EtOAc/MeOH eluent, ~20 mL), the filtrate was concentrated by rotary evaporation, and the resulting residue was dissolved in EtOAc and then filtered through a second plug of SiO_2 (5 mm x 20 mm, EtOAc → 9:1 EtOAc/MeOH eluent, ~20 mL). The filtrate was concentrated by rotary evaporation, and the crude residue was purified by flash column chromatography (4:1 EtOAc/hexanes eluent) to afford olefin **8bl** (24.4 mg, 64% yield, R_f = 0.38 in 4:1 EtOAc/hexanes) as a white solid.

Olefin 8bl: ^{1}H NMR (400 MHz, $CDCl_3$) δ 8.56 (d, *J* = 4.8 Hz, 1H), 8.42 (d, *J* = 15.6 Hz, 1H), 7.80 (d, *J* = 15.6 Hz, 1H), 7.63 (d, *J* = 7.6 Hz, 1H), 7.56 (t, *J* = 4.4 Hz, 1H), 7.25 (dd, *J* = 7.6, 4.8 Hz, 1H), 7.22 (app. d, *J* = 4.4 Hz, 2H), 6.25 (d, *J* = 1.6 Hz, 1H), 5.45 (dd, *J* = 13.2, 1.6 Hz, 1H), 5.16 (d, *J* = 13.2 Hz, 1H), 5.06 (d, *J* = 10.4 Hz, 1H), 4.88 (d, *J* = 10.4 Hz, 1H), 4.46 (t, *J* = 8.0 Hz, 2H), 4.18-4.11 (comp. m, 2H), 2.49 (s, 3H); ^{13}C NMR (100 MHz, $CDCl_3$) δ 165.5, 157.5, 153.7, 149.8, 144.6, 139.0, 135.5,

134.6, 133.5, 132.7, 129.9, 128.8, 125.2, 123.6, 118.9, 104.3, 70.7, 63.4, 62.2, 43.0, 19.8; IR (film) 1768, 1684, 1352, 1221, 1005 cm^{-1}; HRMS (ESI+) *m/z* calc'd for $(M + H)^+$ $[C_{21}H_{20}N_2O_5+ H]^+$: 381.1445, found 381.1445.

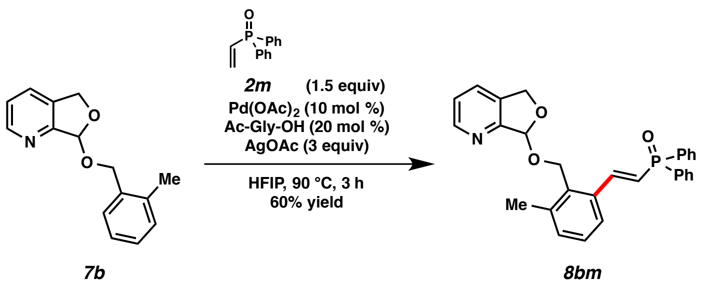

A suspension of acetal **7b** (24.1 mg, 0.100 mmol), diphenylvinylphosphine oxide[5] (**2m**, 34.2 mg, 0.150 mmol), $Pd(OAc)_2$ (2.2 mg, 10.0 µmol), *N*-acetylglycine (2.3 mg, 20.0 µmol), and AgOAc (50.1 mg, 0.300 mmol) in hexafluoroisopropanol (2.00 mL) in a 2-dram vial with a PTFE-lined cap was heated at 90 °C and stirred for 3 h. Upon completion, the reaction was cooled to ambient temperature, ethylenediamine (40 µL) and celite (70 mg) were added, and the resulting mixture was stirred overnight. The mixture was filtered through a plug of SiO_2 (5 mm x 20 mm, EtOAc → 9:1 EtOAc/MeOH eluent, ~20 mL), the filtrate was concentrated by rotary evaporation, and the resulting residue was dissolved in EtOAc and then filtered through a second plug of SiO_2 (5 mm x 20 mm, EtOAc → 9:1 EtOAc/MeOH eluent, ~20 mL). The filtrate was concentrated by rotary evaporation, and the crude residue was purified by flash column chromatography (19:1 EtOAc/MeOH eluent) to afford olefin **8bm** (28.2 mg, 60% yield, $R_f = 0.26$ in EtOAc) as a colorless oil.

Olefin 8bm: ^{1}H NMR (400 MHz, $CDCl_3$) δ 8.49 (d, J = 4.8 Hz, 1H), 7.93 (dd, $^4J_{H\text{-}P}$ = 19.8 Hz, $^3J_{H\text{-}H}$ = 17.3 Hz, 1H), 7.84 (dd, $^3J_{H\text{-}P}$ = 12.0 Hz, $^3J_{H\text{-}H}$ = 7.4 Hz, 2H), 7.75 (dd, $^3J_{H\text{-}P}$ = 12.0 Hz, $^3J_{H\text{-}H}$ = 7.4 Hz, 2H), 7.55 (d, J = 7.6 Hz, 1H), 7.54-7.38 (comp. m, 7H), 7.24-7.17 (comp. m, 3H), 6.81 (dd, $^4J_{H\text{-}P}$ = 23.2 Hz, $^3J_{H\text{-}H}$ = 17.3 Hz, 1H), 6.12 (d, J = 1.2 Hz, 1H), 5.30 (d, J = 13.2, 1.2 Hz, 1H), 5.03 (d, J = 13.2 Hz, 1H), 5.02 (d, J = 10.4 Hz, 1H), 4.76 (d, J = 10.4 Hz, 1H), 2.48 (s, 3H); ^{13}C NMR (100 MHz, $CDCl_3$) δ 157.5, 149.7, 146.3 (d, $^2J_{C\text{-}P}$ = 3.7 Hz), 139.1, 136.9, 136.7, 133.7, 133.45, 133.36 (d, $^1J_{C\text{-}P}$ = 104.5 Hz), 133.2 (d, $^1J_{C\text{-}P}$ = 105.4 Hz), 131.94, 131.87 (d, $^4J_{C\text{-}P}$ = 2.0 Hz), 131.8 (d, $^4J_{C\text{-}P}$ = 2.0 Hz), 131.7 (d, $^2J_{C\text{-}P}$ = 98.0 Hz), 131.6 (d, $^2J_{C\text{-}P}$ = 97.0 Hz), 129.9, 128.72 (d, $^3J_{C\text{-}P}$ = 11.6 Hz), 128.66 (d, $^3J_{C\text{-}P}$ = 12.0 Hz), 125.3, 123.6, 122.6 (d, $^2J_{C\text{-}P}$ = 3.7 Hz), 104.2, 70.7, 63.8, 19.8; IR (film) 1590, 1435, 1180, 1002, 725 cm^{-1}; HRMS (ESI+) *m/z* calc'd for $(M + H)^+$ $[C_{29}H_{26}NO_3P + H]^+$: 468.1723, found 468.1733.

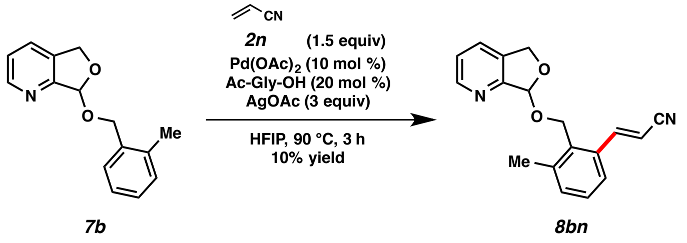

A suspension of acetal **7b** (24.1 mg, 0.100 mmol), acrylonitrile (**2n**, 9.8 µL, 0.150 mmol), $Pd(OAc)_2$ (2.2 mg, 10.0 µmol), *N*-acetylglycine (2.3 mg, 20.0 µmol), and AgOAc (50.1 mg, 0.300 mmol) in hexafluoroisopropanol (2.00 mL) in a 2-dram vial with a PTFE-lined cap was heated at 90 °C and

stirred for 3 h. Upon completion, the reaction was cooled to ambient temperature, ethylenediamine (40 µL) and celite (70 mg) were added, and the resulting mixture was stirred overnight. The mixture was filtered through a plug of SiO_2 (5 mm x 20 mm, EtOAc eluent, ~20 mL), the filtrate was concentrated by rotary evaporation, and the resulting residue was dissolved in EtOAc and then filtered through a second plug of SiO_2 (5 mm x 20 mm, EtOAc eluent, ~20 mL). The filtrate was concentrated by rotary evaporation, and the crude residue was purified by preparatory plate chromatography (1:1 hexanes/EtOAc eluent) to afford acrylonitrile **8bn** (2.9 mg, 10% yield, R_f = 0.36 in 1:1 hexanes/EtOAc) as a colorless film.

Acrylonitrile 8bn: ^{1}H NMR (400 MHz, $CDCl_3$) δ 8.60 (d, *J* = 4.8 Hz, 1H), 7.97 (d, *J* = 16.6 Hz, 1H), 7.66 (d, *J* = 8.0 Hz, 1H), 7.33-7.19 (comp. m, 4H), 6.23 (s, 1H), 5.81 (d, *J* = 16.6 Hz, 1H), 5.34 (d, *J* = 13.2 Hz, 1H), 5.18 (d, *J* = 13.2 Hz, 1H), 4.89 (ABq, *J* = 10.8 Hz, Δν = 22.4 Hz, 2H), 2.49 (s, 3H); ^{13}C NMR (100 MHz, $CDCl_3$) δ 157.1, 150.1, 149.7, 139.2, 134.8, 133.9, 133.2, 133.1, 130.1, 128.9, 124.2, 123.9, 118.6, 104.4, 98.5, 70.9, 63.2, 19.8; IR (film) 2215, 1003, 911, 788, 731 cm^{-1}; HRMS (ESI+) *m/z* calc'd for $(M + H)^+$ $[C_{18}H_{16}N_2O_2 + H]^+$: 293.1285, found 293.1285.

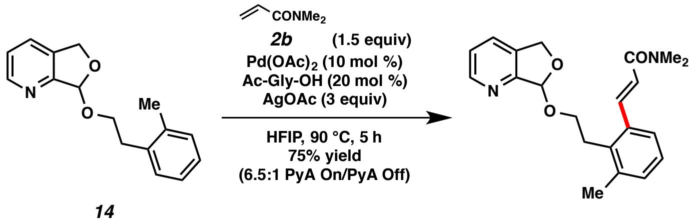

14 *15*

A suspension of acetal **14** (25.5 mg, 0.100 mmol), *N,N*-dimethylacrylamide (**2b**, 15.5 µL, 0.150 mmol), $Pd(OAc)_2$ (2.2 mg, 10.0 µmol), *N*-acetylglycine (2.3 mg, 20.0 µmol), and AgOAc (50.1 mg, 0.150 mmol) in hexafluoroisopropanol (2.00 mL) in a 2-dram vial with a PTFE-lined cap was heated at 90 °C and stirred for 5 h. Upon completion, the reaction was cooled to ambient temperature, ethylenediamine (40 µL) and celite (70 mg) were added, and the resulting mixture was stirred overnight. The mixture was filtered through a plug of SiO_2 (5 mm x 20 mm, EtOAc → 9:1 EtOAc/MeOH eluent, ~20 mL), the filtrate was concentrated by rotary evaporation, and the resulting residue was dissolved in EtOAc and then filtered through a second plug of SiO_2 (5 mm x 20 mm, EtOAc → 9:1 EtOAc/MeOH eluent, ~20 mL). The filtrate was concentrated by rotary evaporation, and the crude residue was purified by preparatory thin-layer chromatography (39:1 EtOAc/MeOH eluent) to afford olefin **15** (25.1 mg, 75% yield (1:0.15 PyA group attached/PyA group detached), R_f = 0.44 in 39:1 EtOAc/MeOH) as a colorless oil.

Olefin 15: ^{1}H NMR (400 MHz, $CDCl_3$) δ 8.57 (d, *J* = 4.8 Hz, 1H), 8.04 (d, *J* = 15.2 Hz, 0.15H), 7.99 (d, *J* = 15.0 Hz, 1H), 7.62 (d, *J* = 7.6 Hz, 1H), 7.38 (d, *J* = 7.2 Hz, 0.15H), 7.35 (d, *J* = 7.2 Hz, 1H), 7.26 (dd, *J* = 7.6, 4.8 Hz, 1H), 7.18-7.08 (comp. m, 2.30H), 6.75 (d, *J* = 15.2 Hz, 0.15H), 6.72 (d, *J* = 15.0 Hz, 1H), 6.14 (d, *J* = 1.6 Hz, 1H), 5.23 (dd, *J* = 13.2, 1.6 Hz, 1H), 5.04 (d, *J* = 13.2 Hz, 1H), 3.89-3.73 (comp. m, 2H), 3.72 (t, *J* = 7.6 Hz, 0.30H), 3.20-3.25 (comp. m, 2.30H), 3.14 (app. s, 1.45H), 3.05 (app. s, 1.45H), 2.37 (s, 0.45H), 2.36 (s, 3H); ^{13}C NMR (100 MHz, $CDCl_3$) δ 166.8, 166.6, 157.4, 149.7, 141.0, 140.4, 137.63, 137.59, 136.0, 135.6, 135.5, 135.2, 133.1, 131.5, 131.3, 129.8, 126.51, 126.48, 124.7, 124.5, 123.4, 120.3, 119.5, 104.8, 70.3, 67.4, 62.2, 37.5, 37.4, 35.92, 35.86, 32.8, 30.2, 20.1, 20.0; IR (film) 1642, 1595, 1005, 910, 741 cm^{-1}; HRMS (ESI+) *m/z* calc'd for $(M + H)^+$ $[C_{21}H_{24}N_2O_3 + H]^+$: 353.1860, found 353.1858.

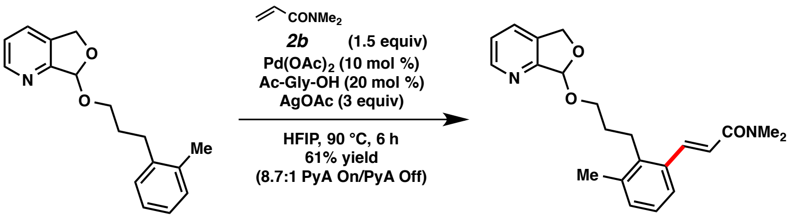

18 ***19***

A suspension of acetal **18** (26.9 mg, 0.100 mmol), *N,N*-dimethylacrylamide (**2b**, 15.5 µL, 0.150 mmol), $Pd(OAc)_2$ (2.2 mg, 10.0 µmol), *N*-acetylglycine (2.3 mg, 20.0 µmol), and AgOAc (50.1 mg, 0.150 mmol) in hexafluoroisopropanol (2.00 mL) in a 2-dram vial with a PTFE-lined cap was heated at 90 °C and stirred for 6 h. Upon completion, the reaction was cooled to ambient temperature, ethylenediamine (40 µL) and celite (70 mg) were added, and the resulting mixture was stirred overnight. The mixture was filtered through a plug of SiO_2 (5 mm x 20 mm, EtOAc → 9:1 EtOAc/MeOH eluent, ~20 mL), the filtrate was concentrated by rotary evaporation, and the resulting residue was dissolved in EtOAc and then filtered through a second plug of SiO_2 (5 mm x 20 mm, EtOAc → 9:1 EtOAc/MeOH eluent, ~20 mL). The filtrate was concentrated by rotary evaporation, and the crude residue was purified by preparatory thin-layer chromatography (39:1 EtOAc/MeOH eluent) to afford olefin **19** (21.6 mg, 61% yield (1:0.11 PyA group attached/PyA group detached), R_f = 0.52 in 39:1 EtOAc/MeOH) as a colorless oil.

Olefin 19: ^{1}H NMR (400 MHz, $CDCl_3$) δ 8.58 (d, *J* = 4.8 Hz, 1H), 8.06 (d, *J* = 15.0 Hz, 0.11H), 7.97 (d, *J* = 15.2 Hz, 1H), 7.63 (d, *J* = 8.0 Hz, 1H), 7.39 (d, *J* = 7.6 Hz, 0.11H), 7.35 (d, *J* = 7.6 Hz, 1H), 7.27 (dd, *J* = 8.0, 4.8 Hz, 1H), 7.17-7.06 (comp. m, 2.22H), 6.74 (d, *J* = 15.0 Hz, 0.11), 6.72 (d, *J* = 15.2 Hz, 1H), 6.14 (s, 1H), 5.25 (d, *J* = 13.4 Hz, 1H), 5.07 (d, *J* = 13.4 Hz, 1H), 3.90-3.71 (comp. m, 2H), 3.68 (t, *J* = 6.0 Hz, 0.22H), 3.17 (s, 0.33H), 3.14 (s, 3H), 3.06 (s, 0.33H), 3.05 (s, 3H), 2.88 (t, *J* = 8.8 Hz, 0.22H), 2.84 (t, *J* = 8.0 Hz, 2H), 1.92-1.80 (comp. m, 2H), 1.79-1.70 (comp. m, 0.22H)**;** ^{13}C NMR (100 MHz, $CDCl_3$) δ 166.9, 157.6, 140.9, 139.9, 137.1, 134.9, 137.1, 134.9, 105.2, 70.5, 68.5, 37.6, 36.0, 30.6, 26.0, 20.0; IR (film) 2924, 1647, 1600, 1004, 727 cm^{-1}; HRMS (ESI+) *m/z* calc'd for $(M + H)^+$ $[C_{22}H_{26}N_2O_3 + H]^+$: 367.2016, found 367.2016.

Note: The mixture of compounds with the PyA group attached and detached mixture were not separable by chromatography, but the mixture could be treated under acetylation conditions. The resulting acetate could thus be removed to provide an analytically pure sample of compound **19**.

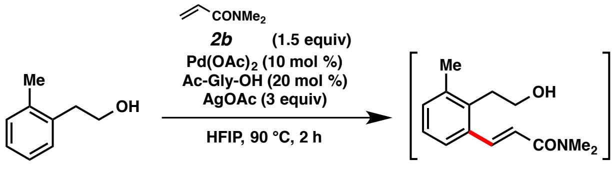

12 ***13***
not observed

A suspension of alcohol **12** (13.6 mg, 0.100 mmol), *N,N*-dimethylacrylamide (**2b**, 15.5 µL, 0.150 mmol), $Pd(OAc)_2$ (2.2 mg, 10.0 µmol), *N*-acetylglycine (2.3 mg, 20.0 µmol), and AgOAc (50.1 mg, 0.300 mmol) in hexafluoroisopropanol (2.00 mL) in a 2-dram vial with a PTFE-lined cap was heated at 90 °C and stirred for 2 h. Upon completion, the reaction was cooled to ambient temperature, ethylenediamine (40 µL) and celite (70 mg) were added, and the resulting mixture was stirred

overnight. The mixture was filtered through a plug of SiO_2 (5 mm x 20 mm, MeOH eluent, ~20 mL), the filtrate was concentrated by rotary evaporation, and the resulting residue was dissolved in EtOAc and then filtered through a second plug of SiO_2 (5 mm x 20 mm, MeOH eluent, ~20 mL). The filtrate was concentrated by rotary evaporation, and olefin **13** was not observed by NMR in the crude residue.

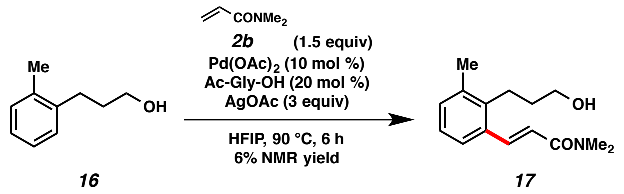

A suspension of alcohol **16** (15.0 mg, 0.100 mmol), *N,N*-dimethylacrylamide (**2b**, 15.5 µL, 0.150 mmol), $Pd(OAc)_2$ (2.2 mg, 10.0 µmol), *N*-acetylglycine (2.3 mg, 20.0 µmol), and AgOAc (50.1 mg, 0.300 mmol) in hexafluoroisopropanol (2.00 mL) in a 2-dram vial with a PTFE-lined cap was heated at 90 °C and stirred for 6 h. Upon completion, the reaction was cooled to ambient temperature, ethylenediamine (40 µL) and celite (70 mg) were added, and the resulting mixture was stirred overnight. The mixture was filtered through a plug of SiO_2 (5 mm x 20 mm, MeOH eluent, ~20 mL), the filtrate was concentrated by rotary evaporation, and the resulting residue was dissolved in EtOAc and then filtered through a second plug of SiO_2 (5 mm x 20 mm, MeOH eluent, ~20 mL). The filtrate was concentrated by rotary evaporation, and olefin **17** was observed in small quantities (6% NMR yield) in the crude residue.

Olefin 17: Representative peaks in ^{1}H NMR (400 MHz, $CDCl_3$) δ 8.07 (d, J = 15.0 Hz, 1H), 6.75 (d, J = 15.0 Hz, 1H).

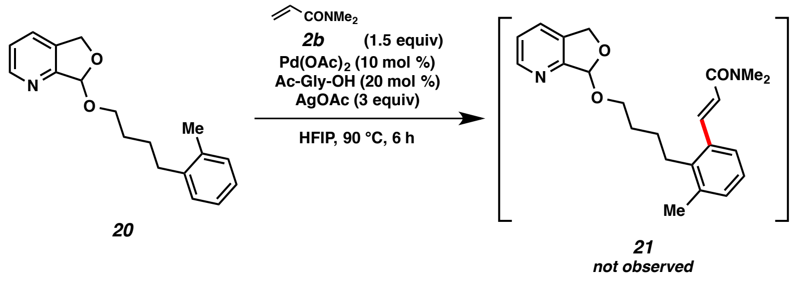

A suspension of acetal **20** (28.4 mg, 0.100 mmol), *N,N*-dimethylacrylamide (**2b**, 15.5 µL, 0.150 mmol), $Pd(OAc)_2$ (2.2 mg, 10.0 µmol), *N*-acetylglycine (2.3 mg, 20.0 µmol), and AgOAc (50.1 mg, 0.300 mmol) in hexafluoroisopropanol (2.00 mL) in a 2-dram vial with a PTFE-lined cap was heated at 90 °C and stirred for 4.5 h. Upon completion, the reaction was cooled to ambient temperature, ethylenediamine (40 µL) and celite (70 mg) were added, and the resulting mixture was stirred overnight. The mixture was filtered through a plug of SiO_2 (5 mm x 20 mm, MeOH eluent, ~20 mL), the filtrate was concentrated by rotary evaporation, and the resulting residue was dissolved in EtOAc and then filtered through a second plug of SiO_2 (5 mm x 20 mm, MeOH eluent, ~20 mL). The filtrate was concentrated by rotary evaporation, and olefin **21** was not observed by NMR in the crude residue.

Olefination Optimization Studies

Table S1. Screening of Oxidants.[a]

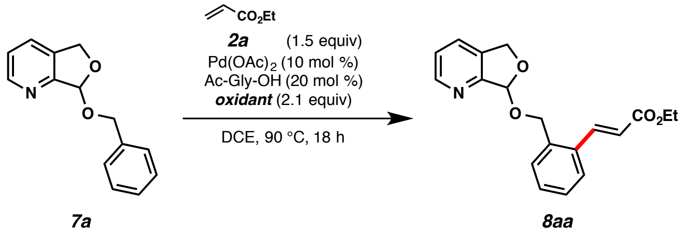

Entry	Oxidant	rsm (%)[b]	yield (mono) (%)[b]	yield (di) (%)[b]
**1**		**89**	**11**	**0**
**2**	**AgOAc**	**35**	**48**	**7**
**3**	**AgOPiv**	**49**	**10**	**0**
**4**	**AgOTfa**	**31**	**0**	**0**
**5**	**AgOTf**	**0**	**0**	**0**
**6**[c]	**Ag_2CO_3**	**57**	**32**	**trace**
**7**	**O_2 (1 atm)**	**53**	**19**	**0**
**8**[d]	**O_2 (1 atm)/$H_4[PMo_{11}VO_{40}]$•$32H_2O$**	**44**	**13**	**0**
**9**	**$Cu(OAc)_2$**	**25**	**26**	**0**
**10**	**benzoquinone**	**78**	**9**	**0**
**11**[e]	***t*-BuOOH (5 M in CH_2Cl_2)**	**36**	**10**	**0**
**12**[e]	**$K_2S_2O_8$**	**0**	**0**	**0**

a) Unless otherwise noted, the reaction conditions were as follows: acetal **7a** (0.100 mmol), ethyl acrylate **2a** (1.5 equiv), $Pd(OAc)_2$ (10 mol %), Ac-Gly-OH (20 mol %), ***oxidant*** (2.1 equiv), and DCE (1.0 mL) were heated at 90 °C for 18 h. b) Yield was determined by ^1H NMR analysis of the unpurified reaction mixture using 1-octene as an internal standard. c) 1.05 equiv of Ag_2CO_3 was used instead of 2.1 equiv. d) 1 mol % of $H_4[PMo_{11}VO_{40}]$•$32H_2O$ was used. e) 2.0 equiv of oxidant was used instead of 2.1 equiv.

Table S2. Screening of Temperature.[a]

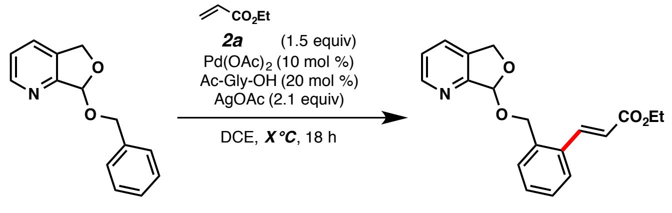

Entry	Temperature (°C)	rsm (%)[b]	yield (mono) (%)[b]	yield (di) (%)[b]
1	70	81	13	0
2	80	72	22	0
3	90	35	48	7
4	100	6	13	2

a) The reaction conditions were as follows: acetal **7a** (0.100 mmol), ethyl acrylate **2a** (1.5 equiv), $Pd(OAc)_2$ (10 mol %), Ac-Gly-OH (20 mol %), AgOAc (2.1 equiv), and DCE (1.0 mL) were heated at ***X* °C** for 18 h. b) Yield was determined by ^{1}H NMR analysis of the unpurified reaction mixture using 1-octene as an internal standard.

Table S3. Screening of Palladium Catalysts.[a]

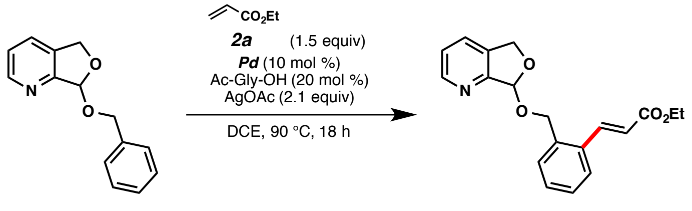

Entry	Pd	rsm (%)[b]	yield (mono) (%)[b]	yield (di) (%)[b]
1	–	100	0	0
2	$Pd(OAc)_2$	35	48	7
3	$Pd(OPiv)_2$	71	10	0
4	$Pd(OTf)_2$	47	35	7
5	$PdCl_2$	80	20	0
6	$PdCl_2(PPh_3)_2$	92	3	0
7	$PdCl_2(MeCN)_2$	72	27	trace
8	$Pd(PPh_3)_4$	85	0	0
9	$Pd_2(dba)_3$	80	20	0

a) The reaction conditions were as follows: acetal **7a** (0.1 mmol), ethyl acrylate **2a** (1.5 equiv), ***Pd*** (10 mol %), Ac-Gly-OH (20 mol %), AgOAc (2.1 equiv), and DCE (1.0 mL) were heated at 90 °C for 18 h. b) Yield was determined by ^{1}H NMR analysis of the unpurified reaction mixture using 1-octene as an internal standard.

Table S4. Screening of Ligands.[a]

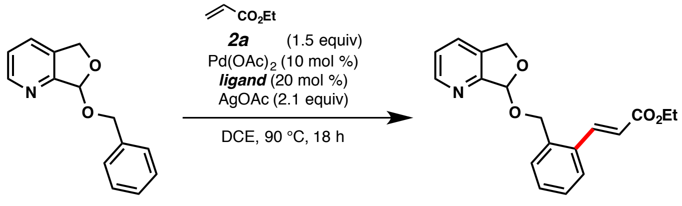

7a *8aa*

Entry	Pd	rsm (%)[b]	yield (mono) (%)[b]	yield (di) (%)[b]
1	–	77	20	0
2	Ac-Gly-OH	35	48	7
3	Ac-Ala-OH	47	38	5
4	Ac-Val-OH	69	21	trace
5	Ac-Leu-OH	65	29	trace
6	Ac-Ile-OH	66	18	trace
7	Ac-*tert*-Leu-OH	86	11	trace
8	Boc-Gly-OH	59	11	0
9	Fmoc-Gly-OH	65	8	0
10	For-Gly-OH	61	16	0
11	Tfa-Gly-OH	74	7	0
12[c]	Ac-Gly-OH	40	34	trace
13	Ac-β-Ala-OH	59	16	0
14	Boc-Phe-OH	53	13	0
15	pyroglutamic acid	65	5	0
16	$(BzNHCH_2)_2$	71	trace	0
17	2-hydroxyacetanilide	61	18	0
18[d]	8-acetamidoquinoline	94	trace	0
19[d]	triazabicyclodecene	82	8	0

a) Unless otherwise noted, the reaction conditions were as follows: acetal **7a** (0.1 mmol), ethyl acrylate **2a** (1.5 equiv), $Pd(OAc)_2$ (10 mol %), ***ligand*** (20 mol %), AgOAc (2.1 equiv), and DCE (1.0 mL) were heated at 90 °C for 18 h. b) Yield was determined by ^{1}H NMR analysis of the unpurified reaction mixture using 1-octene as an internal standard. c) 40 mol % of Ac-Gly-OH was used instead of 20 mol %. d) 10 mol % of the ligand was used instead of 20 mol %.

Note: All ligands in the above screen are known compounds and are commercially available.

Table S5. Screening of Concentration.[a]

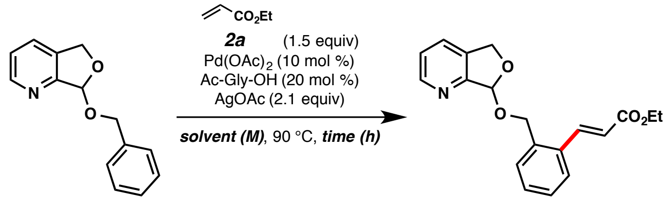

Entry	Solvent	Concentration (M)	Time (h)	rsm (%)[b]	yield (mono) (%)[b]	yield (di) (%)[b]
1	DCE	0.025	18	29	41	8
2	DCE	0.050	18	25	38	8
3	DCE	0.100	18	35	48	7
4	DCE	0.250	18	51	25	1
5	HFIP	0.025	3	10	33	18
6	HFIP	0.050	3	10	53	16
7	HFIP	0.100	3	15	34	9
8	HFIP	0.250	3	18	30	4

a) The reaction conditions were as follows: acetal **7a** (0.100 mmol), ethyl acrylate **2a** (1.5 equiv), $Pd(OAc)_2$ (10 mol %), Ac-Gly-OH (20 mol %), AgOAc (2.1 equiv), and ***DCE*** or ***HFIP*** (***X*** mL) were heated at 90 °C for either 18 or 3 h. b) Yield was determined by ^1H NMR analysis of the unpurified reaction mixture using 1-octene as an internal standard.

Table S6. Screening of Base Additive.[a]

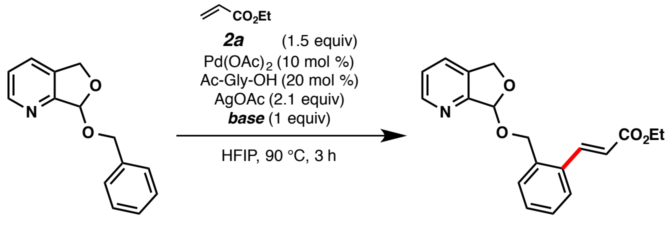

Entry	base	rsm (%)[b]	yield (mono) (%)[b]	yield (di) (%)[b]
1	–	10	53	16
2	NaOAc	28	43	7
3	Na_2CO_3	24	46	12
4	NaOTs	15	40	14
7	4-Me-di-*t*-Bupyridine	30	41	12

a) The reaction conditions were as follows: acetal **7a** (0.100 mmol), ethyl acrylate **2a** (1.5 equiv), $Pd(OAc)_2$ (10 mol %), Ac-Gly-OH (20 mol %), AgOAc (2.1 equiv), ***base*** (1 equiv), and HFIP (2.0 mL) were heated at 90 °C for 18 h. b) Yield was determined by ^1H NMR analysis of the unpurified reaction mixture using 1-octene as an internal standard.

Table S7. Screening of Oxidant Loading.[a]

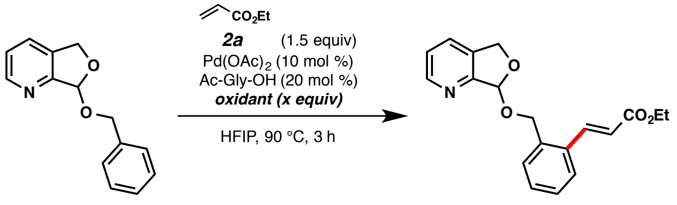

Entry	Oxidant (*x* equiv)	rsm (%)[b]	yield (mono) (%)[b]	yield (di) (%)[b]
1	0	31	5	0
2	AgOAc, 2.1	10	53	16
3	AgOAc, 3.0	10	55	24
4	AgOAc, 4.0	8	30	40
5	Ag_2CO_3, 1.05	53	15	0
6	Ag_2CO_3, 1.5	57	17	0
7	Ag_2CO_3, 2.0	64	22	0

a) The reaction conditions were as follows: acetal **7a** (0.100 mmol), ethyl acrylate **2a** (1.5 equiv), $Pd(OAc)_2$ (10 mol %), Ac-Gly-OH (20 mol %), ***Ag*** (***X*** equiv), and HFIP (2.0 mL) were heated at 90 °C for 3 h. b) Yield was determined by ^1H NMR analysis of the unpurified reaction mixture using 1-octene as an internal standard.

Table S8. Screening of Olefin Loading.[a]

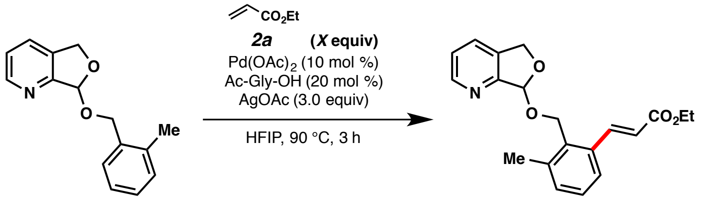

Entry	Olefin (*x* equiv)	rsm (%)[b]	yield (%)[b]
1	1.1	trace	50
2	1.5	5	55
3	2.0	trace	44
4	2.5	trace	43
5	3.0	trace	47

a) The reaction conditions were as follows: acetal **7b** (0.100 mmol), ethyl acrylate **2a** (***X*** equiv), $Pd(OAc)_2$ (10 mol %), Ac-Gly-OH (20 mol %), AgOAc (3.0 equiv), and HFIP (2.0 mL) were heated at 90 °C for 3 h. b) Yield was determined by ^1H NMR analysis of the unpurified reaction mixture using 1-octene as an internal standard.

Table S9. Reaction Workup Analysis.[a]

Note: We based our approach on the findings of Wang and coworkers at Merck, who had investigated the combination of chelating agents with solid adsorbents for the scavenging of palladium.[6]

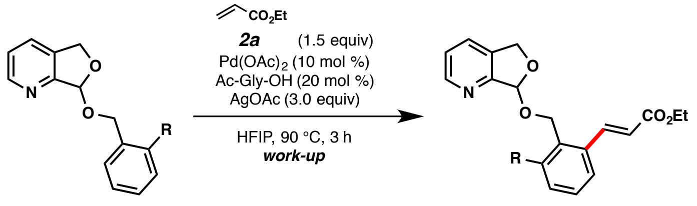

7a **(R = H)**
7b **(R = Me)**

8aa **(R = H)**
8ba **(R = Me)**

Entry	R	Workup	rsm (%)[b]	yield (mono) (%)[b]	yield (di) (%)[b]
1	H	–	10	55	24
2[c]	H	w/ ethylenediamine and celite	7	52	31
3	Me	–	5	55	–
4[c]	Me	w/ ethylenediamine and celite	6	74	–
5	Me	w/ ethylenediamine/SiO_2	12	69	–
6	Me	w/ ethylenediamine/activated C	4	63	–
7	Me	w/ DPPE/SiO_2	0	57	–
8	Me	w/ Ac-Cys-OH/SiO_2	0	61	–

a) The reaction conditions were as follows: acetal **7a** or **7b** (0.100 mmol), ethyl acrylate **2a** (1.5 equiv), $Pd(OAc)_2$ (10 mol %), Ac-Gly-OH (20 mol %), AgOAc (3.0 equiv), and HFIP (2.0 mL) were heated at 90 °C for 3 h. b) Yield was determined by ^{1}H NMR analysis of the unpurified reaction mixture using 1-octene as an internal standard. c) The reaction was cooled to ambient temperature, ethylenediamine (150 wt. % relative to acetal) and celite (300 wt. % relative to acetal) were added, and the mixture was stirred for 18 h.

The HFIP acetal (**S3**) was observed as a side product in some reactions during the optimization screen. It is observed more prominently with longer reaction times and when acid additives are used.

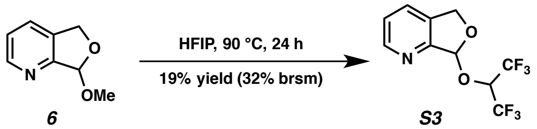

6 ***S3***

A solution of methyl acetal **6** (30.2 mg, 0.200 mmol) in hexafluoroisopropanol (2.00 mL) was heated at 90 °C and stirred for 24 h. The solution was cooled to ambient temperature and concentrated by rotary evaporation. The crude residue was purified by column chromatography (7:3 hexanes/EtOAc eluent) to afford hexafluoroisopropyl acetal **S3** (10.8 mg, 19% yield (32% brsm), R_f = 0.34 in 7:3 hexanes/EtOAc) as a white solid.

Hexafluoroisopropyl acetal S3: ^{1}H NMR (400 MHz, $CDCl_3$) δ 8.63 (d, J = 4.8 Hz, 1H), 7.70 (d, J = 7.6 Hz, 1H), 7.35 (dd, J = 7.6, 4.8 Hz, 1H), 6.30 (s, 1H), 5.27 (d, J = 13.2 Hz, 1H), 5.13 (d, J = 13.2 Hz, 1H), 4.77 (septet, $^3J_{H\text{-}F}$ = 6.0 Hz, 1H); ^{13}C NMR (100 MHz, $CDCl_3$) δ 155.9, 150.3, 133.1, 130.1, 124.4, 105.2, 71.3; ^{19}F NMR (376 MHz, $CDCl_3$) δ -74.4 (dq, $^4J_{F\text{-}F}$ = 8.6 Hz, $^3J_{H\text{-}F}$ = 6.0 Hz, 3F), -75.1 (dq, $^4J_{F\text{-}F}$ = 8.6, $^3J_{H\text{-}F}$ = 6.0 Hz, 3F); IR (film) 1287, 1218, 1187, 1100, 1050, 959 cm^{-1}; HRMS (DART+) *m/z* calc'd for $(M + H)^+$ $[C_{10}H_7F_6NO_2 + H]^+$: 288.0453, found 288.0454.

Analysis of Benzylic Alcohol Protecting Groups and Directing Groups

Functionalizations of Protected Benzyl Alcohols

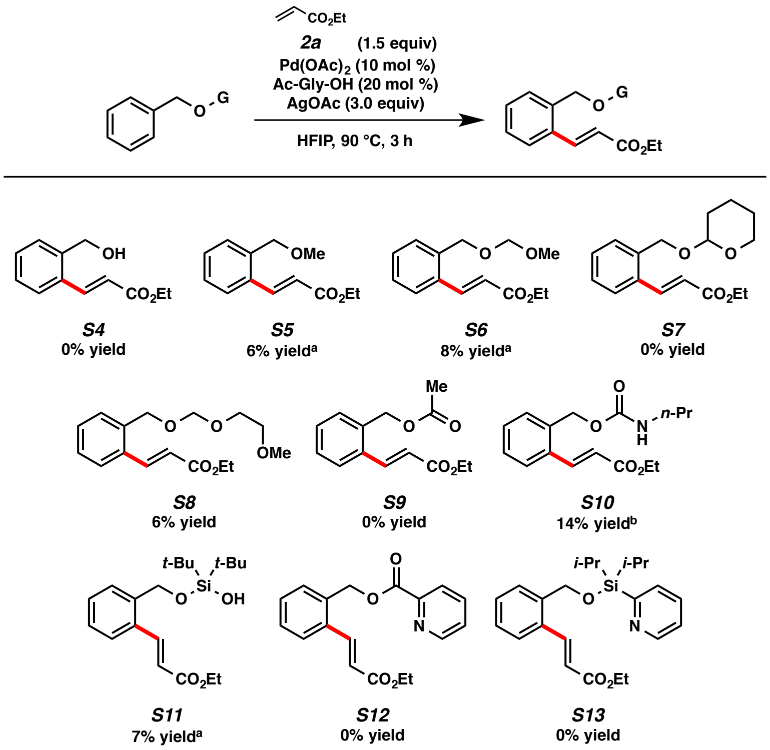

Reactions were preformed according to the general procedure. All values are NMR yields obtained using 1-octene as an internal standard. a) This value corresponds to the *ortho* contribution of a mixture of olefinated products. b) Increasing the reaction time from 3 to 6 h increased the yield to 16%.

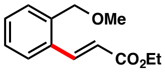

Olefin S5: Representative peaks in ^{1}H NMR (300 MHz, $CDCl_3$) δ 8.03 (d, J = 15.9 Hz, 1H), 6.40 (d, J = 15.9 Hz, 1H).

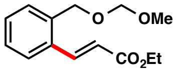

Olefin S6: Representative peaks in ^{1}H NMR (300 MHz, $CDCl_3$) δ 8.03 (d, J = 15.9 Hz, 1H), 6.40 (d, J = 15.9 Hz, 1H).

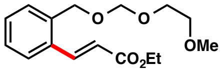

Olefin S8: Representative peak in ^{1}H NMR (300 MHz, $CDCl_3$) δ 8.01 (d, J = 16.2 Hz, 1H).

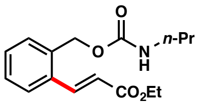

Olefin S10: Representative peaks in ^{1}H NMR (300 MHz, $CDCl_3$) δ 7.99 (d, *J* = 15.9 Hz, 1H), 6.38 (d, *J* = 15.9 Hz, 1H).

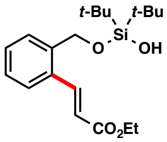

Olefin S11: Representative peak in ^{1}H NMR (300 MHz, $CDCl_3$) δ 8.13 (d, *J* = 15.9 Hz, 1H).

Synthesis of Protected Benzyl Alcohol Substrates

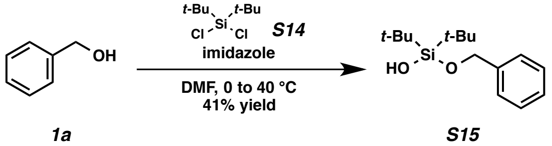

To a stirring solution of benzyl alcohol (**1a**, 248 mg, 2.29 mmol) and imidazole (408 mg, 6.00 mmol) in DMF (10.0 mL) at 0 °C was added di-*t*-butyl dichlorosilane (**S14**, 422 mg, 1.98 mmol). The solution was warmed to 40 °C and stirred for 12 h. Upon completion, the reaction mixture was cooled to 0 °C and sat. aq. $NaHCO_3$ (10 mL) was added, and the mixture was extracted with Et_2O (3 x 50 mL). The combined organic layers were washed with brine (50 mL), dried over $MgSO_4$, and concentrated by rotary evaporation. The crude silanol was purified by flash column chromatography (9:1 hexanes/EtOAc eluent) to afford silanol **S15** (214 mg, 41% yield, R_f = 0.33 in 9:1 hexanes/EtOAc) as a colorless oil.

Silanol S15: ^{1}H NMR (400 MHz, $CDCl_3$) δ 7.39-7.31 (comp. m, 4H), 7.28-7.22 (comp m, 1H), 4.94 (s, 2H), 1.95 (s, 1H), 1.06 (s, 18H); ^{13}C NMR (100 MHz, $CDCl_3$) δ 141.4, 128.4, 127.1, 126.3, 65.4, 27.6, 20.7; IR (film) 2965, 2933, 2890, 2858, 826 cm^{-1}; HRMS (ESI+) *m/z* calc'd for $(M + Na)^+$ $[C_{15}H_{26}O_2Si + Na]^+$: 289.1594, found 289.1593.

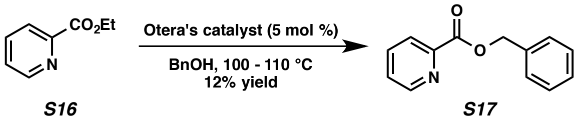

A solution of ethyl 2-picolinate (**S16**, 398 mg, 2.63 mmol) and Otera's catalyst[7] (159 mg, 0.133 mmol) in benzyl alcohol (13.2 mL) was heated at 100-110 °C and stirred for 14 h. The mixture was cooled to ambient temperature and poured into EtOAc (100 mL). The solution was extracted with 1 M aq. HCl (3 x 20 mL), the organic layer was discarded, and the combined aqueous layers were neutralized with sat. aq. Na_2CO_3 (100 mL). The aqueous mixture was extracted with EtOAc (20 mL). The organic layer was dried over Na_2SO_4 and concentrated by rotary evaporation. The resulting oil was purified by flash column chromatography (7:3 hexanes/EtOAc eluent) to afford benzyl 2-picolinate (**S17**, 67.6 mg, 12% yield, R_f = 0.25 in 7:3 hexanes/EtOAc) as a colorless oil.

Benzyl picolinate S17: ^{1}H NMR (400 MHz, $CDCl_3$) δ 8.76 (d, *J* = 4.8 Hz, 1H) 8.13 (d, *J* = 8.0 Hz, 1H), 7.82 (tt, *J* = 8.0, 1.6 Hz, 1H), 7.51-7.44 (comp. m, 3H), 7.41-7.30 (comp. m, 3H), 5.46 (s, 2H);

^{13}C NMR (100 MHz, $CDCl_3$) δ 165.1, 150.1, 148.2, 137.1, 135.8, 128.7, 128.5, 127.0, 125.4, 67.6; IR (film) 1737, 1717, 1584, 1455, 1302, 1290, 1243, 1122 cm^{-1}; LRMS (ESI+) *m/z* calc'd for $(M + H)^+$ $[C_{13}H_{11}NO_2 + H]^+$: 214.1, found 214.1.

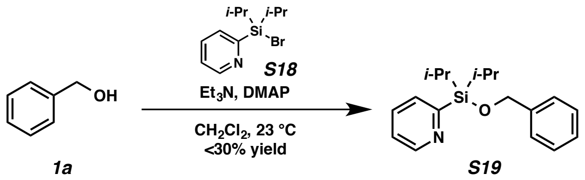

To a stirring solution of pyridyl silane **S18**[8] (250 mg, 1.29 mmol) in CH_2Cl_2 (5.70 mL) in an ambient temperature bath was added *N*-bromosuccinimide (230 mg, 1.29 mmol) in 4 portions over 1 min. Upon completion of the reaction by TLC (within 30 min), the solution was transferred by cannula to a solution of benzyl alcohol (**1a**, 0.147 mL, 1.42 mmol), triethylamine (0.198 mL, 1.42 mmol), and DMAP (15.8 mg, 0.129 mmol) in CH_2Cl_2 (5.70 mL), and the mixture was stirred at room temperature for 2 h. Upon completion, water (5 mL) was added, and the mixture was extracted with CH_2Cl_2 (2 x 10 mL). The combined organic layers were washed with brine (5 mL), dried over $MgSO_4$, and concentrated by rotary evaporation. The crude mixture was purified by flash column chromatography (9:1 hexanes/EtOAc eluent) to afford semi-pure silyl ether **S19** (117 mg, <30% yield, R_f = 0.22 in 9:1 hexanes/EtOAc) as a beige oil. Silyl ether **S19** decomposes on SiO_2 and was found to decompose in hexafluoroisopropanol when heated to 90 °C.

Silyl ether S19: ^1H NMR (400 MHz, $CDCl_3$) δ 8.79 (d, J = 4.8 Hz, 1H), 7.62 (d, J = 7.6 Hz, 1H), 7.58 (t, J = 7.6 Hz, 1H), 7.42 (d, J = 7.2 Hz, 2H), 7.37 (t, J = 7.2 Hz, 2H), 7.27 (t, J = 7.2 Hz, 1H), 7.21 (dd, J = 7.6, 4.8 Hz, 1H), 4.98 (s, 2H), 1.46 (septet, J = 7.2 Hz, 2H), 1.12 (d, J = 7.2 Hz, 6H), 1.07 (d, J = 7.2 Hz, 6H); LRMS (ESI+) *m/z* calc'd for $(M + H)^+$ $[C_{18}H_{25}NOSi + H]^+$: 300.2, found 300.2.

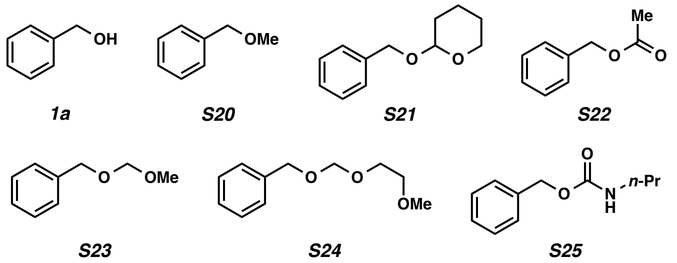

Benzyl alcohol (**1a)**, ether **S20**, acetal **S21**, and ester **S22** are all commercially available. Acetal **S23**[9], acetal **S24**[9], and carbamate **S25**[10] can be synthesized by known procedures.

The Scaffold as a Protecting Group

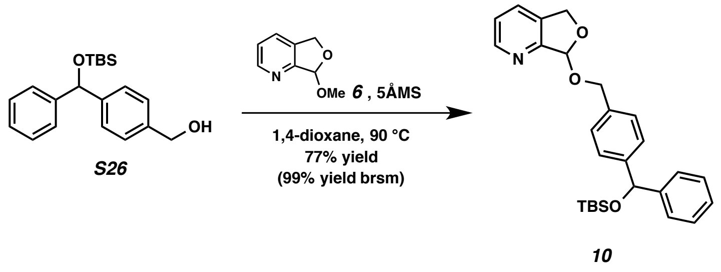

In a 2-dram vial with a PTFE-lined cap, a suspension of alcohol **S26** (98.6 mg, 0.300 mmol), methyl acetal **6** (47.6 mg, 0.315 mmol), and 5Å molecular sieves (375 mg) in 1,4-dioxane (0.476 mL) was heated at 90 °C (without stirring) for 19 h. Upon completion, the reaction was cooled to ambient temperature, and EtOAc (~5.0 mL) and celite (~500 mg) were added to the mixture. This suspension was filtered through a pad of celite (65 mm x 10 mm) with 9:1 EtOAc/MeOH (150 mL), the filtrate concentrated by rotary evaporation, and the residue purified by flash column chromatography (7:3 hexanes/EtOAc eluent) to afford acetal **10** (104 mg, 77% yield (99% yield brsm), R_f = 0.34 in 7:3 hexanes/EtOAc) as a colorless oil.

Acetal 10: ^{1}H NMR (500 MHz, $CDCl_3$) δ 8.57 (d, *J* = 5.5 Hz, 1H), 7.61 (d, *J* = 7.5 Hz, 1H), 7.38-7.32 (comp. m, 6H), 7.29-7.23 (comp. m, 3H), 7.18 (t, *J* = 7.3 Hz, 1H), 6.22 (d, *J* = 2.0 Hz, 1H), 5.75 (s, 1H), 5.25 (dd, *J* = 13.0, 2.0 Hz, 1H), 5.08 (d, *J* = 13.0 Hz, 1H), 4.78 (ABq, *J* = 11.5 Hz, Δν = 36.6 Hz, 2H), 0.92 (s, 9H), -0.02 (s, 6H); ^{13}C NMR (125 MHz, $CDCl_3$) δ 157.6, 149.8, 145.3, 144.9, 136.2, 133.2, 129.9, 128.24, 128.22, 127.0, 126.4, 126.3, 123.6, 104.2, 76.6, 70.5, 70.1, 25.6, 18.4, -4.69, -4.74; IR (film) 1066, 1009, 863, 837, 775 cm^{-1}; HRMS (ESI+) *m/z* calc'd for $(M + H)^+$ $[C_{27}H_{33}NO_3Si + H]^+$: 448.2302, found 448.2302.

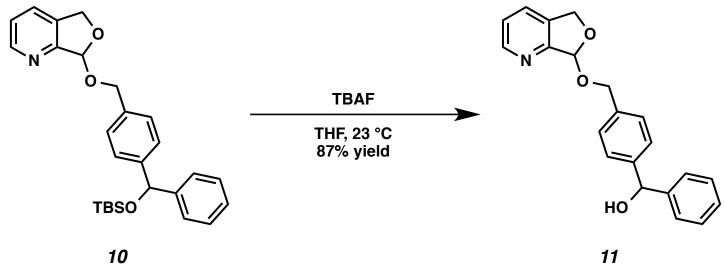

To a stirring solution of acetal **10** (60.4 mg, 0.135 mmol) in THF (0.450 mL) at 23 °C was added TBAF (0.270 mL, 1.0 M in THF, 0.270 mmol). After 3 h, water (10 mL) was added to the reaction mixture, and it was extracted with EtOAc (3 x 15 mL). The combined organic layers were washed with brine (10 mL), dried over $MgSO_4$, and concentrated by rotary evaporation. The crude alcohol was purified by flash column chromatography (1:1 hexanes/EtOAc eluent) to afford alcohol **11** (39.1 mg, 87% yield, R_f = 0.20 in 1:1 hexanes/EtOAc) as a white foam.

Alcohol 11: ^{1}H NMR (500 MHz, $CDCl_3$) δ 8.56 (d, *J* = 5.0 Hz, 1H), 7.62 (d, *J* = 7.5 Hz, 1H), 7.40 (d, *J* = 7.8 Hz, 2H), 7.35 (app. d, *J* = 8.0 Hz, 4H), 7.31 (t, *J* = 7.8 Hz, 2H), 7.28-7.22 (comp. m, 2H), 6.20 (s, 1H), 5.83 (s, 1H), 5.25 (d, *J* = 13.3 Hz, 1H), 5.08 (d, *J* = 13.3 Hz, 1H), 4.81 (ABq, *J* = 11.8 Hz, Δν = 33.8 Hz, 2H), 2.37 (s, 1H); ^{13}C NMR (125 MHz, $CDCl_3$) δ 157.6, 149.9, 143.9, 143.5, 137.0, 133.2, 129.9, 128.6, 128.5, 127.7, 126.7, 126.6, 123.7, 104.2, 76.2, 70.5, 70.1; IR (film) 3269, 1082, 1008, 793, 704 cm^{-1}; HRMS (ESI+) *m/z* calc'd for $(M + H)^+$ $[C_{21}H_{19}NO_3 + H]^+$: 334.1438, found 334.1439.

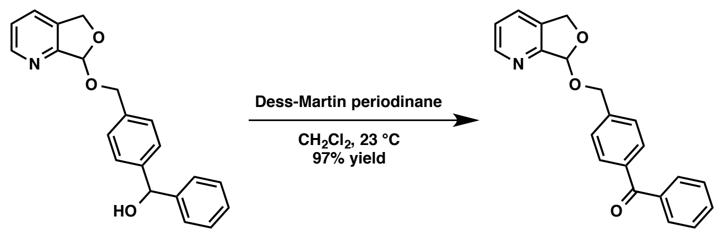

To a stirring solution of alcohol **11** (66.7 mg, 0.200 mmol) in CH_2Cl_2 (2.86 mL) at 23 °C was added Dess-Martin periodinane (89.1 mg, 0.210 mmol). After 1.5 h, water (20 mL) was added to the reaction mixture, and the mixture was extracted with CH_2Cl_2 (3 x 20 mL). The combined organic layers were washed with brine (10 mL), dried with $MgSO_4$, and concentrated by rotary evaporation. The crude oil was purified by flash column chromatography (1:1 hexanes/EtOAc) to afford ketone **7n** (64.1 mg, 97% yield, R_f = 0.36 in 1:1 hexanes/EtOAc) as a colorless oil that slowly transformed to a white solid.

Ketone 7n: ^{1}H NMR (400 MHz, $CDCl_3$) δ 8.60 (d, J = 4.8 Hz, 1H), 7.80-7.75 (comp. m, 4H), 7.65 (d, J = 8.0 Hz, 1H), 7.60-7.51 (comp. m, 3H), 7.46 (t, J = 7.6 Hz, 2H), 7.29 (dd, J = 8.0, 4.8 Hz, 1H), 6.26 (d, J = 1.6 Hz, 1H), 5.27 (dd, J = 13.2, 1.6 Hz, 1H), 5.11 (d, J = 13.2 Hz, 1H), 4.92 (ABq, J = 12.4 Hz, Δν = 22.5 Hz, 2H); ^{13}C NMR (100 MHz, $CDCl_3$) δ 196.5, 157.4, 150.0, 142.6, 137.8, 136.9, 133.2, 132.5, 130.4, 130.1, 130.0, 128.4, 127.7, 123.8, 104.5, 70.6, 69.6; IR (film) 1652, 1310, 1273, 2005, 704 cm^{-1}; HRMS (ESI+) *m/z* calc'd for $(M + H)^+$ $[C_{21}H_{17}NO_3 + H]^+$: 332.1281, found 332.1282.

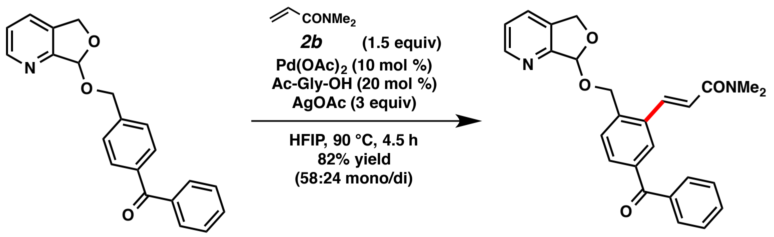

A suspension of acetal **7n** (33.1 mg, 0.100 mmol), *N,N*-dimethylacrylamide (**2b**, 15.5 µL, 0.150 mmol), $Pd(OAc)_2$ (2.2 mg, 10.0 µmol), *N*-acetylglycine (2.3 mg, 20.0 µmol), and AgOAc (50.1 mg, 0.300 mmol) in hexafluoroisopropanol (2.00 mL) in a 2-dram vial with a PTFE-lined cap was heated at 90 °C and stirred for 4.5 h. Upon completion, the reaction was cooled to ambient temperature, ethylenediamine (40 µL) and celite (70 mg) were added, and the resulting mixture was stirred overnight. The mixture was filtered through a plug of SiO_2 (5 mm x 20 mm, EtOAc → 1:1 EtOAc/MeOH eluent, ~20 mL), the filtrate was concentrated by rotary evaporation, and the resulting residue was dissolved in EtOAc and then filtered through a second plug of SiO_2 (5 mm x 20 mm, EtOAc → 1:1 EtOAc/MeOH eluent, ~20 mL). The filtrate was concentrated by rotary evaporation, and the crude residue was purified by flash column chromatography (EtOAc eluent) to afford monoolefin **8nb$_{mono}$** (24.9 mg, 58% yield, R_f = 0.26 in EtOAc) as a white solid. The column was washed with MeOH, the filtrate concentrated by rotary evaporation, and the residue was purified by preparatory plate chromatography (9:1 EtOAc/MeOH eluent) to afford diolefin **8nb$_{di}$** (12.8 mg, 24% yield, R_f = 0.15 in 9:1 EtOAc/MeOH) as a white film.

Monoolefin 8nb$_{mono}$: ^{1}H NMR (500 MHz, $CDCl_3$) δ 8.57 (d, J = 5.0 Hz, 1H), 8.00 (s, 1H), 7.97 (d, J = 15.6 Hz, 1H), 7.77 (d, J = 7.6 Hz, 2H), 7.70-7.56 (comp. m, 4H), 7.47 (t, J = 7.6 Hz, 2H), 7.28 (dd, J =

7.6, 5.0 Hz, 1H), 6.87 (d, *J* = 15.6 Hz, 1H), 6.28 (d, *J* = 1.2 Hz, 1H), 5.43 (dd, *J* = 13.2, 1.2 Hz, 1H), 5.15 (d, *J* = 13.2 Hz, 1H), 5.14 (d, *J* = 12.4 Hz, 1H), 4.89 (d, *J* = 12.4 Hz, 1H), 3.14 (s, 3H), 3.06 (s, 3H); ^{13}C NMR (125 MHz, $CDCl_3$) δ 196.3, 166.3, 157.3, 149.9, 140.7, 138.3, 137.5, 137.4, 135.0, 133.5, 132.8, 130.9, 130.2, 130.1, 129.2, 128.5, 128.0, 123.8, 121.4, 104.5, 70.8, 67.3, 37.7, 36.1; IR (film) 1652, 1594, 1394, 1005, 726 cm^{-1}; HRMS (ESI+) *m/z* calc'd for $(M + H)^+$ $[C_{26}H_{24}N_2O_4 + H]^+$: 429.1809, found 429.1818.

Diolefin 8nb$_{di}$: ^{1}H NMR (500 MHz, $CDCl_3$) δ 8.52 (d, *J* = 4.8 Hz, 1H), 8.10 (d, *J* = 15.2 Hz, 2H), 7.85 (s, 2H), 7.78 (d, *J* = 8.0 Hz, 2H), 7.65 (d, *J* = 8.0 Hz, 1H), 7.61 (t, *J* = 8.0 Hz, 1H), 7.49 (t, *J* = 8.0 Hz, 2H), 7.27-7.22 (comp. m, 1H), 6.86 (d, *J* = 15.2 Hz, 2H), 6.29 (s, 1H), 5.62 (d, *J* = 14.0 Hz, 1H), 5.21 (d, *J* = 14.0 Hz, 1H), 5.13 (d, *J* = 10.4 Hz, 1H), 4.92 (d, *J* = 10.4 Hz, 1H), 3.17 (s, 6H), 3.08 (s, 6H); ^{13}C NMR (125 MHz, $CDCl_3$) δ 196.0, 166.3, 157.3, 149.8, 139.3, 138.1, 138.0, 137.5, 137.1, 133.8, 133.2, 130.3, 130.1, 129.2, 128.7, 123.8, 123.1, 104.4, 71.1, 63.1, 37.8, 36.2; IR (film) 1649, 1612, 1397, 1008, 730 cm^{-1}; HRMS (ESI+) *m/z* calc'd for $(M + H)^+$ $[C_{31}H_{31}N_3O_5 + H]^+$: 526.2336, found 526.2337.

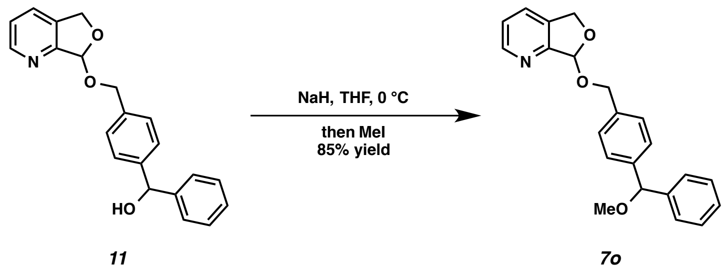

To a solution of alcohol **11** (39.1 mg, 0.117 mmol) in THF (1.17 mL) at 0 °C was added NaH (10.3 mg, 60% dispersion in mineral oil, 0.258 mmol), and the mixture was stirred at this temperature for 30 min. MeI (22 μL, 0.351 mmol) was added to the reaction mixture at 0 °C, and the mixture was stirred for 2 h. An additional portion of MeI (22 μL, 0.351 mmol) was then added to the reaction mixture at 0 °C, and the mixture was stirred for 2 h. Upon completion as determined by TLC, the reaction mixture was diluted with water (20 mL) and extracted with EtOAc (3 x 30 mL). The combined organic layers were washed with brine (15 mL), dried over $MgSO_4$, and concentrated by rotary evaporation. The crude ether was purified by flash column chromatography (7:3 hexanes/EtOAc) to afford methyl ether **7o** (34.7 mg, 85% yield, R_f = 0.16 in 7:3 hexanes/EtOAc) as a colorless oil.

Methyl ether 7o: ^{1}H NMR (400 MHz, $CDCl_3$) δ 8.57 (d, *J* = 4.5 Hz, 1H), 7.61 (d, *J* = 7.5 Hz, 1H), 7.40 (d, *J* = 8.0 Hz, 2H), 7.35-7.27 (comp. m, 6H), 7.25 (dd, *J* = 7.5, 4.5 Hz, 1H), 7.22 (t, *J* = 6.5 Hz, 1H), 6.21 (d, *J* = 2.0 Hz, 1H), 5.25 (d, *J* = 13.0 Hz, 1H), 5.23 (s, 1H), 5.07 (d, *J* = 13.0 Hz, 1H), 4.82 (ABq, *J* = 11.5 Hz, Δν = 34.9 Hz, 2H), 3.36 (s, 3H); ^{13}C NMR (100 MHz, $CDCl_3$) δ 157.6, 149.8, 142.1, 141.7, 136.8, 133.2, 129.9, 128.4, 127.5, 127.1, 126.9, 123.6, 104.1, 85.3, 70.5, 70.0, 57.1; IR (film) 1086, 1075, 1005, 789, 700 cm^{-1}; HRMS (ESI+) *m/z* calc'd for $(M + H)^+$ $[C_{22}H_{21}NO_3 + H]^+$: 348.1594, found 348.1594.

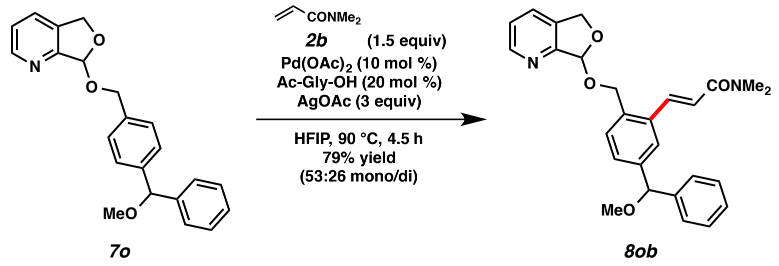

A suspension of acetal **7o** (34.7 mg, 99.9 µmol), *N,N*-dimethylacrylamide (**2b**, 15.4 µL, 0.150 mmol), $Pd(OAc)_2$ (2.2 mg, 9.99 µmol), *N*-acetylglycine (2.3 mg, 20.0 µmol), and AgOAc (50.0 mg, 0.300 mmol) in hexafluoroisopropanol (2.00 mL) in a 2-dram vial with a PTFE-lined cap was heated at 90 °C and stirred for 4.5 h. Upon completion, the reaction was cooled to ambient temperature, ethylenediamine (40 µL) and celite (70 mg) were added, and the resulting mixture was stirred overnight. The mixture was filtered through a plug of SiO_2 (5 mm x 20 mm, EtOAc → 1:1 EtOAc/MeOH eluent, ~20 mL), the filtrate was concentrated by rotary evaporation, and the resulting residue was dissolved in EtOAc and then filtered through a second plug of SiO_2 (5 mm x 20 mm, EtOAc → 1:1 EtOAc/MeOH eluent, ~20 mL). The solution was concentrated by rotary evaporation and the crude reaction mixture was purified by flash column chromatography (EtOAc eluent) to afford monoolefin **8ob**$_{\mathbf{mono}}$ (23.4 mg, 53% yield, R_f = 0.19 in EtOAc) as a white foam. The column was washed with MeOH, the filtrate concentrated by rotary evaporation, and the residue was purified by preparatory plate chromatography (9:1 EtOAc/MeOH eluent) to afford diolefin **8ob**$_{\mathbf{di}}$ (13.8 mg, 26% yield, R_f = 0.16 in 9:1 EtOAc/MeOH) as a white solid.

Monoolefin 8ob$_{\mathbf{mono}}$: ^{1}H NMR (400 MHz, $CDCl_3$) δ 8.53 (d, *J* = 4.5 Hz, 1H), 7.96 (d, *J* = 15.5 Hz, 1H), 7.62 (d, *J* = 7.0 Hz, 1H), 7.53 (s, 1H), 7.45 (d, *J* = 8.0 Hz, 1H), 7.31-7.26 (comp. m, 5H), 7.25-7.21 (comp. m, 2H), 6.80 (d, *J* = 15.5 Hz, 1H), 6.23 (d, *J* = 1.5 Hz, 1H), 5.43 (dd, *J* = 13.0, 1.5 Hz, 1H), 5.23 (s, 1H), 5.12 (d, *J* = 13.0 Hz, 1H), 5.06 (d, *J* = 11.3 Hz, 1H), 4.76 (d, *J* = 11.3 Hz, 1H), 3.37 (s, 3H), 3.14 (s, 3H), 3.06 (s, 3H); ^{13}C NMR (100 MHz, $CDCl_3$) δ 166.7, 157.5, 149.7, 142.2, 141.8, 139.3, 135.4, 135.2, 133.5, 130.4, 130.0, 128.5, 128.0, 127.9, 127.7, 127.0, 125.12, 125.08, 123.6, 120.4, 104.2, 85.1, 70.7, 67.6, 57.2, 37.7, 36.1; IR (film) 1648, 1600, 1072, 1003, 732 cm^{-1}; HRMS (ESI+) *m/z* calc'd for $(M + H)^+$ $[C_{27}H_{28}N_2O_4 + H]^+$: 445.2122, found 445.2122.

Diolefin 8ob$_{\mathbf{di}}$: ^{1}H NMR (400 MHz, $CDCl_3$) δ 8.48 (d, *J* = 5.0 Hz, 1H), 8.03 (d, 15.5 Hz, 2H), 7.61 (d, *J* = 8.0 Hz, 1H), 7.44 (s, 2H), 7.33-7.22 (comp. m, 5H), 7.21 (dd, *J* = 8.0, 5.0, 1H), 6.81 (d, *J* = 15.5 Hz, 2H), 6.24 (s, 1H), 5.58 (d, *J* = 13.5 Hz, 1H), 5.22 (s, 1H), 5.17 (d, *J* = 13.5 Hz, 1H), 5.05 (d, *J* = 10.8 Hz, 1H), 4.82 (d, *J* = 10.8 Hz, 1H), 3.37 (s, 3H), 3.18 (s, 3H), 3.08 (s, 3H); ^{13}C NMR (100 MHz, $CDCl_3$) δ 166.6, 157.4, 149.6, 142.6, 141.4, 140.0, 138.0, 133.7, 132.8, 130.0, 128.6, 127.9, 127.11, 127.09, 126.6, 126.5, 123.6, 122.2, 104.1, 85.0, 84.9, 70.9, 63.28, 63.26, 57.29, 57.26, 37.8, 36.1; IR (film) 1649, 1607, 1397, 1003, 669 cm^{-1}; HRMS (ESI+) *m/z* calc'd for $(M + H)^+$ $[C_{32}H_{35}N_3O_5 + H]^+$: 542.2649, found 542.2649.

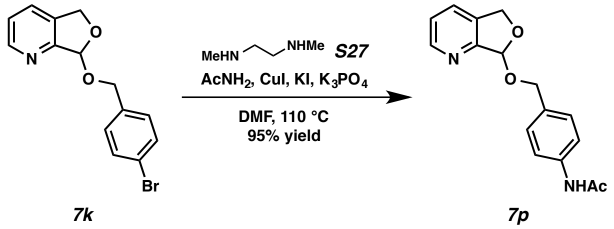

In a 2-dram vial, a suspension of acetal **7k** (91.8 mg, 0.300 mmol), K_3PO_4 (191 mg, 0.900 mmol), KI (49.8 mg, 0.300 mmol), *N,N'*-dimethylethylenediamine (**S27**, 29.1 µL, 0.270 mmol), CuI (17.1 mg, 90.0 µmol), and acetamide (70.9 mg, 1.20 mmol) in DMF (0.690 mL) was stirred while Ar was bubbled through the mixture (10 min). The vial was flushed with Ar, sealed with a PTFE-lined cap, and heated to 110 °C and stirred for 15 h. Upon completion, the mixture was diluted with a 10% aq. LiCl solution (30 mL) and was extracted with EtOAc (3 x 50 mL). The combined organic layers were washed with water (30 mL), then brine (30 mL), dried over $MgSO_4$, and concentrated by rotary evaporation. The crude amide was purified by flash column chromatography (EtOAc eluent) to afford acetanilide **7p** (81.0 mg, 95% yield, R_f = 0.12 in 40:1 EtOAc/MeOH) as a white solid.

Acetanilide 7p: ^{1}H NMR (400 MHz, $CDCl_3$) δ 8.56 (d, *J* = 4.8 Hz, 1H), 7.89 (br. s, 1H), 7.63 (d, *J* = 7.6 Hz, 1H), 7.46 (d, *J* = 8.4 Hz, 2H), 7.31 (d, *J* = 8.4 Hz, 2H), 7.27 (dd, *J* = 7.6, 4.8 Hz, 1H), 6.20 (s, 1H), 5.25 (d, *J* = 13.2 Hz, 1H), 5.08 (d, *J* = 13.2 Hz, 1H), 4.76 (ABq, *J* = 11.2 Hz, Δν = 34.1 Hz, 2H), 2.10 (s, 3H); ^{13}C NMR (100 MHz, $CDCl_3$) δ 168.6, 157.5, 149.7, 137.8, 133.3, 133.2, 130.1, 129.1, 123.7, 119.8, 104.0, 70.5, 69.9, 24.6; IR (film) 3306, 1668, 1537, 1315, 1007 cm^{-1}; HRMS (ESI+) *m/z* calc'd for $(M + Na)^+$ $[C_{16}H_{16}N_2O_3 + Na]^+$: 307.1053, found 307.1054.

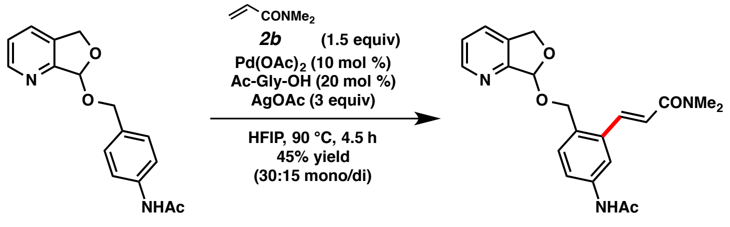

A suspension of acetal **7p** (28.4 mg, 0.100 mmol), *N,N*-dimethylacrylamide (**2b**, 15.5 µL, 0.150 mmol), $Pd(OAc)_2$ (2.2 mg, 10.0 µmol), *N*-acetylglycine (2.3 mg, 20.0 µmol), and AgOAc (50.1 mg, 0.300 mmol) in hexafluoroisopropanol (2.00 mL) in a 2-dram vial with a PTFE-lined cap was heated at 90 °C and stirred for 4.5 h. Upon completion, the reaction was cooled to ambient temperature, ethylenediamine (40 µL) and celite (70 mg) were added, and the resulting mixture was stirred overnight. The mixture was filtered through a plug of SiO_2 (5 mm x 20 mm, MeOH eluent, ~20 mL), the filtrate was concentrated by rotary evaporation, and the resulting residue was dissolved in EtOAc and then filtered through a second plug of SiO_2 (5 mm x 20 mm, MeOH eluent, ~20 mL). The filtrate was concentrated by rotary evaporation, and the crude residue was purified by flash column chromatography (9:1 EtOAc/MeOH eluent) to afford monoolefin **8pb$_{mono}$** (10.7 mg, 28% yield, R_f = 0.15 in 9:1 EtOAc/MeOH) as a white solid. The column was washed with MeOH, the filtrate concentrated by rotary evaporation, and the residue was purified by preparatory plate chromatography (9:1 EtOAc/MeOH eluent) to afford diolefin **8pb$_{di}$** (8.1 mg, 7.9:1 di/mono, 17% yield, R_f = 0.03 in 9:1 EtOAc/MeOH) as a beige solid.

Monoolefin 8pb$_{mono}$: ^{1}H NMR (400 MHz, $CDCl_3$) δ 8.53 (d, *J* = 4.8 Hz, 1H), 8.14 (s, 1H), 7.92 (d, *J* = 15.2 Hz, 1H), 7.88 (s, 1H), 7.64 (d, *J* = 7.6 Hz, 1H), 7.32-7.23 (comp. m, 3H), 6.80 (d, *J* = 15.2 Hz, 1H), 6.23 (s, 1H), 5.43 (d, *J* = 13.6 Hz, 1H), 5.11 (d, *J* = 13.6 Hz, 1H), 5.01 (d, *J* = 11.0 Hz, 1H), 4.68 (d, *J* = 11.0 Hz, 1H), 3.10 (s, 3H), 3.03 (s, 3H), 2.13 (s, 3H); ^{13}C NMR (100 MHz, $CDCl_3$) δ 168.8, 166.8, 157.5, 149.6, 139.1, 138.5, 135.7, 133.7, 131.7, 131.1, 130.2, 123.8, 120.5, 120.4, 117.7, 104.1, 70.7, 67.6, 37.6, 36.1, 24.7; IR (film) 1642, 1589, 1394, 1005, 726 cm^{-1}; HRMS (ESI+) *m/z* calc'd for $(M + H)^+$ $[C_{21}H_{23}N_3O_4 + H]^+$: 382.1761, found 382.1761.

Diolefin 8pb$_{di}$: ^{1}H NMR (400 MHz, $CDCl_3$) δ 8.83 (br. s, 1H), 8.50 (d, *J* = 4.8 Hz, 1H), 7.92 (d, *J* = 15.2 Hz, 2H), 7.68 (d, *J* = 7.6 Hz, 1H), 7.63 (s, 2H), 7.30-7.24 (comp. m, 1H), 6.68 (d, *J* = 15.2, 2H),

6.33 (s, 1H), 5.56 (d, *J* = 13.8 Hz, 1H), 5.20 (d, *J* = 13.8 Hz, 1H), 5.05 (d, *J* = 10.6 Hz, 1H), 4.80 (d, *J* = 10.6 Hz, 1H), 3.07 (s, 6H), 3.02 (s, 6H), 2,14 (s, 3H); ^{13}C NMR (100 MHz, $CDCl_3$) δ 169.2, 166.4, 157.5, 149.4, 139.2, 139.1, 137.5, 133.9, 130.3, 129.1, 123.9, 121.5, 118.6, 104.3, 70.9, 63.0, 37.6, 36.1, 24.6; IR (film) 1642, 1595, 1005, 910, 741 cm^{-1}; HRMS (ESI+) *m/z* calc'd for $(M + H)^+$ $[C_{26}H_{30}N_4O_5 + H]^+$: 479.2289, found 479.2290.

Structural Confirmation of Specifically Functionalized Compounds

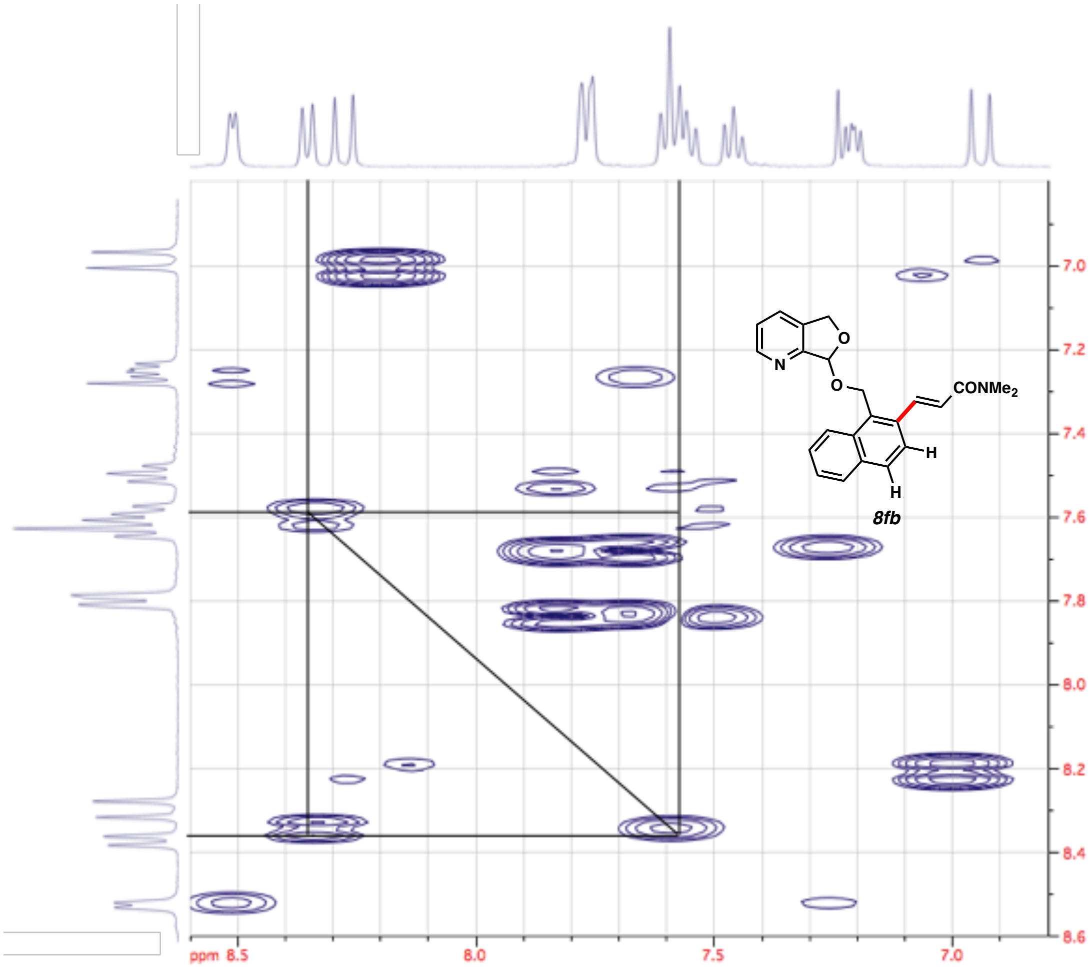

Acetal **7f** was olefinated at the C-2 position on the naphthyl ring. To unambiguously assign the structure of olefin **8fb**, a ^{1}H-^{1}H COSY was performed. Three arene spin systems were observed: the pyridine system containing 3 hydrogens, a system containing 4 hydrogens, and a third system that contains only 2 hydrogens. This evidence supports the assigned structure where olefination occurs at C-2.

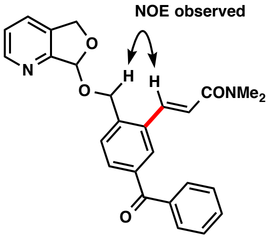

8nb

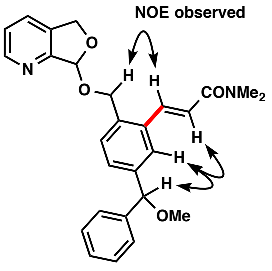

8ob

The structure of monoolefin **8nb** and monoolefin **8ob** were determined by the observation of an NOE interaction.

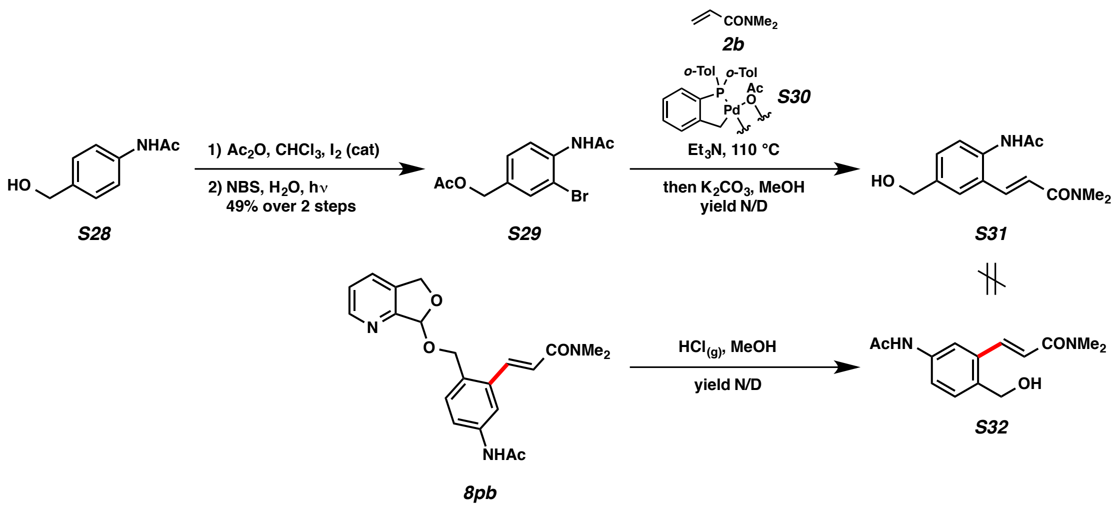

The structure of monoolefin **8pb** was determined by cleavage of the PyA scaffold with methanolic HCl to afford alcohol **S32** and comparing the resulting product to independently synthesized alcohol **S31**. Alcohol **S28** was synthesized by acetylation of benzyl alcohol **1a**[11] and bromination[12] of the resulting ester. Cross coupling between the aryl bromide **S29** and acrylamide **2b** followed by methanolic potassium carbonate afforded alcohol **S31**. Alcohol **S31** and alcohol **S32** did not match by ^{1}H NMR.

Analysis of Competency of Potentially Competitive Directing Groups

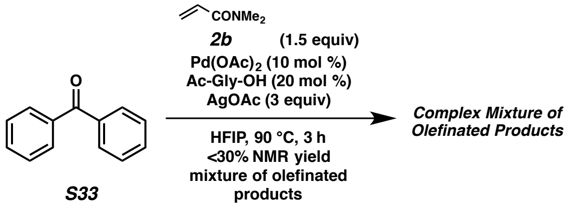

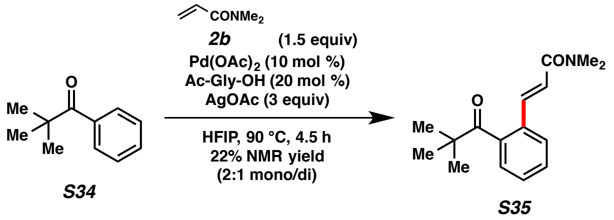

Benzophenone (**S33**) was subjected to the reaction conditions, affording a complex mixture of olefinated products. Ketone **S34** was subjected to the reaction conditions, affording olefin **S35** (2:1 mono/di, 22% NMR yield).

Ketone S35: Representative peaks of mono- and diolefinated products in ^{1}H NMR (400 MHz, $CDCl_3$) δ 7.66 (d, J = 15.6 Hz), 7.64 (d, J = 15.6 Hz), 6.86 (d, J = 15.6 Hz), 6.84 (d, J = 15.9 Hz).

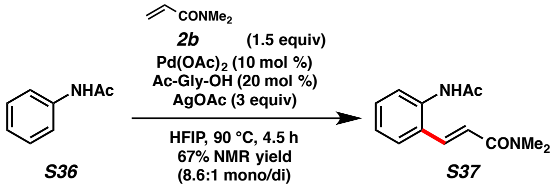

Acetanilide (**S36**) was subjected to the reaction conditions, affording olefin **S37** (8.6:1 mono/di, 67% NMR yield).

Olefin S37: This compound's spectroscopic data corresponds to previously reported data.[13]

Alcohol Substrate Synthesis

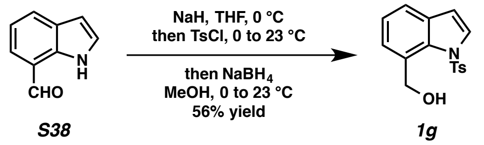

To a stirring solution of 7-formylindole (**S38**,[14] 400 mg, 2.76 mmol) in THF (7.08 mL) at 0 °C was added NaH (165 mg, 60% dispersion in mineral oil, 4.13 mmol) in 4 portions over 2 min, and the mixture was held at 0 °C for 10 min. Then TsCl (788 mg, 4.13 mmol) was added in a single portion, and the resulting mixture was warmed to 23 °C. After 1 h, the reaction mixture was cooled to 0 °C, MeOH (2.00 mL) was added, and then $NaBH_4$ (313 mg, 8.27 mmol) was added in 4 portions over 2 min. The mixture was warmed to ambient temperature and stirred for 1 h. Upon completion, the reaction mixture was quenched with sat. aq. Na_2CO_3 (20 mL) and extracted with EtOAc (3 x 20 mL). The combined organic layers were washed with brine (20 mL), dried over $MgSO_4$, and concentrated by rotary evaporation. The crude reaction mixture was purified by flash column chromatography (13:7 hexanes/EtOAc eluent) to afford alcohol **1g** (465 mg, 56% yield, R_f = 0.33 in 13:7 hexanes/EtOAc) as a colorless oil that slowly transformed to a white solid.

Alcohol 1g: ^{1}H NMR (400 MHz, $CDCl_3$) δ 7.70 (d, *J* = 3.8 Hz, 1H), 7.52 (d, *J* = 8.2 Hz, 2H), 7.49 (d, *J* = 7.6 Hz, 1H), 7.33 (d, *J* = 7.6 Hz, 1H), 7.24 (t, *J* = 7.6 Hz, 1H), 7.20 (d, *J* = 8.2 Hz, 2H), 6.73 (d, *J* = 3.8 Hz, 1H), 4.88 (s, 2H), 3.31 (br. s, 1H), 2.35 (s, 3H); ^{13}C NMR (100 MHz, $CDCl_3$) δ 145.2, 135.7, 133.6, 133.5, 130.1, 130.0, 128.4, 127.9, 126.4, 124.5, 121.6, 110.3, 63.9, 21.7; IR (film) 3379, 1350, 1165, 1072, 980 cm^{-1}; HRMS (ESI+) *m/z* calc'd for $(M + Na)^+$ $[C_{16}H_{15}NO_3S + Na]^+$: 324.0665, found 324.0672.

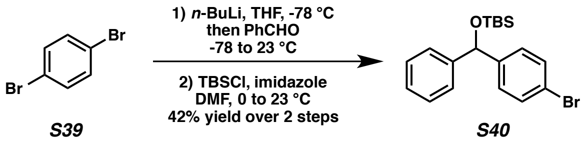

To a solution of 1,4-dibromobenzene (**S39**, 2.00 g, 8.48 mmol) in THF (42.4 mL) at -78 °C was added *n*-BuLi (3.56 mL, 2.50 M in hexanes, 8.90 mmol) over 5 min, and the solution was stirred for 30 min. To this solution was added benzaldehyde (0.948 mL, 9.33 mmol) dropwise over 1 min, and the solution was left to stir for 30 min before the cooling bath was removed and the reaction was allowed to warm to 23 °C. The reaction mixture was diluted with water (30 mL) and extracted with Et_2O (3 x 30 mL). The combined organic layers were washed with brine (15 mL), dried with $MgSO_4$, and concentrated by rotary evaporation. The residue was purified by flash column chromatography (4:1 hexanes/Et_2O) to afford the alcohol (2.04 g, R_f = 0.22 in 4:1 hexanes/Et_2O) that was sufficiently pure to be carried to the next step. This material was dissolved in DMF (14.5 mL) and cooled to 0 °C. TBSCl (2.33 g, 15.5 mmol) and then imidazole (1.58 g, 23.2 mmol) were added to the stirring solution, and it was maintained at 0 °C for 30 min before the solution was warmed to 23 °C. The solution was stirred at 23 °C for 24 h, and it was then diluted with water (30 mL) and extracted with Et_2O (3 x 30 mL). The combined organic layers were washed with brine (15 mL), dried over $MgSO_4$, and concentrated by rotary evaporation. The crude material was purified by flash column chromatography (hexanes eluent) to afford silyl ether **S40** (1.89 g, 42% yield over 2 steps, R_f = 0.38 in hexanes) as a colorless oil.

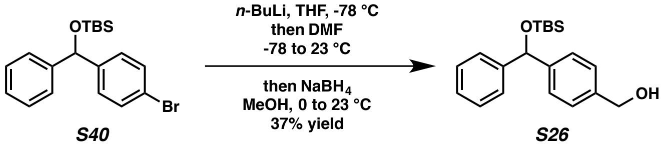

To a solution of silyl ether **S40** (783 mg, 2.08 mmol) in THF (10.3 mL) at -78 °C was added *n*-BuLi (0.871 mL, 2.50 M in hexanes, 2.18 mmol) over 5 min, and the solution was stirred for 30 min. To this orange solution was added DMF (0.800 mL, 10.4 mmol) dropwise over 1 min, and the solution was stirred for 30 min before the cooling bath was removed, and the reaction was allowed to warm to 23 °C. The reaction mixture was cooled to 0 °C, and MeOH (5.20 mL) was added, followed by $NaBH_4$ (157 mg, 4.15 mmol) in 1 portion. The cooling bath was removed, and the suspension was stirred for 10 min at 23 °C. The reaction mixture was diluted with water (20 mL) and extracted with Et_2O (3 x 20 mL). The combined organic layers were washed with brine (20 mL), dried over $MgSO_4$, and concentrated by rotary evaporation. The crude alcohol was purified by flash column chromatography (3:2 hexanes/Et_2O) to afford alcohol **S26** (254 mg, 37% yield, R_f = 0.33 in 3:2 hexanes/Et_2O) as a colorless oil.

Alcohol S26: ^{1}H NMR (500 MHz, $CDCl_3$) δ 7.40 (d, *J* = 8.0 Hz, 2H), 7.37 (d, *J* = 7.0 Hz, 2H), 7.30 (t, *J* = 7.0 Hz, 2H), 7.29 (d, *J* = 8.0 Hz, 2H), 7.21 (t, *J* = 7.0 Hz, 1H), 5.77 (s, 1H), 4.64 (s, 2H), 1.79 (s, 1H), 0.94 (s, 9H), 0.00 (s, 6H); ^{13}C NMR (125 MHz, $CDCl_3$) δ 145.3, 144.9, 139.6, 128.3, 127.1, 126.6, 126.3, 76.5, 65.3, 26.0, 18.4, -4.68, -4.70; IR (film) 3113, 862, 835, 778, 699 cm^{-1}; HRMS (ESI+) *m/z* calc'd for $(M + Na)^+$ $[C_{20}H_{28}O_2Si + Na]^+$: 351.1751, found 351.1751.

Alternative Scaffold Attachment Method, Telescoping, and Scaffold Recovery

*Hemiacetal **5** as an option for attachment and functionalization*

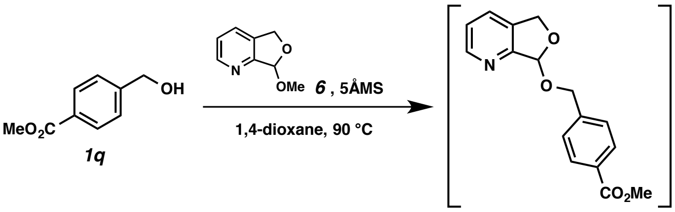

7q
not observed

In a 2-dram vial with a PTFE-lined cap, a suspension of alcohol **1q** (166 mg, 1.00 mmol), methyl acetal **6** (159 mg, 1.05 mmol), and 5Å molecular sieves (1.25 g) in 1,4-dioxane (1.59 mL) was heated at 90 °C (without stirring) for 8 h. Upon completion, the reaction was cooled to ambient temperature, and EtOAc (~20.0 mL) and celite (~2.00 g) were added to the mixture. This suspension was filtered through a pad of celite (65 mm x 15 mm) with 9:1 EtOAc/MeOH (200 mL), and the filtrate was concentrated by rotary evaporation. Acetal **7q** was not observed by NMR in the crude residue.

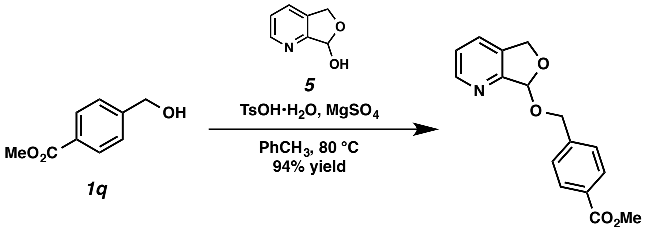

7q

A suspension of alcohol **1q** (28.7 mg, 0.173 mmol), hemiacetal **5** (25.0 mg, 0.182 mmol), TsOH•H_2O (3.3 mg, 17.3 µmol), and $MgSO_4$ (62.5 mg, 0.519 mmol) in $PhCH_3$ (0.576 mL) in a 2-dram vial was heated to 80 °C and stirred 24 h. Upon completion, water (15 mL) was added, and the mixture was extracted with EtOAc (3 x 15 mL). The combined organic layers were washed with brine (15 mL), dried over $MgSO_4$, and concentrated by rotary evaporation. The crude mixture was purified by flash column chromatography (3:2 hexanes/EtOAc eluent) to afford acetal **7q** (46.2 mg, 94% yield, R_f = 0.30 in 3:2 hexanes/EtOAc) as a white solid.

Acetal 7q: ^{1}H NMR (400 MHz, $CDCl_3$) δ 8.60 (d, *J* = 4.8 Hz, 1H), 8.00 (d, *J* = 8.0 Hz, 2H), 7.65 (d, *J* = 7.6 Hz, 1H), 7.48 (d, *J* = 8.0 Hz, 2H), 7.29 (dd, *J* = 7.6, 4.8 Hz, 1H), 6.23 (s, 1H), 5.26 (d, *J* = 13.2 Hz, 1H), 5.09 (d, *J* = 13.2 Hz, 1H), 4.89 (ABq, *J* = 12.4 Hz, Δν = 22.3, 2H), 3.90 (s, 3H); ^{13}C NMR (100 MHz, $CDCl_3$) δ 167.1, 157.4, 150.0, 143.1, 133.2, 130.0, 129.8, 129.5, 127.7, 123.8, 104.6, 70.6, 69.6, 52.2; IR (film) 1719, 1279, 1108, 1008, 755 cm^{-1}; HRMS (ESI+) *m/z* calc'd for $(M + H)^+$ $[C_{16}H_{15}NO_4 + H]^+$: 286.1074, found 286.1075.

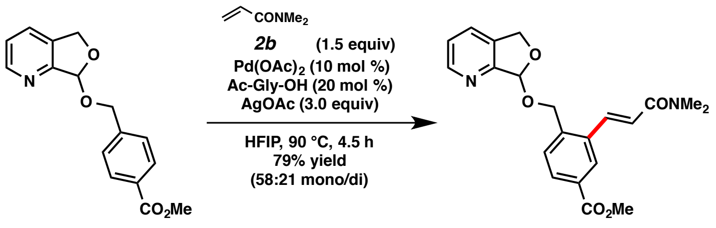

7q 8qb

A suspension of acetal **7q** (28.5 mg, 0.100 mmol), *N,N*-dimethylacrylamide (**2b**, 15.5 µL, 0.150 mmol), $Pd(OAc)_2$ (2.2 mg, 10.0 µmol), *N*-acetylglycine (2.3 mg, 20.0 µmol), and AgOAc (50.1 mg, 0.150 mmol) in hexafluoroisopropanol (2.00 mL) in a 2-dram vial with a PTFE-lined cap was heated to 90 °C and stirred for 4.5 h. Upon completion, the reaction was cooled to ambient temperature, ethylenediamine (40 µL) and celite (70 mg) were added, and the resulting mixture was stirred overnight. The mixture was filtered through a plug of SiO_2 (5 mm x 20 mm, EtOAc → 1:1 EtOAc/MeOH eluent, ~20 mL), the filtrate was concentrated by rotary evaporation, and the resulting residue was dissolved in EtOAc and then filtered through a second plug of SiO_2 (5 mm x 20 mm, EtOAc → 1:1 EtOAc/MeOH eluent, ~20 mL). The filtrate was concentrated by rotary evaporation, and the crude residue was purified by flash column chromatography (EtOAc eluent) to afford monoolefin **8qb** (22.2 mg, 58% yield, R_f = 0.26 in EtOAc) as a white solid. The column was washed with MeOH, the filtrate concentrated by rotary evaporation, and the residue was purified by preparatory thin-layer chromatography (9:1 EtOAc/MeOH eluent) to afford diolefin **8qb** (10.1 mg, 21% yield, R_f = 0.18 in 9:1 EtOAc/MeOH) as a white solid.

Monoolefin 8qb: ^{1}H NMR (400 MHz, $CDCl_3$) δ 8.56 (d, *J* = 4.4 Hz, 1H), 8.20 (d, *J* = 1.6 Hz, 1H), 7.96 (dd, *J* = 8.0, 1.6 Hz, 1H), 7.94 (d, *J* = 15.2 Hz, 1H), 7.65 (d, *J* = 8.0 Hz, 1H), 7.60 (d, *J* = 8.0 Hz, 1H), 7.27 (dd, *J* = 8.0, 4.4 Hz, 1H), 6.90 (d, *J* = 15.6 Hz, 1H), 6.26 (d, *J* = 1.6 Hz, 1H), 5.41 (dd, *J* = 13.0, 1.6 Hz, 1H), 5.13 (d, *J* = 13.0 Hz, 1H), 5.11 (d, *J* = 12.2 Hz, 1H), 4.86 (d, *J* = 12.2 Hz, 1H), 3.92 (s, 3H), 3.18 (s, 3H), 3.08 (s, 3H); ^{13}C NMR (100 MHz, $CDCl_3$) δ 166.8, 166.4, 157.3, 149.9, 141.3, 138.2, 134.8, 133.5, 130.11, 130.07, 129.9, 129.5, 127.7, 123.8, 121.1, 104.5, 70.8, 67.3, 52.4, 37.7, 36.1; IR (film) 1721, 1651, 1605, 998, 727 cm^{-1}; HRMS (ESI+) *m/z* calc'd for $(M + H)^+$ $[C_{21}H_{22}N_2O_5 + H]^+$: 383.1601, found 383.1600.

Diolefin 8qb: ^{1}H NMR (400 MHz, $CDCl_3$) δ 8.50 (d, *J* = 4.8 Hz, 1H), 8.11 (s, 2H), 8.07 (d, *J* = 15.2 Hz, 2H), 7.63 (d, *J* = 7.6 Hz, 1H), 7.23 (dd, *J* = 7.6, 4.8 Hz, 1H), 6.90 (d, *J* = 15.2 Hz, 2H), 6.26 (s, 1H), 5.60 (d, *J* = 13.8 Hz, 1H), 5.20 (d, *J* = 13.8 Hz, 1H), 5.09 (d, *J* = 10.2 Hz, 1H), 4.89 (d, *J* = 10.2 Hz, 1H), 3.93 (s, 3H), 3.20 (s, 6H), 3.09 (s, 6H); ^{13}C NMR (100 MHz, $CDCl_3$) δ 166.4, 166.3, 157.2, 149.7, 139.2, 138.1, 133.7, 130.4, 130.0, 128.7, 123.7, 122.8, 104.3, 71.0, 63.0, 52.6, 37.8, 36.1; IR (film) 1722, 1654, 1612, 1397, 1008 cm^{-1}; HRMS (ESI+) *m/z* calc'd for $(M + H)^+$ $[C_{26}H_{29}N_3O_6 + H]^+$: 480.2128, found 480.2131.

Telescoping procedure

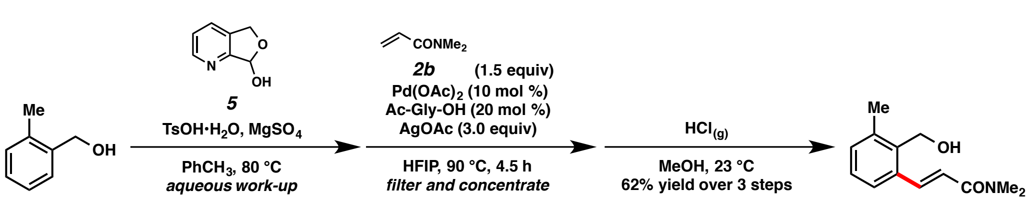

1b 9bb

A suspension of 2-methylbenzyl alcohol (**1b**, 12.2 mg, 0.100 mmol), hemiacetal **5** (13.7 mg, 0.105 mmol), TsOH•H_2O (1.9 mg, 10.0 µmol), and $MgSO_4$ (36.1 mg, 0.300 mmol) in $PhCH_3$ (0.330 mL) in a 2-dram vial was heated to 80 °C and stirred for 18 h. Upon completion, sat. aq. Na_2CO_3 (5 mL) was added to the reaction mixture, and the mixture was extracted into EtOAc (3 x 10 mL). The combined organic layers were washed with brine (10 mL), dried over $MgSO_4$, and concentrated by rotary evaporation to afford a crude mixture containing the product acetal (**7b**). This crude mixture was treated with $Pd(OAc)_2$ (2.0 mg, 9.00 µmol), *N*-acetylglycine (2.1 mg, 18.0 µmol), AgOAc (45.1 mg, 0.270 mmol), and *N,N*-dimethylacrylamide (**2b**, 13.9 µL, 0.135 mmol) in hexafluoroisopropanol (2.00 mL) in a 2-dram vial with a PTFE-lined cap, which was then heated to 90 °C and stirred for 4.5 h. Upon completion, the reaction was cooled to ambient temperature, ethylenediamine (40 µL) and celite (70 mg) were added, and the resulting mixture was stirred overnight. The mixture was filtered through a plug of SiO_2 (5 mm x 20 mm, EtOAc → 9:1 EtOAc/MeOH eluent, ~20 mL), the filtrate was concentrated by rotary evaporation, and the resulting residue was dissolved in EtOAc and then filtered through a second plug of SiO_2 (5 mm x 20 mm, EtOAc → 9:1 EtOAc/MeOH eluent, ~20 mL). The filtrate was concentrated by rotary evaporation to afford crude olefin **8bb**. This mixture was dissolved in MeOH (1.00 mL), and anhydrous HCl was bubbled into the solution for 1 min. The reaction mixture was stirred at 23 °C in a sealed 2-dram vial for 24 h, and it was then concentrated by rotary evaporation, and MeOH was azeotropically removed with Et_2O. The crude mixture was purified by flash column chromatography (EtOAc eluent) on basic SiO_2 (treated with 9:1 hexanes/triethylamine and dried by rotary evaporation) to afford alcohol **9bb** (13.5 mg, 62% yield over 3 steps, R_f = 0.20 in EtOAc) as a white solid.

Alcohol 9bb: ^{1}H NMR (400 MHz, $CDCl_3$) δ 8.07 (d, *J* = 15.2 Hz, 1H), 7.38 (dd, *J* = 5.8, 3.0 Hz, 1H), 7.20 (d, *J* = 3.0 Hz, 1H), 7.20 (d, *J* = 5.8 Hz, 1H), 6.79 (d, *J* = 15.2 Hz, 1H), 4.81 (s, 2H), 3.15 (s, 3H), 3.04 (s, 3H), 2.71 (br. s, 1H), 2.46 (s, 3H); ^{13}C NMR (100 MHz, $CDCl_3$) δ 166.9, 140.7, 138.3, 137.3, 135.8, 131.7, 128.2, 125.0, 120.4, 58.5, 37.6, 36.1, 19.6; IR (film) 3358, 1642, 1589, 1394, 1010 cm^{-1}; HRMS (ESI+) *m/z* calc'd for $(M + H)^+$ $[C_{13}H_{17}NO_2 + H]^+$; 220.1332, found 220.1333.

Note: Attempts to streamline the telescoping procedure further by circumventing the metal scavenging process with immediate acidic workup were met with limited success, as illustrated in the below experiment.

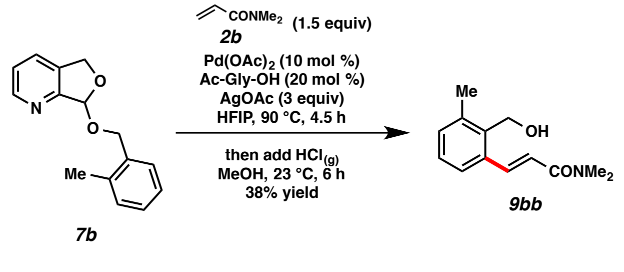

A suspension of acetal **7b** (24.1 mg, 0.100 mmol), *N,N*-dimethylacrylamide (**2b**, 15.5 µL, 0.150 mmol), $Pd(OAc)_2$ (2.2 mg, 10.0 µmol), *N*-acetylglycine (2.3 mg, 20.0 µmol), and AgOAc (50.1 mg, 0.300 mmol) in hexafluoroisopropanol (2.00 mL) in a 2-dram vial with a PTFE-lined cap was heated at 90 °C and stirred for 4.5 h. Upon completion by TLC, acidic MeOH (1.00 mL, bubbled with anhydrous HCl for 1 min) was added to the suspension, and the resulting mixture was stirred at 23 °C for 6 h. Upon completion by TLC, the reaction mixture was poured into water (15 mL) and extracted into EtOAc (3 x 15 mL). The combined organic layers were washed with brine (15 mL), dried over $MgSO_4$, and

concentrated by rotary evaporation. The crude mixture was purified by flash column chromatography (EtOAc eluent) on basic SiO_2 (treated with 9:1 hexanes/triethylamine and dried by rotary evaporation) to afford alcohol **9bb** (8.3 mg, 38% yield, R_f = 0.20 in EtOAc).

Note: As a comparison, the same reaction sequence was performed stepwise with intermediate purifications, affording alcohol **9bb** in 74% overall yield.

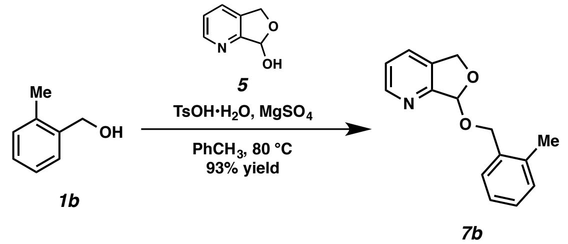

A suspension of 2-methylbenzyl alcohol (**1b**, 12.2 mg, 0.100 mmol), hemiacetal **5** (13.7 mg, 0.105 mmol), TsOH•H_2O (1.9 mg, 10.0 µmol), and $MgSO_4$ (36.1 mg, 0.300 mmol) in $PhCH_3$ (0.330 mL) in a 2-dram vial was heated to 80 °C and stirred for 18 h. Upon completion, sat. aq. Na_2CO_3 (5 mL) was added to the reaction mixture, and the mixture was extracted with EtOAc (3 x 10 mL). The combined organic layers were washed with brine (10 mL), dried over $MgSO_4$, and concentrated by rotary evaporation. The crude residue was purified by flash column chromatography (7:3 hexanes/EtOAc eluent) to afford acetal **7b** (22.3 mg, 93% yield, R_f = 0.36 in 3:2 hexanes/EtOAc) as a colorless oil.

The functionalization step (page S15) afforded acetal **8bb** in 80% yield.

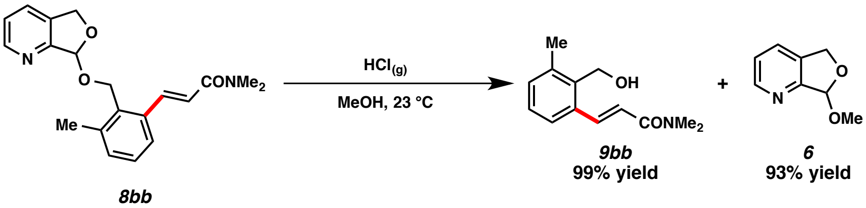

To a solution of acetal **8bb** (36.7 mg, 0.108 mmol) in MeOH (1.08 mL) was bubbled anhydrous HCl for 1 min. The reaction mixture was stirred at 23 °C in a sealed 2-dram vial for 36 h, and it was then concentrated by rotary evaporation and MeOH was azeotropically removed with Et_2O. The crude mixture was purified by flash column chromatography (EtOAc eluent) on basic SiO_2 (treated with 9:1 hexanes/triethylamine and dried by rotary evaporation) to afford alcohol **9bb** (23.6 mg, 99% yield, R_f = 0.20 in EtOAc) as a white solid and methyl acetal **6** (15.3 mg, 93% yield, R_f = 0.43 in EtOAc) as an amber oil.

Additional example of scaffold recovery

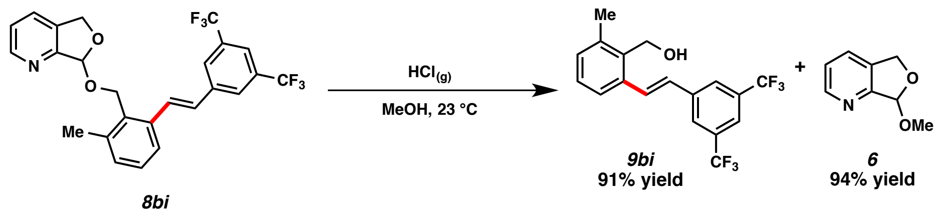

To a solution of acetal **8bi** (15.9 mg, 0.0332 mmol) in MeOH (0.332 mL) at 23 °C was bubbled anhydrous HCl for 1 min. The reaction mixture was stirred at 23 °C in a sealed 2-dram vial for 28 h, and it was then concentrated by rotary evaporation and MeOH was azeotropically removed with Et_2O. The crude mixture was purified by flash column chromatography (9:1 hexanes/EtOAc → EtOAc eluent) to afford alcohol **9bi** (10.9 mg, 91% yield, R_f = 0.25 in 9:1 hexanes/EtOAc) as a white solid and methyl acetal **6** (4.7 mg, 94% yield, R_f = 0.43 in EtOAc) as an amber oil.

Alcohol 9bi: ^{1}H NMR (400 MHz, $CDCl_3$) δ 7.92 (s, 2H), 7.76 (s, 1H), 7.70 (d, J = 16.2 Hz, 1H), 7.48 (d, J = 7.6 Hz, 1H), 7.27 (t, J = 7.6 Hz, 1H), 7.21 (d, J = 7.6 Hz, 1H), 7.05 (d, J = 16.2 Hz, 1H), 4.88 (d, J = 4.8 Hz, 2H), 2.49 (s, 3H), 1.47 (t, J = 4.8 Hz, 1H); ^{13}C NMR (100 MHz, $CDCl_3$) δ 139.7, 137.9, 136.8, 136.1, 132.2 (q, $^2J_{C\text{-}F}$ = 33.0 Hz), 131.2, 130.6, 128.8, 128.7, 126.5 (q, $^3J_{C\text{-}F}$ = 3.2 Hz), 124.8, 123.5 (q, $^1J_{C\text{-}F}$ = 271.0 Hz), 121.1 (septet, $^3J_{C\text{-}F}$ = 3.8 Hz), 59.2, 19.7; IR (film) 3374, 1379, 1278, 1163, 1126 cm^{-1}; HRMS (ESI-) m/z calc'd for $(M - H)^-$ $[C_{18}H_{14}F_6O - H]^-$: 359.0876, found 359.0877.

Additional Analysis of Acetal Cleavage

In addition to cleaving the acetal with gaseous HCl in MeOH, we found that the scaffold could be cleaved using aqueous HCl.

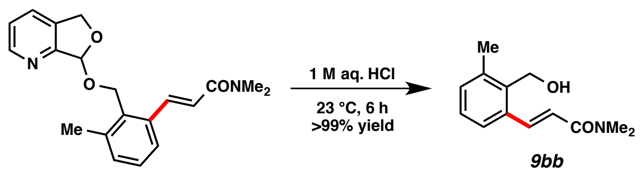

8bb ***9bb***

Acetal **8bb** (36.7 mg, 0.100 mmol) was dissolved in 1 M HCl (1.00 mL), and the mixture was stirred at 23 °C for 6 h. As the reaction proceeded, the clear suspension slowly became opaque. Upon completion by TLC, the reaction mixture was poured into water (15 mL) and extracted with EtOAc (3 x 15 mL). The combined organic layers were washed with brine (15 mL), dried over $MgSO_4$, and concentrated by rotary evaporation. The crude residue was purified by flash column chromatography (EtOAc eluent) on basic SiO_2 (treated with 9:1 hexanes/triethylamine and dried by rotary evaporation) to afford alcohol **9bb** (21.9 mg, >99% yield, R_f = 0.20 in EtOAc) as a white solid.

Structural Variants to Probe the Nature of the Directing Group

Synthesis of Scaffold Variants

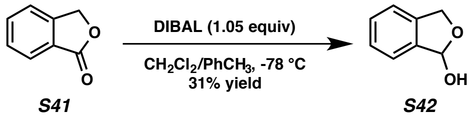

To a solution of phthalide **S41** (1.00 g, 7.46 mmol) in CH_2Cl_2 (34 mL) at -78 °C was added a solution of diisobutylaluminum hydride (5.65 mL, 1.32 M in $PhCH_3$, 7.83 mmol) over 10 min. The reaction mixture was stirred for 1.5 h at -78 °C, and upon completion sat. aq. Na_2SO_4 (5 mL) was added, and the mixture was allowed to warm to ambient temperature. To this mixture was added anhydrous Na_2SO_4 (10 g), and the mixture was stirred overnight and then filtered through a Buchner funnel and washed with CH_2Cl_2 (50 mL). The filtrate was concentrated by rotary evaporation, and the resulting oil was purified by flash column chromatography (1:1 Et_2O/hexanes → 2:1 Et_2O/hexanes eluent) to afford hemiacetal **S42** (314 mg, 31% yield, R_f = 0.21 in 2:1 hexanes/E_2O) as a colorless oil.
Hemiacetal S42: This compound's spectroscopic data corresponds to previously reported data.[15]

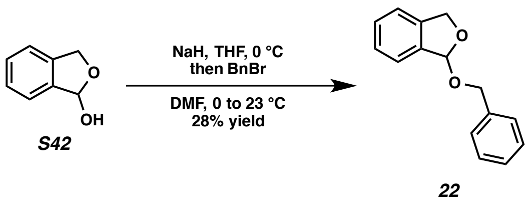

To a solution of hemiacetal **S42** (101 mg, 0.742 mmol) in THF (3.7 mL) at 0 °C was added NaH (74.2 mg, 60% dispersion in mineral oil, 1.86 mmol) in one portion, and the mixture was stirred at 0 °C for 1 h. To the suspension was added dropwise a solution of BnBr (1.00 mL, 0.705 M in DMF, 0.705 mmol), and the mixutre was warmed to 23 °C by removal of the cooling bath. The suspension was stirred for 20 h, and upon completion as determined by TLC, it was then quenched with sat. aq. $NaHCO_3$ (10 mL). The mixture was extracted with Et_2O (3 x 30 mL). The combined organic layers were washed with brine (10 mL), dried over $MgSO_4$, and concentrated by rotary evaporation. The crude residue was purified by column chromatography (39:1 → 9:1 hexanes/Et_2O eluent) to afford acetal **22** (46.4 mg, 28% yield, R_f = 0.36 in 9:1 hexanes/Et_2O) as a colorless oil.
Acetal 22: ^{1}H NMR (400 MHz, $CDCl_3$) δ 7.44-7.26 (comp. m, 9H), 6.36 (s, 1H), 5.27 (d, J = 12.6 Hz, 1H), 5.08 (d, J = 12.6 Hz, 1H), 4.73 (ABq, J = 11.6 Hz, Δν = 22.3 Hz, 2H); ^{13}C NMR (100 MHz, $CDCl_3$) δ 140.1, 138.2, 137.7, 129.3, 128.5, 128.1, 127.81, 127.76, 123.2, 121.2, 106.3, 72.5, 69.1; IR (film) 2359, 2340, 1005, 908, 729 cm^{-1}; HRMS (ESI+) *m/z* calc'd for $(M + H)^+$ $[C_{15}H_{14}O_2 + Na]^+$: 249.0886, found 249.0886.

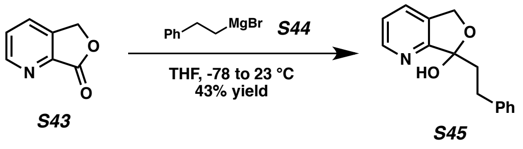

Magnesium turnings were ground in a mortar immediately before use. The metal (919 mg, 37.8 mmol) was suspended in THF (54 mL) and a few drops of iodine in methyl *tert*-butyl ether (10 M) were added. Then phenethyl bromide (5.00 g, 27.0 mmol) was added portionwise at 23 °C. The stirring solution was refluxed for 2 h to afford a solution of phenethylmagnesium bromide (0.035 M). In a separate flask, lactone **S43**[16] (250 mg, 1.85 mmol) was suspended in THF (1.9 mL) cooled to -78 °C, and to this suspension was added the Grignard solution (52.9 mL, 0.035 M, 1.85 mmol) dropwise over 10 min. The resulting suspension was stirred for 2.5 h, and then it was warmed to ambient temperature by removal of the cooling bath. Sat. aq. $NaHCO_3$ (20 mL) was added, and the mixture was extracted with EtOAc (4 x 50 mL). The combined organic layers were washed with brine (30 mL), dried over $MgSO_4$, and concentrated by rotary evaporation. The resulting oil was dissolved in CH_3CN (50 mL) and washed with pentane (3 x 50 mL), and the CH_3CN layer was removed by rotary evaporation. The crude oil was purified by flash column chromatography (3:1 EtOAc/hexanes eluent) to afford hemiacetal **S45** (192 mg, 43% yield, R_f = 0.29 in 3:1 EtOAc/hexanes) as a white solid.

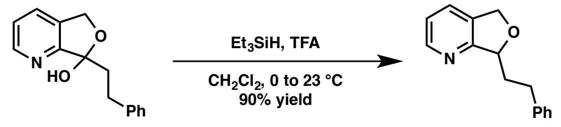

To a solution of hemiacetal **S45** (136 mg, 0.565 mmol) and triethylsilane (0.451 mL, 2.82 mmol) in CH_2Cl_2 (4.0 mL) at 0 °C was added trifluoroacetic acid (0.649 mL, 8.47 mmol) dropwise. The solution was stirred at 0 °C for 2 h, and then it was warmed to 23 °C by removal of the cooling bath, and it was stirred for 30 min. Upon completion as determined by TLC, water (5 mL) and CH_2Cl_2 (30 mL) were added to the reaction mixture, and the mixture was extracted with sat. aq. NH_4Cl (3 x 20 mL). The combined aqueous layers were brought to pH 12 by the addition of sat. aq. Na_2CO_3 (60 mL), and the mixture was extracted with EtOAc (4 x 30 mL). The combined EtOAc layers were washed with brine (20 mL), dried over $MgSO_4$, and concentrated by rotary evaporation to afford analytically pure ether **23** (115 mg, 90% yield, R_f = 0.37 in EtOAc) as a colorless oil.

Ether 23: ^{1}H NMR (400 MHz, $CDCl_3$) δ 8.46 (app. s, 1H), 7.85 (d, *J* = 8.0 Hz, 1H) 7.32-7.25 (comp. m, 3H), 7.23-7.16 (comp. m, 3H), 4.92 (br. s, 1H), 4.57 (ABq, *J* = 12.8 Hz, Δν = 36.7 Hz, 2H), 2.89-2.76 (comp. m, 2H), 2.03-1.88 (comp m, 2H); ^{13}C NMR (100 MHz, $CDCl_3$) δ 159.0, 146.8, 141.7, 137.2, 132.9, 128.7, 128.5, 126.1, 123.0, 69.3, 60.6, 39.8, 31.9; IR (film) 2359, 1678, 1454, 1202, 1036 cm^{-1}; HRMS (ESI+) *m/z* calc'd for $(M + H)^+$ $[C_{15}H_{15}NO + H]^+$: 226.1226, found 226.1226.

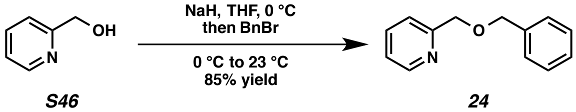

To a solution of pyridine **S46** (417 mg, 3.82 mmol) in THF (9.6 mL) at 0 °C was added NaH (229 mg, 60% dispersion in mineral oil, 5.73 mmol) in one portion. The suspension was stirred for 25 min at 0 °C, and then benzyl bromide (500 μL, 4.21 mmol) was added to the reaction in one portion at this temperature. The ice bath was removed, allowing the reaction to warm to 23 °C, and it was stirred for 2 h before being quenched with water (10 mL). Et_2O (30 mL) was added, and the mixture was extracted with 1 M aq. HCl (3 x 30 mL). The combined aqueous layers were neutralized with sat. aq. Na_2CO_3 (90 mL), and the mixture was extracted with Et_2O (3 x 30 mL). The combined organic layers were washed with brine (30 mL), dried over $MgSO_4$, and concentrated by rotary evaporation to afford ether

24 (643 mg, 85% yield, R_f = 0.27 in 4:1 hexanes/EtOAc) as a yellow oil, which was sufficiently pure to be evaluated in the functionalization.

Ether 24: This compound's spectroscopic data corresponds to previously reported data.[17]

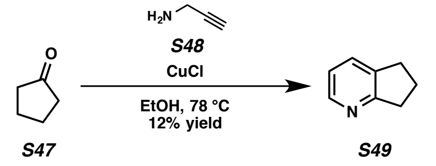

A suspension of cylcopentanone (**S47**, 1.26 g, 15.0 mmol), propargylamine (**S48**, 1.44 mL, 15.0 mmol), and anhydrous CuCl (50.4 mg, 0.375 mmol) in absolute EtOH (60 mL) was heated at reflux for 13 h. Upon completion as determined by TLC, EtOAc (50 mL) and 1 M aq. HCl (50 mL) were added. The layers were separated, and the organic layer was discarded. The pH of the aqueous layer was raised by the addition of sat. Na_2CO_3 (50 mL). The mixture was then extracted with EtOAc (3 x 50 mL), and the combined organic layers were dried over Na_2SO_4 and concentrated by rotary evaporation to afford pyridine **S49** (222 mg, 12% yield, R_f = 0.31 in EtOAc) as a light green oil, which was sufficiently pure to be carried to the next step.

Pyridine S49: This compound's spectroscopic data corresponds to previously reported data.[18]

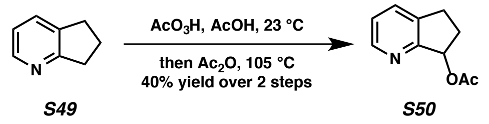

To a solution of pyridine **S49** (222 mg, 1.86 mmol) in CH_2Cl_2 (4.2 mL) at 23 °C was added peracetic acid (2.65 g, 32% w/w solution in dilute AcOH, 11.2 mmol), and the mixture was stirred at 23 °C for 8 h. Upon completion as determined by TLC, the mixture was extracted with water (5 mL), and residual peracetic acid was azeotropically removed by successive additions of water and concentration by rotary evaporation. (Peracetic acid removal was confirmed by starch paper test.) Ac_2O (3.2 mL) was added to the resulting crystalline solid (284 mg), and the mixture was heated to 105 °C and stirred for 10 min. The resulting dark brown mixture was concentrated by rotary evaporation, and Ac_2O was azeotropically removed with heptane. The residue was purified by flash column chromatography (1:1 EtOAc/hexanes eluent) to afford acetate **S50** (131 mg, 40% yield over 2 steps, R_f = 0.16 in 1:1 EtOAc/hexanes) as a colorless oil.

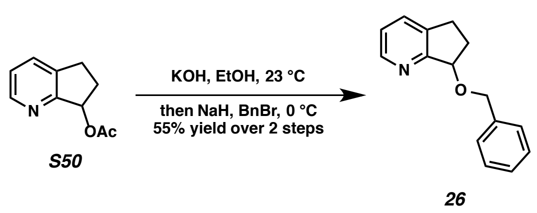

To a solution of acetate **S50** (131 mg, 0.738 mmol) in EtOH (1.5 mL) at 23 °C was added powdered KOH (124 mg, 2.21 mmol), and the reaction was stirred for 10 min. Upon completion as determined by TLC, AcOH (125 μL) was added to the reaction mixture, and it was then concentrated by rotary evaporation to afford a mixture of clear crystals suspended in a purple oil. This mixture was dissolved in THF (6.0 mL), and it was then cooled to 0 °C. To this solution was added NaH (148 mg, 60%

dispersion in mineral oil, 3.69 mmol), and the resulting mixture was stirred at 0 °C for 30 min. To the suspension was added benzyl bromide (439 µL, 3.69 mmol), and the reaction mixture was stirred at 0 °C for 1 h. The ice bath was removed and the mixture was allowed to warm to 23 °C and stirred for 1 h. Upon completion as determined by TLC, the reaction mixture was poured into 1 M aq. HCl (30 mL) and washed with EtOAc (3 x 30 mL). The organic layers were discarded, and the pH of the aqueous layer was raised with sat. aq. Na_2CO_3 (30 mL). The aqueous mixture was extracted with EtOAc (3 x 30 mL), and the combined organic layers were washed with brine (15 mL), dried over $MgSO_4$, and concentrated by rotary evaporation. The crude residue was purified with column chromatography (4:1 hexanes/EtOAc eluent) to afford ether **26** (91.2 mg, 55% yield over 2 steps, R_f = 0.25 in 4:1 hexanes/EtOAc) as a colorless oil.

Ether 26: ^{1}H NMR (400 MHz, $CDCl_3$) δ 8.49 (d, *J* = 4.8 Hz, 1H), 7.56 (d, *J* = 7.6 Hz, 1H), 7.41 (d, *J* = 7.2 Hz, 2H), 7.33 (t, *J* = 7.2 Hz, 2H), 7.26 (t, *J* = 7.2 Hz, 1H), 7.15 (dd, *J* = 7.6, 4.8 Hz, 1H), 4.95 (dd, *J* = 6.8, 3.6 Hz, 1H), 4.85 (ABq, *J* = 12.0 Hz, Δν = 44.0, 2H), 3.11 (ddd, *J* = 16.0, 8.0, 7.2 Hz, 1H), 2.81 (ddd, *J* = 16.0, 8.8, 4.8 Hz, 1H), 2.41-2.32 (comp. m, 1H), 2.25-2.15 (comp. m, 1H); ^{13}C NMR (100 MHz, $CDCl_3$) δ 163.3, 148.2, 138.7, 137.4, 133.1, 128.4, 128.0, 127.5, 123.1, 81.7, 71.4, 31.2, 28.1; IR (film) 1090, 1069, 791, 737, 698 cm^{-1}; HRMS (ESI+) *m/z* calc'd for $(M + H)^+$ $[C_{15}H_{15}NO + H]^+$: 226.1226, found 226.1227.

Functionalizations of Scaffold Variants

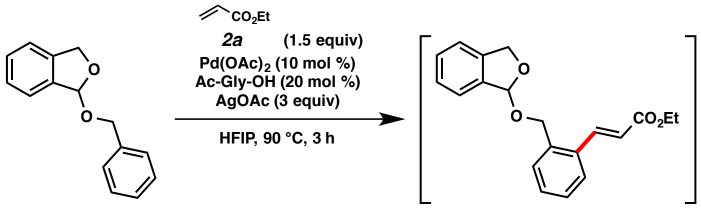

22 **S51** *not observed*

A suspension of acetal **22** (22.6 mg, 0.100 mmol), ethyl acrylate (**2a**, 16.0 µL, 0.150 mmol), $Pd(OAc)_2$ (2.2 mg, 10.0 µmol), *N*-acetylglycine (2.3 mg, 20.0 µmol), and AgOAc (50.1 mg, 0.300 mmol) in hexafluoroisopropanol (2.00 mL) in a 2-dram vial with a PTFE-lined cap was heated at 90 °C and stirred for 3 h. Upon completion, the reaction was cooled to ambient temperature, ethylenediamine (40 µL) and celite (70 mg) were added, and the resulting mixture was stirred overnight. The mixture was filtered through a plug of SiO_2 (5 mm x 20 mm, MeOH eluent, ~20 mL), the filtrate was concentrated by rotary evaporation, and the resulting residue was dissolved in EtOAc and then filtered through a second plug of SiO_2 (5 mm x 20 mm, MeOH eluent, ~20 mL). The filtrate was concentrated by rotary evaporation, and olefin **S51** was not observed by NMR in the crude residue.

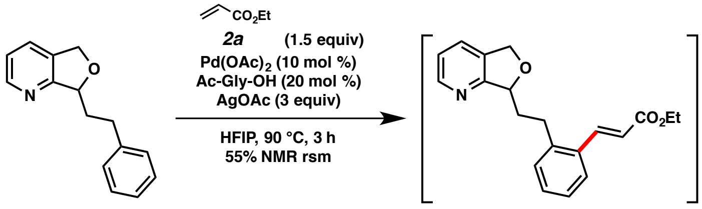

23 **S52** *not observed*

A suspension of pyridine **23** (22.5 mg, 0.100 mmol), ethyl acrylate (**2a**, 16.0 µL, 0.150 mmol), $Pd(OAc)_2$ (2.2 mg, 10.0 µmol), *N*-acetylglycine (2.3 mg, 20.0 µmol), and AgOAc (50.1 mg, 0.300 mmol) in hexafluoroisopropanol (2.00 mL) in a 2-dram vial with a PTFE-lined cap was heated at 90 °C and stirred for 3 h. Upon completion, the reaction was cooled to ambient temperature, ethylenediamine (40 µL) and celite (70 mg) were added, and the resulting mixture was stirred overnight. The mixture was filtered through a plug of SiO_2 (5 mm x 20 mm, MeOH eluent, ~20 mL), the filtrate was concentrated by rotary evaporation, and the resulting residue was dissolved in EtOAc and then filtered through a second plug of SiO_2 (5 mm x 20 mm, MeOH eluent, ~20 mL). The filtrate was concentrated by rotary evaporation, and olefin **S52** was not observed by NMR in the crude residue. Pyridine **23** (55% recovered starting material) was observed by NMR in the crude residue.

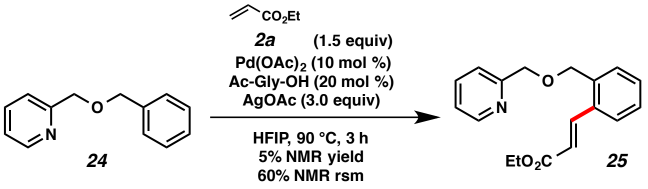

A suspension of ether **24** (19.9 mg, 0.100 mmol), ethyl acrylate (**2a**, 16.0 µL, 0.150 mmol), $Pd(OAc)_2$ (2.2 mg, 10.0 µmol), *N*-acetylglycine (2.3 mg, 20.0 µmol), and AgOAc (50.1 mg, 0.300 mmol) in hexafluoroisopropanol (2.00 mL) in a 2-dram vial with a PTFE-lined cap was heated to 90 °C and stirred for 3 h. Upon completion, the reaction was cooled to ambient temperature, ethylenediamine (40 µL) and celite (70 mg) were added, and the resulting mixture was stirred overnight. The mixture was filtered through a plug of SiO_2 (5 mm x 20 mm, EtOAc → 9:1 EtOAc/MeOH eluent), the filtrate was concentrated by rotary evaporation, and the resulting residue was dissolved in EtOAc and then filtered through a second plug of SiO_2 (5 mm x 20 mm, EtOAc → 9:1 EtOAc/MeOH eluent). The filtrate was concentrated by rotary evaporation, and olefin **25** was observed in small quantities (5% NMR yield, 60% recovered starting material) in the crude residue.

Olefin 25: Representative peaks in ^1H NMR (400 MHz, $CDCl_3$) δ 8.04 (d, *J* = 16.0 Hz, 1H), 6.39 (d, *J* = 16.0 Hz, 1H).

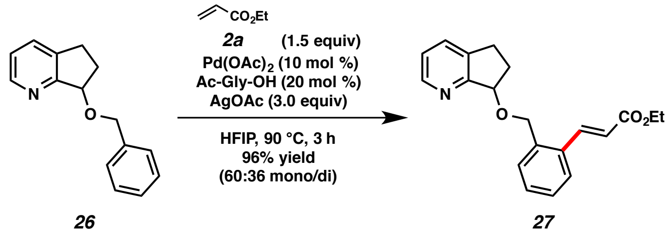

A suspension of pyridine **26** (22.6 mg, 0.100 mmol), ethyl acrylate (**2a**, 16.0 µL, 0.150 mmol), $Pd(OAc)_2$ (2.2 mg, 10.0 µmol), *N*-acetylglycine (2.3 mg, 20.0 µmol), and AgOAc (50.1 mg, 0.300 mmol) in hexafluoroisopropanol (2.00 mL) in a 2-dram vial with a PTFE-lined cap was heated to 90 °C and stirred for 3 h. Upon completion, the reaction was cooled to ambient temperature, ethylenediamine (40 µL) and celite (70 mg) were added, and the resulting mixture was stirred overnight. The mixture was filtered through a plug of SiO_2 (5 mm x 20 mm, EtOAc → 9:1 EtOAc/MeOH eluent), the filtrate was concentrated by rotary evaporation, and the resulting residue was dissolved in EtOAc and then filtered through a second plug of SiO_2 (5 mm x 20 mm, EtOAc → 9:1 EtOAc/MeOH eluent). The filtrate was concentrated by rotary evaporation, and the crude residue

was purified by flash column chromatography (1:1 hexanes/EtOAc eluent) to afford olefin **27** (34.9 mg, 1.6:1 mono/di, 96% yield, $R_{f(mono)}$ = 0.31 and $R_{f(di)}$ = 0.20 in 1:1 hexanes/EtOAc) as a white solid.
Olefin 27: ^{1}H NMR (400 MHz, $CDCl_3$) δ 8.55 (d, *J* = 4.8 Hz, 1H), 8.49 (d, *J* = 4.8 Hz, 1.6H), 8.15 (d, *J* = 15.6 Hz, 2H), 8.05 (d, *J* = 15.6 Hz, 1H), 7.61-7.56 (comp. m, 2.6H), 7.55 (d, *J* = 7.6 Hz, 2H), 7.48 (d, *J* = 7.6 Hz, 1.6H), 7.43-7.26 (comp. m, 5.8H), 7.21-7.14 (comp. m, 2.6H), 6.36 (d, *J* = 15.6 Hz, 1.6H), 6.33 (d, *J* = 15.6 Hz, 2H), 5.20 (d, *J* = 11.2 Hz, 1H), 5.03-4.96 (comp. m, 4.2H), 4.90 (d, *J* = 11.6 Hz, 1.6H), 4.88 (d, *J* = 11.2 Hz, 1H), 4.26 (q, *J* = 7.2 Hz, 7.2H), 3.18-3.08 (comp m, 2.6H), 2.89-2.79 (comp m, 2.6H), 2.48-2.38 (comp m, 2.6H), 2.28-2.18 (comp m, 2.6H), 1.34 (t, *J* = 7.2 Hz, 10.8H); ^{13}C NMR (100 MHz, $CDCl_3$) δ 167.0, 166.7, 163.1, 163.0, 148.3, 148.2, 142.6, 142.1, 137.6, 137.5, 137.4, 136.3, 135.5, 134.0, 133.2, 130.1, 130.0, 128.8, 128.5, 128.4, 128.3, 128.0, 126.8, 123.2, 121.7, 121.7, 120.1, 82.4, 82.3, 69.5, 64.6, 60.6, 60.5, 31.1, 28.2, 14.5; IR (film) 1711, 1166, 1045, 909, 730 cm^{-1}; HRMS (ESI+) *m/z* calc'd for $(M_{mono} + H)^+$ $[C_{20}H_{21}NO_3 + H]^+$: 324.1594, found 324.1595; HRMS (ESI+) *m/z* calc'd for $(M_{di} + H)^+$ $[C_{25}H_{27}NO_5 + H]^+$: 422.1962, found 422.1964.

---

[1] Piomelli, D.; Bandiera, T.; Bertozzi, F.; Nuzzi, A.; Fiasella, A.; Ponzano, S.; Pagliuca, C.; Reggiani, A. M. Carbamate Derivatives of Lactam Based *N*-Acylethanolamine Acid Amidase (NAAA) Inhibitors. Eur. Pat. Appl. WO 2014/144836 (A2), 2014.
[2] Shakhmaev, R. N.; Ishbaeva, A. U.; Zorin, V. V. *Russ. J. Org. Chem.* **2012**, *48*, 908-913.
[3] Corminboeuf, O.; Renaud, P. *Org. Lett.* **2002**, *4*, 1735-1738.
[4] Arisawa, M.; Fujii, Y.; Kato, H.; Fukuda, H.; Matsumoto, T.; Ito, M.; Abe, H.; Ito, Y.; Shuto, S. *Angw. Chem. Int. Ed.* **2013**, *52*, 1003-1010.
[5] Cuadrado, P.; González-Nogal, A. M.; Angeles Sarmentero, M. *Chem. Eur. J.* **2004**, *10*, 4491-4497.
[6] Wang, L.; Green, L.; Li, Z.; McCabe Dunn, J.; Bu, X.; Welch, C. J.; Li, C.; Wang, T.; Tu, Q.; Bekos, E.; Richardson, D.; Eckert, J.; Cui, J. *Org. Process Res. Dev*. **2011**, *15*, 1371-1376.
[7] Otera, J.; Dan-oh, N.; Nozaki, H. *J. Org. Chem*. **1991**, 56, 5307-5311.
[8] Chernyak, N.; Dudnik, A. S.; Huang, C.; Gevorgyan, V. *J. Am. Chem. Soc.* **2010**, *132*, 8270-8272.
[9] Lee, A. S.-Y.; Hu, Y.-J., Chu, S.-F. *Tetrahedron* **2001**, 57, 2121-2126.
[10] Bratt, M. O.; Taylor, P. C. *J. Org. Chem.* **2003**, *68*, 5439-5444.
[11] Drillaud, N.; Banaszak-Léonard, E.; Pezron, I.; Len, C. *J. Org. Chem.* **2012**, 9553-9561.
[12] Podgorsek, A.; Stavber, A.; Zupan, M.; Iskra, J. *Tetrahedron Lett.* **2006**, *47*, 1097-1099.
[13] Kim, B. S.; Jang, C.; Lee, D. J.; Youn, S. W. *Chem.—Asian J.* **2010**, 2336-2340.
[14] Moyer, M. P.; Shiurba, J. F.; Rapoport, H. *J. Org. Chem.* **1986**, *51*, 5106-5110.
[15] Mikami, K.; Ohmura, H. *Org. Lett.* **2002**, *4,* 3355-3357.
[16] He, G.; Chen, G. *Angew. Chem. Int. Ed.* **2011**, *50*, 5192-5196.
[17] Legault, C. Y.; Charette, A. B. *J. Am. Chem. Soc.* **2005**, *127*, 8966-8967.
[18] Abbiati, G.; Arcadi, A.; Bianchi, G.; Giuseppe, S. D.; Marenelli, F.; Rossi, E. *J. Org. Chem.* **2003**, *68*, 6959-6955.

Supporting Information - Spectra Compilation

The Design of a Readily Attachable and Cleavable Molecular Scaffold for ortho-Selective C–H Alkenylation of Arene Alcohols

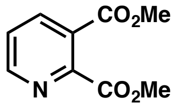

4
^{1}H NMR
(400 MHz, $CDCl_3$)

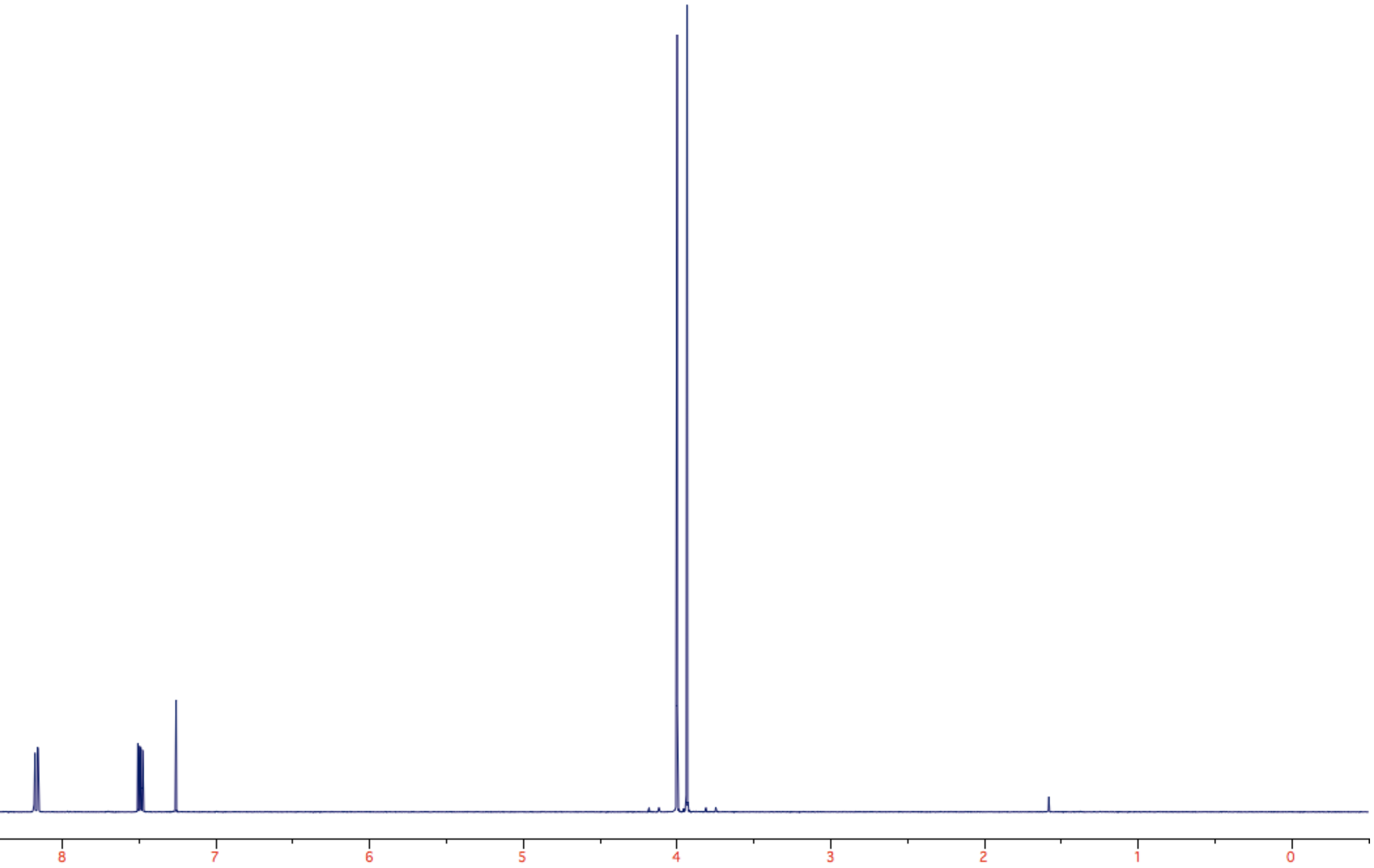

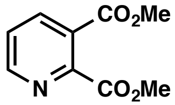

4

^{13}C NMR

(100 MHz, $CDCl_3$)

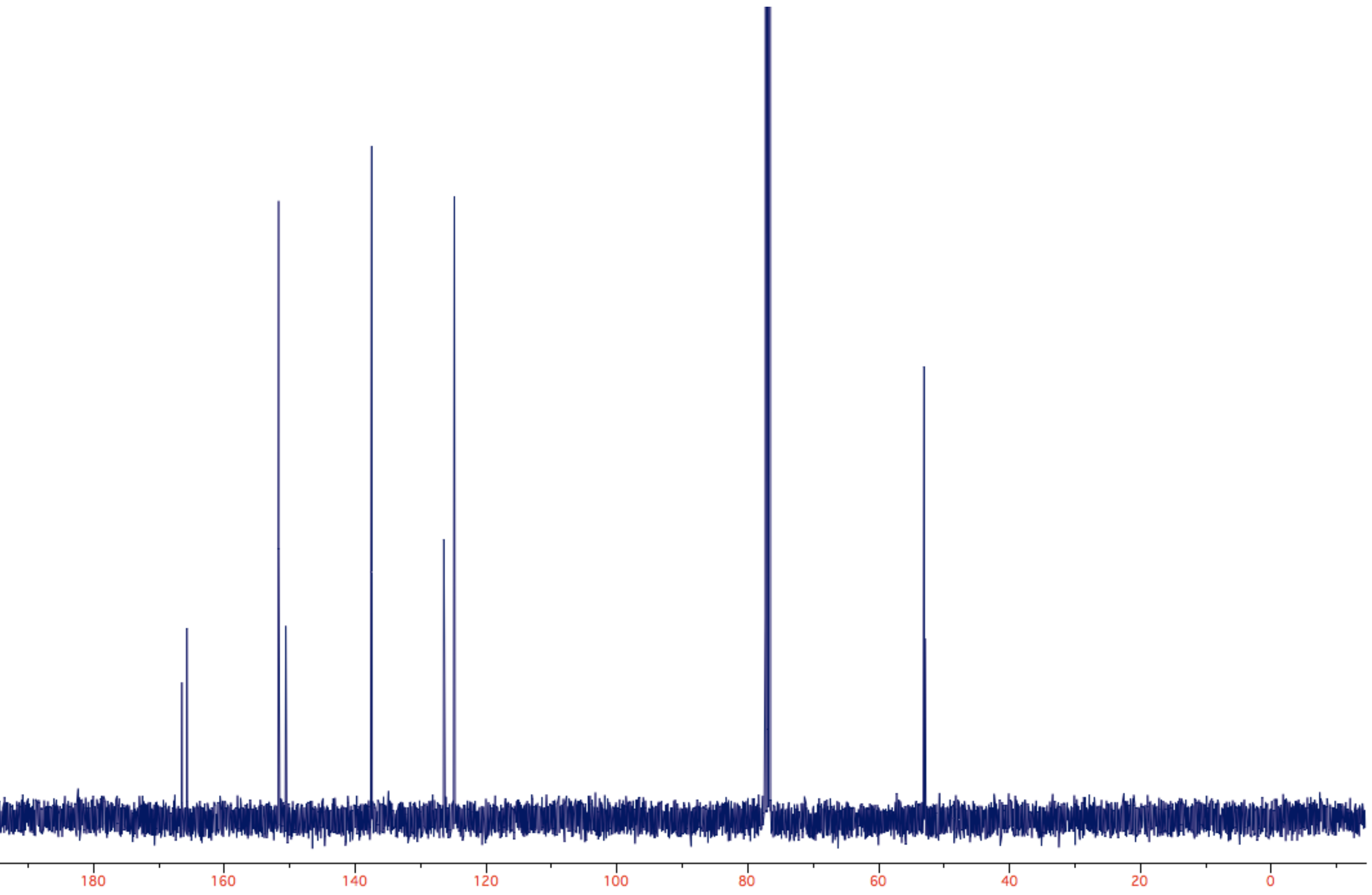

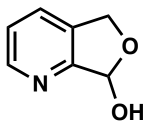

5
^{1}H NMR
(400 MHz, $CDCl_3$
major tautomer drawn)

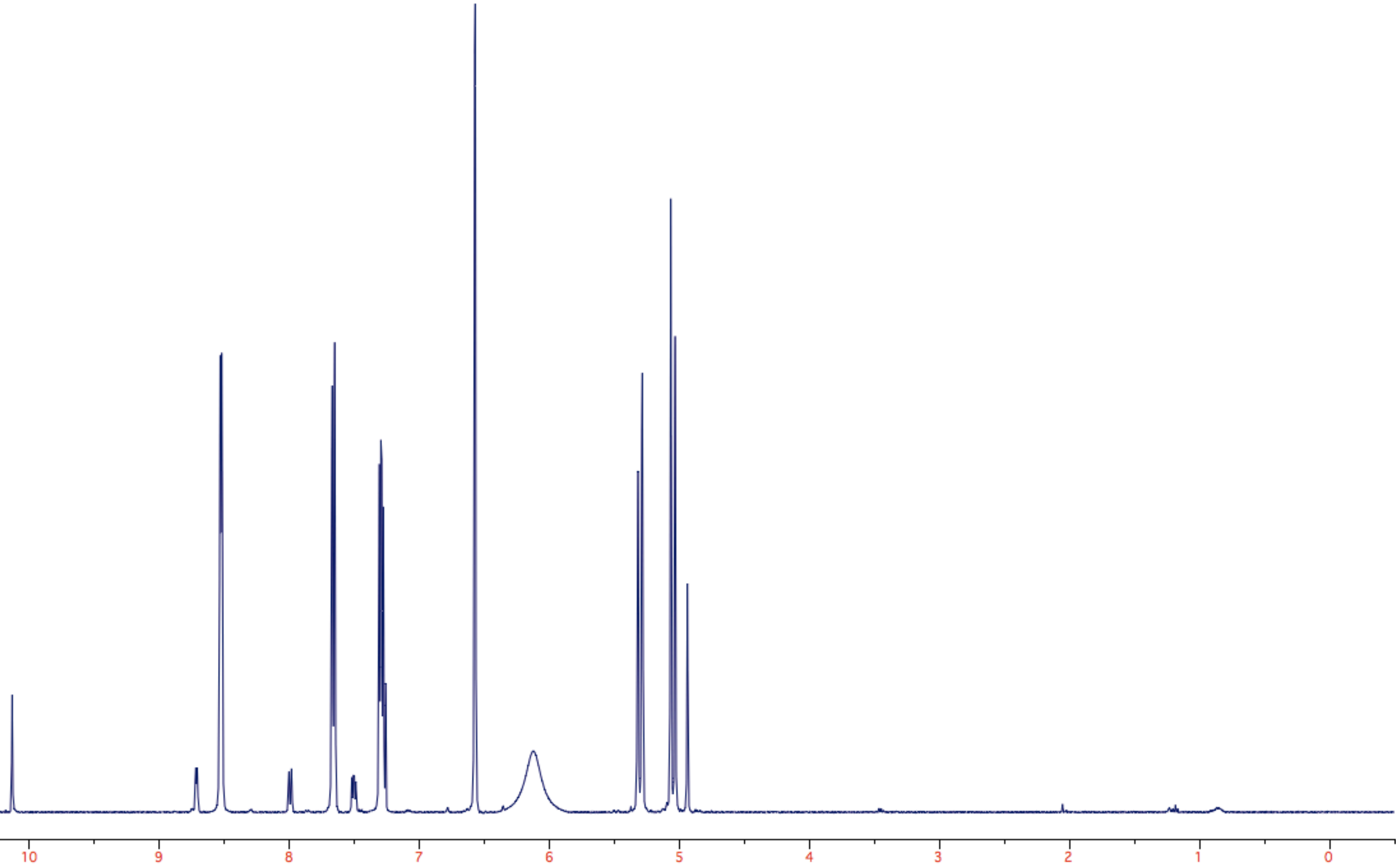

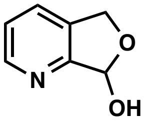

5

^{13}C NMR
(100 MHz, $CDCl_3$
major tautomer drawn)

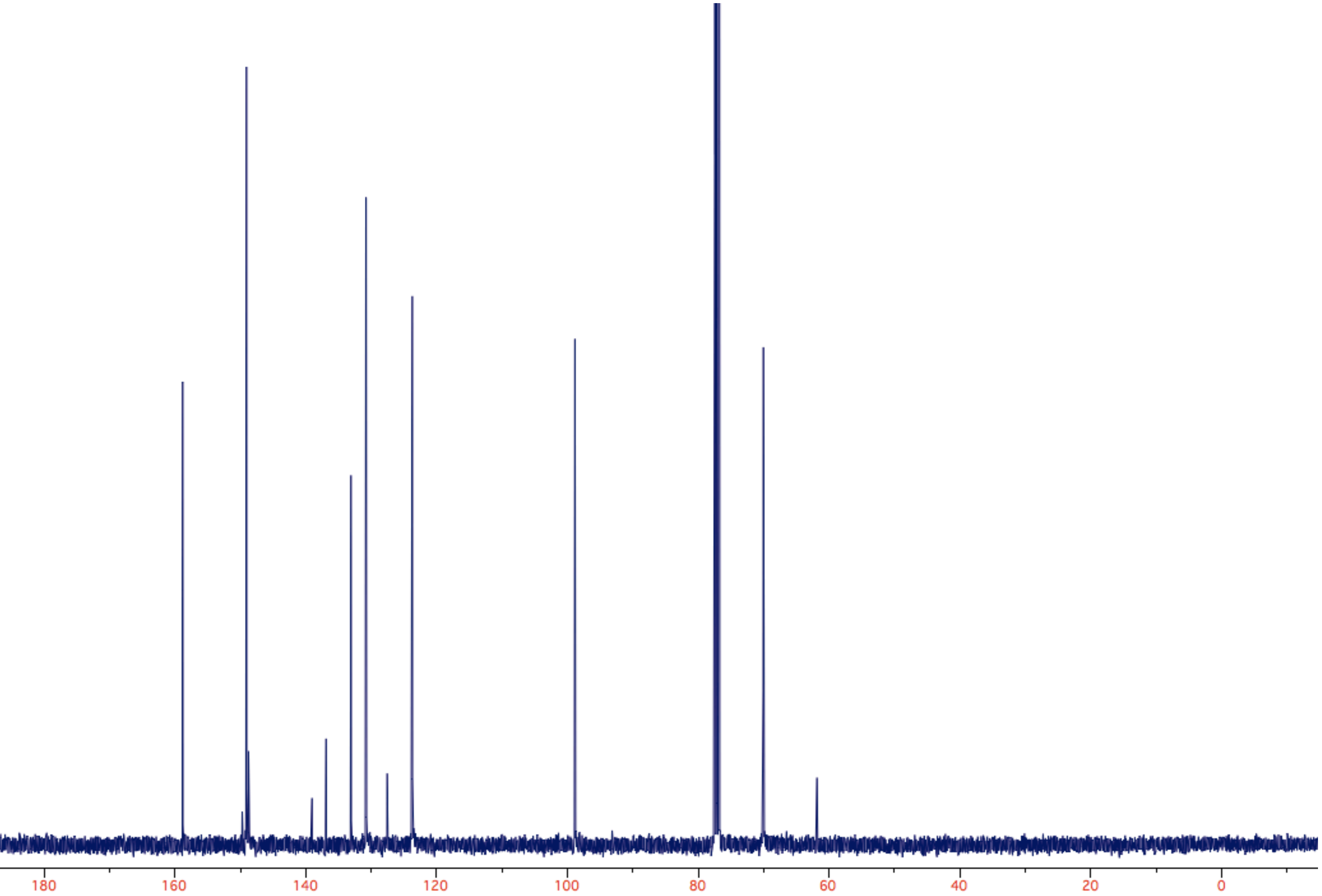

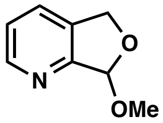

6

^{1}H NMR

(400 MHz, $CDCl_3$)

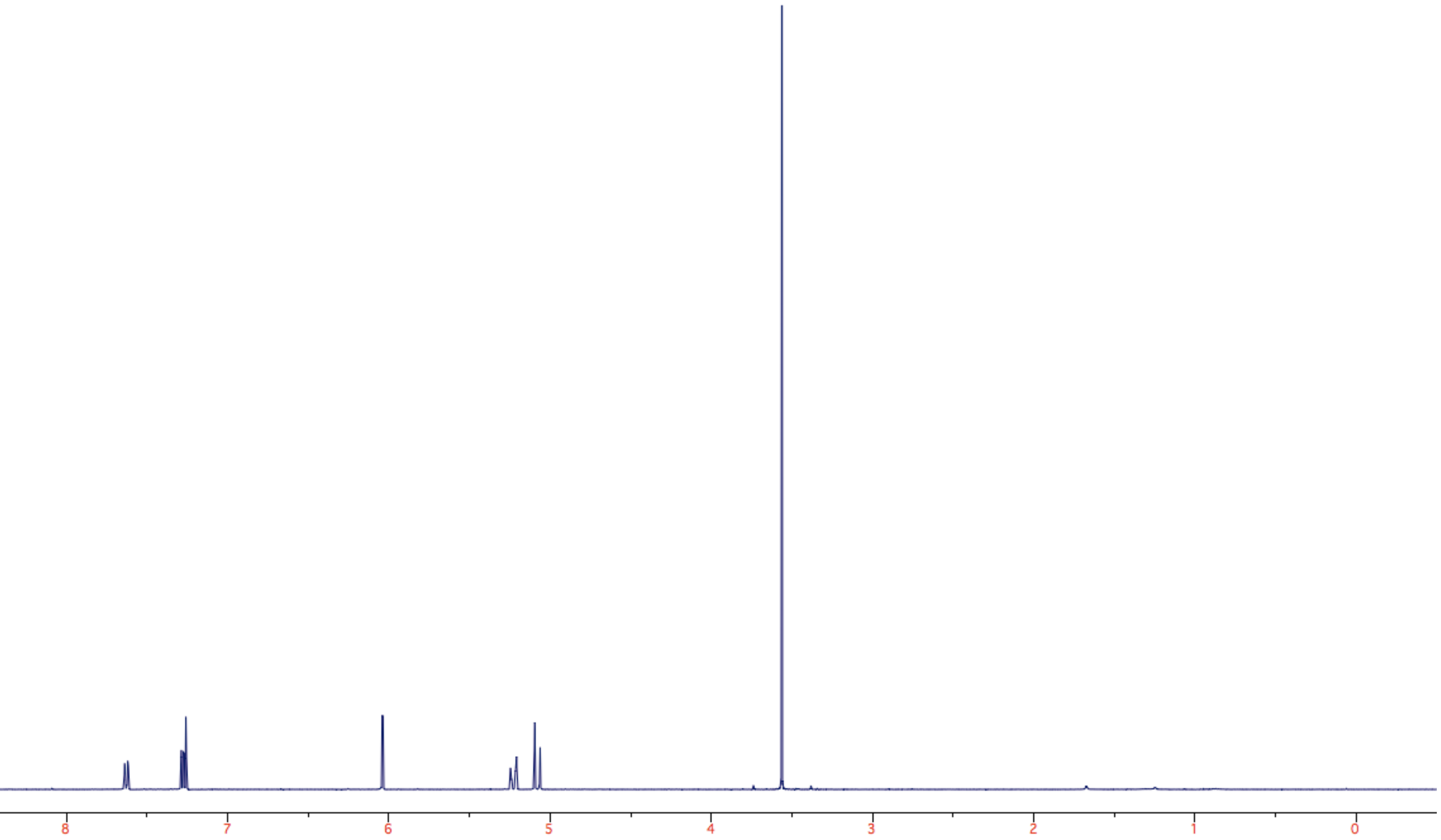

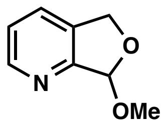

6

^{13}C NMR

(100 MHz, $CDCl_3$)

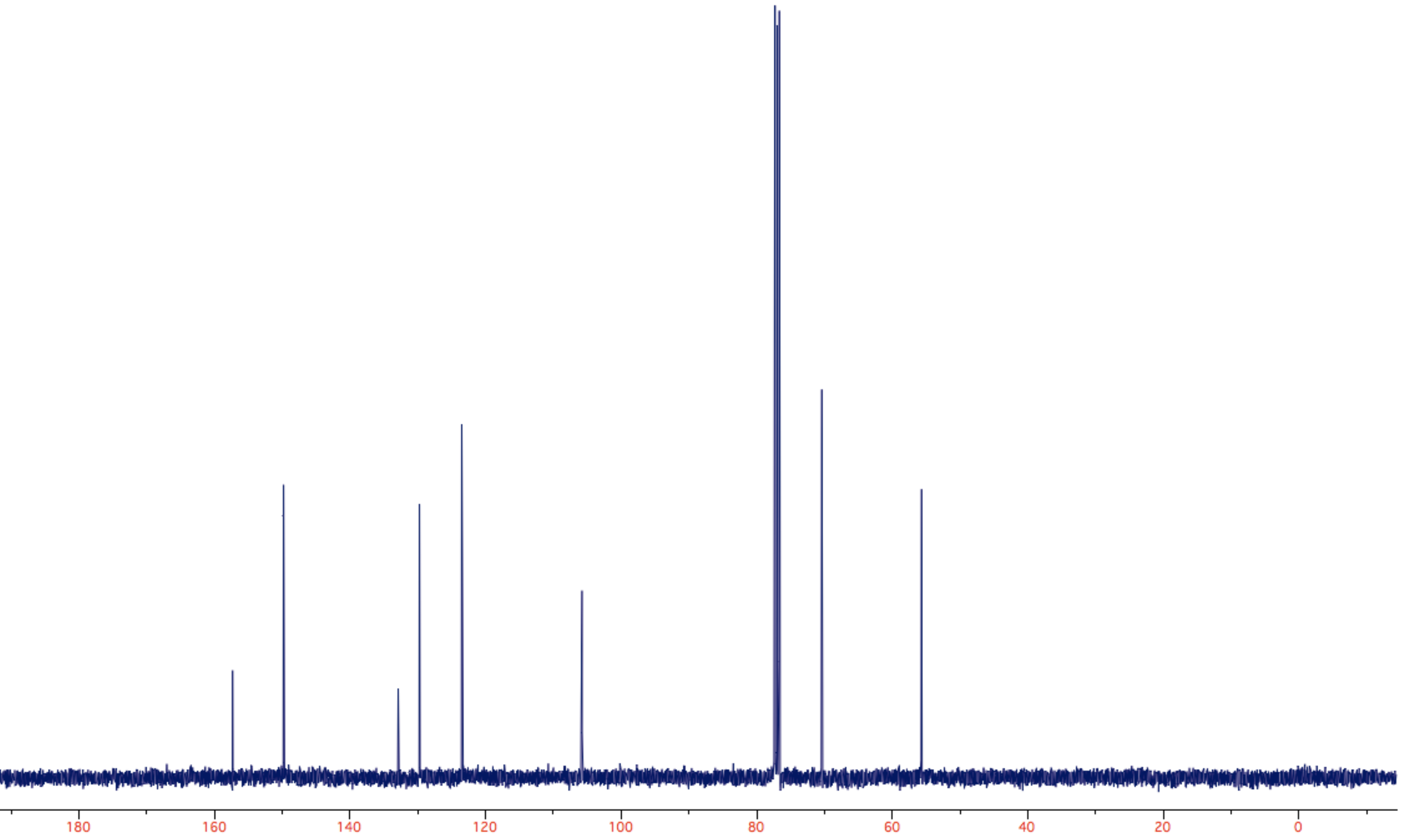

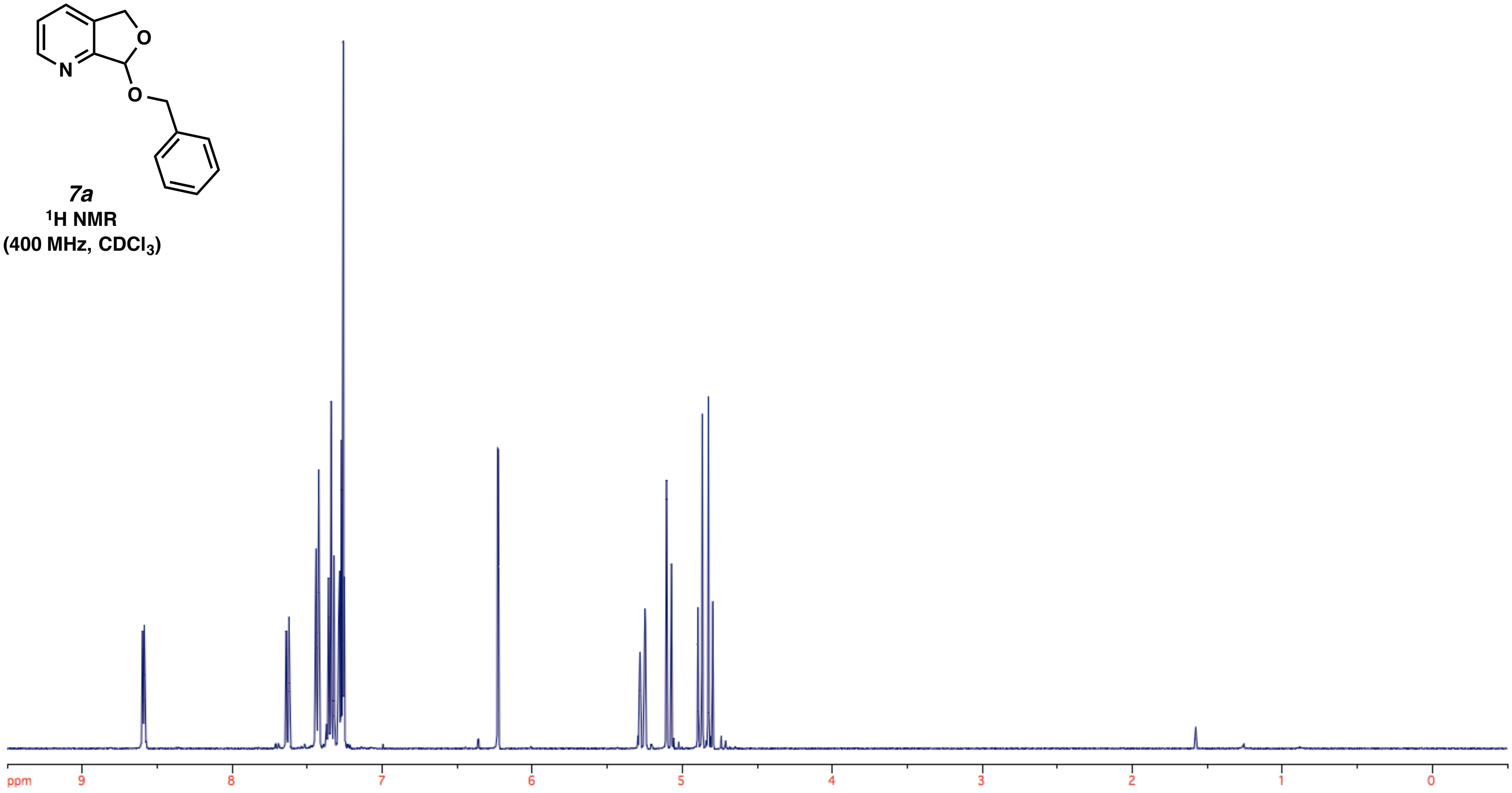

7a

^{1}H NMR

(400 MHz, $CDCl_3$)

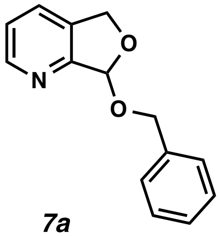

7a
^{13}C NMR
(100 MHz, $CDCl_3$)

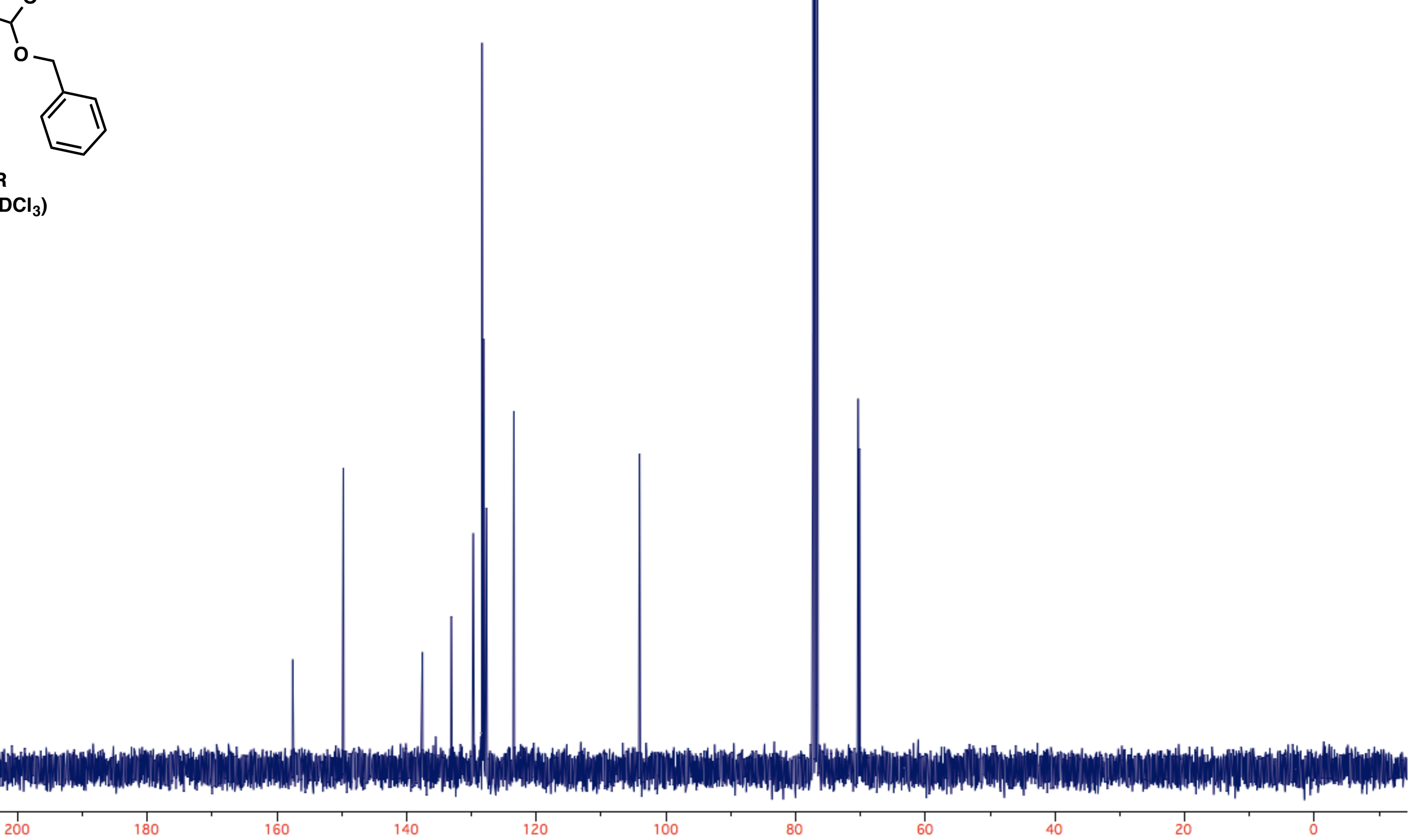

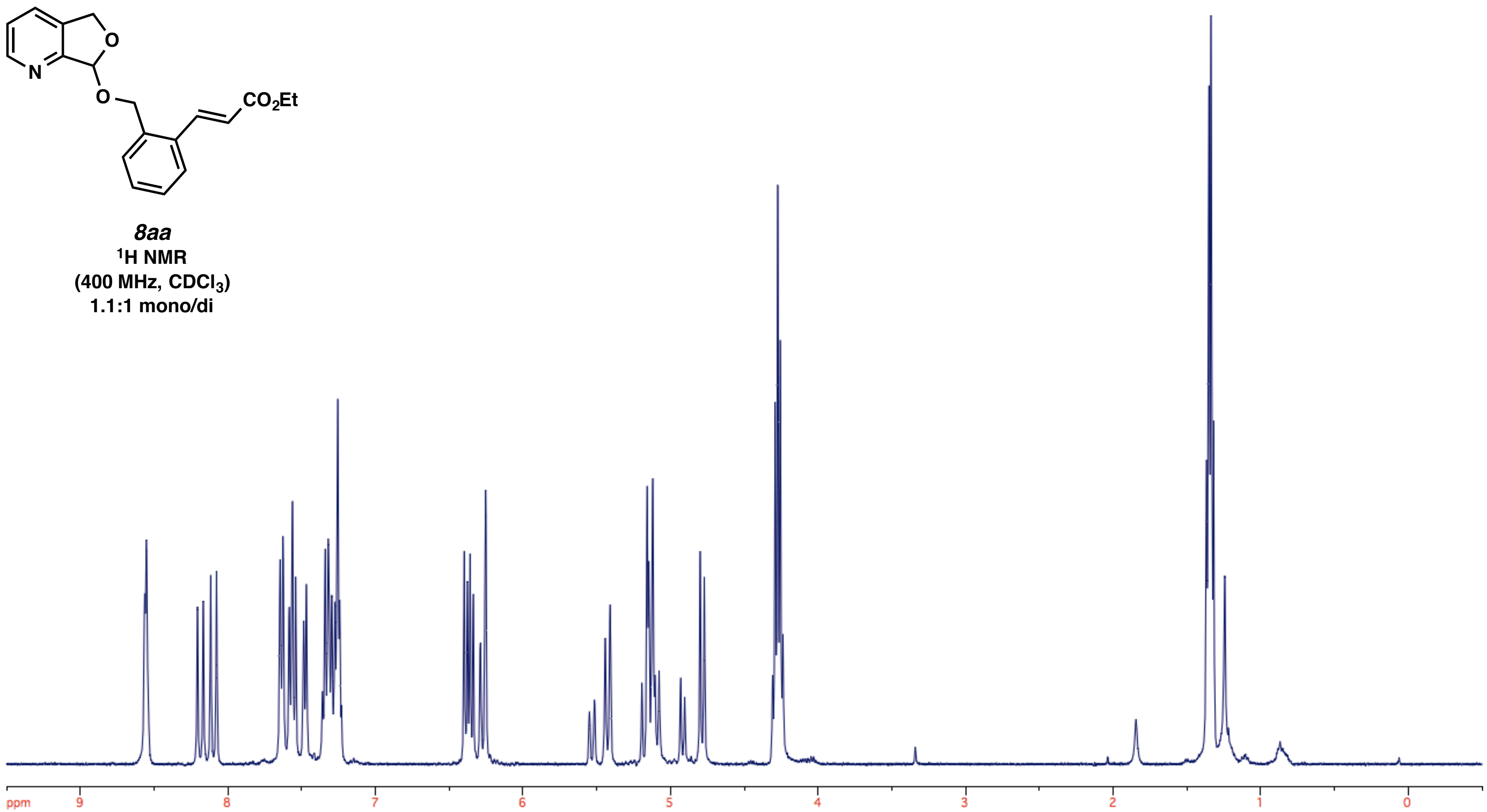

8aa

^{1}H NMR

(400 MHz, $CDCl_3$)

1.1:1 mono/di

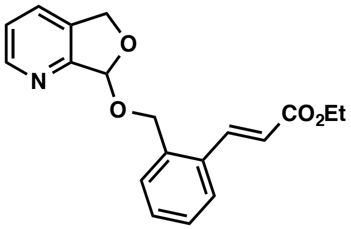

8aa
^{13}C NMR
(100 MHz, $CDCl_3$)
1.1:1 mono/di

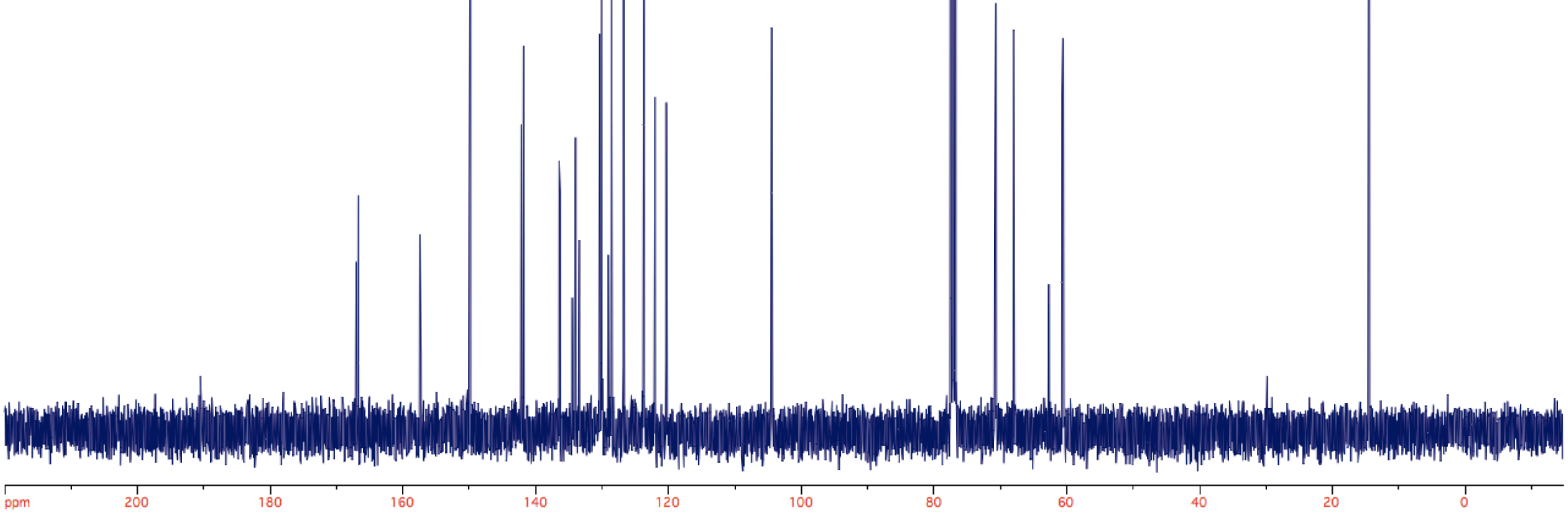

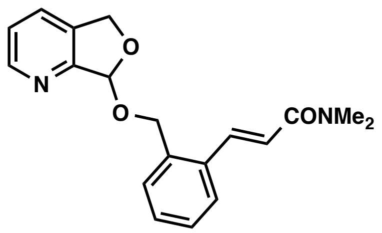

***8ab**$_{mono}$*

^{1}H NMR

(400 MHz, $CDCl_3$)

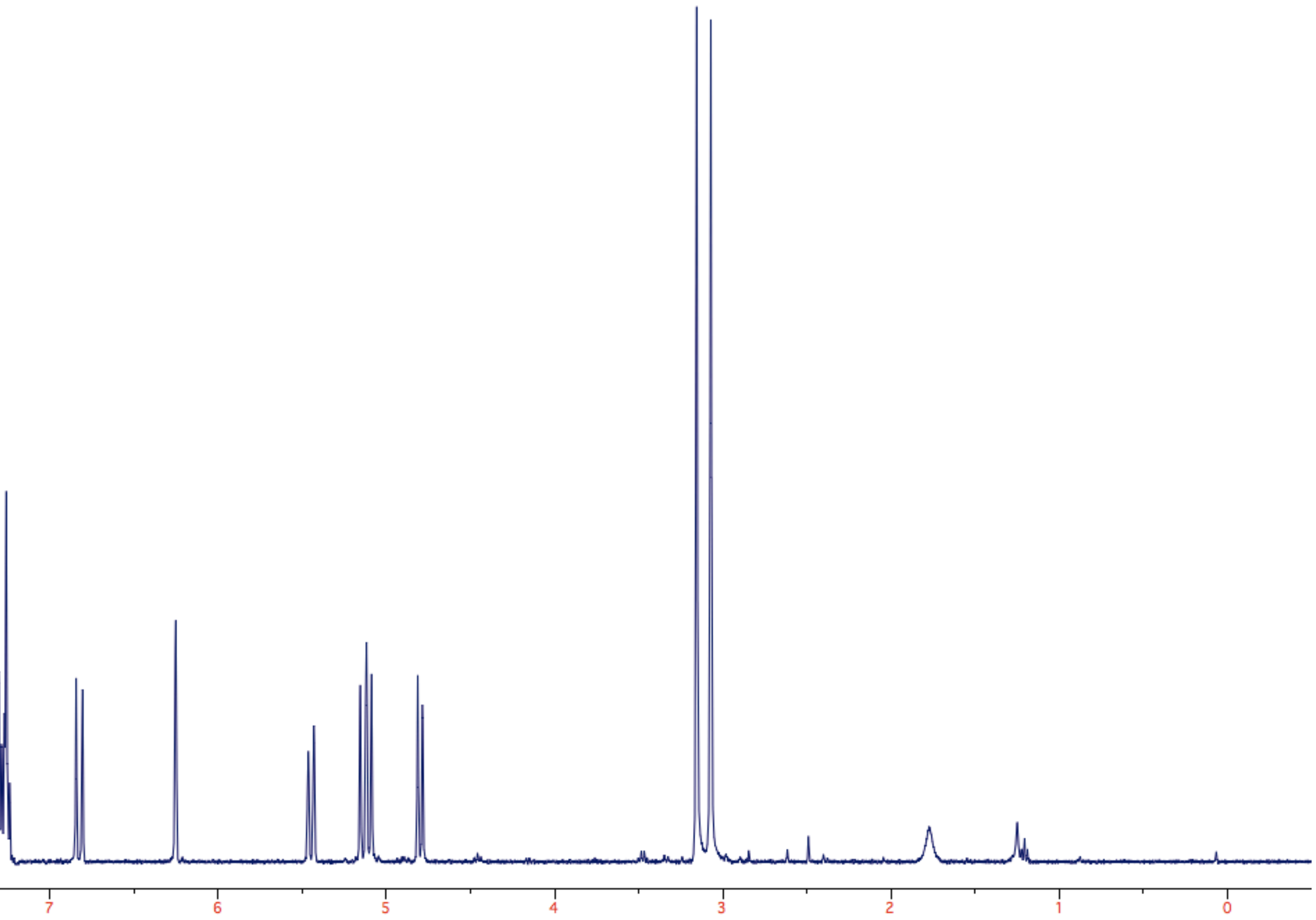

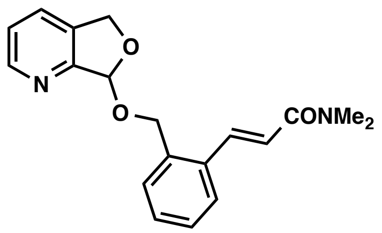

***8ab**$_{mono}$*
^{13}C NMR
(100 MHz, $CDCl_3$)

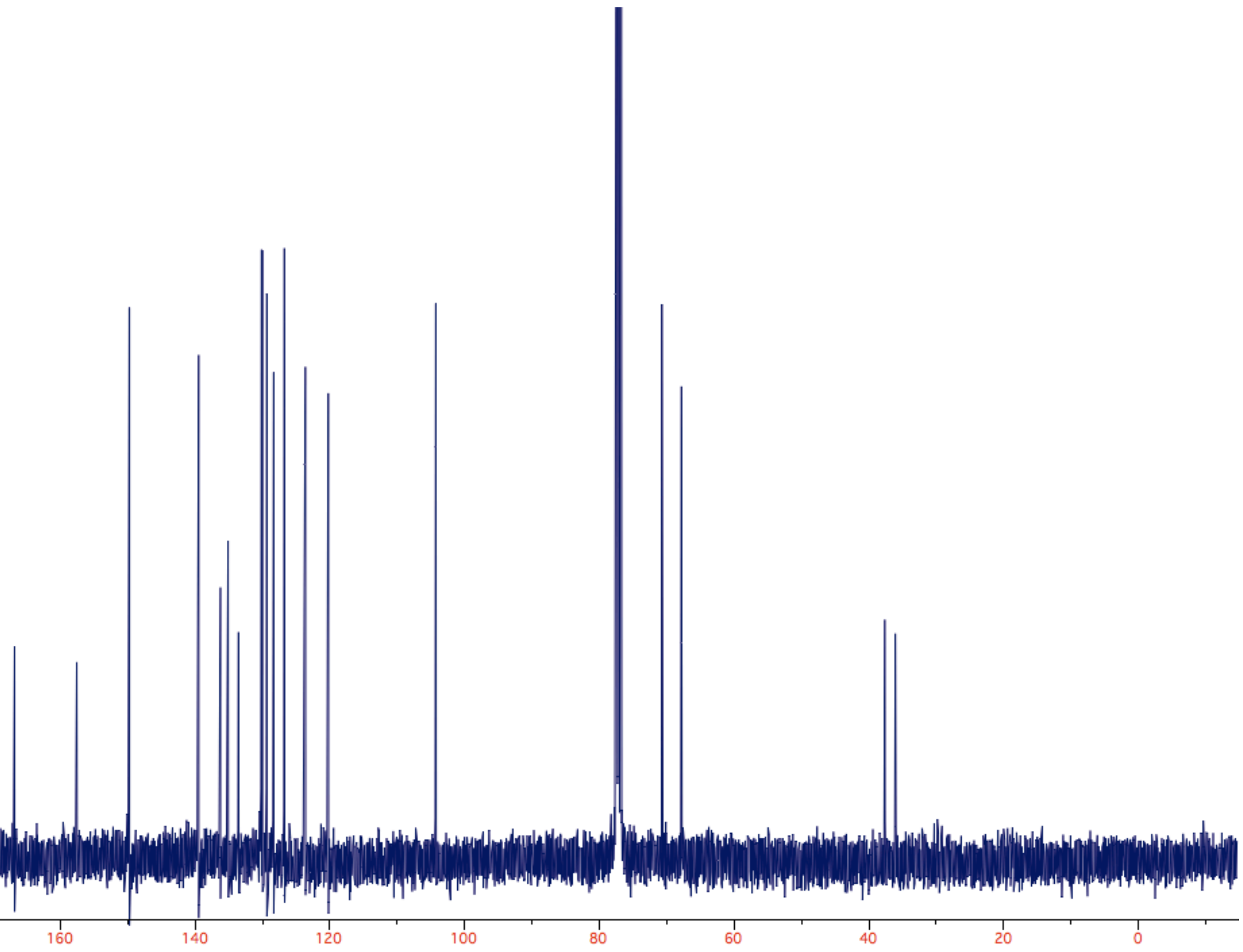

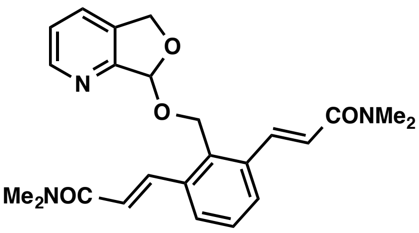

***8ab**$_{di}$*
^{1}H NMR
(400 MHz, $CDCl_3$)

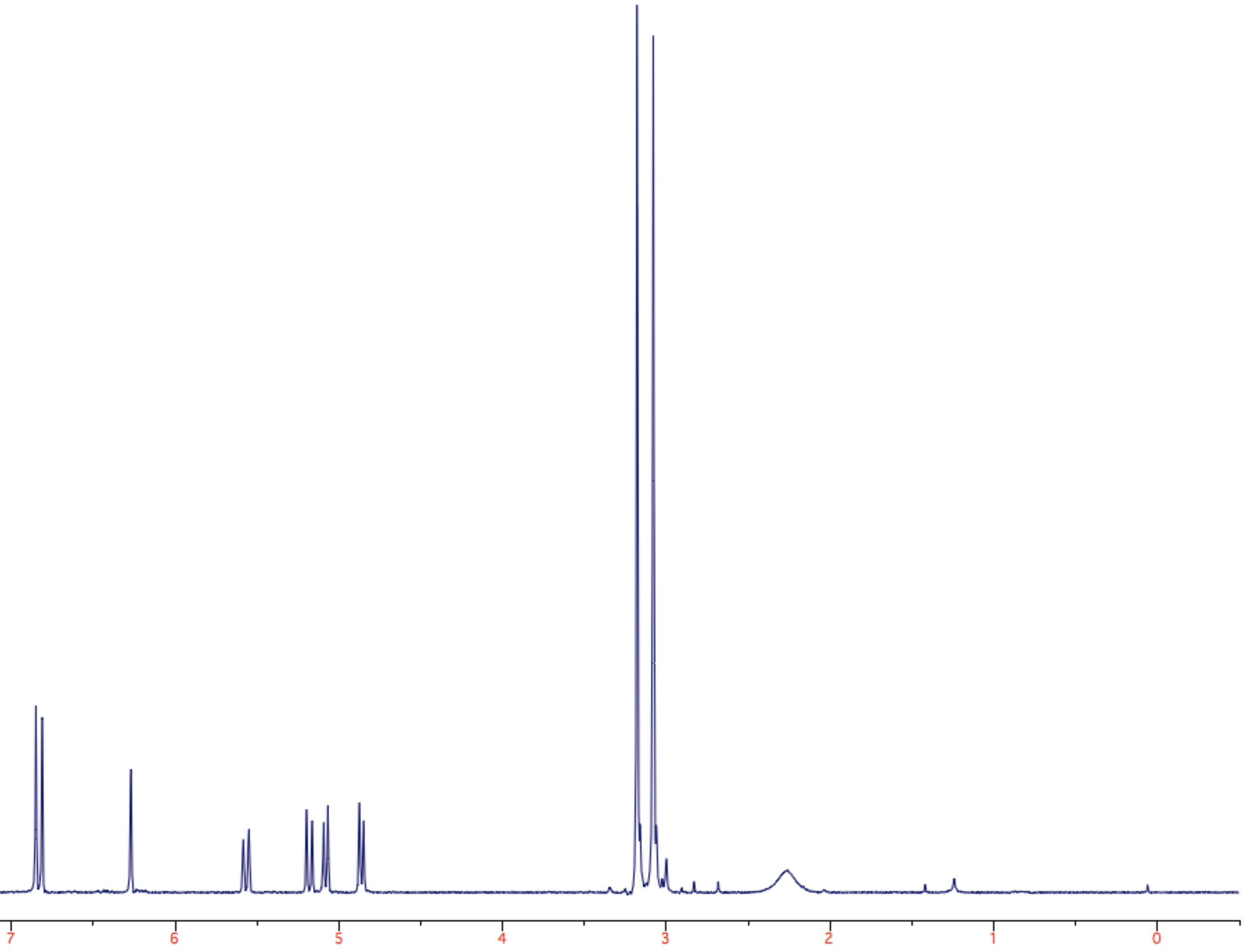

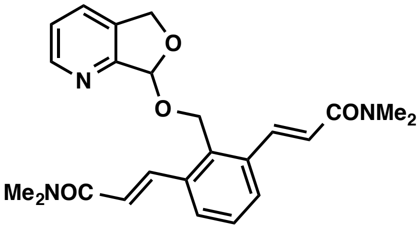

***8ab**$_{di}$*
^{13}C NMR
(100 MHz, $CDCl_3$)

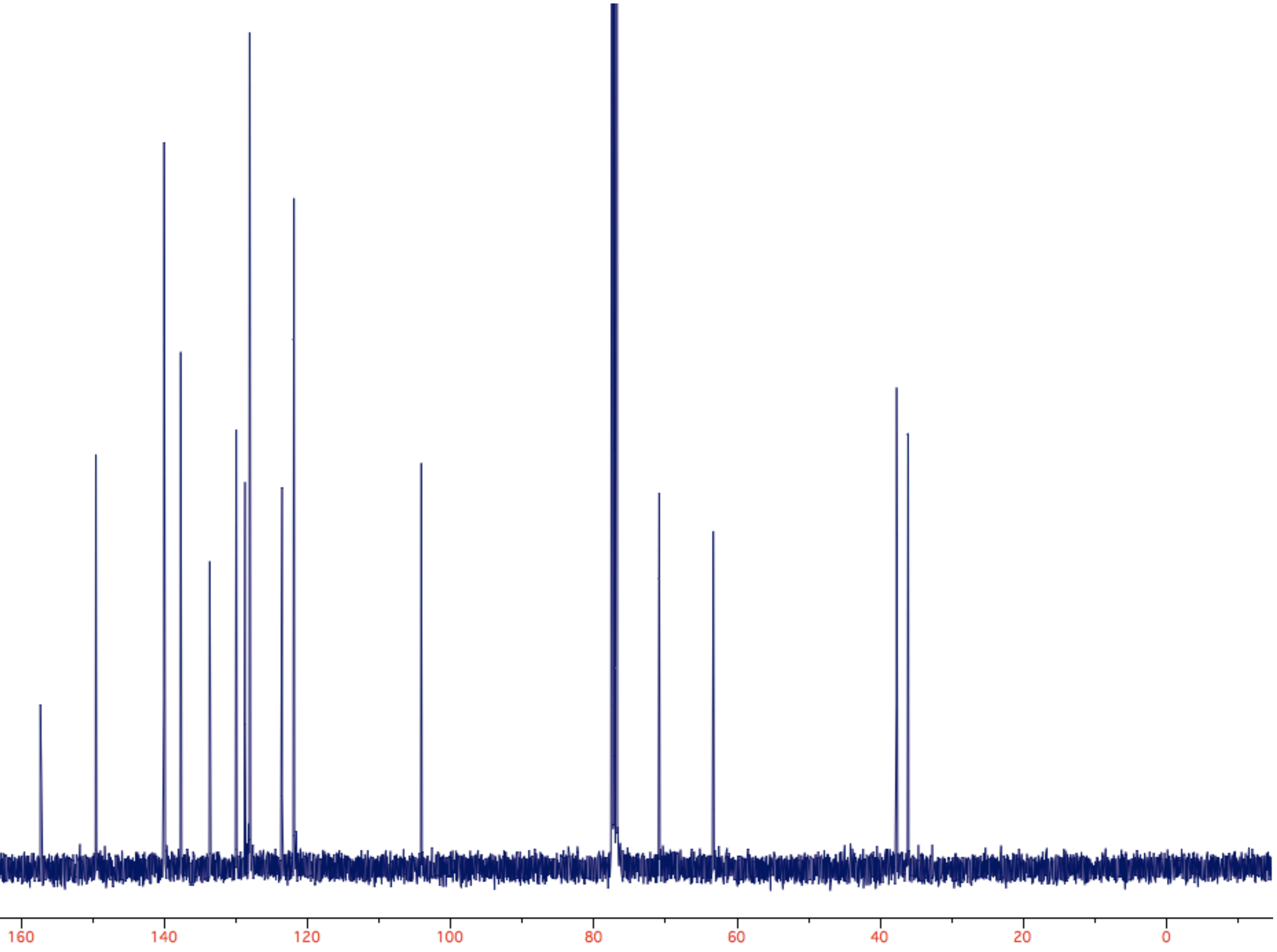

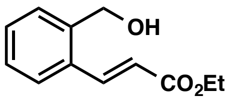

9aa
^{1}H NMR
(400 MHz, $CDCl_3$)
12:1 mono/di

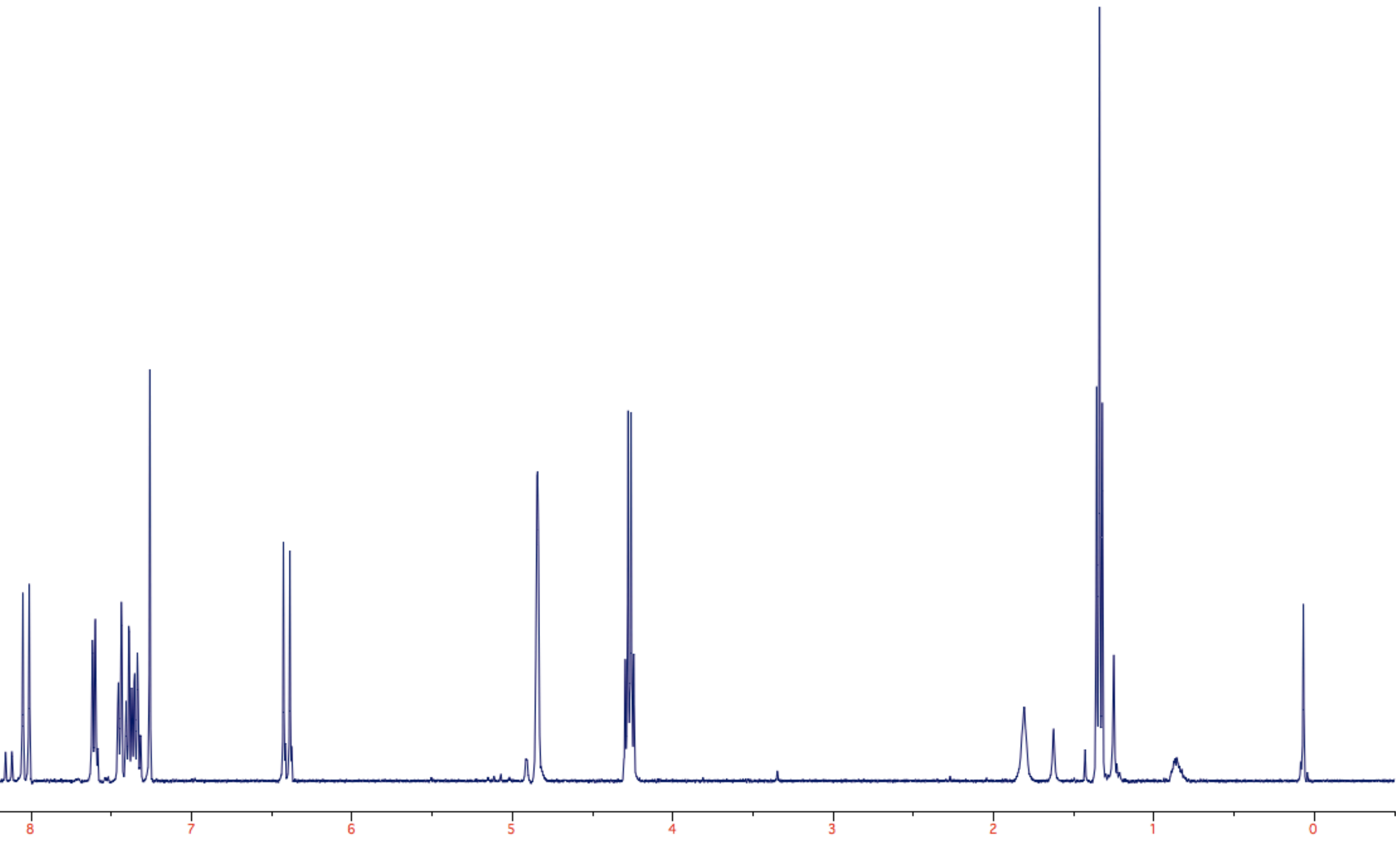

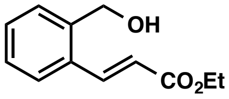

9aa
^{13}C NMR
(125 MHz, $CDCl_3$)
12:1 mono/di

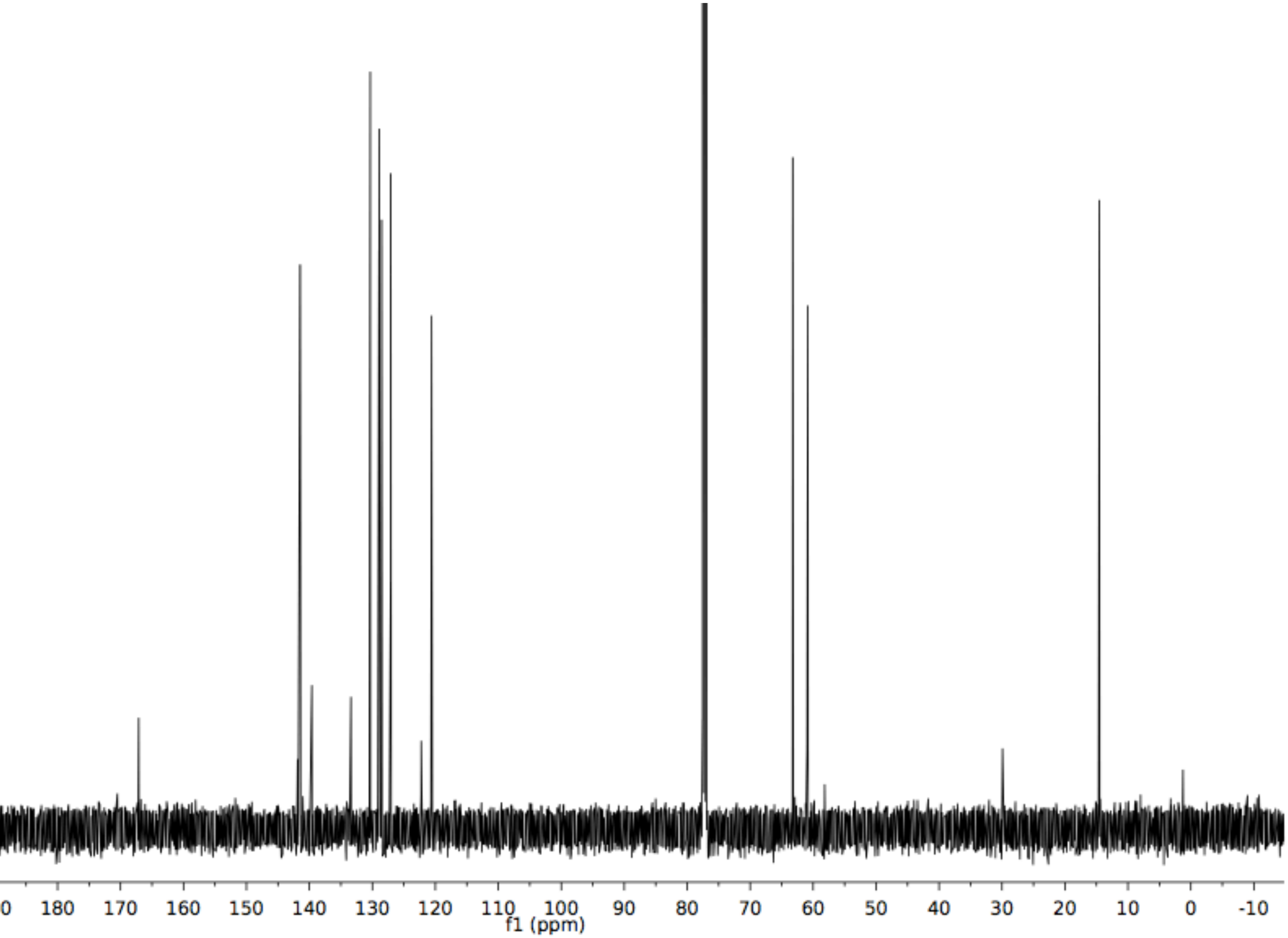

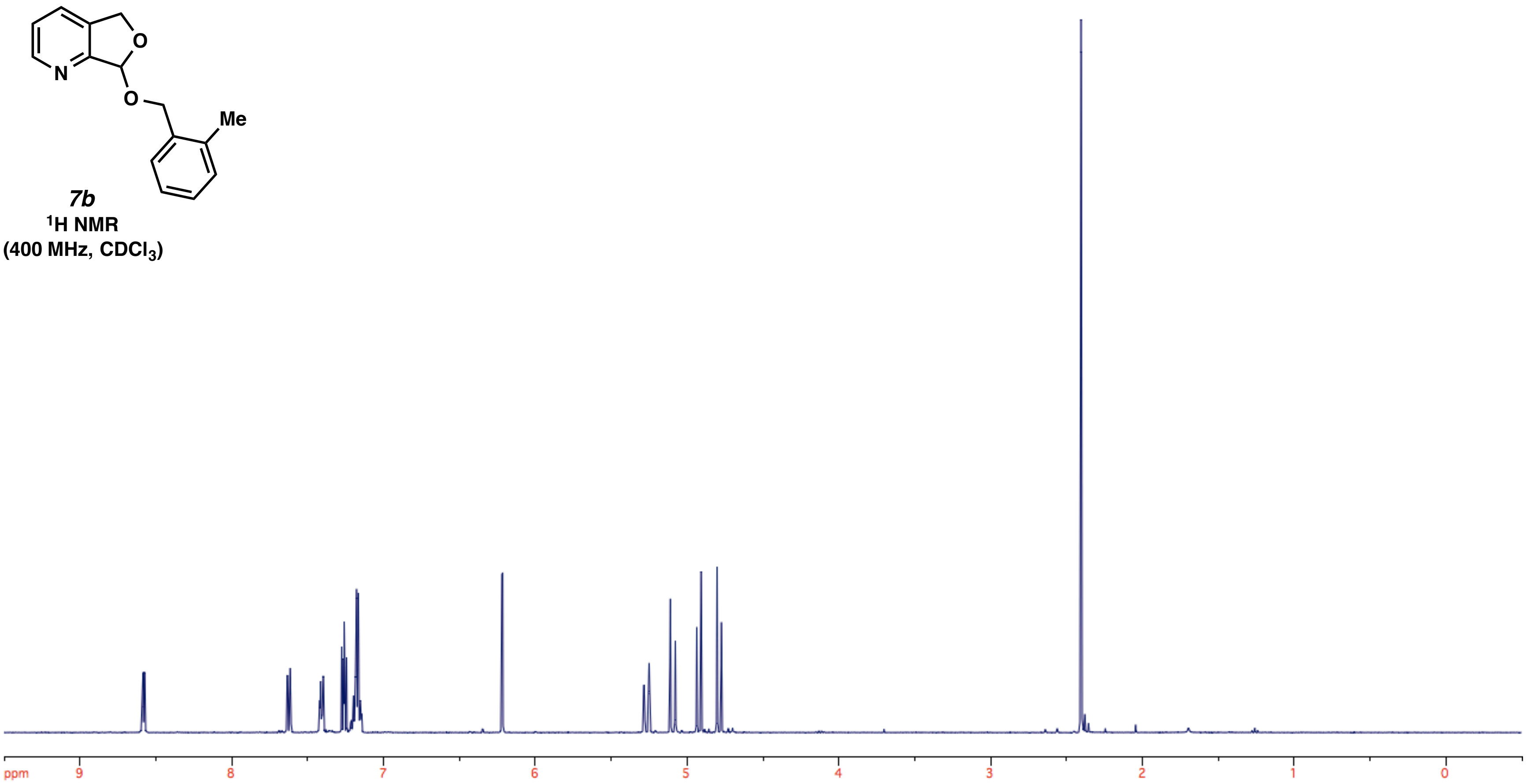

7b
^{1}H NMR
(400 MHz, $CDCl_3$)

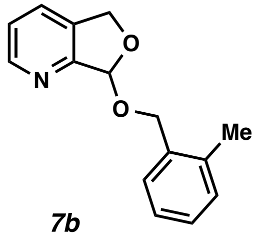

7b
^{13}C NMR
(100 MHz, $CDCl_3$)

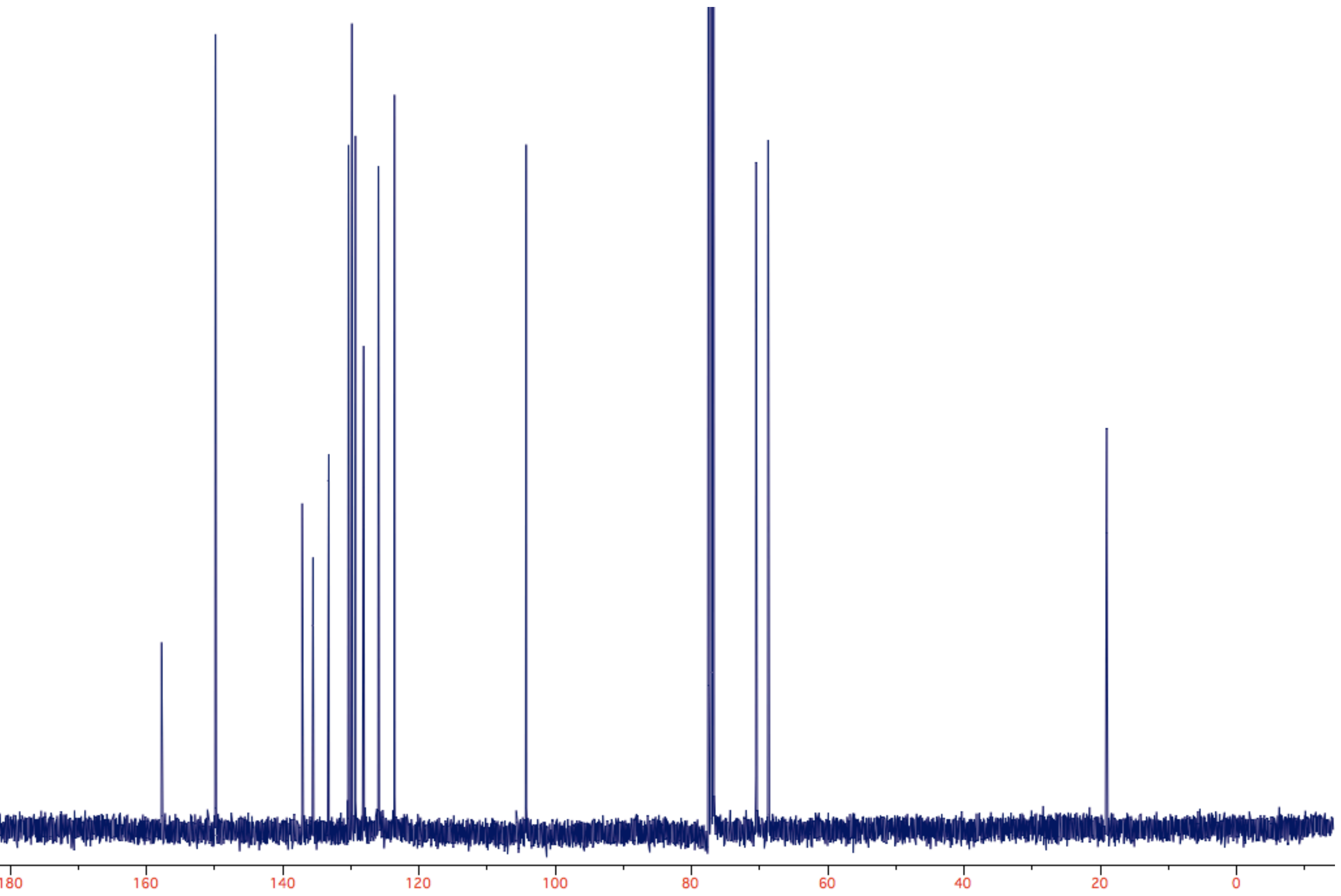

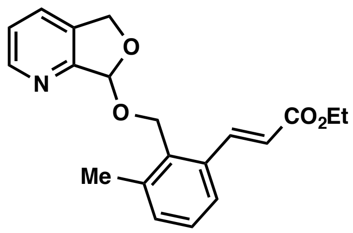

8ba
^{1}H NMR
(400 MHz, $CDCl_3$)

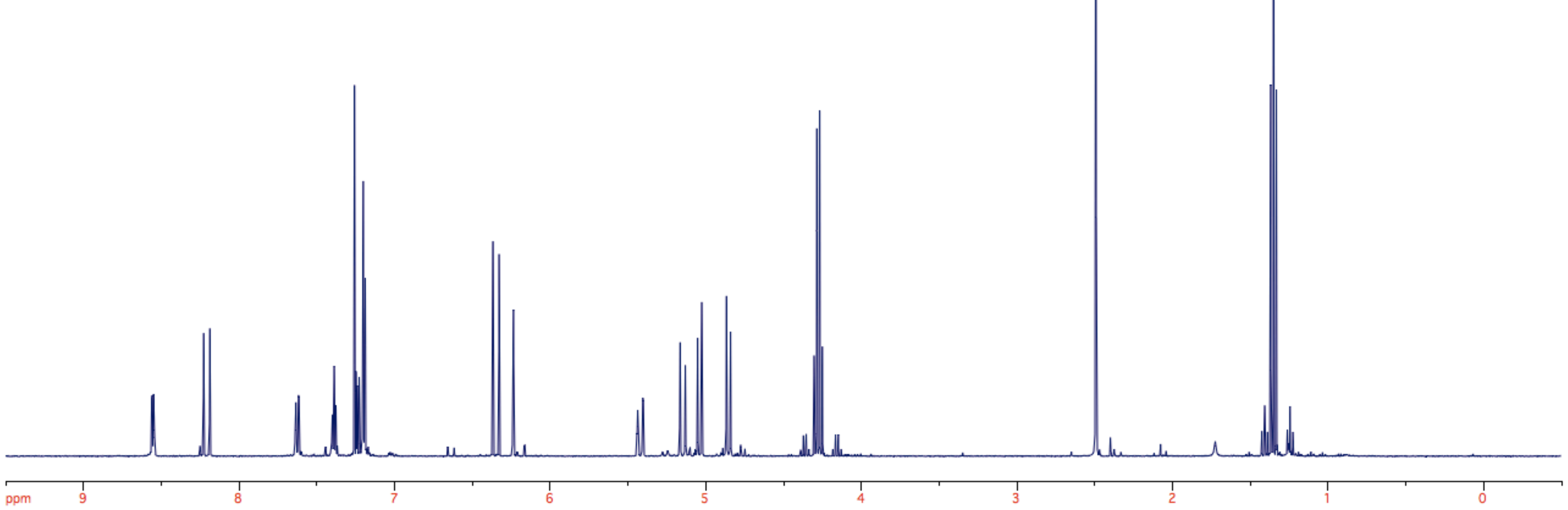

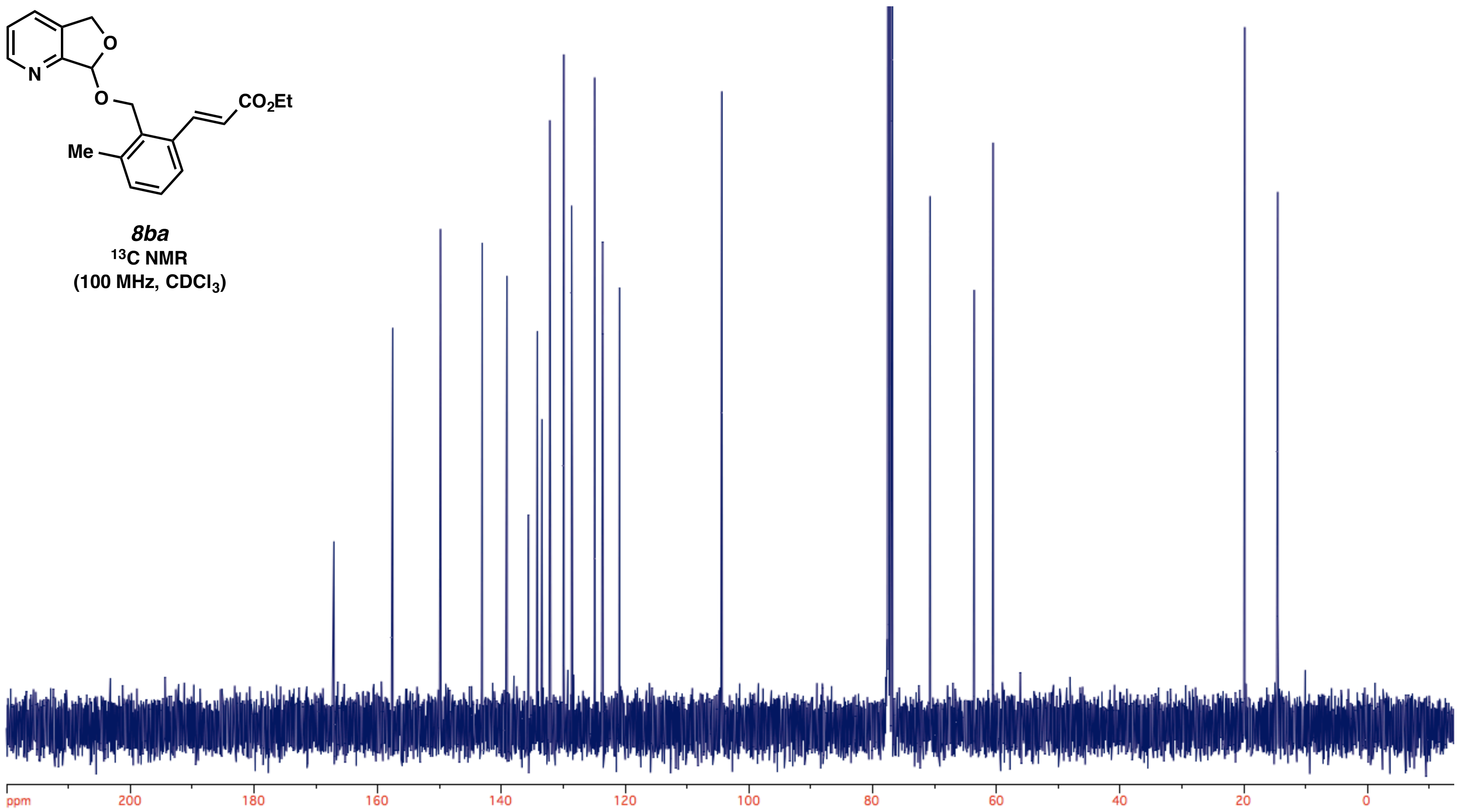

8ba
^{13}C NMR
(100 MHz, $CDCl_3$)

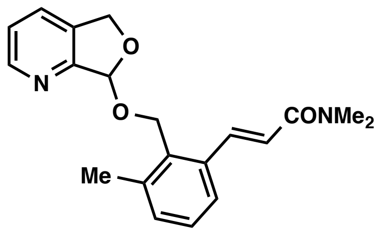

8bb
^{1}H NMR
(400 MHz, $CDCl_3$)

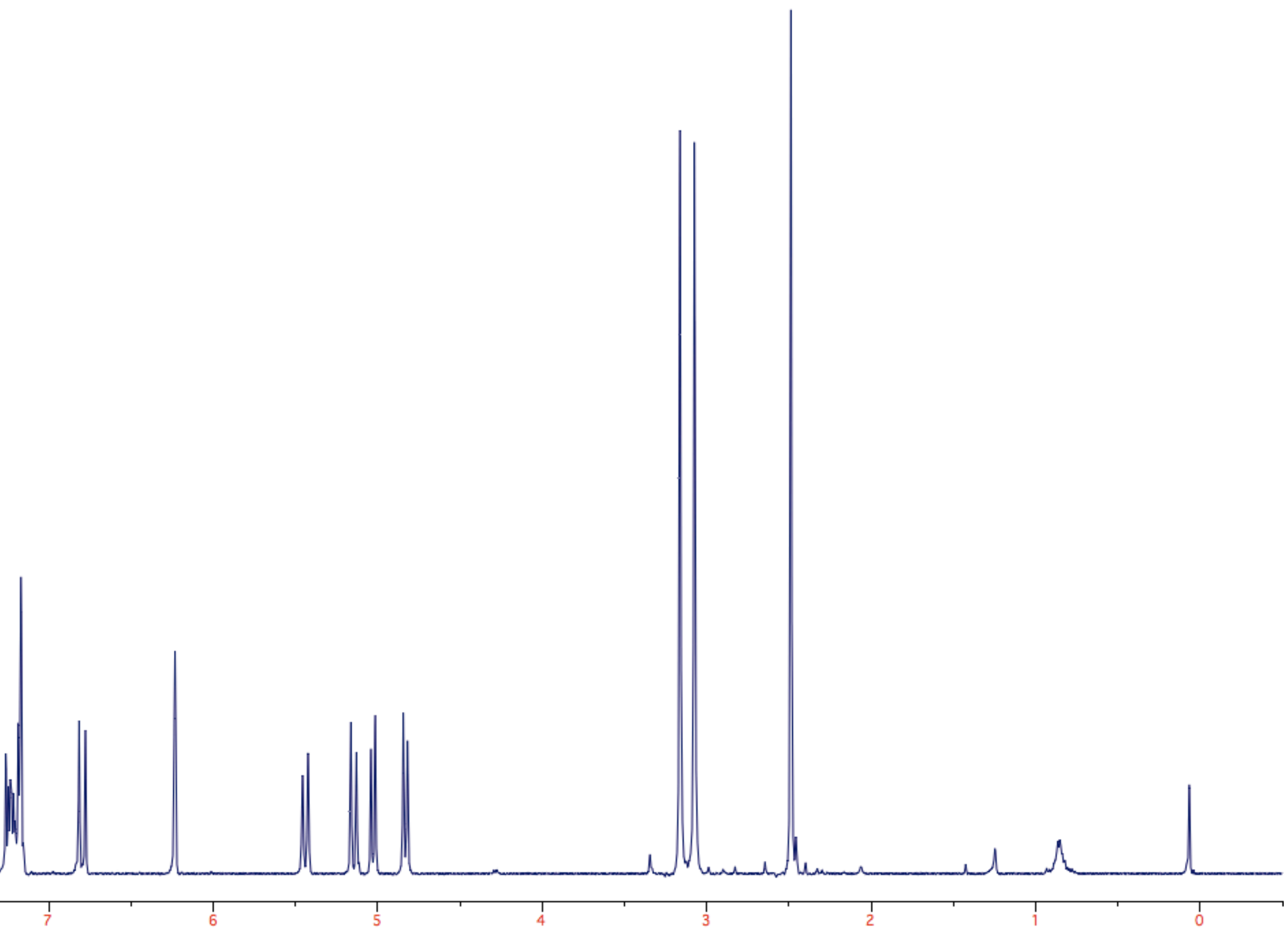

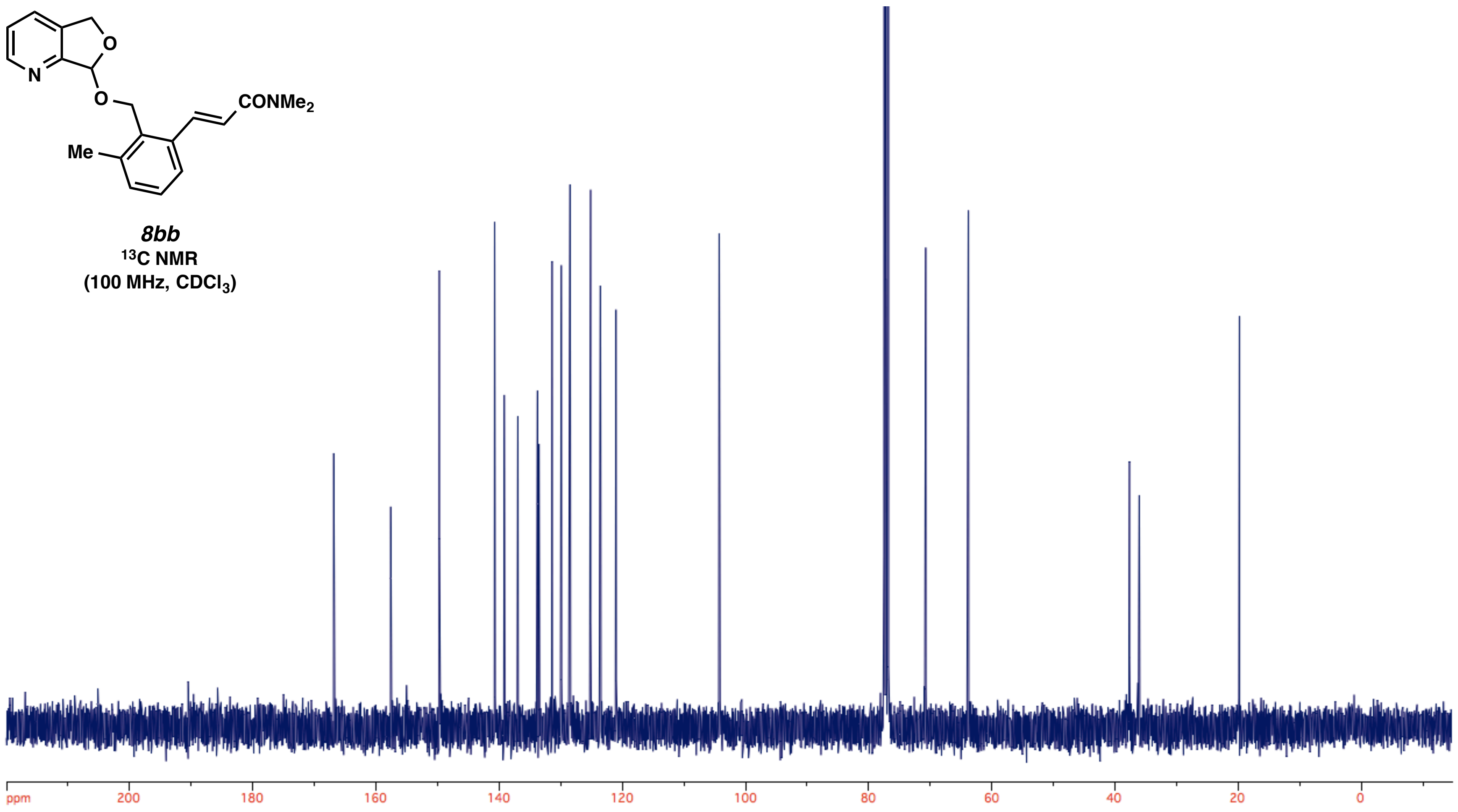
N
O
O
CONMe$_2$
Me
8bb
^{13}C NMR
(100 MHz, CDCl$_3$)
ppm
200
180
160
140
120
100
80
60
40
20
0

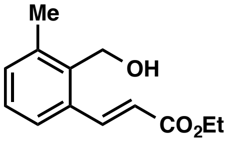

9ba

^{1}H NMR

(400 MHz, $CDCl_3$)

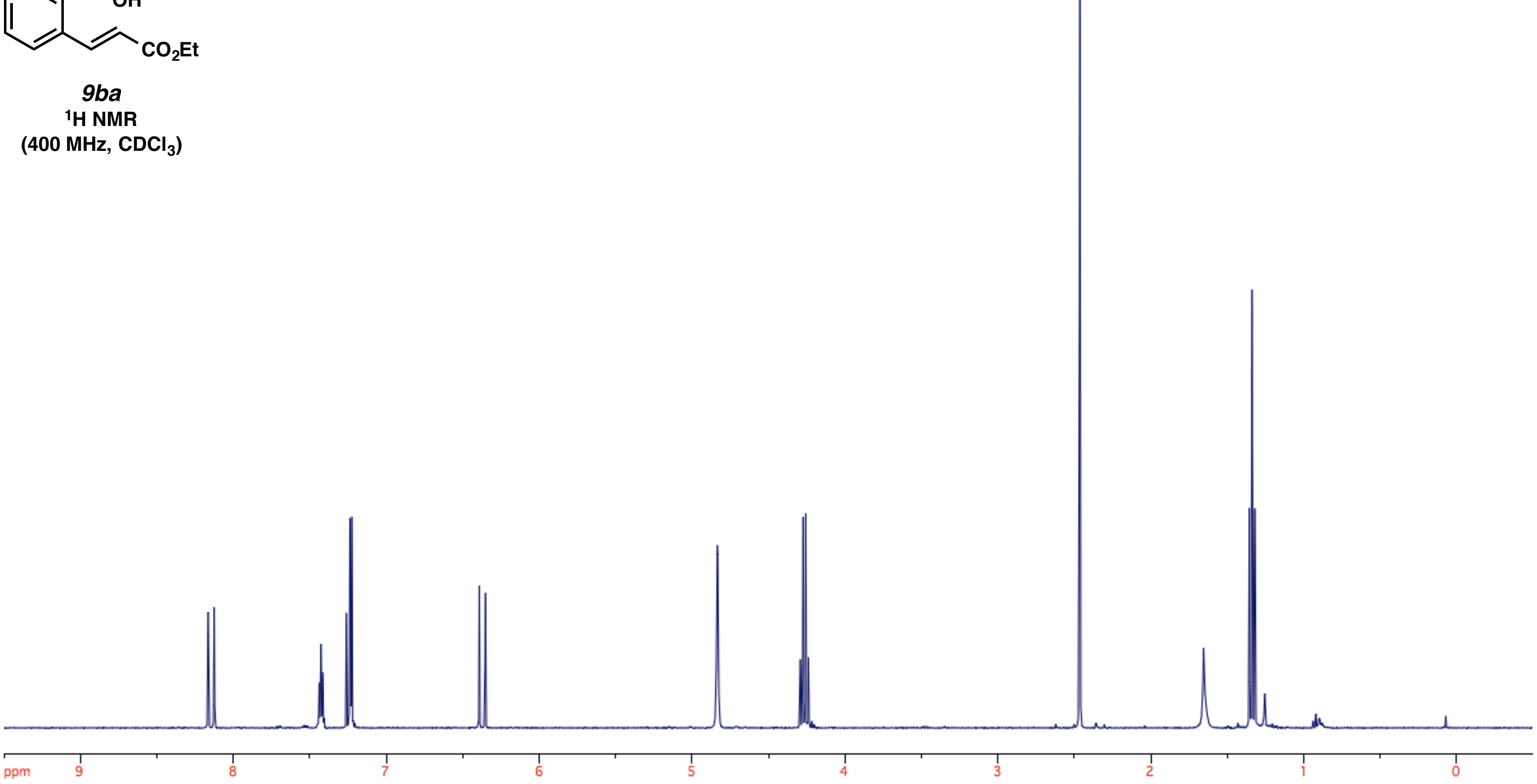

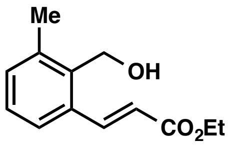

9ba
^{13}C NMR
(100 MHz, $CDCl_3$)

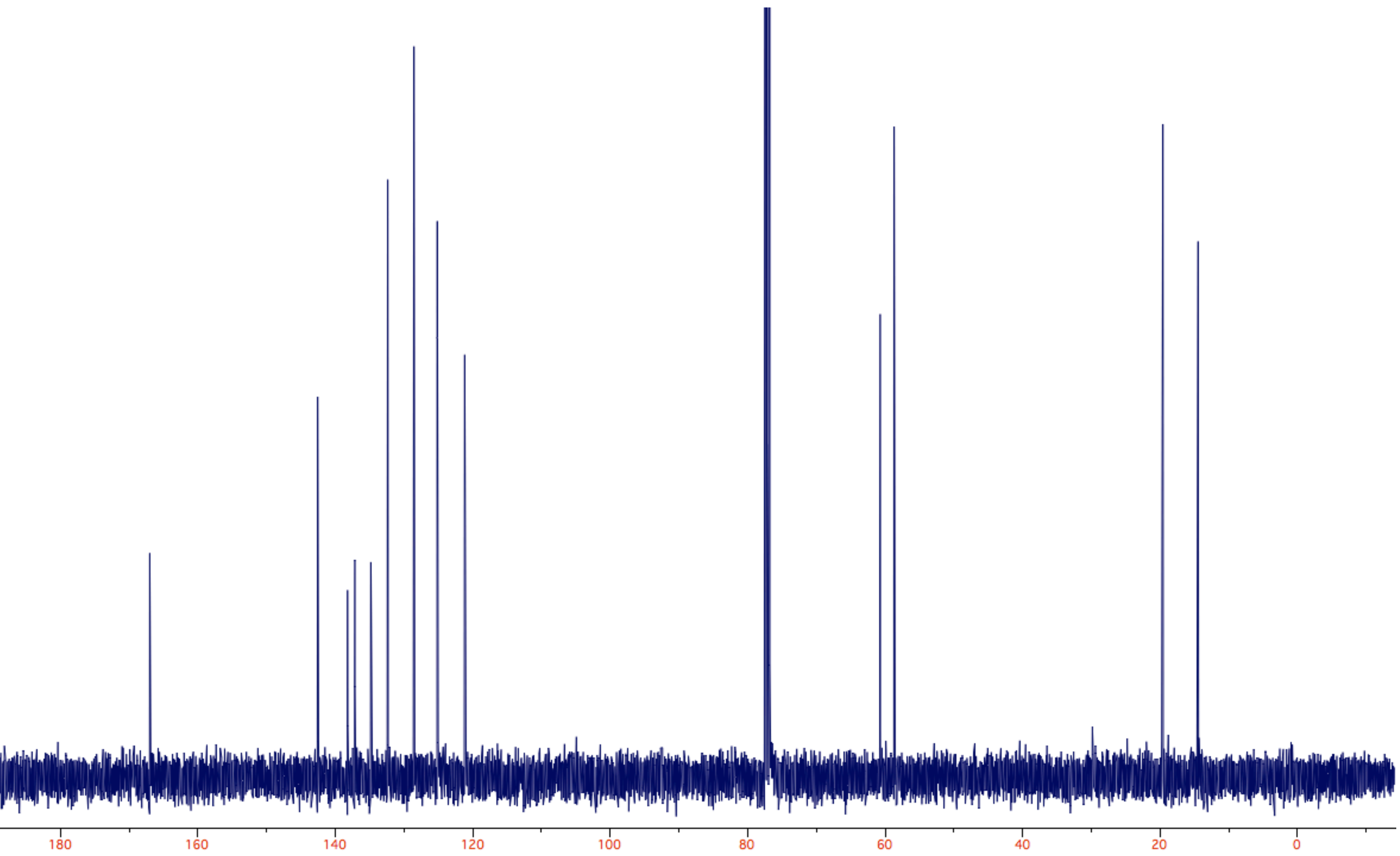

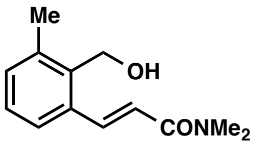

9bb
^{1}H NMR
(400 MHz, $CDCl_3$)

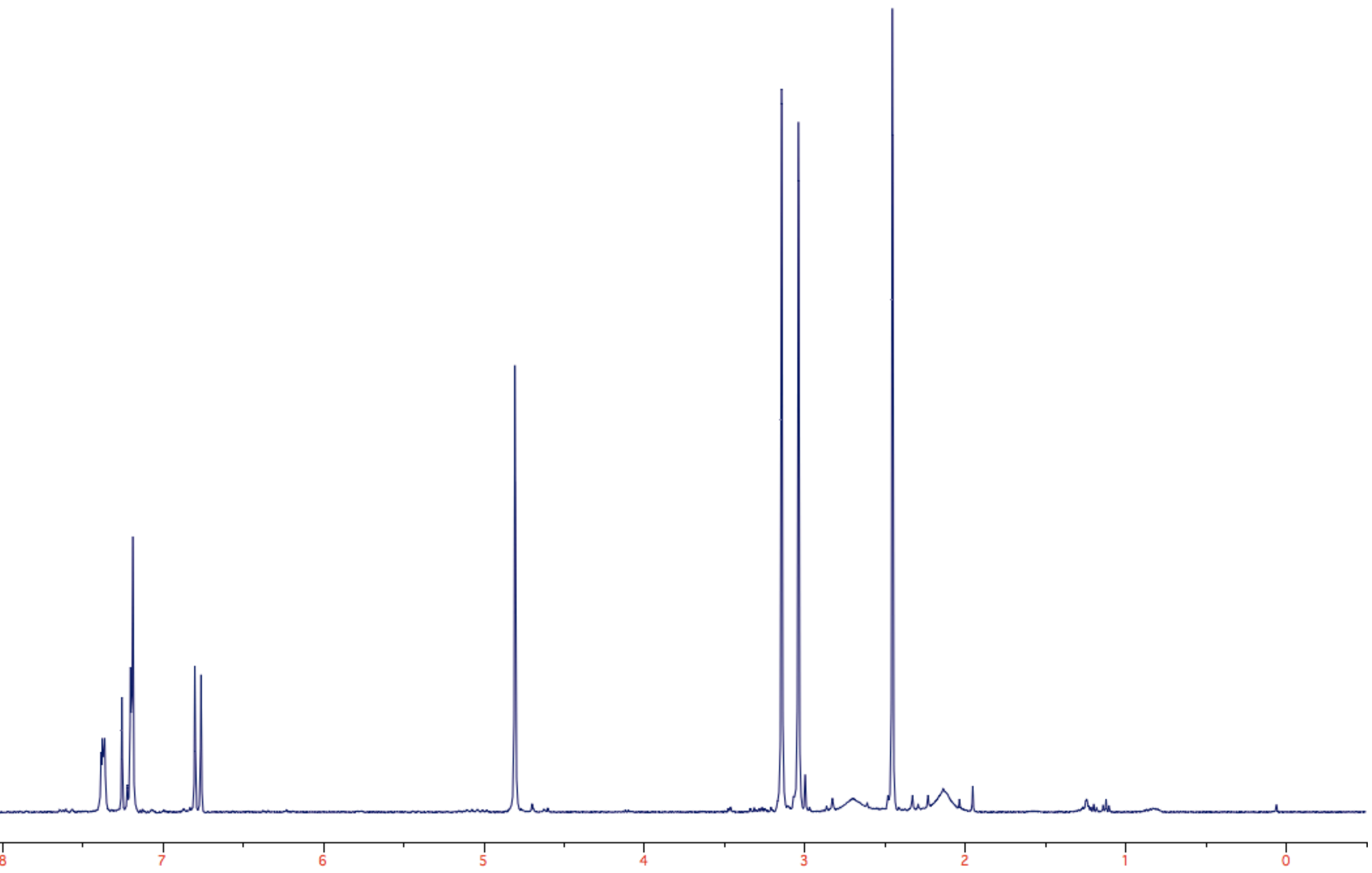

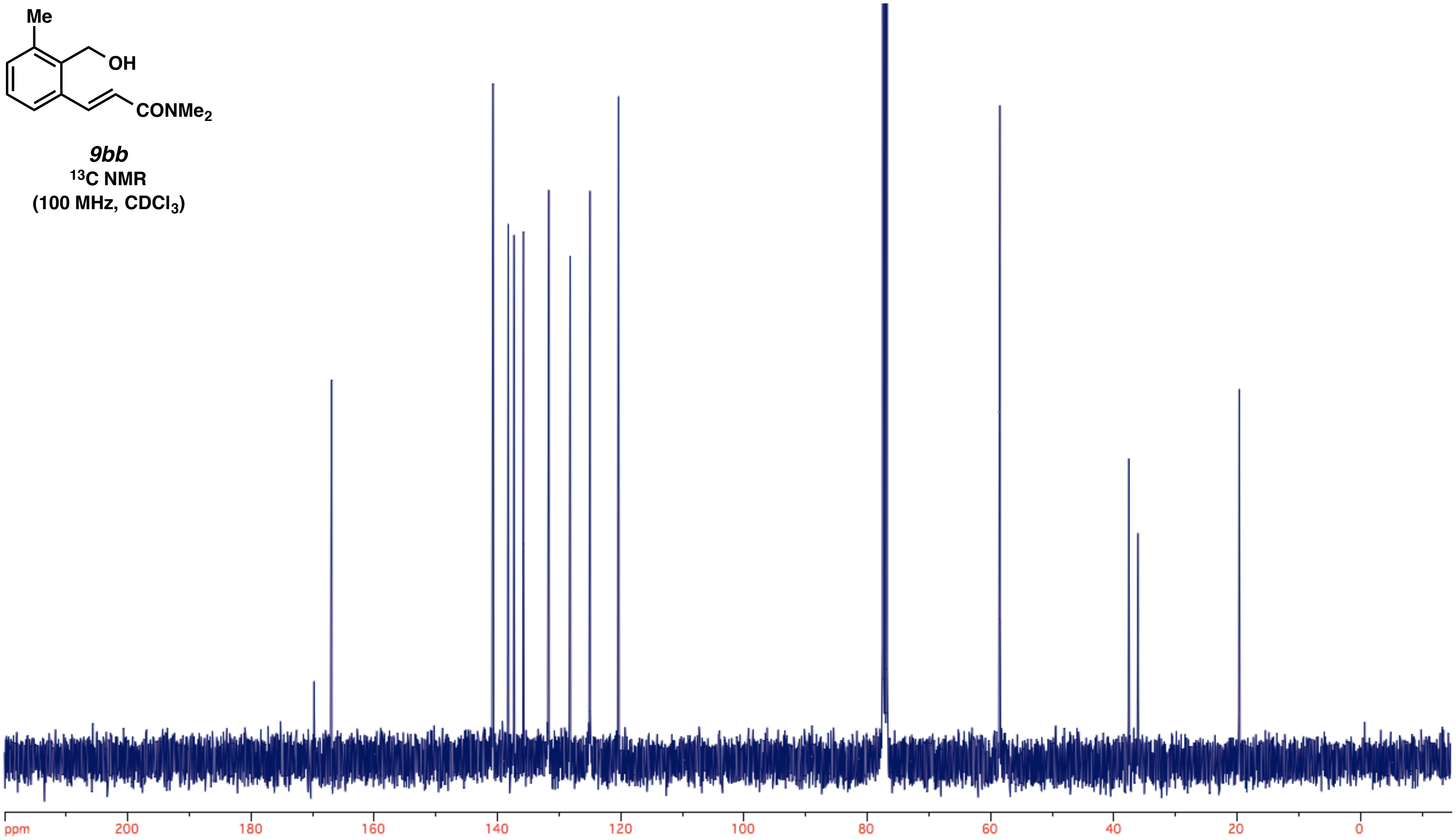

9bb
^{13}C NMR
(100 MHz, $CDCl_3$)

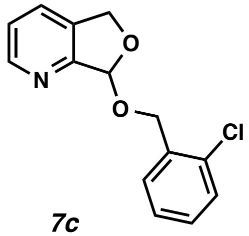

7c
^{1}H NMR
(400 MHz, $CDCl_3$)

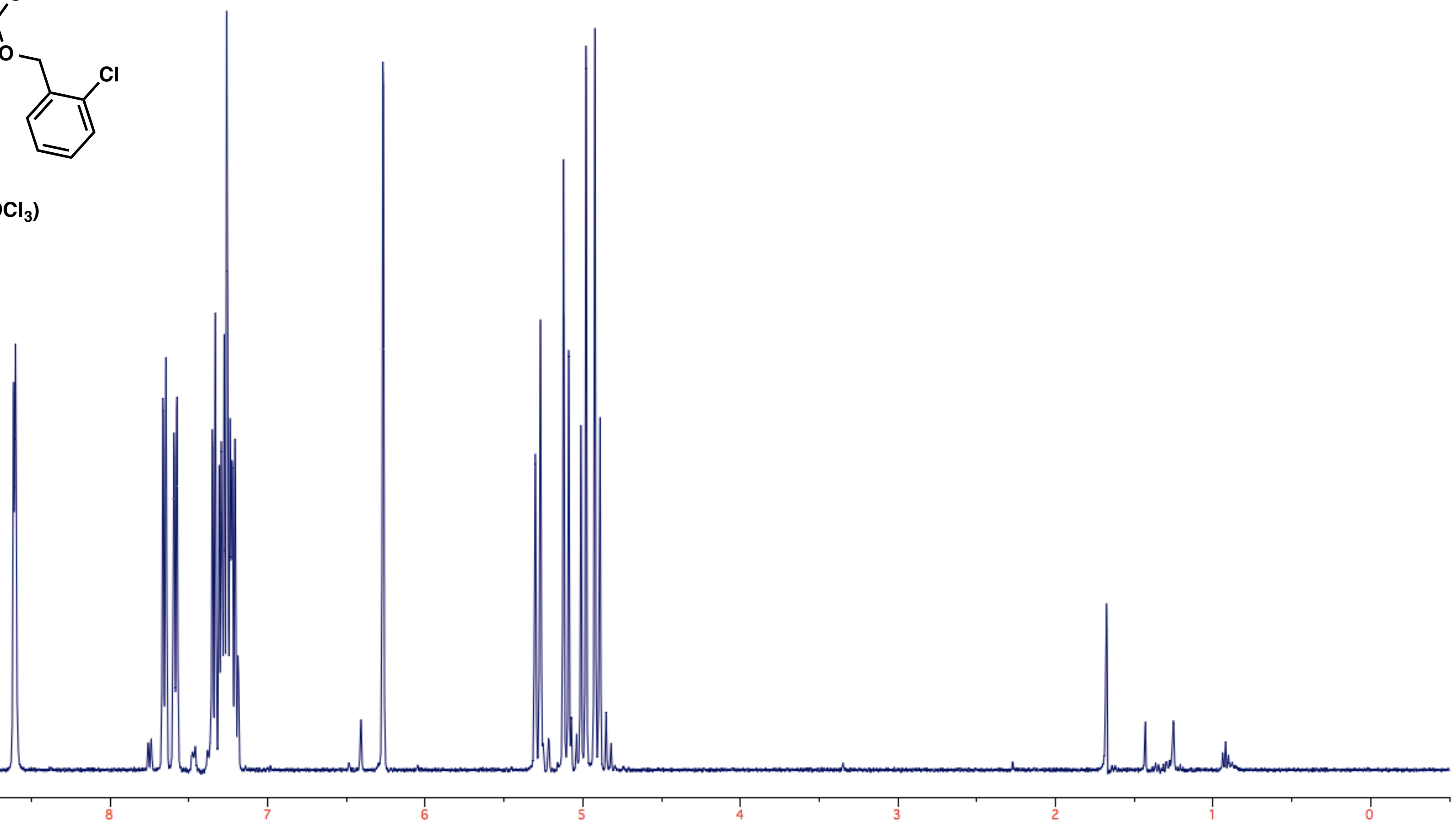

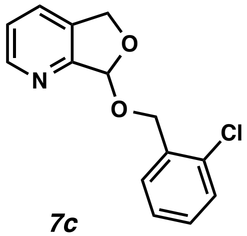

7c
^{13}C NMR
(100 MHz, $CDCl_3$)

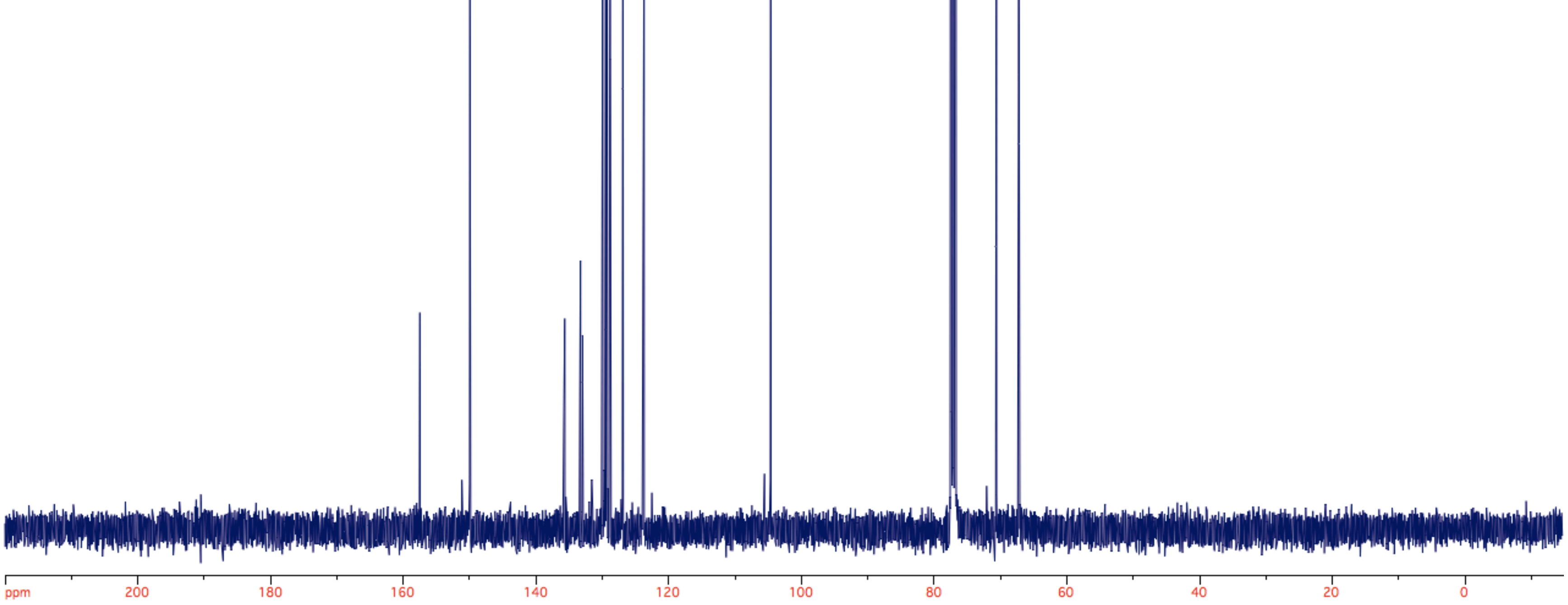

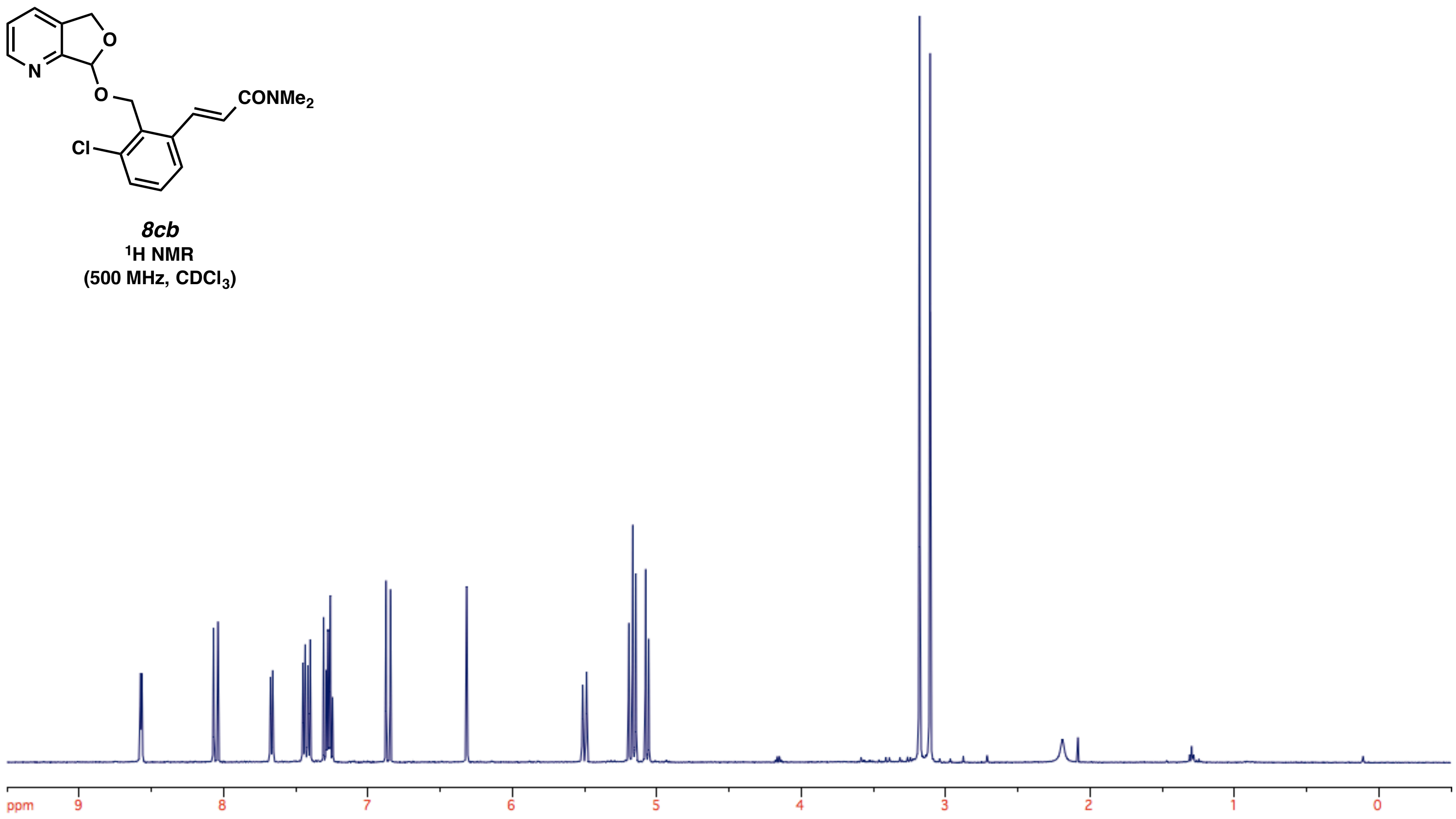

8cb

^{1}H NMR

(500 MHz, $CDCl_3$)

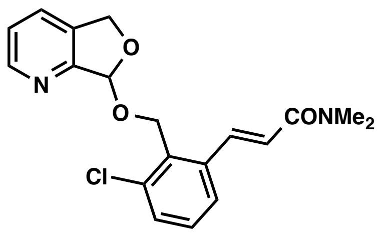

8cb
^{13}C NMR
(125 MHz, $CDCl_3$)

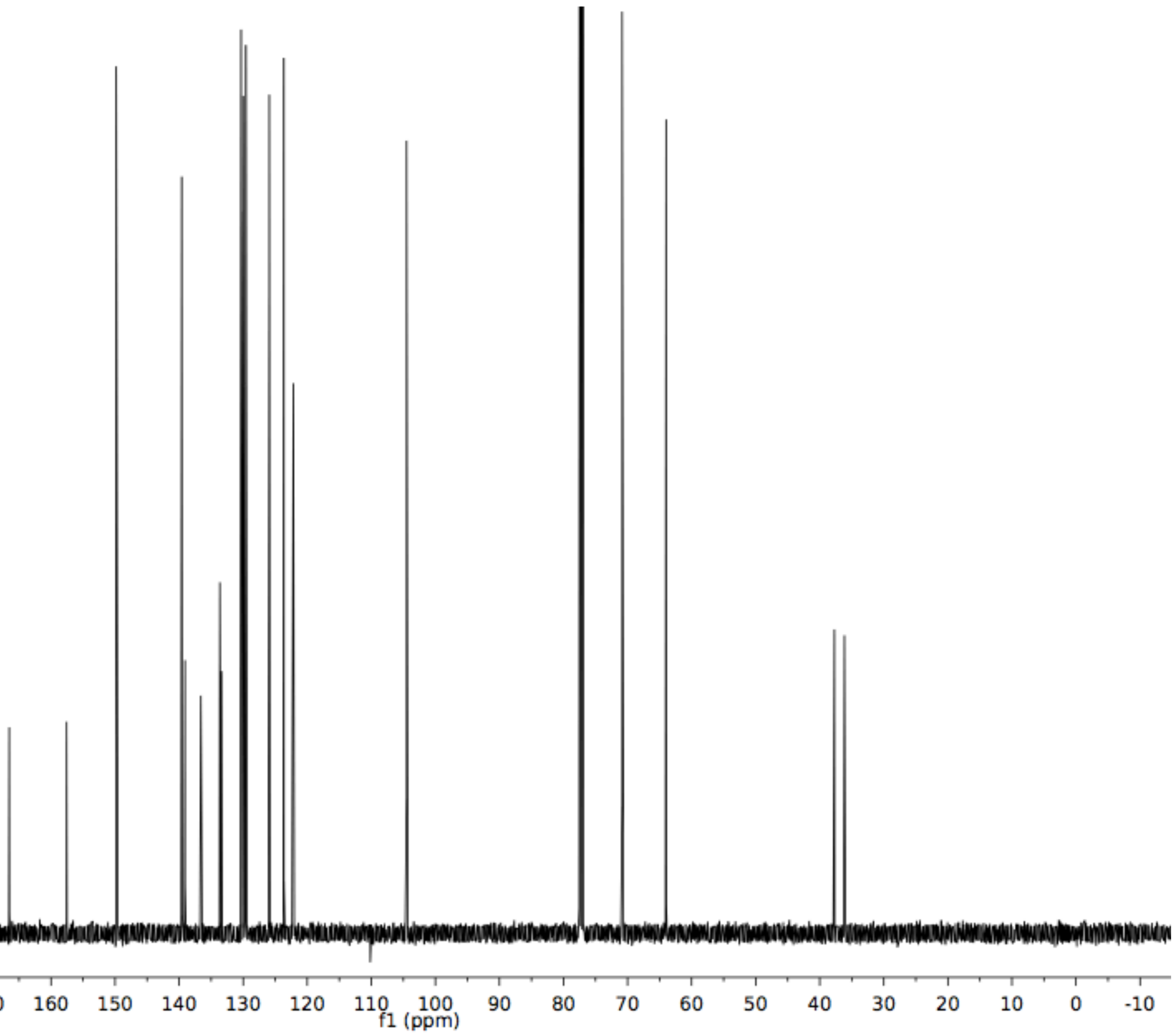

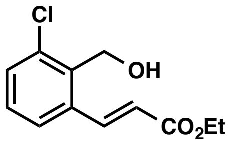

9ca
^{1}H NMR
(500 MHz, $CDCl_3$)

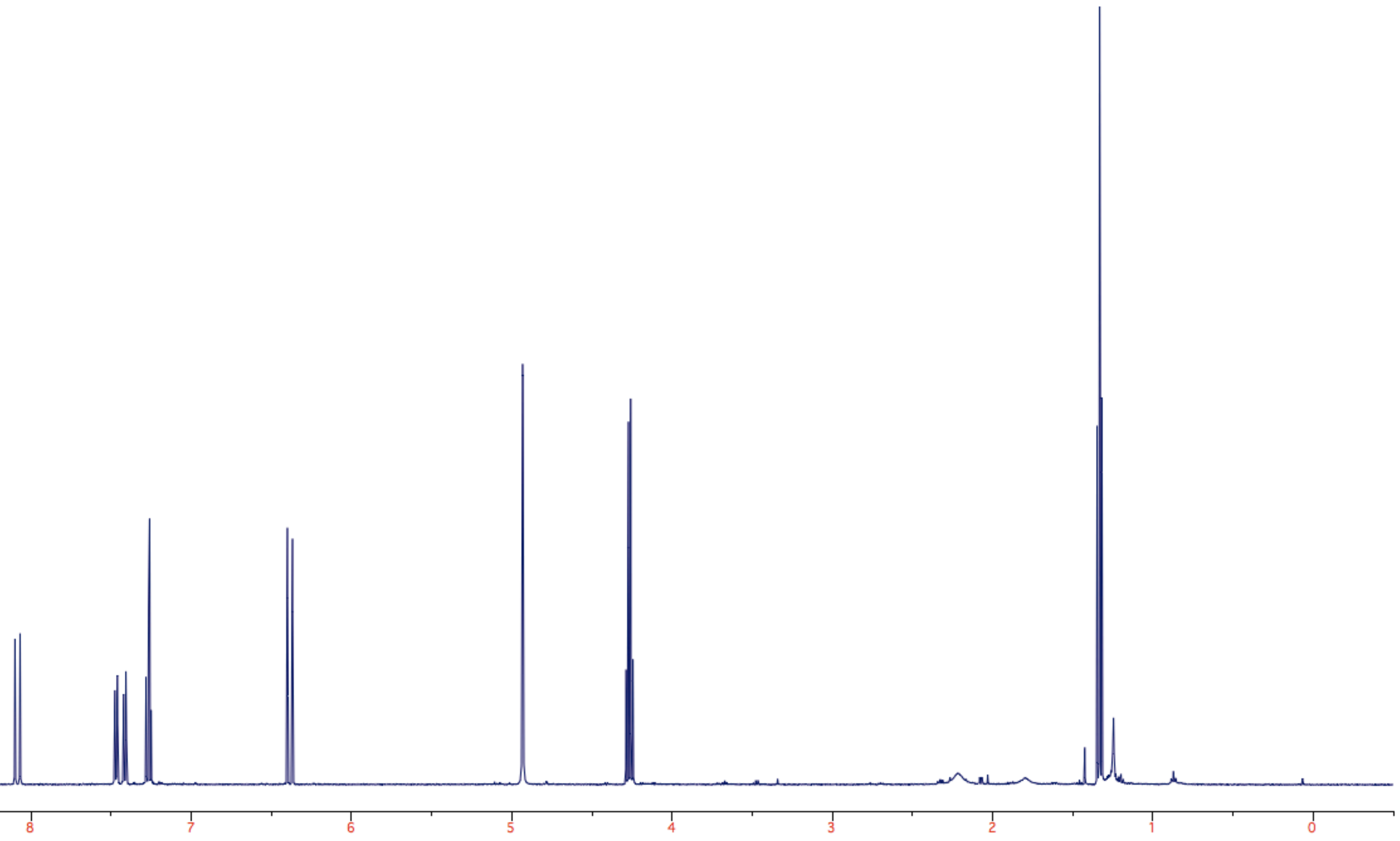

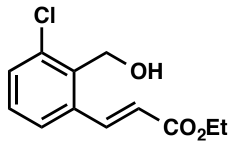

9ca

^{13}C NMR

(125 MHz, $CDCl_3$)

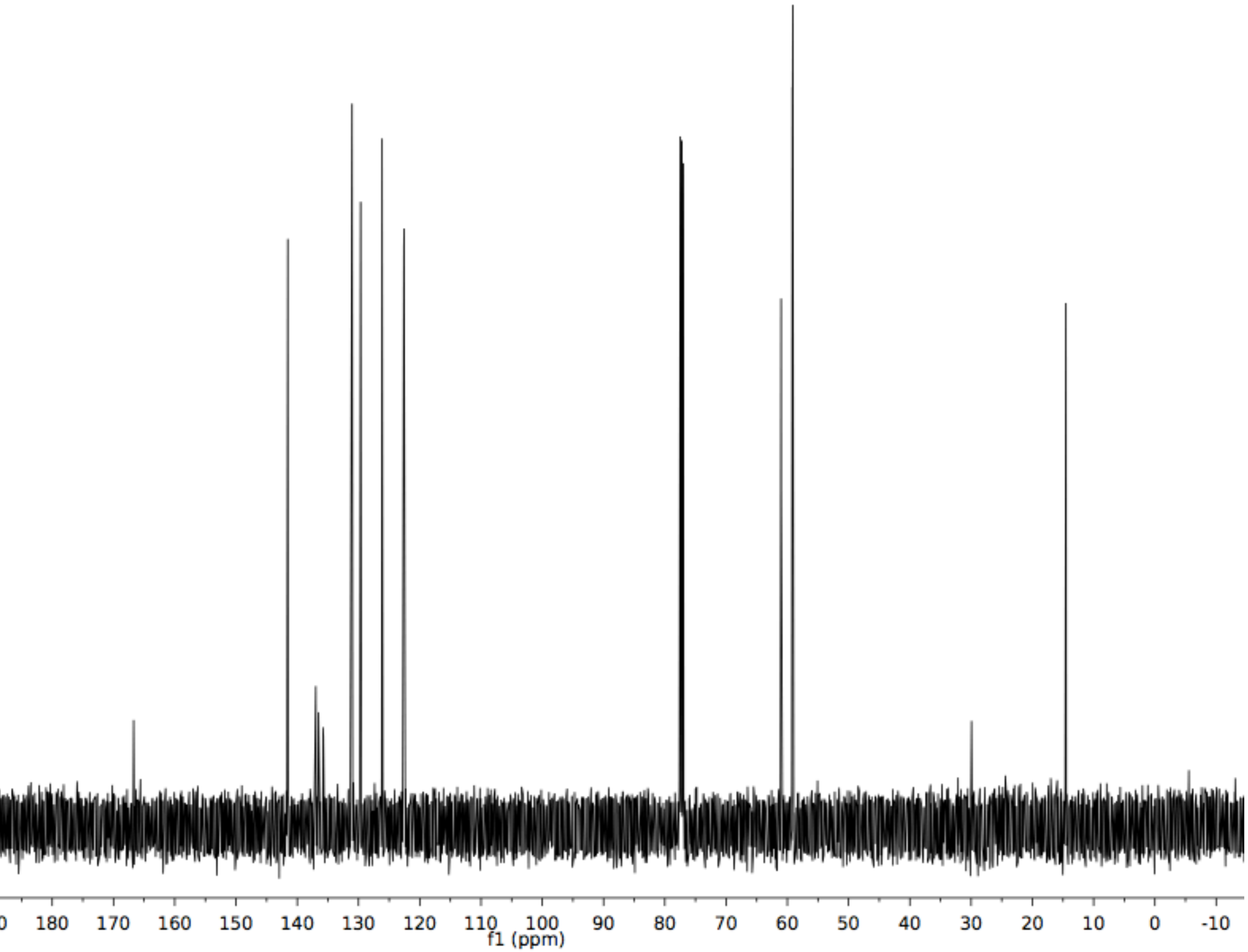

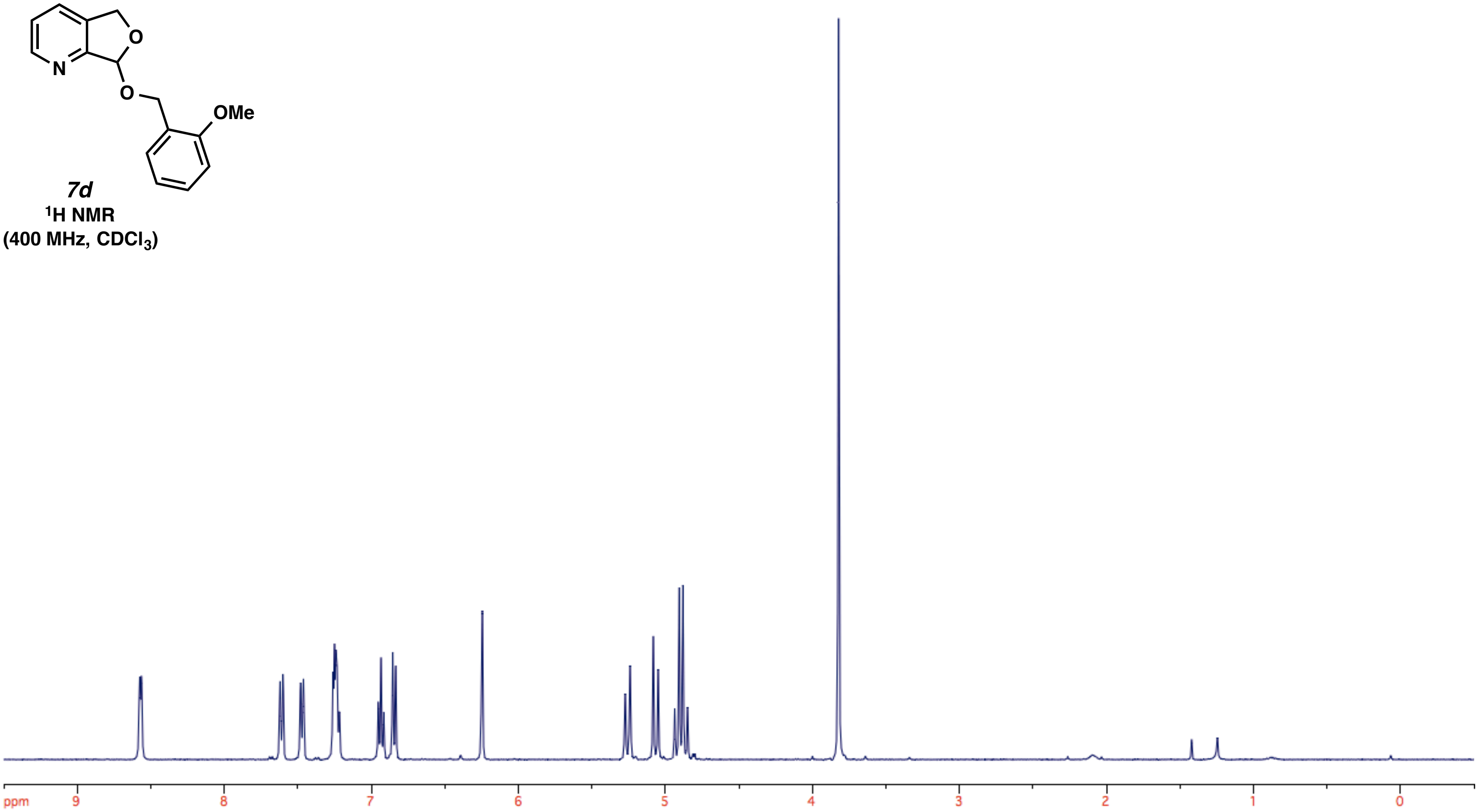

7d
^{1}H NMR
(400 MHz, $CDCl_3$)

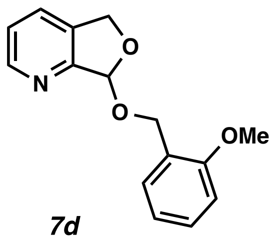

7d
^{13}C NMR
(100 MHz, $CDCl_3$)

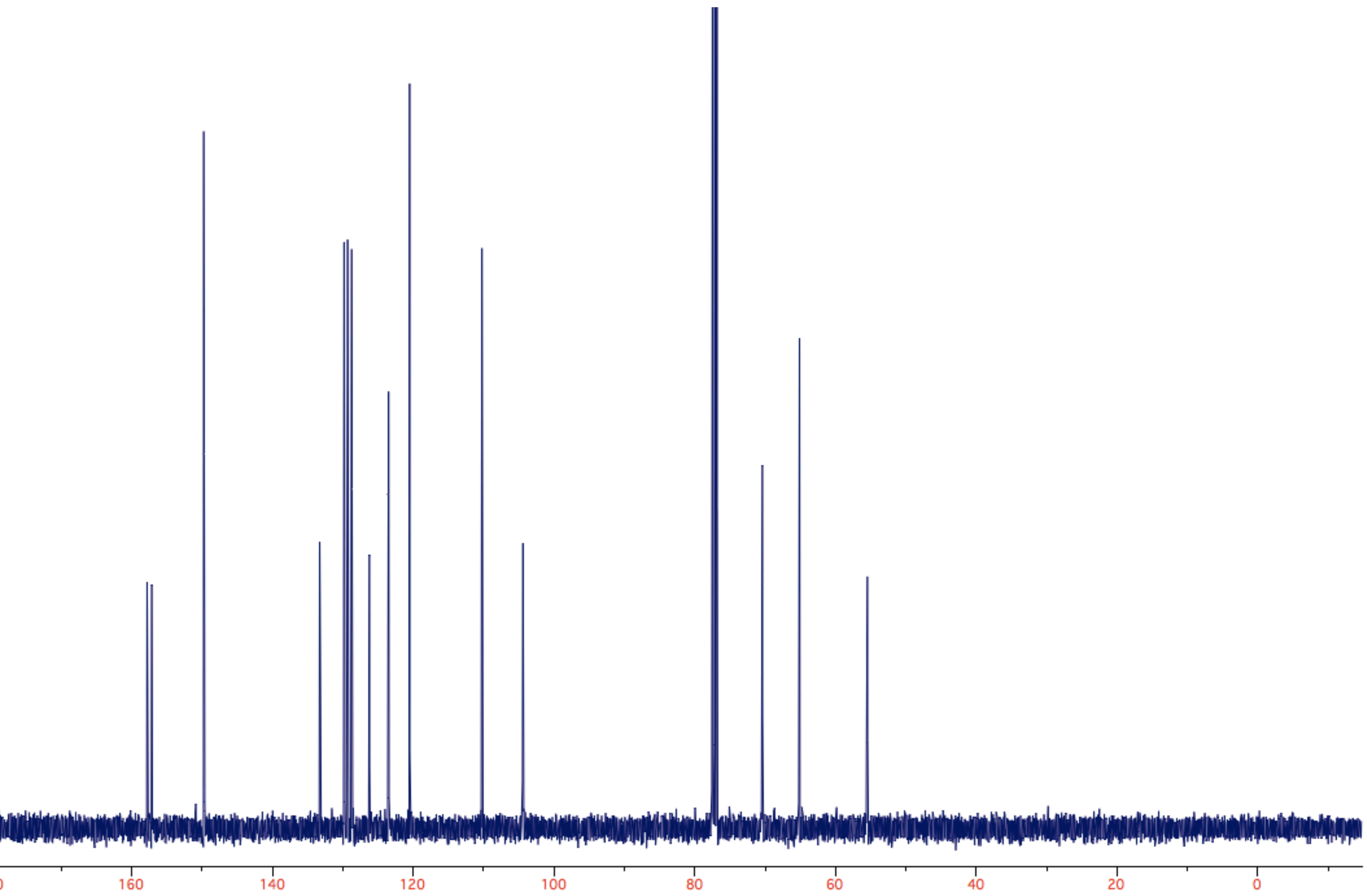

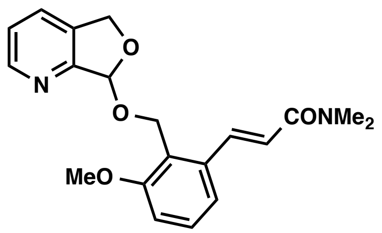

8db
^{1}H NMR
(400 MHz, $CDCl_3$)

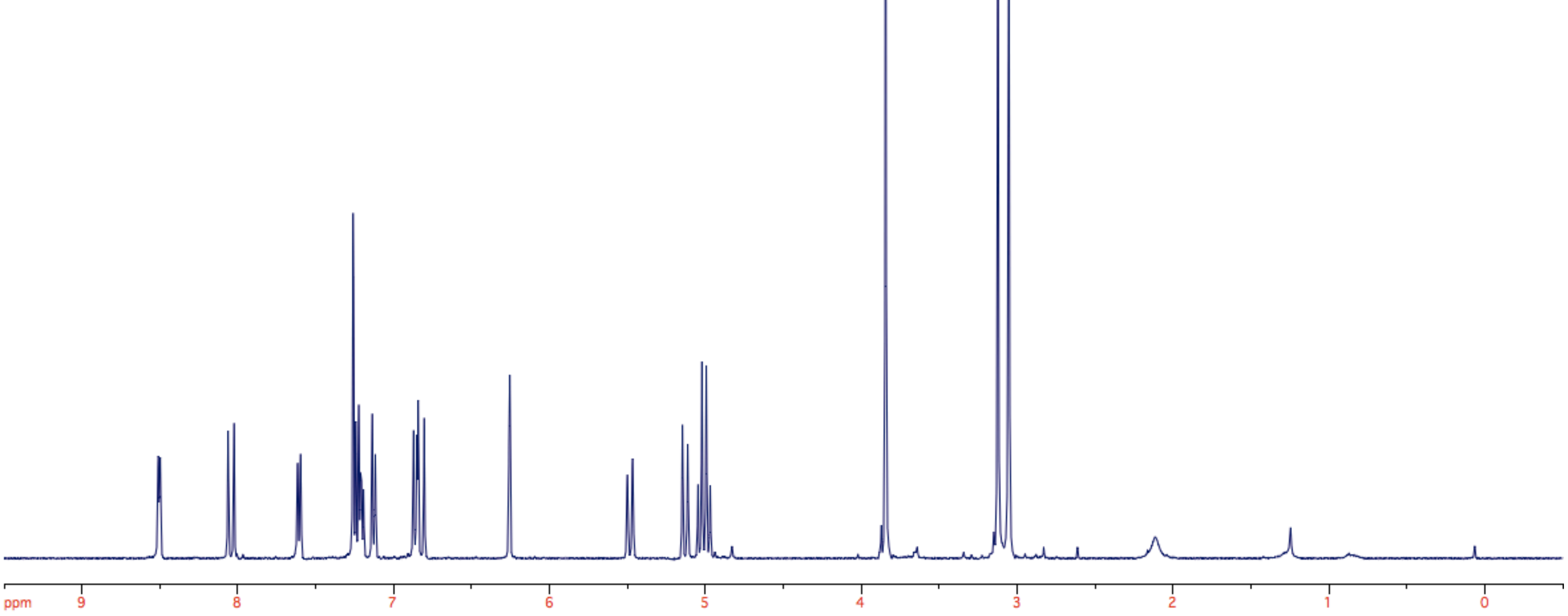

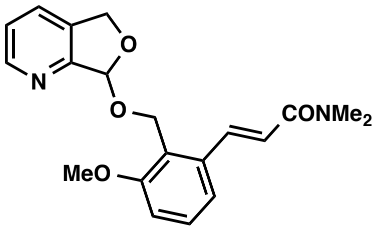

8db
^{13}C NMR
(100 MHz, $CDCl_3$)

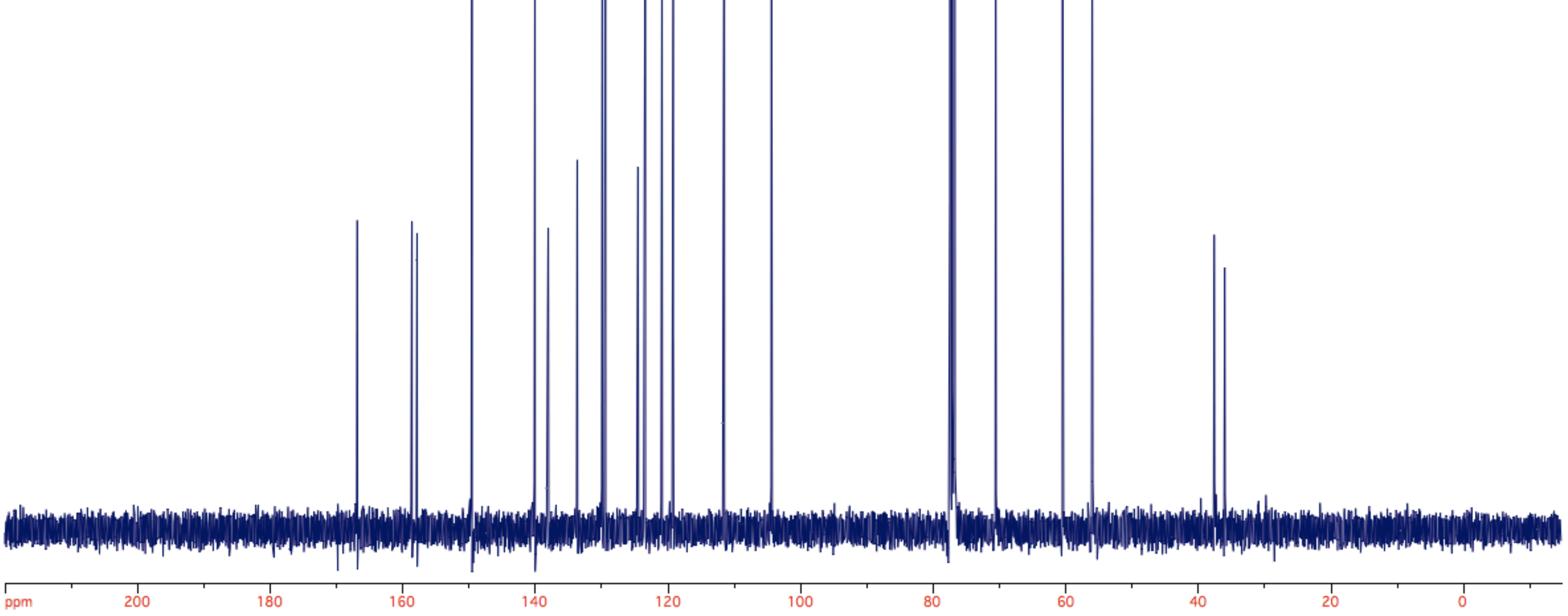

7e
^{1}H NMR
(400 MHz, $CDCl_3$)

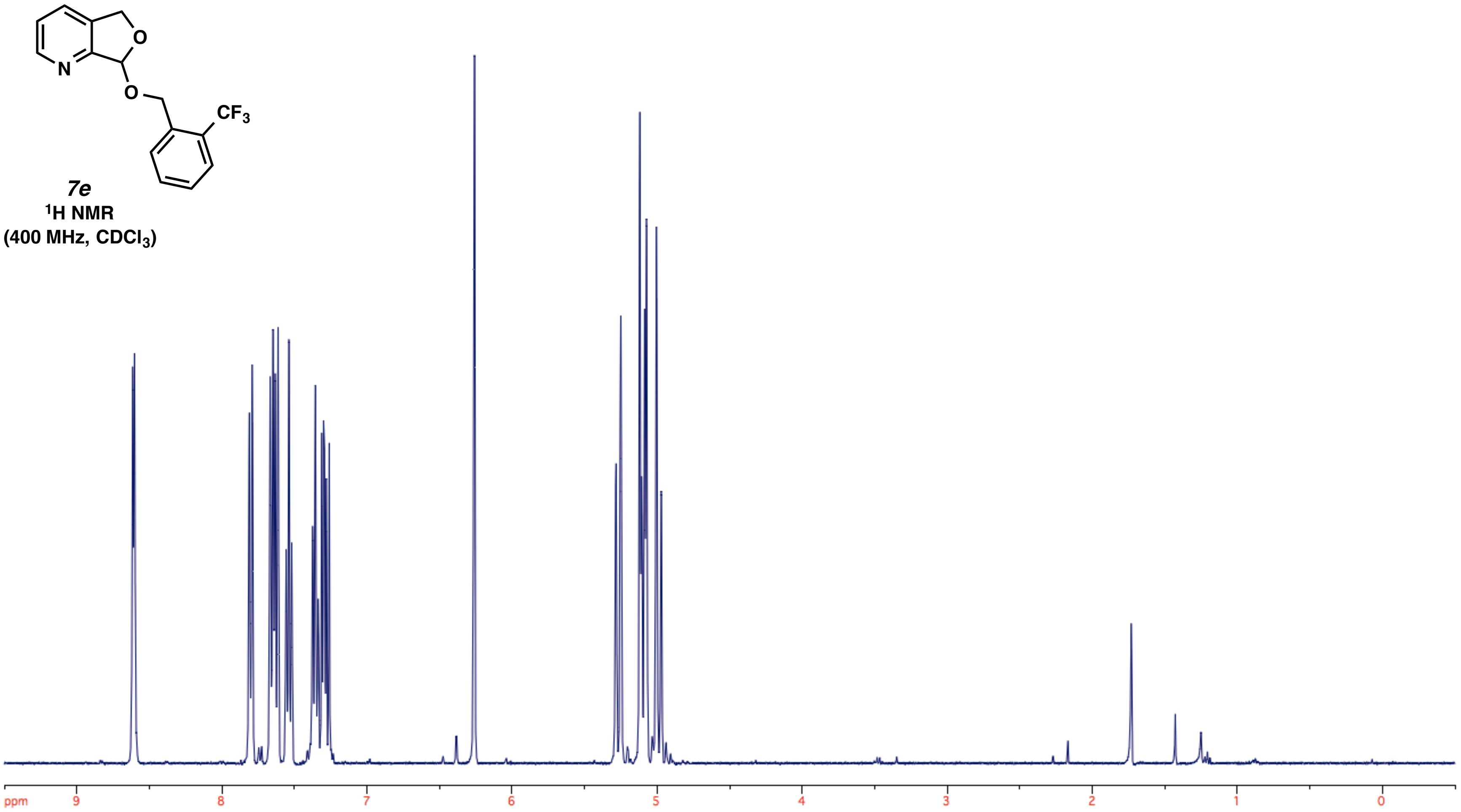

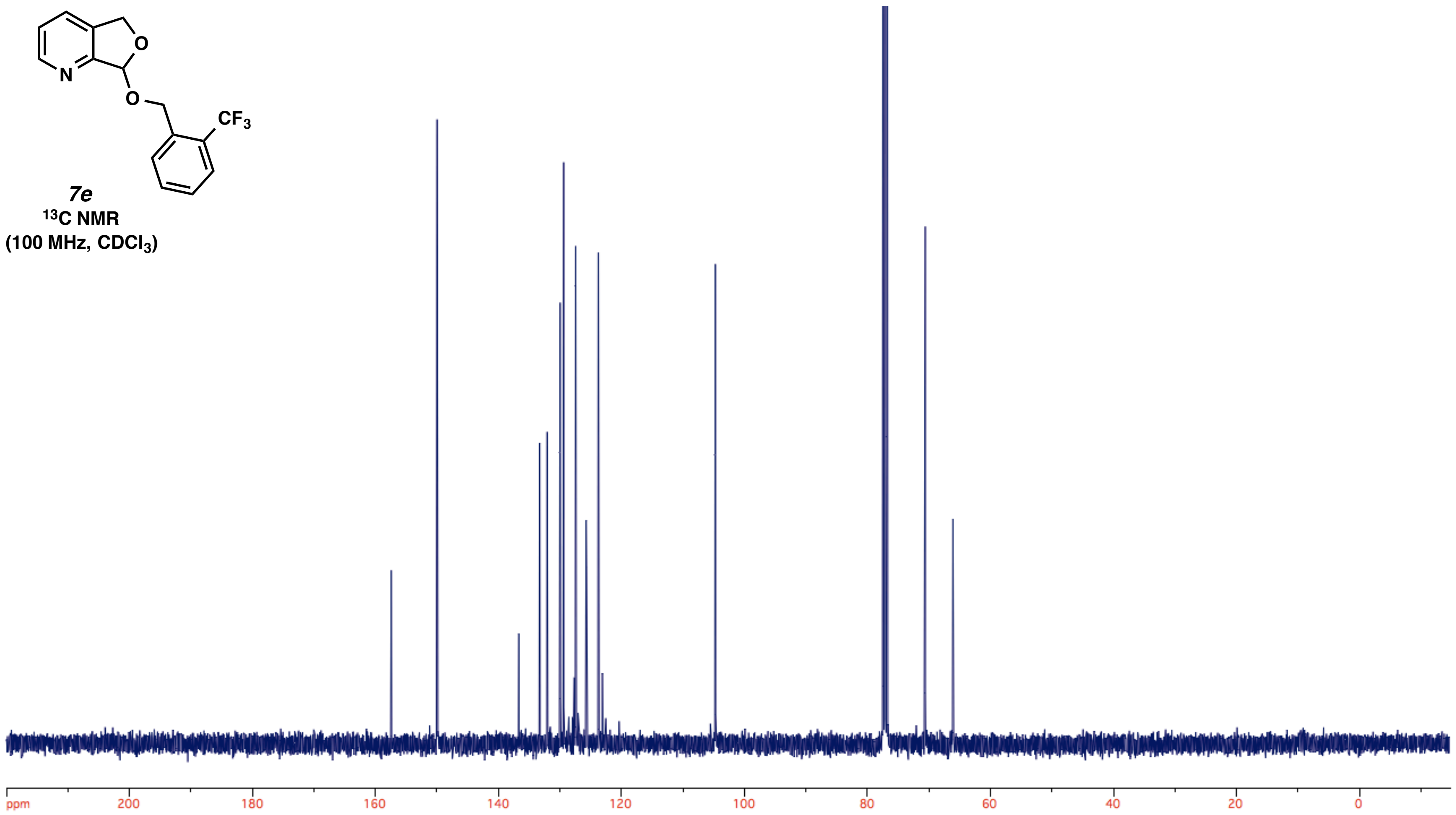
CF3
N
O
7e
13C NMR
(100 MHz, CDCl3)
ppm
200
180
160
140
120
100
80
60
40
20
0

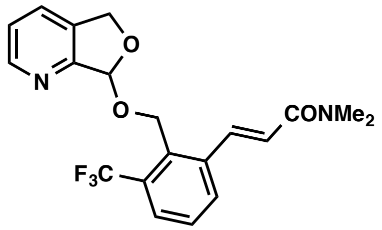

8eb
^{1}H NMR
(400 MHz, $CDCl_3$)

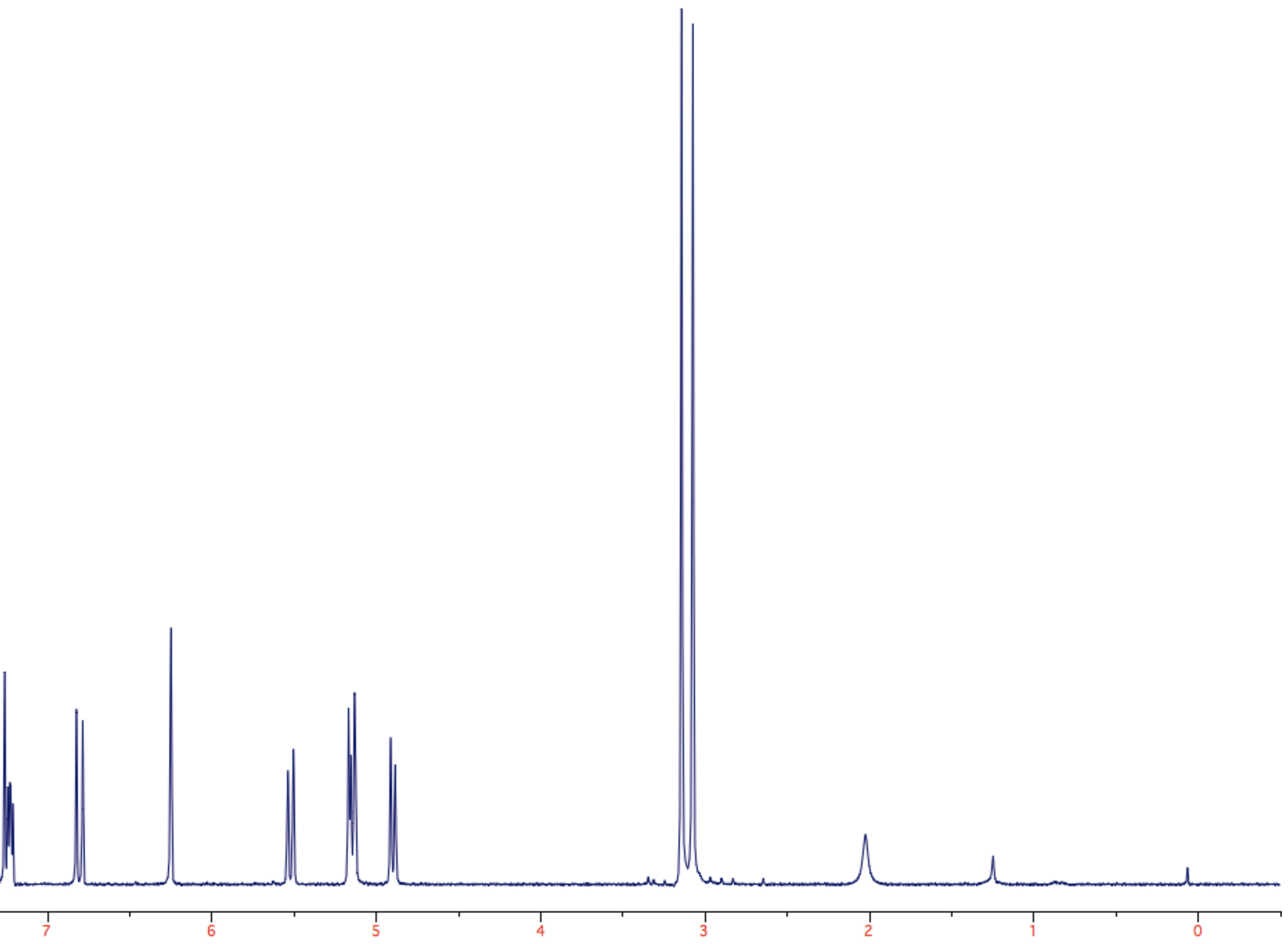

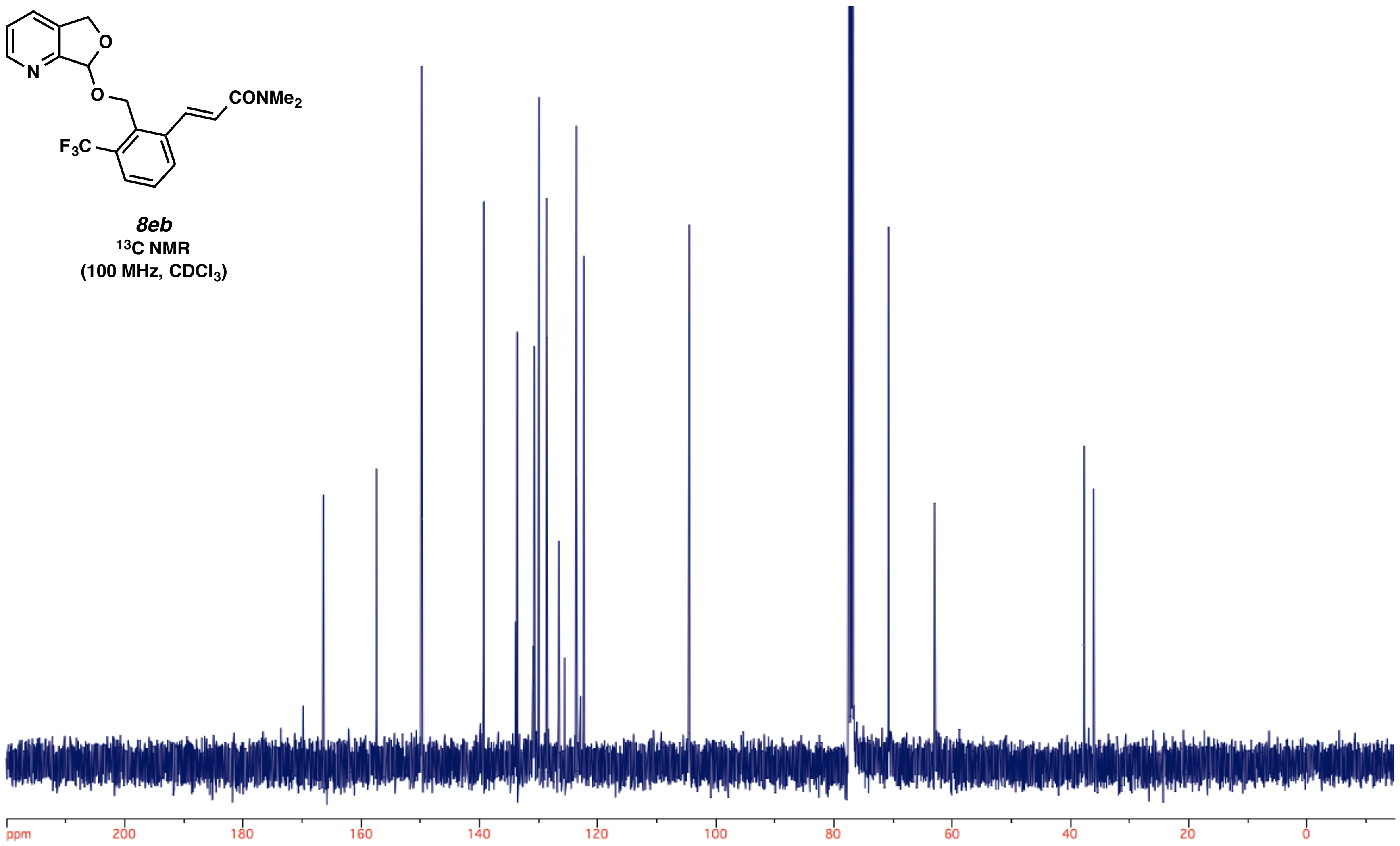
O
N
O
CONMe2
F3C
8eb
13C NMR
(100 MHz, CDCl3)
ppm
200
180
160
140
120
100
80
60
40
20
0

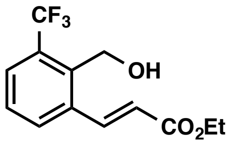

9ea
^{1}H NMR
(400 MHz, $CDCl_3$)

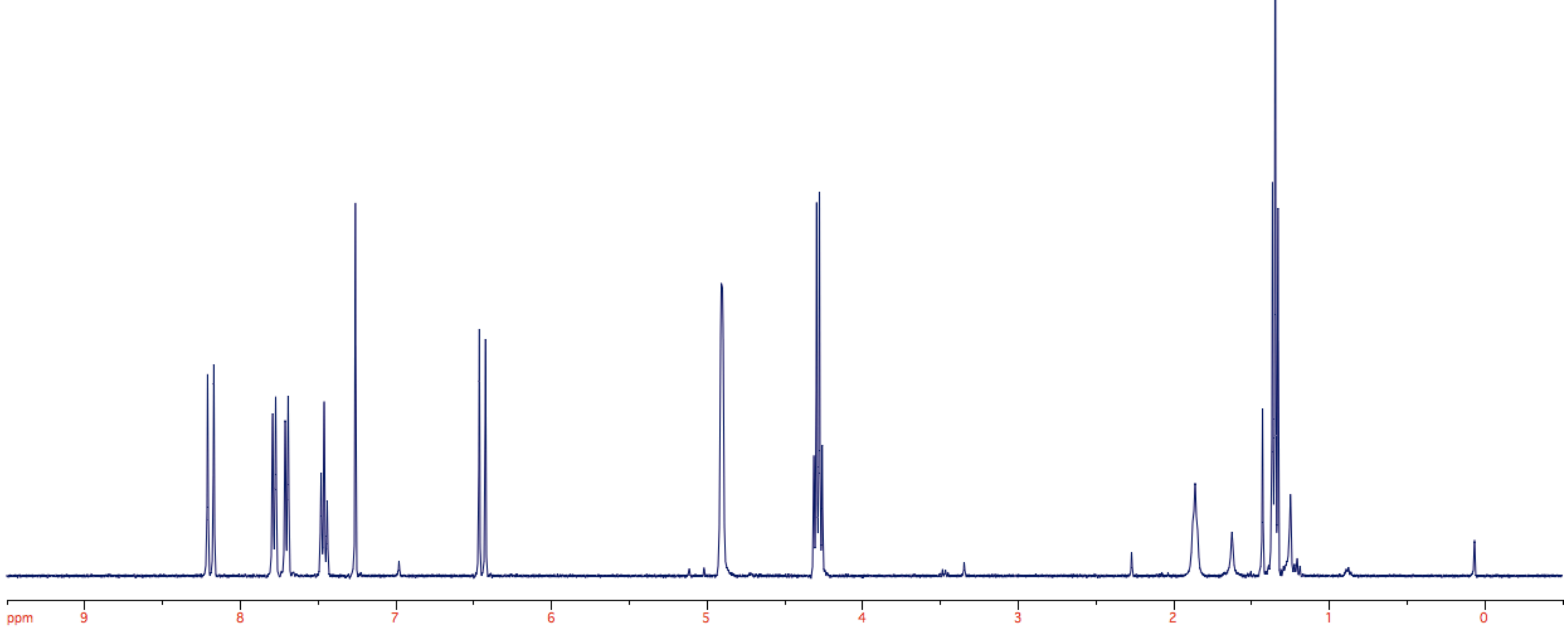

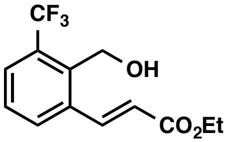

9ea
^{13}C NMR
(125 MHz, $CDCl_3$)

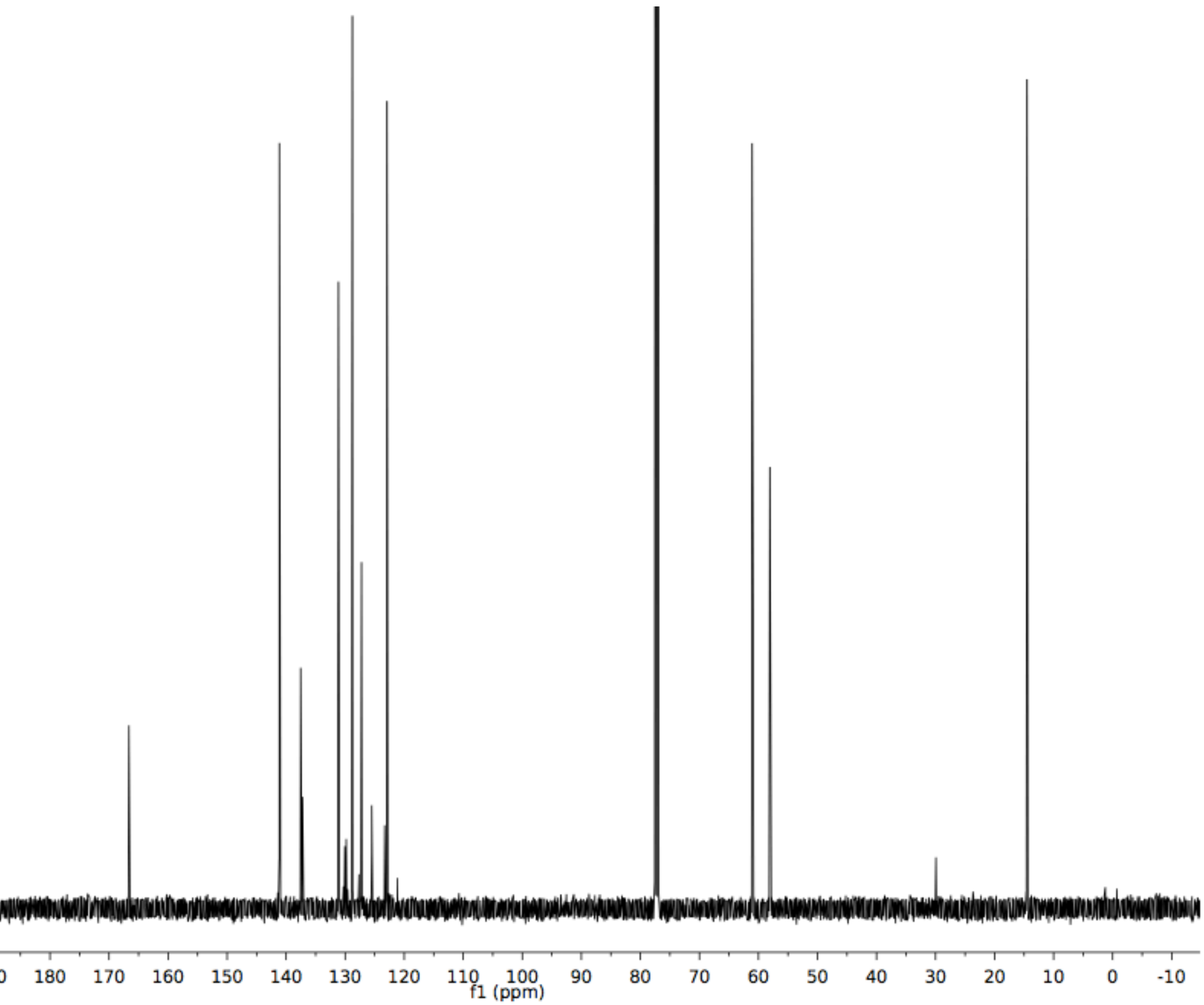

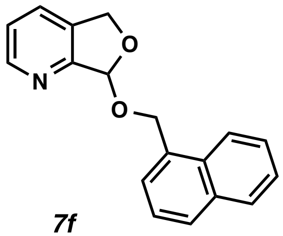

7f
¹H NMR
(400 MHz, $CDCl_3$)

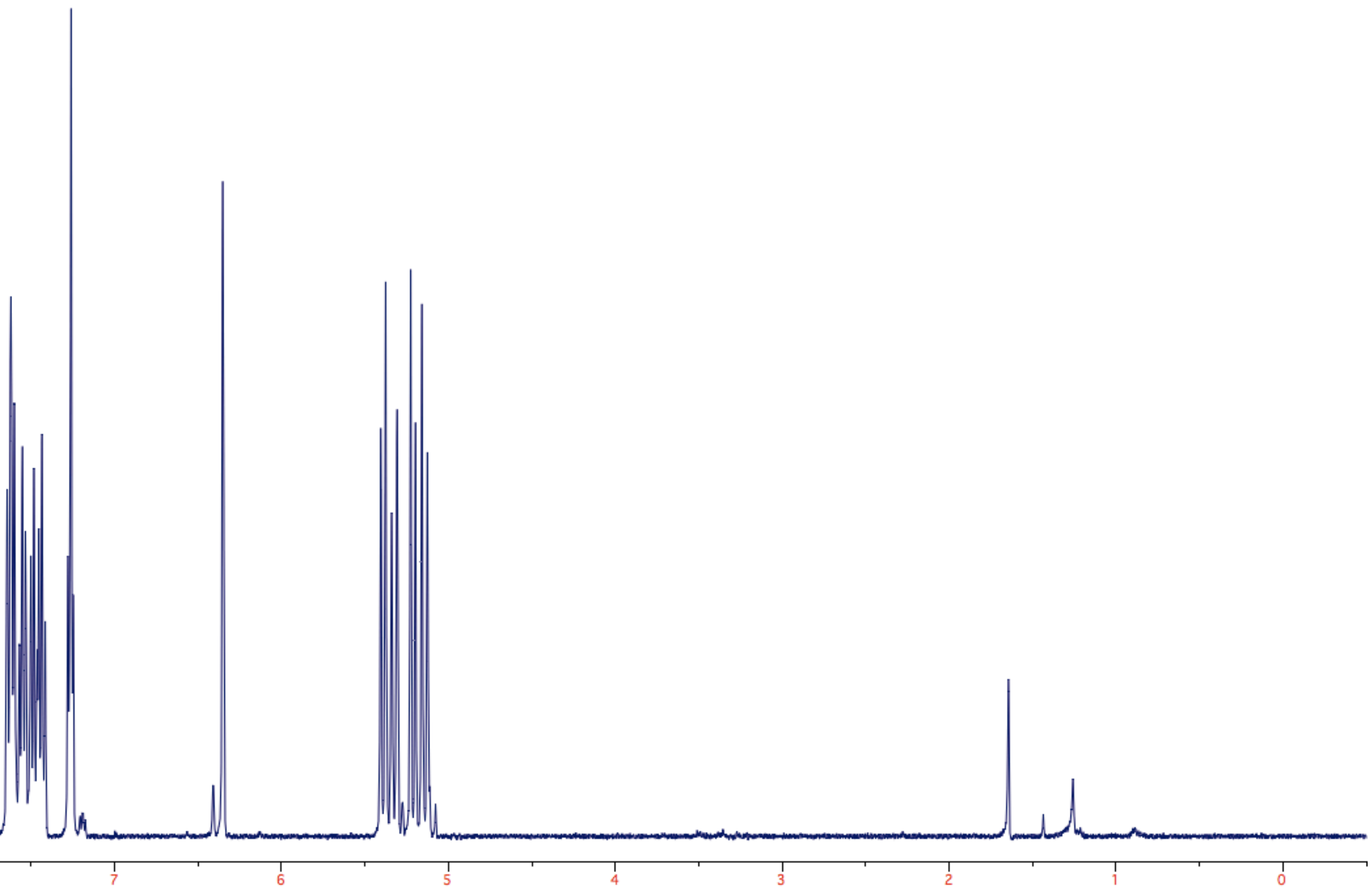

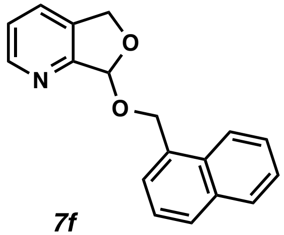

7f
^{13}C NMR
(100 MHz, $CDCl_3$)

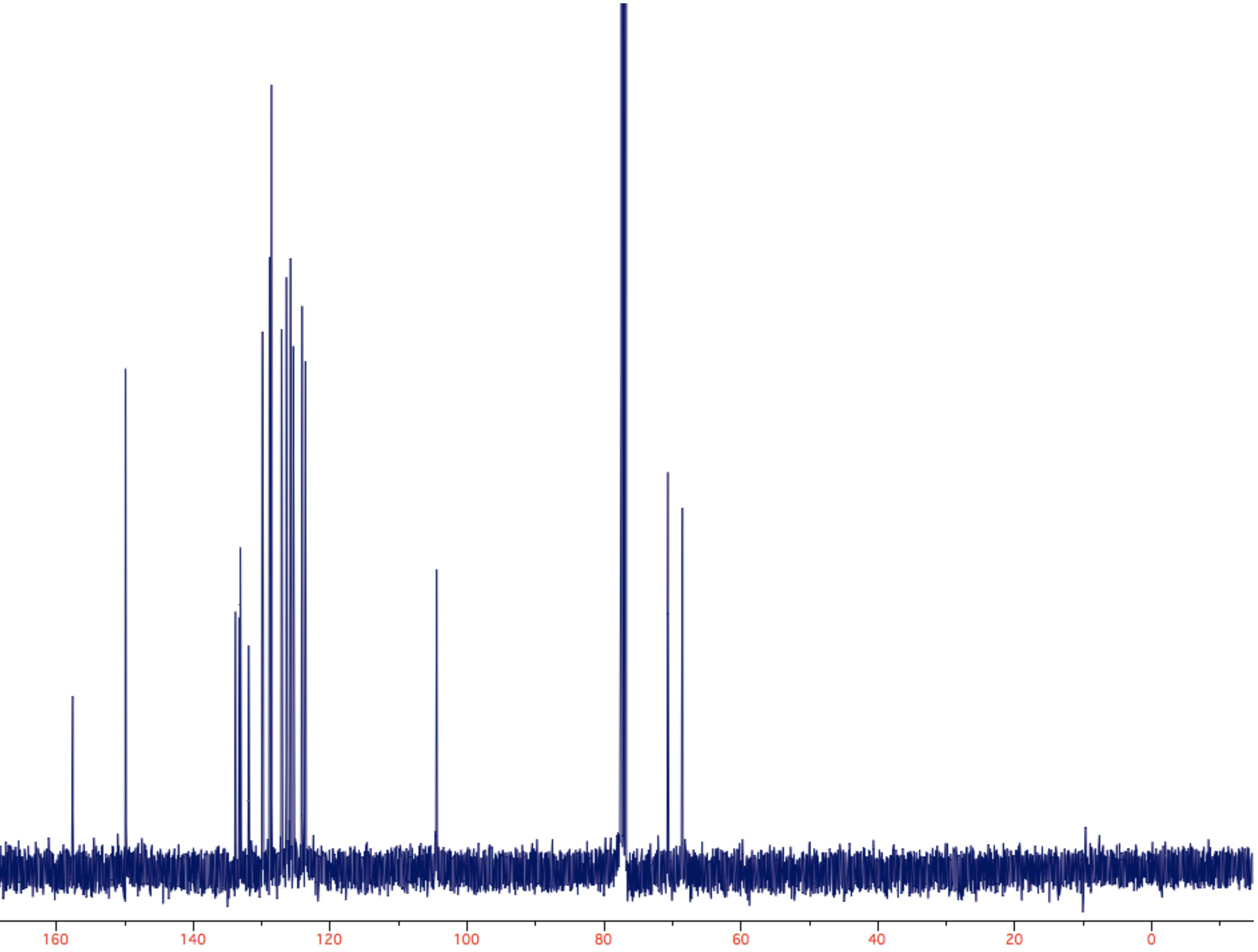

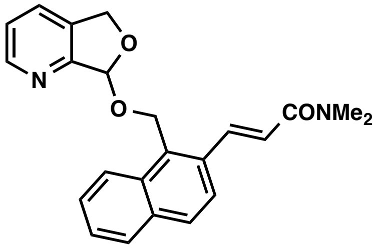

8fb
^{1}H NMR
(400 MHz, $CDCl_3$)

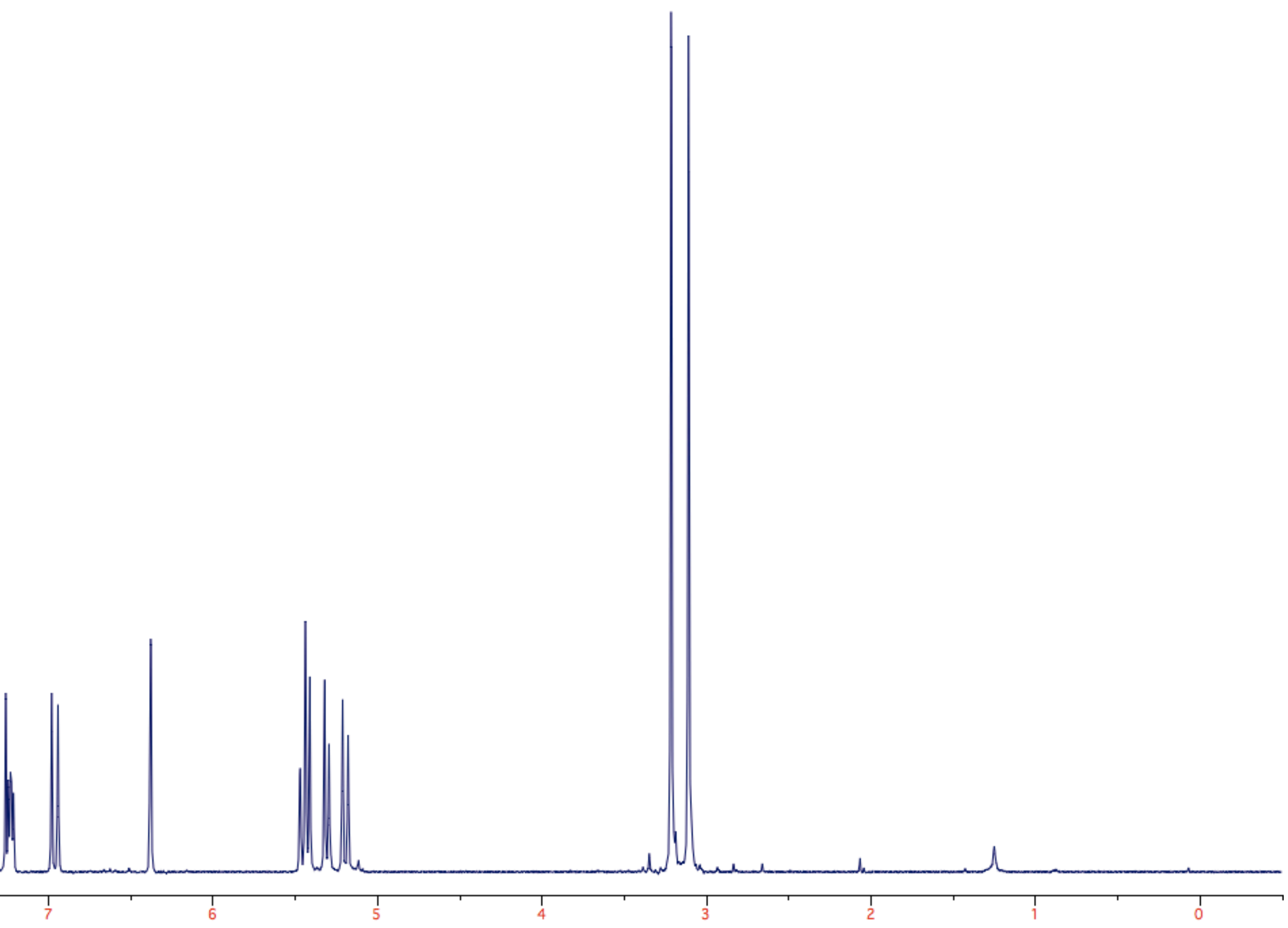

8fb
^{13}C NMR
(100 MHz, $CDCl_3$)

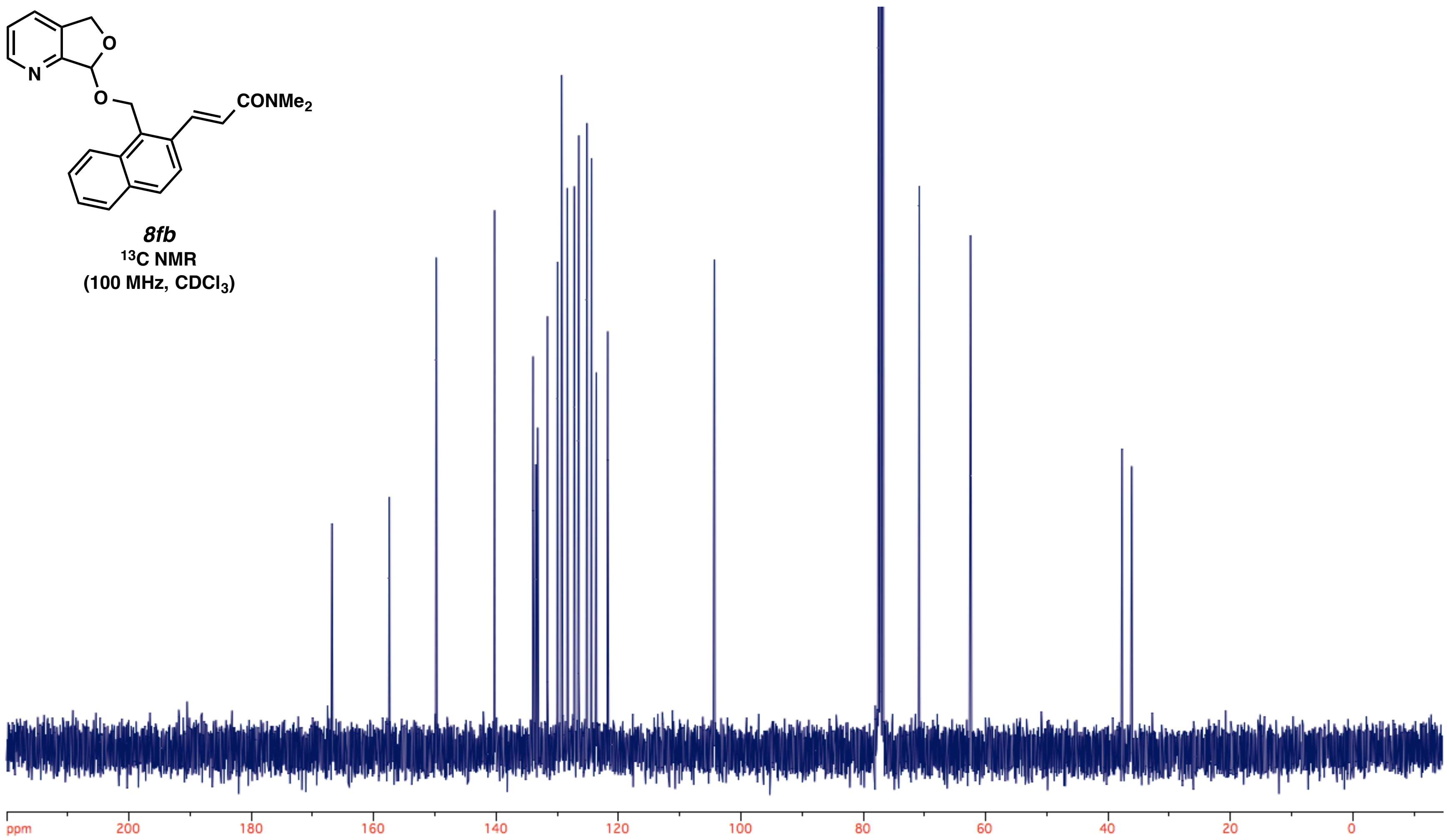

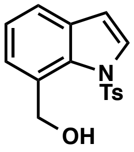

1g
^{1}H NMR
(400 MHz, $CDCl_3$)

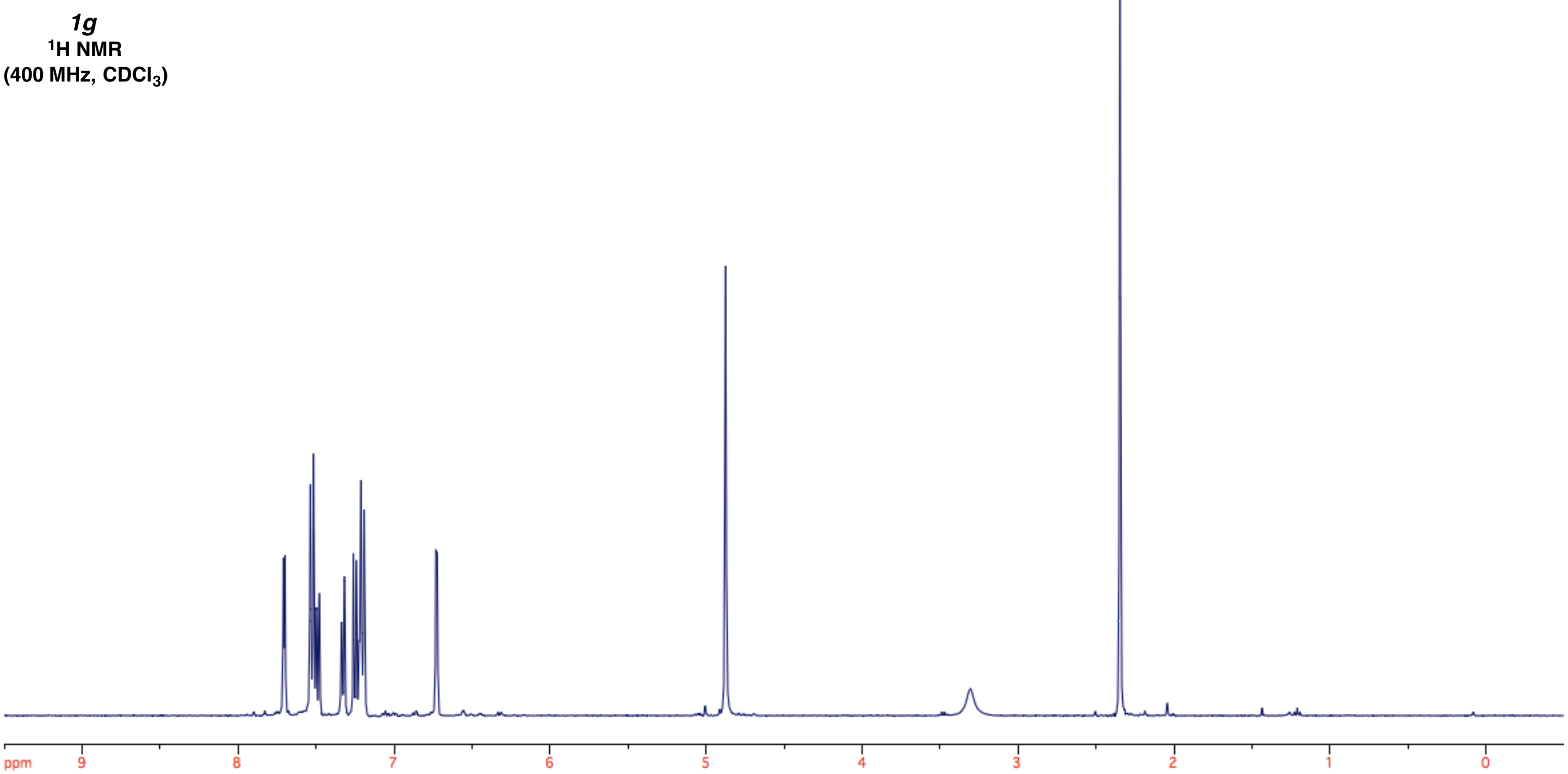

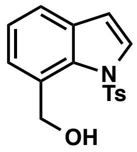

1g
^{13}C NMR
(100 MHz, $CDCl_3$)

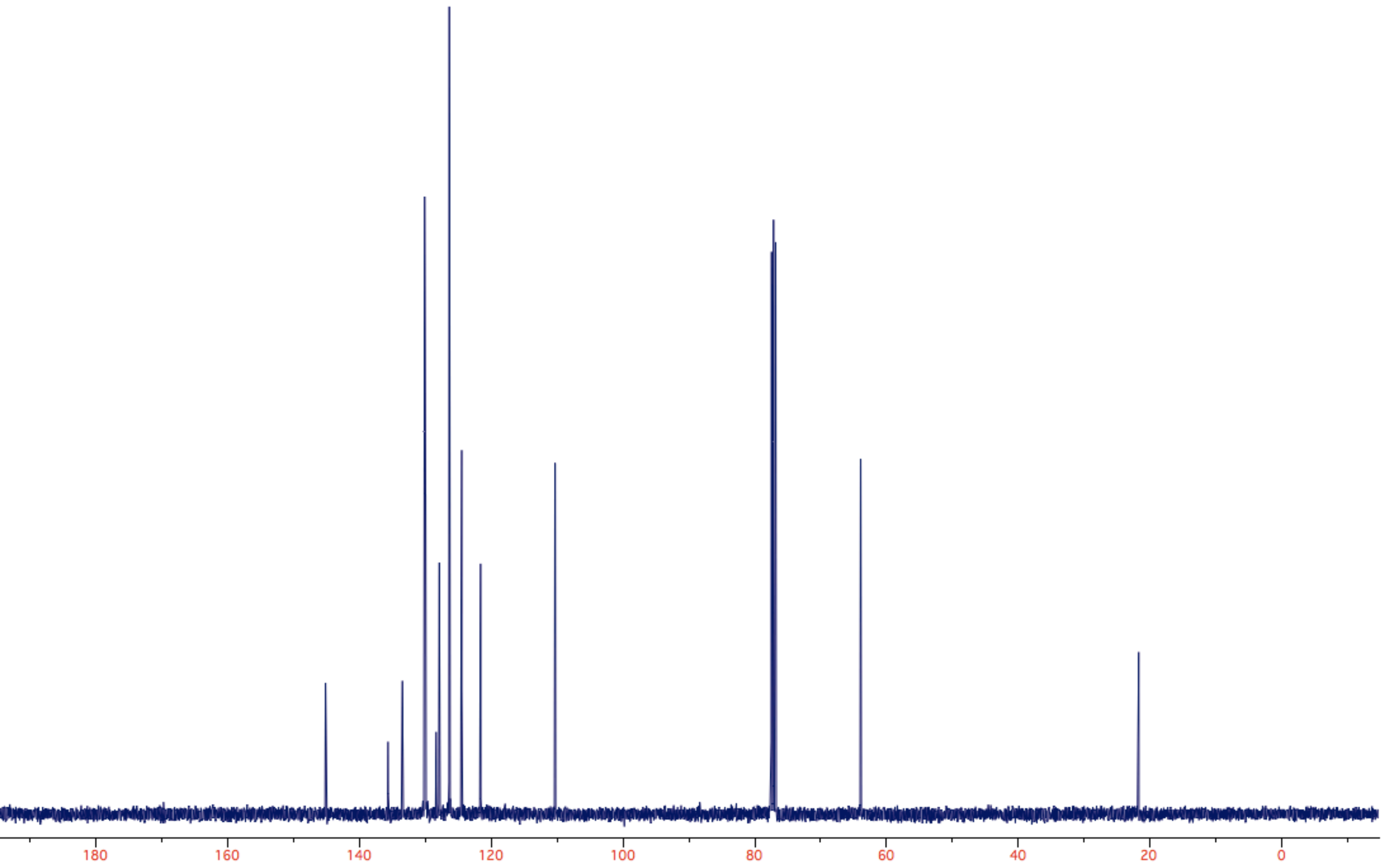

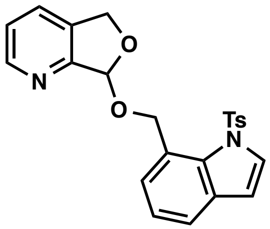

7g
^{1}H NMR
(400 MHz, $CDCl_3$)

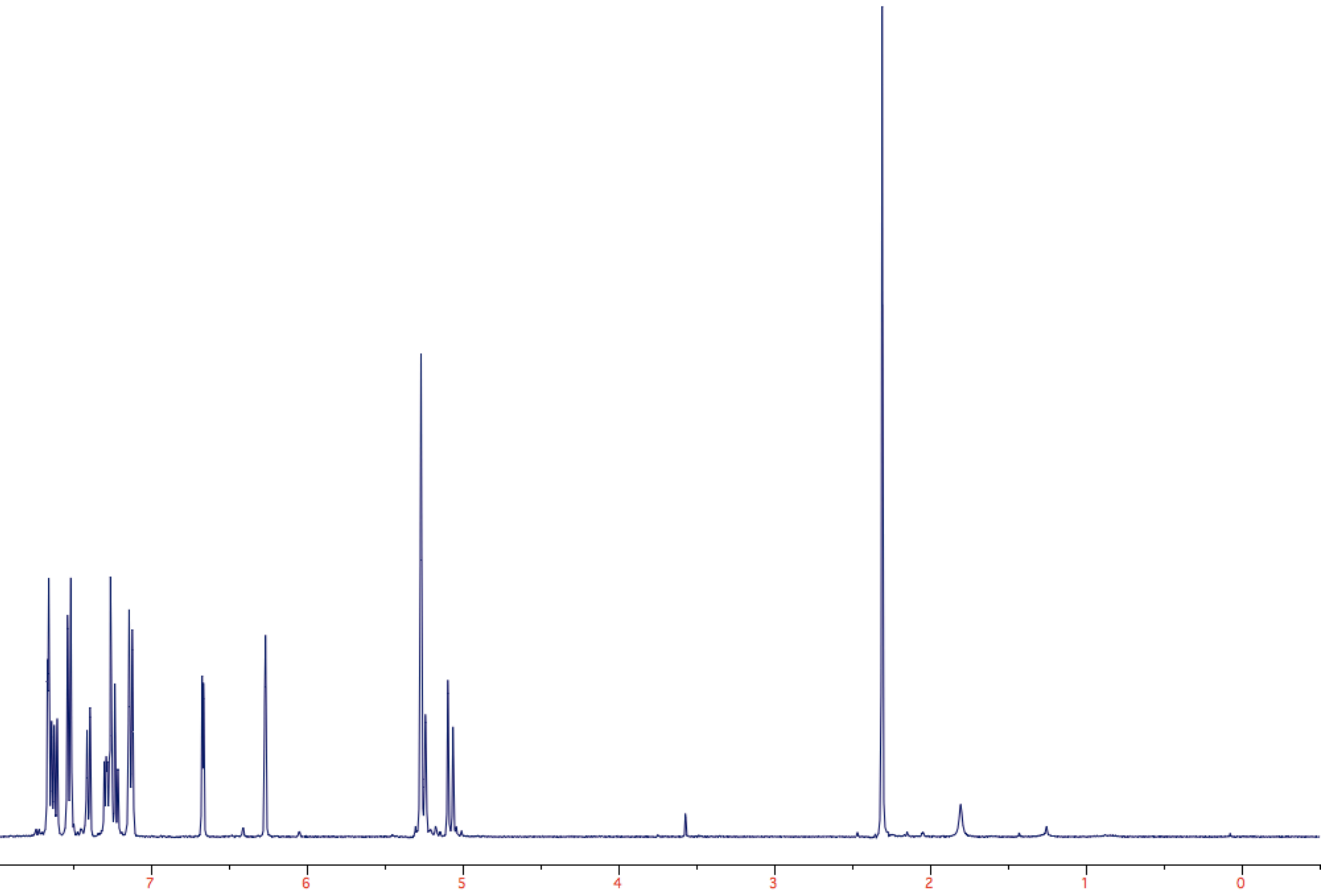

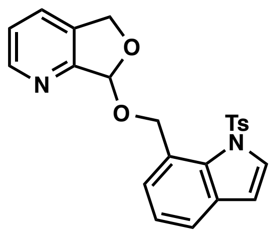

7g
^{13}C NMR
(100 MHz, $CDCl_3$)

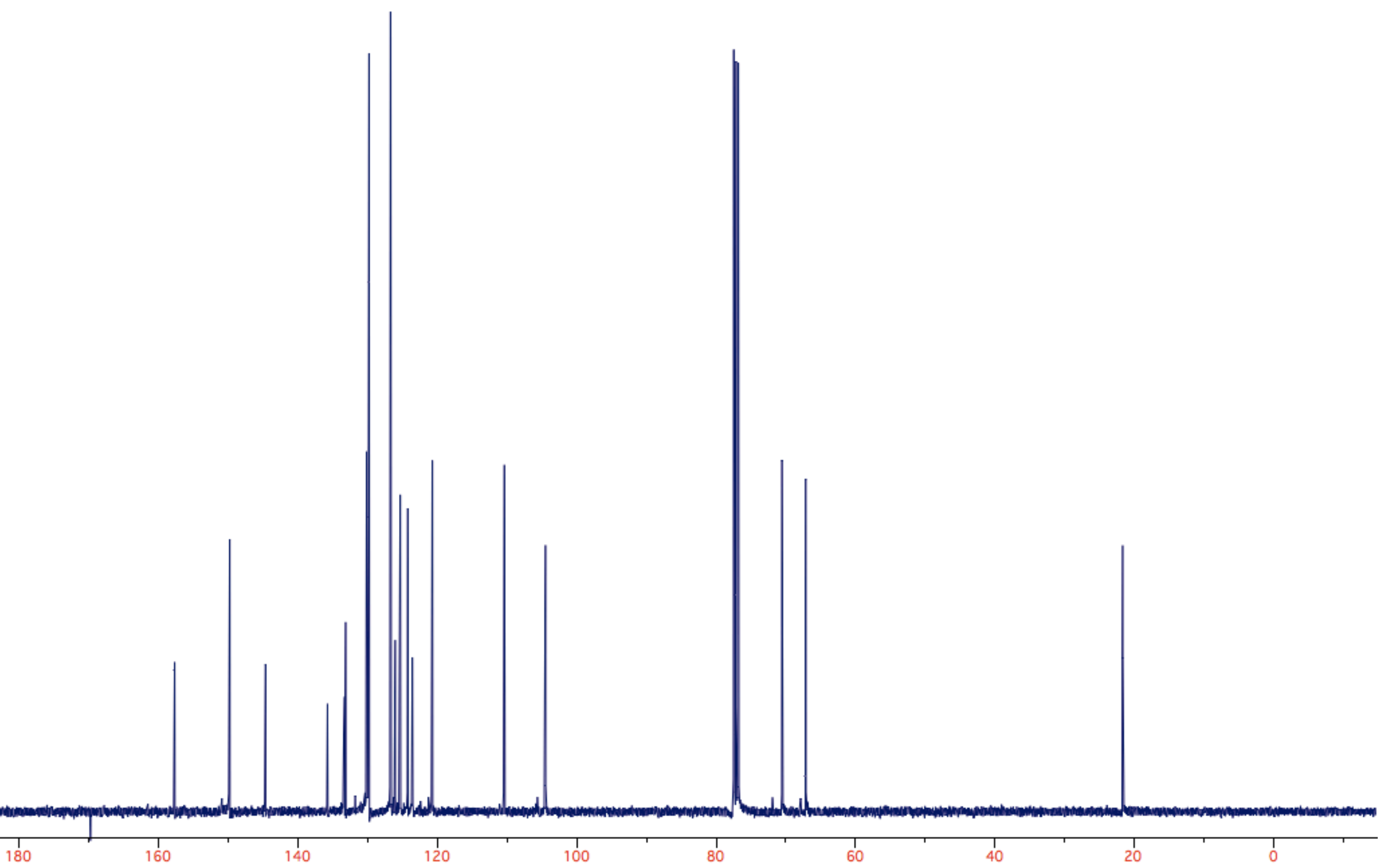

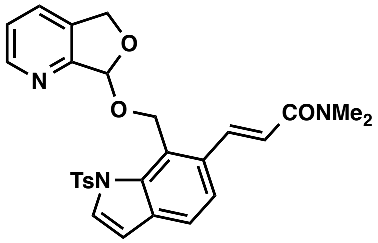

8gb
^{1}H NMR
(400 MHz, $CDCl_3$)

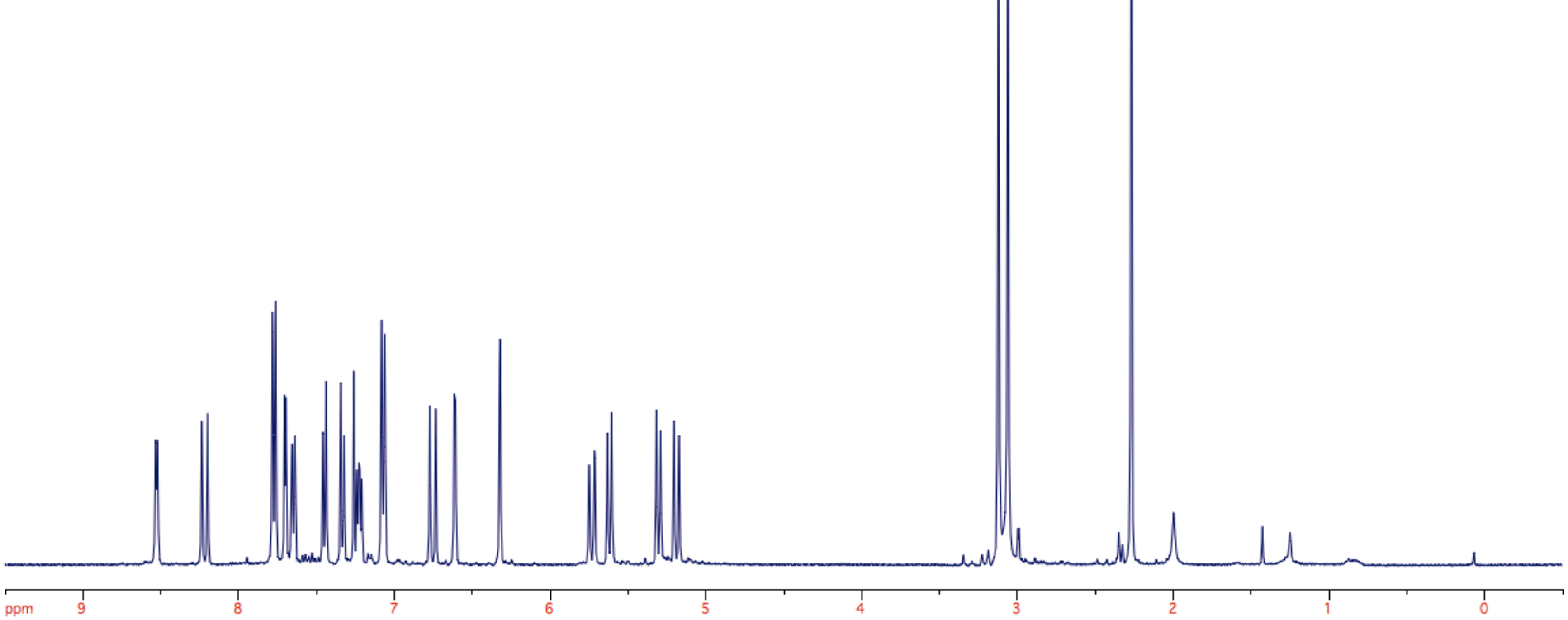

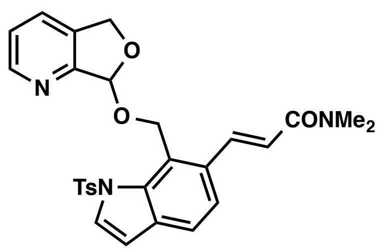

8gb
^{13}C NMR
(100 MHz, $CDCl_3$)

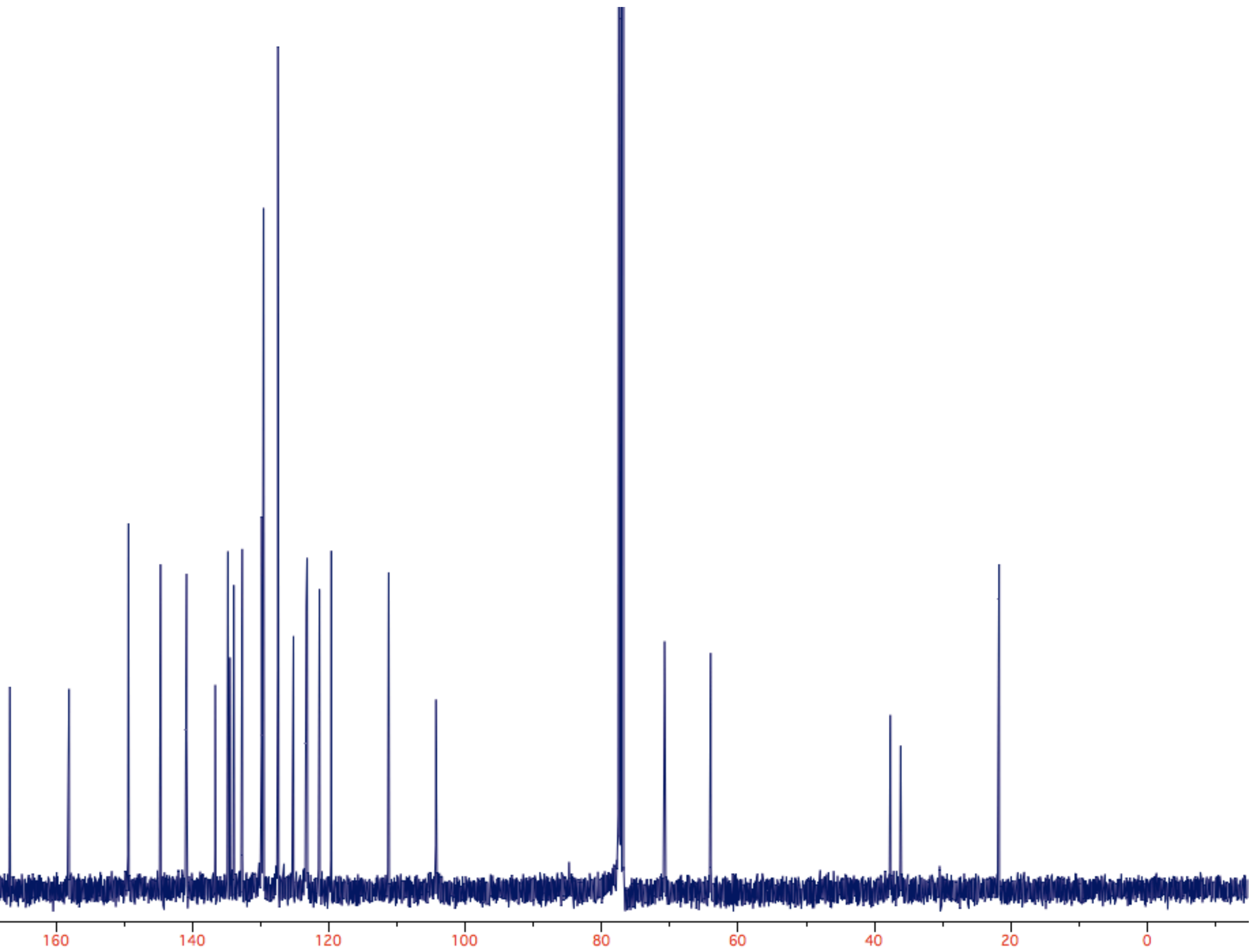

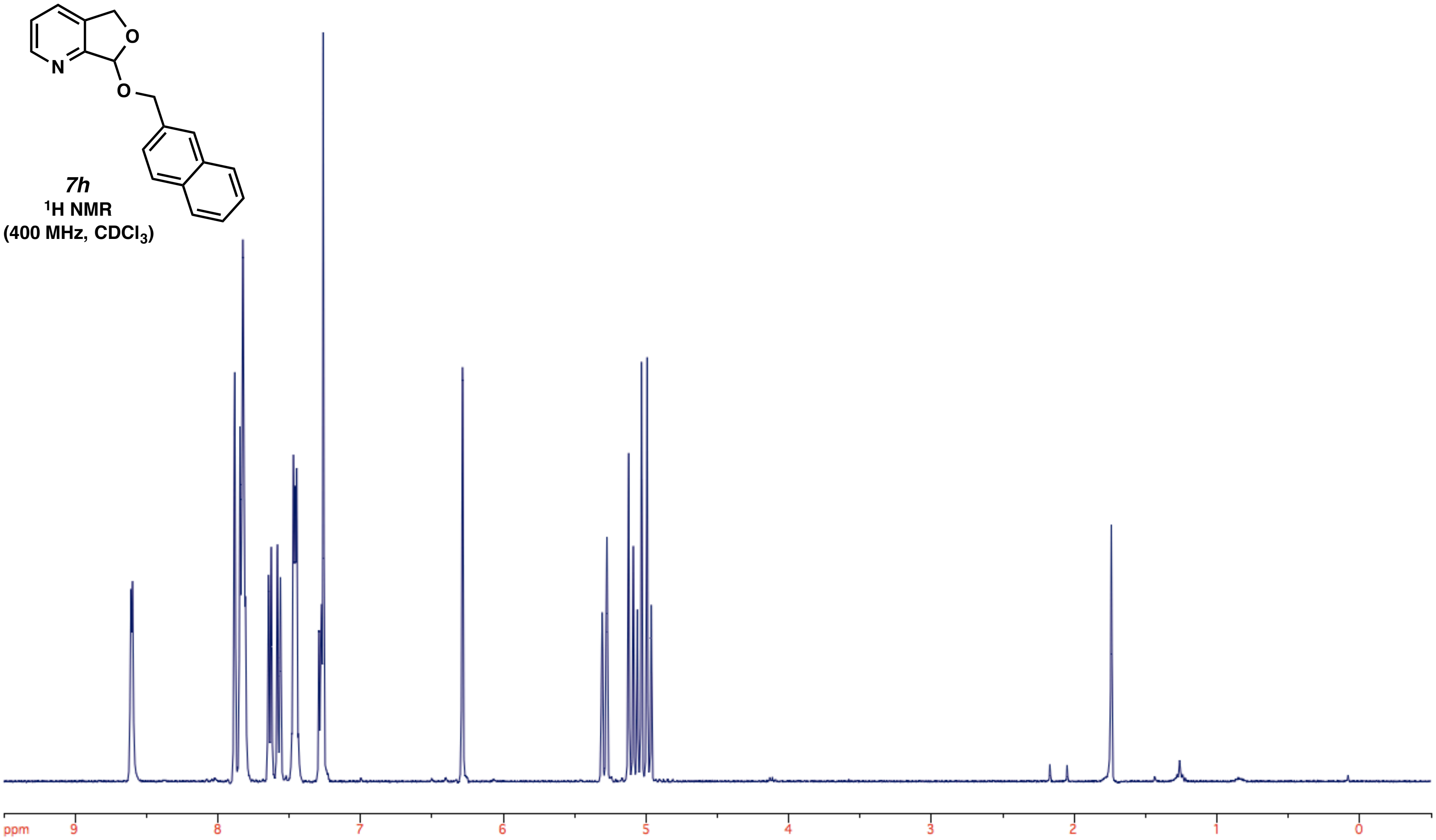

7h
^{1}H NMR
(400 MHz, $CDCl_3$)

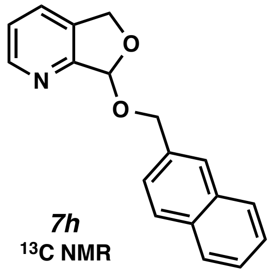

7h
^{13}C **NMR**
(100 MHz, $CDCl_3$)

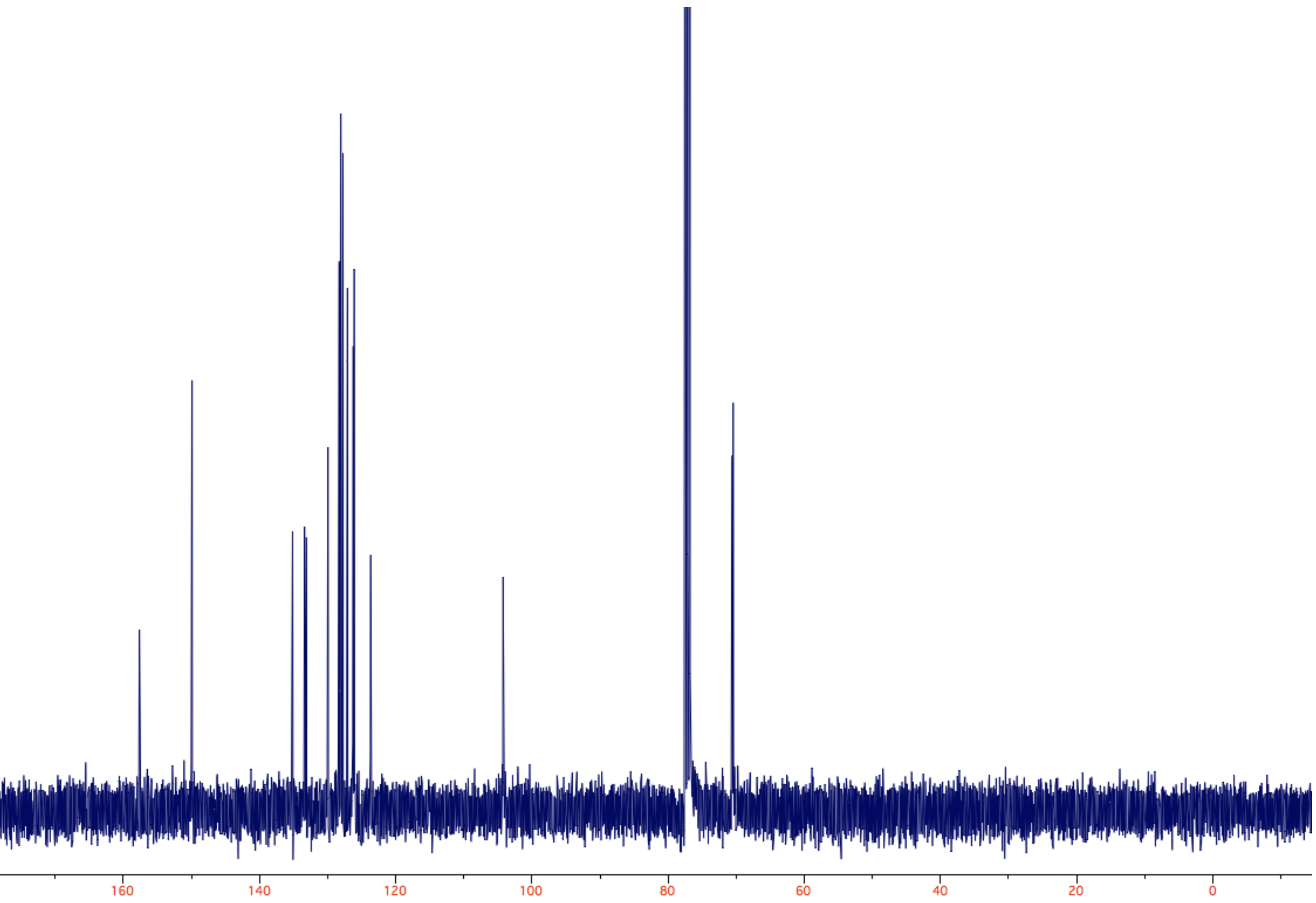

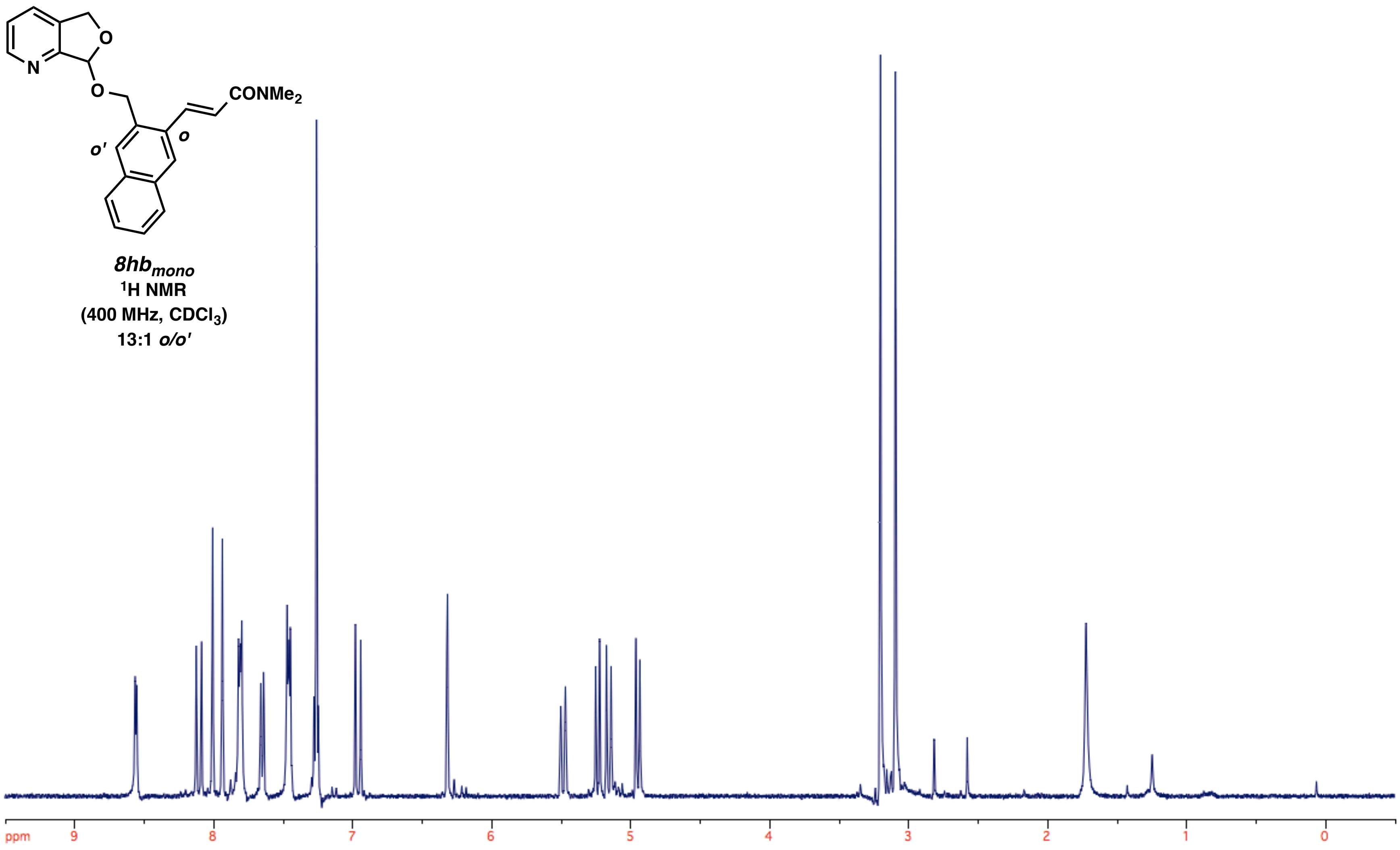

***8hb**$_{mono}$*
^{1}H NMR
(400 MHz, $CDCl_3$)
13:1 *o/o'*

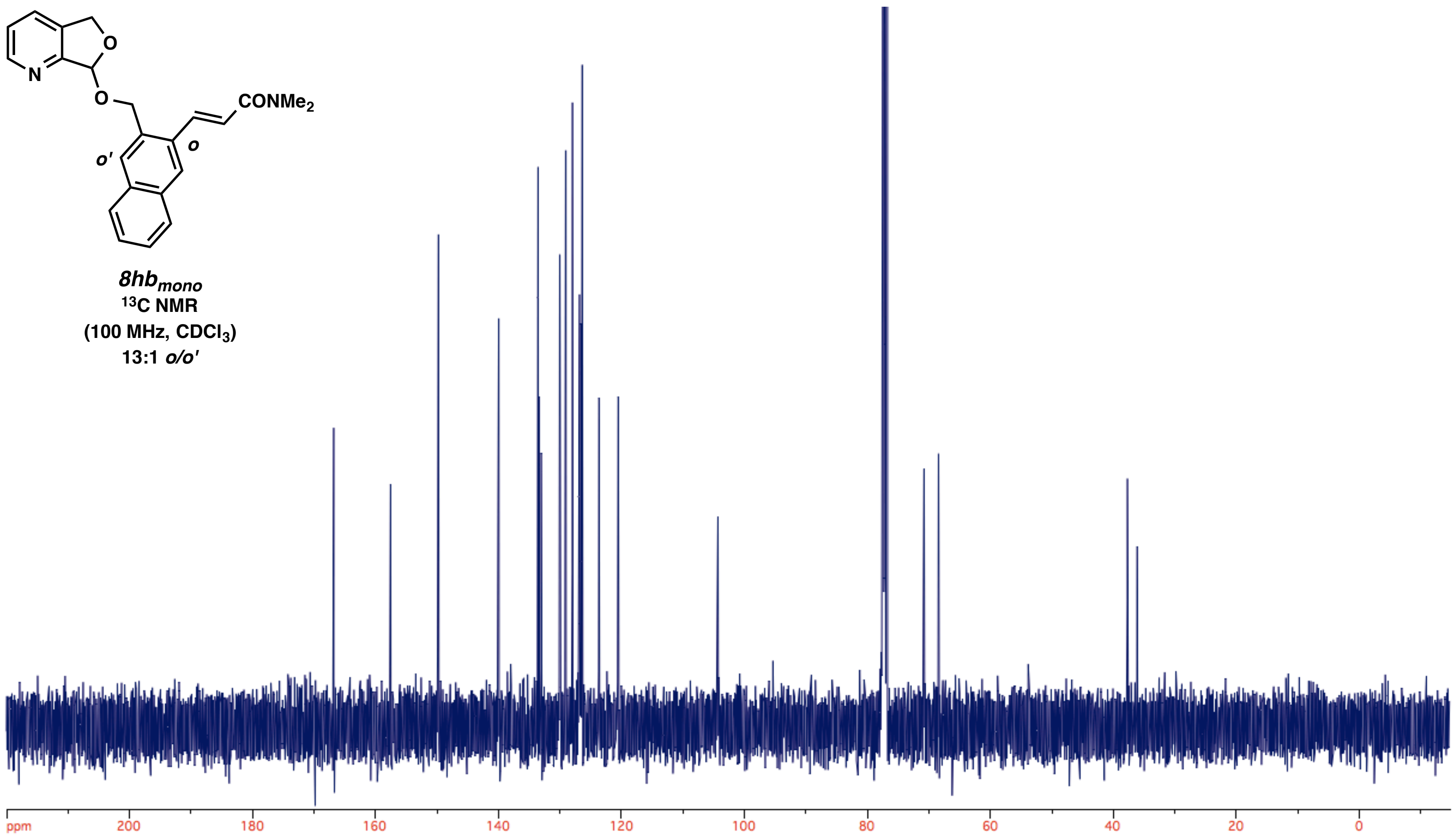

8hb

$8hb_{mono}$

^{13}C NMR

(100 MHz, $CDCl_3$)

13:1 *o/o′*

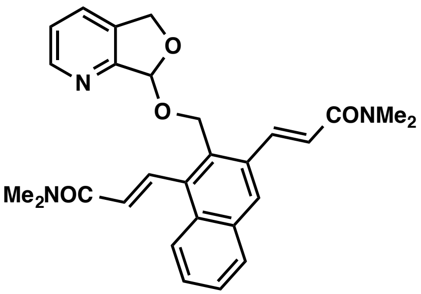

***8hb**$_{di}$*
^{1}H NMR
(400 MHz, $CDCl_3$)

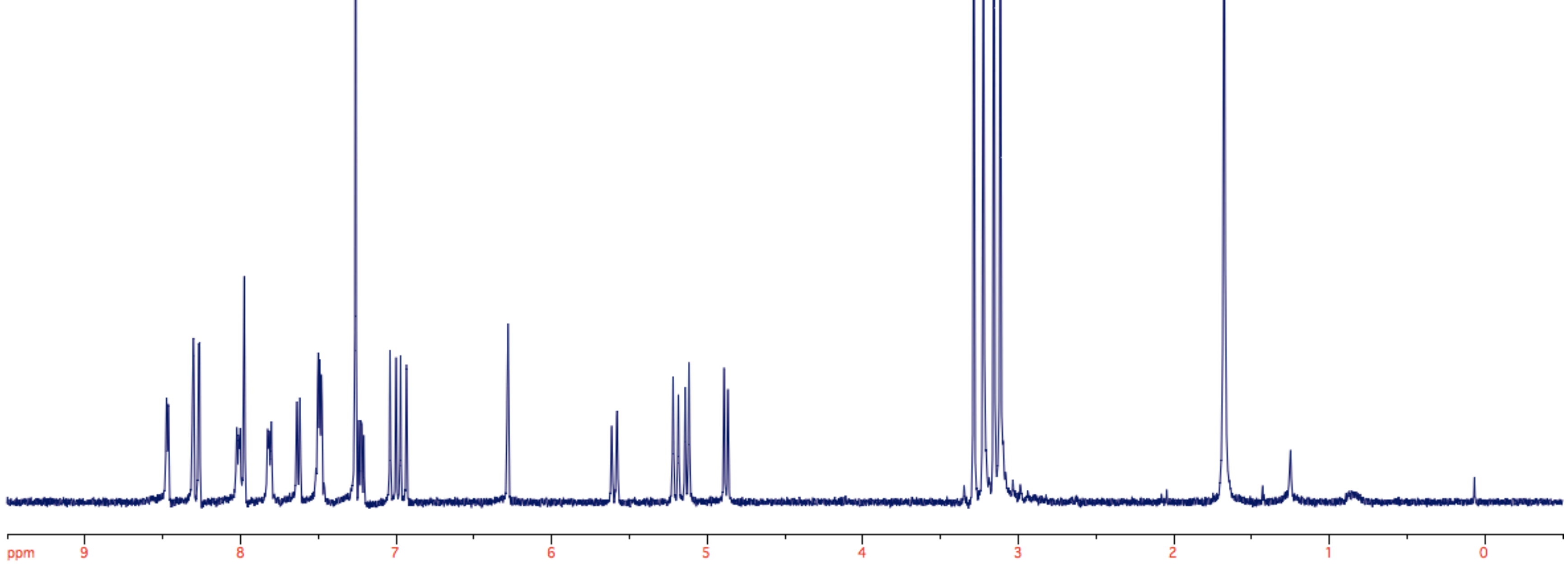

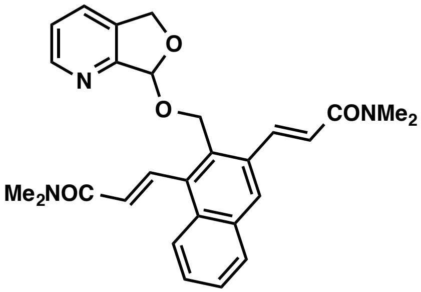

***8hb**$_{di}$*
^{13}C NMR
(125 MHz, $CDCl_3$)

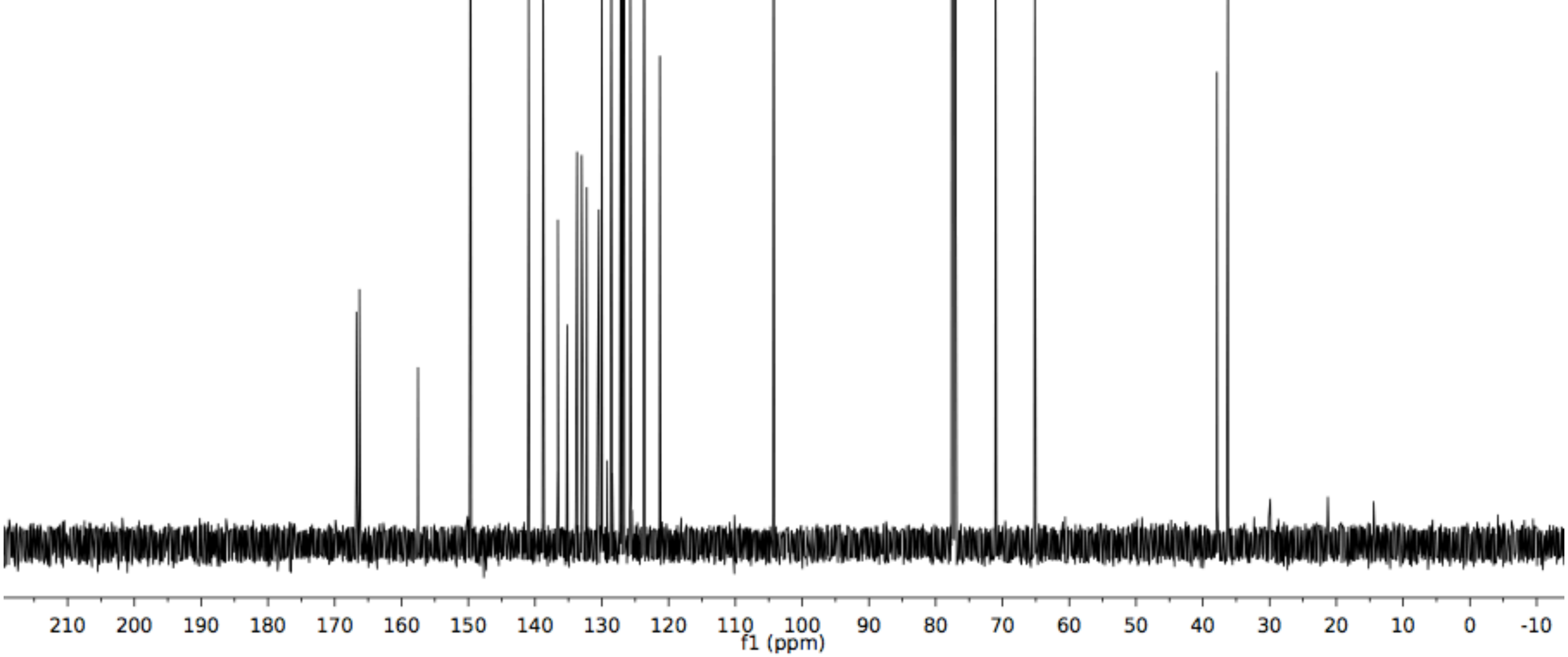

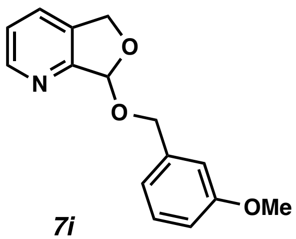

7i
^{1}H NMR
(400 MHz, $CDCl_3$)

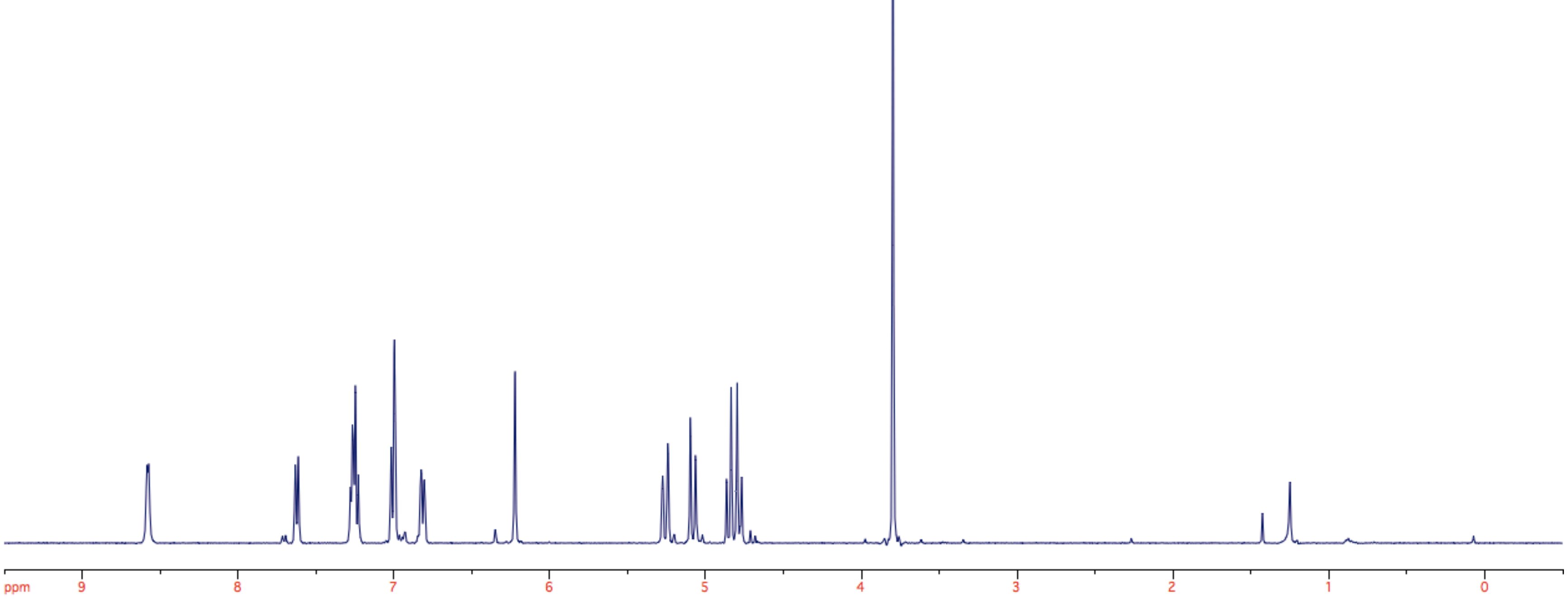

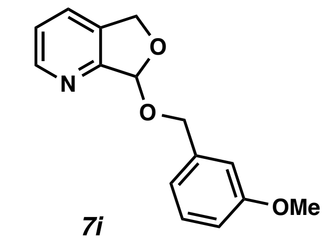

7i
^{13}C NMR
(100 MHz, $CDCl_3$)

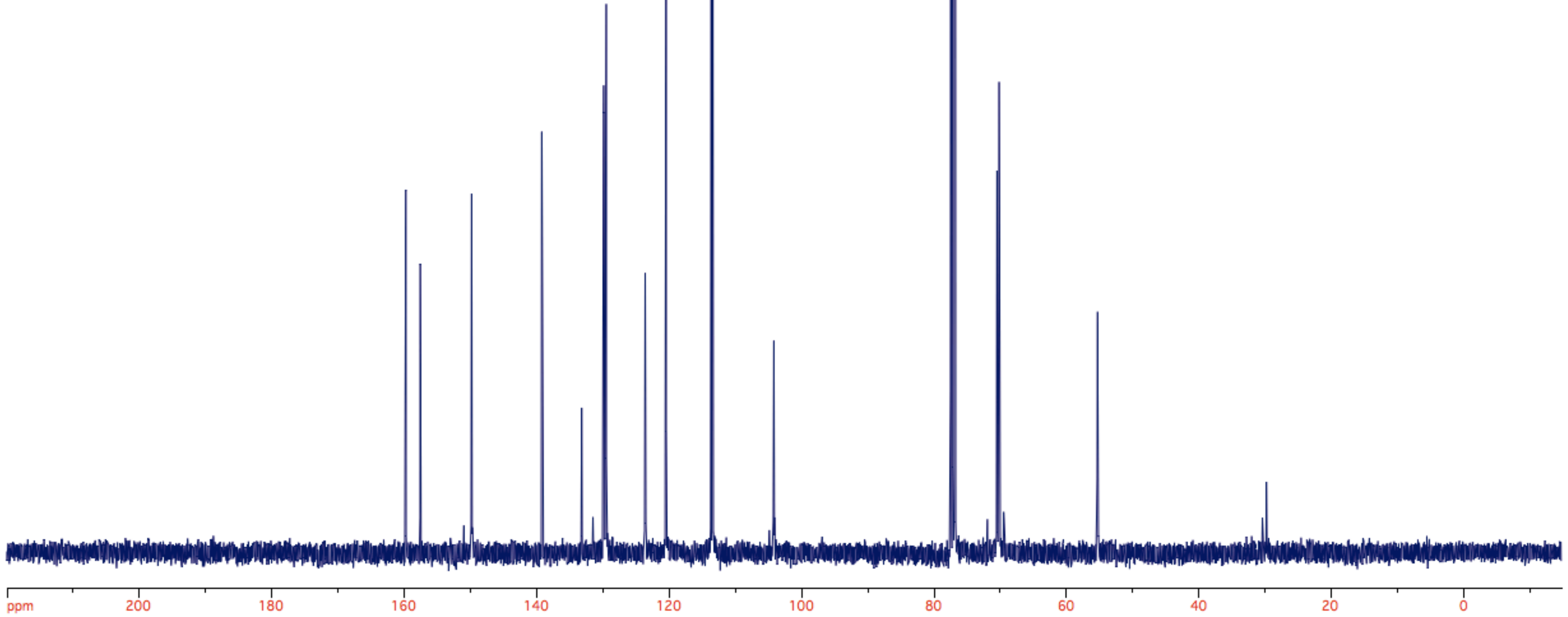

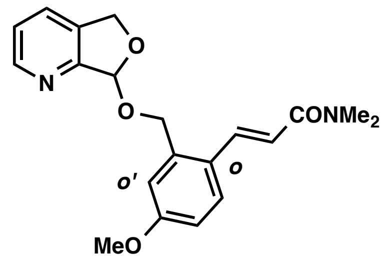

***8ib**$_{mono}$*
^{1}H NMR
(400 MHz, $CDCl_3$)
6.6:1 *o/o′*

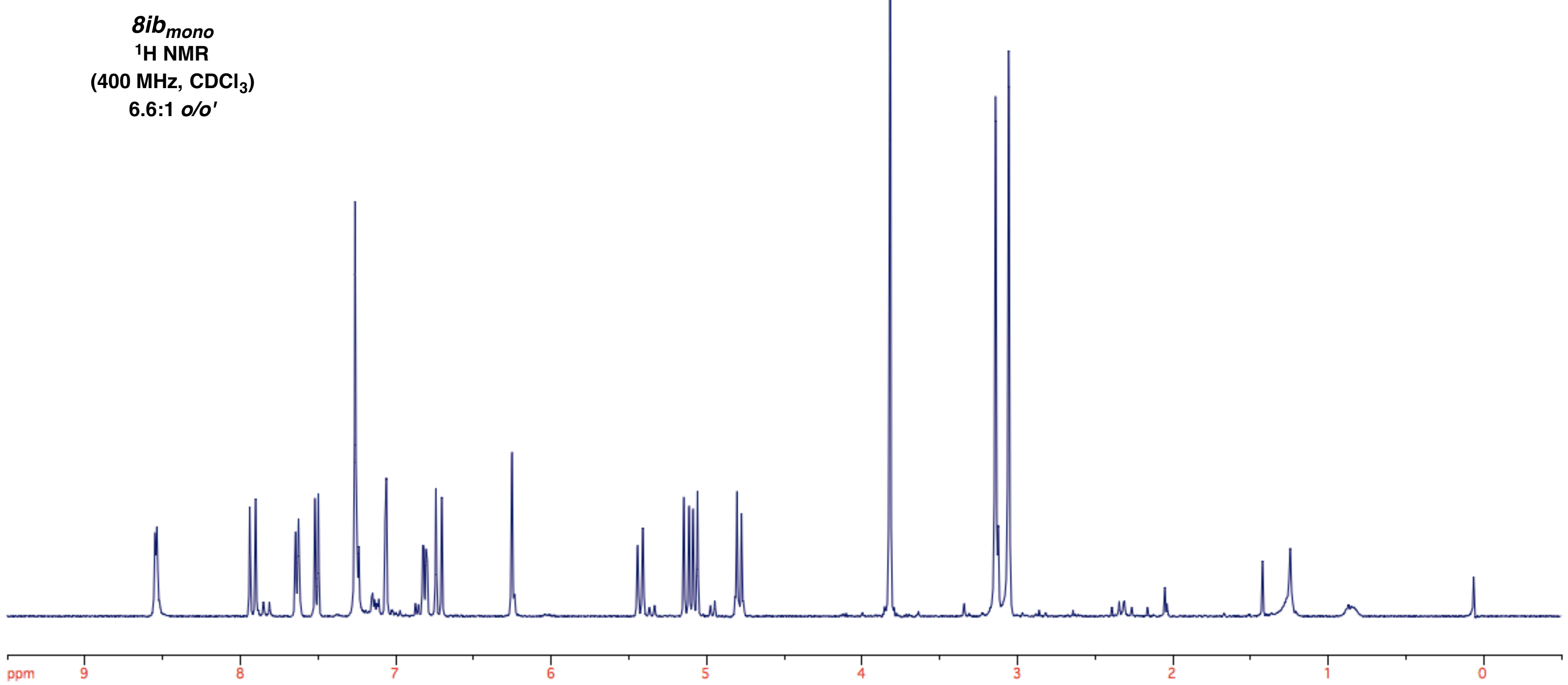

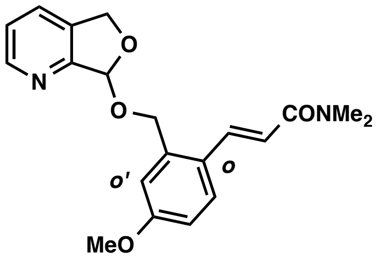

8ib$_{mono}$
^{13}C NMR
(100 MHz, $CDCl_3$)
6.6:1 *o/o'*

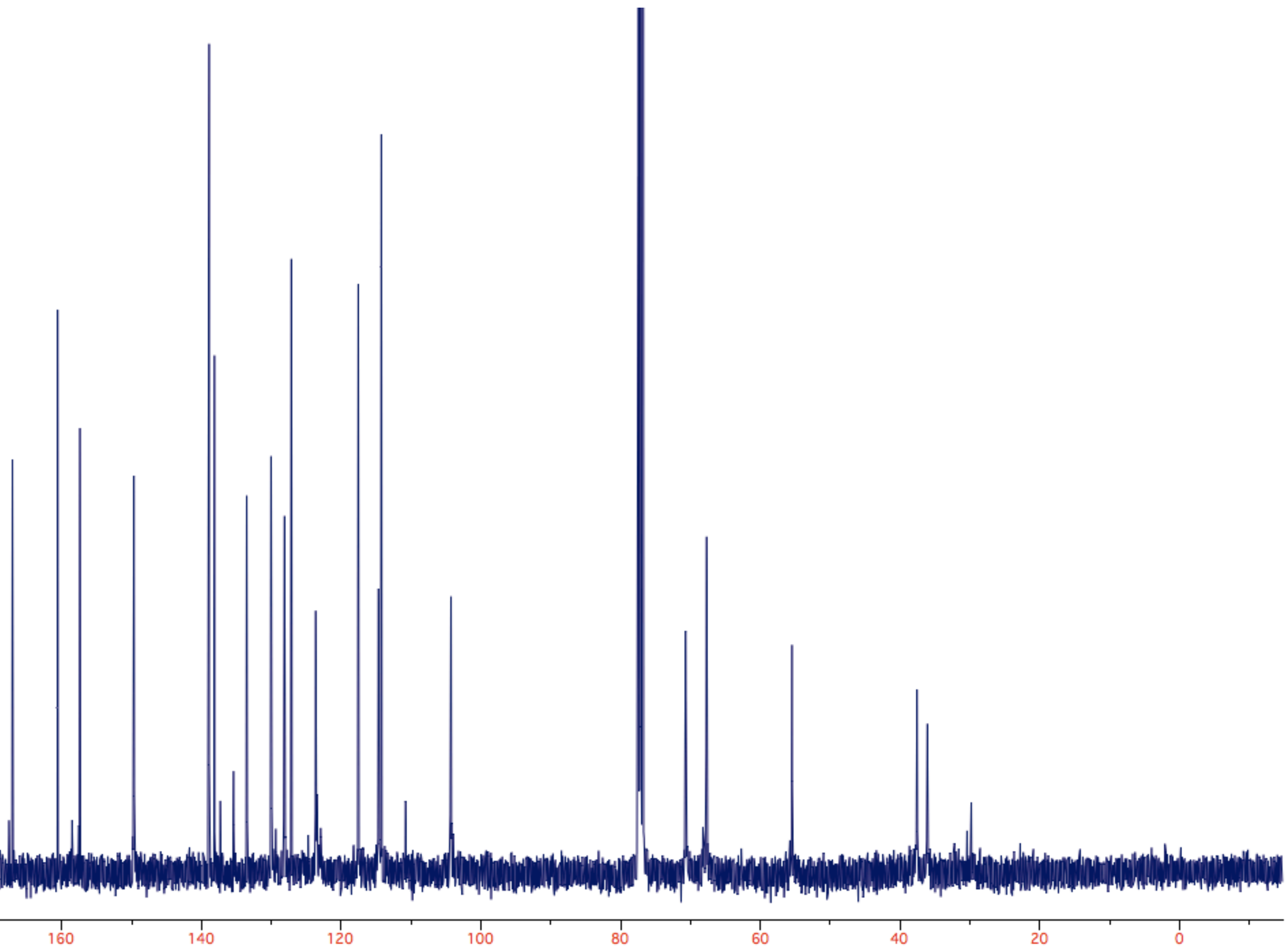

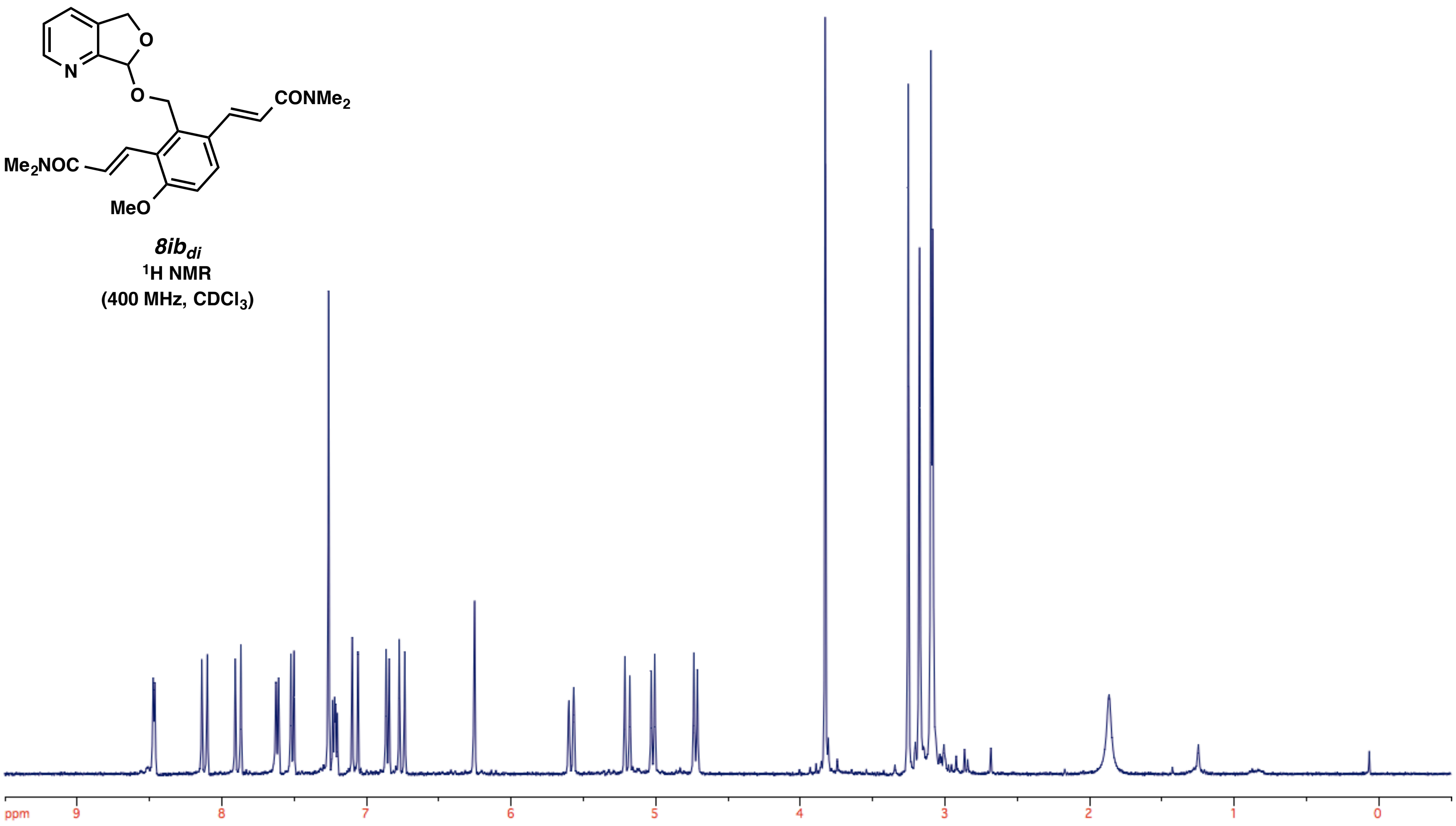
CONMe$_2$
Me$_2$NOC
MeO
N
O
8ib$_{di}$
^{1}H NMR
(400 MHz, CDCl$_3$)
ppm 9 8 7 6 5 4 3 2 1 0

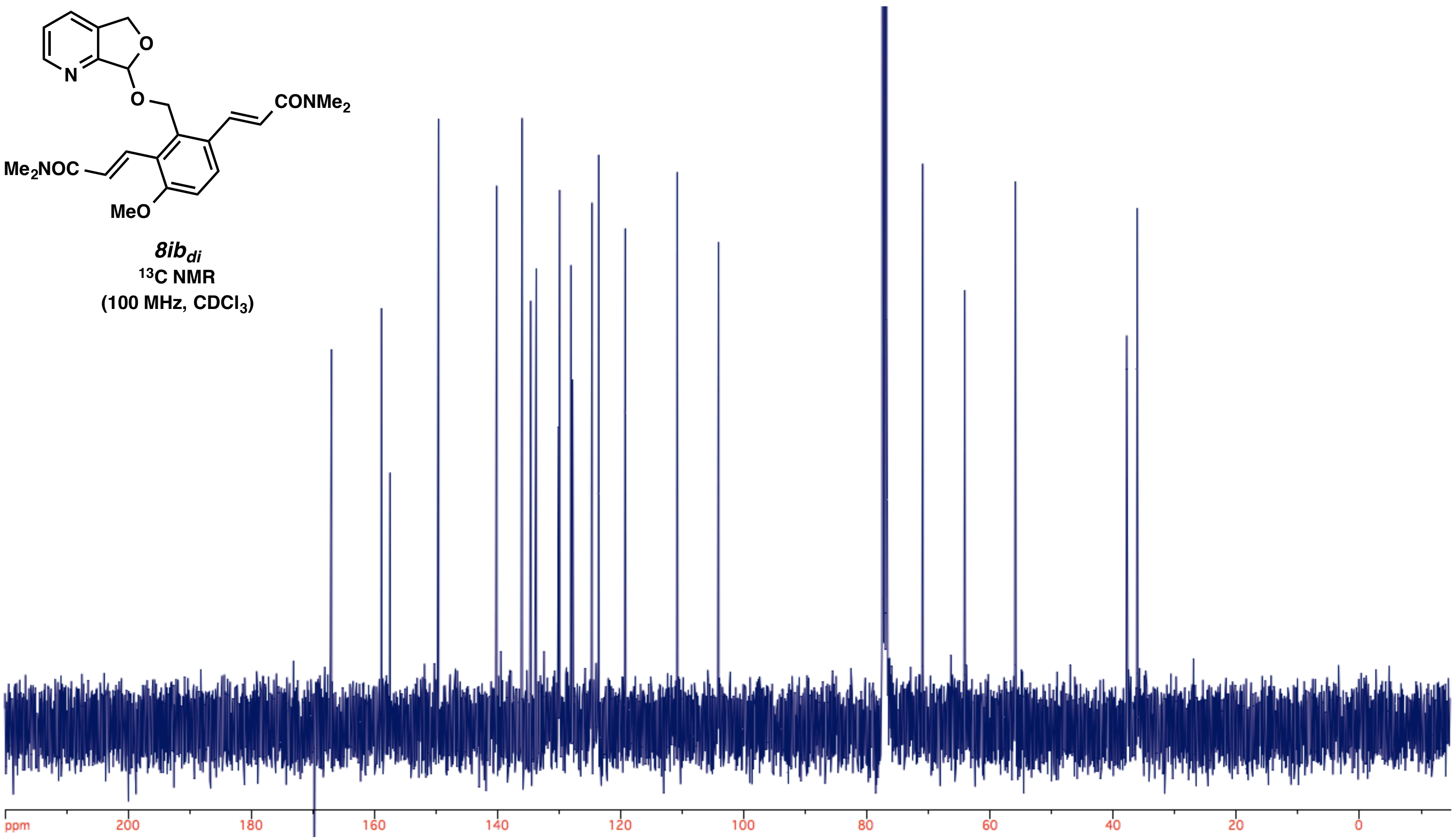

***8ib**$_{di}$*
^{13}C NMR
(100 MHz, $CDCl_3$)

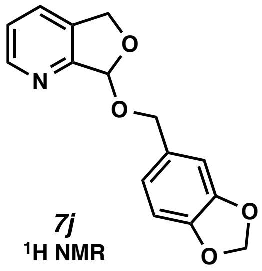

7j
^1H NMR
(400 MHz, $CDCl_3$)

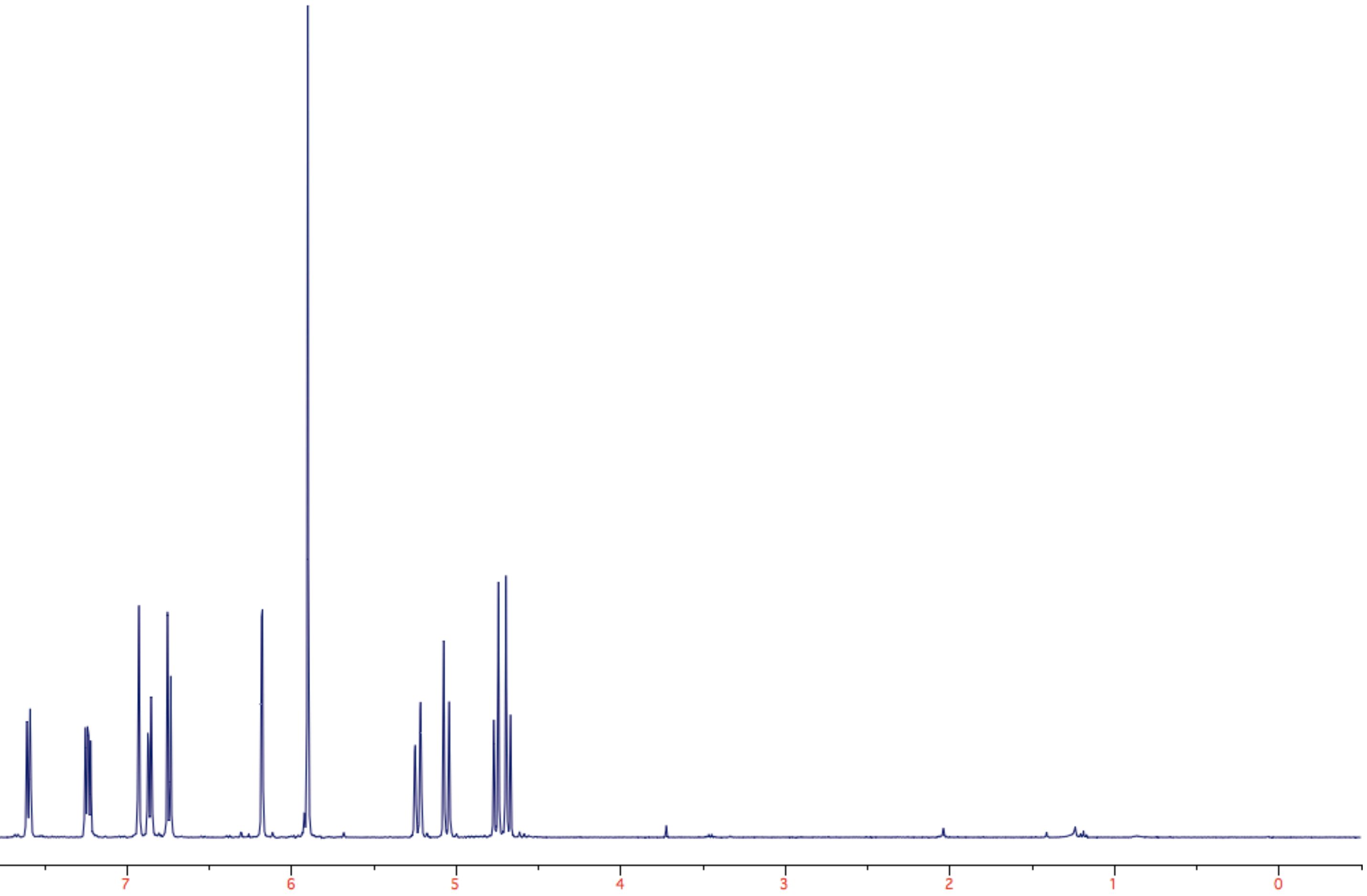

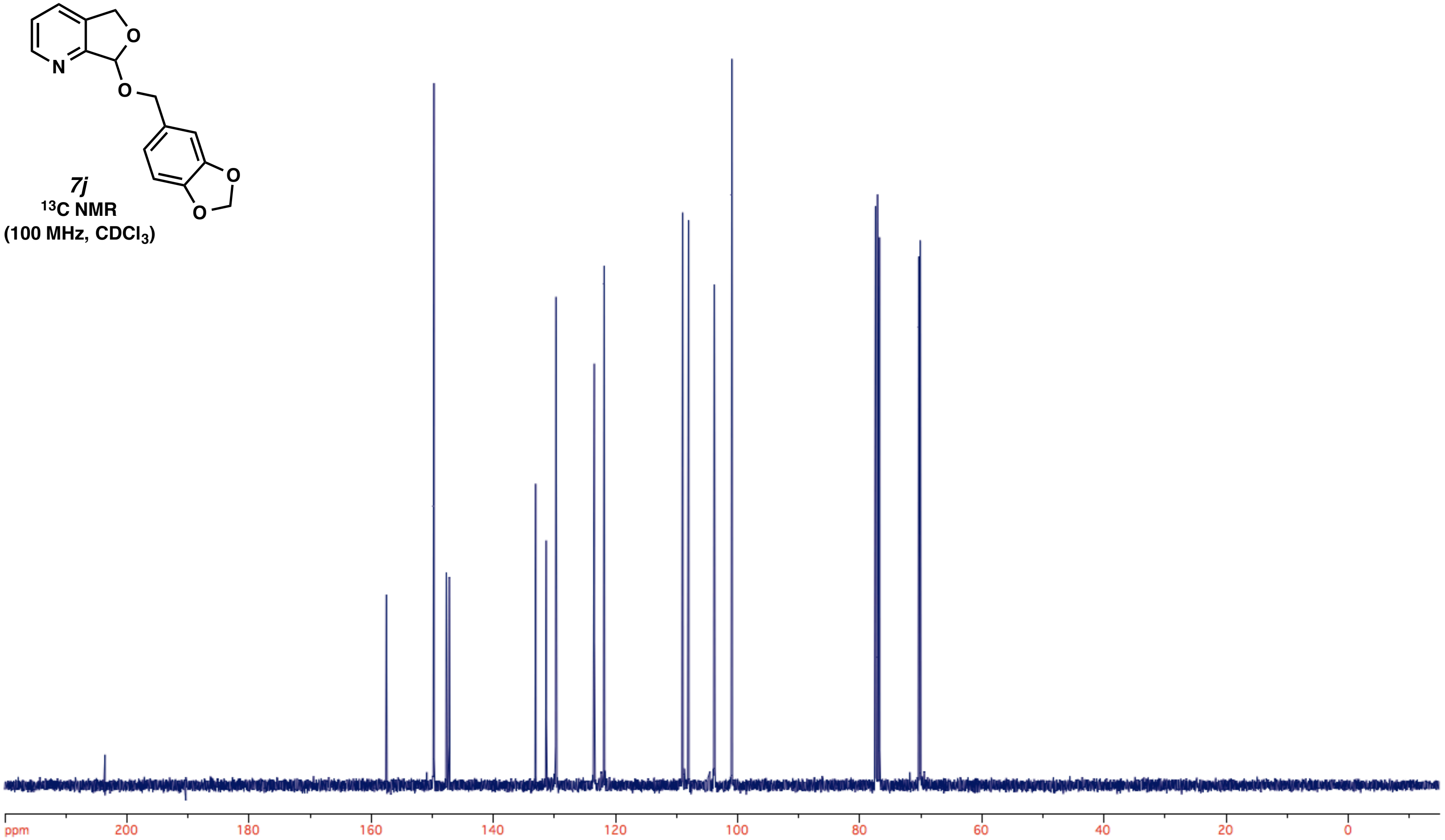

7j
^{13}C NMR
(100 MHz, $CDCl_3$)

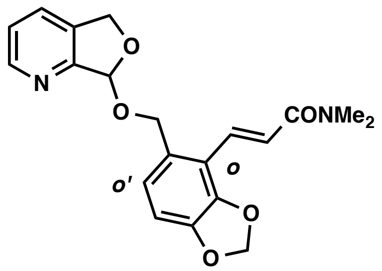

8jb$_{mono}$
^{1}H NMR
(400 MHz, $CDCl_3$)
1.9:1 *o/o'*

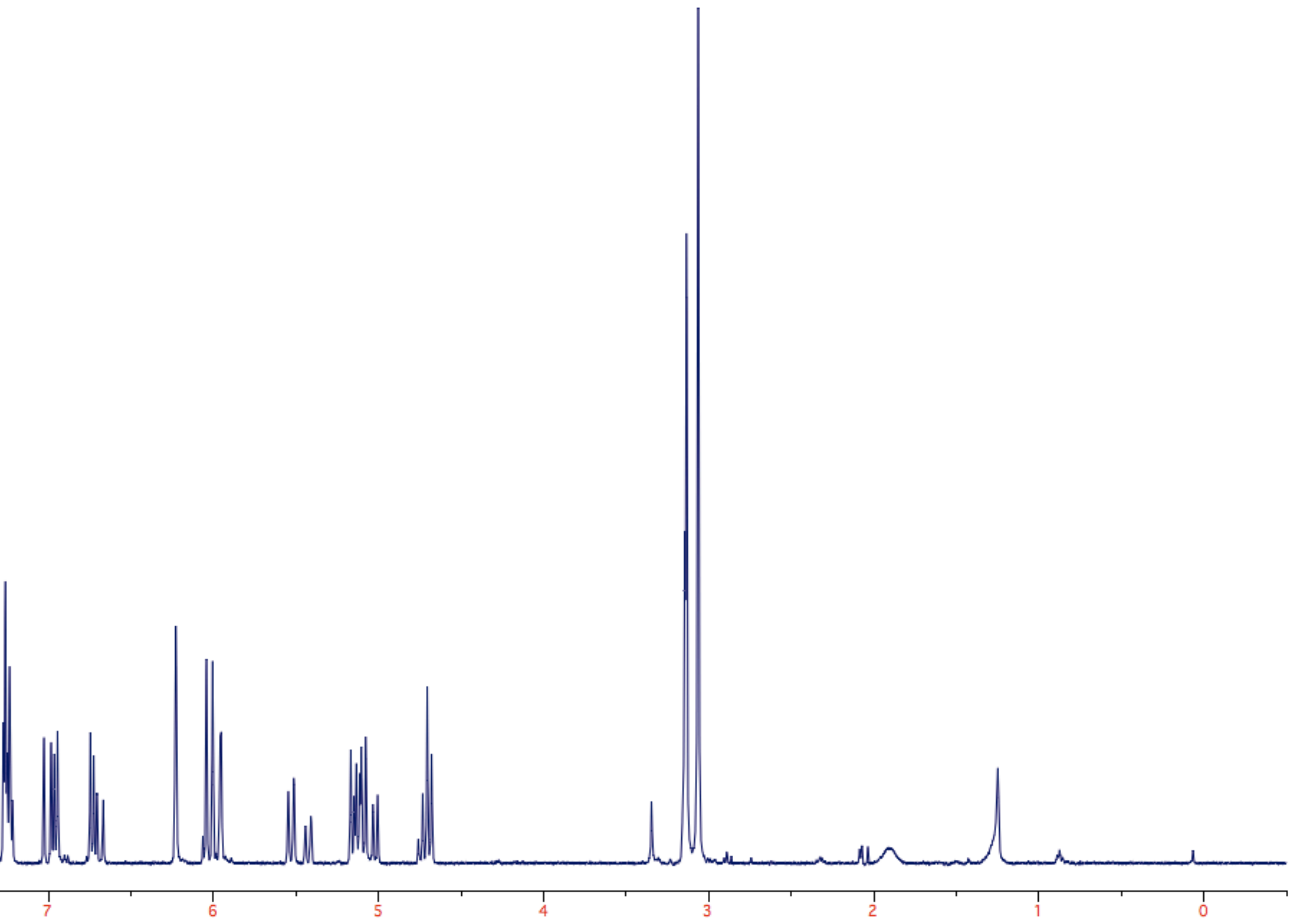

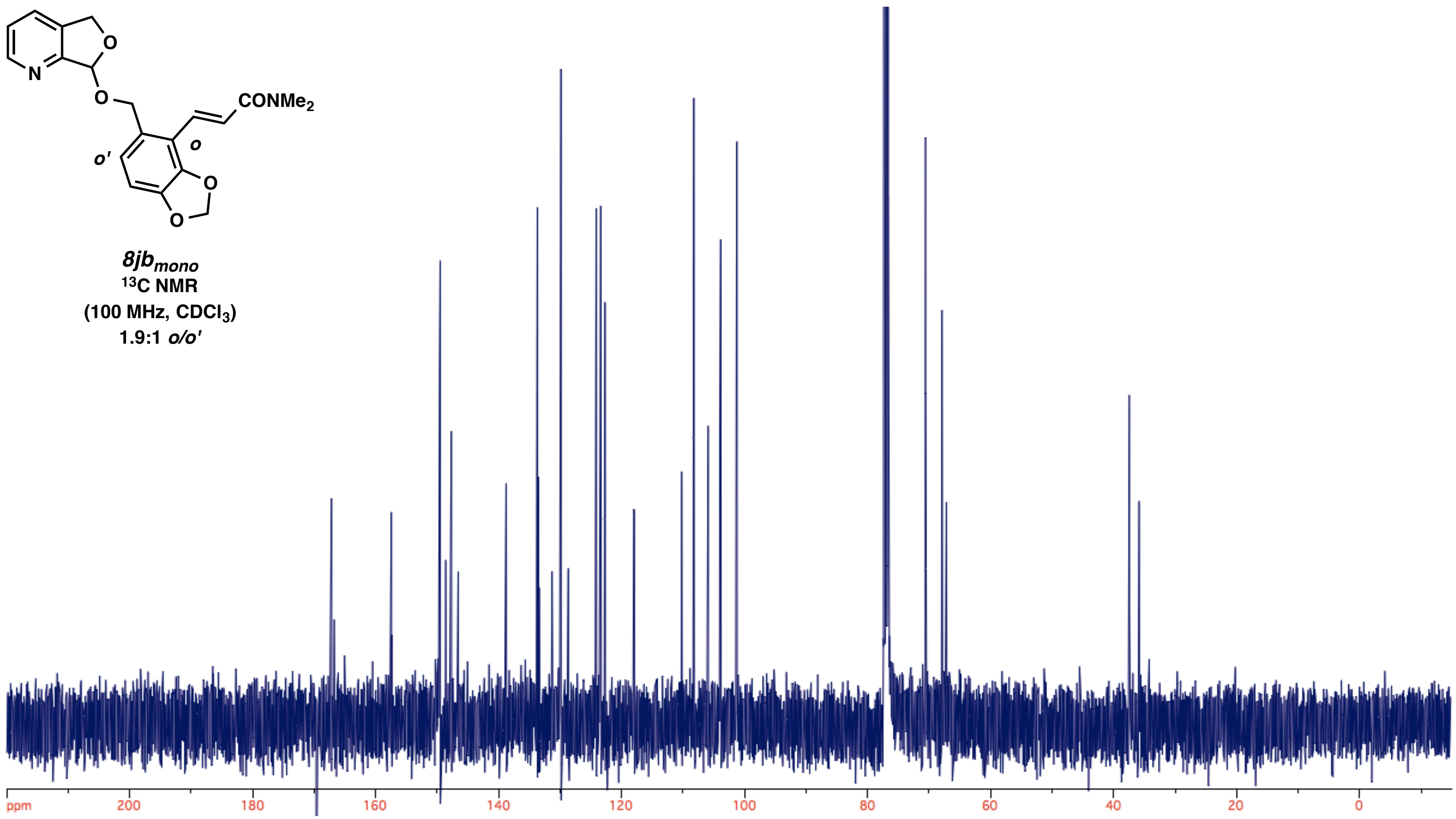
N
O
O
CONMe$_2$
o'
o
O
O
8jb$_{mono}$
^{13}C NMR
(100 MHz, $CDCl_3$)
1.9:1 o/o'
ppm
200
180
160
140
120
100
80
60
40
20
0

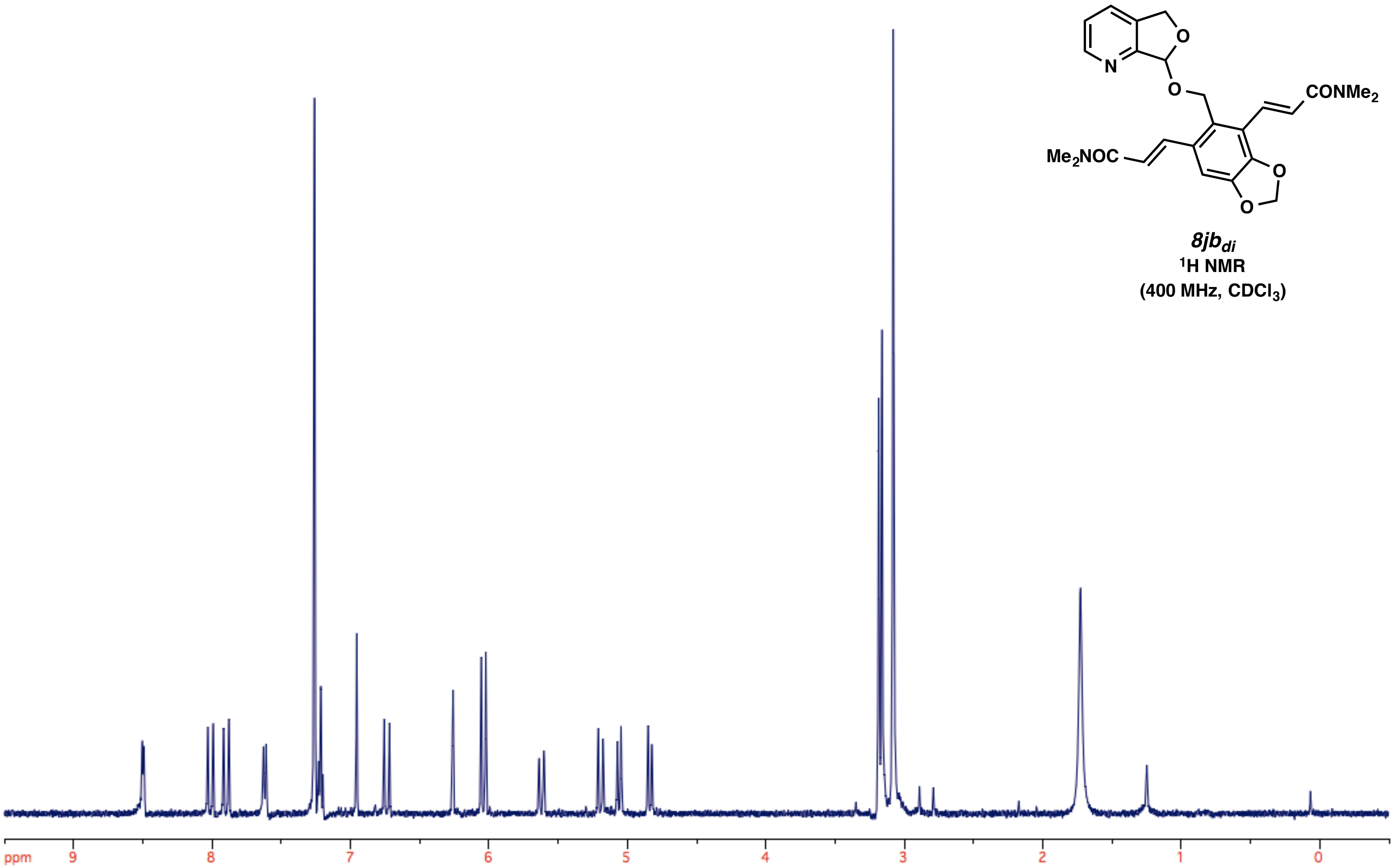

8jb*di*

^{1}H NMR

(400 MHz, CDCl$_3$)

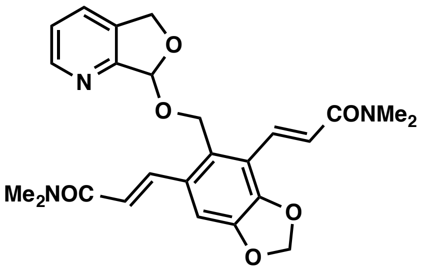

***8jb*$_{di}$**
^{13}C NMR
(125 MHz, $CDCl_3$)

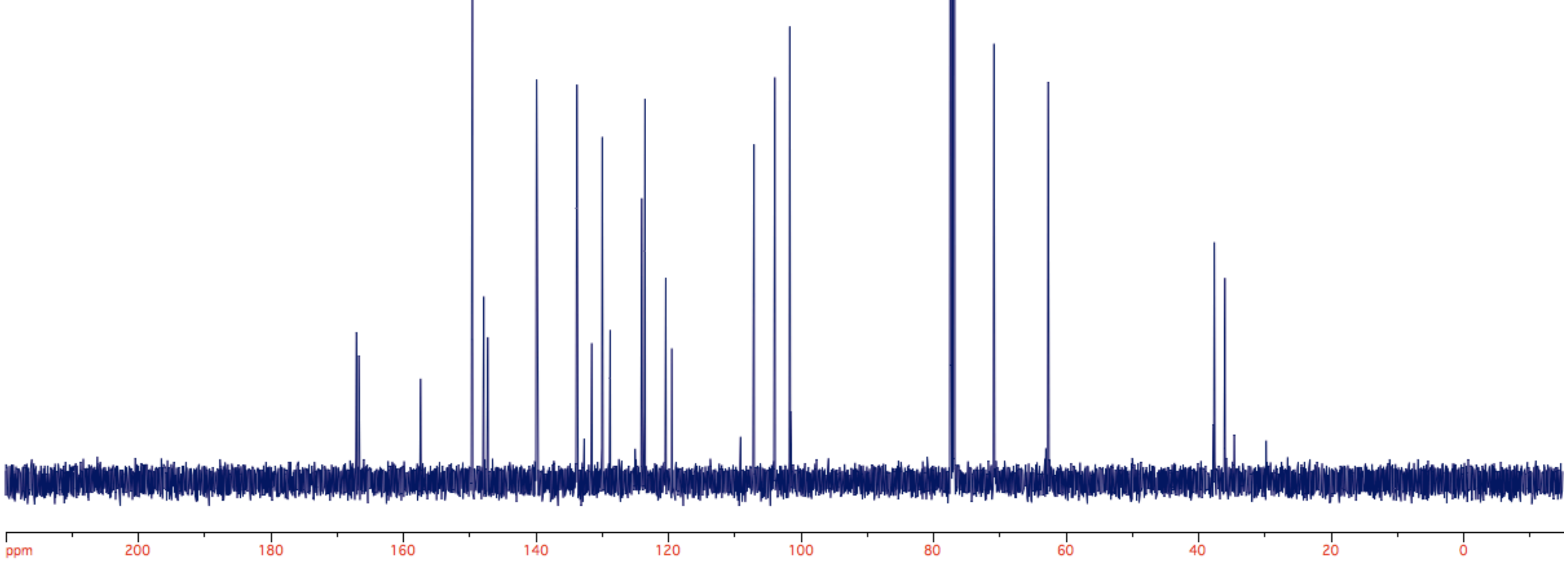

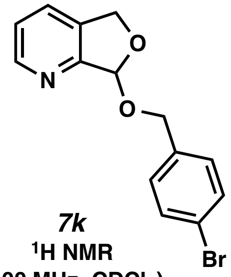

7k
^{1}H NMR
(400 MHz, CDCl$_3$)

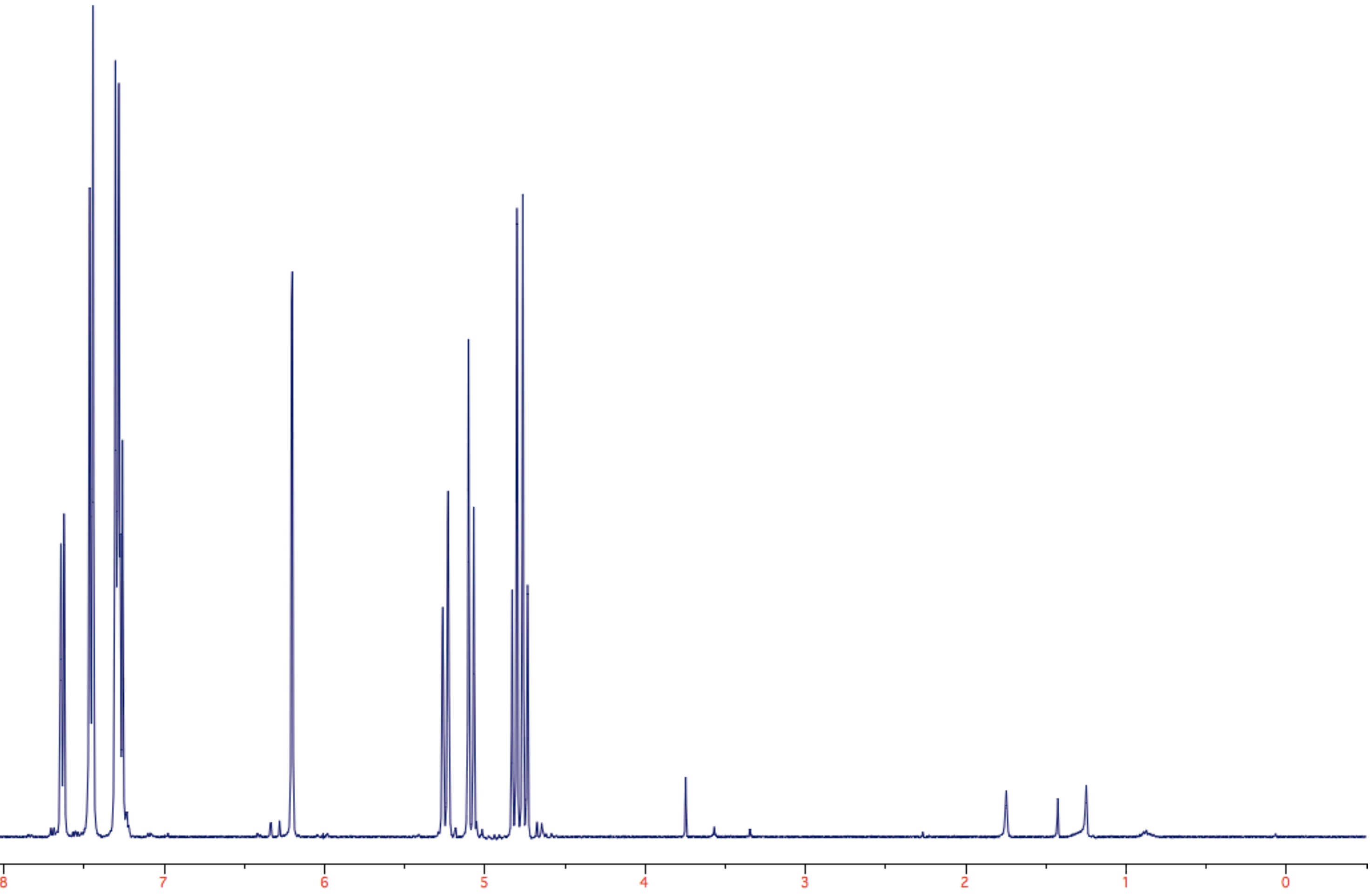

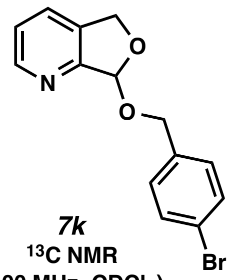

7k
^{13}C NMR
(100 MHz, $CDCl_3$)

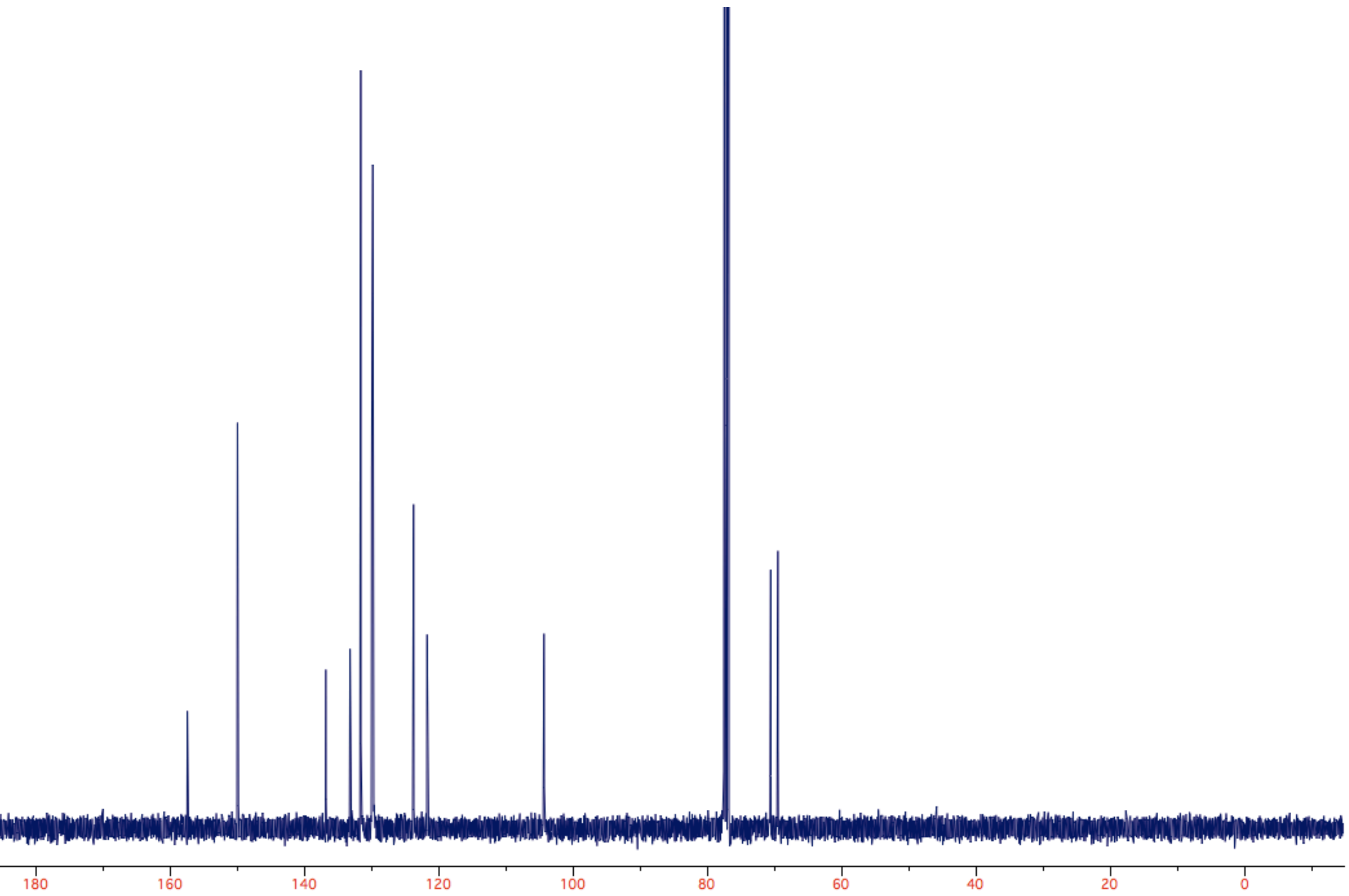

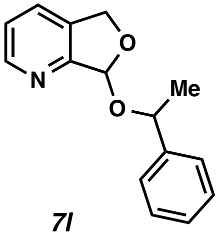

7l
(major diastereomer)
^{1}H NMR
(400 MHz, $CDCl_3$)

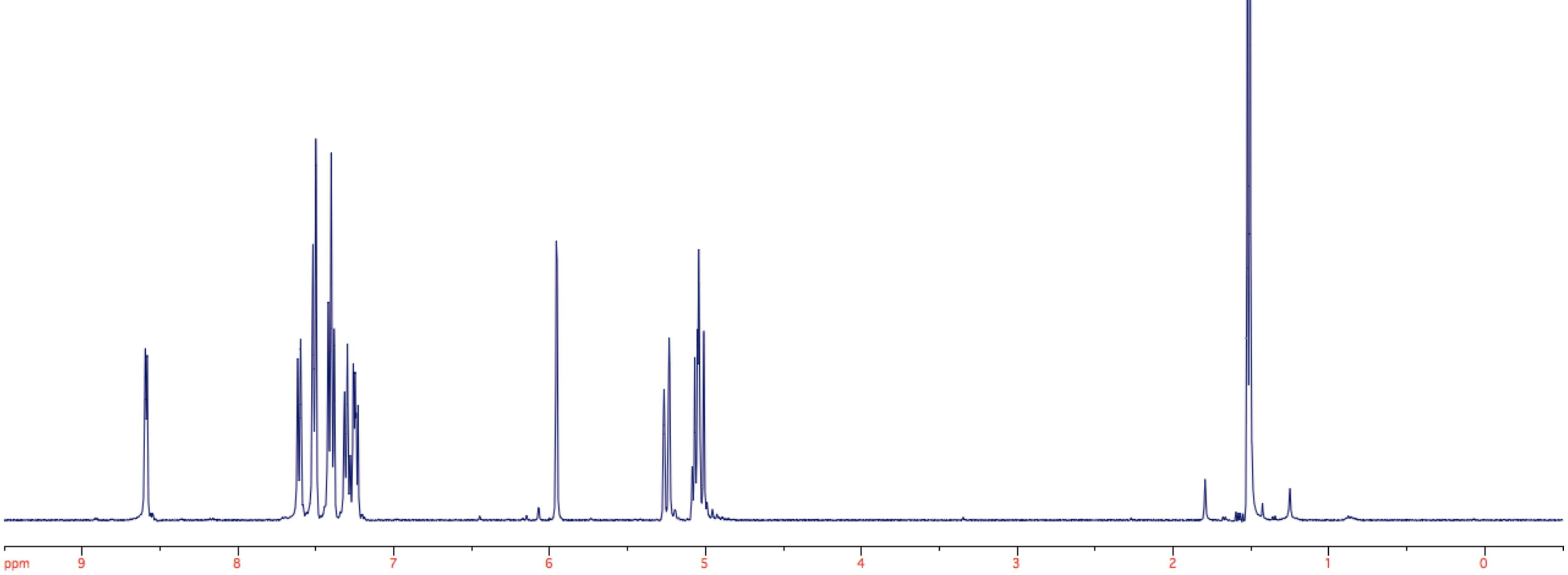

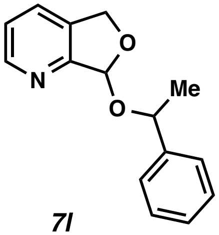

7l
(major diastereomer)
^{13}C NMR
(100 MHz, $CDCl_3$)

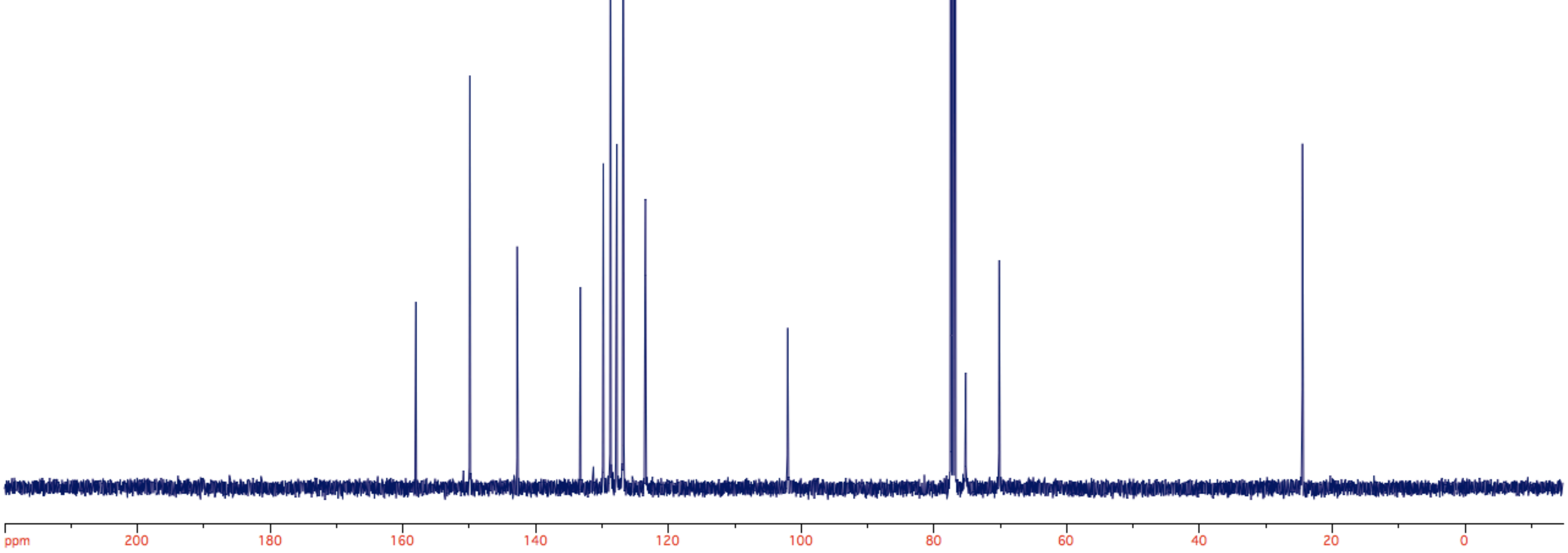

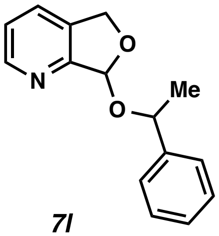

7l
(minor diastereomer)
^{1}H NMR
(400 MHz, $CDCl_3$)

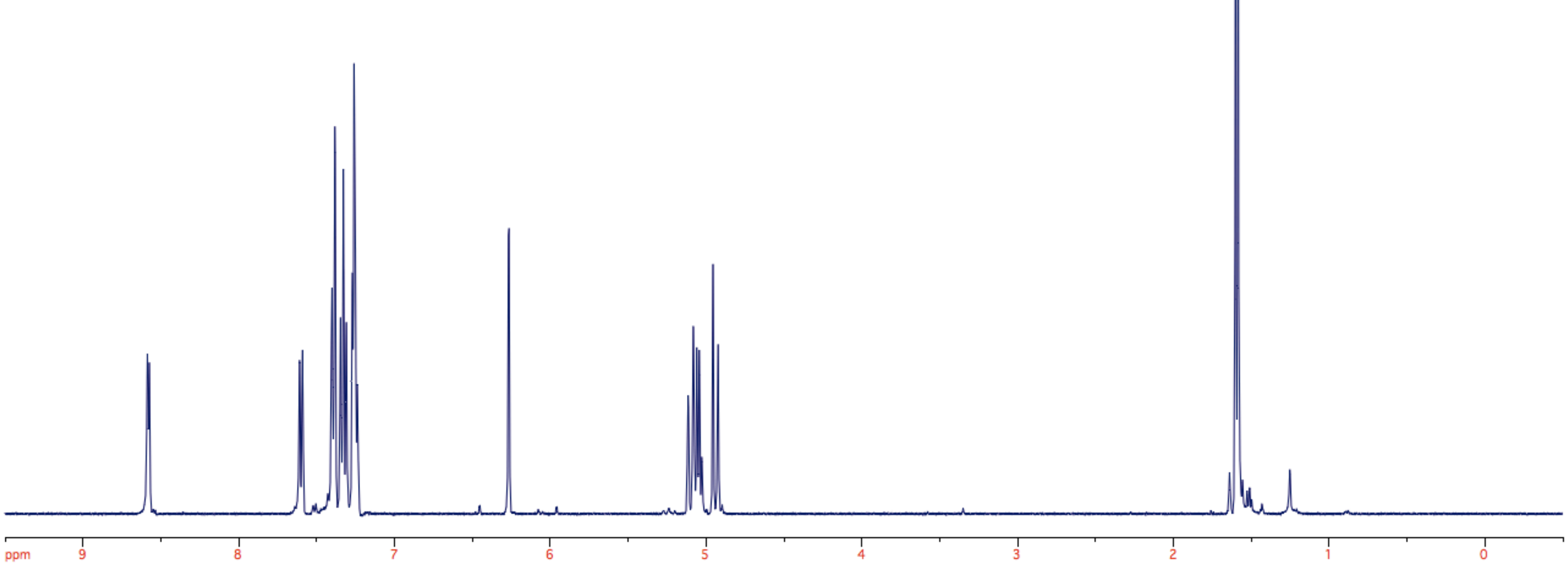

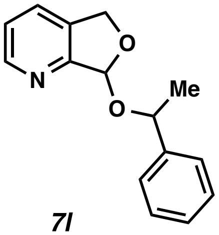

7l
(minor diastereomer)
^{13}C NMR
(100 MHz, $CDCl_3$)

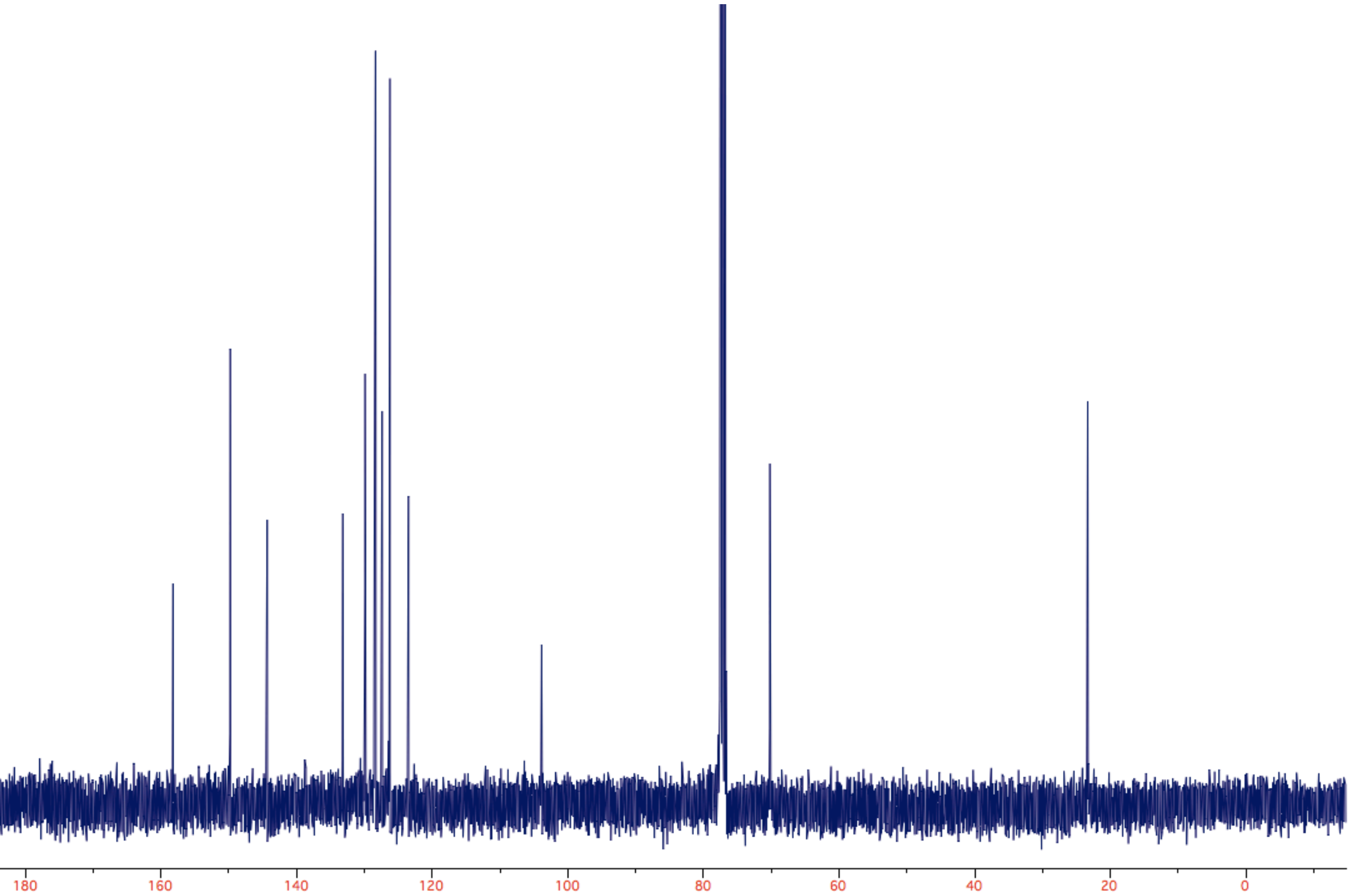

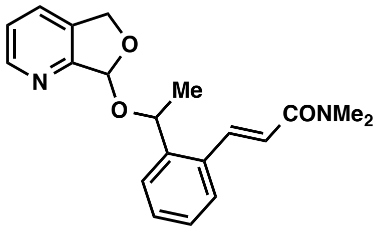

8lb

^{1}H NMR

(400 MHz, $CDCl_3$)

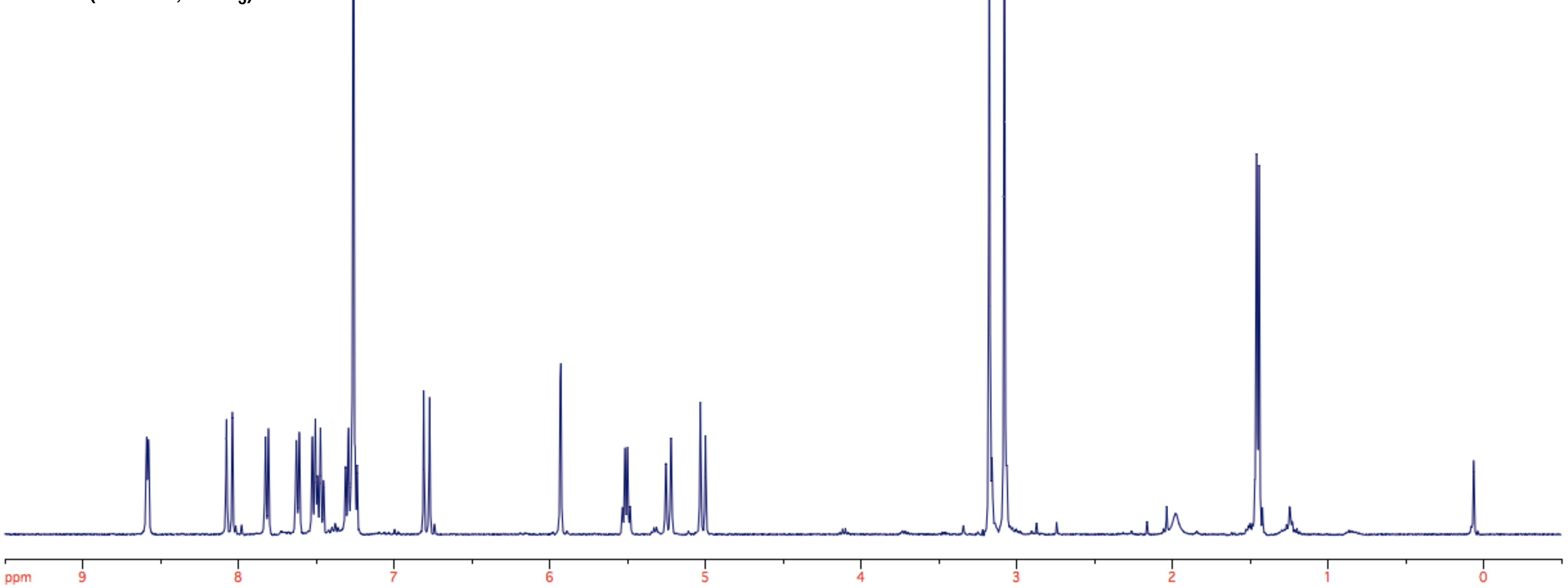

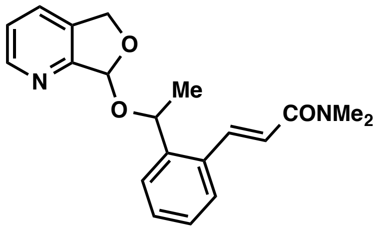

8lb
^{13}C NMR
(100 MHz, $CDCl_3$)

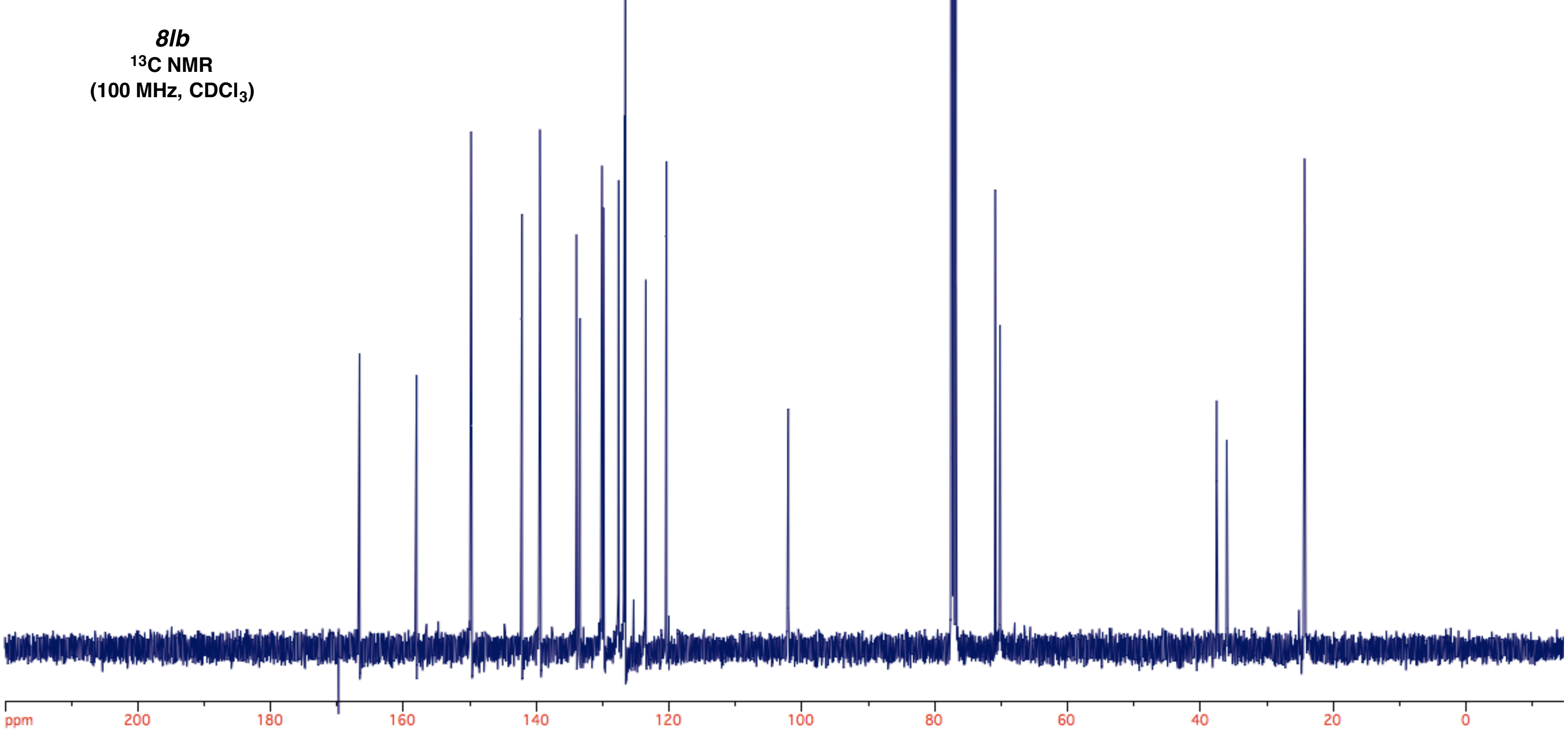

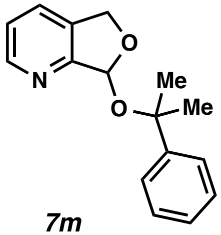

7m

^{1}H NMR

(400 MHz, $CDCl_3$)

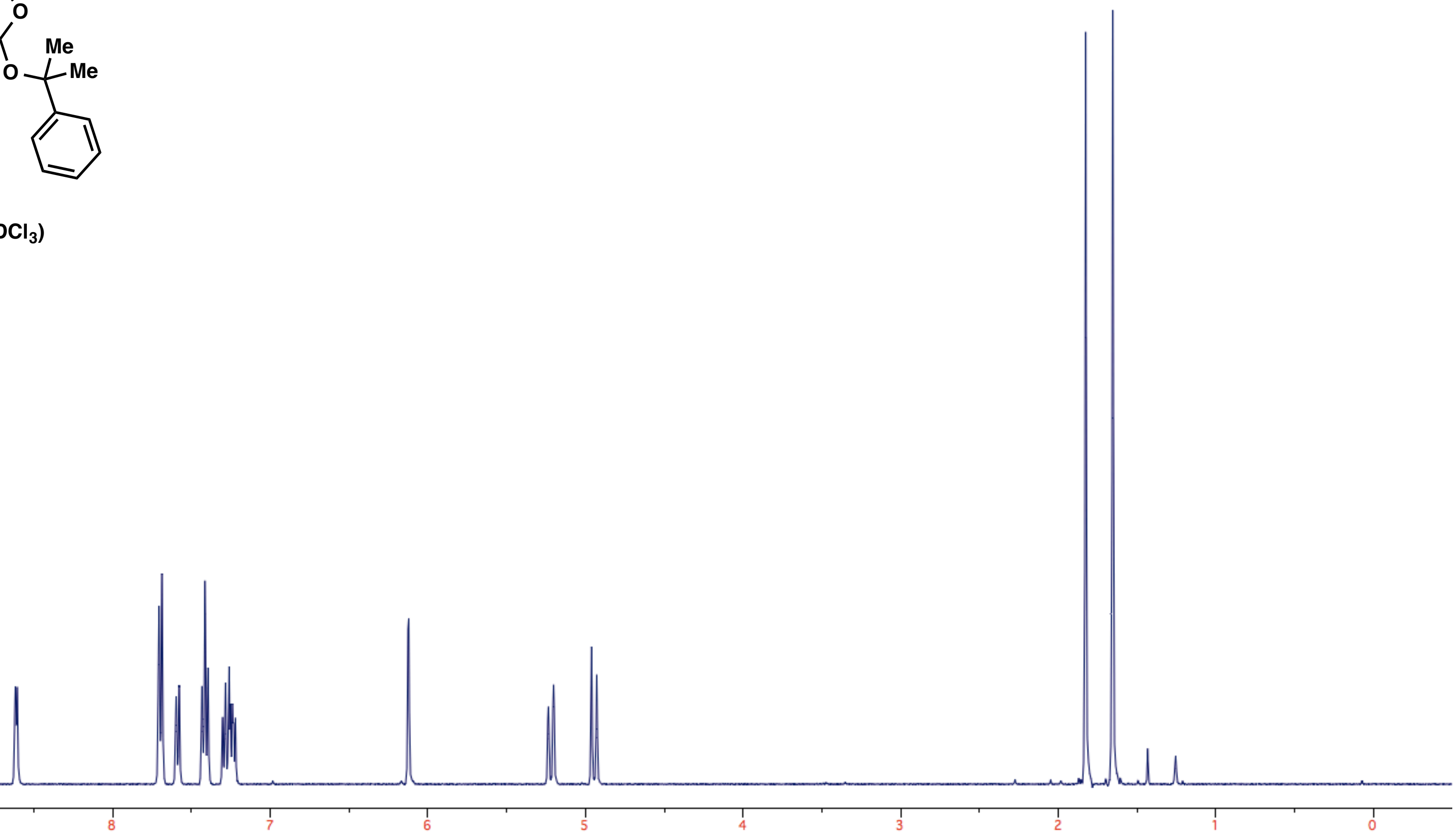

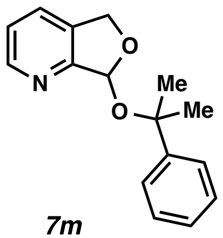

7m

^{13}C NMR

(100 MHz, $CDCl_3$)

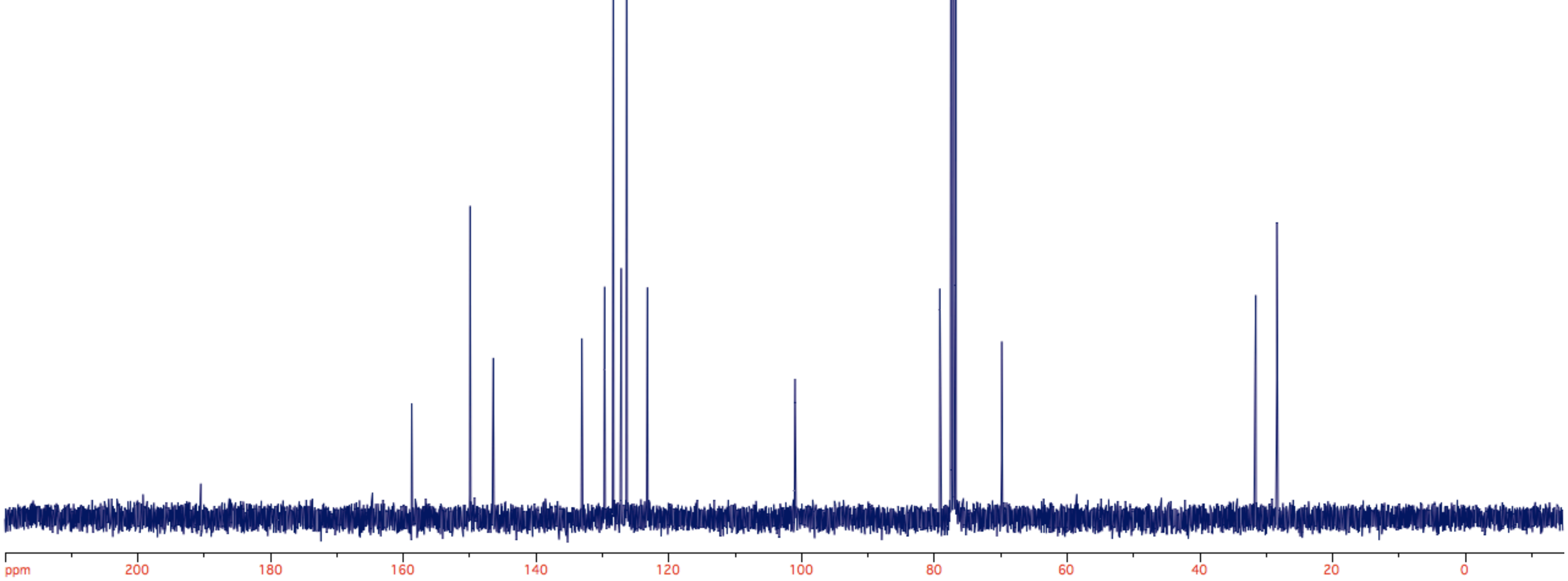

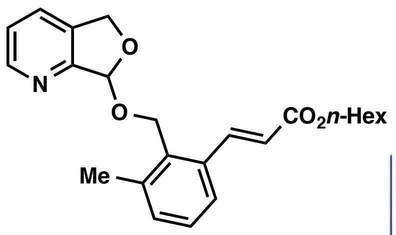

8bc
^{1}H NMR
(400 MHz, $CDCl_3$)

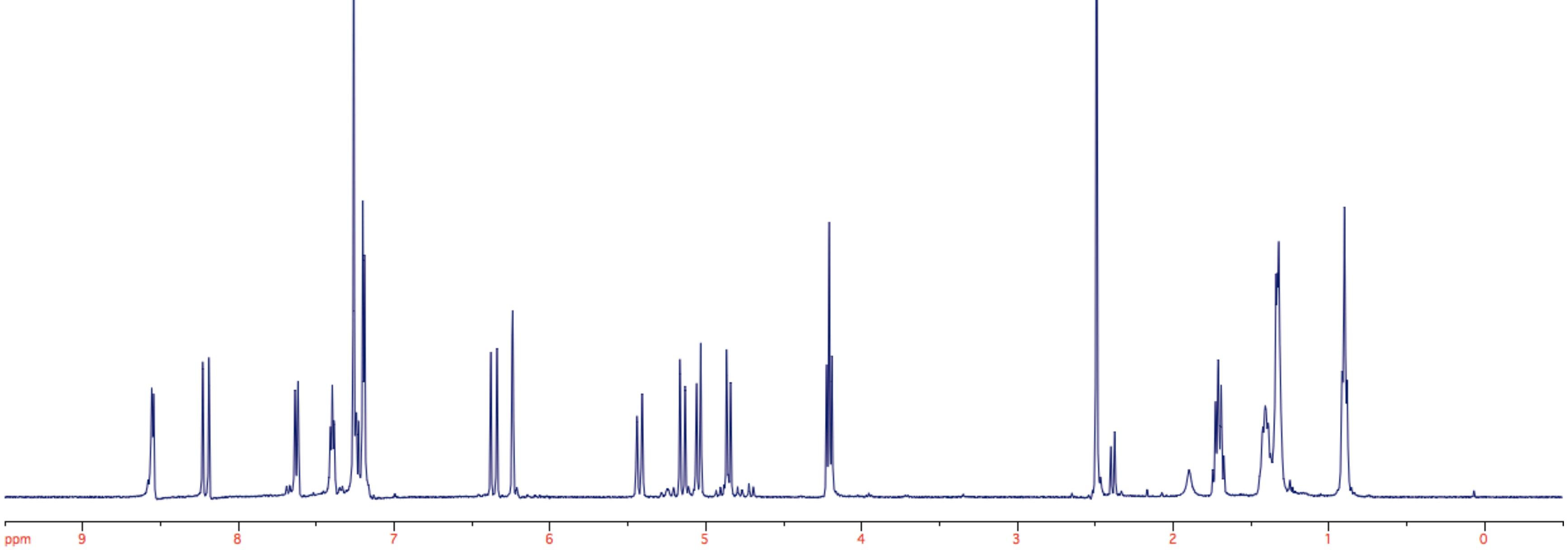

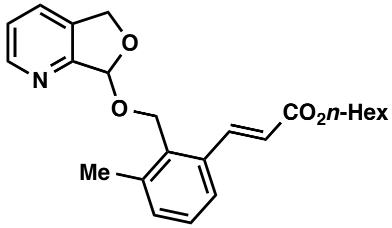

8bc

^{13}C NMR

(100 MHz, $CDCl_3$)

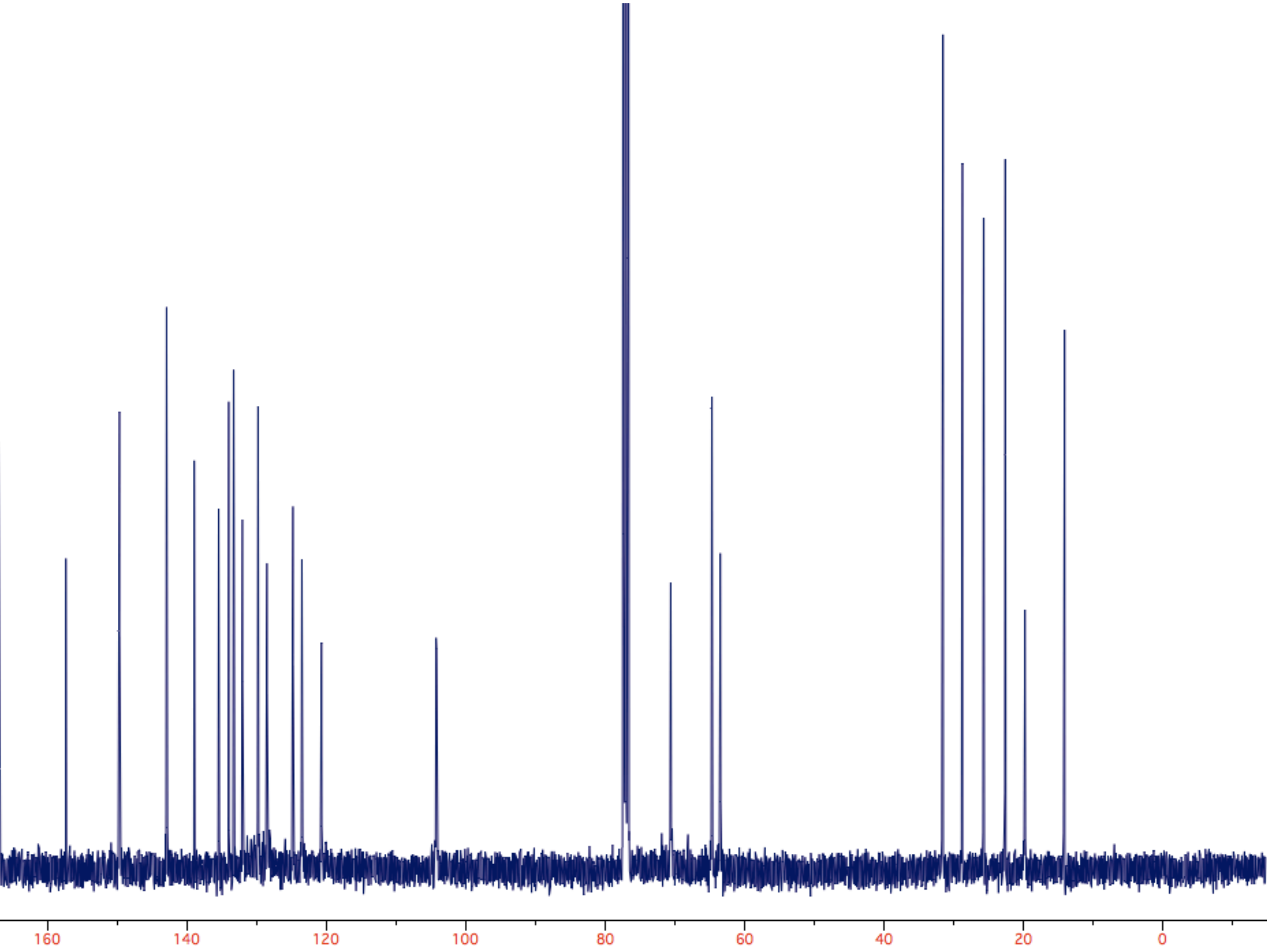

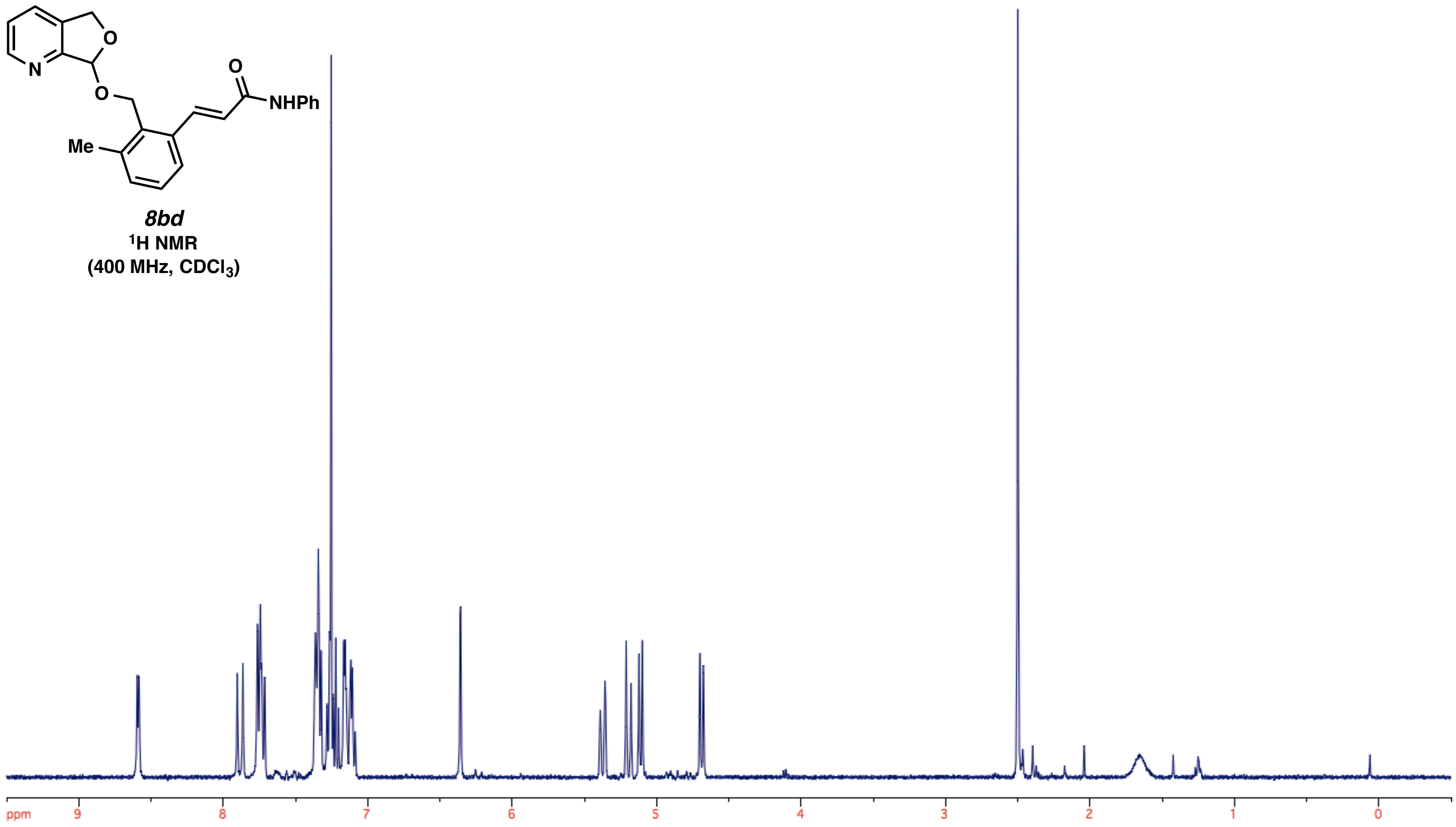
O
N
O
O
NHPh
Me
8bd
^{1}H NMR
(400 MHz, $CDCl_3$)
ppm
9
8
7
6
5
4
3
2
1
0

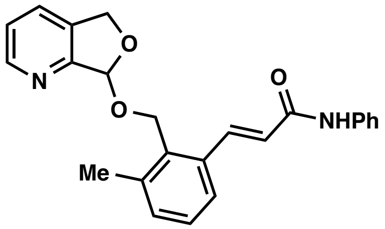

8bd
^{13}C NMR
(100 MHz, $CDCl_3$)

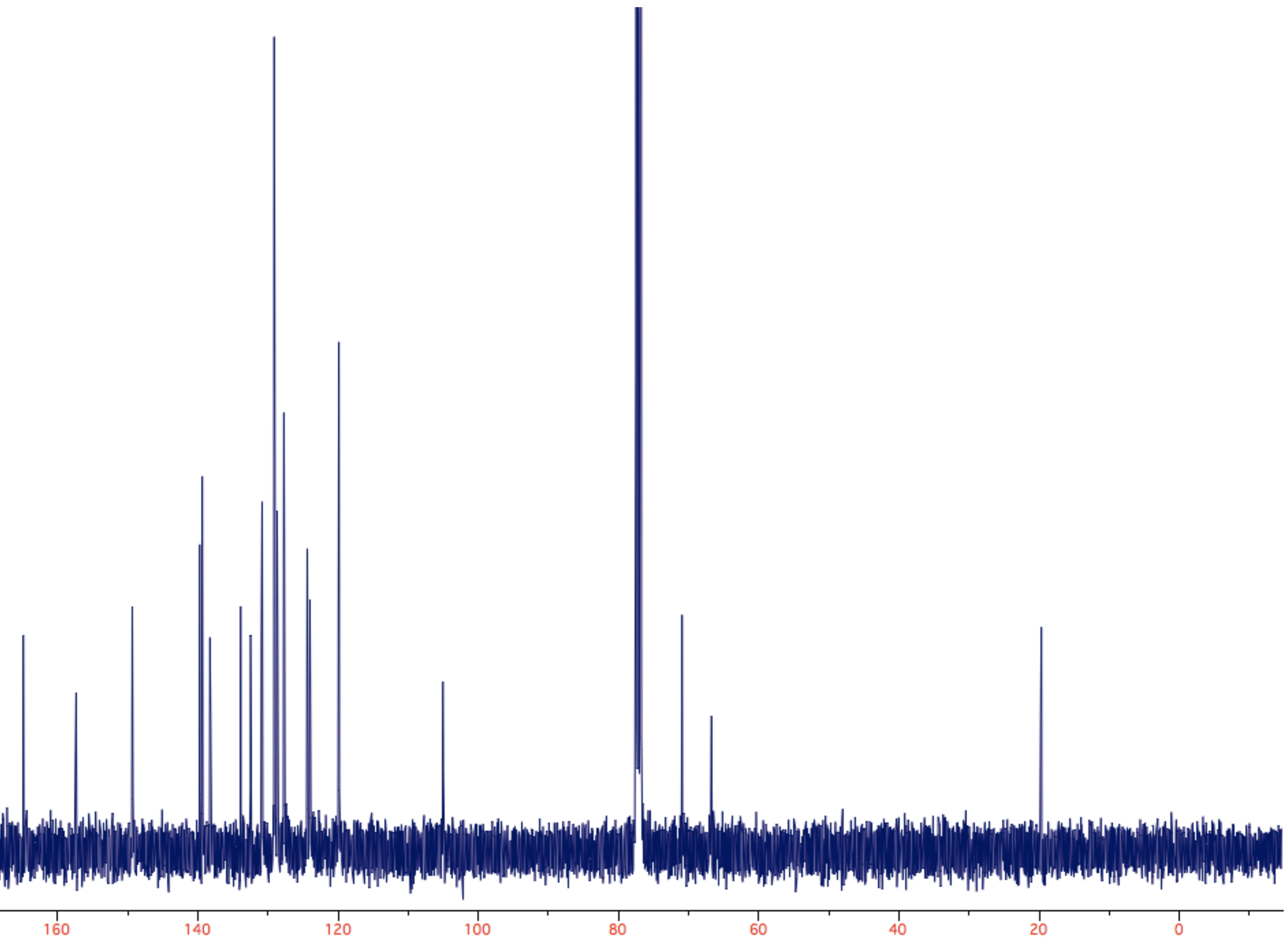

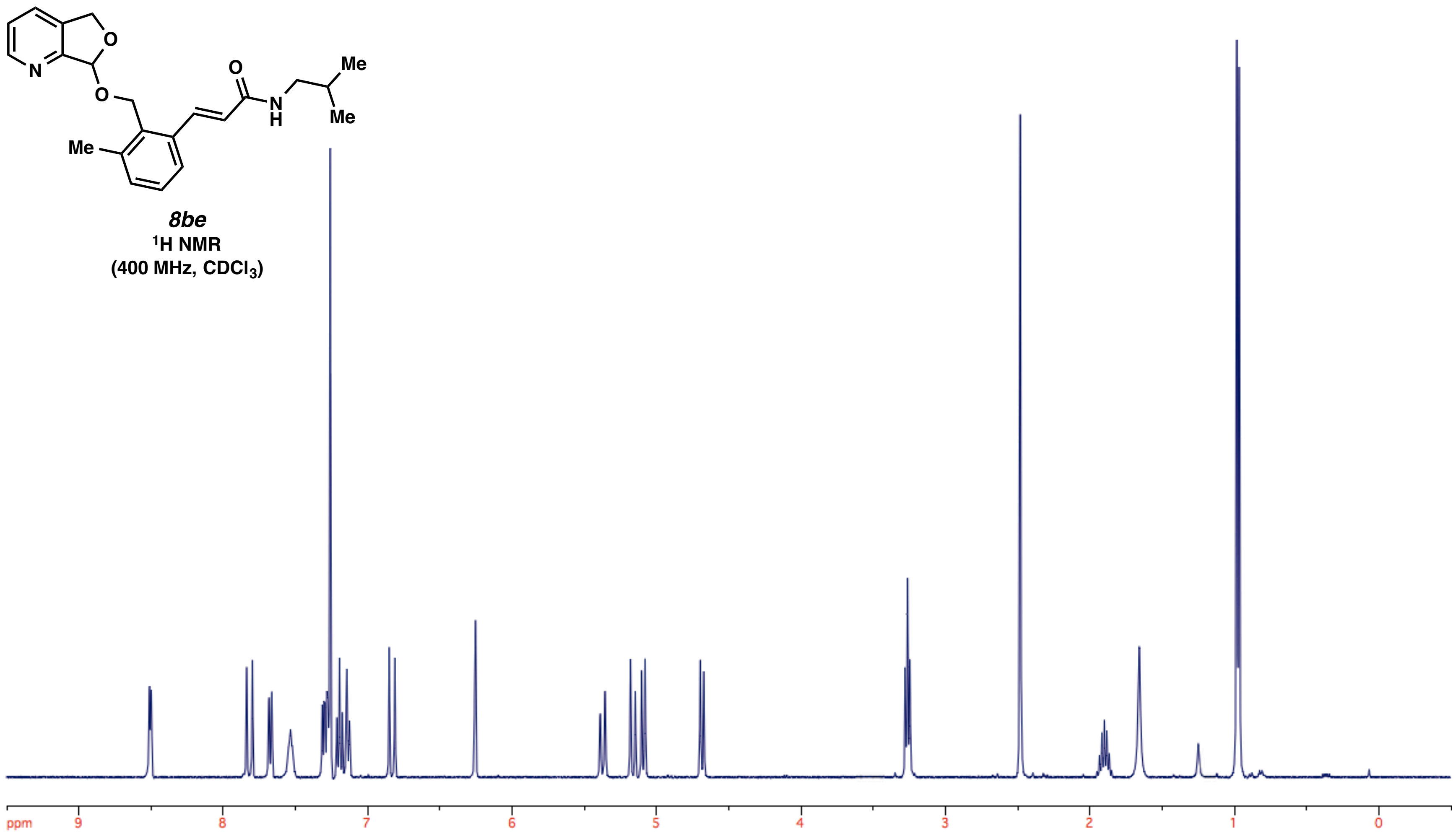

Me
O
N
H
Me
O
O
N
Me
8be
¹H NMR
(400 MHz, CDCl3)
ppm
9
8
7
6
5
4
3
2
1
0

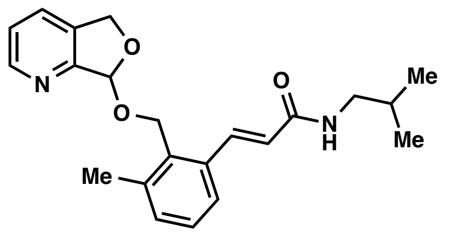

8be
^{13}C NMR
(100 MHz, $CDCl_3$)

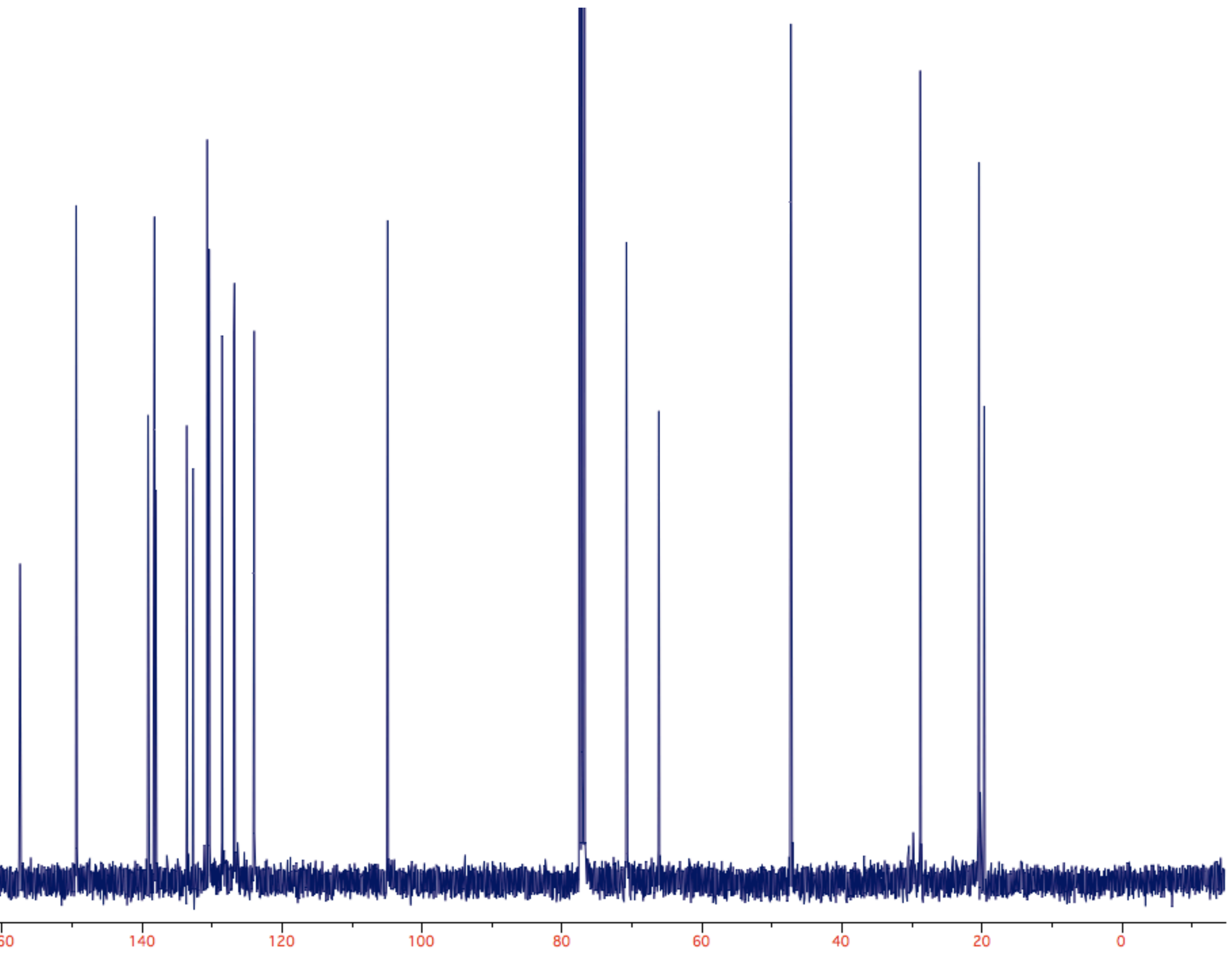

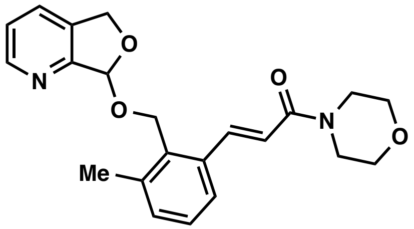

8bf
^{1}H NMR
(400 MHz, $CDCl_3$)

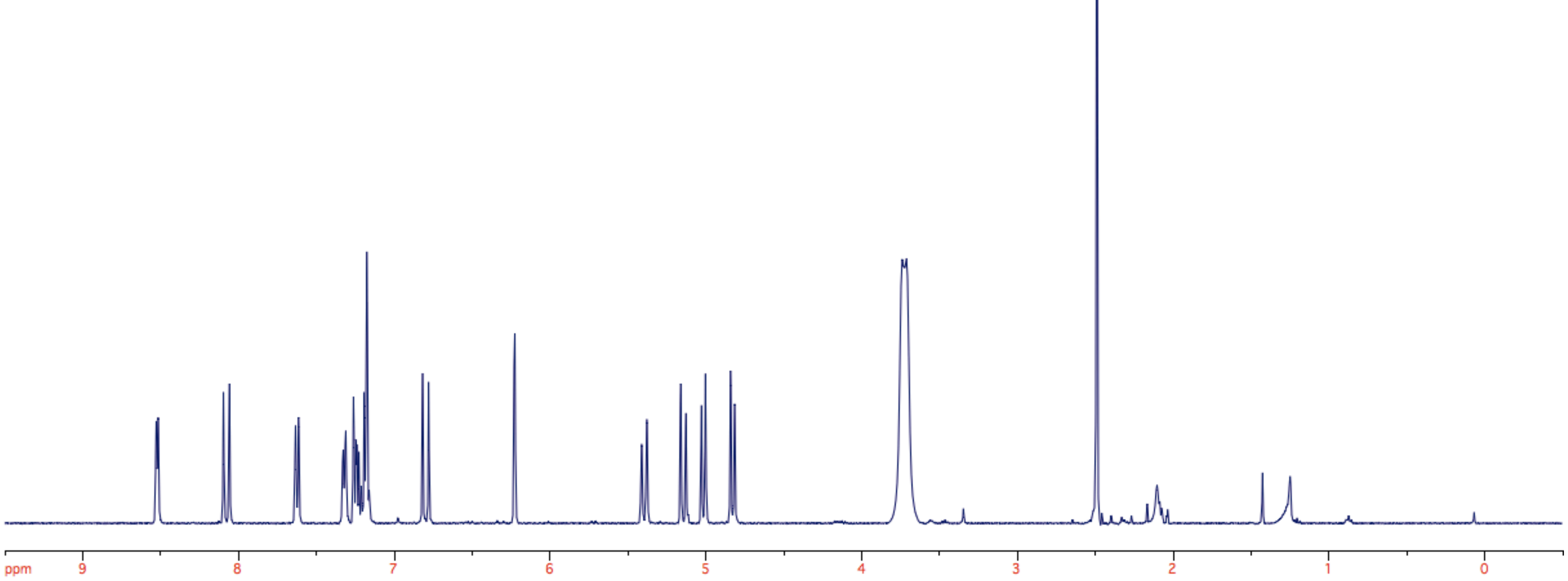

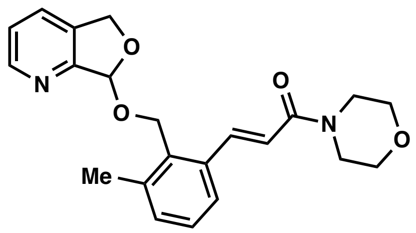

8bf
^{13}C NMR
(100 MHz, $CDCl_3$)

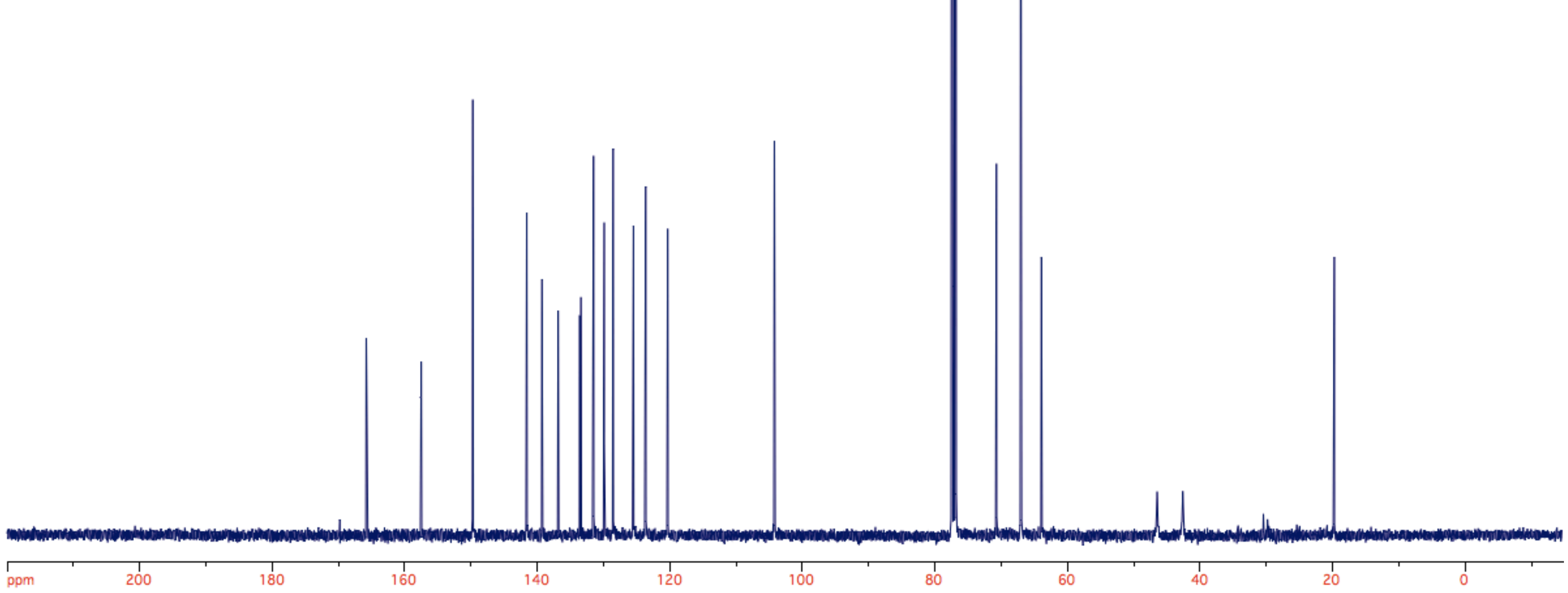

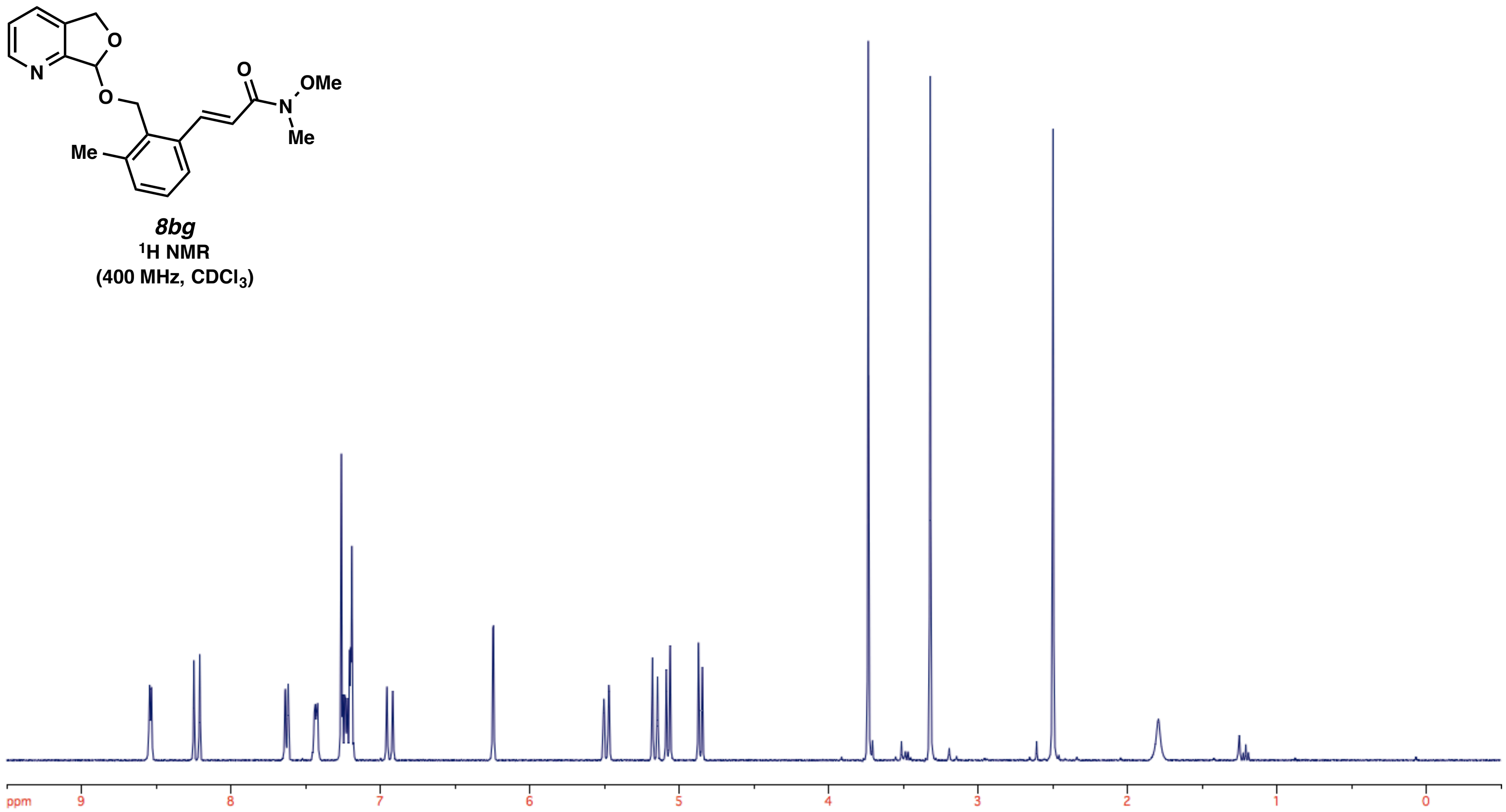

8bg
^{1}H NMR
(400 MHz, $CDCl_3$)

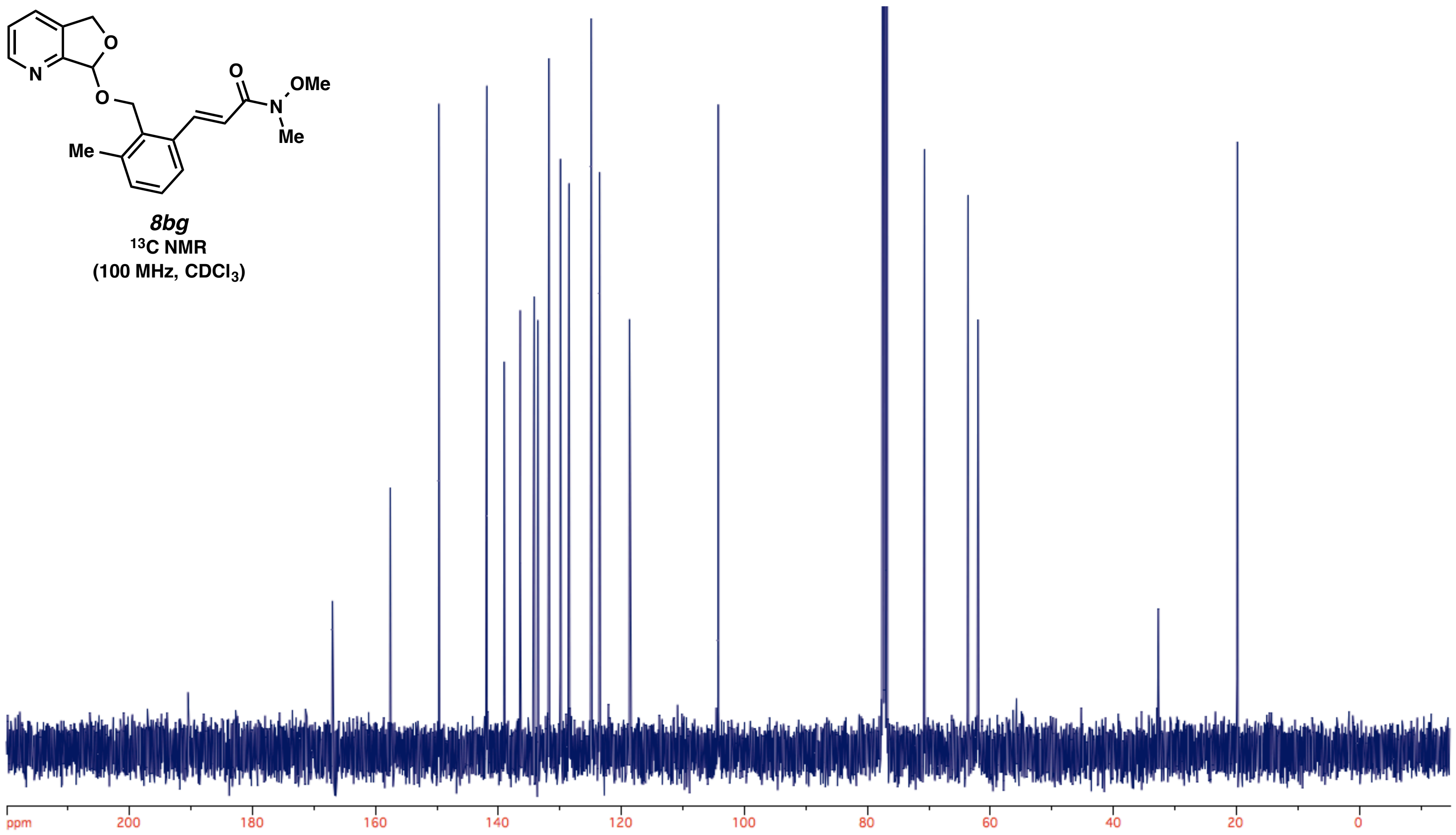

8bg
^{13}C NMR
(100 MHz, $CDCl_3$)

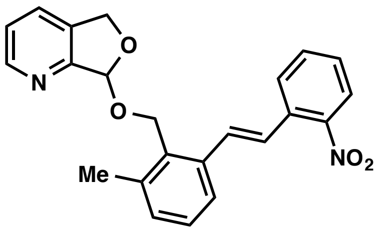

8bh
^{1}H NMR
(400 MHz, $CDCl_3$)

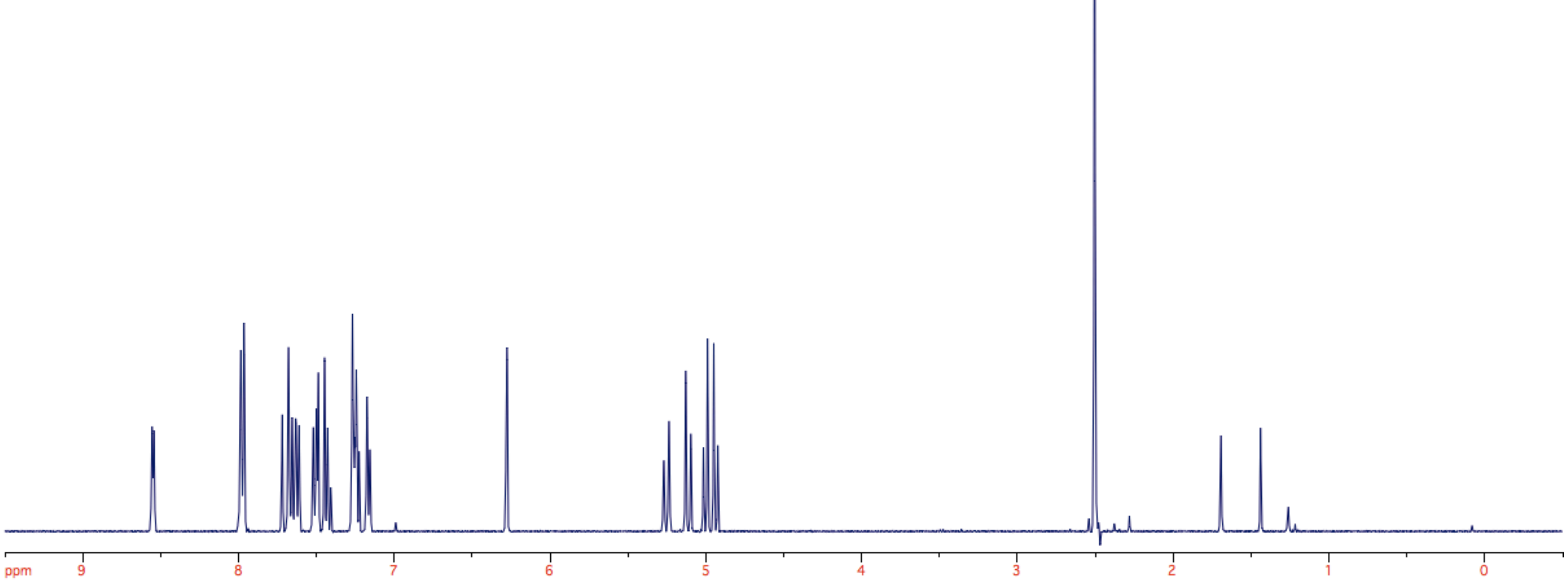

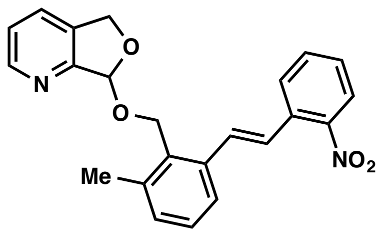

8bh
^{13}C NMR
(100 MHz, $CDCl_3$)

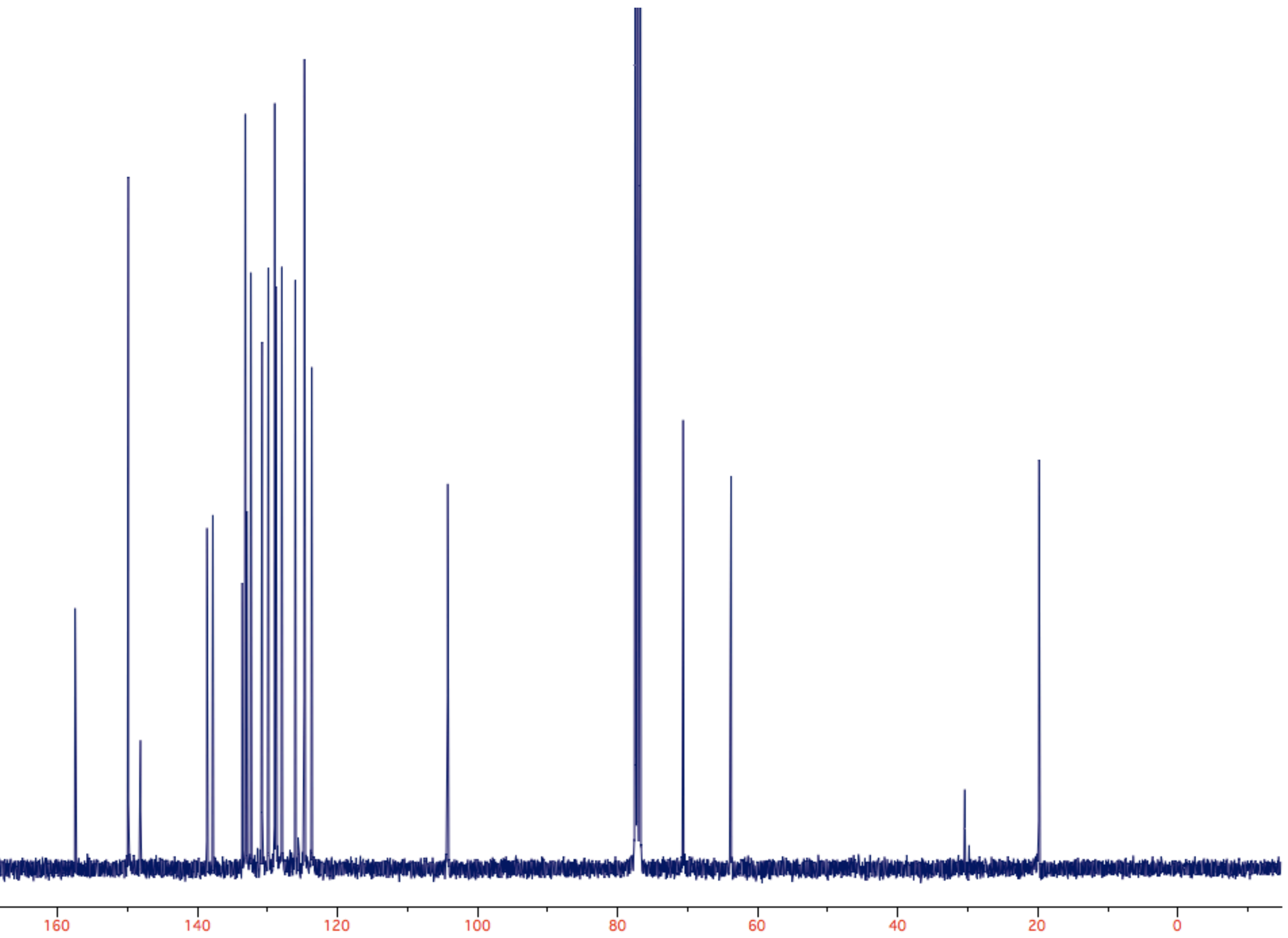

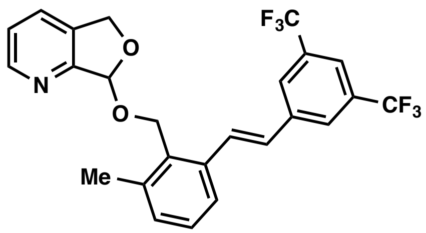

8bi
^{1}H NMR
(400 MHz, $CDCl_3$)

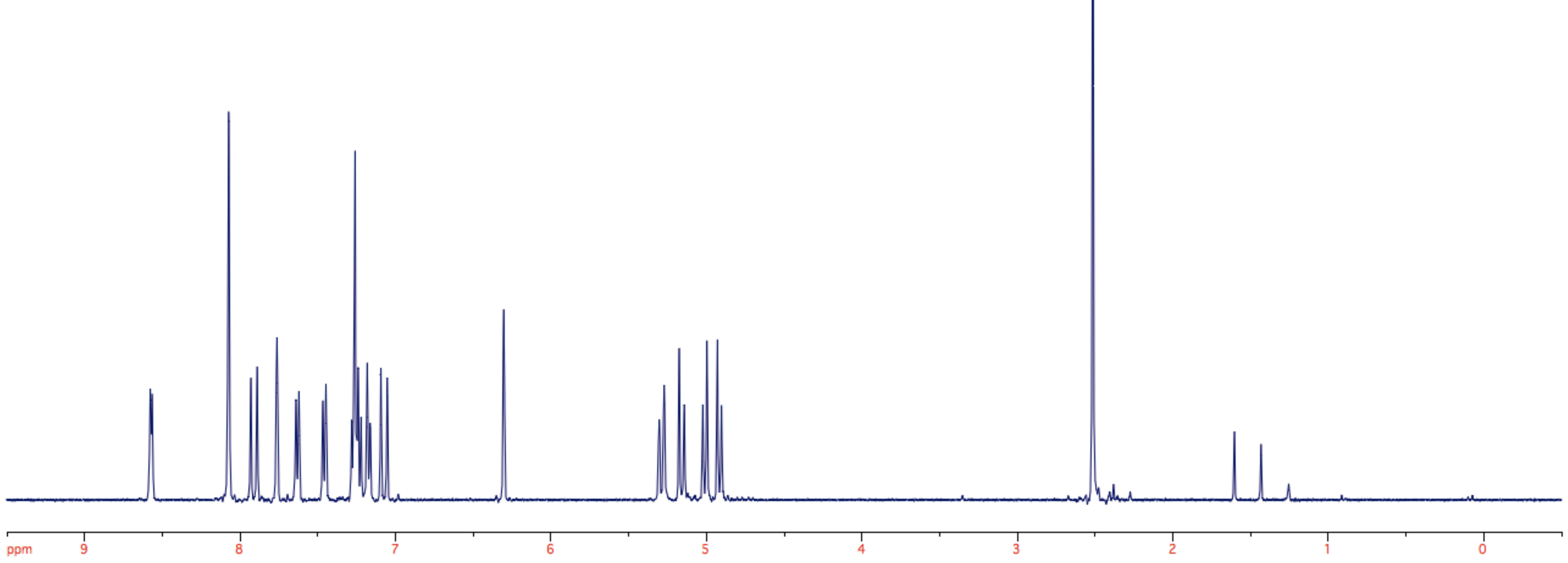

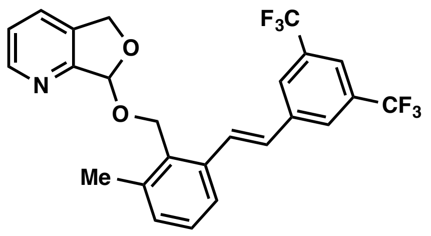

8bi
^{13}C NMR
(100 MHz, $CDCl_3$)

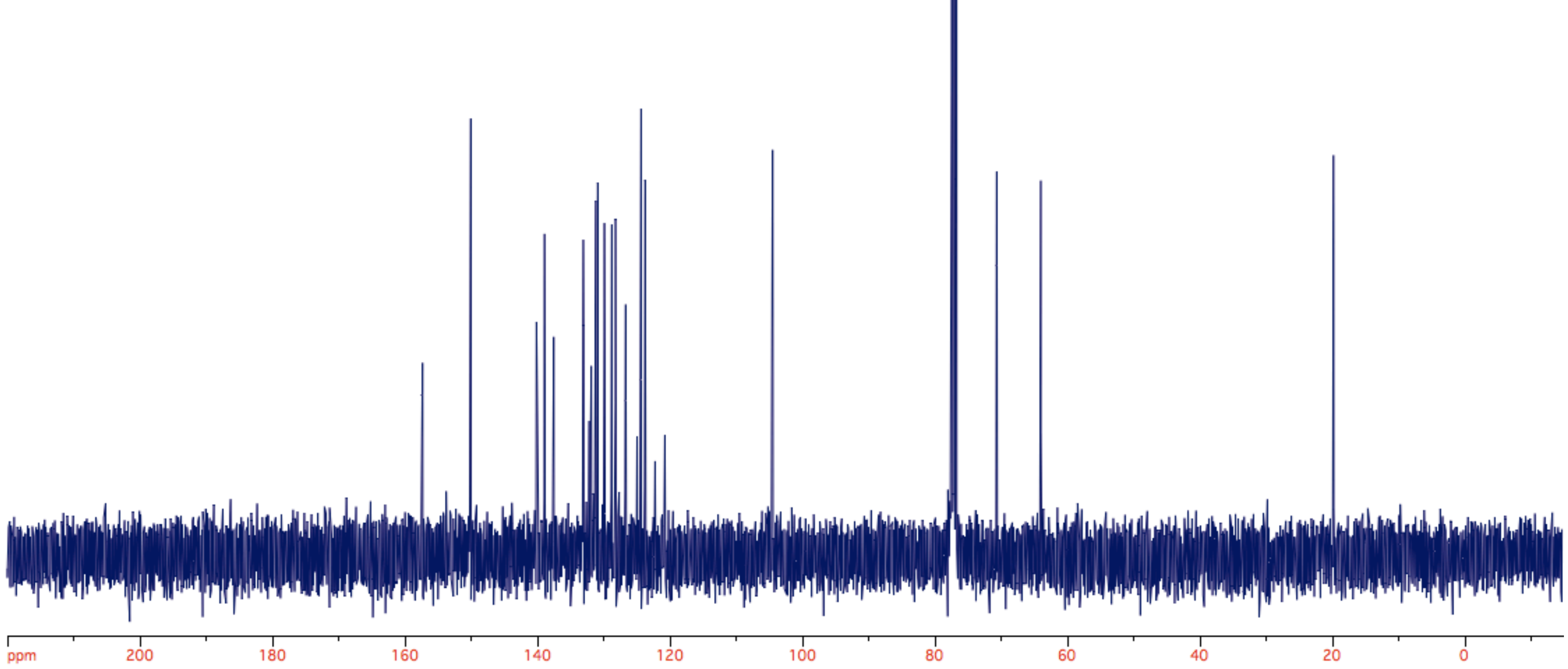

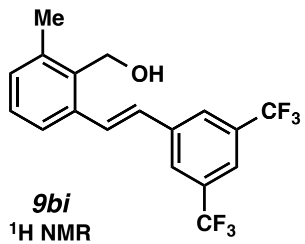

9bi
^{1}H NMR
(400 MHz, $CDCl_3$)

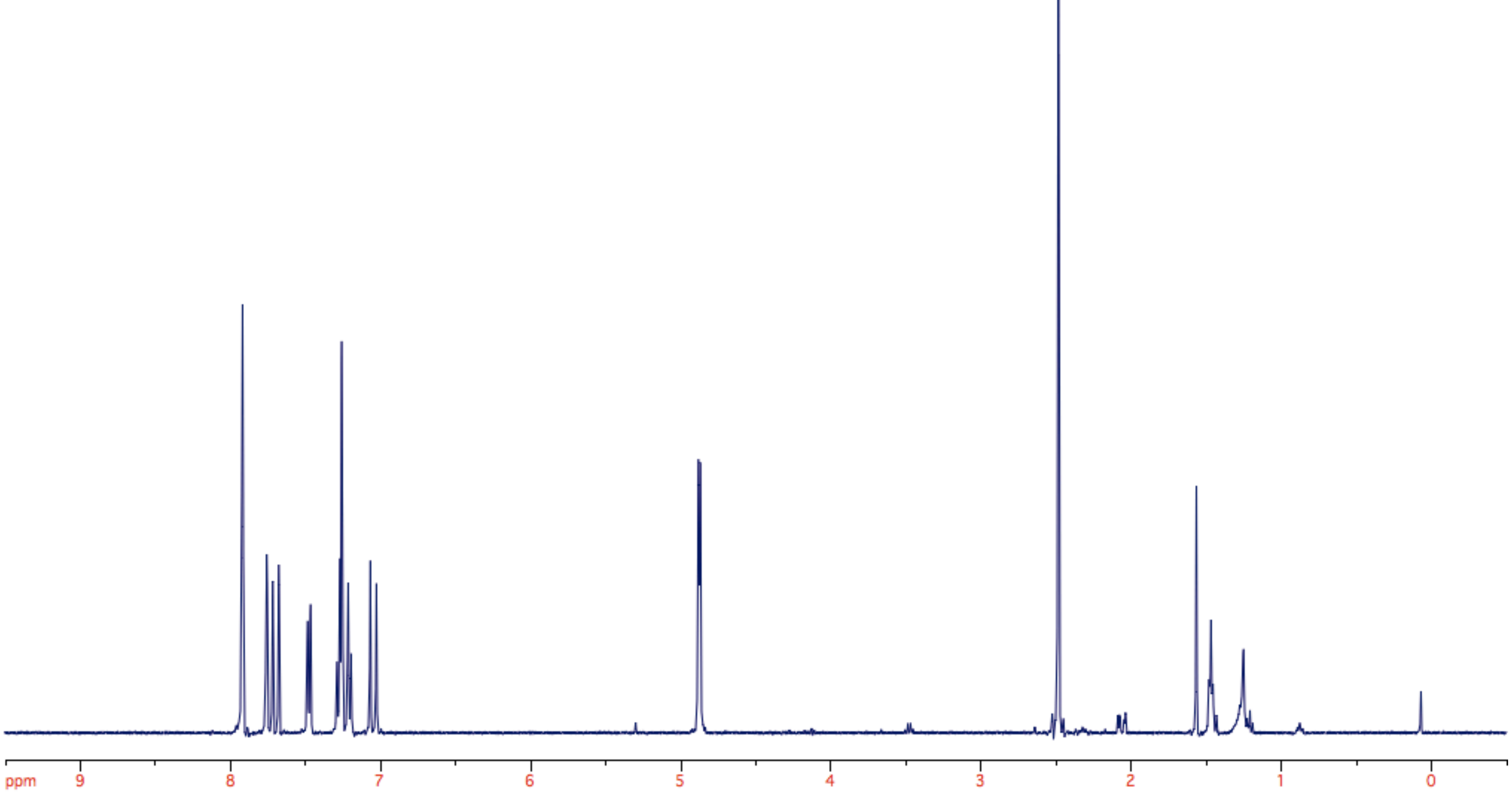

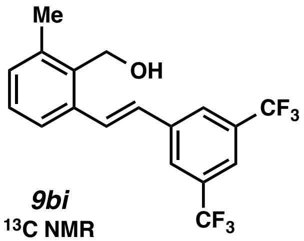

9bi
^{13}C NMR
(100 MHz, $CDCl_3$)

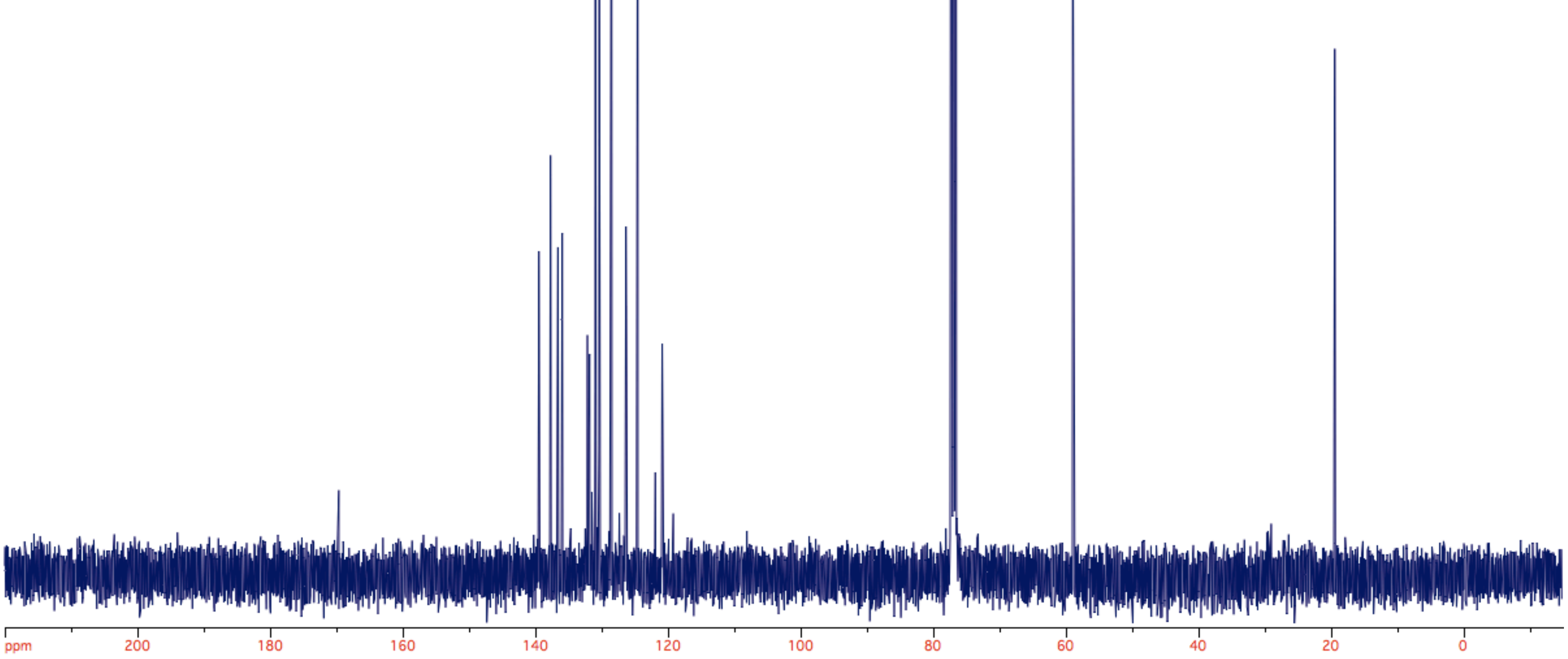

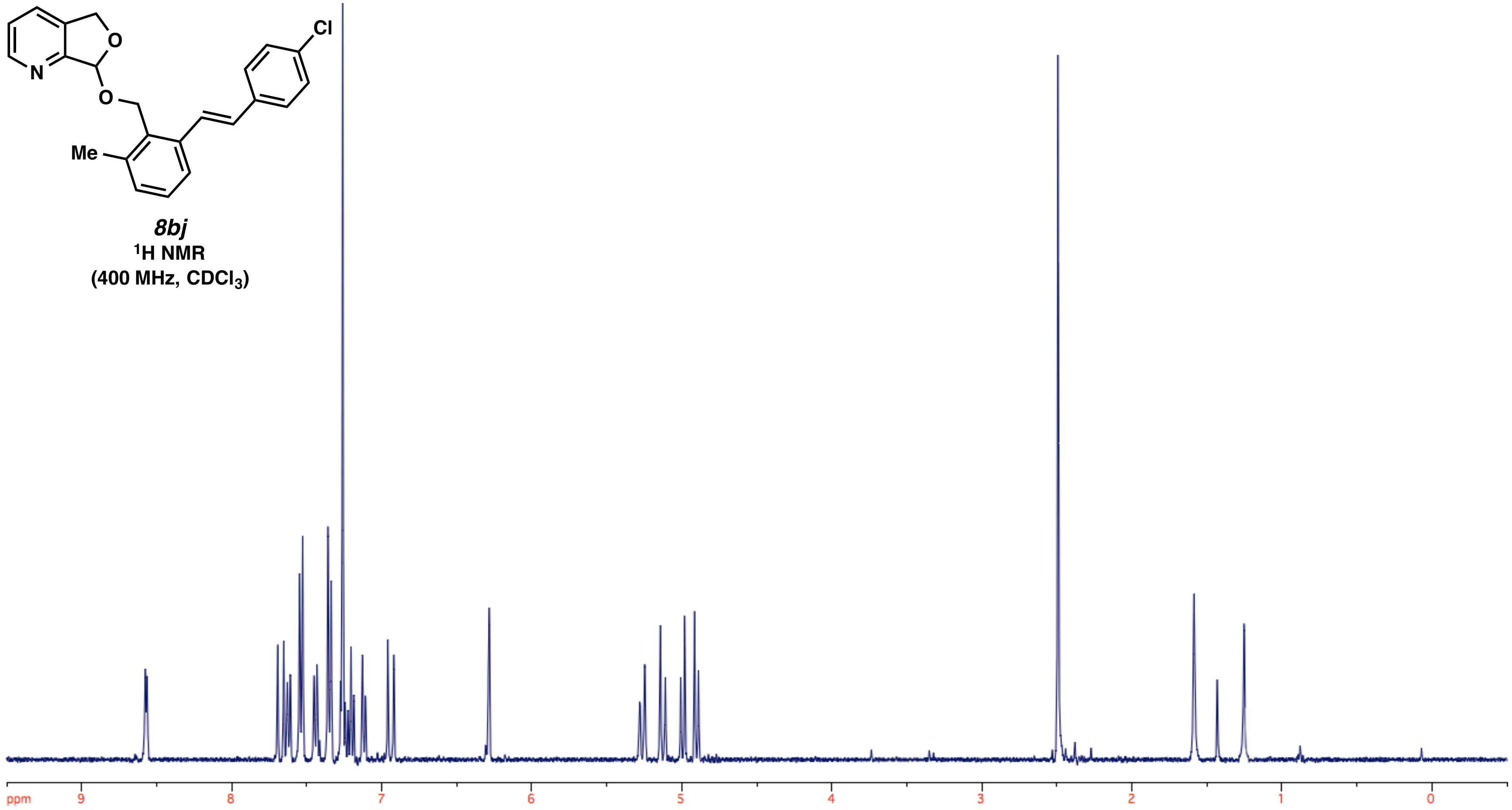

8bj
^{1}H NMR
(400 MHz, $CDCl_3$)

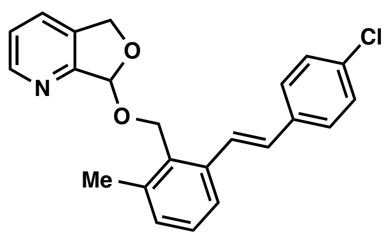

8bj
^{13}C NMR
(100 MHz, $CDCl_3$)

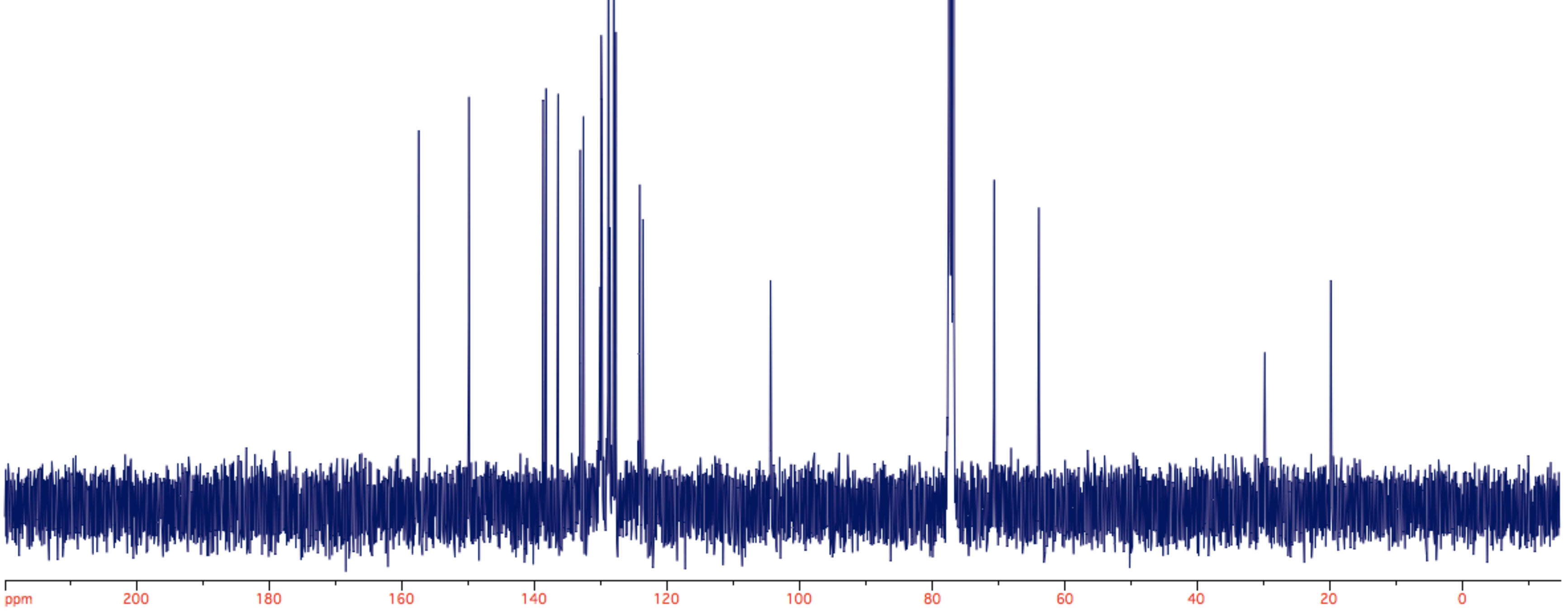

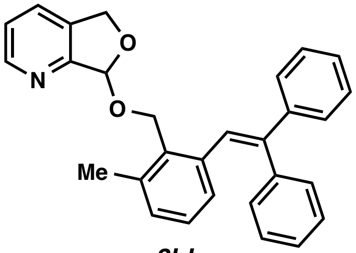

8bk
^{1}H NMR
(400 MHz, $CDCl_3$)

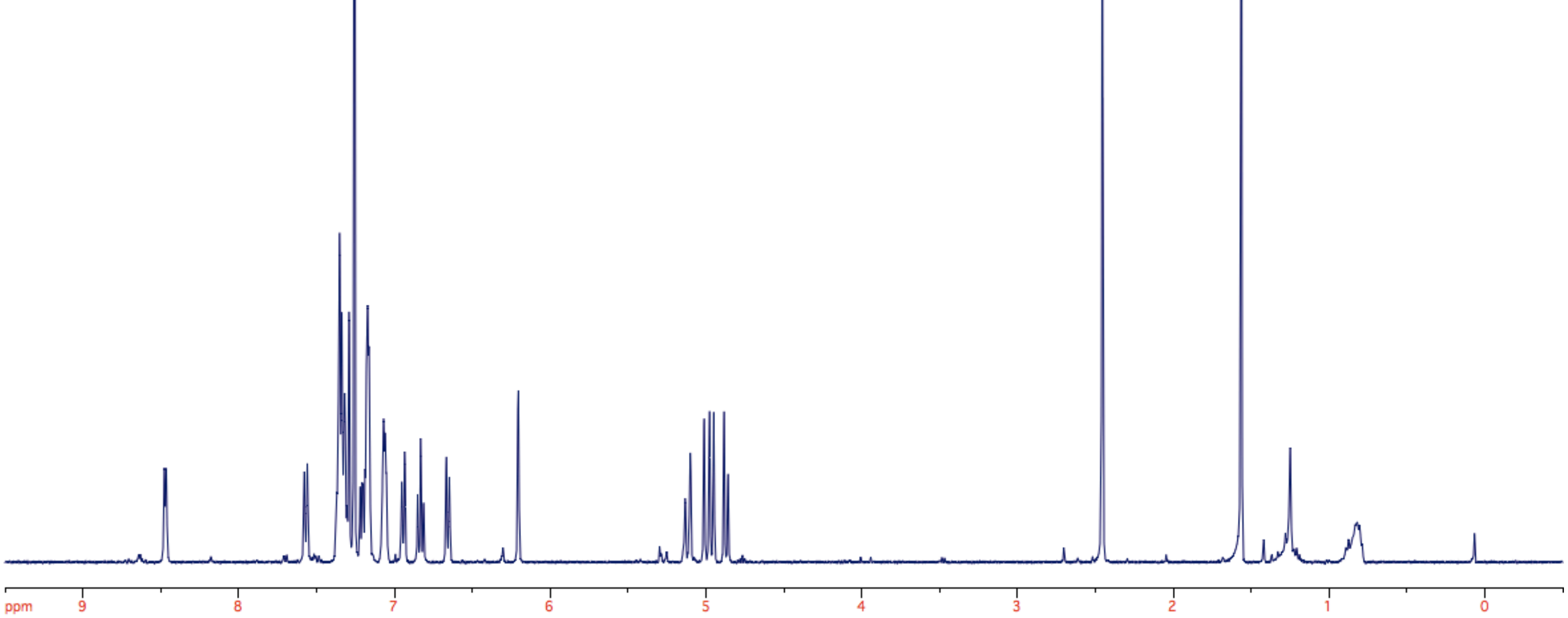

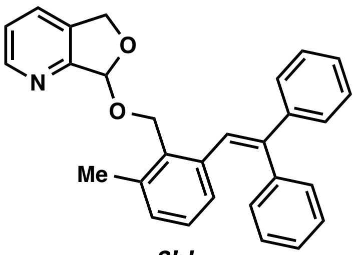

8bk
^{13}C NMR
(100 MHz, $CDCl_3$)

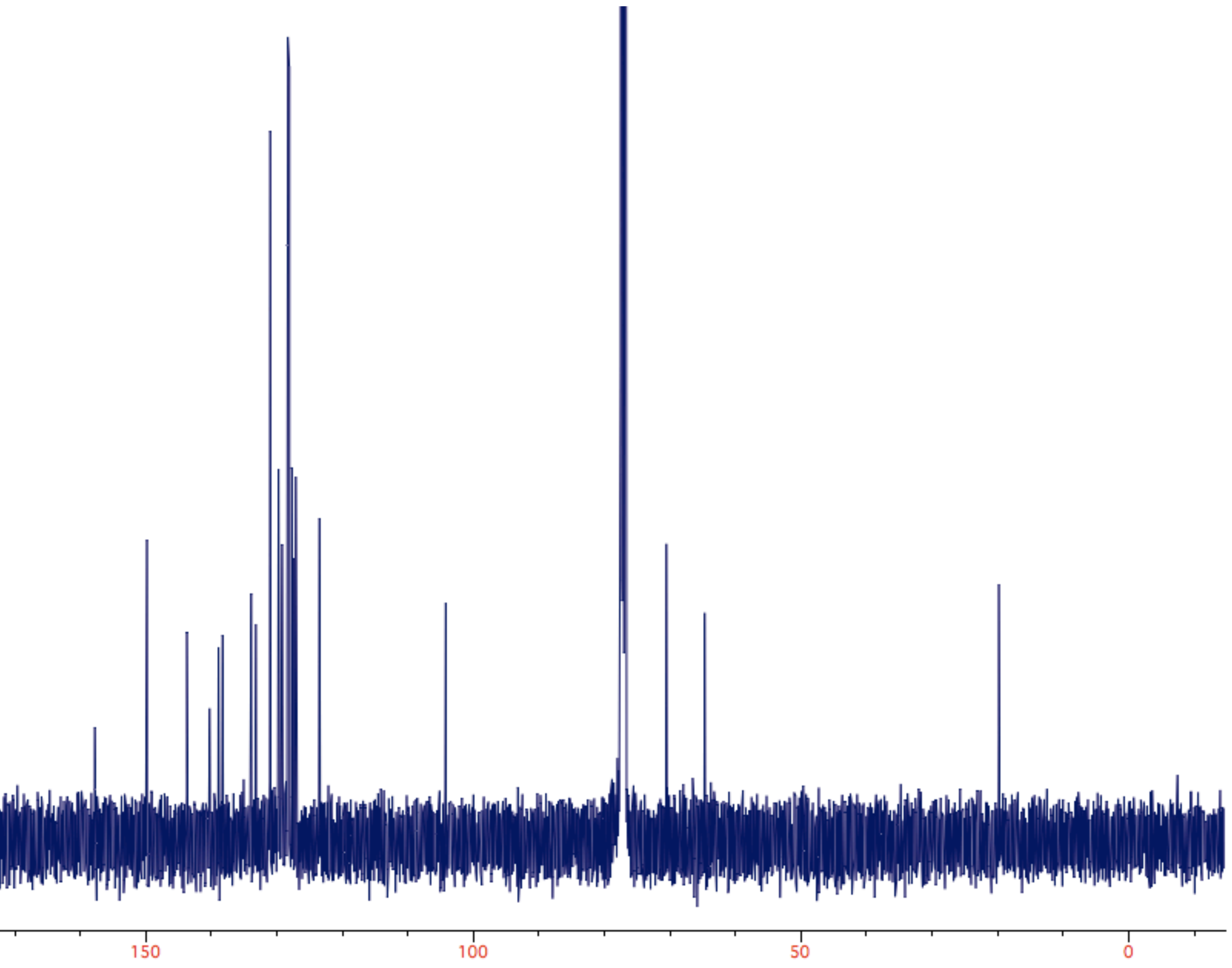

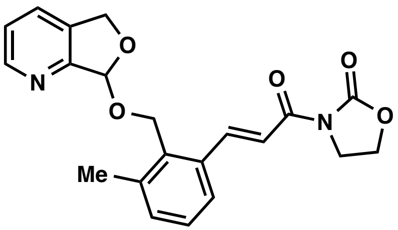

8bl
^{1}H NMR
(400 MHz, $CDCl_3$)

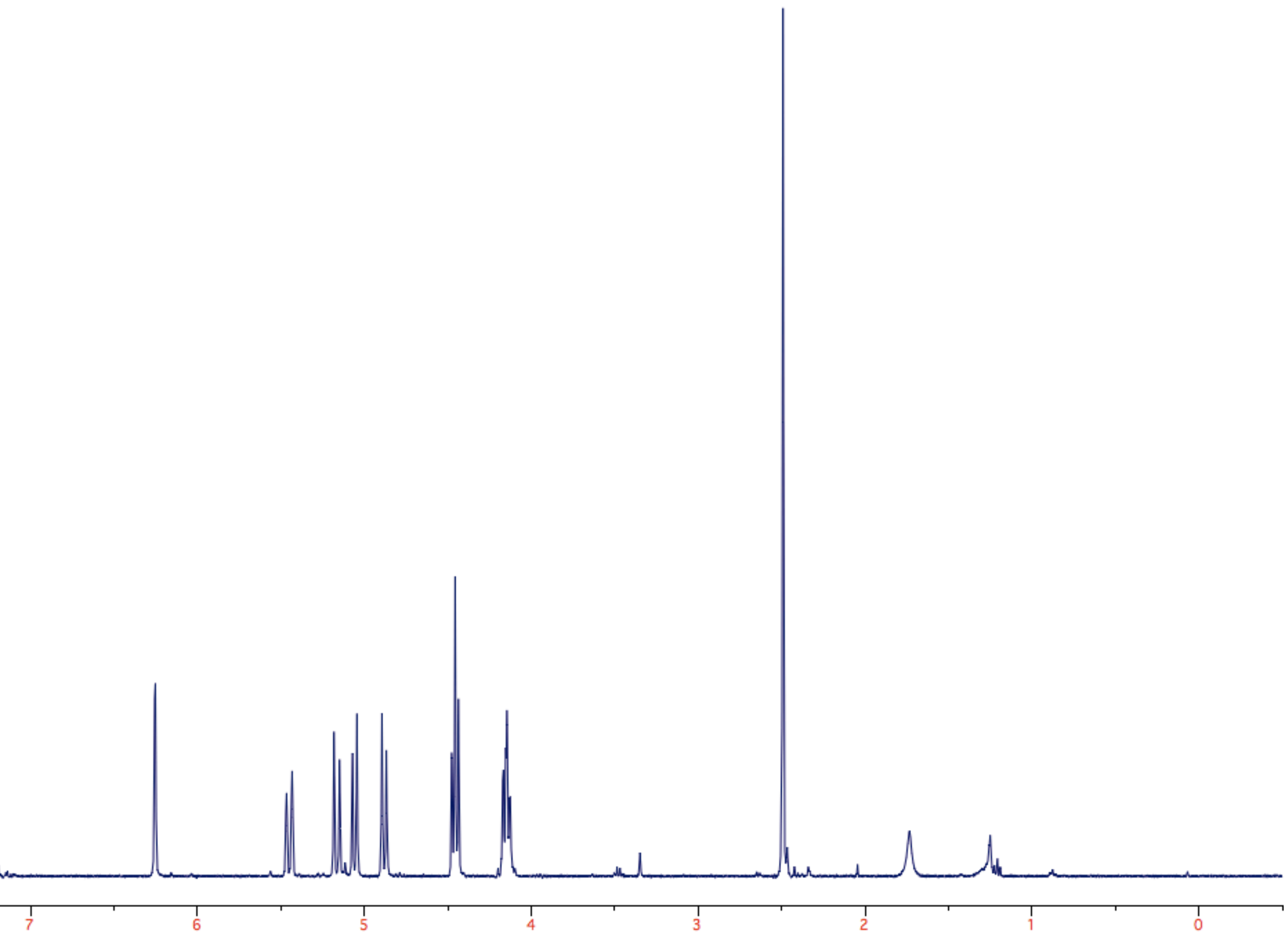

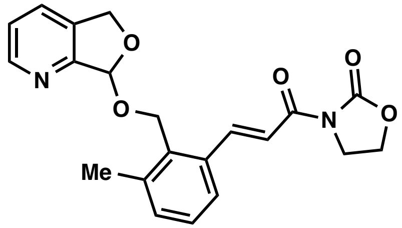

8bl
^{13}C NMR
(100 MHz, $CDCl_3$)

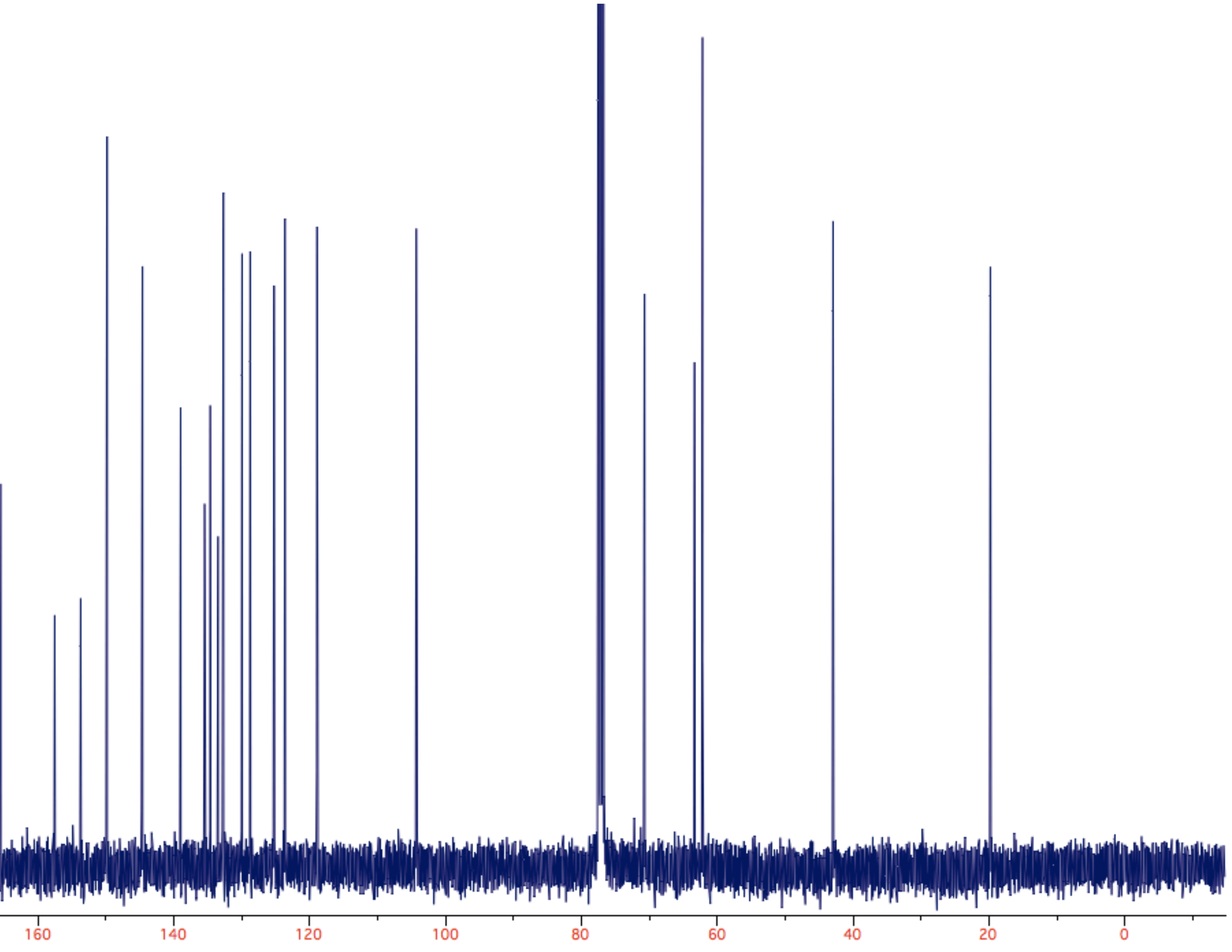

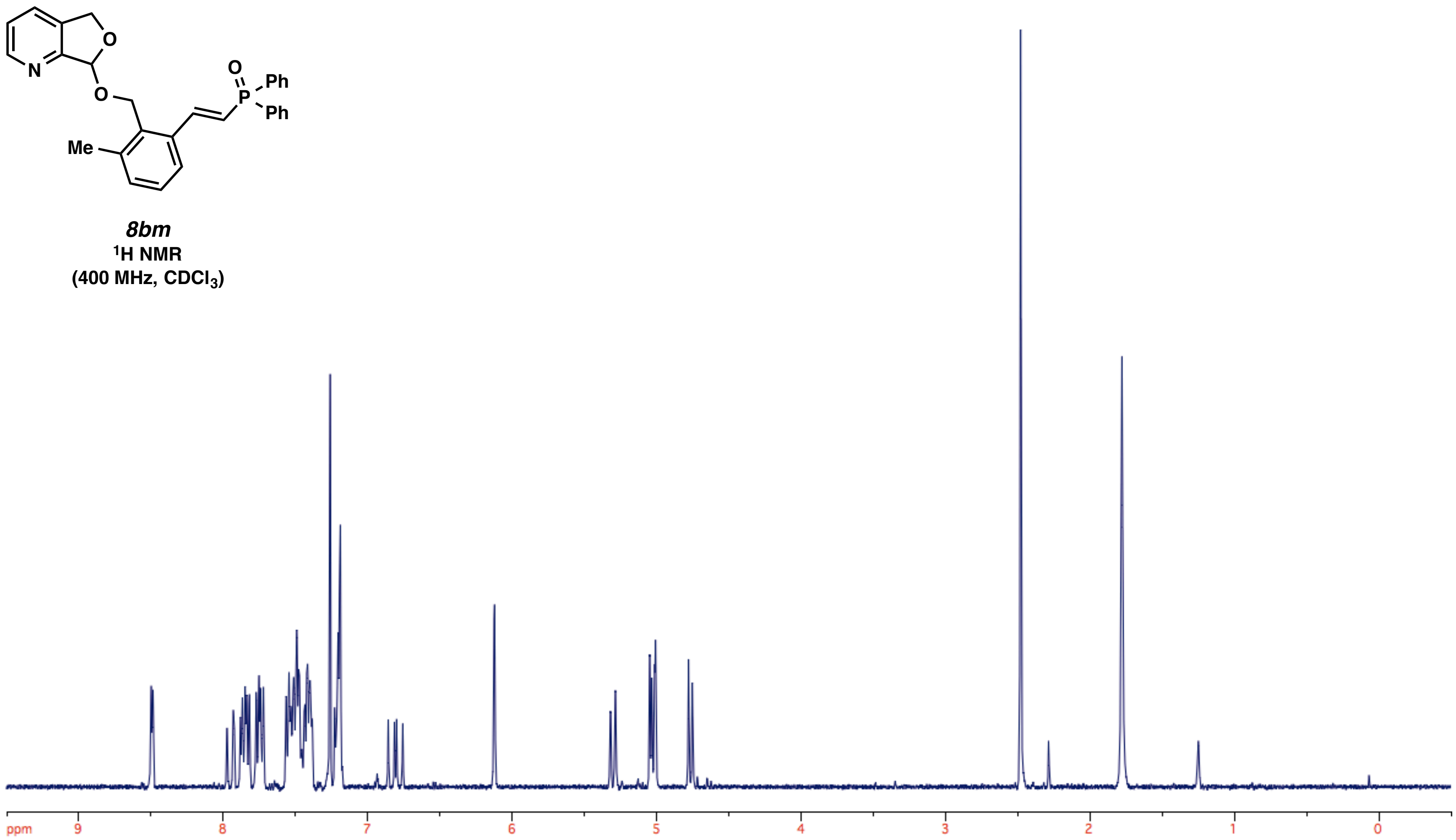

8bm
^{1}H NMR
(400 MHz, $CDCl_3$)

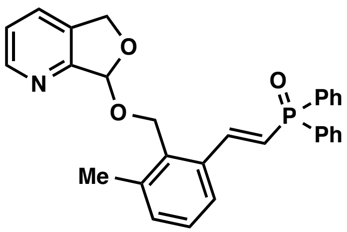

8bm
^{13}C NMR
(100 MHz, $CDCl_3$)

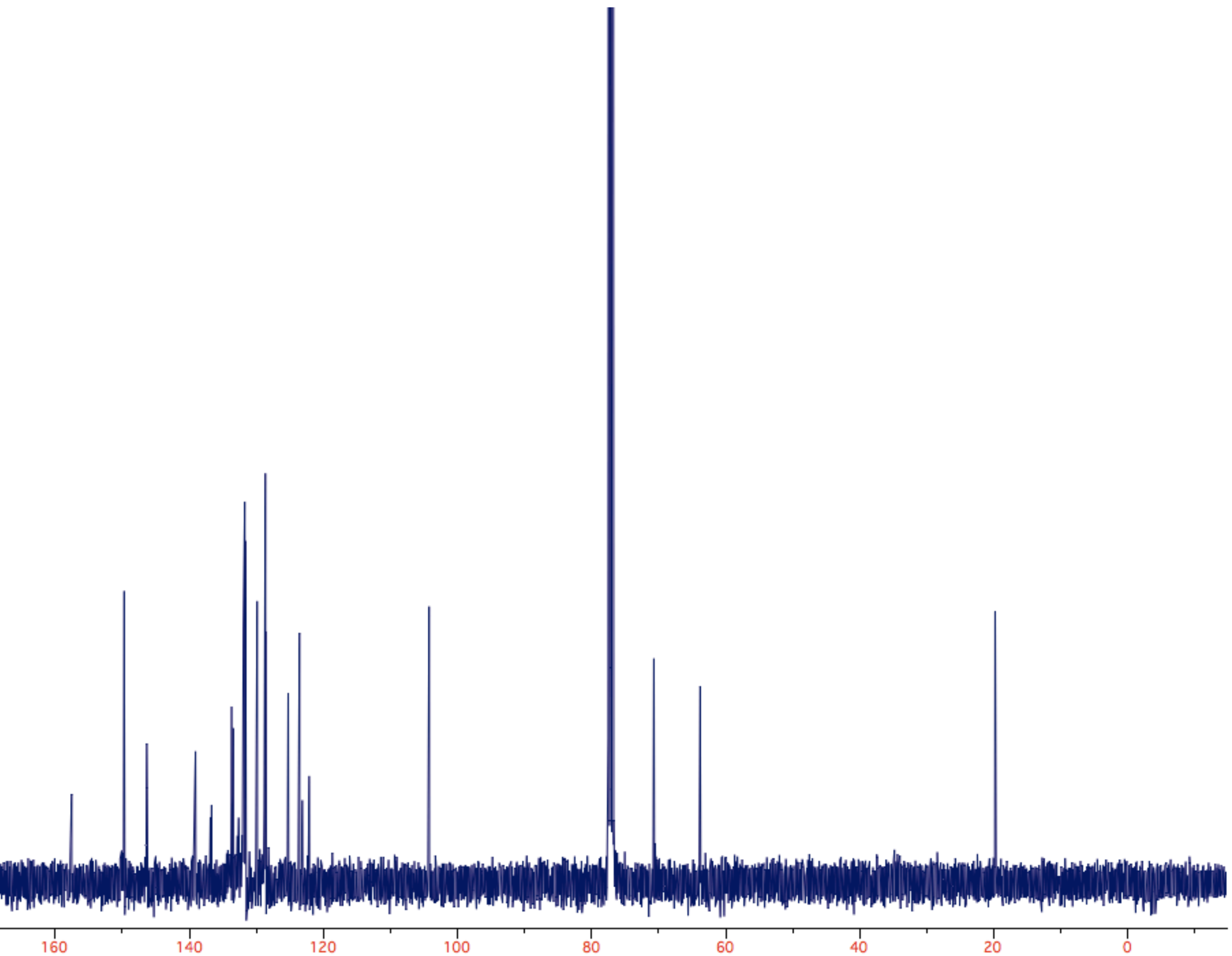

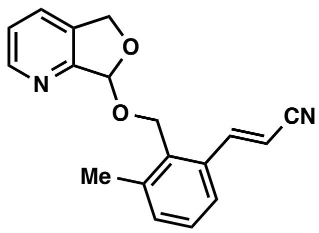

8bn
^{1}H NMR
(400 MHz, $CDCl_3$)

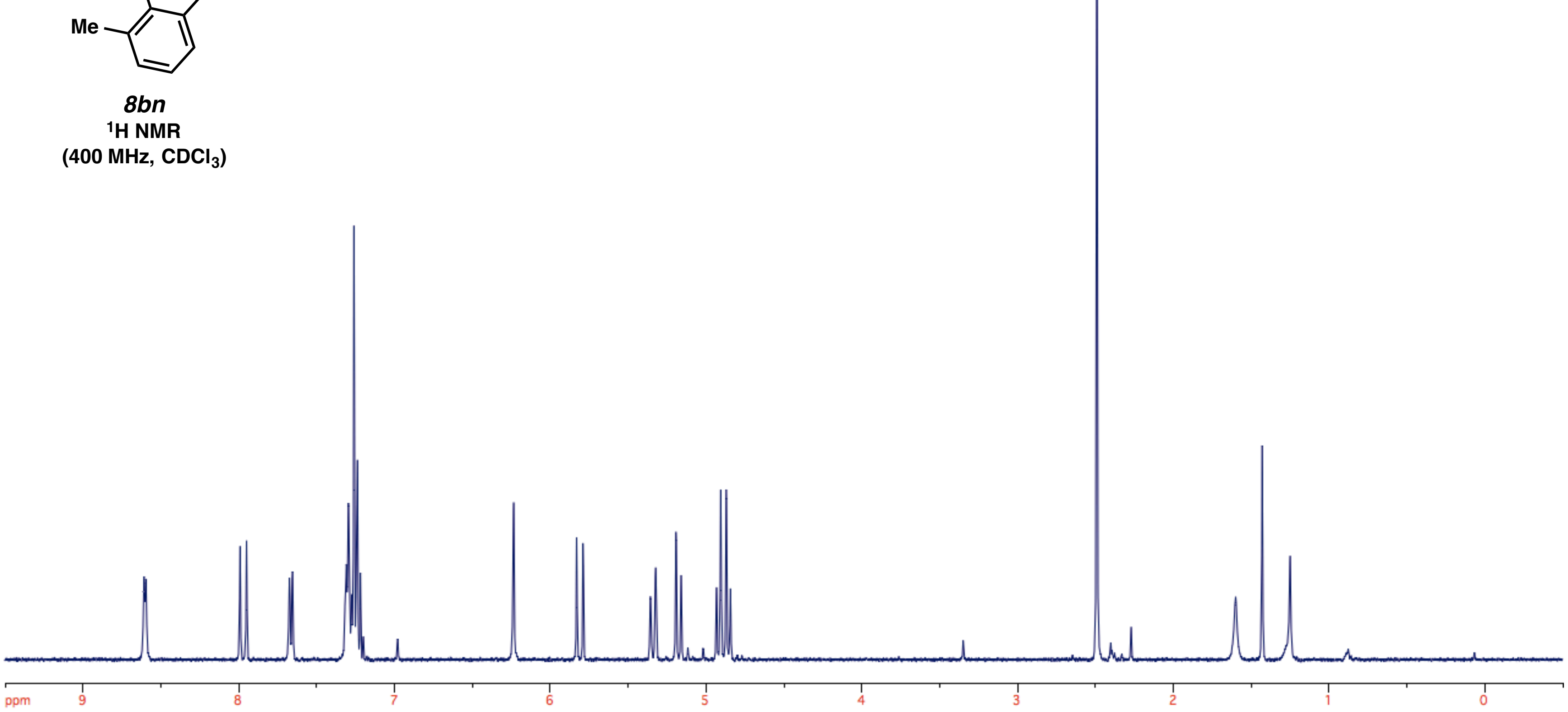

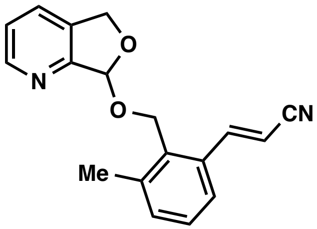

8bn
^{13}C NMR
(100 MHz, $CDCl_3$)

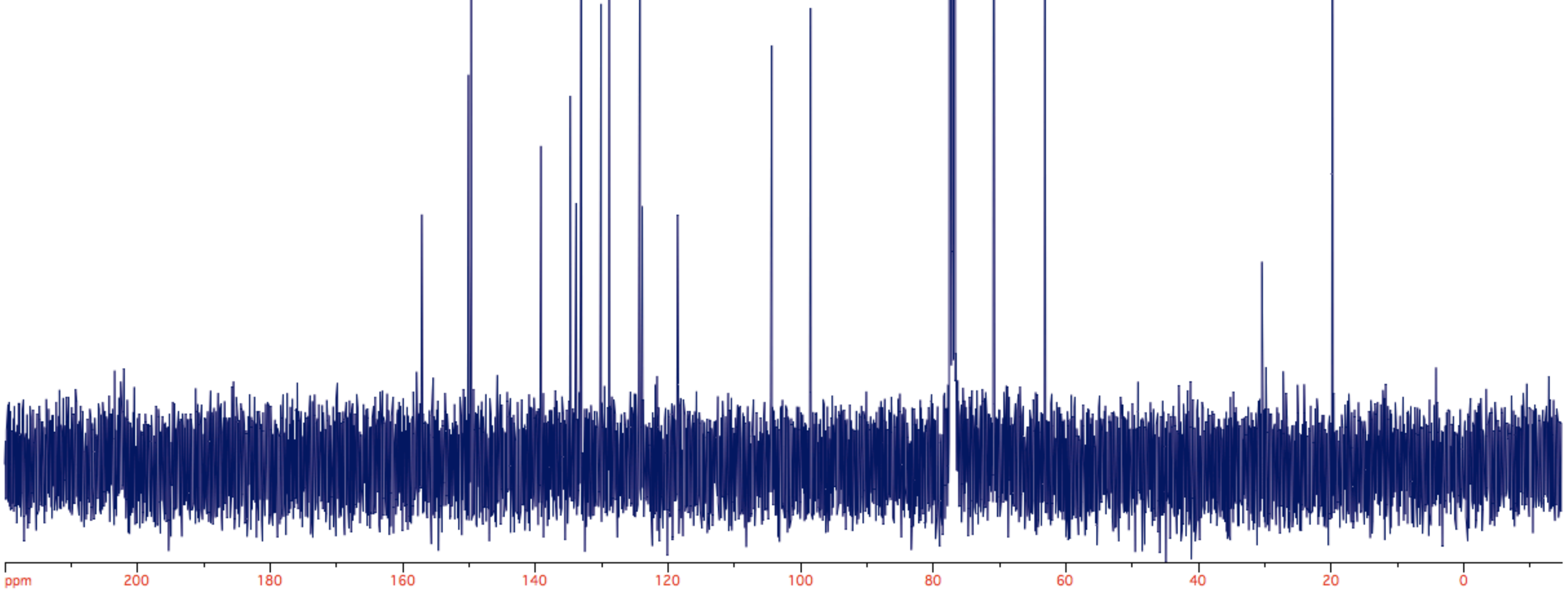

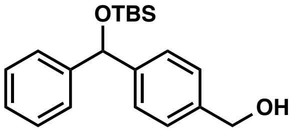

S26
^{1}H NMR
(500 MHz, $CDCl_3$)

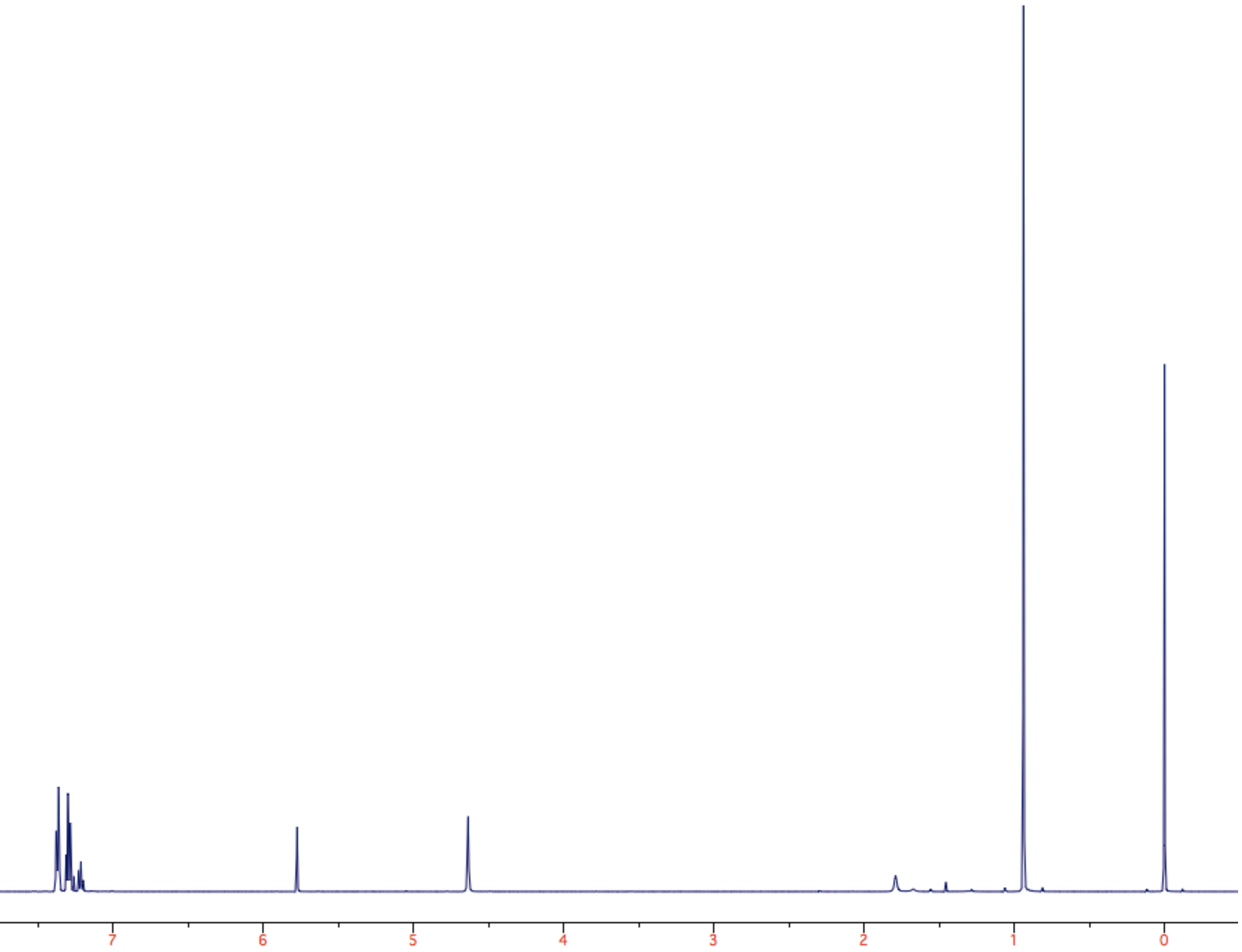

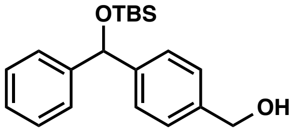

S26
^{13}C NMR
(125 MHz, $CDCl_3$)

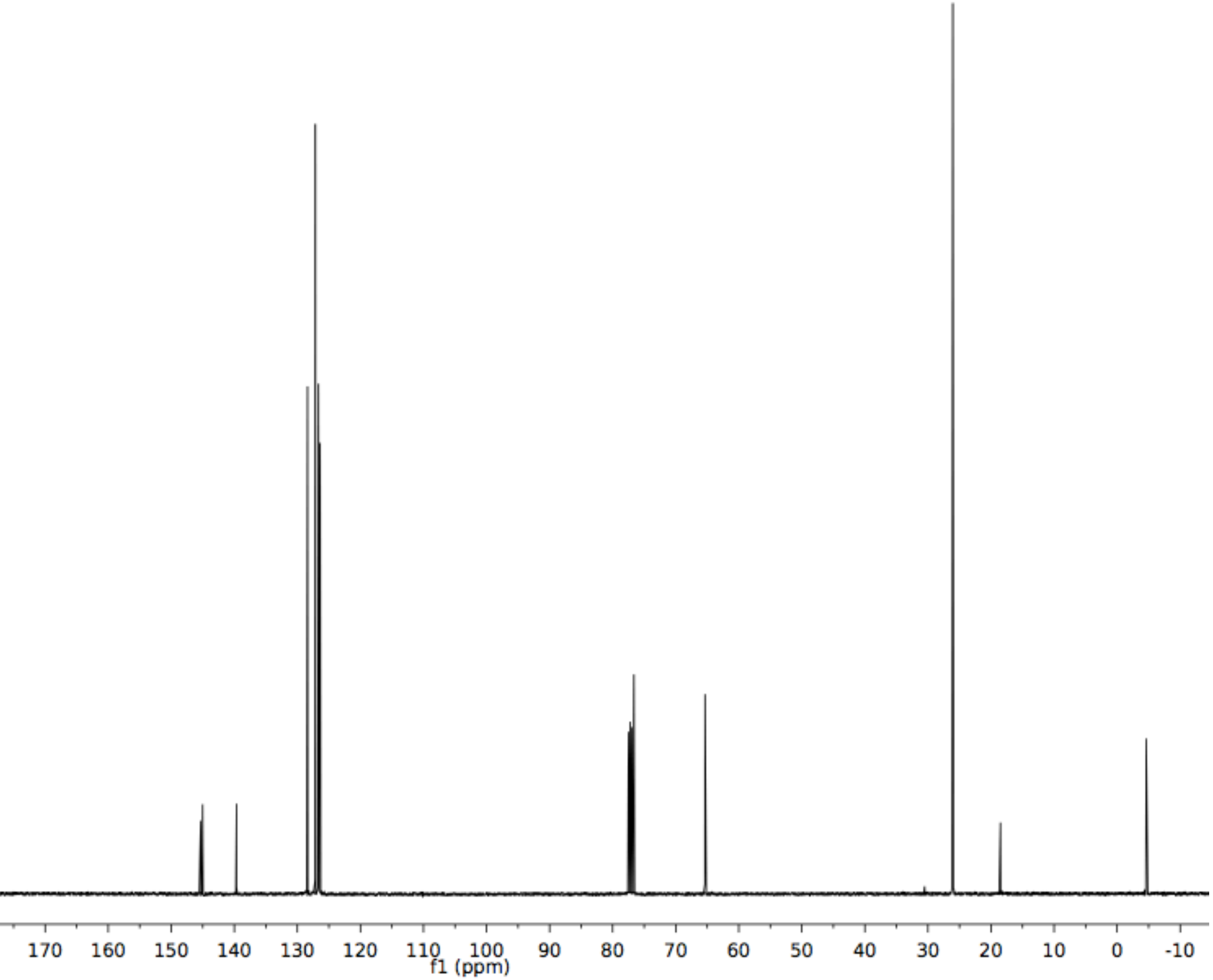

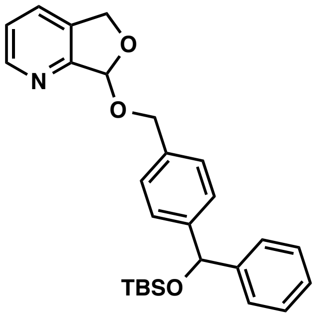

10
^{1}H NMR
(500 MHz, $CDCl_3$)

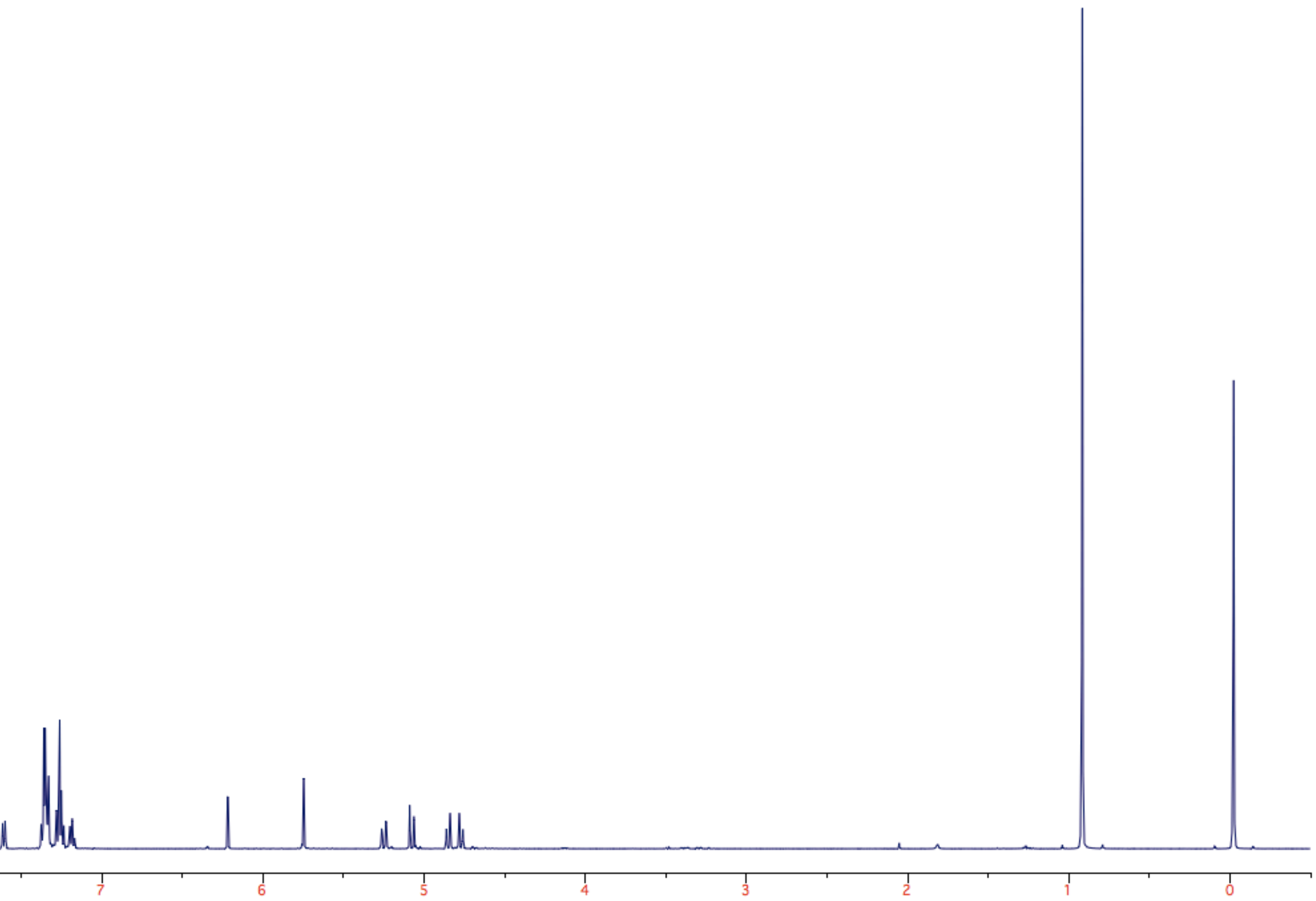

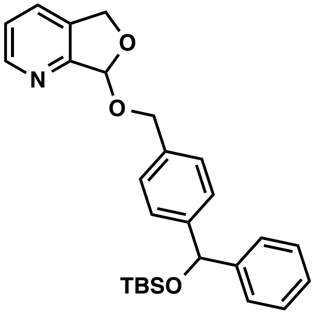

10
^{13}C NMR
(125 MHz, $CDCl_3$)

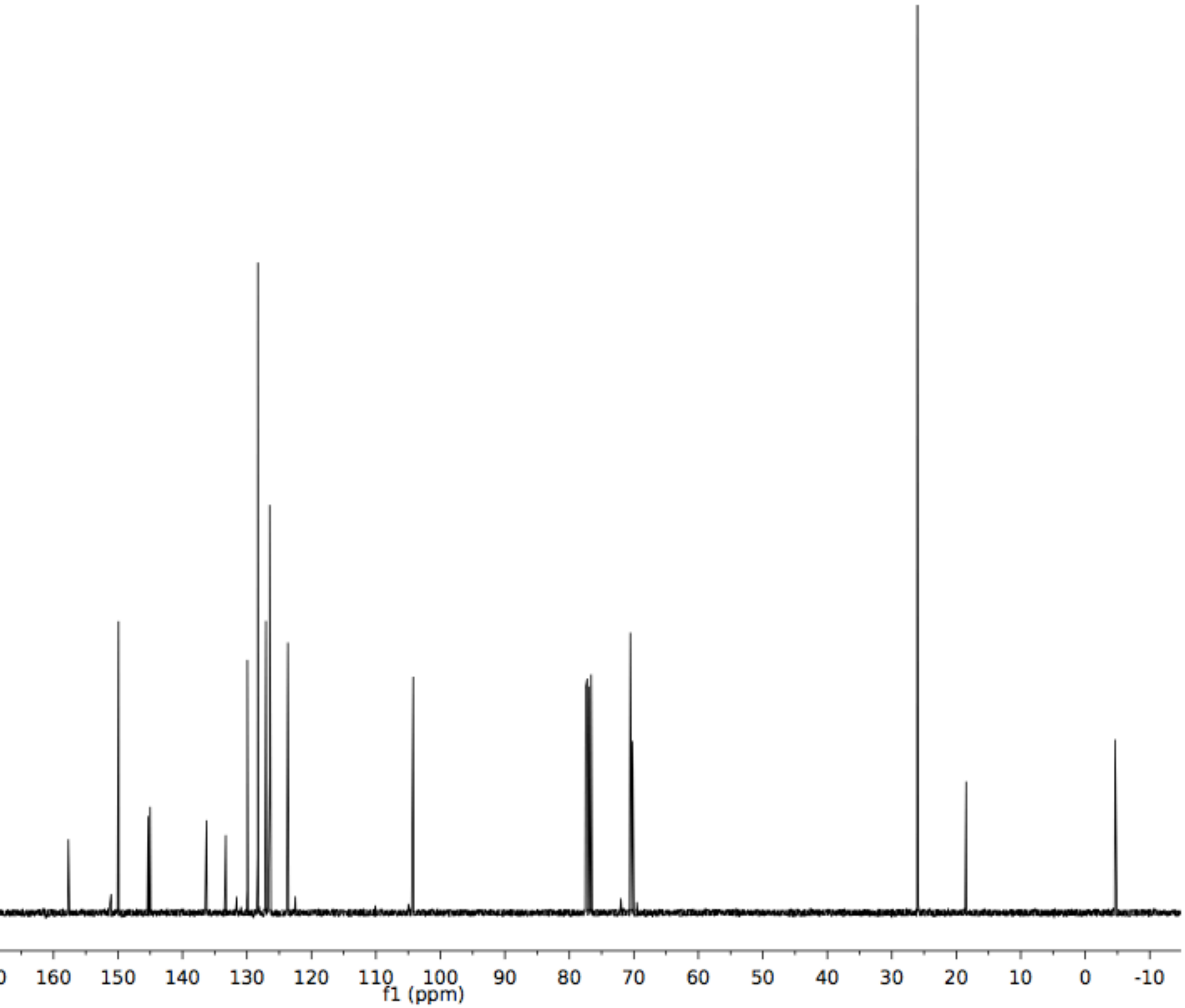

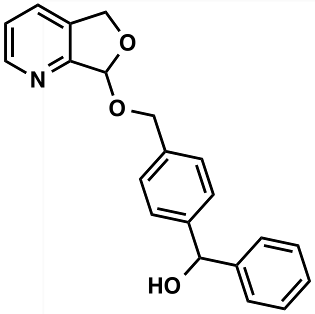

11
^{1}H NMR
(500 MHz, $CDCl_3$)

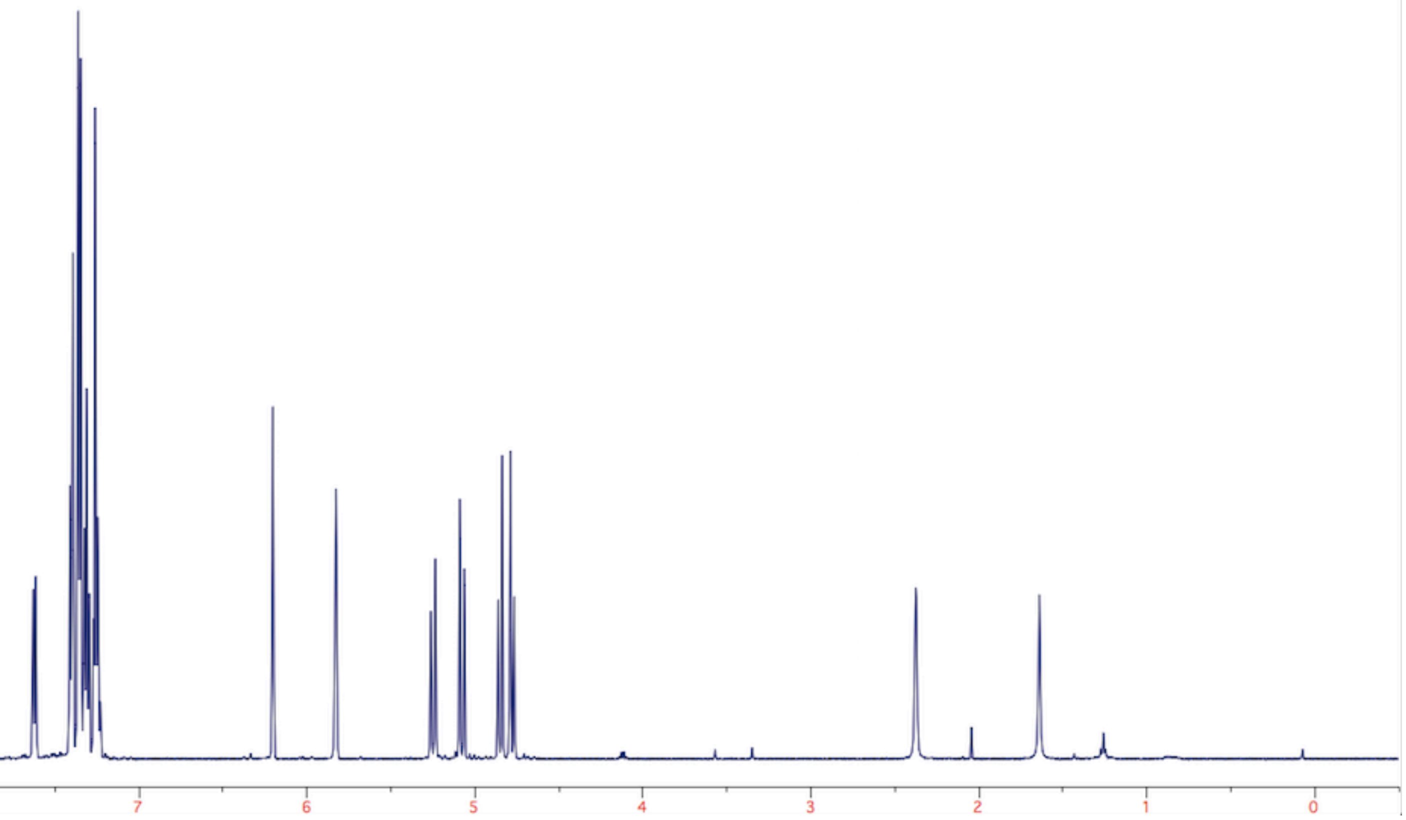

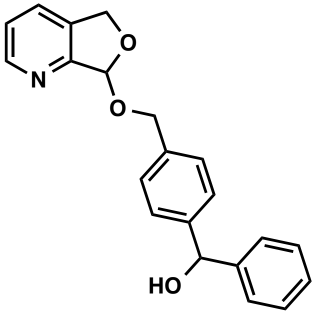

11
^{13}C NMR
(125 MHz, $CDCl_3$)

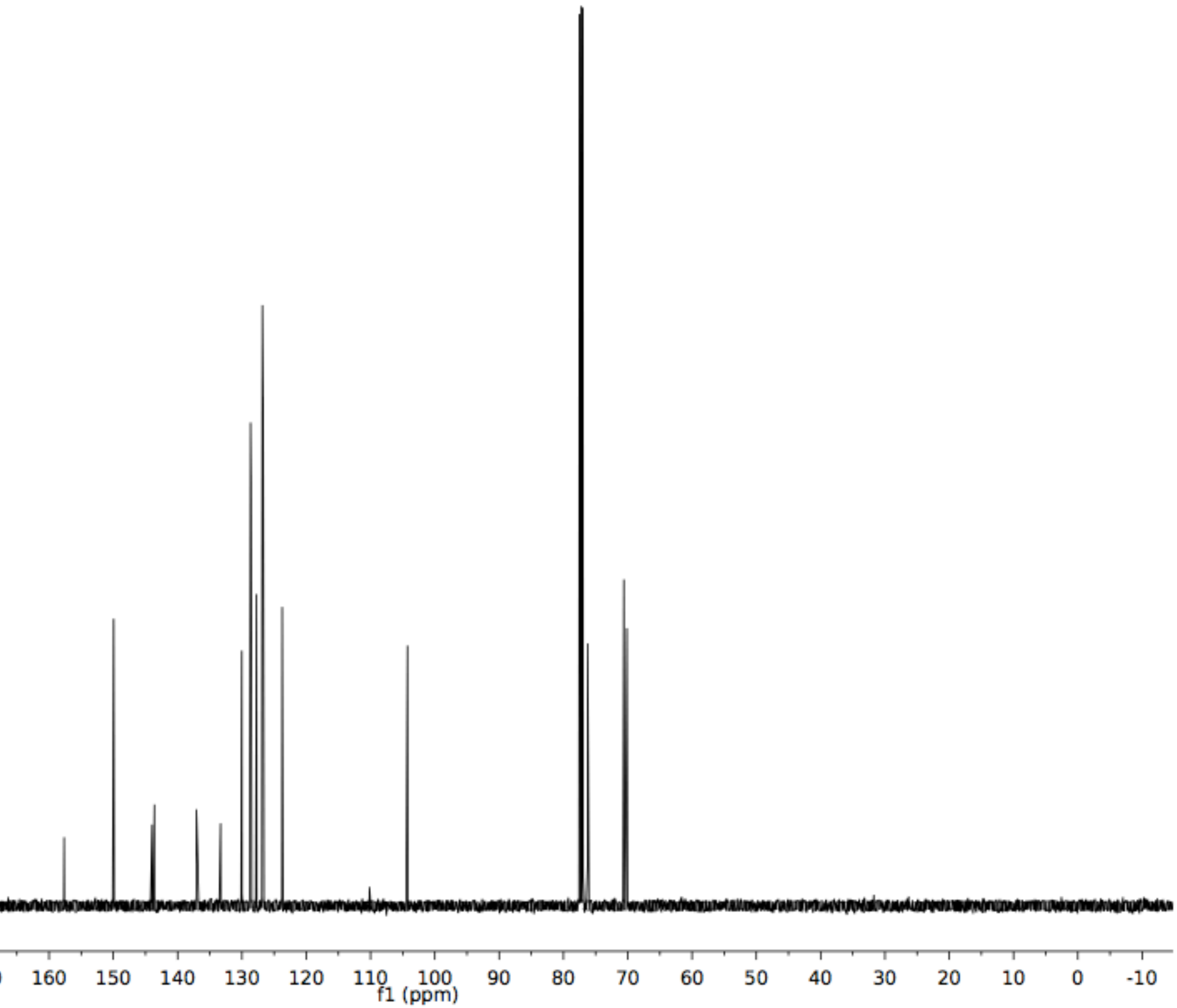

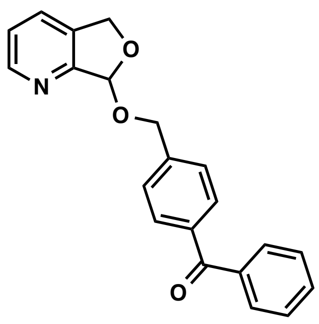

7n
^{1}H NMR
(400 MHz, $CDCl_3$)

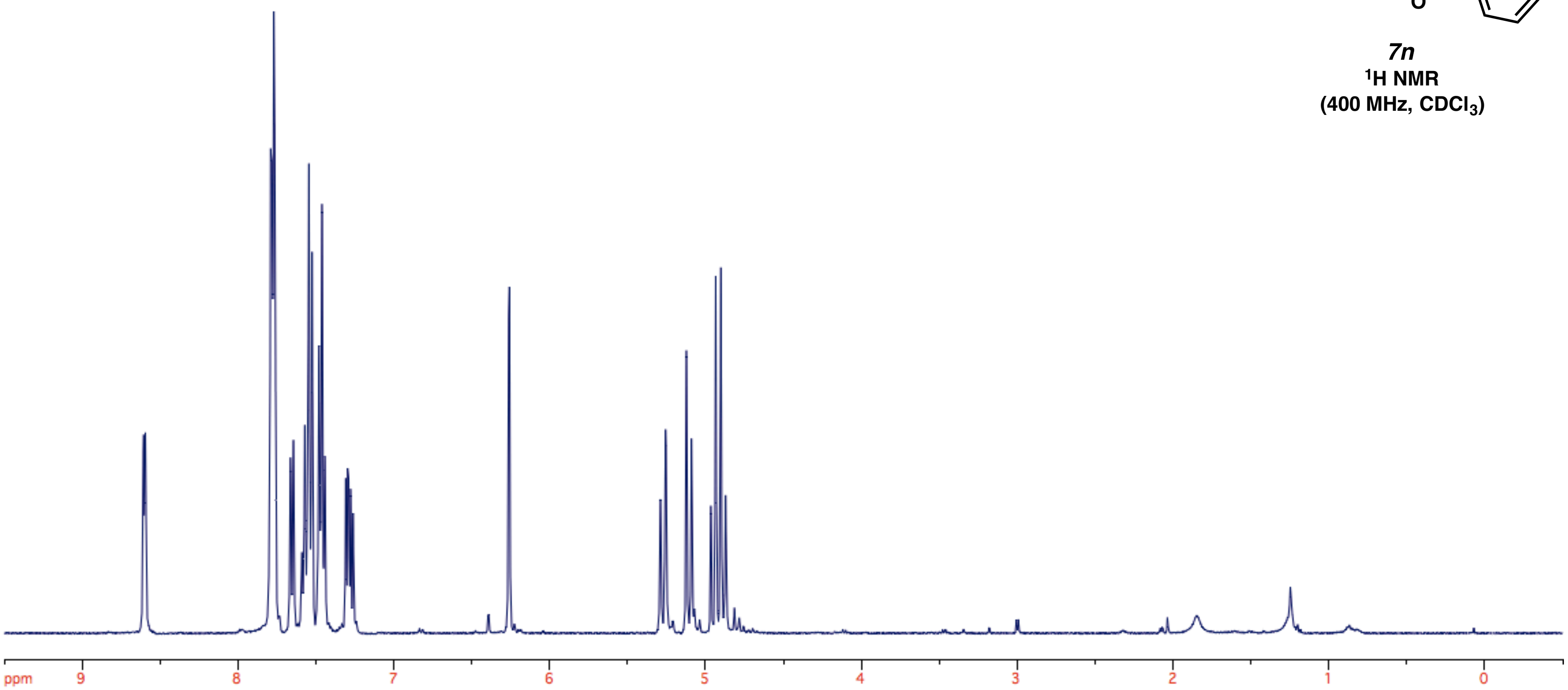

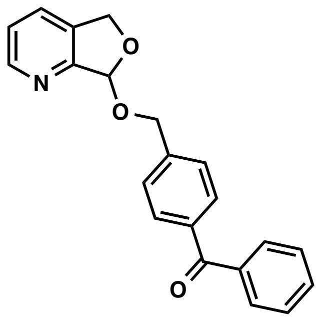

7n
^{13}C NMR
(100 MHz, $CDCl_3$)

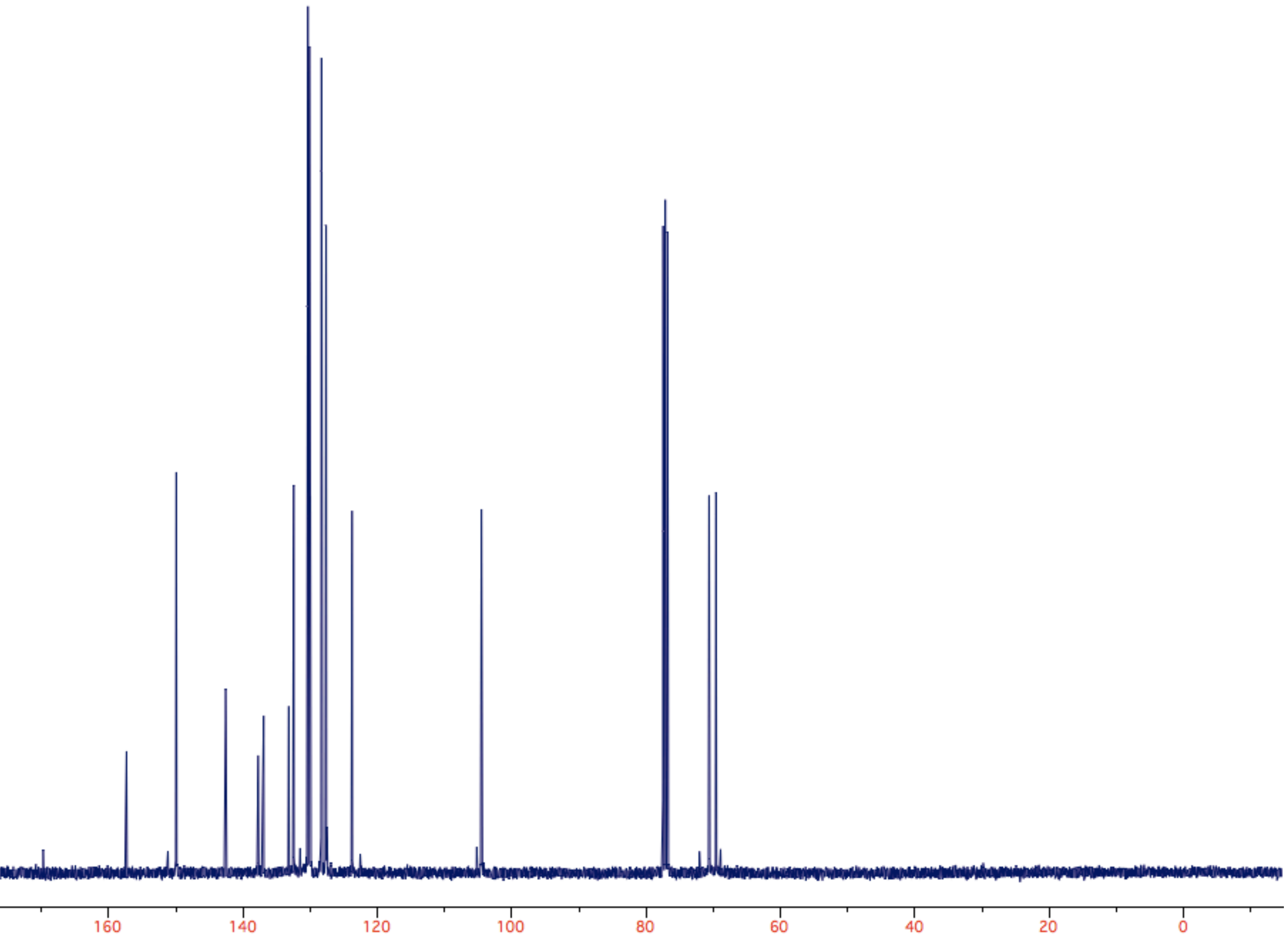

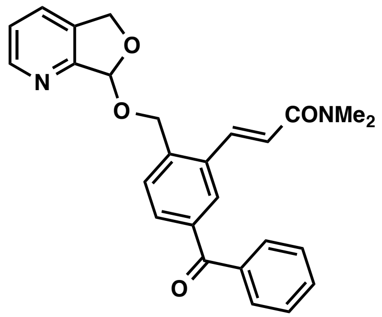

***8nb**$_{mono}$*
^{1}H NMR
(400 MHz, $CDCl_3$)

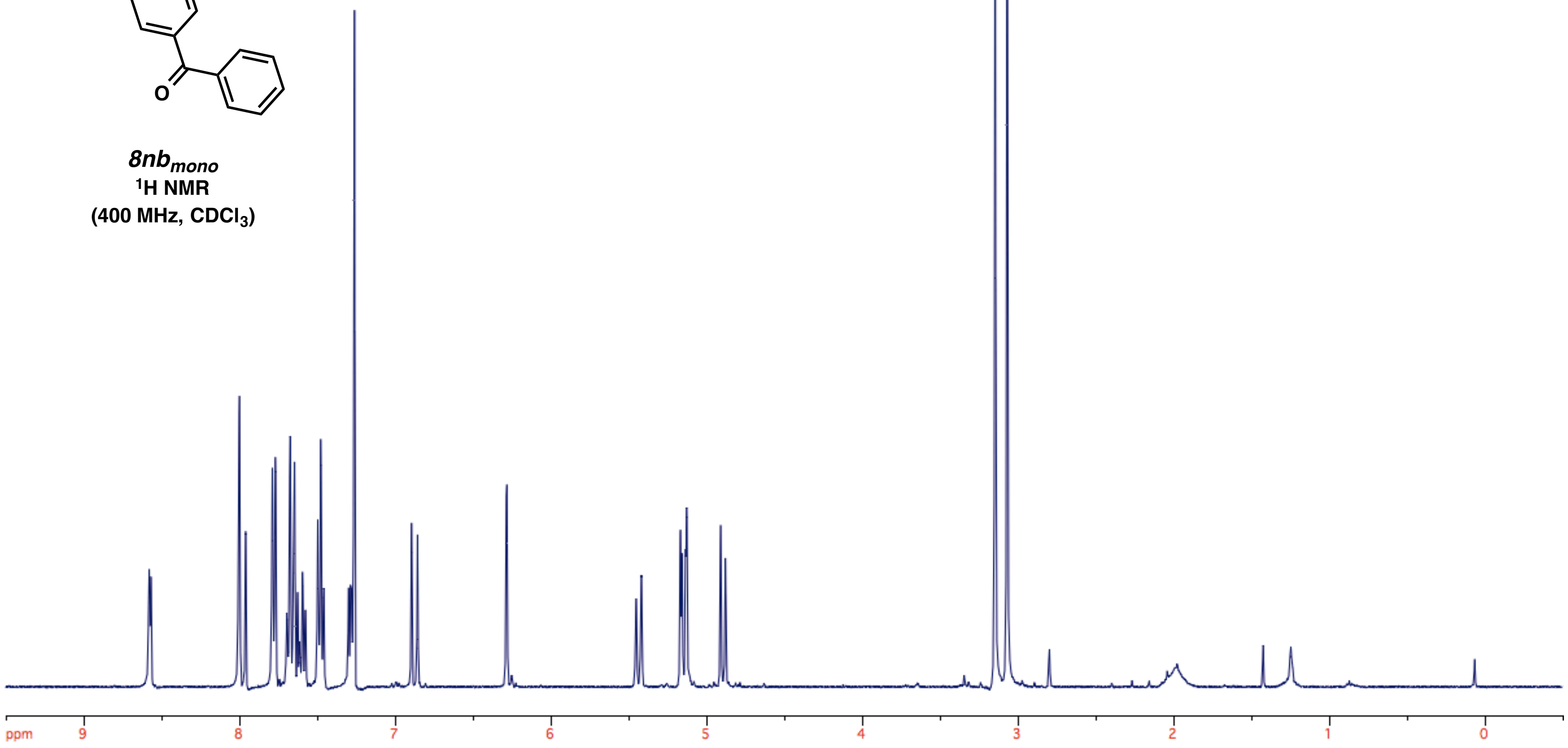

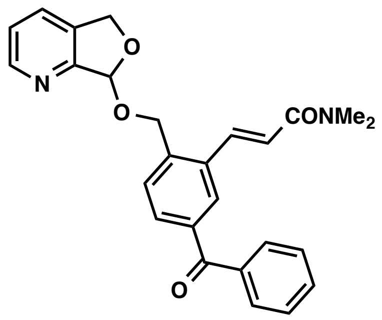

***8nb**$_{mono}$*
^{13}C NMR
(100 MHz, $CDCl_3$)

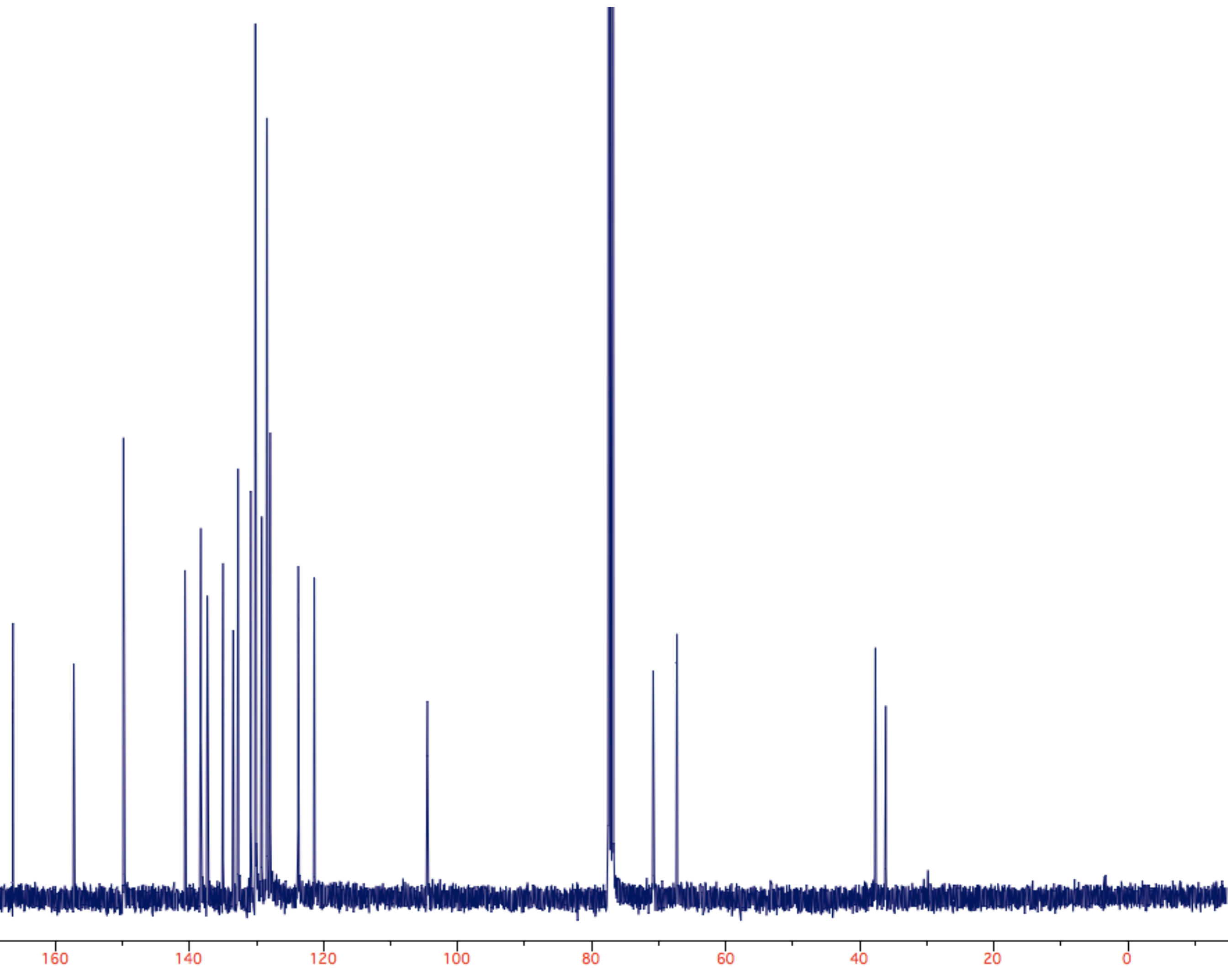

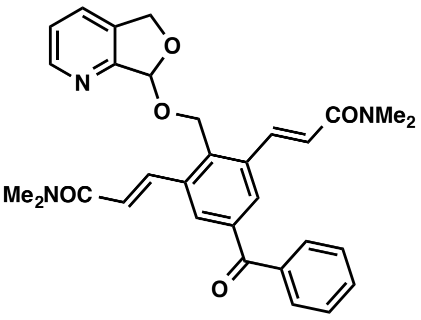

8nb$_{di}$
^{1}H NMR
(500 MHz, $CDCl_3$)

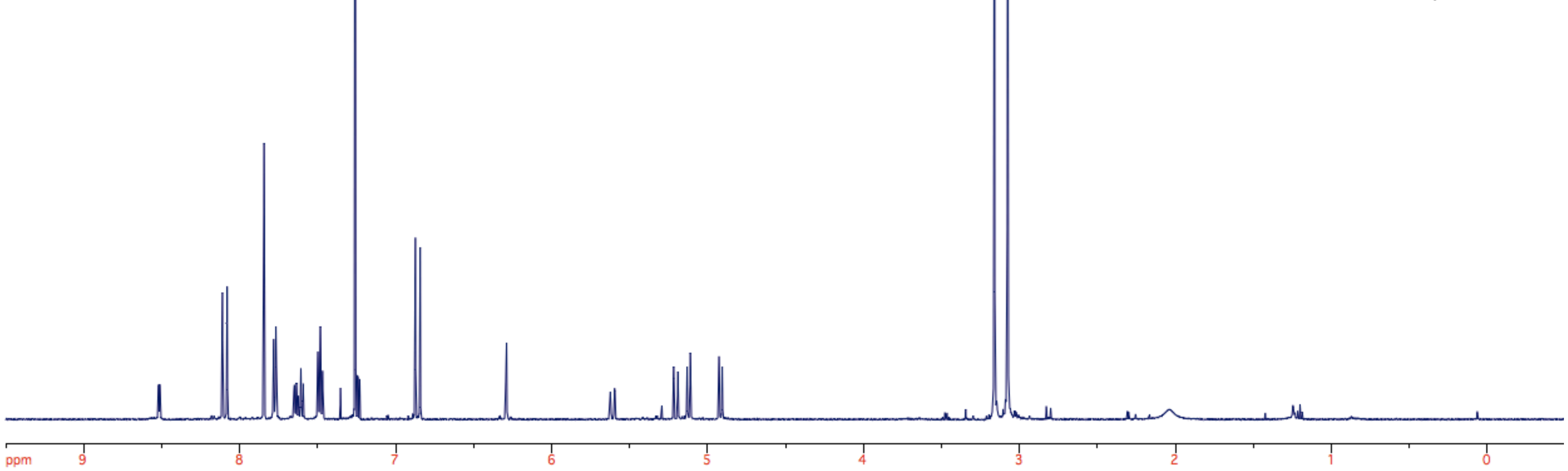

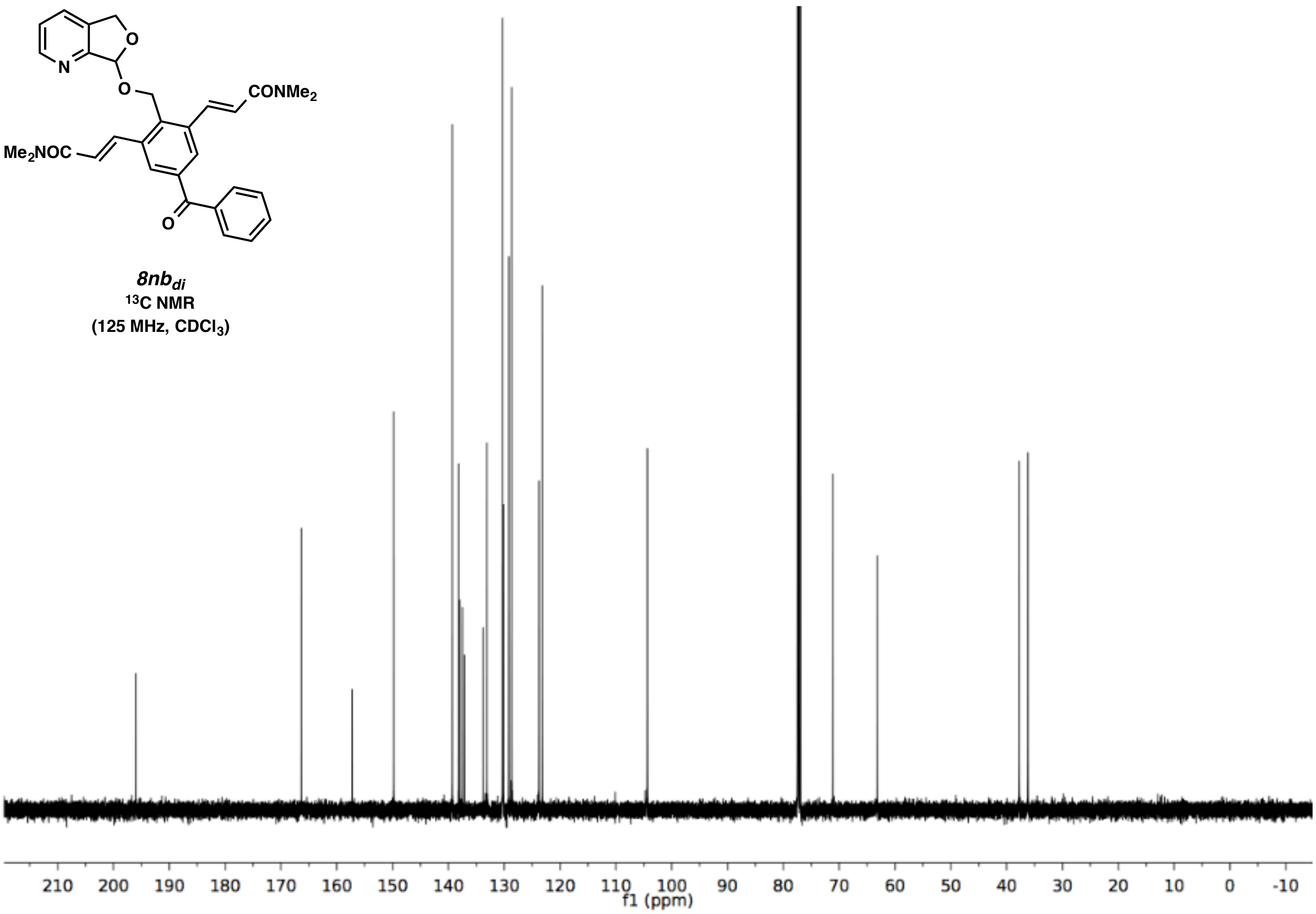

***8nb**$_{di}$*
^{13}C NMR
(125 MHz, $CDCl_3$)

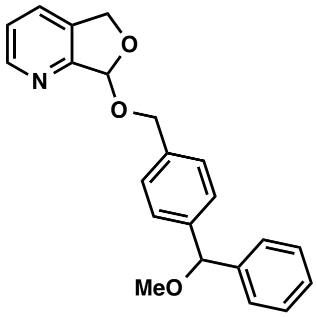

7o
^{1}H NMR
(500 MHz, $CDCl_3$)

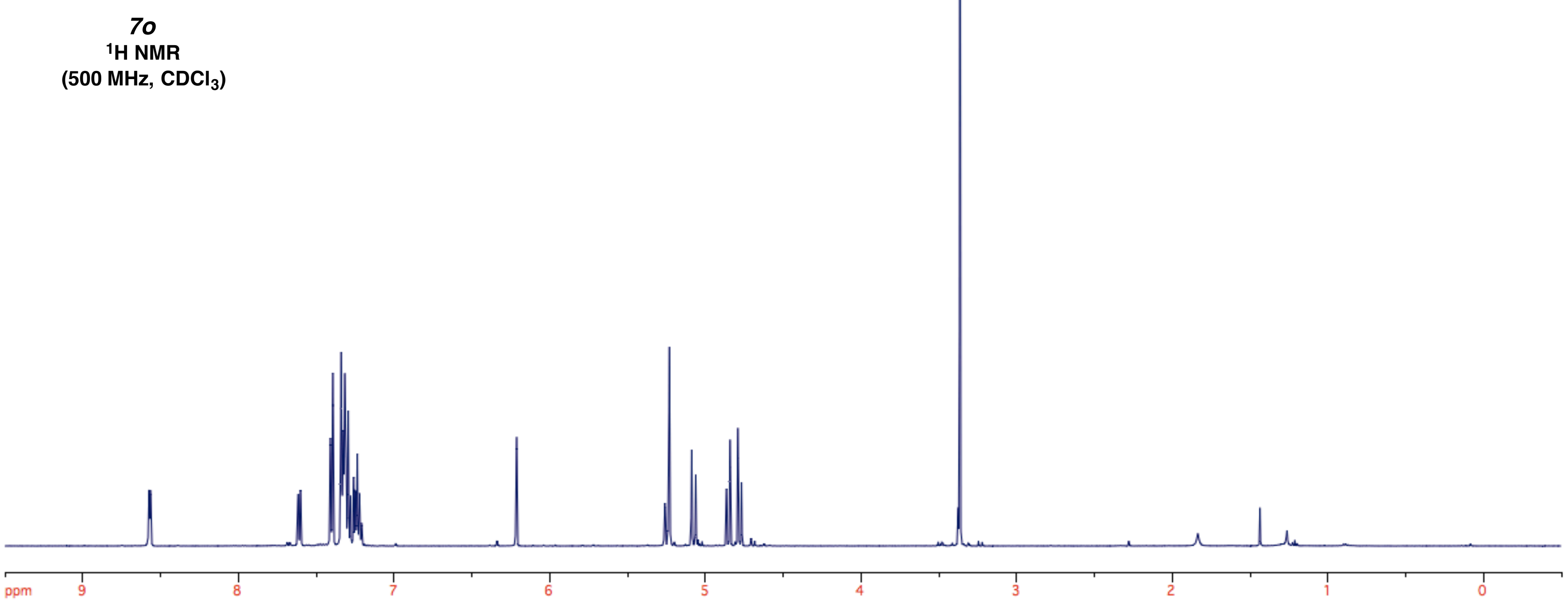

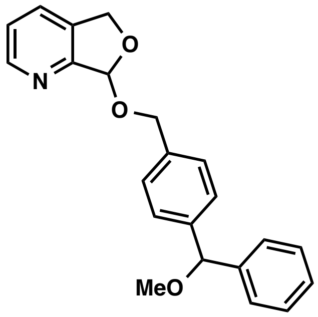

7o
^{13}C NMR
(125 MHz, $CDCl_3$)

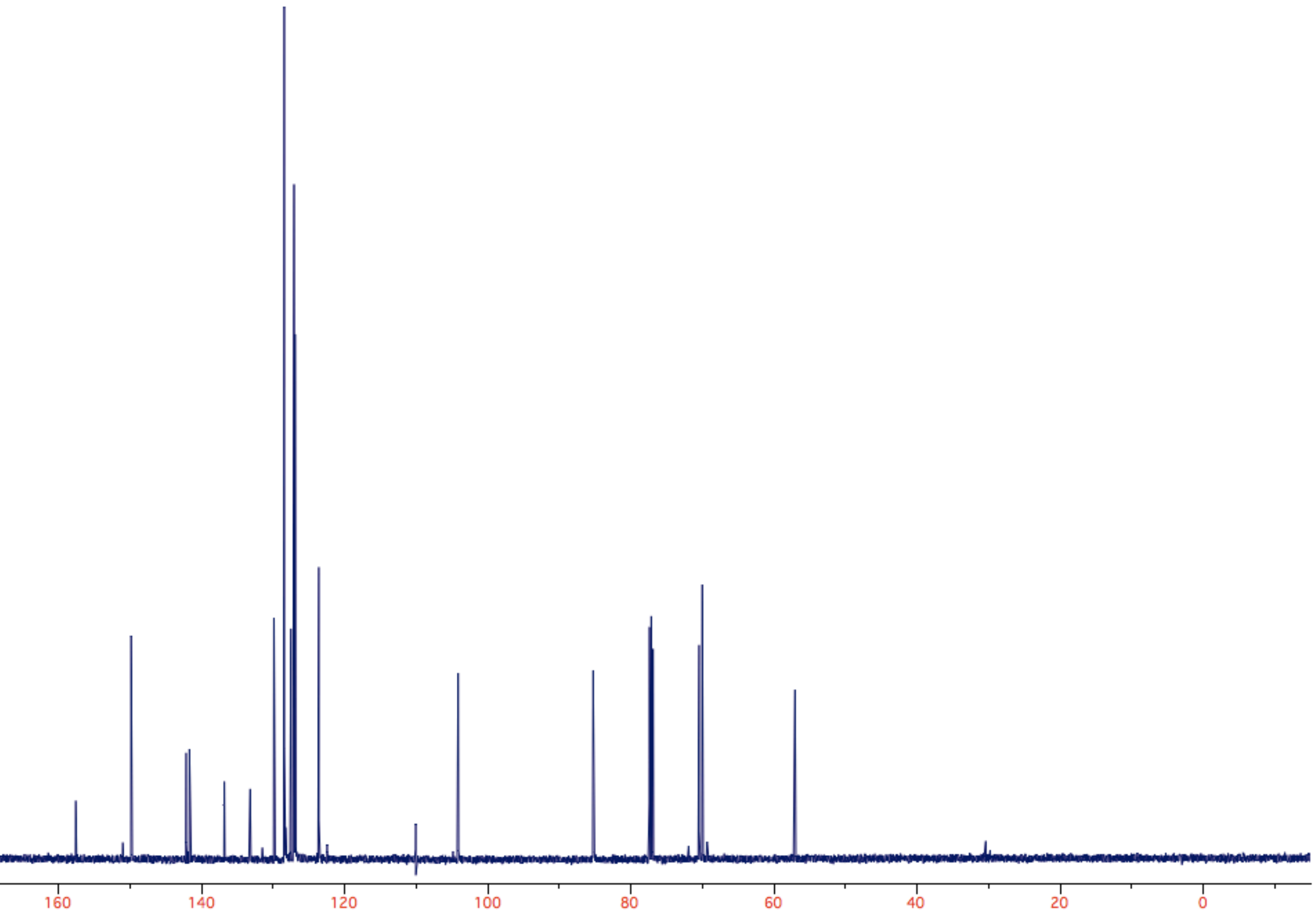

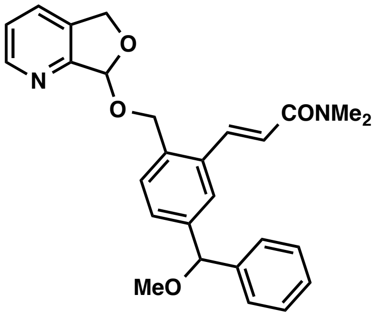

***8ob**$_{mono}$*
^{1}H NMR
(500 MHz, $CDCl_3$)

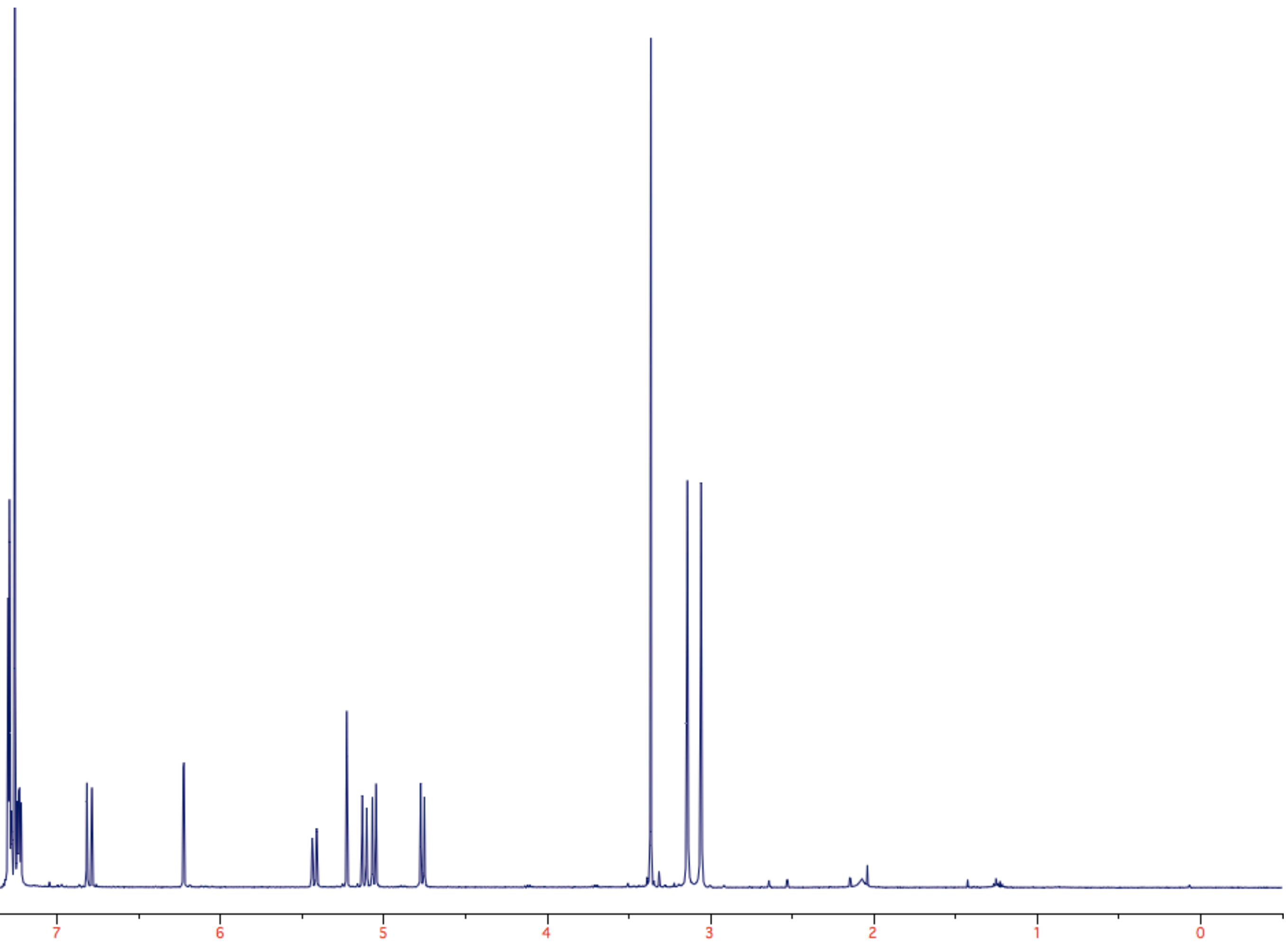

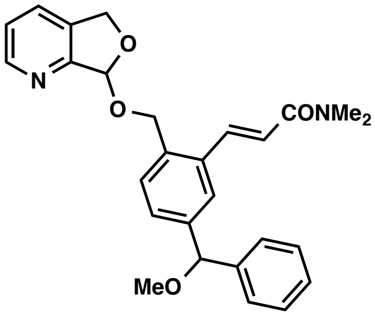

***8ob**mono*

^{13}C NMR

(125 MHz, $CDCl_3$)

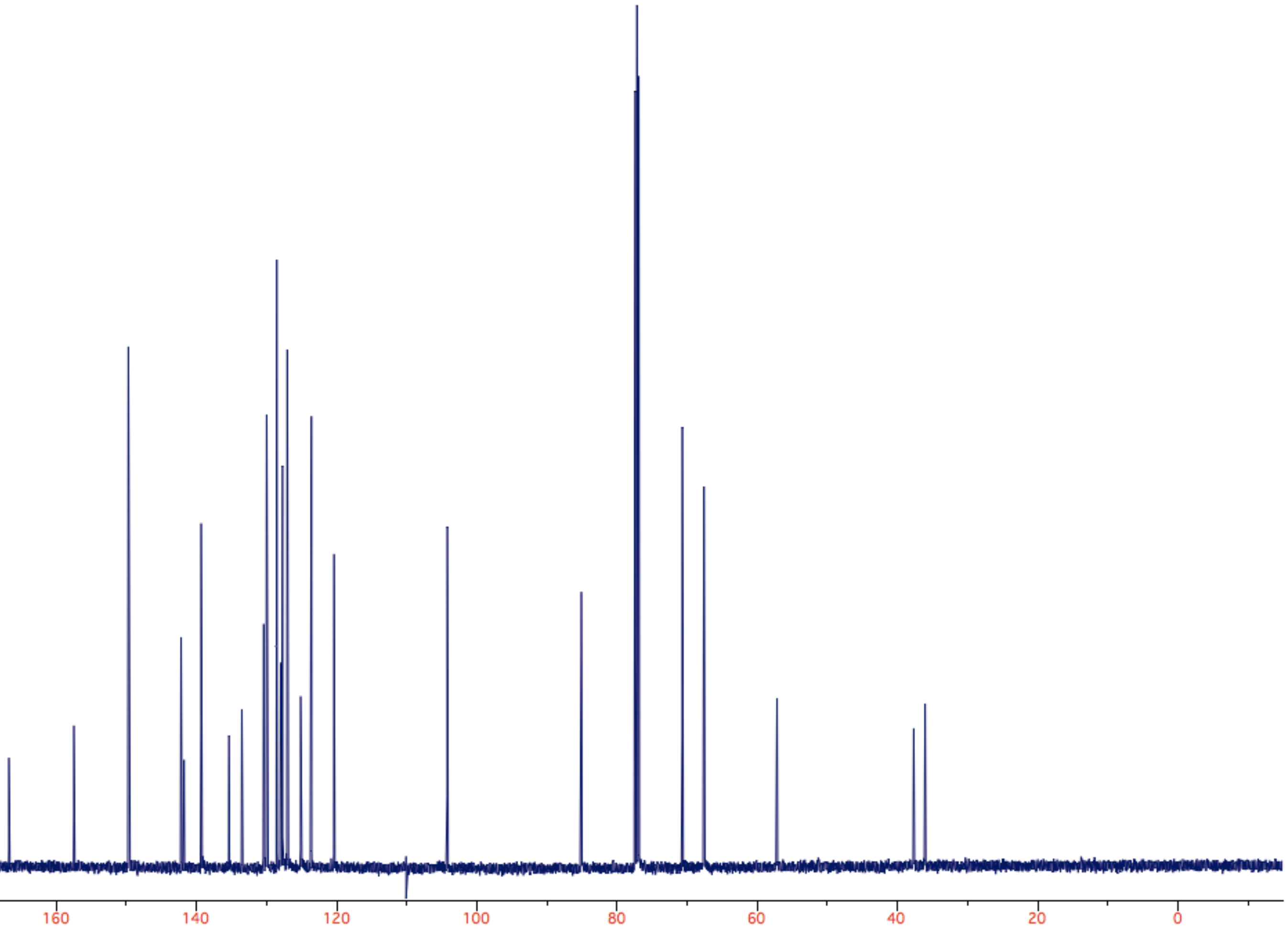

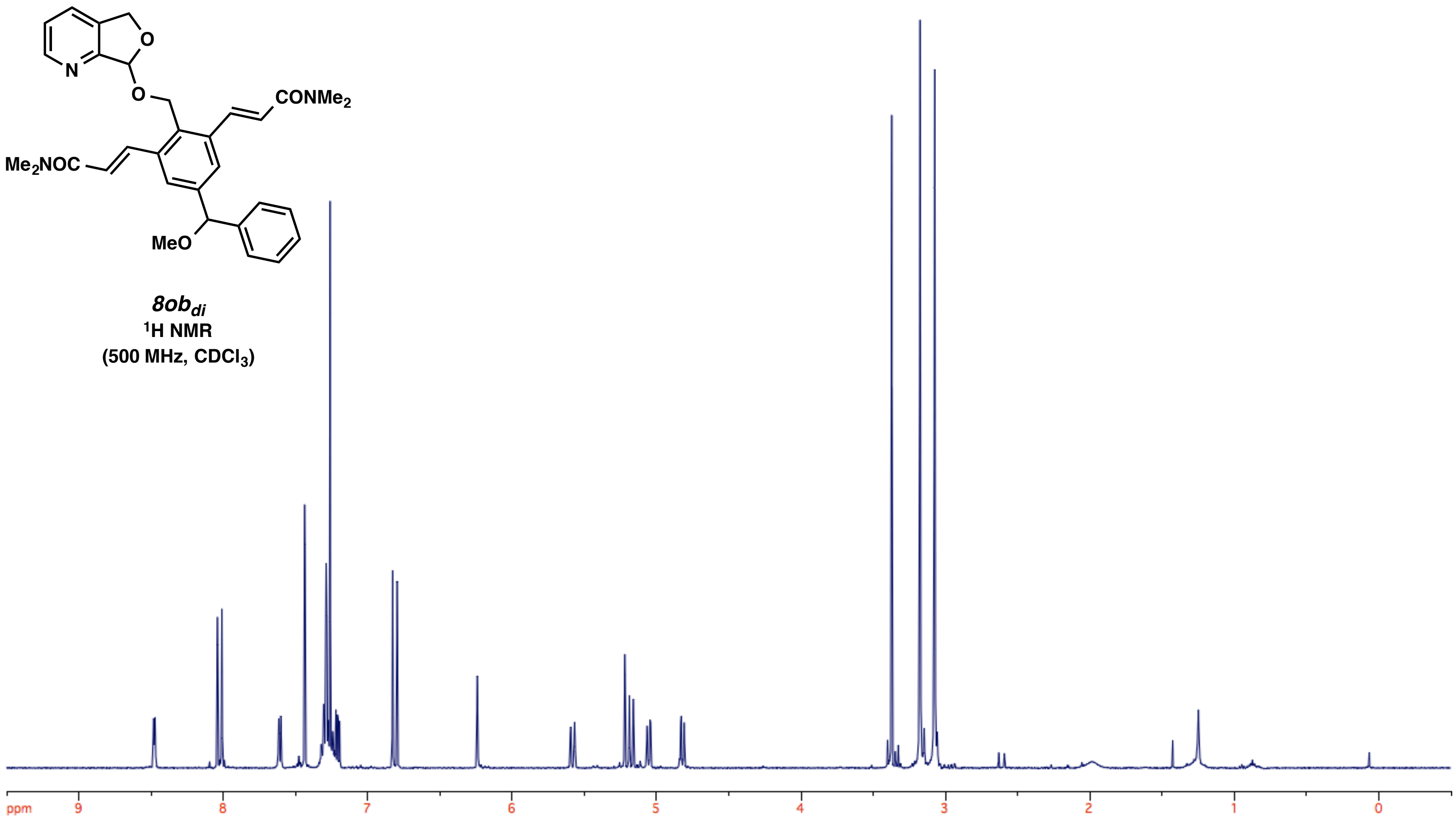

***8ob**$_{di}$*

^{1}H NMR

(500 MHz, $CDCl_3$)

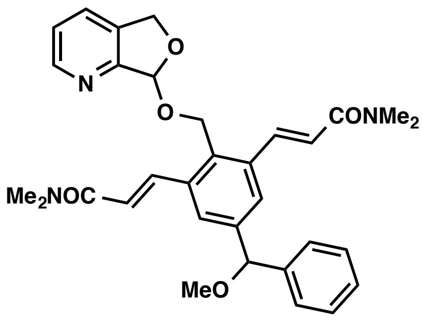

***8ob**$_{di}$*
^{13}C NMR
(125 MHz, $CDCl_3$)

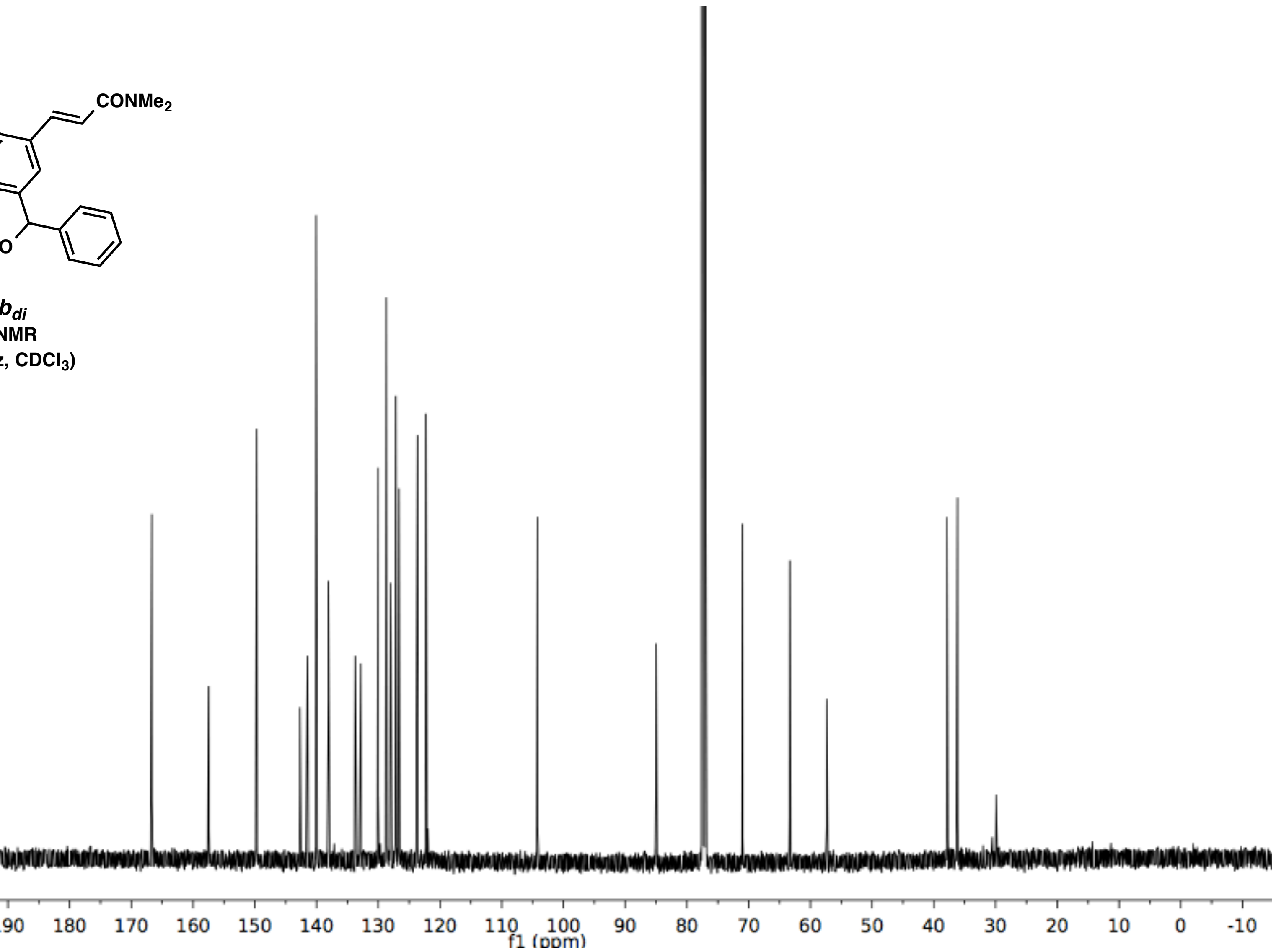

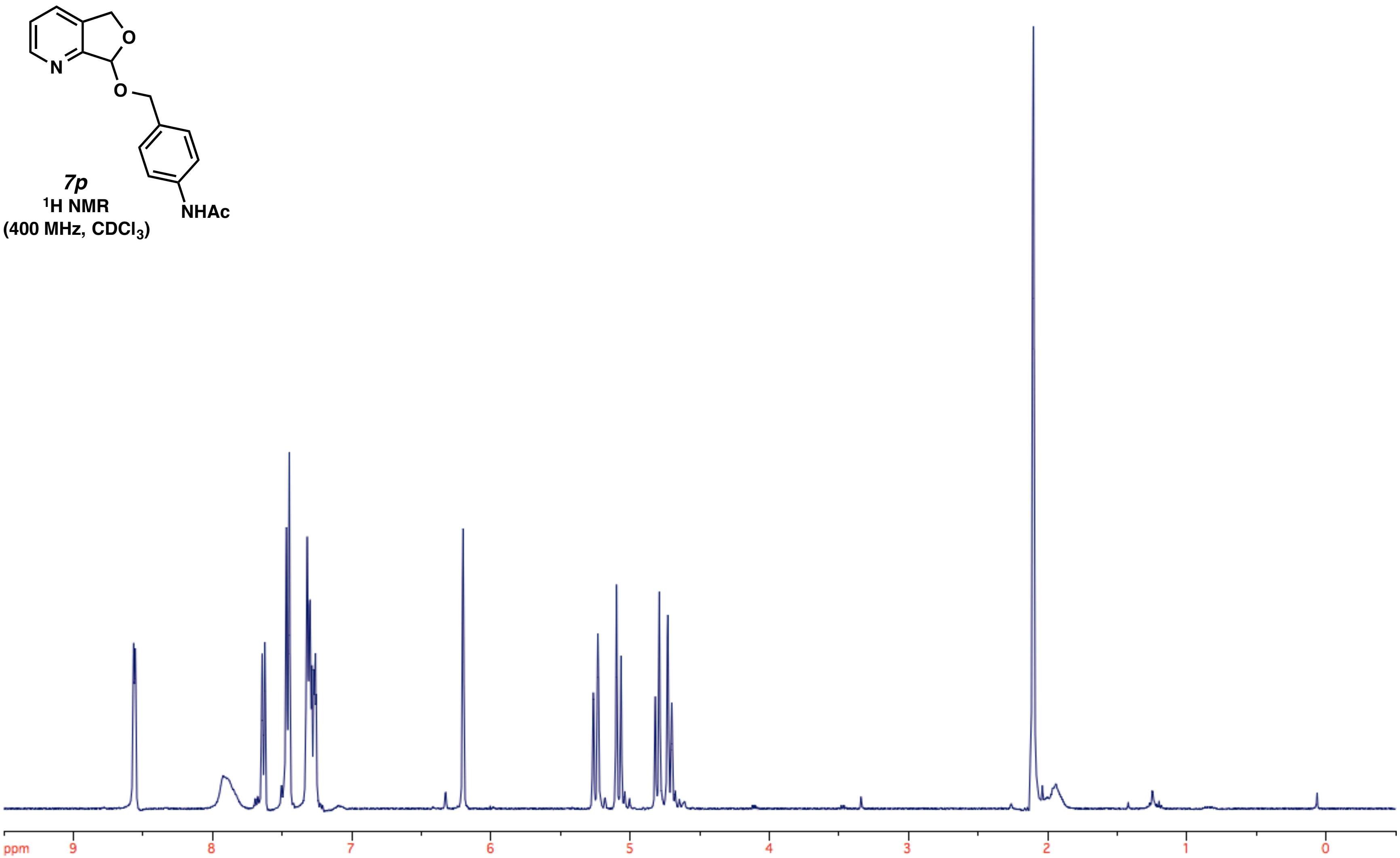

7p
^{1}H NMR
(400 MHz, $CDCl_3$)

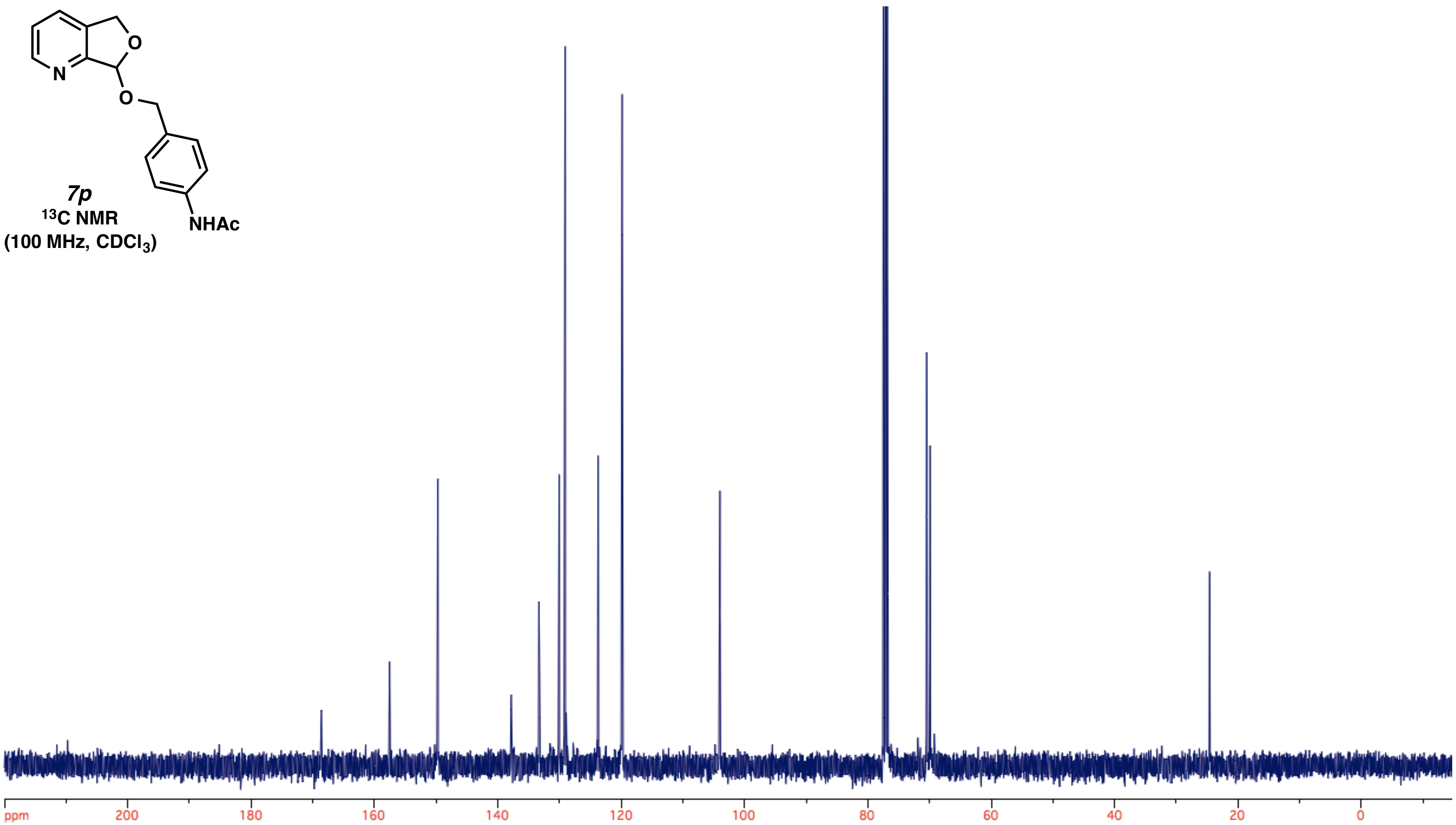

7p
^{13}C NMR
(100 MHz, $CDCl_3$)

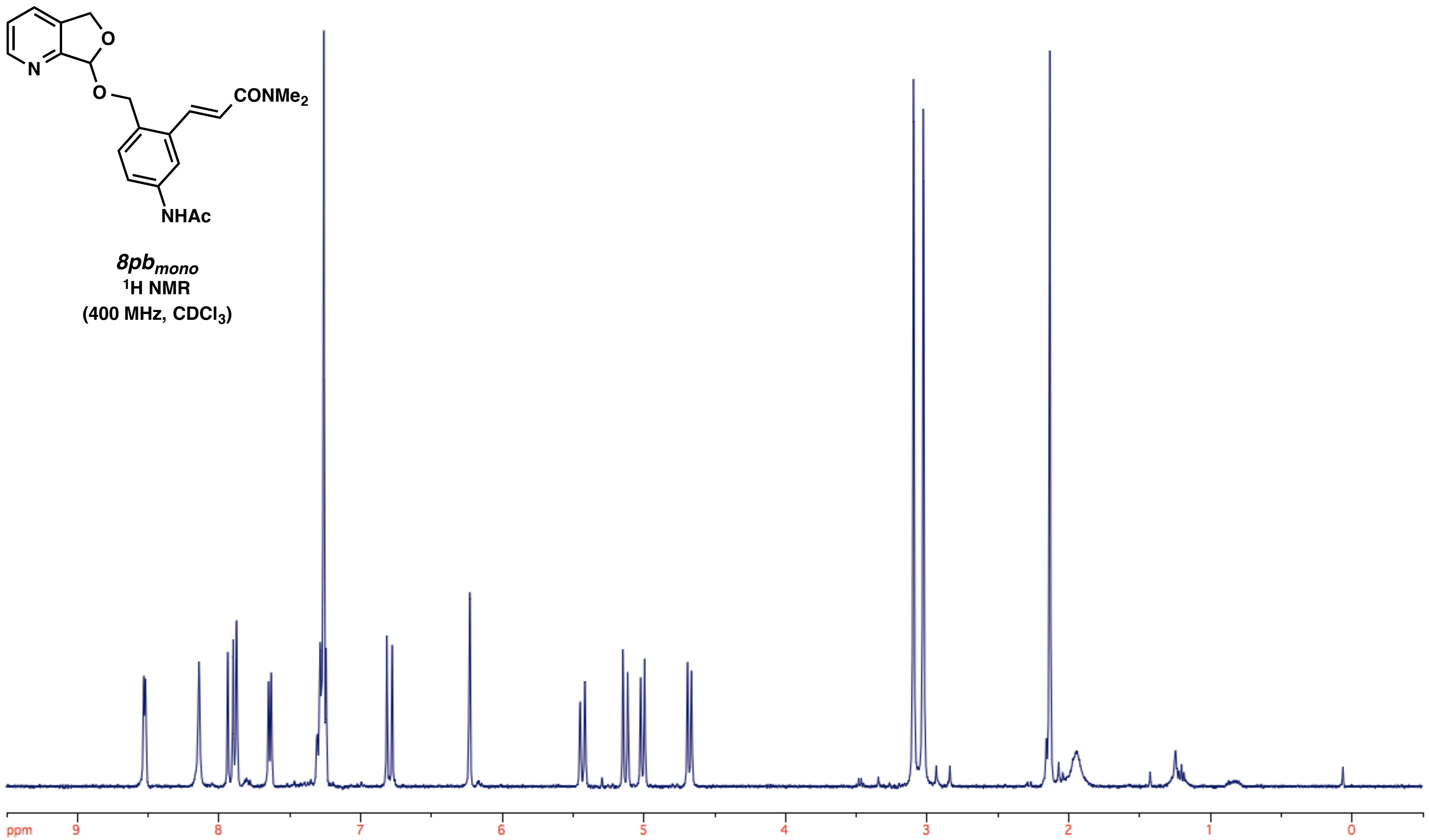

***8pb**_mono_*
^{1}H NMR
(400 MHz, $CDCl_3$)

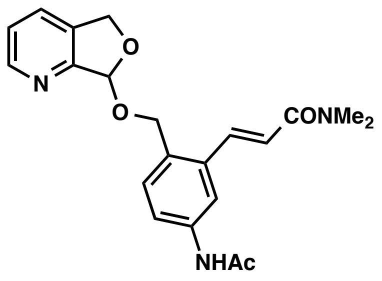

***8pb**_mono_*
^{13}C NMR
(100 MHz, $CDCl_3$)

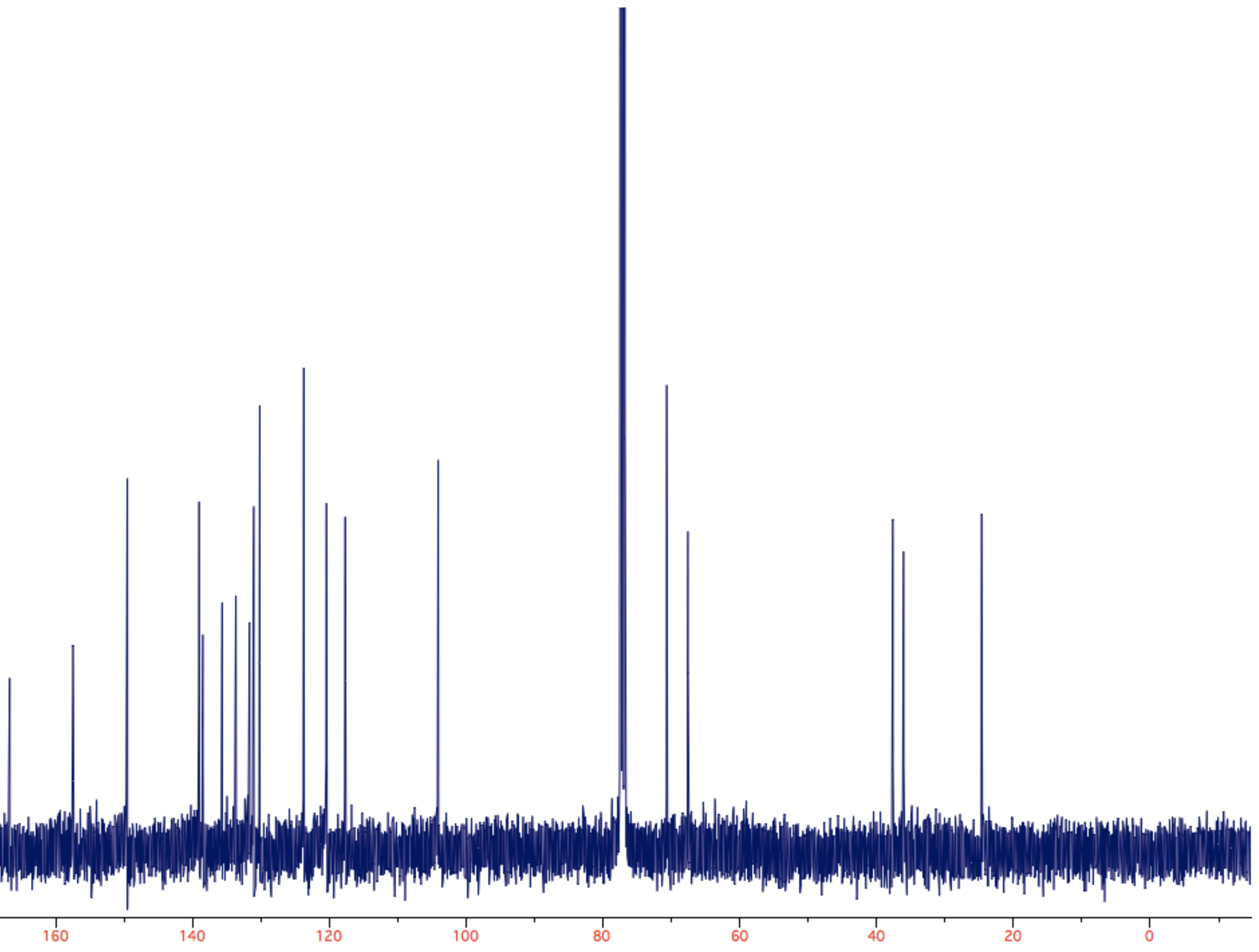

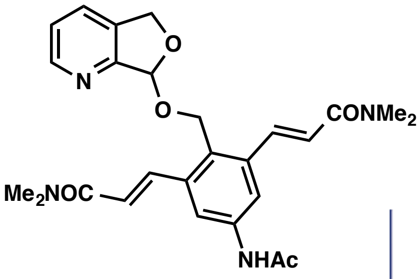

8pb$_{di}$
^{1}H NMR
(400 MHz, $CDCl_3$)
7.9:1 di/mono

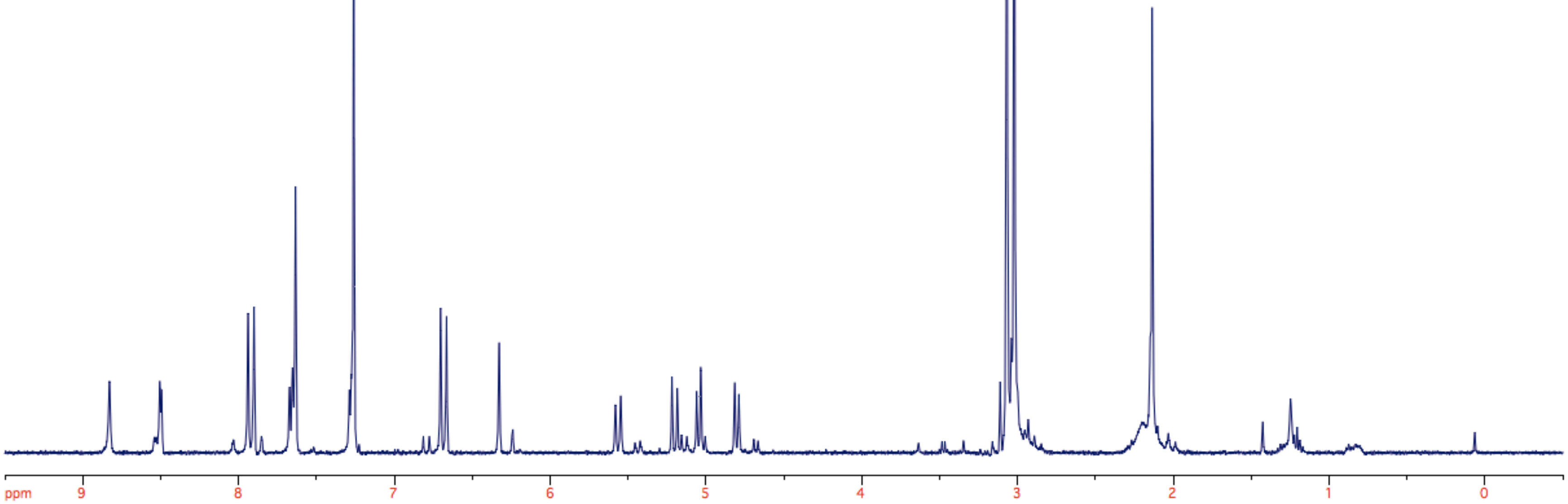

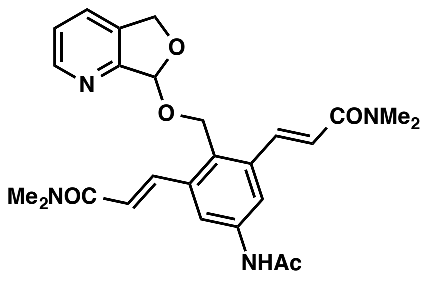

***8pb**$_{di}$*
^{13}C NMR
(400 MHz, $CDCl_3$)
7.9:1 di/mono

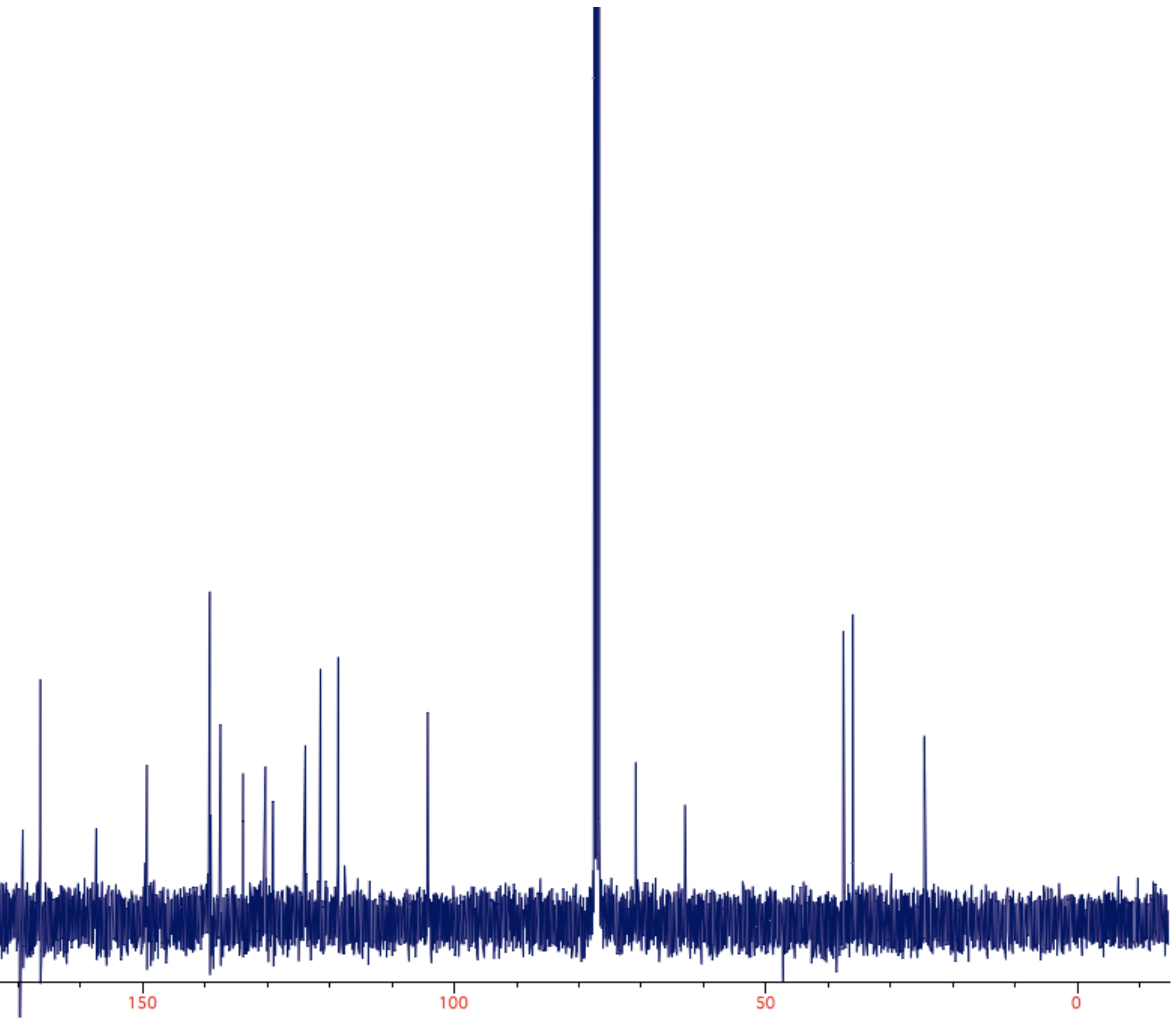

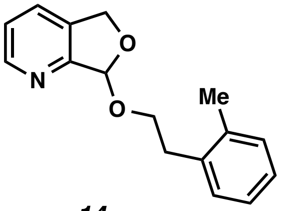

14
^{1}H NMR
(400 MHz, $CDCl_3$)

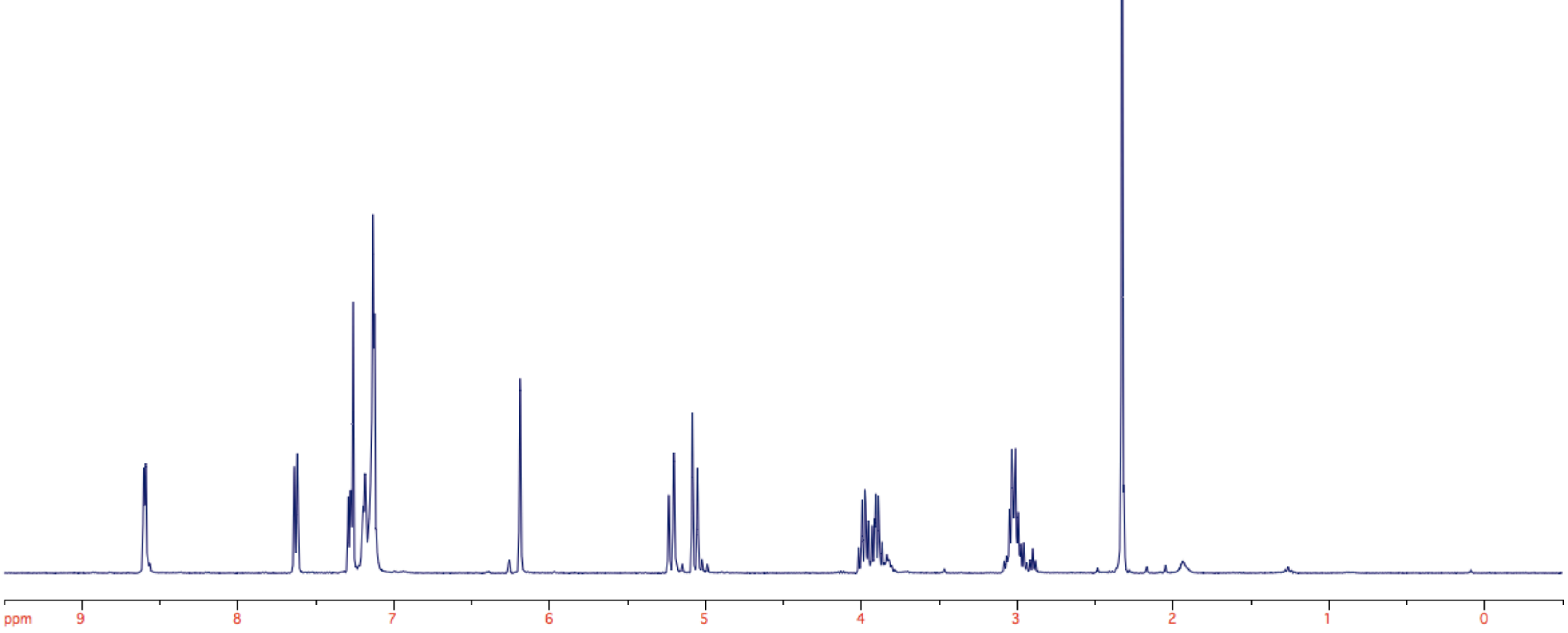

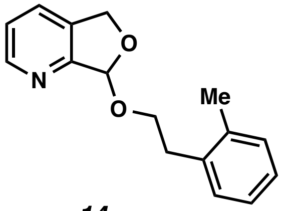

14
^{13}C NMR
(100 MHz, $CDCl_3$)

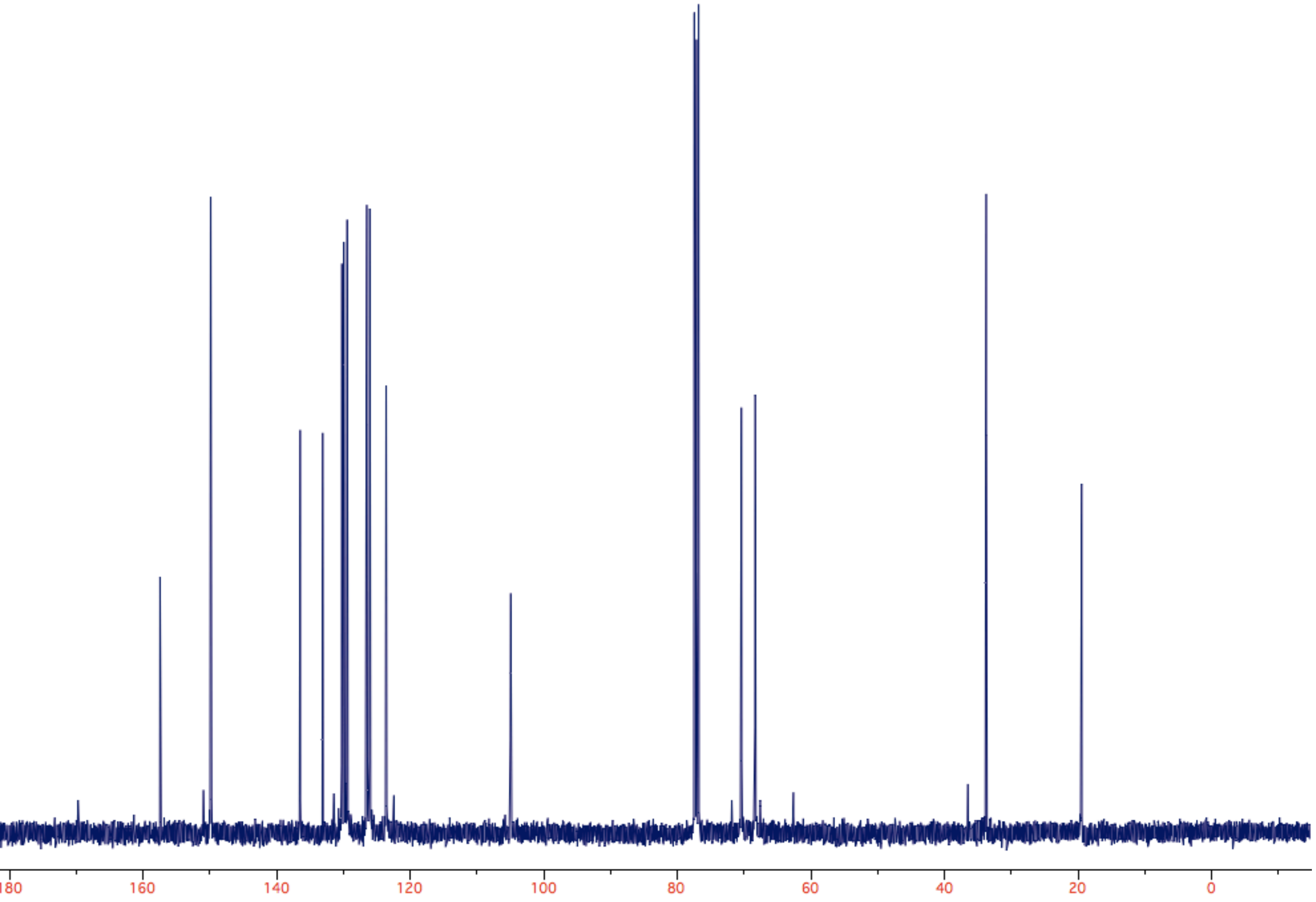

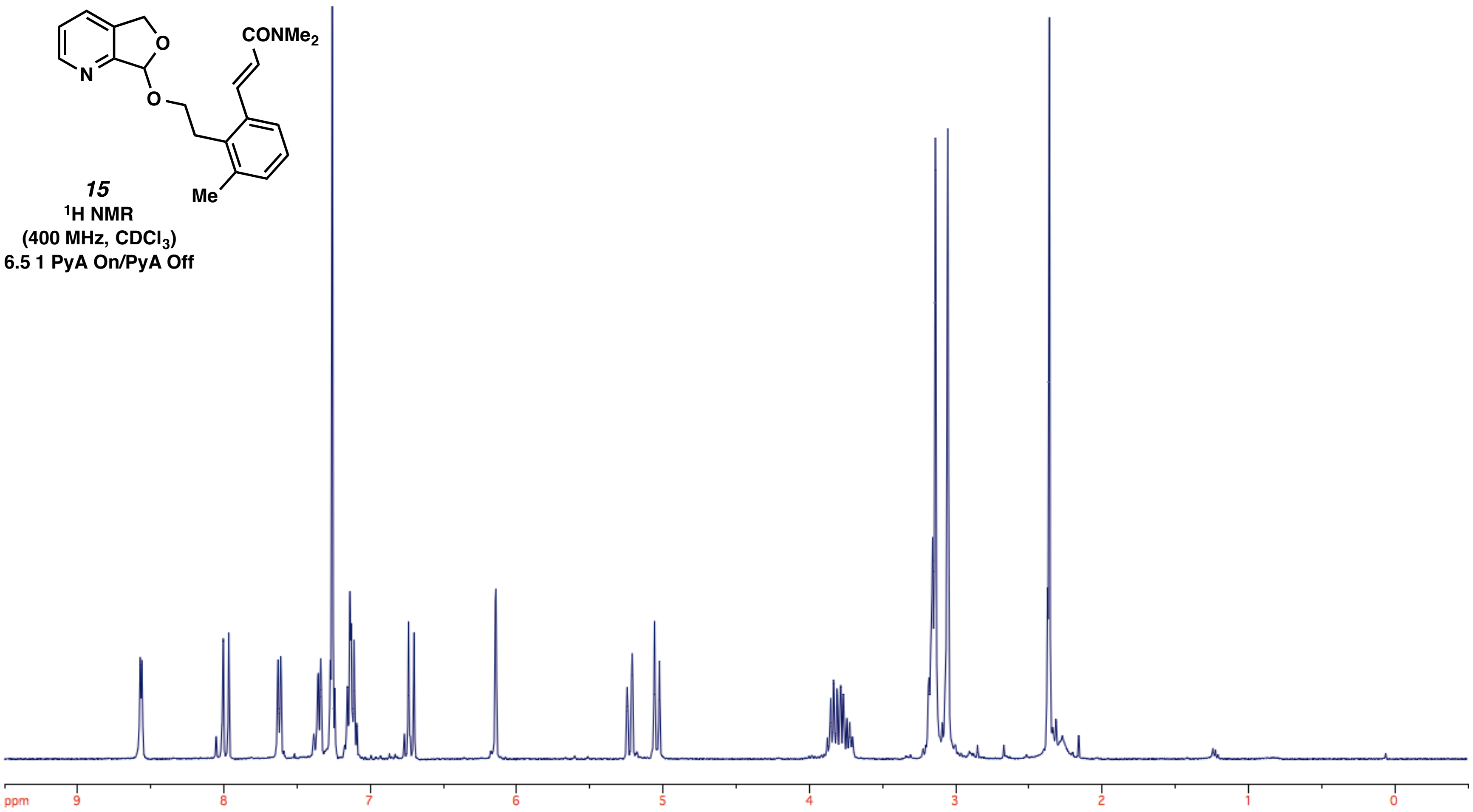
CONNMe2
O
N
O
Me
15
1H NMR
(400 MHz, CDCl3)
6.5 1 PyA On/PyA Off
ppm
9
8
7
6
5
4
3
2
1
0

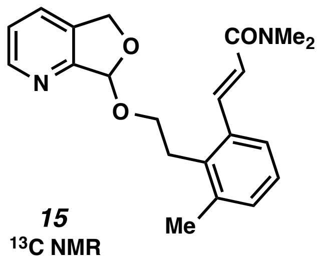

15
^{13}C NMR
(100 MHz, $CDCl_3$)
6.5:1 PyA On/PyA Off

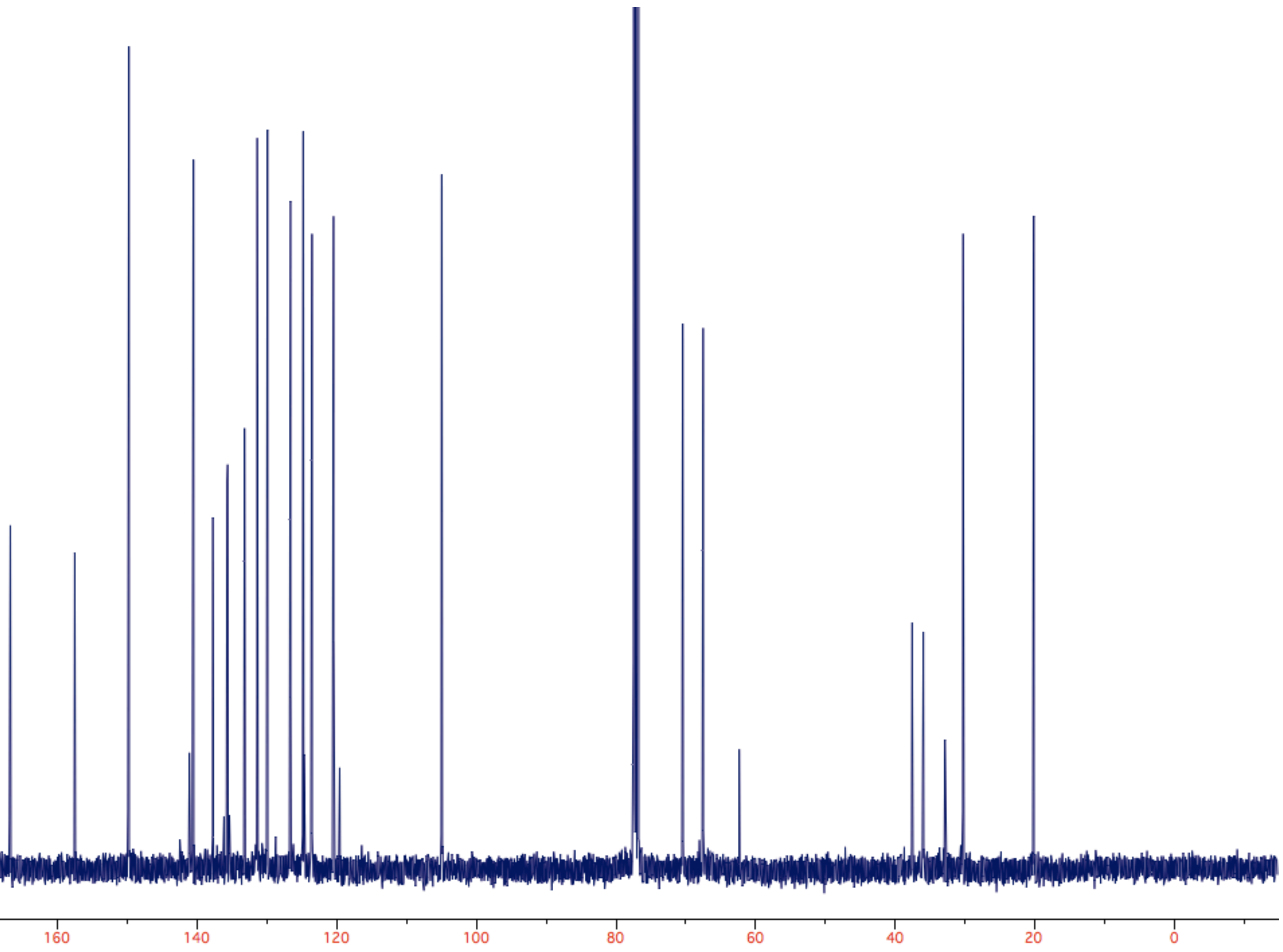

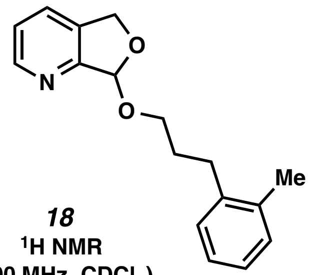

18
^{1}H NMR
(400 MHz, $CDCl_3$)

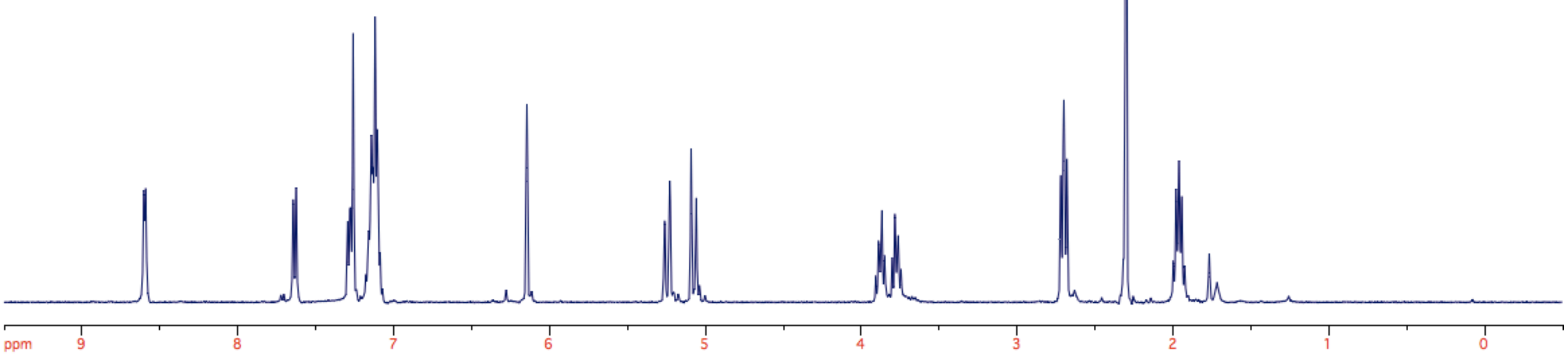

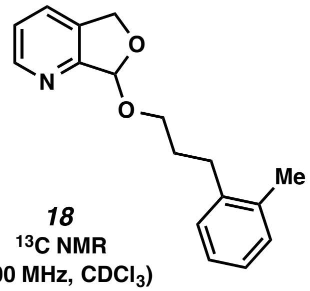

18
^{13}C NMR
(100 MHz, $CDCl_3$)

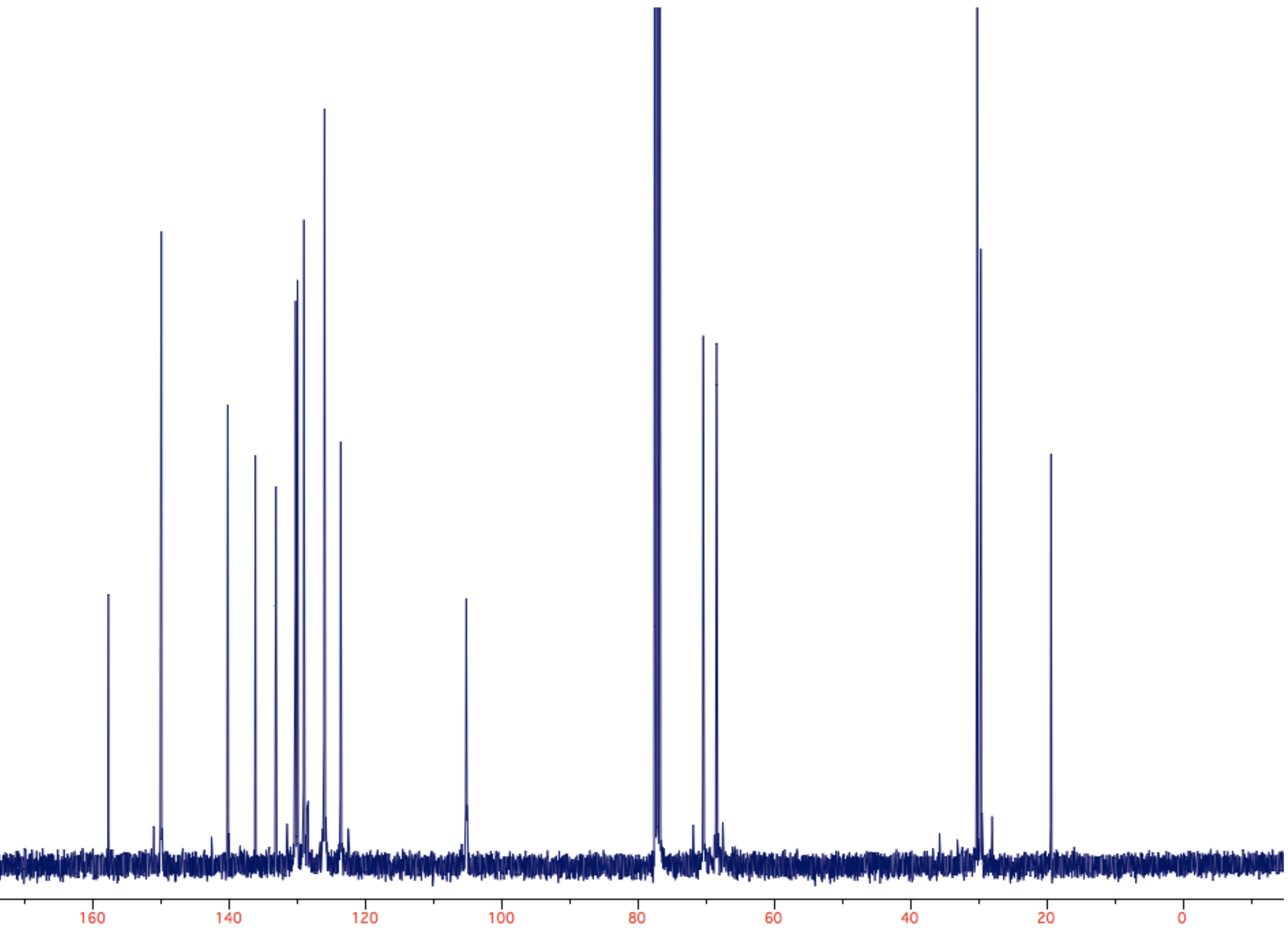

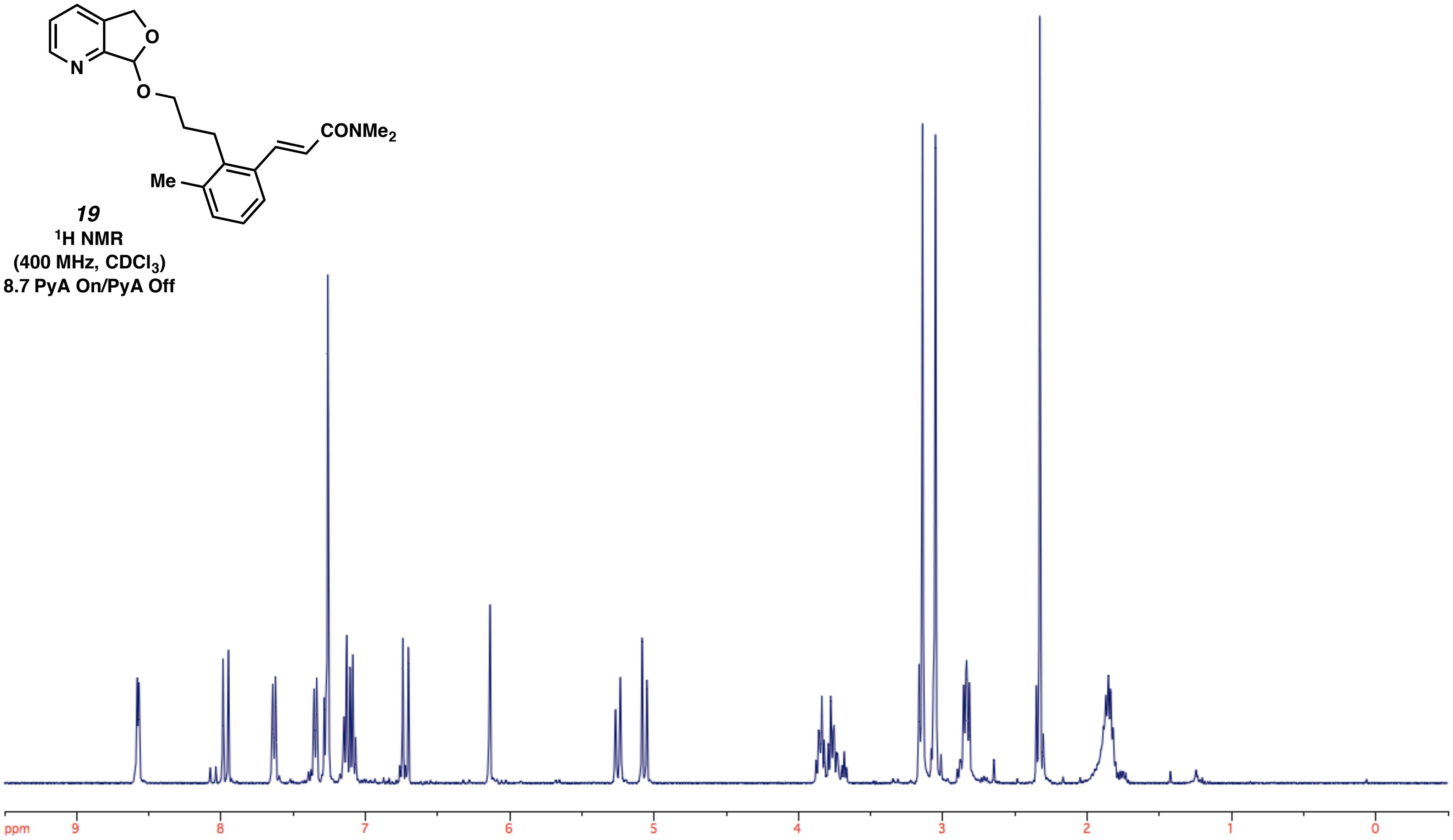

19
^{1}H NMR
(400 MHz, $CDCl_3$)
8.7 PyA On/PyA Off

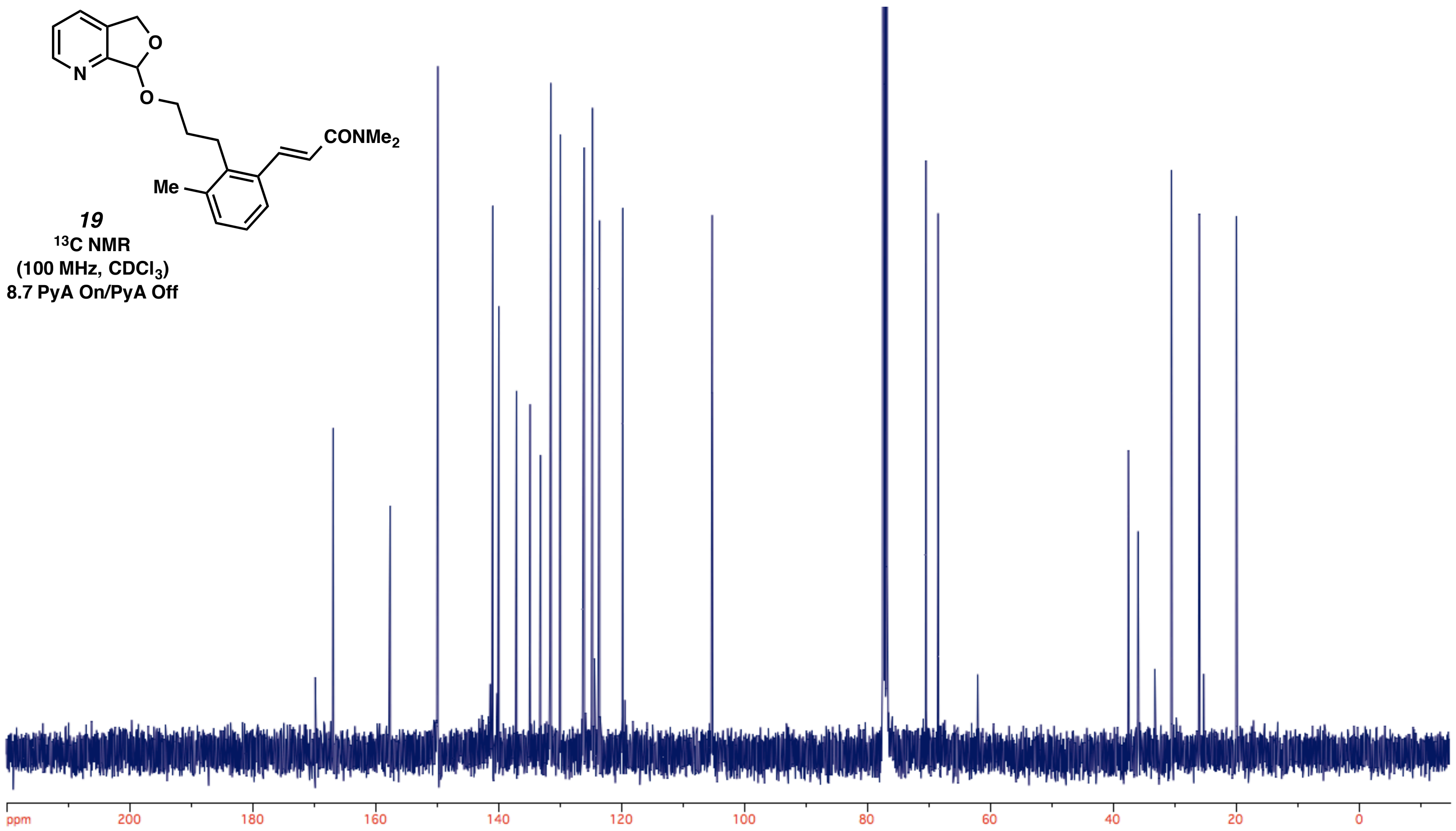

19
^{13}C NMR
(100 MHz, $CDCl_3$)
8.7 PyA On/PyA Off

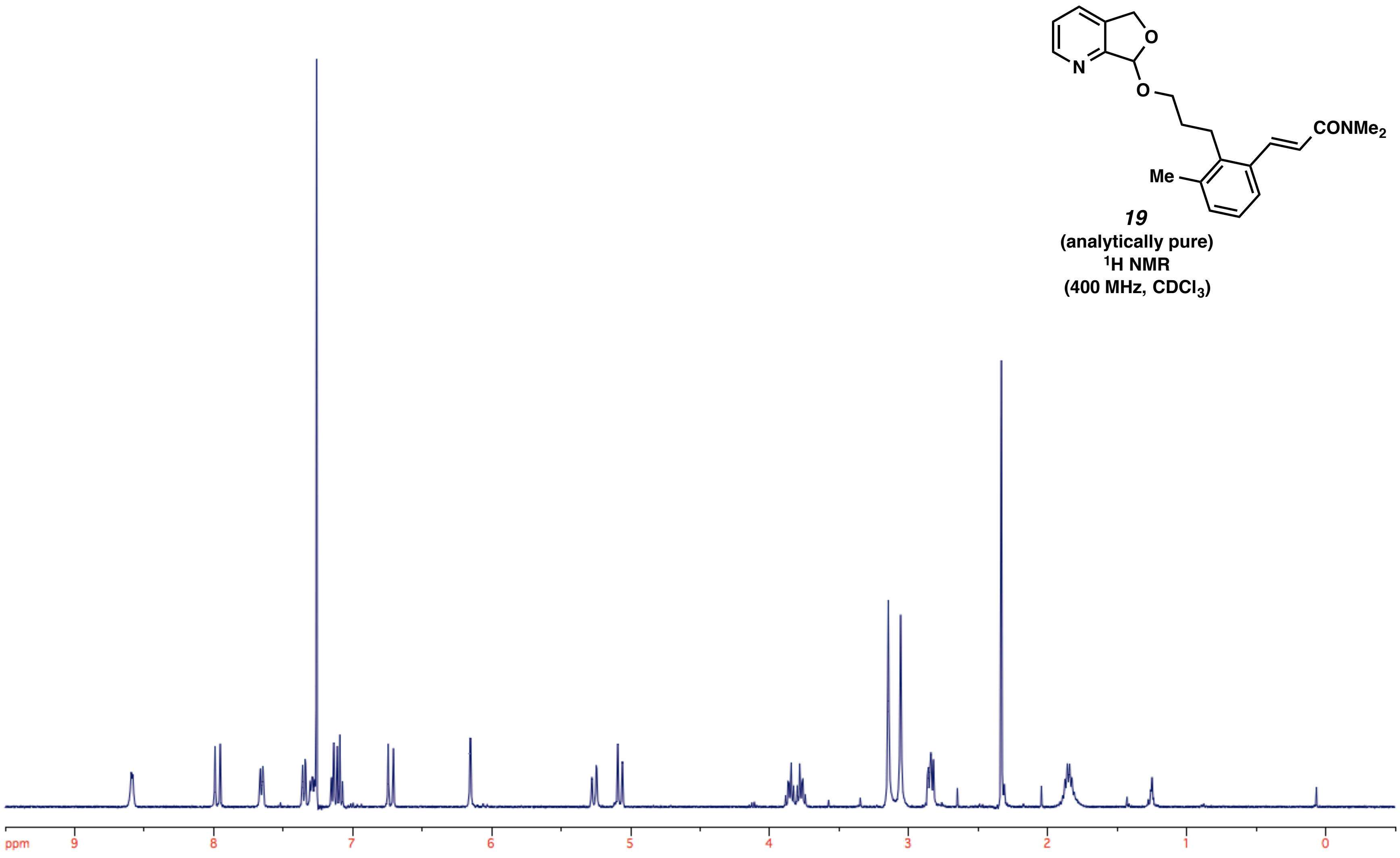

N
O
O
CONMe2
Me
19
(analytically pure)
1H NMR
(400 MHz, CDCl3)
ppm
9
8
7
6
5
4
3
2
1
0

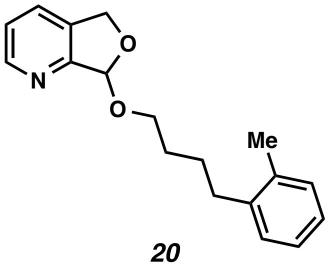

20
^{1}H NMR
(500 MHz, $CDCl_3$)

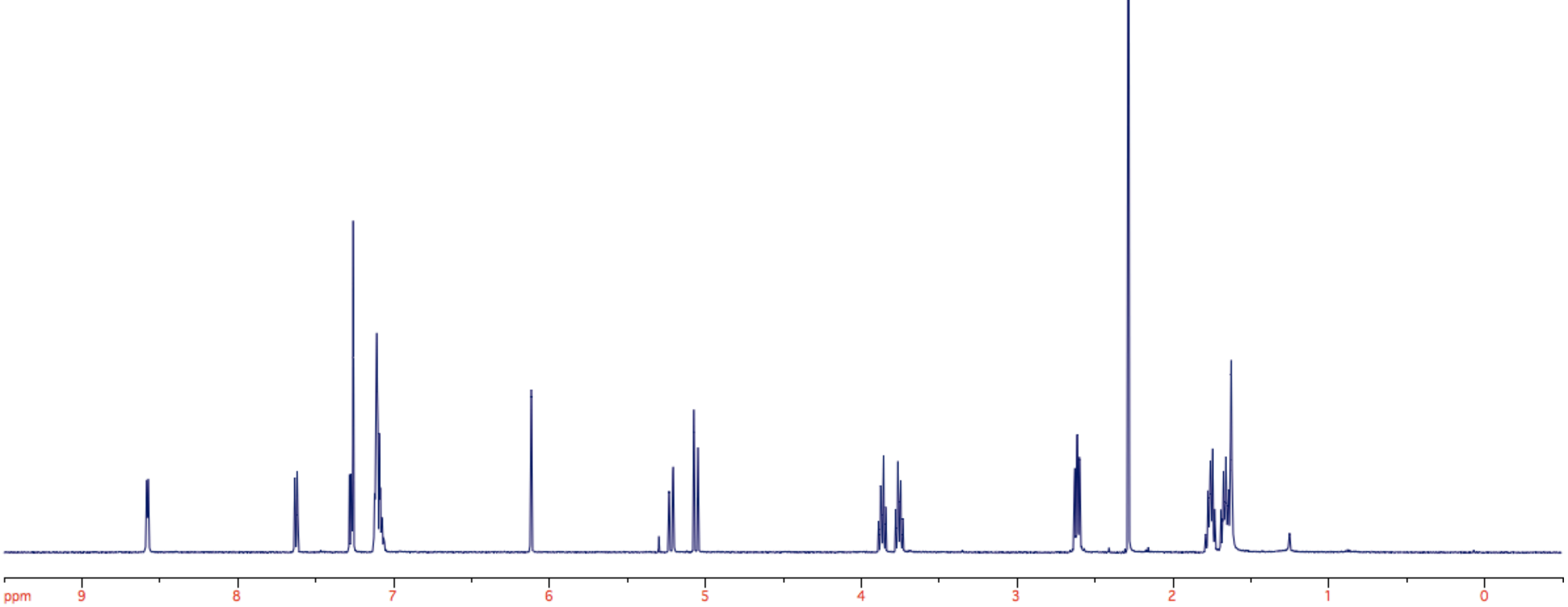

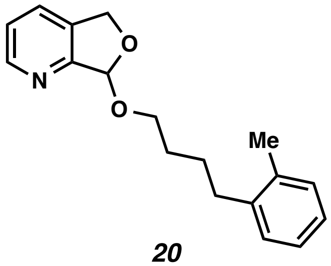

20
^{13}C NMR
(125 MHz, $CDCl_3$)

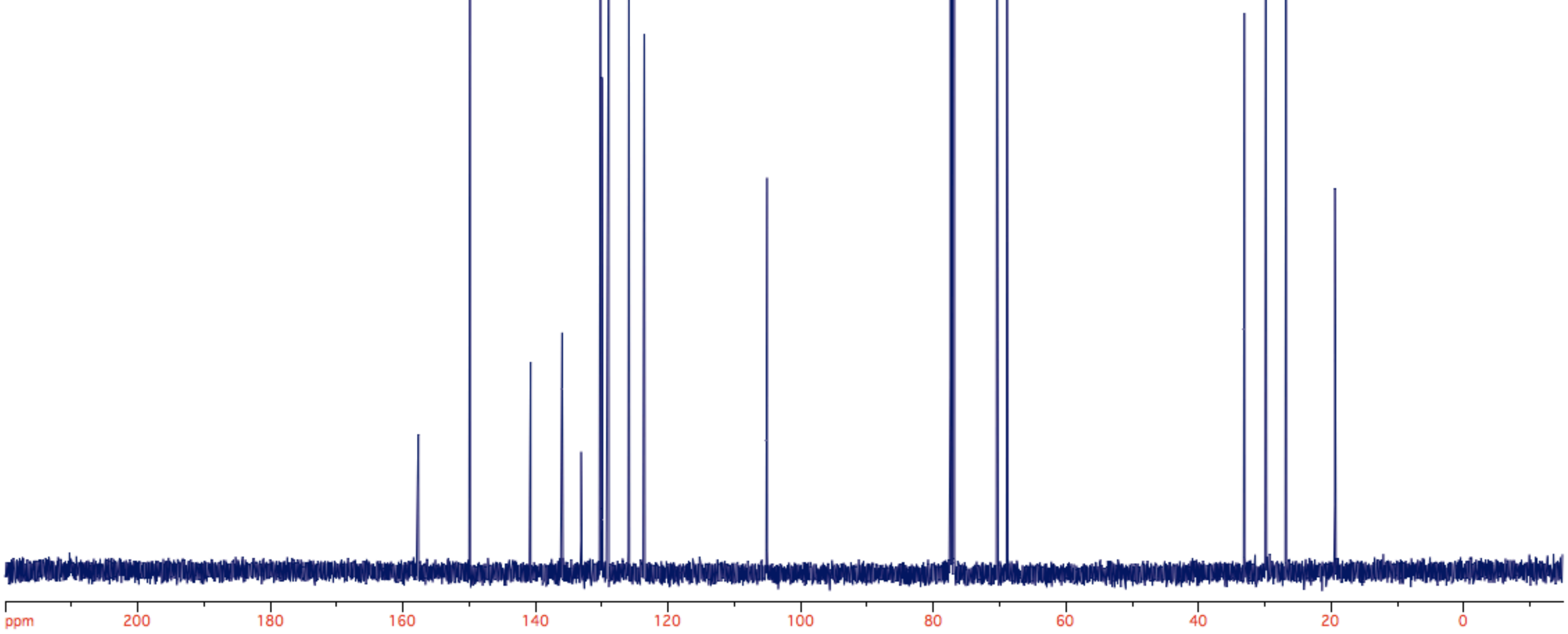

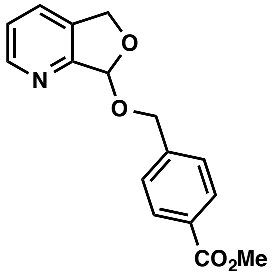

7q
^{1}H NMR
(400 MHz, $CDCl_3$)

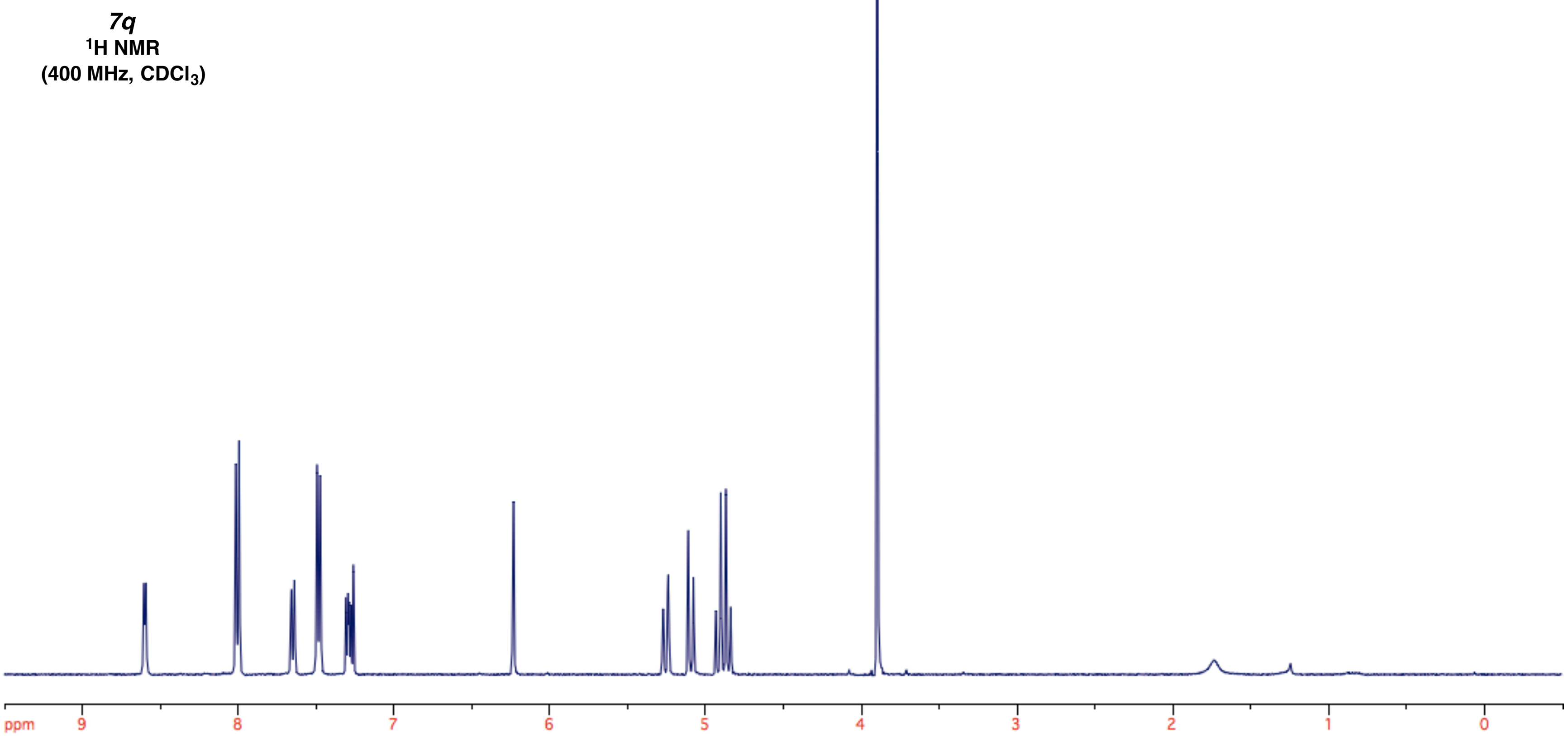

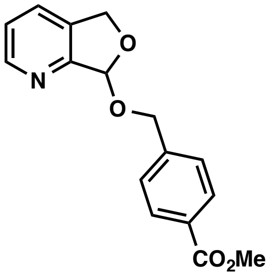

7q
^{13}C NMR
(100 MHz, $CDCl_3$)

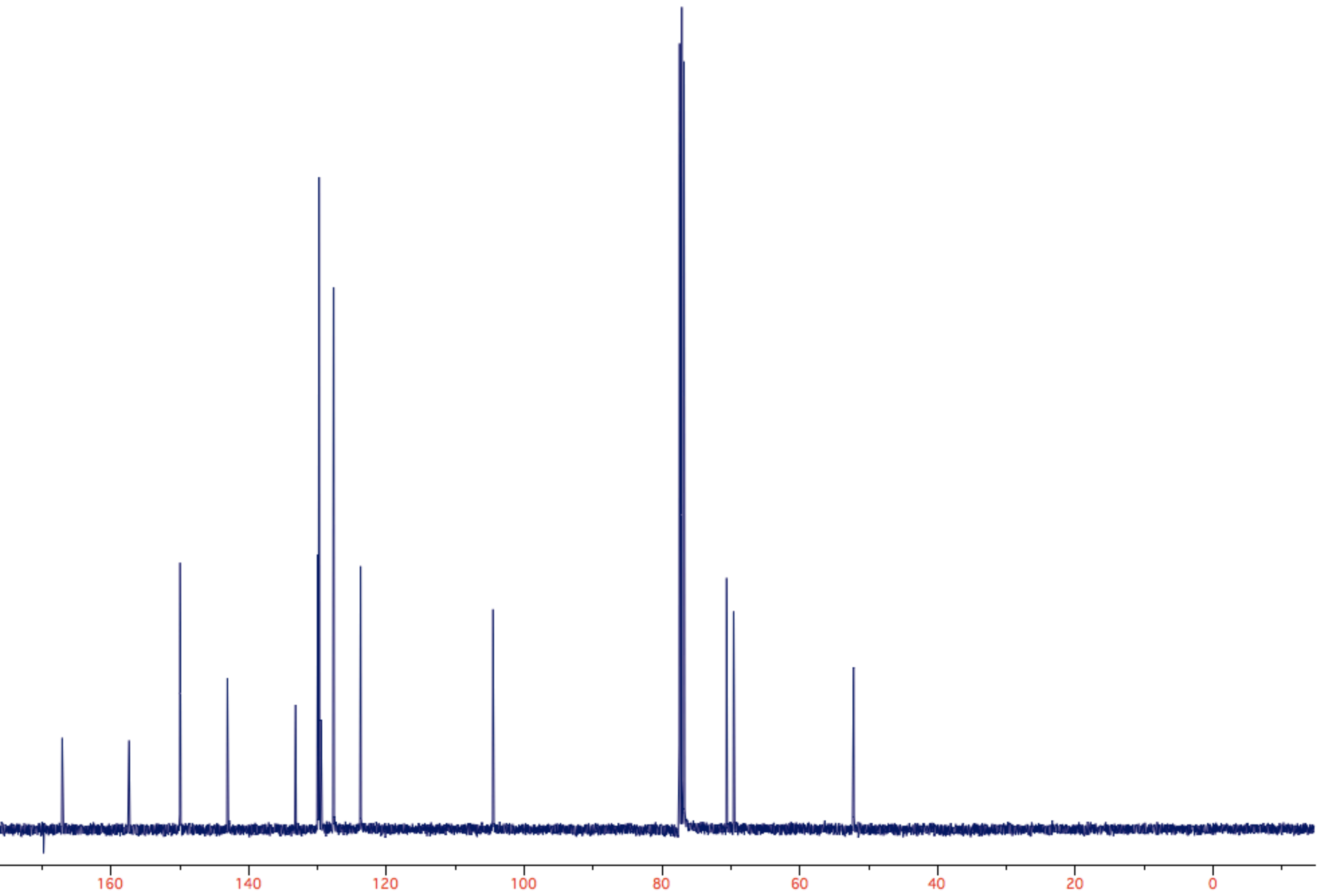

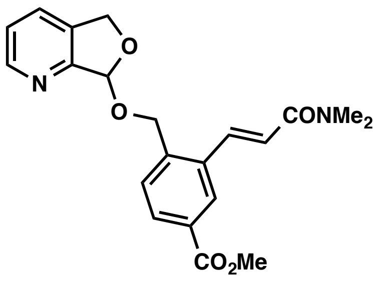

8qb$_{mono}$
^{1}H NMR
(400 MHz, $CDCl_3$)

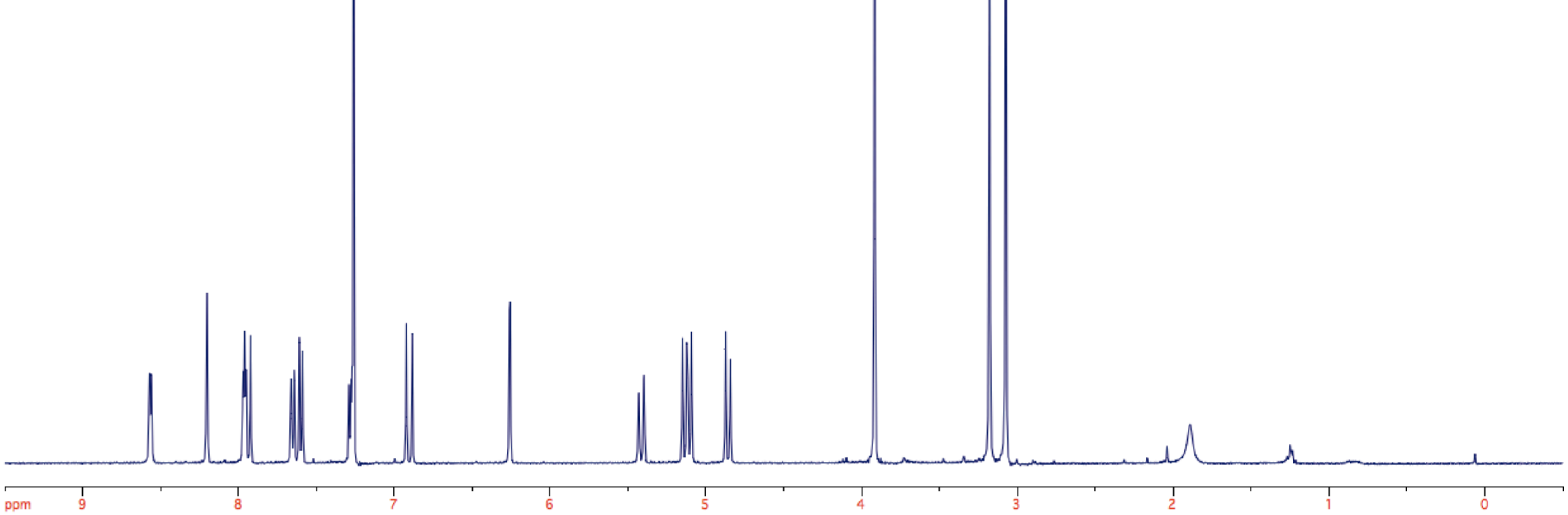

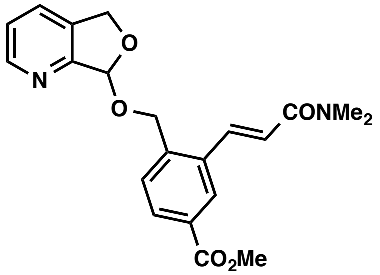

***8qb**mono*
^{13}C NMR
(100 MHz, $CDCl_3$)

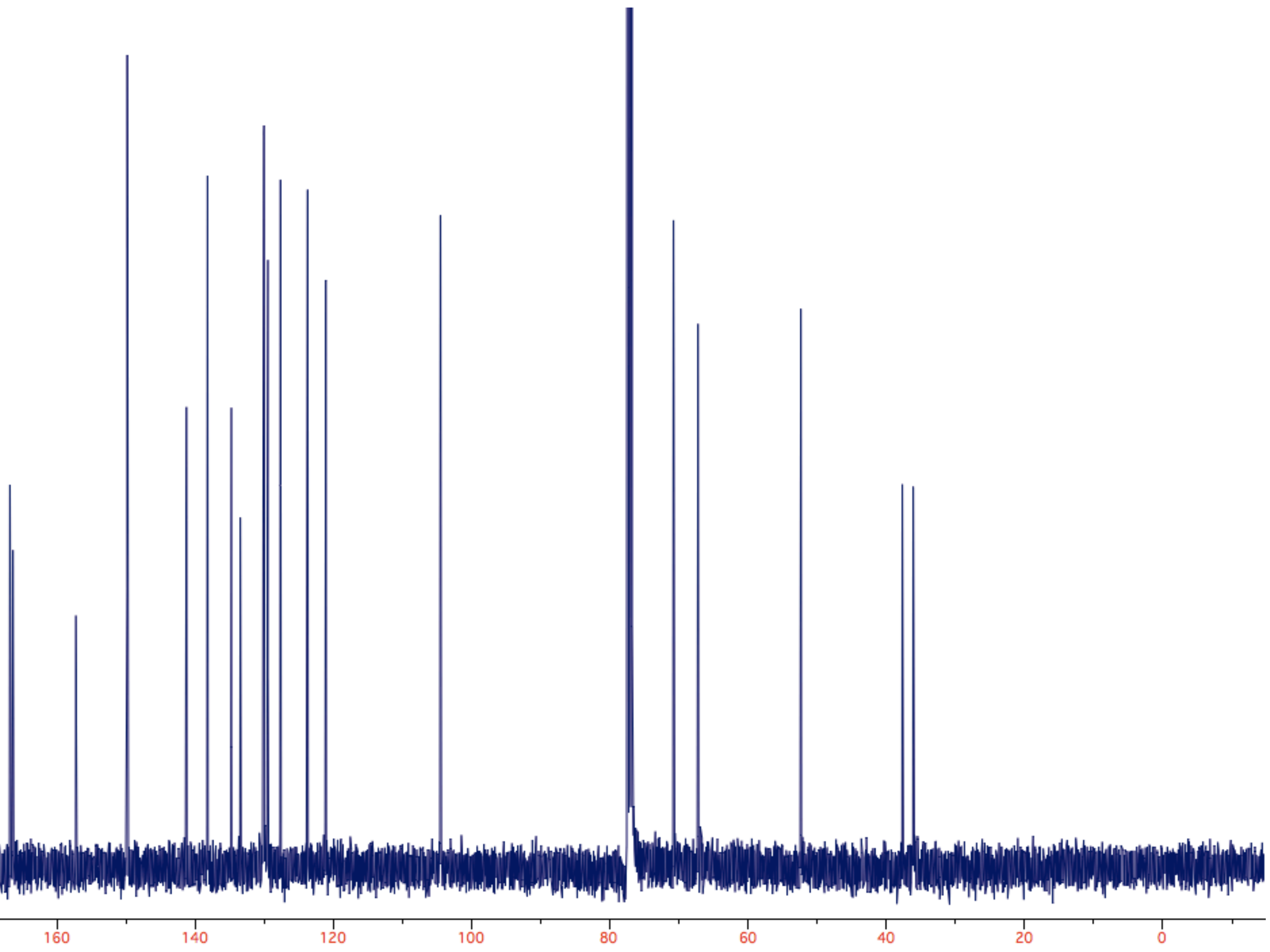

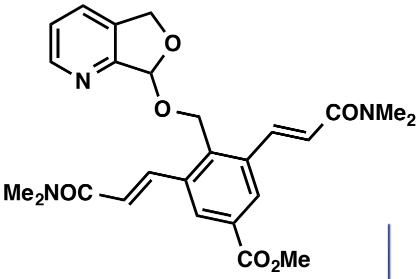

***8qb**$_{di}$*
^{1}H NMR
(400 MHz, $CDCl_3$)

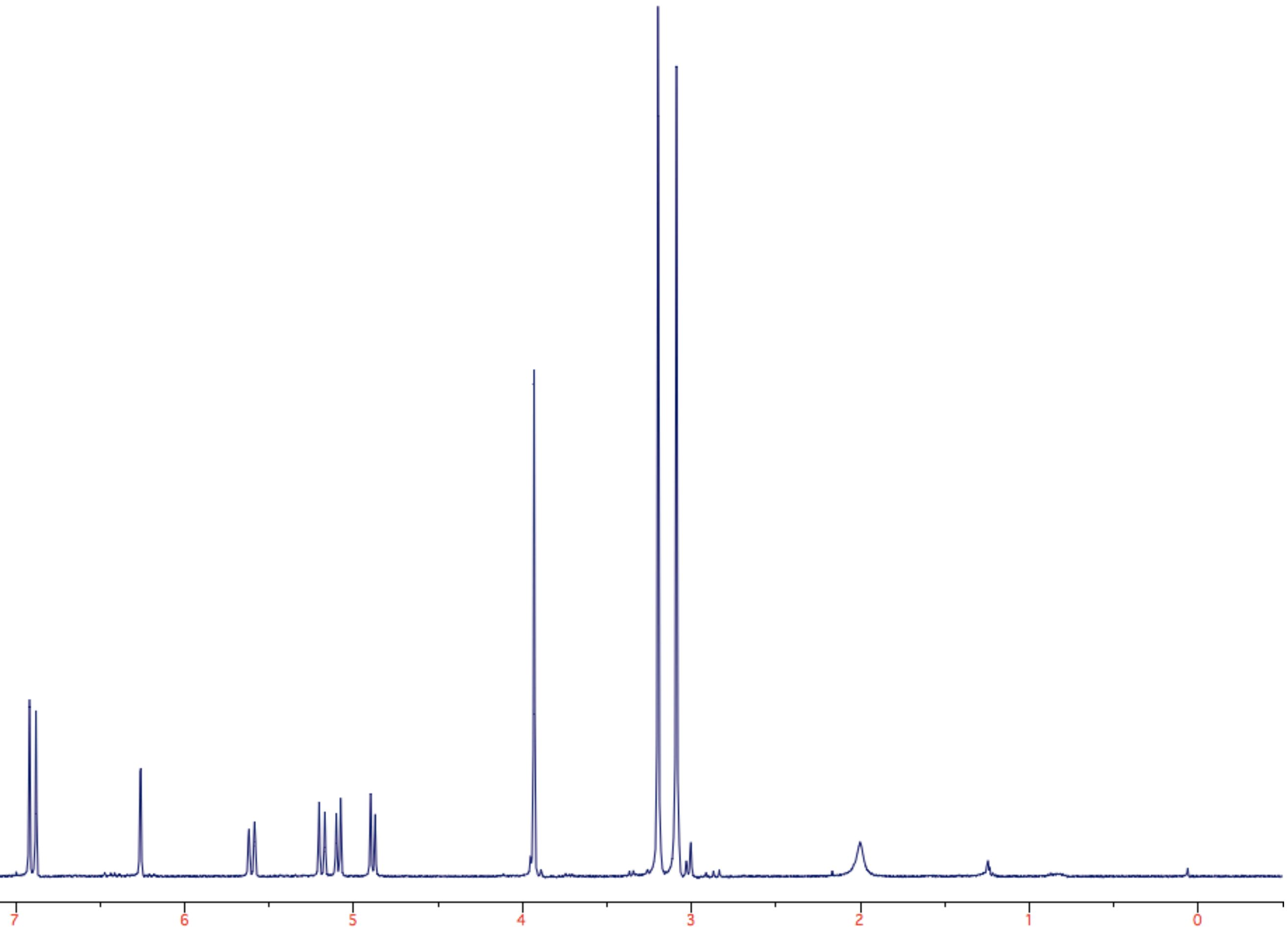

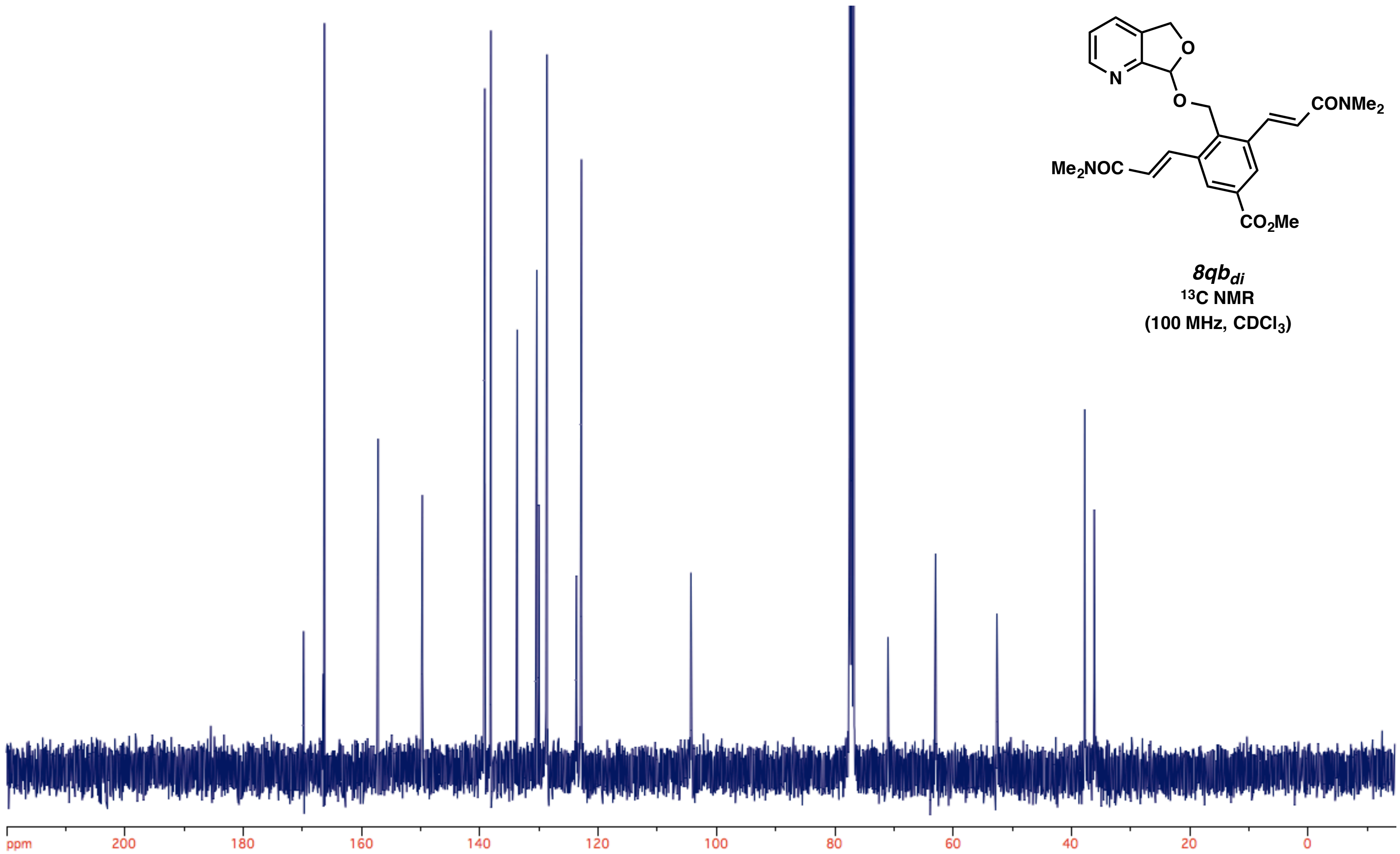
CONNMe2
Me2NOC
CO2Me
N
O
O
8qbdi
13C NMR
(100 MHz, CDCl3)
ppm
200
180
160
140
120
100
80
60
40
20
0

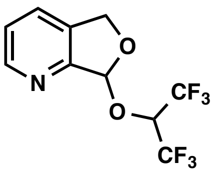

S3

^{1}H NMR

(400 MHz, $CDCl_3$)

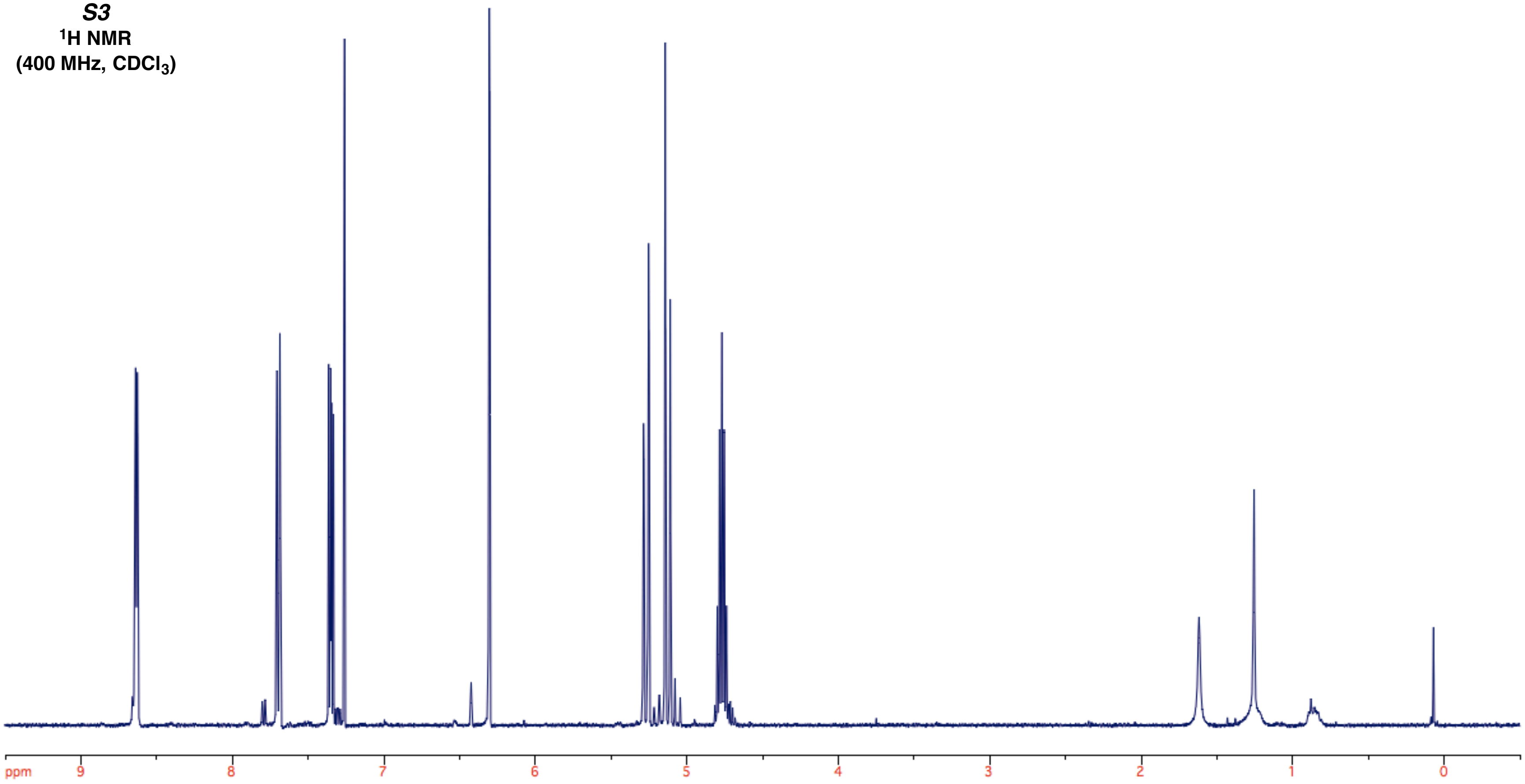

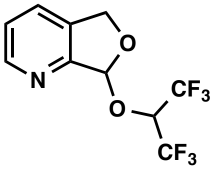

S3

^{13}C NMR

(100 MHz, $CDCl_3$)

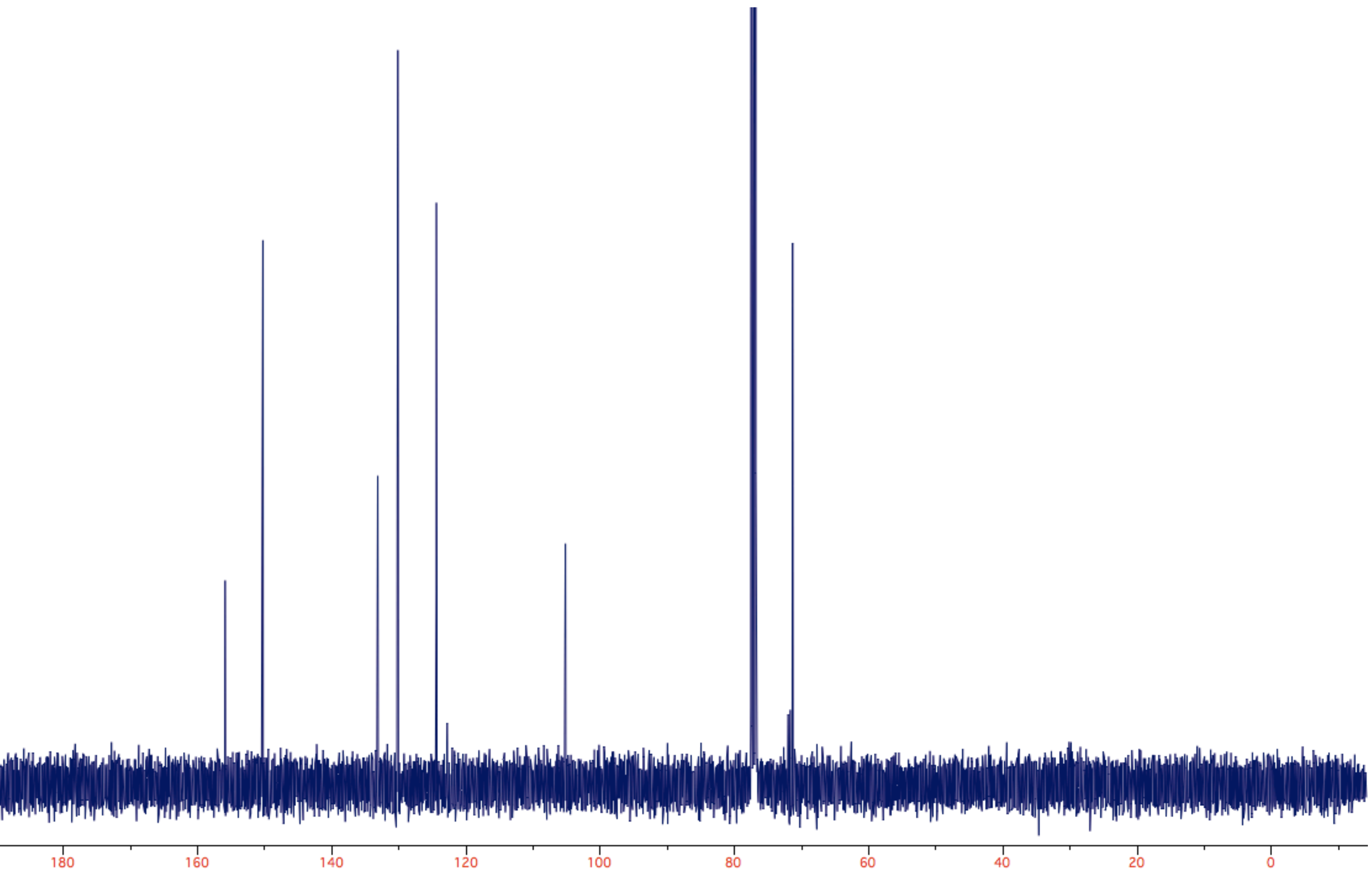

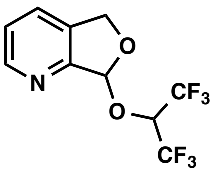

S3
^{19}F NMR
(376 MHz, $CDCl_3$)

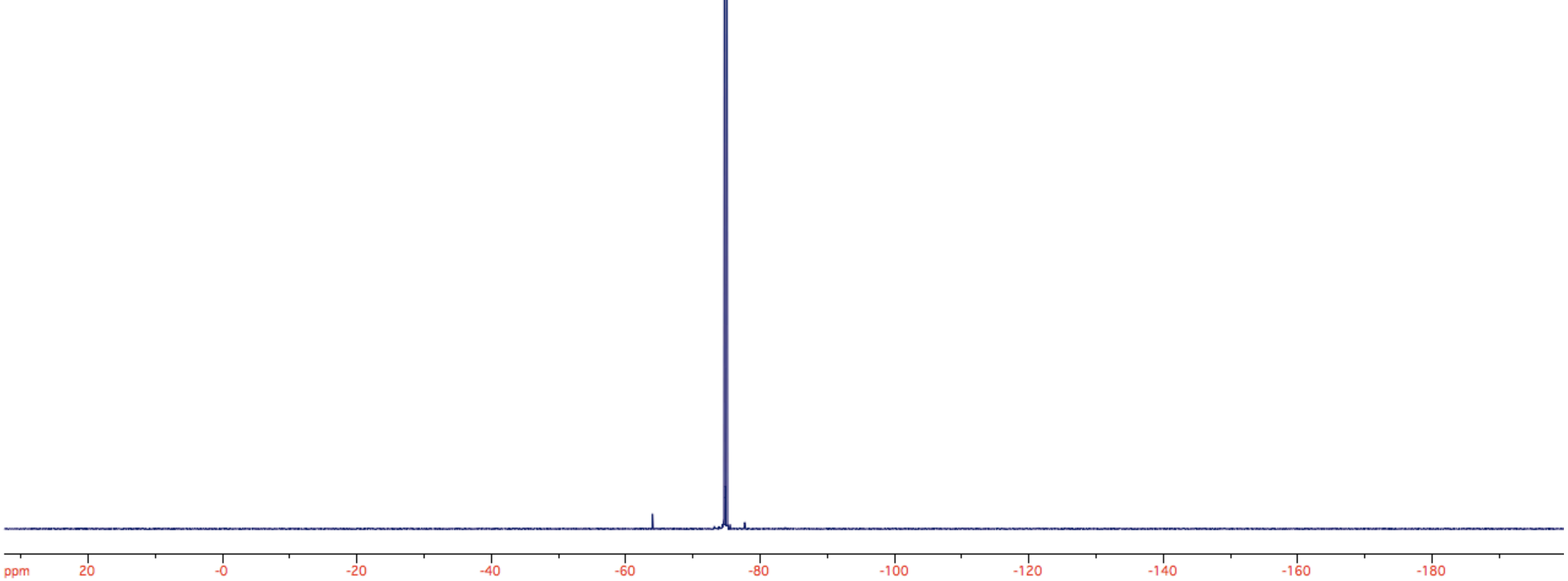

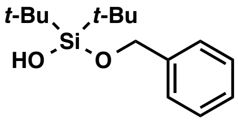

S15

^{1}H NMR

(400 MHz, $CDCl_3$)

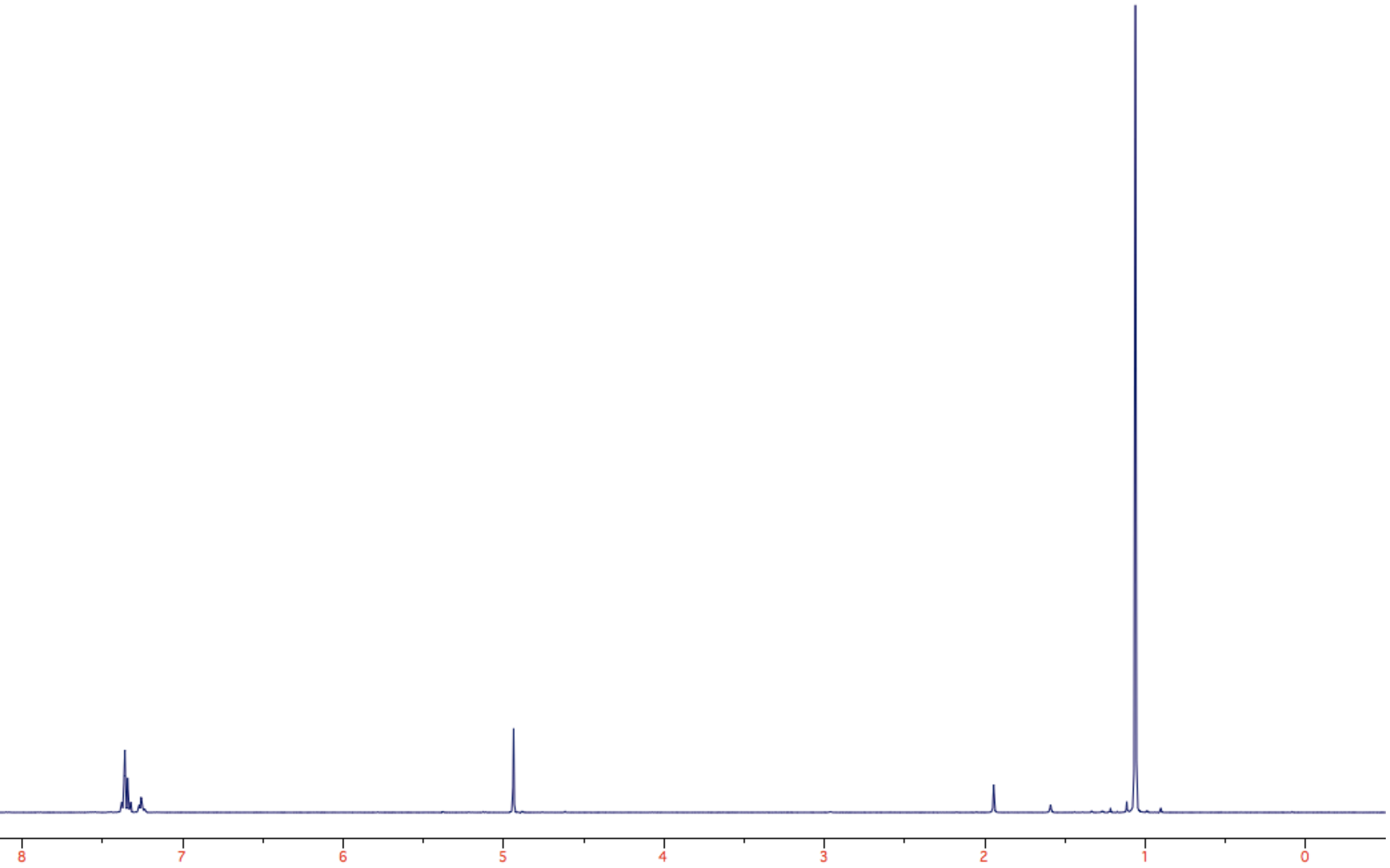

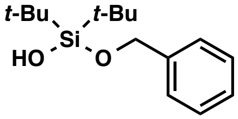

S15

^{13}C NMR

(400 MHz, $CDCl_3$)

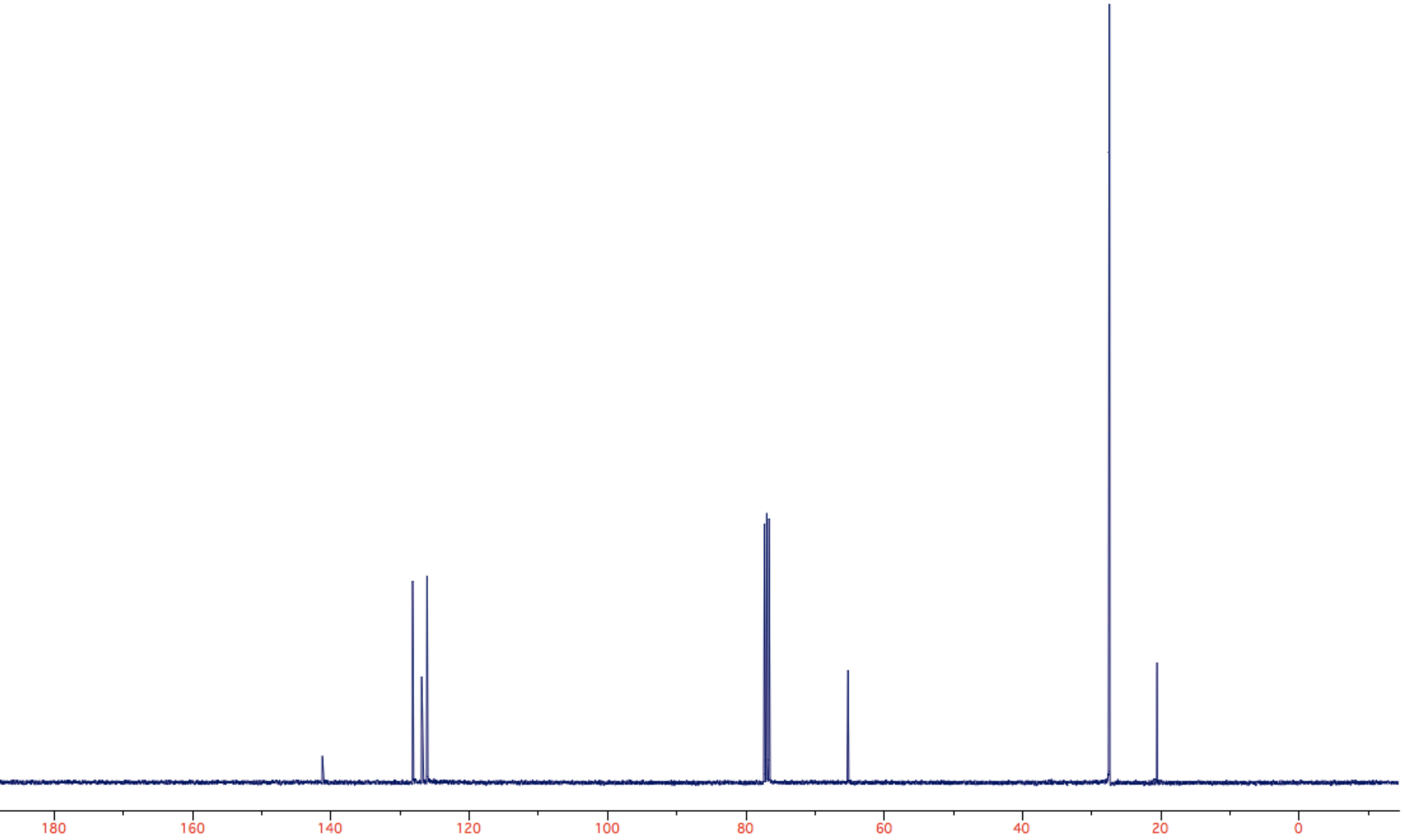

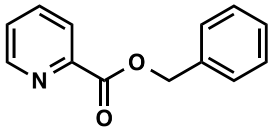

S17
^{1}H NMR
(100 MHz, $CDCl_3$)

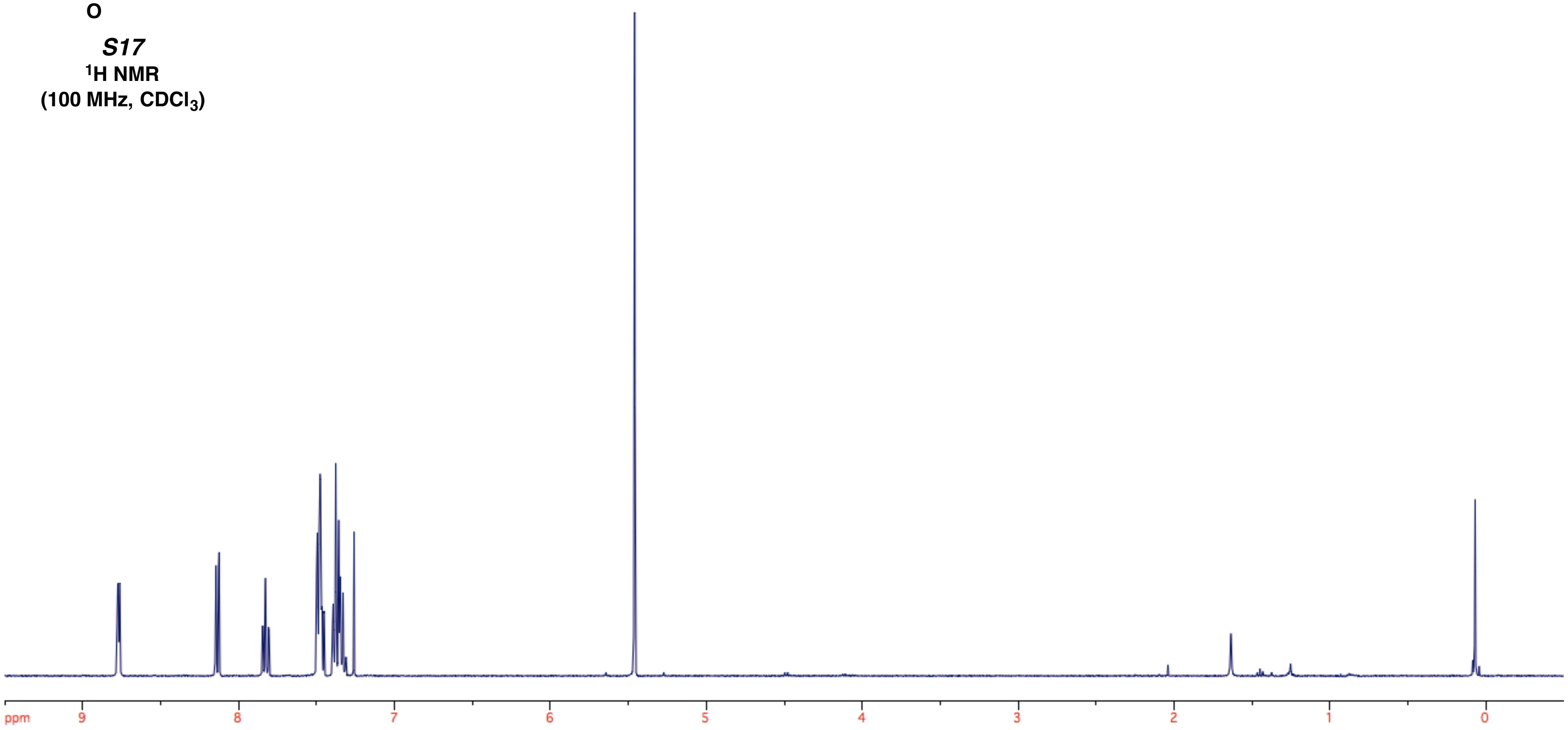

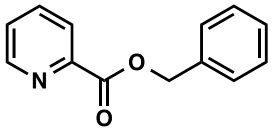

S17
^{13}C NMR
(100 MHz, $CDCl_3$)

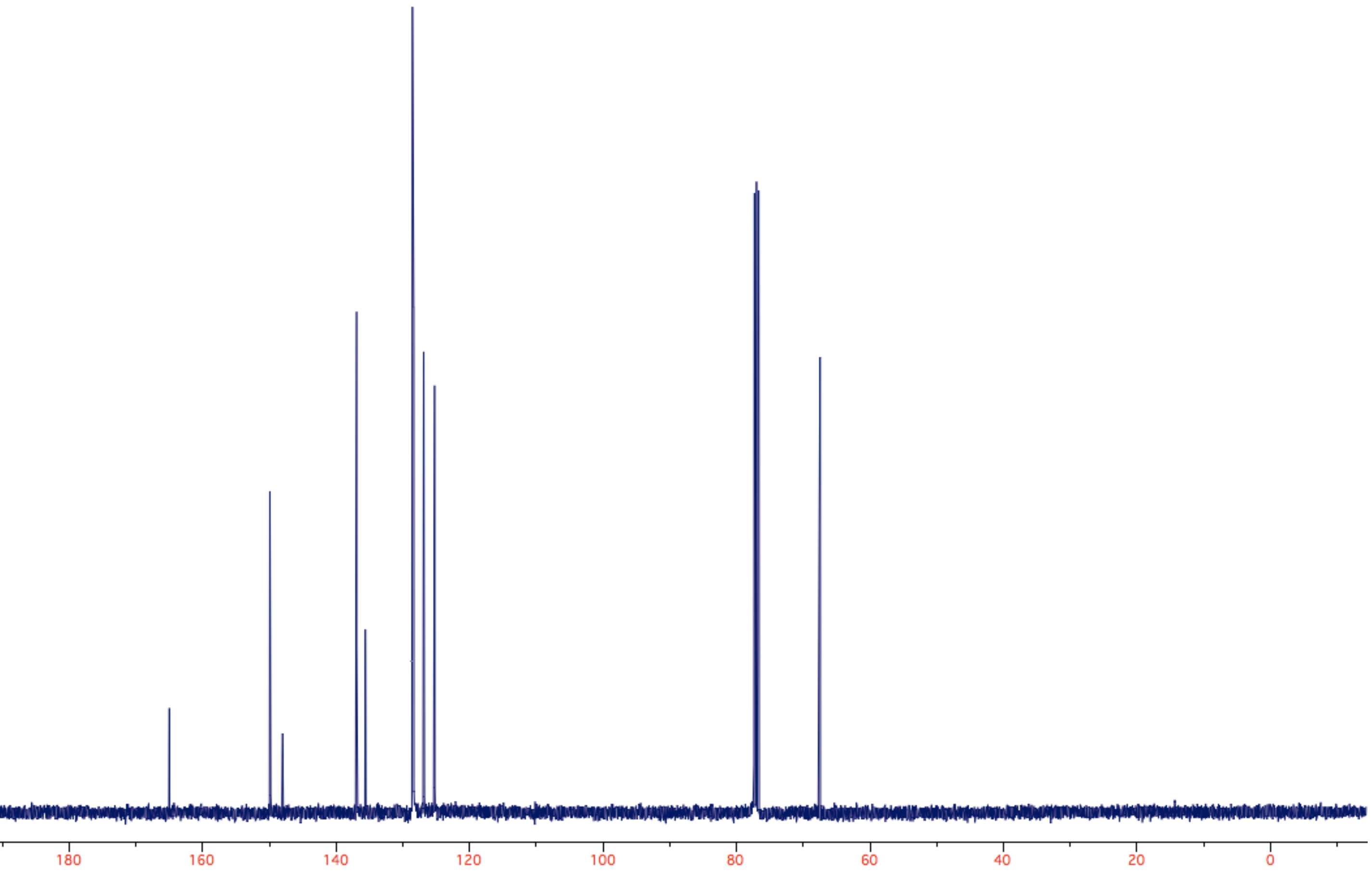

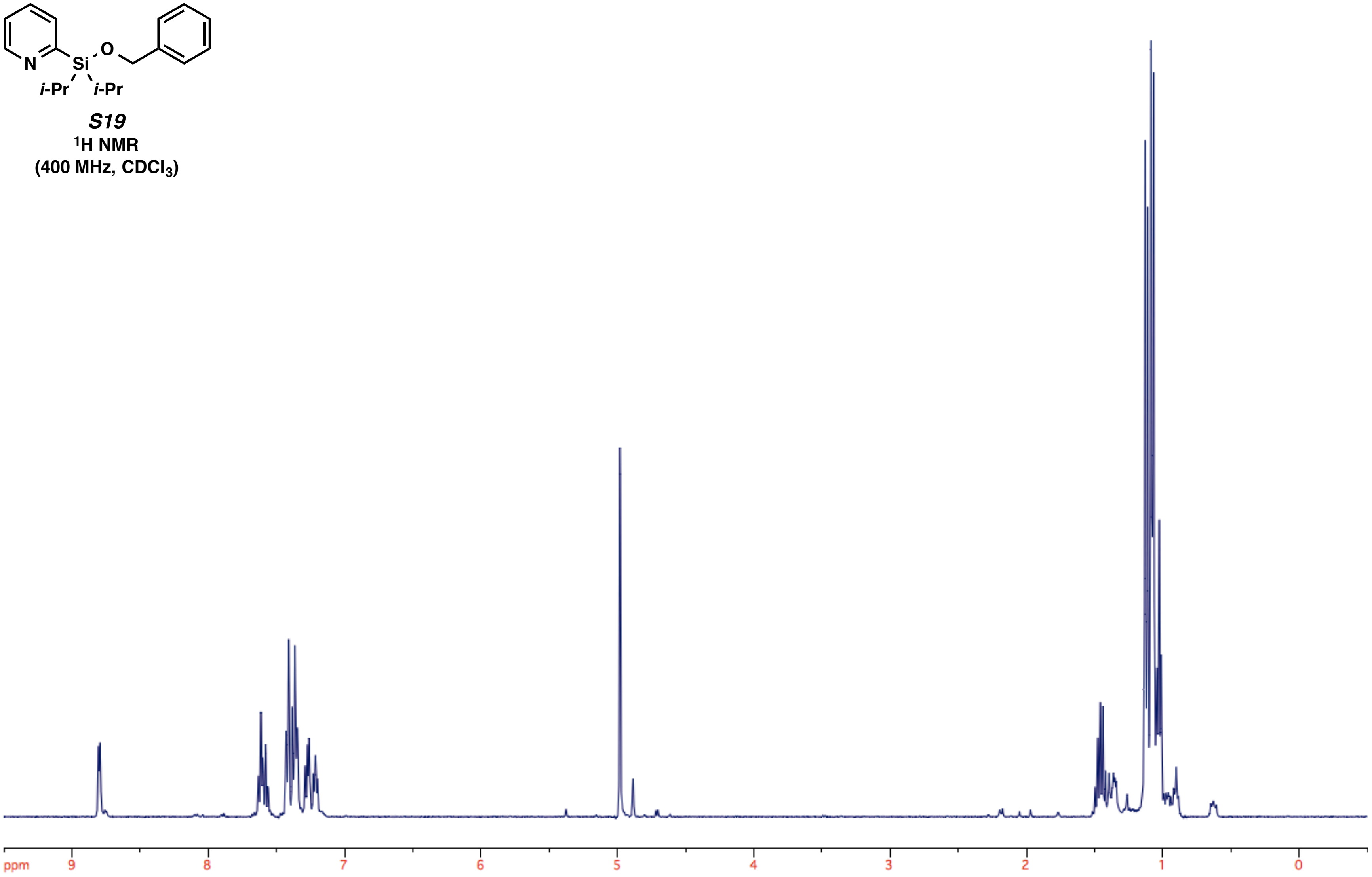
N
Si
O
i-Pr
i-Pr
S19
^{1}H NMR
(400 MHz, $CDCl_3$)
ppm
9
8
7
6
5
4
3
2
1
0

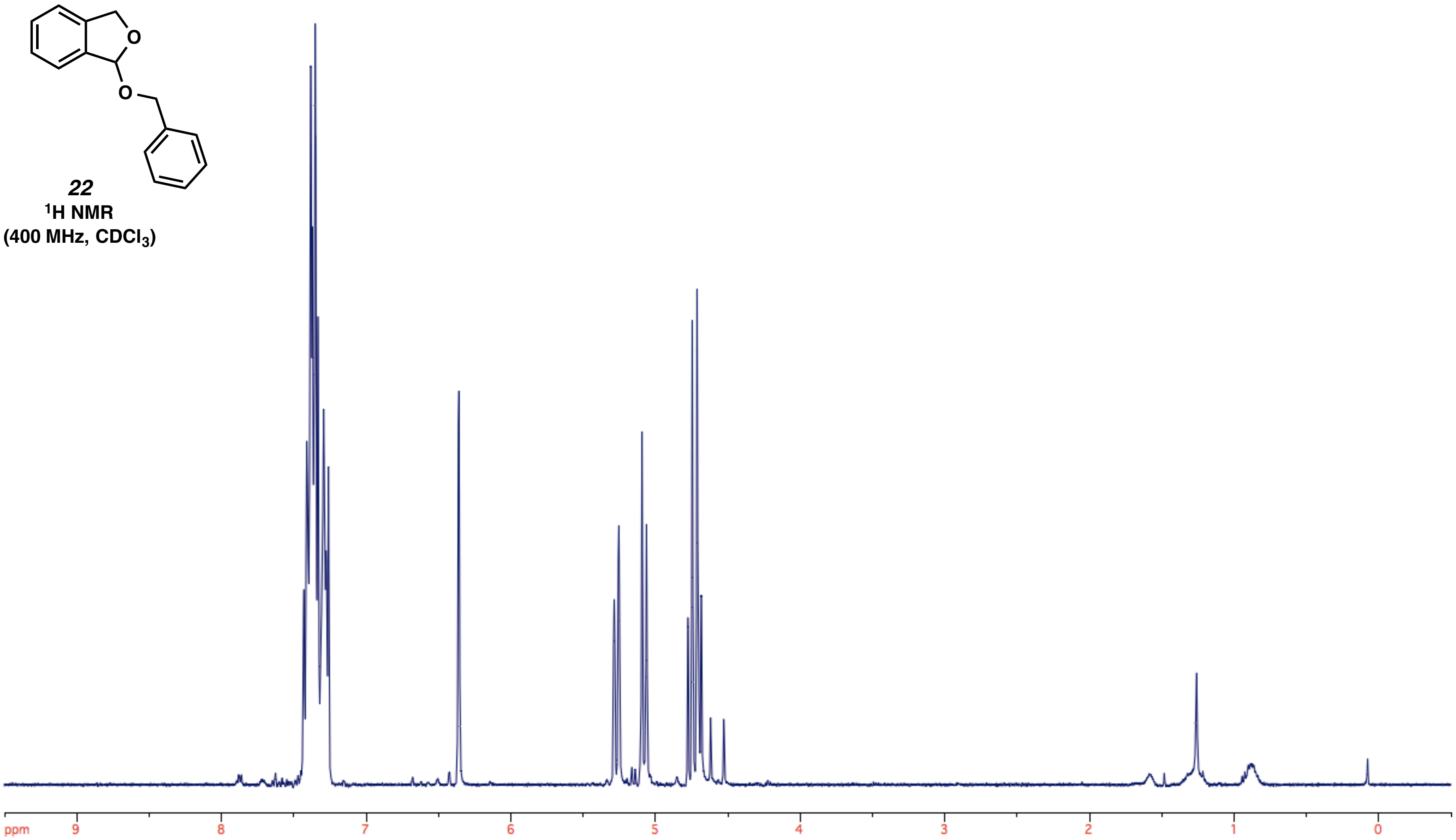

22
^{1}H NMR
(400 MHz, $CDCl_3$)

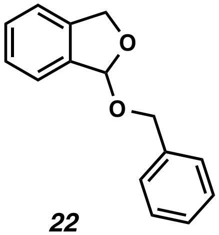

22
^{13}C NMR
(100 MHz, $CDCl_3$)

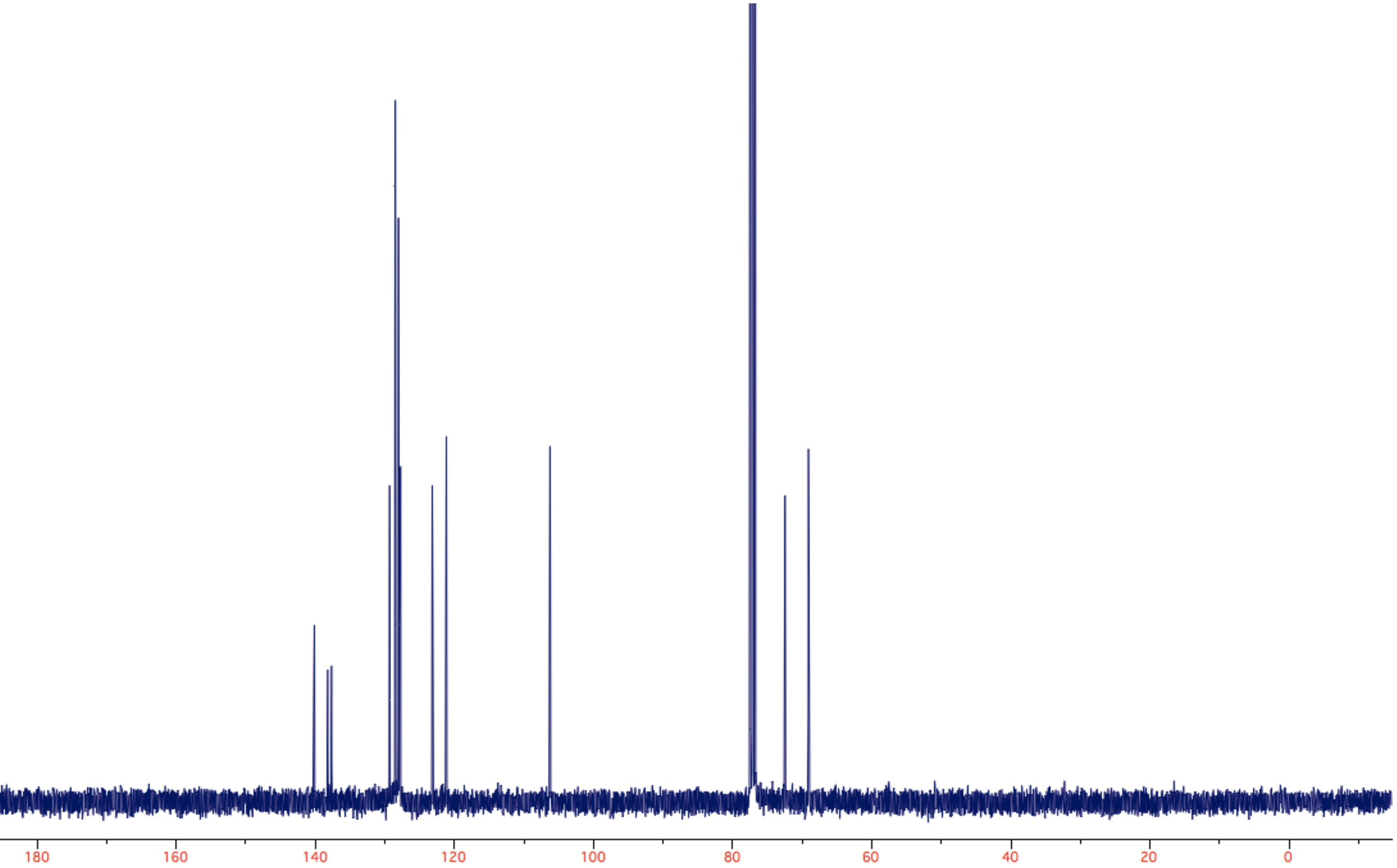

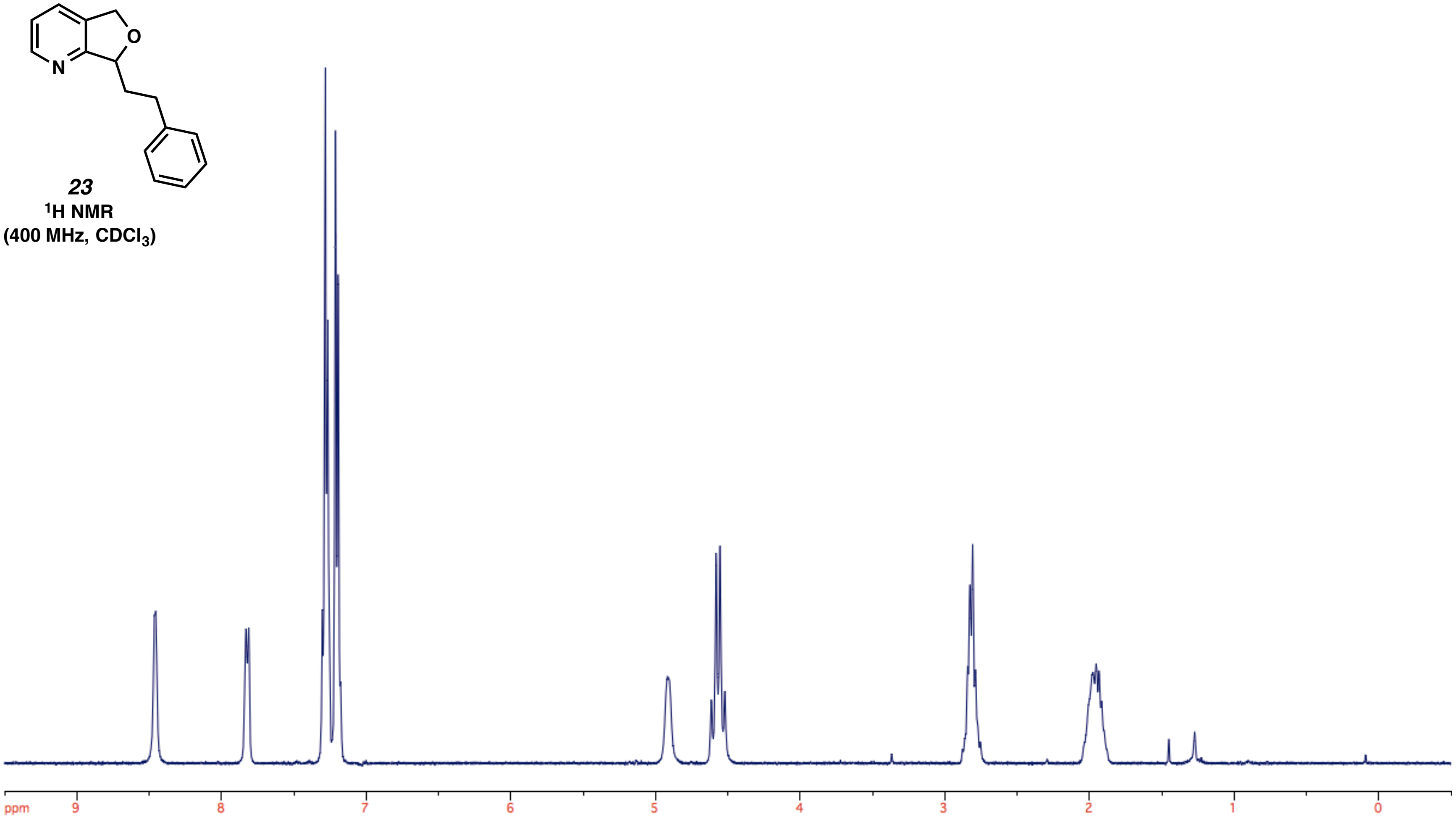

23
^{1}H NMR
(400 MHz, $CDCl_3$)

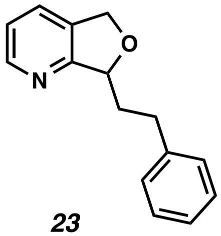

23
^{13}C NMR
(100 MHz, $CDCl_3$)

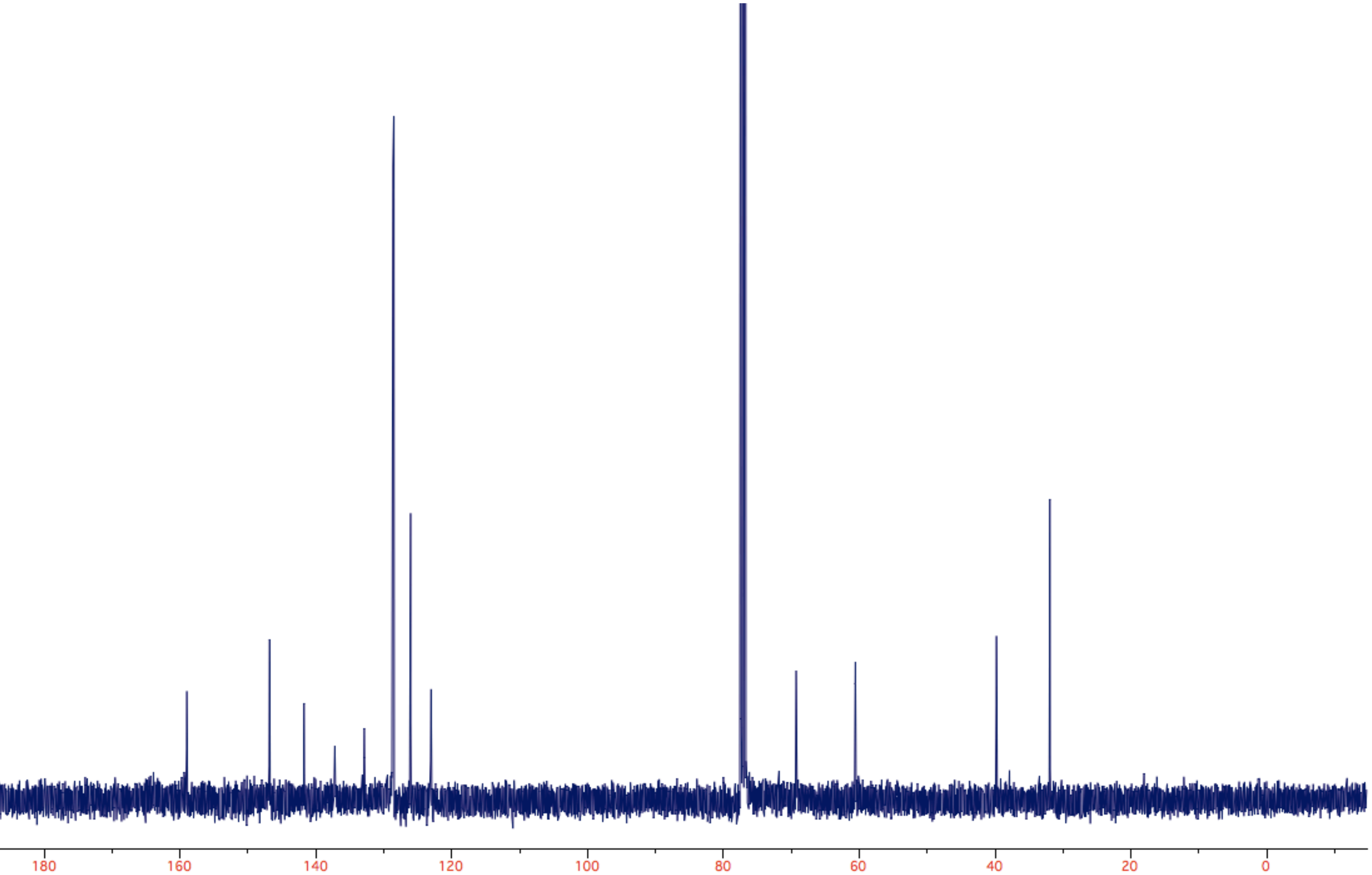

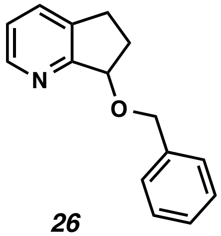

26

^{1}H NMR

(400 MHz, $CDCl_3$)

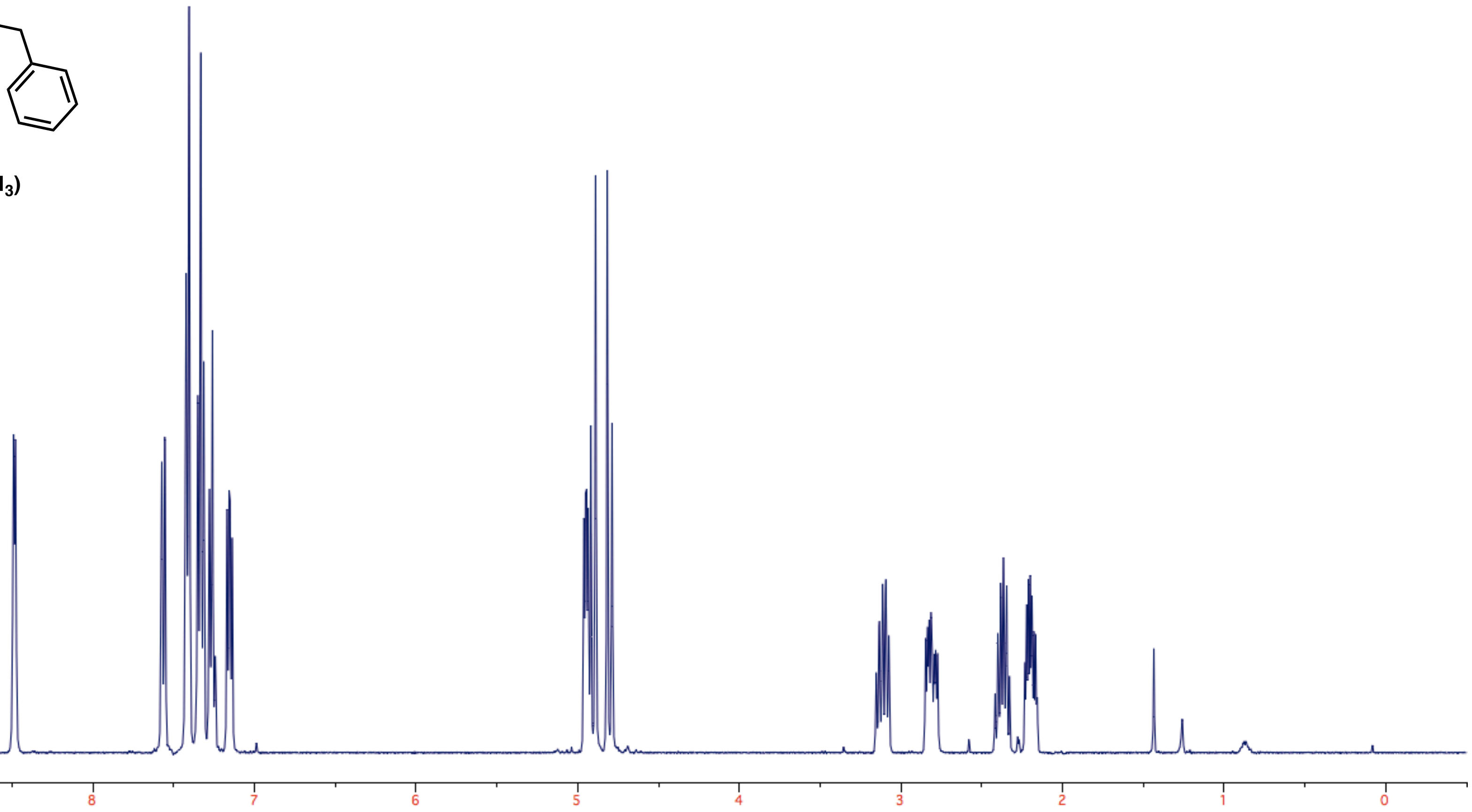

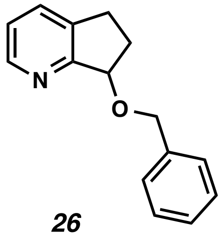

26
^{13}C NMR
(100 MHz, $CDCl_3$)

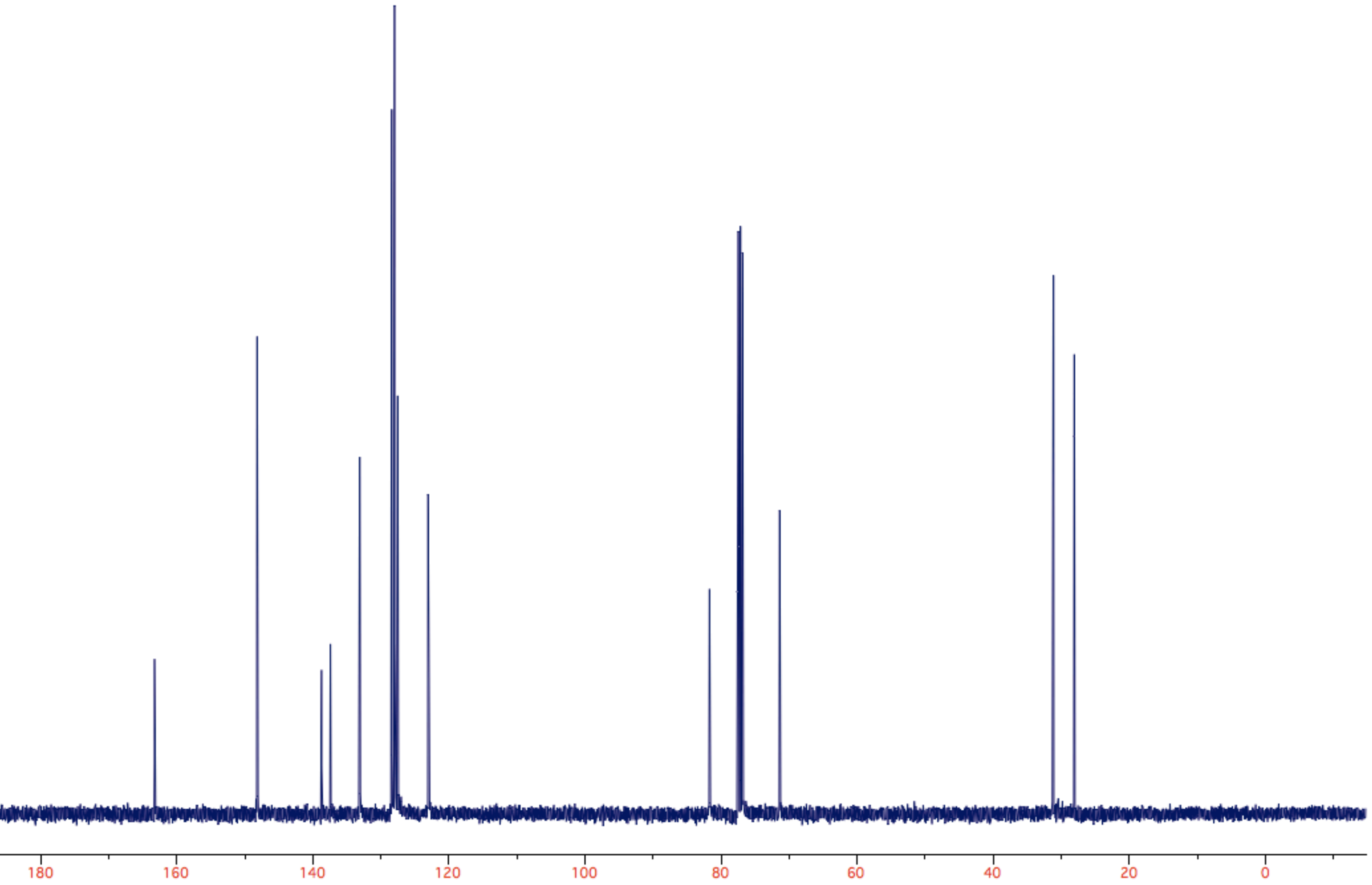

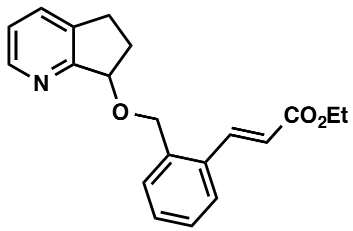

27
^{1}H NMR
(400 MHz, $CDCl_3$)
1.6:1 mono/di

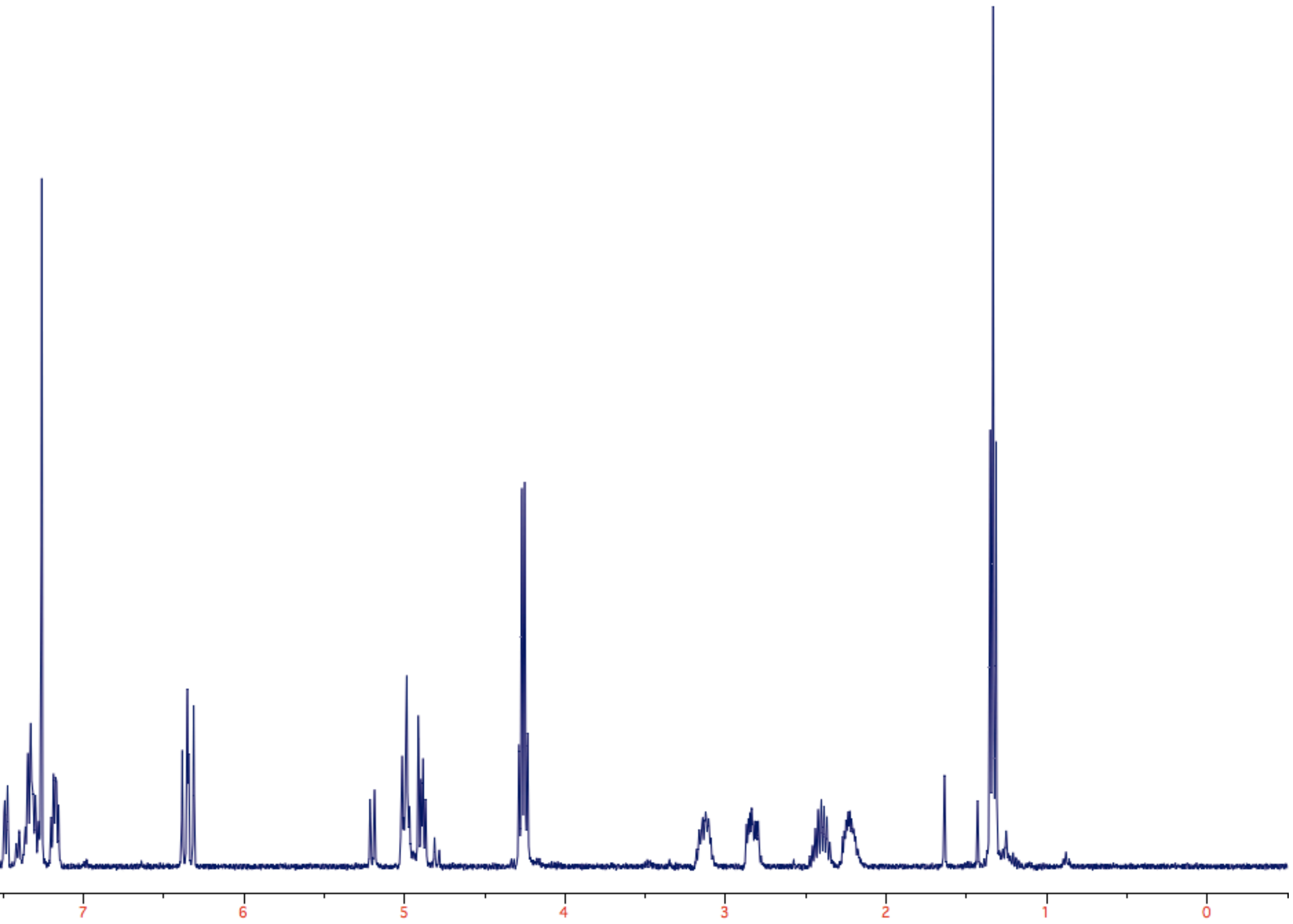

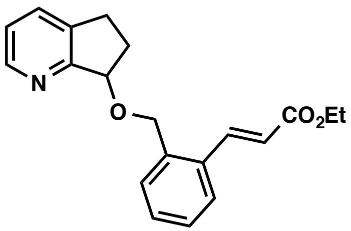

27
^{13}C NMR
(100 MHz, $CDCl_3$)
1.6:1 mono/di

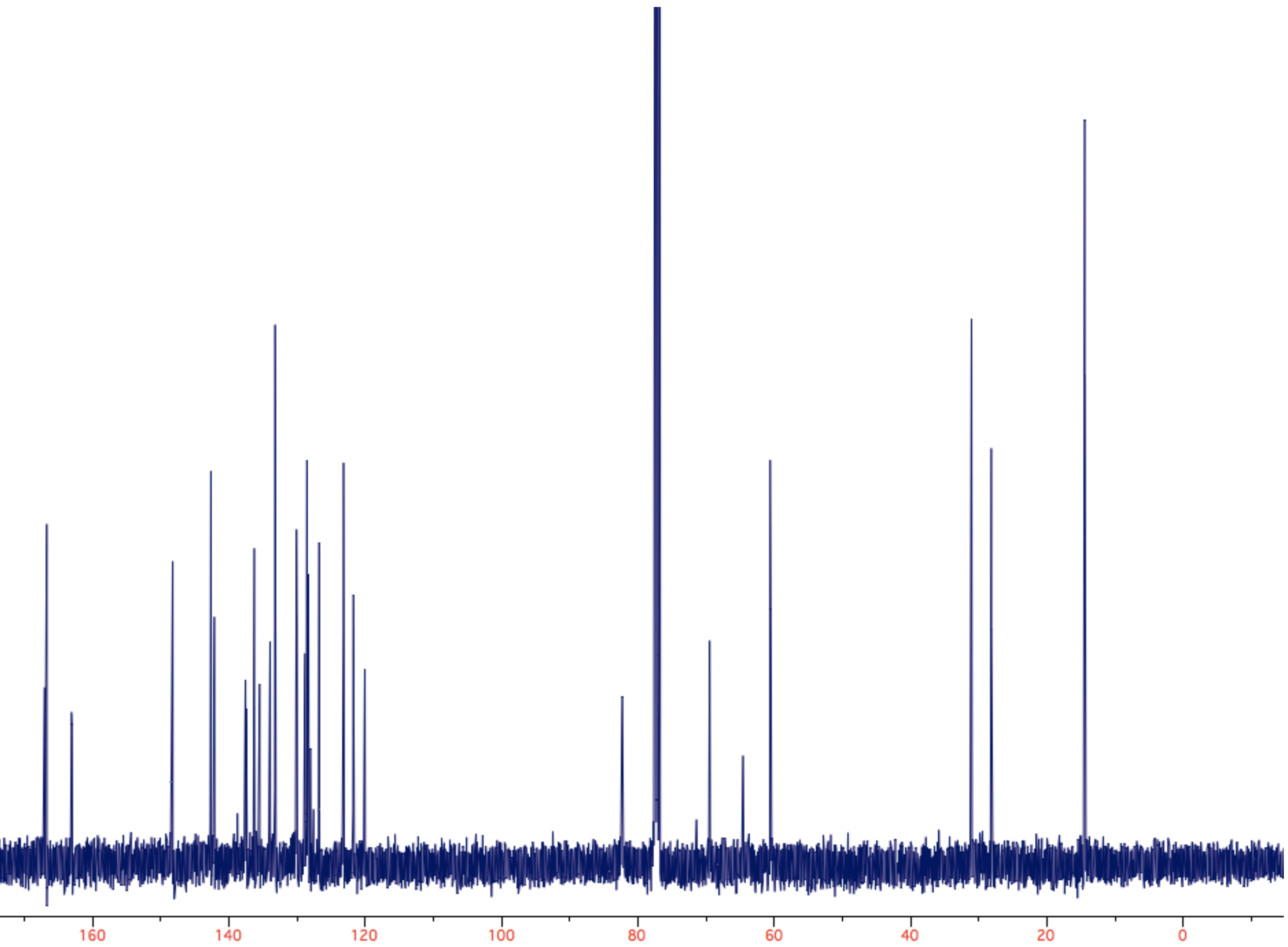

Supplement: Supplementary file 1 [file SC-007-C5SC03948G-s001.pdf]
